# Poster Session 4: Thursday, February 12, 2026 3:30 PM – 5:00 PM

**DOI:** 10.1002/pmf2.70169

**Published:** 2026-02-09

**3:30 PM – 5:00 PM**

## 998 | Iron Deficiency Without Anemia in the First Trimester: A Missed Opportunity to Prevent Anemia?

Adam K. Lewkowitz^1^; Nicole Konecke^1^; Tracy L. Jackson^1^; Irving Angeles^1^; Iman I. Sigman^1^; Crystal F. Ware^1^; Ashley E. Benson^2^; Joseph J. Shatzel^2^; Nandini Raghuraman^3^; Molly J. Stout^4^; Ann M. Bruno^5^; Steven Fein^6^; Carolyn M. Webster^7^; Michael Auerbach^8^; Jamie O. Lo^9^; Methodius G. Tuuli^10^

^1^Women & Infants Hospital of Rhode Island / Warren Alpert Medical School of Brown University, Providence, RI; ^2^Oregon Health & Science University, Portland, OR; ^3^Washington University School of Medicine, St. Louis, MO; ^4^University of Michigan, Ann Arbor, MI; ^5^University of Utah Health, Salt Lake City, UT; ^6^Heme On Call, Miami, FL; ^7^University of Alabama at Birmingham, Birmingham, AL; ^8^Georgetown University School of Medicine, Washington DC, DC; ^9^Oregon Health & Science University, Oregon Health & Science University, OR; ^10^Department of Obstetrics and Gynecology, Warren Alpert Medical School at Brown University, and Women & Infants Hospital of Rhode Island, Providence, RI

**Objective**: First‐trimester iron deficiency without anemia (IDWA: ferritin <30 ng/mL with hemoglobin (Hgb) >11 g/dL) may progress to iron deficiency anemia (IDA), which is associated with adverse perinatal outcomes for mother and infant. We aimed to examine the association between first trimester IDWA and development of IDA later in pregnancy.

**Study Design**: This was a single center prospective cohort study of individuals with singleton gestations who completed their routine obstetric intake labs—which include CBC and ferritin—in the first trimester from August 2022 to December 2024 Those with first‐trimester IDA (ferritin <30 ng/mL and Hgb<11 g/dL) were excluded. Individuals with IDWA were compared with non‐anemic, non‐iron‐deficient people (ferritin >30 ng/mL and Hgb > 11). The primary outcome was progression to IDA after 14 weeks’ gestation (  >14 wks). Relative risks (RR) were calculated adjusting for receiving iron therapy and race/ethnicity. A subgroup analysis included those in either group who did not receive iron therapy.

**Results**: Among 514 eligible individuals, 136 (26%) had first‐trimester IDWA. Those with IDWA were more likely to be Hispanic or Native American than those who were non‐anemic and non‐iron deficient, but age, parity, and body mass index were similar between groups (Table 1). Progression to IDA requiring treatment >14 wks was higher among those with IDWA than those who were non‐anemic, non‐iron deficient (59% versus 33%; *p* < 0.001). Among those who did not receive iron therapy, people with IDWA were nearly four times more likely to develop IDA >14 wks compared with those who were non‐anemic, non‐iron deficient (16% vs 4%; RR 3.64 (95% CI 1.58, 8.36)) (Table 2).

**Conclusion**: First‐trimester IDWA is common and associated with high likelihood of requiring IDA treatment prior to delivery, and those who are untreated have a nearly four‐fold increased risk of developing IDA compared to non‐anemic, non‐iron‐deficient individuals. These findings suggest that identifying iron deficiency in the first trimester provides an opportunity to prevent overt IDA.

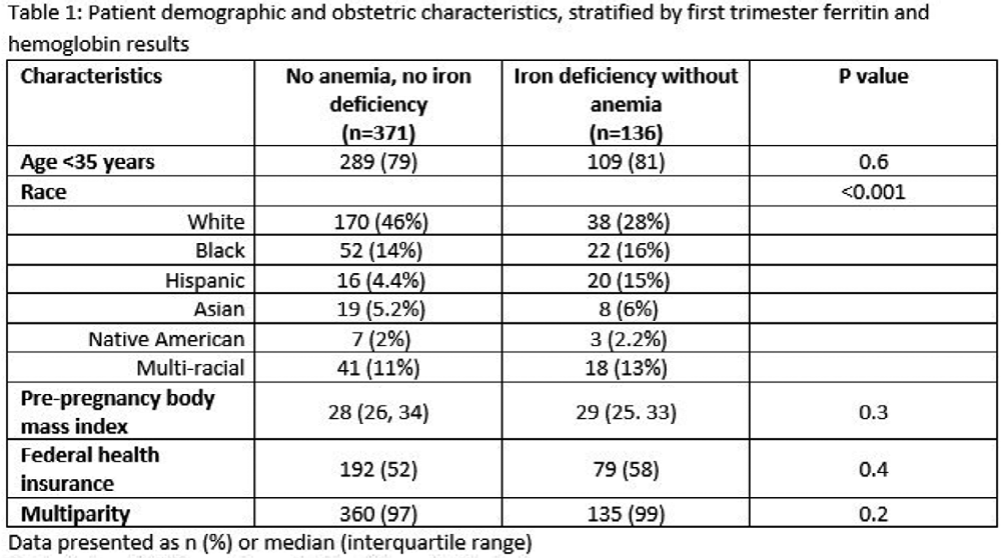

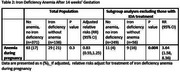

**3:30 PM – 5:00 PM**

## 999 | **Association Between Preconception GLP‐1 Use in Patients Without Diabetes and Risk of Gestational Diabetes**

Adina R. Kern‐Goldberger^1^; Marissa A. Hand^2^; Easha Patel^3^; Cara D. Dolin^4^; Sarah Nazeer^5^; Jodi Meridieth^6^; Stacey Ehrenberg^7^

^1^Cleveland Clinic Lerner College of Medicine, Cleveland, OH; ^2^Cleveland Clinic Foundation, Cleveland Clinic Foundation, Cleveland, OH; ^3^Cleveland Clinic Foundation, Cleveland, OH; ^4^Cleveland Clinic, Clevelend, OH; ^5^Cleveland Clinic Lerner College of Medicine, Cleveland Clinic Lerner College of Medicine, OH; ^6^Cleveland Clinic, Cleveland, OH; ^7^Cleveland Clinic Foundation, Cleveland Clinic Foundation, OH

**Objective**: Glucagon‐like peptide‐1 (GLP‐1) receptor agonists are a novel treatment for obesity and may be used by reproductive age patients. This study evaluates the association between GLP‐1 agonist use in patients without diabetes prior to pregnancy and risk of gestational diabetes (GDM).

**Study Design**: This is a retrospective cohort study of patients delivering in a large health system from 1/1/2021‐6/30/2025 with BMI >30. All deliveries at > 20 weeks in patients without a diagnosis of pre‐gestational diabetes were included. Patient demographic and clinical characteristics, including prescriptions for the 6 months preceding pregnancy, were extracted from the EHR and compared in bivariable analyses. The primary exposure was a prescription for a GLP‐1 receptor agonist in the 6 months prior to pregnancy. The association of preconception GLP‐1 use with the primary outcome (incidence of GDM) and secondary outcomes (GDM requiring insulin, cesarean delivery, and shoulder dystocia) was assessed with multivariable logistic regression.

**Results**: 8213 patients were included, 208 of which were prescribed GLP‐1 receptor agonists prior to pregnancy (2.5%). Patients prescribed GLP‐1 receptor agonists tended to be older and have higher pregravid and delivery BMIs as well as higher rates of bariatric surgery and chronic hypertension [Table 1]. They also had earlier presentation to prenatal care. Adjusted analysis demonstrated no significant association between GDM and GLP‐1 use, though GDM requiring insulin was marginally associated (aOR 1.32, 95% CI 0.93‐1.89; aOR 1.52, 95% CI 1.01‐2.38, respectively) [Table 2]. There were no significant differences in the other secondary outcomes.

**Conclusion**: Despite higher prevalence of risk factors for GDM such as higher BMI and older age in patients prescribed GLP‐1 receptor agonists, odds of GDM were not increased. This may suggest a protective effect of pre‐pregnancy GLP‐1 receptor agonist use which requires further detailed study to elucidate opportunities to optimize pregnancy outcomes for patients with obesity.

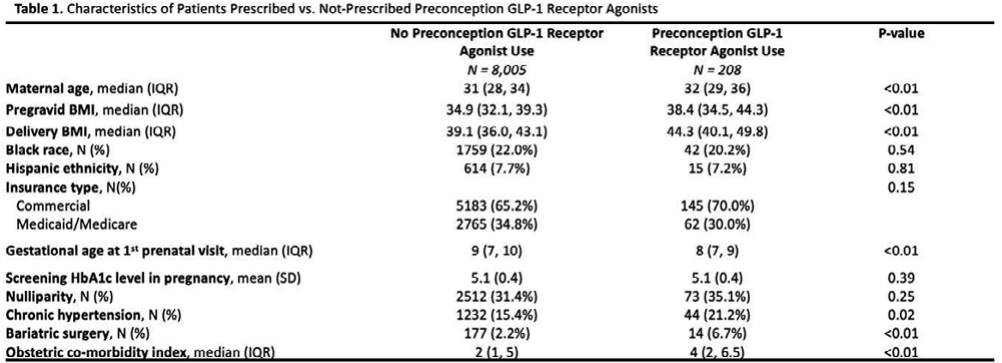

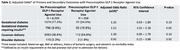

**3:30 PM – 5:00 PM**

## 1000 | **Evaluating a Universal Low Dose Aspirin Strategy After 1 Year in a Large Health System**

Adina R. Kern‐Goldberger^1^; Elizabeth Raiff^2^; Antonio Bajan^2^; Dana Baraki^2^; Monique Y. Katsuki^2^; Oluwatosin Goje^2^; Justin R. Lappen^2^

^1^Cleveland Clinic Lerner College of Medicine, Cleveland, OH; ^2^Cleveland Clinic Foundation, Cleveland, OH

**Objective**: Implementing a universal recommendation for low dose aspirin (LDA) is one strategy to optimize treatment uptake in populations with high rates of preeclampsia risk factors. This study evaluates the impact of a universal LDA (uLDA) policy on uptake in a high‐risk obstetric population and assesses characteristics of the residual population not prescribed aspirin to identify potential disparities and opportunities for improvement.

**Study Design**: This is a retrospective cohort study of all deliveries in a multihospital health system from 5/1/2023‐5/31/2025 (uLDA initiated 5/1/2024). First, LDA prescription rates at 16 weeks were extracted from the EHR for a sub‐group of “high‐risk” patients defined according to the ACOG/USPSTF criteria for LDA and evaluated by month and by race. Poisson regression evaluated the incidence rate ratio (IRR) of LDA prescription before and after the universal policy. LDA prescription rates were then evaluated for the entire patent population after initiation of uLDA and characteristics of patients prescribed vs. not prescribed LDA were compared in bivariable analyses.

**Results**: 24,187 patients were included (4,751 “high‐risk,” 19.6%) and LDA prescriptions were consistently higher in Black vs. non‐Black patients in the high‐risk group, with plateau for both groups beginning 1/2025 at approximately 80% (Figure 1). IRR for LDA after the intervention was 1.27 (95% CI 1.16‐1.40; *p* < 0.01) for Black and 1.76 (95% CI 1.53‐2.04; *p* < 0.01) for non‐Black patients. There were 11,524 patients in the post‐intervention group with characteristics in Table 1. Patients not prescribed LDA were more likely to be non‐English speaking and to have Medicaid insurance (*p* < 0.01 for both) and to have substance use disorder.

**Conclusion**: At 1‐year following a QI initiative to increase LDA prescription rates in a large health system, these data demonstrate equitable implementation by race but residual challenges for patients with other social determinants of health including language barriers. Further QI work will attempt to refine this intervention to optimize equitable implementation.

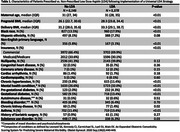

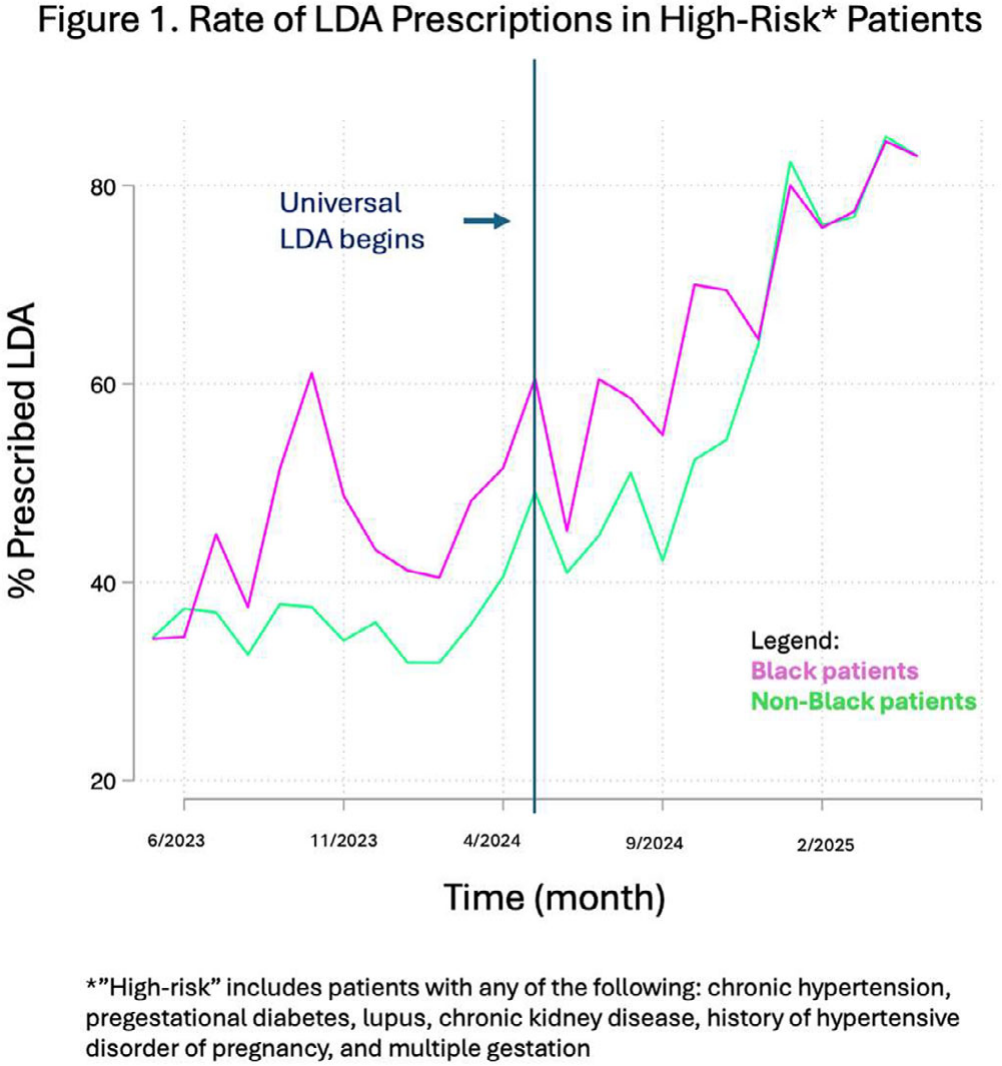

**3:30 PM – 5:00 PM**

## 1001 | **Clinical Outcomes Following Unintended Hysterotomy Extension During Second‐Stage Labor Cesarean**

Adwoa A. Baffoe‐Bonnie^1^; Leticia S. Flores^1^; Claire Wang^1^; Hannah Kelly^1^; Lillian Boettcher^2^; Sarahn M. Wheeler^3^; Rachel L. Wood^4^

^1^Duke University School of Medicine, Durham, NC; ^2^Department of Obstetrics and Gynecology, Duke University School of Medicine, Duke University School of Medicine/Durham, NC; ^3^Division of Maternal Fetal Medicine, Department of Obstetrics and Gynecology, Duke University, Duke University/Durham, NC; ^4^Division of Maternal Fetal Medicine, Department of Obstetrics and Gynecology, Duke University, Durham, NC

**Objective**: Unintended hysterotomy extensions are common in second‐stage cesarean delivery and may cause tissue trauma with implications for future pregnancies, including risk of spontaneous preterm birth (sPTB). We evaluated subsequent pregnancy outcomes after unintended hysterotomy extension during second‐stage cesarean delivery.

**Study Design**: Retrospective cohort study of patients who underwent second‐stage cesarean between March 2014–August 2024 and had a subsequent delivery ≥14 weeks at an academic tertiary care center. Unintended extension was defined as a documented extension beyond the initial hysterotomy; planned T or J incisions were excluded. The primary outcome was the occurrence of sPTB in the subsequent pregnancy. Secondary outcomes included delivery outcomes of the index and subsequent pregnancies. Multivariate logistic regression was performed on subsequent pregnancy outcomes to model the effect of hysterotomy extension.

**Results**: A total of 268 patients met inclusion criteria, with 70 (25.4%) having a documented unintended hysterotomy extension during second‐stage cesarean delivery. Of those with extensions, 10/70 (14.3%) involved the cervix. In unadjusted analysis, rates of subsequent sPTB were similar in the unintended extension group compared to the no‐extension group (11.4% vs. 5.6%, *p* = 0.100). However, in a multivariate logistic regression controlling for confounders, patients with an extension in their index cesarean delivery had significantly higher odds of sPTB in their subsequent pregnancy (aOR 2.83, 95% CI 1.03–7.8). Compared to no extensions, patients with extensions had significantly greater operative estimated blood loss, longer operative times, and higher rates of postpartum hemorrhage, O'Leary stitch placement, interventional radiology embolization, blood transfusion, and maternal ICU admission (all *p* <  .05, Table 1).

**Conclusion**: Hysterotomy extensions significantly increased maternal morbidity in the index pregnancy and increased the odds of sPTB in the subsequent pregnancy. Future studies should assess methods for primary prevention of hysterotomy extensions.

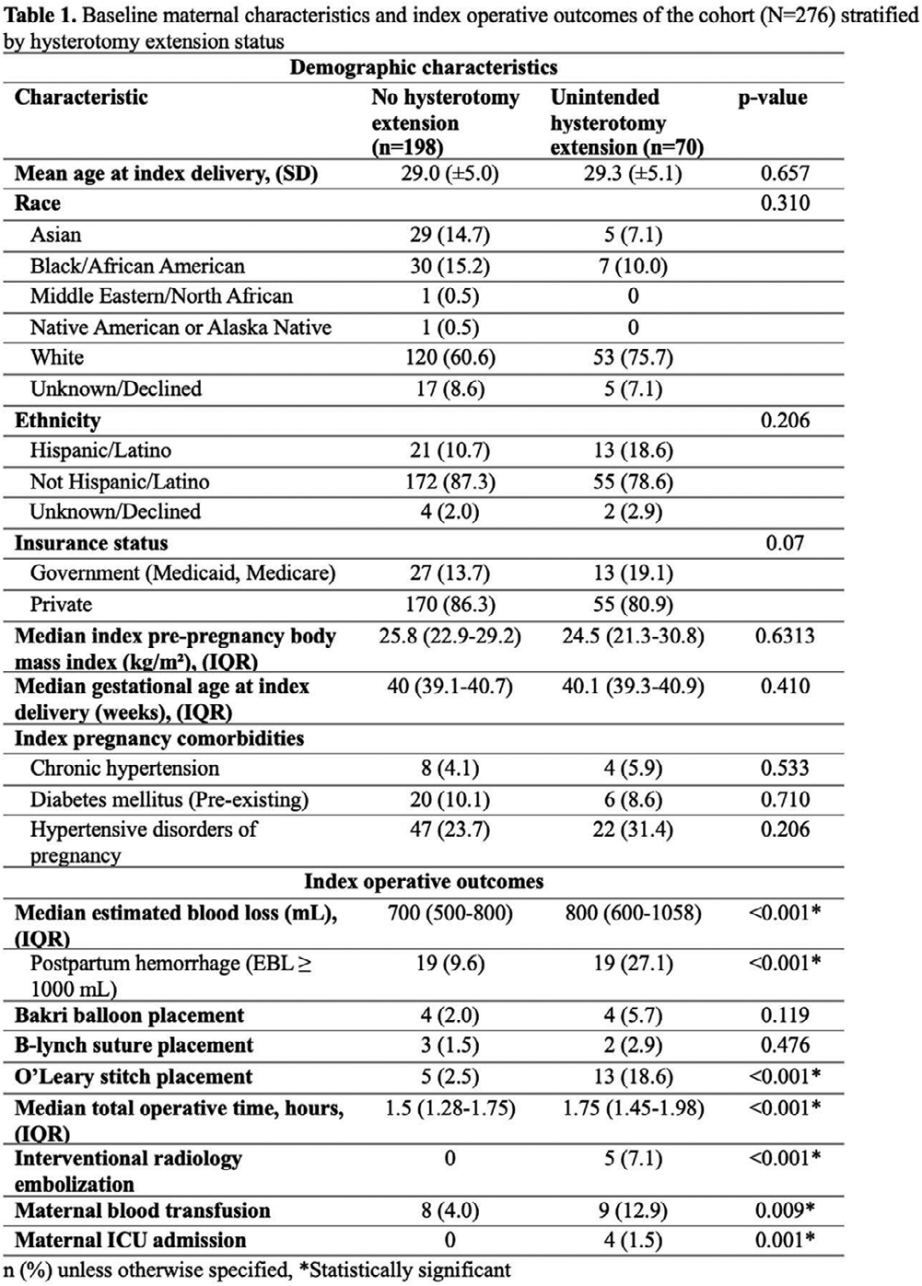

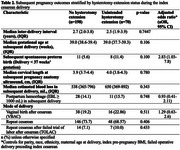

**3:30 PM – 5:00 PM**

## 1002 | **Knowledge Gaps in Cardiovascular Maternal Mortality: A Regional Survey of Frontline Providers**

Karen L. Florio, N/A^1^; Cassondra J. Kambeitz^2^; Akira Clemons^2^; Colin Paris^2^; Sharon T. Phelan^3^; Traci Johnson^4^; Michelle Y. Owens^5^; Niraj R. Chavan^6^; Veronica Gillespie‐Bell^7^; Sue Kendig, JD, WHNP‐BC^8^; Kensey Gosch^9^; Cornelia R. Graves^10^; John Spertus^4^

^1^University of Missouri, Lees Summit, MO; ^2^University of Missouri, Columbia, MO; ^3^Department of Obstetrics and Gynecology in UAB's Heersink School of Medicine, Helena, AL; ^4^University of Missouri‐Kansas City, Kansas City, MO; ^5^Mississippi State Department of Health, Jackson, MS; ^6^Saint Louis University, Saint Louis, MO; ^7^Louisiana Perinatal Quality Collaborative & Pregnancy Associated Mortality Review, New Orleans, LA; ^8^SSM Health, Saint Louis, MO; ^9^Saint Luke's Hospital of Kansas City, Kansas City, MO; ^10^Tennessee Maternal Fetal Medicine, Nashville, TN

**Objective**: To evaluate knowledge, attitudes, and clinical practices of non‐OB providers across ACOG District VII regarding cardiovascular‐related (CV) maternal morbidity and mortality, with a focus on awareness, use of CV screening tools, and familiarity with national maternal safety initiatives (AIM).

**Study Design**: A cross‐sectional electronic survey was administered to 1775 healthcare providers across eight states (AL, AR, KS, LA, MS, MO, OK, and TN). Of these, 610 providers met inclusion criteria and completed the survey. Respondents included physicians (65.9%), nurse practitioners (33.0%), and trainees (1.1%) specializing in family medicine, internal medicine, emergency medicine, and cardiology. The mean respondent age was 45.5 years, with an average of 13.9 years in clinical practice. The survey evaluated familiarity with the CDC definition of maternal mortality, use of validated CVD screening tools, awareness of AIM maternal safety bundles, and counseling practices related to CVD risk.

**Results**: Only 39.3% of respondents correctly identified maternal mortality as death within 365 days postpartum. Less than half (43.9%) reported using a CVD screening tool during pregnancy, and only 20.8% were aware of the AIM safety bundles. A minority (23.3%) recognized postpartum as the time of greatest risk for maternal mortality. While most respondents correctly identified Black, uninsured, and low‐income individuals as disproportionately affected, few were aware that American Indian/Alaska Native  have the highest and most rapidly rising mortality risk. Just over half (54.9%) acknowledged systemic discrimination as a contributing factor to maternal mortality, and 46.7% had ever completed formal education on the topic.

**Conclusion**: Providers caring for pregnant and postpartum patients have a significant gap in knowledge regarding maternal mortality and limited awareness of CV risk assessment tools or ongoing national safety initiatives. There is an urgent need for targeted educational interventions and systems‐level improvements for providers to improve their knowledge, and hopefully their care, for pregnant women.

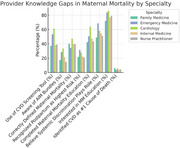

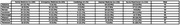

**3:30 PM – 5:00 PM**

## 1003 | **Transfusion Requirements by Acuity for Prenatally Suspected Placenta Accreta Spectrum**

Alesha M. White^1^; Jessica E. Pruszynski^2^; Anne M. Ambia^2^; Catherine Y. Spong^2^; Christina L. Herrera^2^

^1^University of Texas Southwestern Medical Center, Grand Prairie, TX; ^2^University of Texas Southwestern Medical Center, Dallas, TX

**Objective**: Morbidity associated with placenta accreta spectrum (PAS) is reduced with prenatal diagnosis and multidisciplinary management. Transfusion is a known complication of PAS, but there is limited data on transfusion requirements based on acuity of delivery. The objective of this study is to compare transfusion needs based on acuity of delivery.

**Study Design**: This is a retrospective cohort study that included all patients with prenatally suspected PAS at a single institution with a multidisciplinary team between 2019 and 2025. Maternal demographics and transfusion characteristics were compared between patients with prenatally suspected PAS who underwent scheduled delivery to those who developed another earlier indication for delivery. A multidisciplinary PAS team was involved in the coordination of delivery, regardless of acuity. Patients diagnosed with PAS intraoperatively without prior concern were excluded.

**Results**: Of 169 cases of prenatally suspected PAS, 114 (67%) were scheduled. As anticipated, patients with acute indication delivered at earlier gestational ages (34 (30.5–35.5) vs 36 (36–36), *p* < 0.001) and were more likely to have a diagnosis of gestational hypertension (*p* = 0.025) or pre‐eclampsia with severe features (*p* < 0.001). Twenty‐four (44%) of these patients were delivered for vaginal bleeding. Degree of PAS based on pathology, hysterectomy rates, and estimated blood loss were not different between groups (Table 1). Although rate of transfusion and activation of massive transfusion protocol did not differ, deliveries with acute indication were more likely to receive a transfusion of platelets (16% vs 3%, *p* = 0.002). Total number of units (4 (1–55) vs 5 (1–33), *p* = 0.499) and timing of transfusion did not significantly differ (Table 2).

**Conclusion**: Early indicated delivery accounted for a third of prenatally suspected PAS cases. While receiving platelets at a higher rate to compensate for hemorrhage and lost clotting factors, overall transfusion characteristics based on acuity of delivery do not significantly differ.

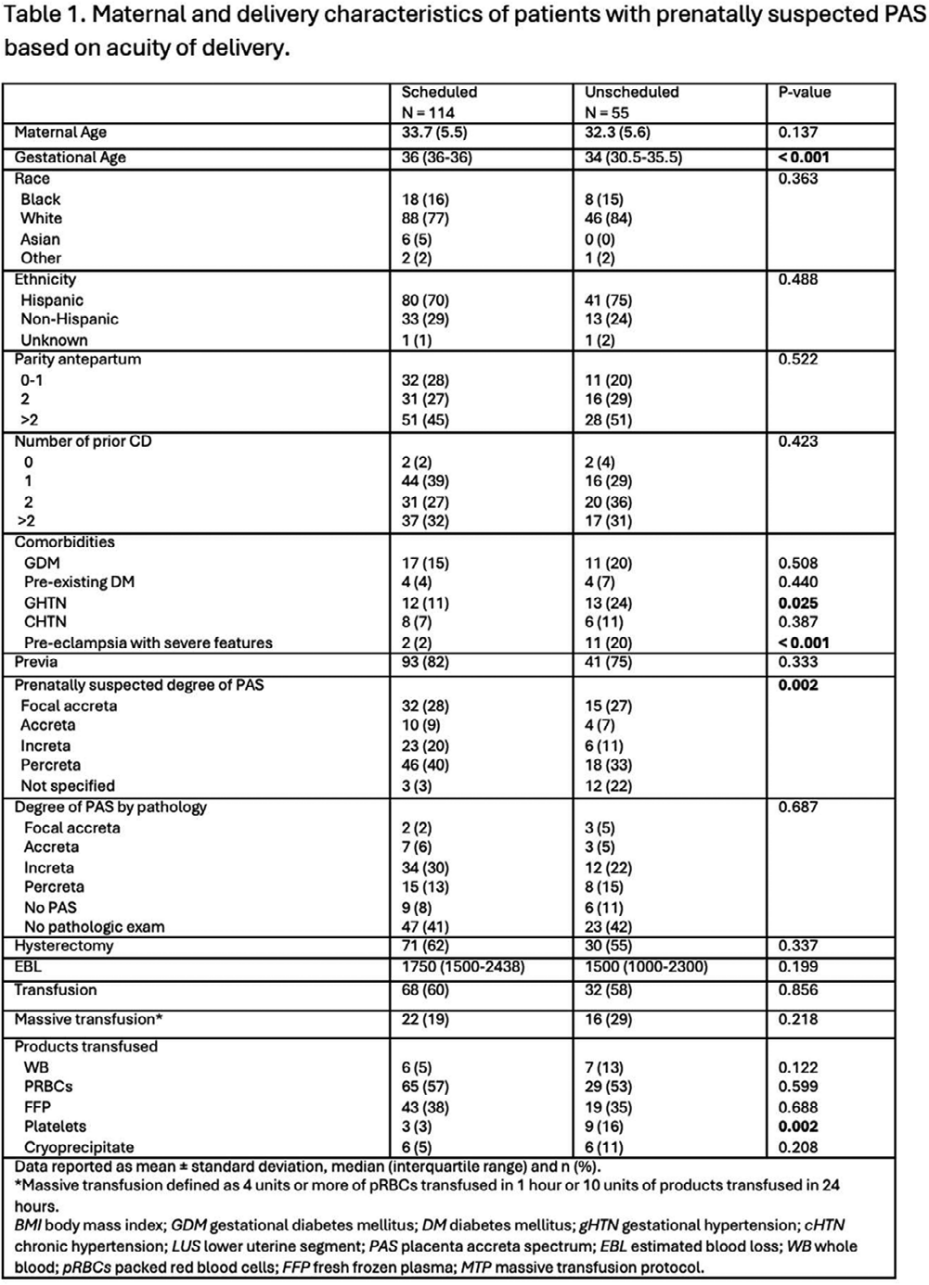

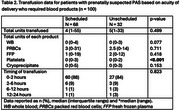

**3:30 PM – 5:00 PM**

## 1004 | **Maternal Morbidity in Cases of Cesarean Scar Ectopic Pregnancy Managed Expectantly**

Alesha M. White^1^; Catherine Y. Spong^2^; Elaine L. Duryea^2^; Christina L. Herrera^2^

^1^University of Texas Southwestern Medical Center, Grand Prairie, TX; ^2^University of Texas Southwestern Medical Center, Dallas, TX

**Objective**: The objective was to report maternal outcomes in cases of cesarean scar ectopic pregnancies (CSEPs) with cardiac motion (CM) managed expectantly.

**Study Design**: Retrospective study of patients with a sonographic concern for cesarean scar implantation with CM and who were expectantly managed and delivered at a single institution between 2012 and 2025. Maternal outcomes for these patients are reported with specific emphasis on outcomes for patients who progressed to the third trimester. SMFM criteria for CSEP were applied.

**Results**: During the study period 23 patients were diagnosed with a CSEP with CM, approximate incidence 1.1 per 10,000 pregnancies. Patients were diagnosed at a median gestational age of 6 weeks (6, 7). Three (13%) patients experienced a miscarriage in the first trimester, two of whom required hysterectomy due to persistent trophoblastic tissue. Four (17%) patients delivered in the second trimester, two required transfusion and all required hysterectomy. The remaining 16 (67%) delivered a livebirth in the third trimester (Figure). The median gestational age at time of delivery was 35 weeks (34, 36). All 16 required blood transfusion, and 13 (81%) required hysterectomy. Median EBL was 5000cc (2000, 6250) and median products transfused was 13 units (4, 31). Seven (41%) patients were transferred to the surgical intensive care unit after delivery and 7 (44%) experienced additional surgical complications. Fourteen patients had pathology available and of these 13 (93%) had pathology positive for PAS. All 13 of these patients met at least 5 of the 6 SMFM criteria for CSEP and had a pathology of 3A or higher (Table). The 3 (13%) patients who did not require hysterectomy each met fewer than 5 SMFM criteria for CSEP.

**Conclusion**: While 67% of patients with a CSEP and CM will go on to deliver a liveborn infant in the 3^rd^ trimester, they experience very high rates of maternal morbidity including hemorrhage and hysterectomy, with almost universal progression to at least Grade 3A pathologic PAS. This information is important to allow for informed decision making on how best to mitigate maternal risk.

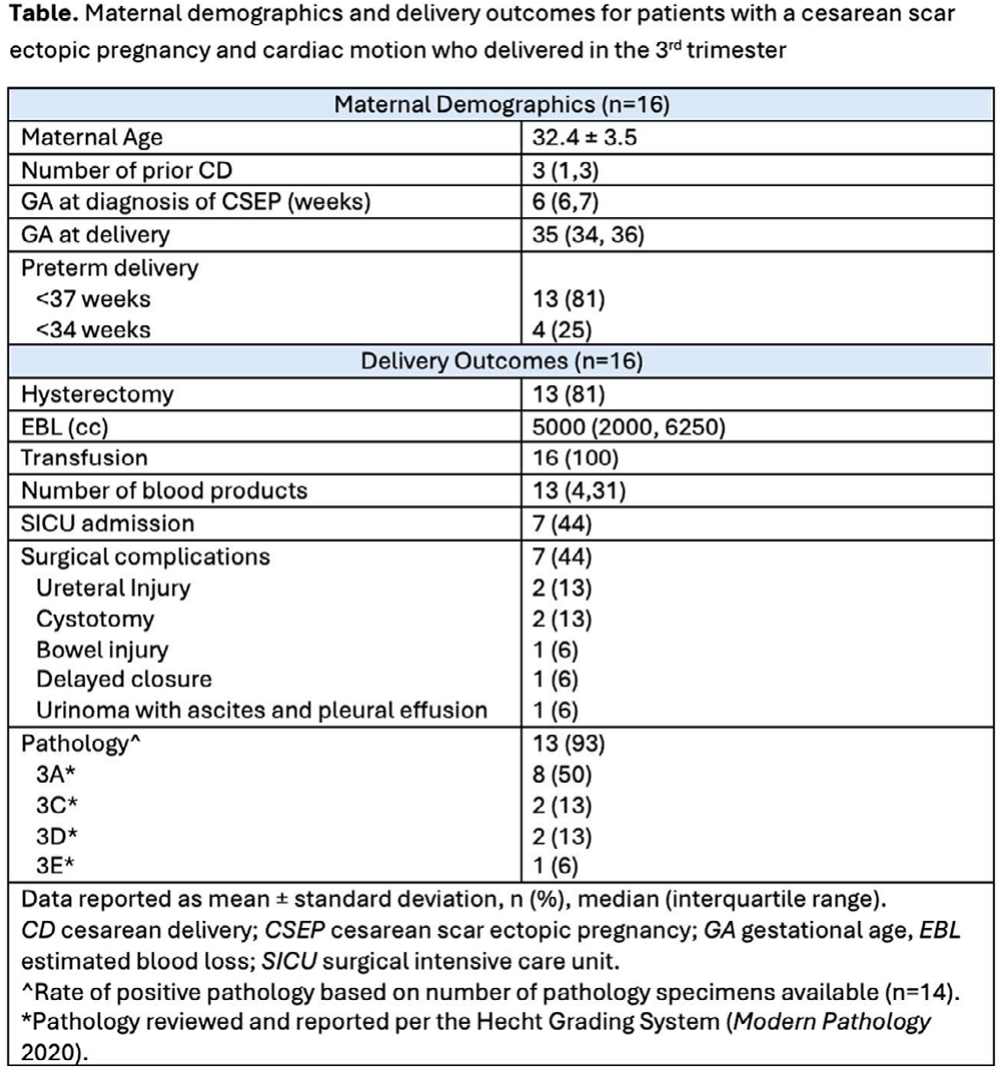

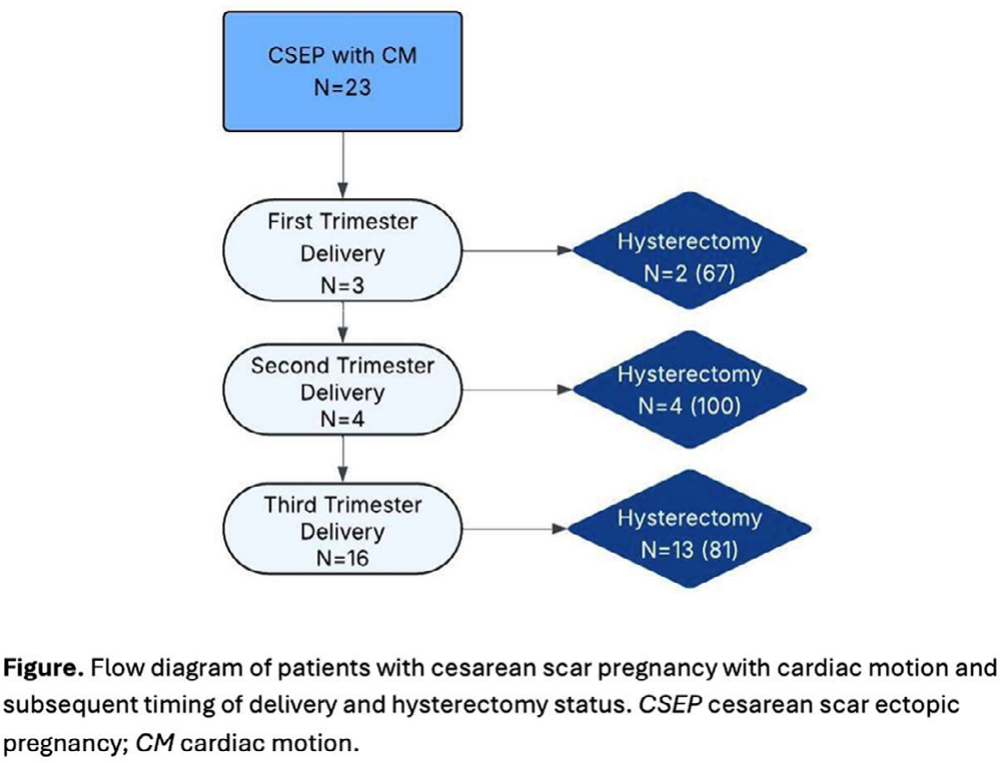

**3:30 PM – 5:00 PM**

## 1005 | **Evaluation of Protein Expression in Cases of Postpartum Hemorrhage of Varying Etiologies**

Alesha M. White^1^; Andrea Woods^2^; Andrew Lemoff^2^; Ashley Solmonson^2^; Sean Yates^2^; David B. Nelson^2^

^1^University of Texas Southwestern Medical Center, Grand Prairie, TX; ^2^University of Texas Southwestern Medical Center, Dallas, TX

**Objective**: The objective of this study was to evaluate varying etiologies of PPH using proteomic analysis. We hypothesized that proteins would differ among cases of PPH due to atony compared to other causes.

**Study Design**: This is a prospective observational study of patients who experienced PPH or who were at risk of experiencing PPH at the time of delivery. This data is a subgroup from a single‐center within a larger, multicenter industry sponsored study to validate a point of care (POC) viscoelastic assay device, the Quantra® sonorheometry (SEER) system. Samples were analyzed for proteomics by LC‐MS/MS following abundant plasma protein depletion, disulfide bond reduction and alkylation, trypsin digestion, TMTpro labelling, and high‐pH fractionation. The peptide data was analyzed with Proteome Discoverer 3.0 against the human reviewed protein database from UniProt. Proteins with an abundance in 1/3 or more of the samples were analyzed. Fold change was compared between patients with PPH secondary to atony, lysis of adhesions (LOA) and all other causes. Benjamini‐Hochberg corrected p‐values were reported with p‐value of < 0.05 considered to be significant.

**Results**: There were 41 total blood samples were collected of which 33 (80%) experienced PPH. Of patients with PPH, 13 (39%) were due to uterine atony, 8 (24%) from LOA, and 12 (36%) for all other causes. A total of 1129 proteins were identified in cases of PPH. 61 of these proteins were identified to be upregulated more than 2 fold in all cases of PPH (*p* < 0.001). 3 specific proteins, C‐reactive protein, serum amyloid A‐2 and serum amyloid A‐1 were noted to be upregulated 2 fold in cases of uterine atony and all other causes of PPH but downregulated 2 fold in cases of LOA (all *p* < 0.001) (Figures). All of these proteins are considered acute phase reactants produced in response to inflammation.

**Conclusion**: Utilizing proteomic analysis, we identified that protein expression varies by cause of PPH. Future analysis may determine pathway association which can be utilized for risk prediction.

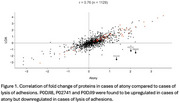

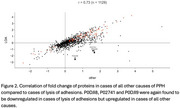

**3:30 PM – 5:00 PM**

## 1006 | **Is Forewarned Forearmed? Pregnancy Outcomes in Early‐Onset Fetal Growth Restriction**

Alex Dakin^1^; Ronan Daly^1^; Denisa Asandei^2^; Zara Molphy^3^; Patrick Dicker^4^; Michael Boyle^5^; Etaoin Kent^6^; Fergal D. Malone^7^

^1^Department of Obstetrics and Gynaecology, Royal College of Surgeons in Ireland, Dublin, Dublin; ^2^Department of Obstetrics and Gynaecology, Royal College of Surgeons in Ireland, Royal College of Surgeons in Ireland, Dublin; ^3^Royal College of Surgeons in Ireland, Dublin, Dublin; ^4^Department of Obstetrics and Gynaecology & Department of Public Health and Epidemiology, Royal College of Surgeons in Ireland, Royal College of Surgeons in Ireland, Dublin; ^5^Rotunda Hospital, Rotunda Hospital, Dublin; ^6^Rotunda Hospital, Dublin, Dublin; ^7^Department of Obstetrics and Gynaecology, Royal College of Surgeons in Ireland, Dublin, Ireland., Dublin, Dublin

**Objective**: Early‐onset fetal growth restriction (EOFGR) is associated with significant perinatal morbidity and mortality. Our aim was to determine perinatal and infant outcomes of EOFGR, and to evaluate for presence of antenatal predictors of these adverse outcomes.

**Study Design**: A retrospective cohort study was carried out from July 2016 to June 2022 of singleton pregnancies with uteroplacental EOFGR diagnosed prior to 32 weeks’ gestation, per Delphi consensus definition, at a tertiary institution with over 8000 deliveries per year. Genetic, structural or infectious causes were excluded, along with preterm pre‐labour rupture of membranes. Adverse outcome was defined as either perinatal mortality (intrauterine fetal demise or neonatal death) or composite neonatal morbidity (outlined in figure 1).

**Results**: 622 cases of EOFGR were diagnosed during the study period, of which 55% (341/622) were uteroplacental in origin. There was a 95% livebirth rate (325/341), and no terminations of pregnancy. There were 16 intrauterine fetal deaths (7.8%, 16/341) and 11 neonatal deaths (3.2%, 11/341), all occurring in cases diagnosed prior to 28 weeks’ gestation. Adverse outcomes of this cohort are described in figure 1. Table 1 correlates presence or absence of adverse outcome with maternal demographics, ultrasound characteristics and pregnancy outcomes. Unsurprisingly, earlier gestation and lower birthweight at delivery were associated with worse perinatal outcome, along with earlier gestation at diagnosis.

**Conclusion**: Reassuringly, there was a high livebirth rate in our cohort, however almost one in four pregnancies experienced an adverse outcome. Maternal demographics such as obesity, assisted reproductive technology, and chronic hypertension are not only risk factors for development of EOFGR, but also markers of its severity. Doppler abnormalities on the last ultrasound prior to delivery are more predictive of adverse outcome than amniotic fluid abnormalities, and development of co‐existent pre‐eclampsia confers higher risk. This information provides valuable insights for clinicians caring for these high‐risk pregnancies.

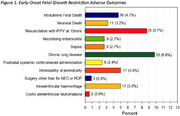

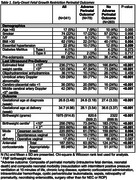

**3:30 PM – 5:00 PM**

## 1007 | **Provider Perspectives on Telemedicine in Tailored Prenatal Care: Barriers and Facilitators Post‐COVID‐19**

Sophia D. Fraga^1^; Tanvi Sharma^2^; Elizabeth Krans^3^; David M. Haas^4^; Alex Peahl^5^; Michelle Moniz^6^; Molly J. Stout^7^; Rachel A. Clark^8^; Kelsey Hoskin^2^; Julia Erickson^6^

^1^University of Michigan Medical School, University of Michigan, MI; ^2^University of Michigan Medical School, Ann Arbor, MI; ^3^UPMC Magee‐Womens Pregnancy and Women's Recovery Center, Pittsburgh, PA; ^4^Indiana University, Indiannapolis, IN; ^5^University of Michigan Department of Obstetrics & Gynecology, Ann Arbor, MI; ^6^University of Michigan, University of Michigan, MI; ^7^University of Michigan, Ann Arbor, MI; ^8^Trinity Health ‐ Ann Arbor Hospital, Ann Arbor, MI

**Objective**: Telemedicine is a key care delivery option in the new American College of Obstetricians and Gynecologists’ Clinical Consensus Document: Tailored Prenatal Care. Telemedicine care can improve access to prenatal care services; however, practices may require support to implement it. We describe providers’ perspectives of barriers and facilitators of adopting one aspect of tailored prenatal care: telemedicine.

**Study Design**: We conducted semi‐structured interviews with 30 clinically active (  >20% clinical FTE) maternity care professionals at three academic sites. Interviews were transcribed, imported into MaxQDA, and iteratively coded using the Consolidated Framework for Implementation Research (CFIR), which provides a structured approach to identifying barriers and facilitators of implementing evidence‐based interventions. Findings were organized by tailored care components and then CFIR domain to summarize barriers and facilitators of tailored prenatal care adoption.

**Results**: The most cited drivers of telemedicine implementation were in the outer setting, inner setting, and individual domains. Outer setting facilitators included COVID‐19 and social distancing requirements, which accelerated adoption of telehealth. Barriers included inequitable access to technology (e.g., broadband internet, home devices). Inner setting barriers were logistical: lack of private space for virtual visits, difficulties coordinating care with interpreters or other support services, and workflow challenges with support staff. At the individual level, barriers to telemedicine included provider discomfort with telehealth platforms and inconsistent patient access to home monitoring tools (e.g. blood pressure cuffs).

**Conclusion**: While telemedicine is promising for overcoming barriers to care, providers and health systems face multilevel barriers to implementation. A combination of local strategies, including workflow optimization and support staff, and policy‐level strategies, including payment reform, are needed to support access to virtual care for all patients.

**3:30 PM – 5:00 PM**

## 1008 | **Does Buprenorphine Administered for OUD Improve Stimulant Abstinence in Those With Concomitant Stimulant Use Disorder**

Alexander M. Harrison, Sr.^1^; Cynthia Cockerham^2^; Victoria L. Stanton^2^; Gregory S. Hawk^2^; Barbara V. Parilla^3^

^1^University Of Kentucky, Lexington, KY; ^2^University of Kentucky, Lexington, KY; ^3^University of Kentucky, University of Kentucky, KY

**Objective**: To evaluate if buprenorphine for opioid use disorder (OUD) decreases stimulant use in pregnant persons with a concomitant Stimulant Use Disorder (StUD).

**Study Design**: Prospective cohort study of pregnant patients receiving prenatal care through a comprehensive perinatal substance use treatment program between 2014 and 2025. All patients with OUD were offered MOUD primarily with buprenorphine. The study group was comprised of patients with both OUD and StUD who were prescribed buprenorphine, compared to control patients not receiving MOUD. The primary outcome was abstinence from stimulant use based on confirmatory urine drug screens (UDS) obtained during prenatal visits and at delivery. Fisher's exact test was used for categorical variables, and two sample t‐tests or Poisson regression were used to calculate p‐values as appropriate for quantitative variables.

**Results**: 260 patients participated in prenatal care while on buprenorphine for MOUD with a co‐occurring StUD, compared to 33 patients not receiving MOUD in the setting of OUD and StUD. There were no significant differences in demographics between groups with respect to age, race, insurance, tobacco use or psychiatric diagnosis, except that patients in the no MOUD group were more likely to have experienced PTSD, 41% v 16%, *p* = 0.0028. During prenatal care, 36% of UDS’ were positive for cocaine or methamphetamine for the no MOUD group compared to 18% for those on MOUD, *p* = 0.0042. Patients receiving MOUD attended more prenatal visits, 5 (3–11) v 9 (4–16), *p* = 0.0236. There was no significant difference in neonatal birthweight, 3045 g +/− 556 v 2944 g +/− 571, *p* = 0.4218, or treatment days for Neonatal Opioid Withdrawal Syndrome, 17 (13.8–25.5) v 14 (11.0–22.0), *p* = 0.2039. UDS at delivery demonstrated cocaine positive rate of 15% v 5% for those not on MOUD, compared to those on MOUD, *p* = 0.039.

**Conclusion**: Buprenorphine is a safe and effective medication used to treat OUD during pregnancy. It may also provide some benefit for concomitant StUD during pregnancy by decreasing stimulant use and increasing engagement with prenatal care. Further studies are needed.

**3:30 PM – 5:00 PM**

## 1009 | **Outcomes of Non‐Placenta Accreta Spectrum Peripartum Hysterectomy Following Implementation of a Placenta Accreta Spectrum Program**

Alexandra D. Forrest; Amanda Finney; Laura Prichett; Debra Eluobaju; Kristin Martin; Arthur Jason Vaught

Johns Hopkins University, Baltimore, MD

**Objective**: Peripartum hysterectomy carries high risks of maternal morbidity and mortality. While hysterectomies for placenta accreta spectrum (PAS) are often planned and performed by multidisciplinary teams, those for non‐PAS indications typically occur emergently and are less likely to have multidisciplinary approaches. Our institution launched a dedicated PAS program in 2018. We hypothesized that program‐related advances would improve non‐PAS peripartum hysterectomy outcomes.

**Study Design**: We conducted a single‐center retrospective cohort study of all non‐PAS peripartum hysterectomies from January 2013 to December 2024. Cases were grouped as pre‐ (2013–2017) and post‐ (2018–2024) PAS program implementation. We compared demographic and clinical outcomes before and after implementation using chi‐square or Fisher's exact tests and t‐tests or Mann‐Whitney U tests, as appropriate.

**Results**: 79 non‐PAS peripartum hysterectomies were performed: 42 pre‐ and 37 post‐program. Baseline demographics were similar except patients in the post‐program group had higher parity (median 3 vs 2, *p* = 0.04), lower prevalence of hypertension (8% vs 26%, *p* = 0.04), and were more likely to have an MFM as primary surgeon (97% vs 33%, *p* < 0.01). Post‐program patients also required fewer pRBC units (3 vs 6, *p* < 0.01), fewer total transfused units (6 vs 12, *p* = 0.02), had decreased ICU admission (49% vs 71%, *p* = 0.04), and fewer intraoperative consults (41% vs 76%, *p* < 0.01). 2 maternal deaths and 3 cardiac arrests occurred pre‐program and none occurred post‐program. In a subgroup analysis limited to hysterectomies for postpartum hemorrhage, ICU admission remained significantly lower post‐program (38% vs 75%, *p* = 0.01), though transfusion metrics were similar.

**Conclusion**: Implementation of a PAS program was associated with improved outcomes for peripartum hysterectomy performed for non‐PAS indications. These findings suggest that PAS program development may reduce adverse outcomes, ICU admission, and resource utilization in other peripartum hysterectomies.

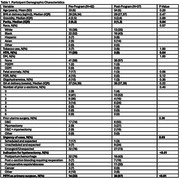

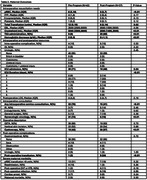

**3:30 PM – 5:00 PM**

## 1010 | **Perioperative Resource Utilization, Practice Patterns and Outcomes for High BMI Patients Undergoing Cesarean Delivery**

Alexandra C. Gallagher^1^; Elizabeth B. Sherwin^2^; Stephanie A. Leonard^3^; Dipro Chakraborty^1^; Natasha Harrison^1^; Selin Kandemir^4^; Pervez Sultan^5^; Ilona T. Goldfarb^2^

^1^Stanford University, Palo Alto, CA; ^2^Stanford University, Stanford, CA; ^3^Stanford University, Stanford University, CA; ^4^Vanderbilt University, Nashville, TN; ^5^Stanford University Healthcare, Palo Alto, CA

**Objective**: Despite evidence of increased perioperative risk for high BMI patients undergoing cesarean delivery, resource utilization rates are poorly characterized and tailored evidence‐based guidelines are lacking. This study aimed to describe perioperative resource utilization rates, practice patterns and outcomes in patients with high BMI and without to identify clinical gaps.

**Study Design**: We analyzed electronic health record data in a standardized common data model from a single, high volume academic institution between 2015 to 2023. We included patients with BMI 15–80 and stratified by delivery BMI <30, BMI 30–39.9 and BMI ≥40. Resource and practice pattern variables included obstetric anesthesia consultation, anesthesia type, 3 g cefazolin prophylactic antibiotic use, pharmacologic venous thromboembolism (VTE) prophylaxis, midline vertical skin incision, and Alexis and pannus retractor use. Maternal outcomes included incision to delivery interval, quantitative blood loss, hemorrhage (PPH), uterotonic medication use, transfusion, VTE, surgical site infection (SSI) and postpartum readmission. Neonatal outcomes included 5‐minute APGAR <7 and birthweight in grams.

**Results**: Among 8560 patients delivered by cesarean, 4507 (52.6%) had BMI <30, 3362 (39.3%) had BMI 30–39.9 and 691 (8.1%) had BMI ≥40. Potentially risk‐reducing resource utilization rates for patients with BMI ≥40 were low; 400 (58%) patients underwent obstetric anesthesia consultation, 260 (38%) received 3 g cefazolin, 53 (7.7%) utilized a pannus retractor, 135 (20%) utilized an Alexis retractor and 218 (32%) received pharmacologic VTE prophylaxis. Incision to delivery interval, conversion to general anesthesia, midline skin incision, PPH, SSI, VTE and postpartum readmission were observed at higher rates in the BMI ≥40 cohort, as was 5‐minute APGAR <7.

**Conclusion**: Despite higher morbidity for high BMI patients undergoing cesarean, there is wide variability in practice and underutilization of potentially risk‐reducing resources. Future research across institutions should investigate barriers and solutions.

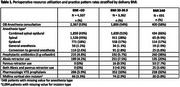

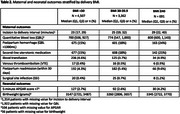

**3:30 PM – 5:00 PM**

## 1011 | **Latency Antibiotics Following Previable Preterm Prelabor Rupture of Membranes Prolongs Pregnancy by Two Weeks**

Alexandra L. Hammerquist^1^; Alexander M. Saucedo^1^; Michael D. Jochum, Jr.^1^; Enrico R. Barrozo^2^

^1^Baylor College of Medicine, Houston, TX; ^2^Baylor College of Medicine and Texas Children's Hospital, Houston, TX

**Objective**: Following preterm prelabor rupture of membranes (PPROM), antibiotics have been shown to extend the time between ROM and delivery (latency) as well as improve neonatal outcomes. However, it is unclear whether a similar benefit is seen in pregnancies with previable PPROM. We aimed to ascertain if treatment with antibiotics following previable PPROM prolongs latency to delivery.

**Study Design**: Single‐center retrospective cohort of pregnancies diagnosed with previable PPROM between 14 0/7 and 21 6/7 weeks between 2012 and 2024. Those who underwent termination, had major fetal anomalies, had a contraindication to expectant management, or met criteria for delivery within 48 hours of ROM, were excluded. Administration of antibiotics at any time after ROM was the exposure of interest. The primary outcome was the latency period between ROM and delivery. Secondary maternal and neonatal outcomes were collected. A multivariable linear regression was performed, adjusting for gestational age at ROM and clustering of observations.

**Results**: Of the 95 patients that met inclusion criteria, 63 (66.3%) received antibiotics. The latency period to delivery after previable PPROM for patients who received antibiotics after ROM was 38.15 days. This was 14.7 days (SE = 0.98, *p* = 0.034) longer compared to a latency of 11.62 days for those who did not receive antibiotics. Patients who received antibiotics delivered more frequently ≥ 22 0/7 weeks (80.95% vs. 15.62%, *p* < 0.005; Figure 1), had lower rates of intrauterine fetal demise (IUFD) (20.63% vs. 65.62%, *p* <  0.005), and higher rates of neonatal survival to discharge (49.21% vs. 6.25%, *p* < 0.031) (Table 1).

**Conclusion**: Latency antibiotics after previable PPROM prolonged the time to delivery by an average of 14.7 days. Those who received antibiotics delivered more frequently ≥ 22 0/7 weeks, experienced higher neonatal survival to discharge, and had lower incidence of IUFD. Although randomized prospective studies are needed, these  findings highlight the potential benefit of latency antibiotics after previable PPROM and support their consideration in expectant management.

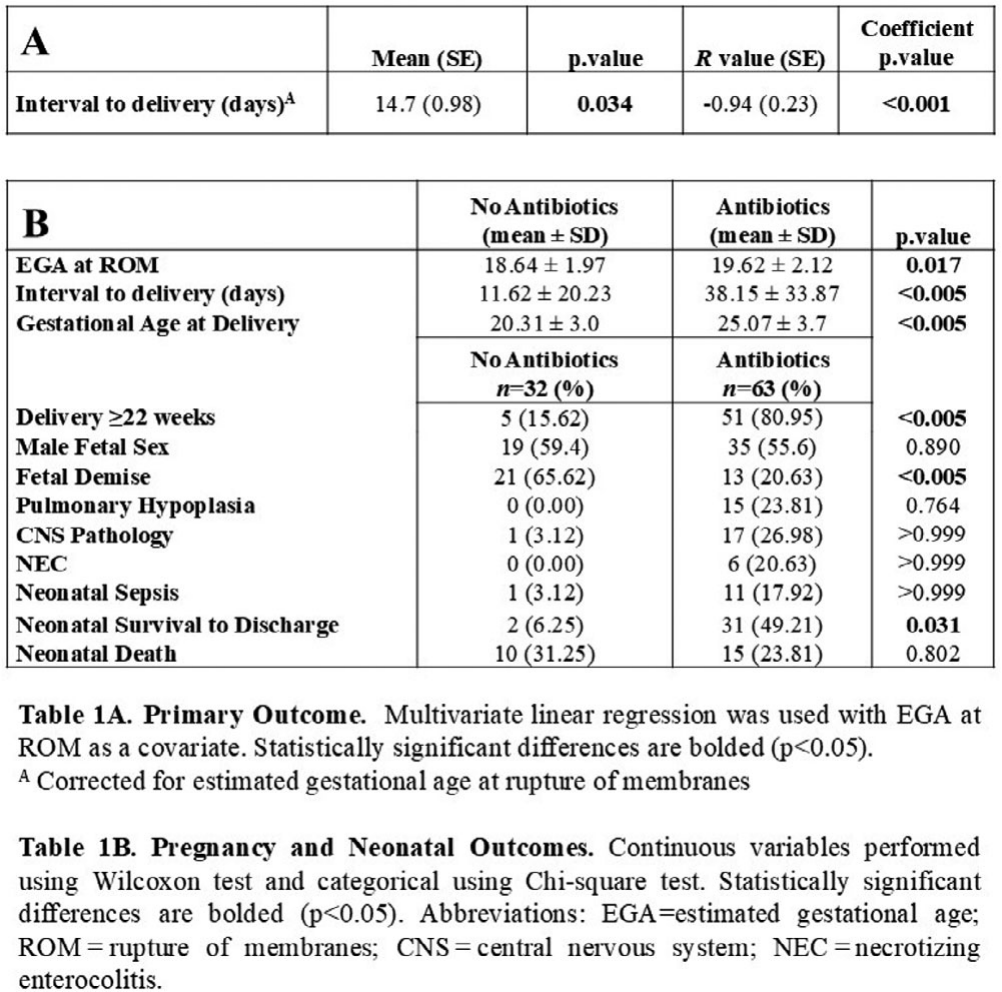

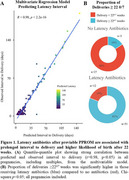

**3:30 PM – 5:00 PM**

## 1012 | **Pregnant Patients With Neurofibromatosis 2 and Associated Perinatal and Maternal Outcomes: A Systematic Review**

Alexandra Mahdasian‐Miller; Amelie Pham; Megan Nocita; Motolani Shonibare

Vanderbilt University Medical Center, Nashville, TN

**Objective**: To perform a systematic review of the existing literature on maternal neurofibromatosis 2 (NF2) in pregnancy and add a new case report to consolidate the best available evidence regarding associated adverse maternal and neonatal outcomes.

**Study Design**: A systematic review registered with PROSPERO in 7/2024 and followed PRISMA (Preferred Reporting Items for Systematic Reviews and Meta‐analyses) guidelines. A comprehensive search of PubMed, Embase, CINAHL, Scopus, Cochrane, and ClinicalTrials.gov was conducted through November 19, 2024. Search terms included various combinations of “neurofibromatosis 2” and “pregnancy”. Studies were excluded if no case of maternal NF2 in pregnancy was reported. Screening involved two independent reviewers, with conflicts resolved by a third reviewer. Study quality was assessed with the proposed tool set forth by Murad et. Al for case series and reports.

**Results**: Of the 2946 studies initially screened, 9 studies met inclusion criteria. These included 6 case studies, 2 prospective cohort studies and a retrospective cohort study with a total of 123 cases of NF2 in pregnancy, including a new case report from our institution (Table 1). Study bias‐risk assessment yielded a majority of low‐quality data. The most common maternal adverse outcome reported was advancing neurologic disease (8/75, 11%) and hypertensive disorder of pregnancy (<12/53, <23%). The need for neurological surgical intervention during pregnancy was rare (1/75, 1%). There was 1 case of reported iatrogenic preterm birth for fetal indications among <11/80 (<14%) cases of preterm birth (see Table 2). All patients with reported birth outcome (*N* = 80) had a live birth.

**Conclusion**: Maternal NF2 in pregnancy is rare with few cases described in the literature. Our study summarizes for the largest sample of reported NF2 cases in pregnancy, adding a new case to the existing literature, and underscores the complexity of this maternal condition. Overall birth outcomes were favorable. Further research is needed to better inform clinical management of NF2 in pregnancy and enhance patient counseling.

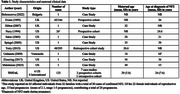

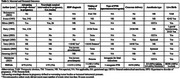

**3:30 PM – 5:00 PM**

## 1013 | **Episiotomy and Anal Sphincter Injury During Operative Vaginal Delivery in a United States Cohort**

Alexis A. Doyle; Amanda A. Allshouse; Torri D. Metz; Rosa J. Speranza

University of Utah, Salt Lake City, UT

**Objective**: To assess the association between episiotomy and obstetric anal sphincter injury (OASI) during operative vaginal delivery (OVD) in a diverse, modern U.S. cohort.

**Study Design**: Secondary analysis of patients who had an OVD of a term, cephalic singleton in the MFMU APEX cohort. The primary outcome was OASI (3rd or 4th degree laceration). The exposure was episiotomy. Multivariable logistic regression adjusted for length of 2nd stage, age, BMI, and birth weight. We tested for interactions between episiotomy and laceration by OVD type, history of vaginal delivery, and episiotomy type.

**Results**: Among 5277 participants, 3453 had a vacuum‐assisted vaginal delivery (VAVD) and 1824 had forceps (FAVD) (Table). An episiotomy was performed in 1704 (32%) of the deliveries (*n* = 1301 midline, *n* = 384 mediolateral, *n* = 19 unknown type). The OASI incidence was 20.1% (95% CI 19.0–21.2). Overall, the odds of OASI were higher among patients with an episiotomy (aOR: 1.45, 95%CI:1.25–1.67). There was a significant interaction between episiotomy and laceration by instrument type (*p* < 0.001), history of vaginal delivery (*p* = 0.02) and episiotomy type (*p* < 0.001) prompting a stratified analysis by these subgroups. In a multivariable model, compared with no episiotomy, midline episiotomy during a VAVD was associated with higher odds of OASI for patients both with or without a prior vaginal delivery. In contrast, mediolateral episiotomy was associated with lower odds of OASI for patients with no prior vaginal delivery for both VAVD and FAVD (Figure). No other subgroups significantly differed from no episiotomy in association with OASI.

**Conclusion**: Mediolateral episiotomy was associated with lower odds of OASI for patients undergoing OVD without a history of vaginal delivery. In contrast, episiotomy was not associated with a difference in OASI for patients with a prior vaginal delivery. These findings are consistent with several previous studies conducted in settings with different baseline rates of OVD and episiotomies and indicate there may be a subgroup of patients who could benefit from mediolateral episiotomy prior to OVD.

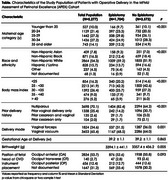

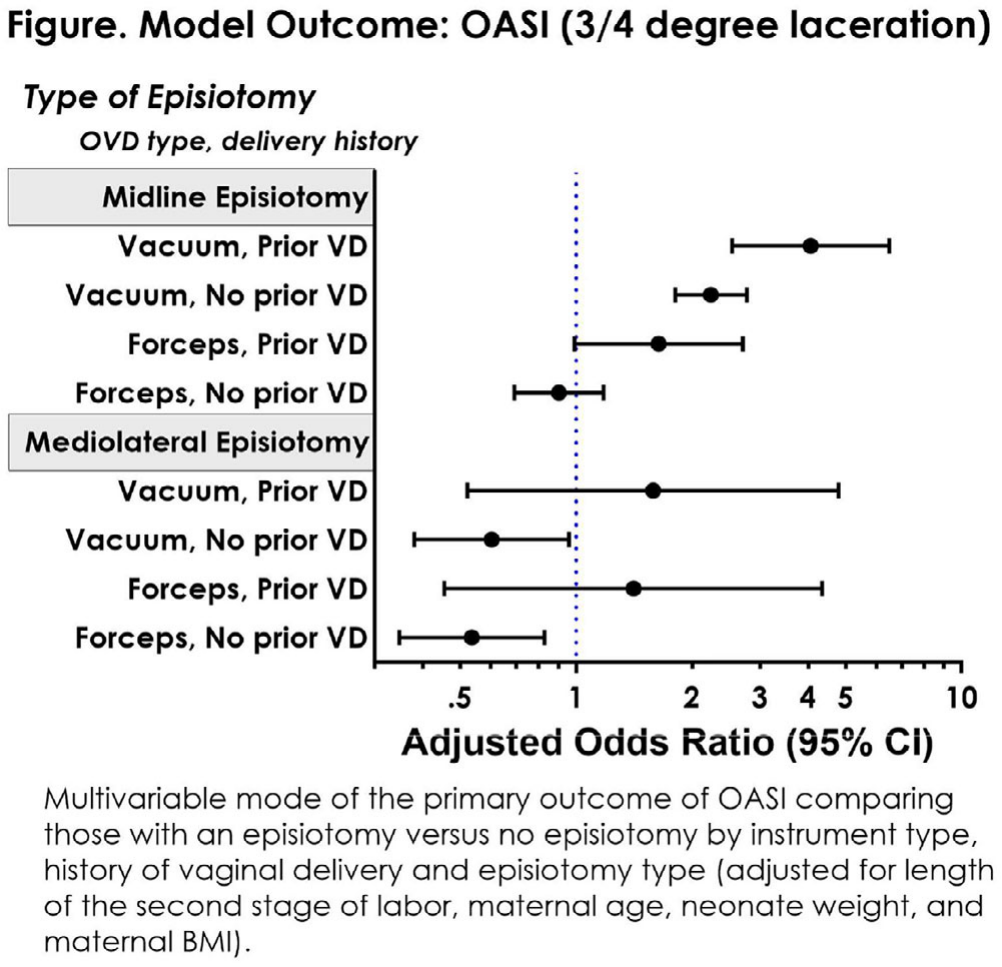

**3:30 PM – 5:00 PM**

## 1014 | **After Love Canal: Regional Perspective of Environmental Toxin Exposure and Development of Congenital Heart Disease**

Alexsaundra Zywicki^1^; Zimeng Gao^2^; Sarah Crimmins^3^

^1^University of Rochester Medical Center, Rochester, NY; ^2^University of Rochester Medical center, Rochester, NY; ^3^University of Rochester Medical Center–Roches, University of Rochester, NY

**Objective**: Investigate the impact of maternal residency in regions with high Hazard Ranking System (HRS) scores on development of congenital heart disease (CHD).

**Study Design**: Retrospective cohort study of neonates with CHD who delivered between 1/1/2016 and 1/1/2024 at Strong Memorial Hospital in Rochester, New York. Antepartum environmental toxin exposure was estimated utilizing the HRS score assigned to the residential zip code at time of delivery. High HRS regions were defined as those listed on the National Priorities List and low HRS regions were those that were not. Inclusion criteria included neonates with confirmed CHD by neonatal echocardiogram or autopsy. Exclusion included age <18 years, poorly control maternal diabetes (prepregnancy Hgb A1C ≥ 7%), family history of CHD, conception by IVF, or maternal teratogenic exposure. Chi‐square tests were performed to compare rates of critical CHD by HRS risk category.

**Results**: Of 786 the neonates born and diagnosed with CHD during the study dates, 462 met study inclusion, of which 27.5% (*n* = 127) resided in low HRS regions and 72.5% (*n* = 335) resided in high HRS regions. Demographic characteristics of the two cohorts were significantly different in maternal ethnicity, gestational age at delivery, and presence of extracardiac anomalies (Table 1). Adjusting against the background of all live births in each region, neonates with CHD and minimal antenatal risk factors had a higher risk of being born to an individual residing in a high risk HRS region (*p* = 0.03).

**Conclusion**: There were significantly higher rates of critical CHD with maternal residency in high HRS regions, as well as higher rates of concurrent neonatal extracardiac anomalies and delivery at earlier gestational ages. Non‐Hispanic Black and Hispanic patients reside in high HRS areas at disproportionate rates when compared to other demographic groups. Continued characterization of the potential reproductive and developmental consequences from maternal exposures to environmental toxicities at a regional level is necessary to optimize pregnancy outcomes for the maternal‐fetal dyad.

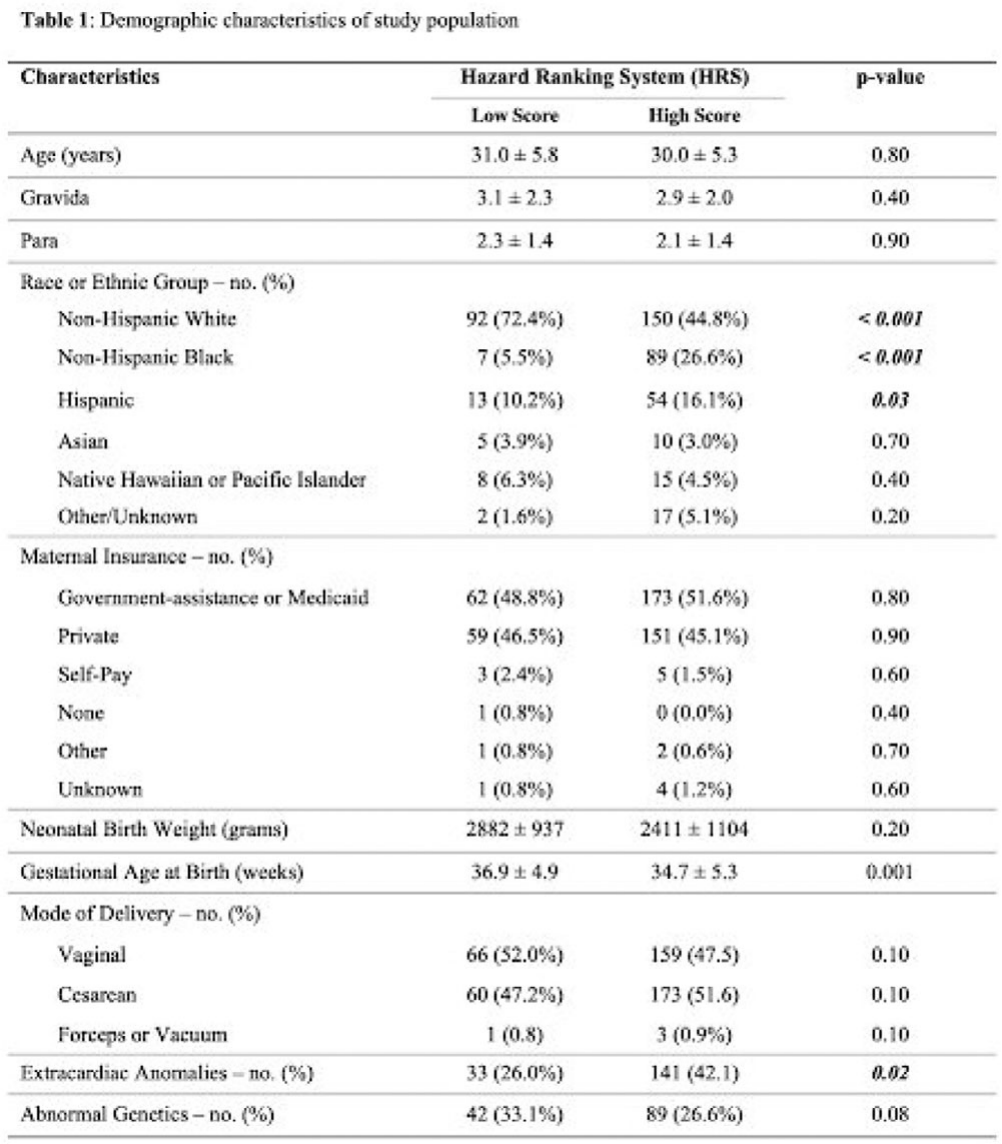

**3:30 PM – 5:00 PM**

## 1015 | **Maternal Immune Thrombocytopenia and Risk of Severe Hemorrhagic Morbidity at Delivery**

Alexzandra Adler^1^; Alexander Boscia^2^; Hannah E. Vincent^1^; Manesha Putra^1^

^1^University of Colorado, Aurora, CO; ^2^UPMC Magee‐Womens Hospital, Pittsburgh, PA

**Objective:  **To perform an updated analysis to evaluate the association between immune thrombocytopenia (ITP) and severe maternal morbidity (SMM) during admission for delivery.

**Study Design**: We conducted a population‐based cross‐sectional study using the most recent National Inpatient Sample (NIS) data from 2016–2022. We identified delivery admissions in patients aged 10–60 years, with and without a diagnosis of ITP (ICD‐10: D69.3). The primary outcome was composite SMM, as defined by the Centers for Disease Control and Prevention, among patients with and without ITP. Previously established comorbidity index (CI) was used to control for additional comorbidities. Logistic regression was used to evaluate the association between ITP and SMM, controlling for the number of CI.

**Results**: Our analysis identified 5,027,079 delivery admissions, of which 5699 individuals carried a diagnosis of ITP. SMM occurs in 8.7 percent of delivery admissions with ITP and 1.8 percent of delivery admissions without ITP. Patients with ITP were significantly more likely to experience SMM during the delivery admission (OR 2.5, CI 2.3–2.8, *p* < 0.001) after controlling for CI.

**Conclusion**: ITP is a rare disorder in pregnancy; however, it carries an approximately four‐fold increased risk of SMM during delivery admission. These findings underscore the importance of preconception counseling for patients with known ITP and the need for complex delivery planning for pregnant patients with ITP.

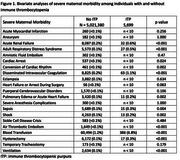

**3:30 PM – 5:00 PM**

## 1016 | **Histopathological Findings of In‐Utero Ventriculosubgaleal Shunt Placement in a Fetal Lamb Model of Induced Hydrocephalus**

Shohra Qaderi^1^; Weston Northam^2^; Soner Duru^3^; Jose L. Peiro^4^; Lucas Peiro^5^; Kjersti M. Aagaard^6^; Alireza A. Shamshirsaz^7^

^1^Baylor College of Medicine, Houston, TX; ^2^Boston Children's Hospital, boston, MA; ^3^Cincinnati, Cincinnati, OH; ^4^New York University Langone Health Fetal Research Lab, New York, NY; ^5^Cincinnati, Cincinnati, OH; ^6^HCA, Houston, TX; ^7^Boston Children's Hospital, Harvard Medical School, Boston, MA

**Objective**: To evaluate the histopathological changes which accompany in‐utero ventriculosubgaleal shunt (VSGS) placement in a fetal lamb model of experimentally induced hydrocephalus.

**Study Design**: Hydrocephalus was induced in fetal lambs via cisterna magna injection of BioGlueⓇ. At 13–15 days, VSGS were placed in utero. Near term, surviving fetal lambs were delivered, and brains were assessed by gross exam, postmortem MRI, and GFAP/Iba1 immunostaining. Shunt patency was tested by injecting saline into the distal end.

**Results**: Of *n* = 14 fetuses that underwent hydrocephalus induction, 7 survived to near term and underwent gross and histopathological evaluation. All exhibited ventriculomegaly, denuded ependyma, and disruption of the corpus callosum (Figure 1A). Shunt patency was confirmed in 4 of 7 lambs (Figure 1B) and in the remaining there was notableobstructed by tissue debris and fibrin. Western blot revealed consistent decreases in N‐cadherin and Beta‐4 tubulin expression, most pronounced in severe cases, correlating with ependymal denudation and loss of ciliated neuroepithelium. GFAP levels increased with hydrocephalus severity, indicating progressive astrocyte activation. In contrast, Iba1 expression remained unchanged or decreased, possibly reflecting limitations in detecting activated microglia. qPCR showed about 0.5‐fold reduction in GFAP.  IBA1 and N‐cadherin levels were similar across groups, while Beta‐4 tubulin decreased progressively with increasing severity.

**Conclusion**: In‐utero VSGS placement is technically feasible and maintains CSF flow in some cases. Despite this, hydrocephalus led to ventriculomegaly, ependymal denudation, and corpus callosum disruption. Severity correlated with loss of cilia‐related proteins and increased astrocyte activation, while microglial response appeared limited. Cerebral histopathology will be an important focus of study with subsequent fetal hydrocephalus treatments.

**3:30 PM – 5:00 PM**

## 1017 | **Low Fetal Fraction and Obstetric Outcomes: Insights From a Matched Cohort Study**

Alison Asirwatham; Sabrina Zhang; Lauryn Bates; Aspen Zheng; Katherine Leung; Heidi K. Leftwich

UMass Chan Medical School, Worcester, MA

**Objective**: Noninvasive prenatal screening analyzes cell‐free DNA (cfDNA) from maternal plasma to detect fetal aneuploidy. Adequate fetal cfDNA is required and low fetal fraction (LFF) can lead to assay failure. LFF is seen with maternal obesity and aneuploidy but may reflect placental dysfunction. While prior studies have associated LFF with hypertension and other adverse outcomes, few have focused specifically on assay failures. We compared obstetric outcomes and placental pathology in patients with normal vs LFF and explored whether prenatal aspirin use is related to placentally mediated adverse outcomes in cases of LFF.

**Study Design**: Retrospective 1:3 matched cohort study of pregnancies at our center (2018–2023) with cfDNA performed ≥ 10 weeks’ gestation and 1^st^ trimester ultrasound or reliable menstrual dating. Cases were defined as a “no call” cfDNA result due to low fetal fraction (<4%), matched to controls by age, parity, and BMI. Exclusions included fetal anomaly, aneuploidy, multifetal gestation, and prenatal anticoagulant use. We compared perinatal outcomes and placental pathology between groups and examined outcomes in cases between aspirin use.

**Results**: 118 cases and 354 controls included. Baseline characteristics were similar, though LFF had fewer male fetuses and were more likely to have a history of hypertension in a prior pregnancy. Those in the LFF group tended to deliver earlier (OR 0.89, *p* = 0.045) and had increased odds of preeclampsia without severe features (OR 2.47, *p* = 0.041). No other differences in outcomes or placental pathology were seen, though a trend towards increased odds of decidual vasculopathy in the LFF group (OR 2.87, *p* = 0.055) was noted. Aspirin use was not related to outcomes in the LFF group.

**Conclusion**: After matching, LFF was associated with earlier delivery and higher odds of preeclampsia, supporting its role as a potential marker of placental dysfunction. A trend towards increased decidual vasculopathy reinforces this association. Aspirin use was not associated with a difference in outcomes, highlighting the need for further study of targeted interventions.

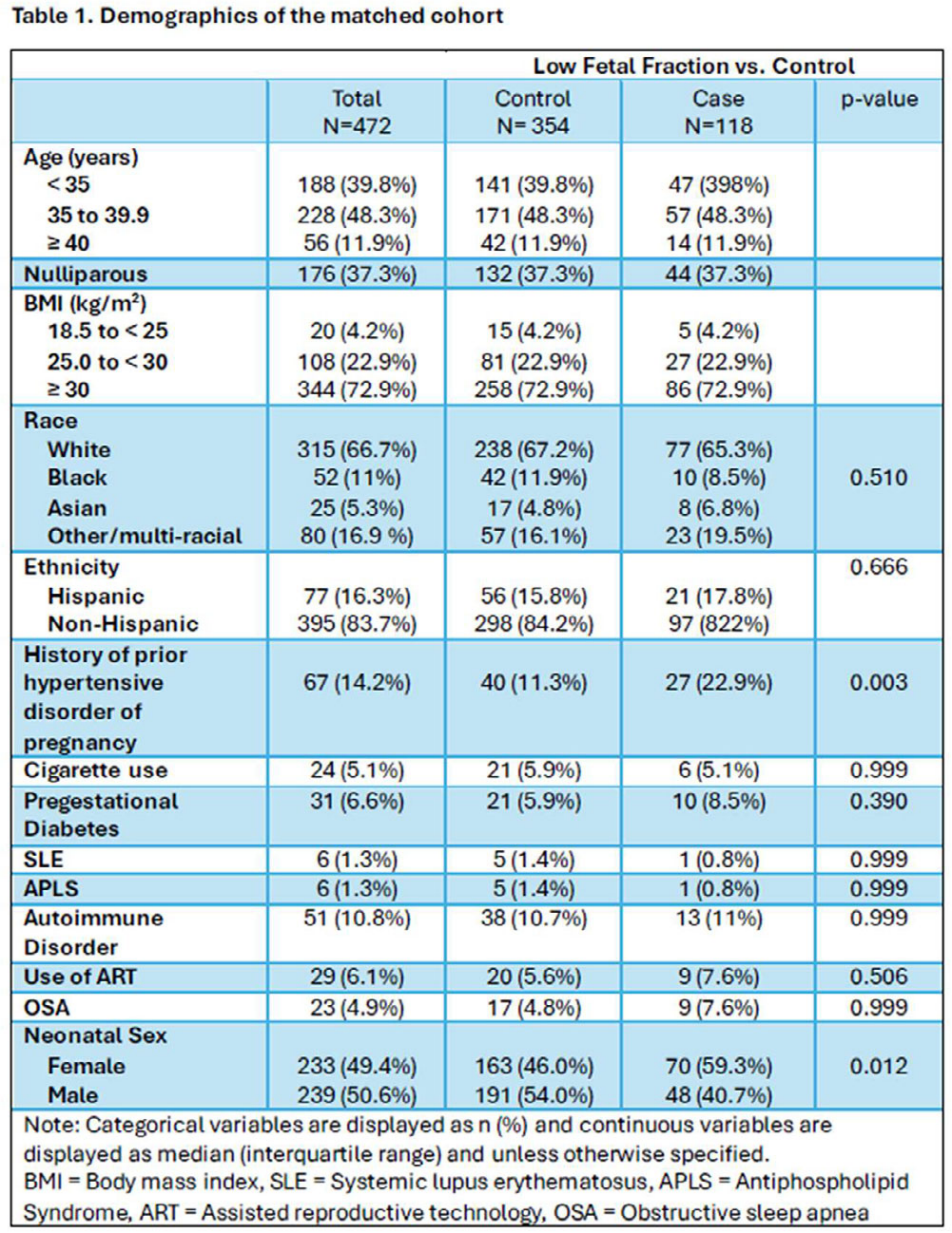

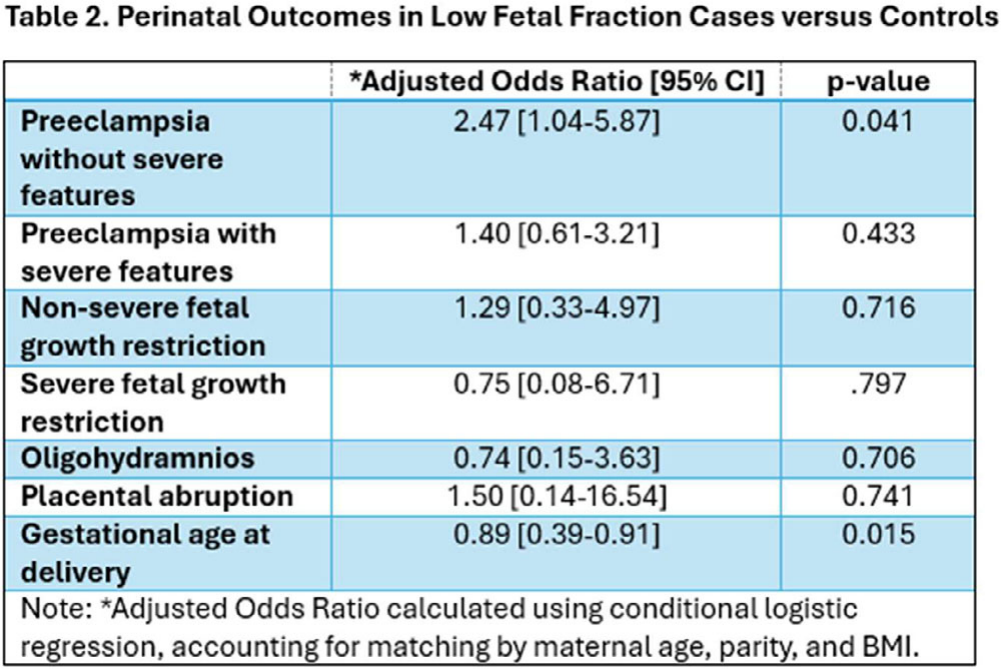

**3:30 PM – 5:00 PM**

## 1018 | **Impact of Altitude on Uterine Activity During Air Transport in Pregnancy**

Alixandria F. Pfeiffer^1^; Karissa Lonon^2^; Katie McBee^2^; Allison Moreno^3^; Hadley Ross^4^; Patrick S. Ramsey^5^; John J. Byrne^6^

^1^University of Texas Health San Antonio, University of Texas health San Antonio, TX; ^2^University of Texas Health San Antonio, San Antonio, TX; ^3^University Health System, San Antonio, TX; ^4^UT Health San Antonio, San Antonio, TX; ^5^Department of Obstetrics and Gynecology University of Texas Health Science Center in San Antonio, San Antonio, TX; ^6^University of Texas Health Science Center in San Antonio, San Antonio, TX

**Objective**: To evaluate the association between differing levels of altitude exposure and changes in uterine activity during air transport among pregnant patients with and without threatened preterm labor (PTL).

**Study Design**: This was a retrospective cohort study of pregnant patients ≥20 and <37 weeks’ gestation transported via air to a tertiary maternal care center between 2020–2025. Altitude exposure was categorized as High (  >3000 ft), Low (≤3000 ft), or Unknown. Referring hospital rurality was defined as Metro/Urban or Rural. The primary outcome was a significant increase in uterine activity during transport (≥1 additional contraction per 10 minutes). Secondary outcomes included preterm delivery and NICU admission. Multivariable logistic regression was used to assess the association between altitude exposure, PTL status, flight type (helicopter vs fixed‐wing), gestational age, insurance, and hospital rurality with all outcomes.

**Results**: Among 185 air‐transported patients, 113 were transferred for PTL. Threatened PTL was significantly associated with increased uterine activity (aOR 2.12, 95% CI 1.07–4.19, *p* = 0.03). Rural origin showed a non‐significant trend toward increased activity (aOR 1.75, 95% CI 0.53–5.85, *p* = 0.36). Altitude exposure was not significantly associated with contraction changes (Low vs High aOR 0.57, 95% CI 0.04–7.74, *p* = 0.93; Unknown vs High aOR 0.31, 95% CI 0.03–3.49, *p* = 0.34). Neither PTL status (aOR 1.38, *p* = 0.69) nor gestational age group (<34 weeks aOR 3.02, *p* = 0.17; ≥34 weeks aOR 0.33, *p* = 0.17) were associated with preterm delivery. For NICU admission, altitude was not predictive. However, rotor transport (aOR 11.80, 95% CI 1.48–93.79, *p* = 0.02) and lower gestational age (aOR 0.70 per week, 95% CI 0.53–0.94, *p* = 0.02) were independently associated with higher NICU risk.

**Conclusion**: Altitude exposure was not associated with increased uterine activity, preterm delivery, or NICU admission. Rotor transport and lower gestational age were the strongest predictors of NICU admission, supporting consideration of flight mode and timing in maternal transport triage.

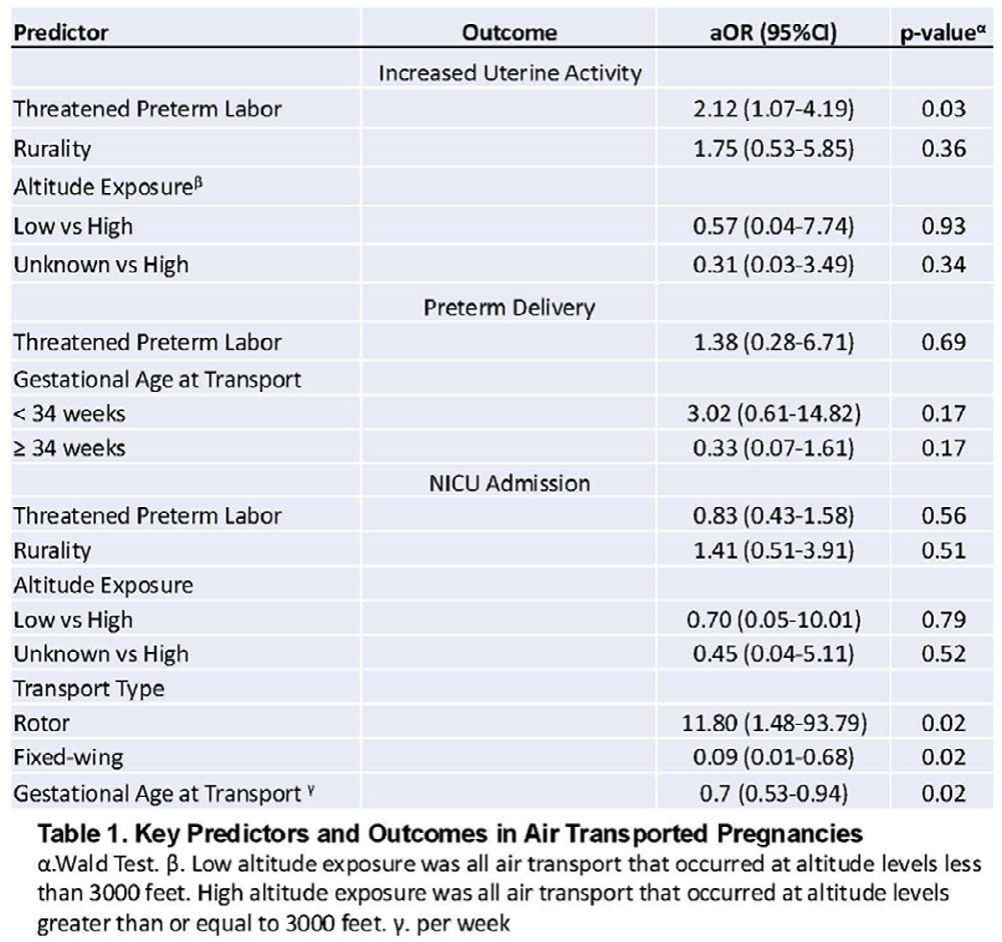

**3:30 PM – 5:00 PM**

## 1019 | **Posttraumatic Stress Disorder Two Months After Induced Vaginal Delivery: A Multicenter Prospective Study**

Alizee Froeliger^1^; Catherine Deneux‐Tharaux^2^; Lola Loussert^3^; Anne Laure SUTTER‐DALLAY^4^; Hugo Madar^5^; Loïc Sentilhes^5^

^1^Department of Obstetrics and Gynecology, Bordeaux University Hospital, Department of Obstetrics and Gynecology, Bordeaux University Hospital, France., Aquitaine; ^2^Université Paris Cité Institut Santé Des Femmes, Centre for Research in Epidemiology and Statistics (CRESS) U1153, Obstetrical Perinatal and Pediatric Epidemiology Research Team (Epopé), INSERM, INRAE, Paris, France., Paris, Ile‐de‐France; ^3^OPPaLE team, Centre for Research in Epidemiology and Statistics (CRESS) U1153, INSERM, Department of Obstetrics and Gynecology, Toulouse University Hospital, France., Languedoc‐Roussillon; ^4^Department of Perinatal Psychiatry, Charles Perrens Hospital, Bordeaux University Hospital, Department of Perinatal Psychiatry, Charles Perrens Hospital, Bordeaux University Hospital, France, Aquitaine; ^5^Bordeaux University Hospital, Bordeaux Universitary Hospital, Aquitaine

**Objective**: To assess PTSD prevalence and identify associated characteristics two months after induced vaginal delivery of singletons at or near term.

**Study Design**: Ancillary cohort study of the TRAAP randomized trial conducted in 15 French university hospitals (2015–2016). Women included had labor induction followed by singleton vaginal delivery after 35 gestational weeks. Labor and delivery characteristics were prospectively collected. PTSD profile at two months was assessed using two self‐administered questionnaires: IES‐R‐based profile (score >95th percentile) or TES‐based profile (presence of all three symptom criteria: B, C, and D). Prevalence was corrected for nonresponse using inverse probability weighting. Associations with potential risk factors were examined using multivariate logistic regression.

**Results**: Of 3891 women in the TRAAP trial, 794 (20.4%) had labor induced. Among them, 560 (70.5%) completed questionnaires and constituted the study population. The corrected PTSD profile prevalence at two months was 10.6% (95%CI, 8.1–13.7). An increased risk of PTSD profile was associated with women born in Africa (North Africa aOR 2.6; 95%CI, 1.1–6.8 and sub‐Saharan Africa aOR 3.3; 95%CI, 1.1–11.1 compared to women born in Europe),  with psychiatric history (aOR 3.0; 95%CI, 1.1–8.9), as well as obstetric factors such as cervical ripening (aOR 1.9; 95%CI, 1.1–3.6), fetal indication for induction (aOR 2.6; 95%CI, 1.2–5.4), and postpartum hemorrhage ≥1000 mL (aOR 4.9; 95%CI, 1.8–13.2). After adjustment, bad memories of delivery at day 2 postpartum also remained associated with increased PTSD risk (aOR 3.5; 95%CI, 1.5–10.2).

**Conclusion**: Two months after induced vaginal delivery at or near term, about 10% of women presented symptoms of PTSD. Apart from the psychosocial risk factors, subgroups at higher risk included women who had cervical ripening, induction of labor for fetal indication, postpartum hemorrhage or bad memories of delivery in the immediate postpartum.

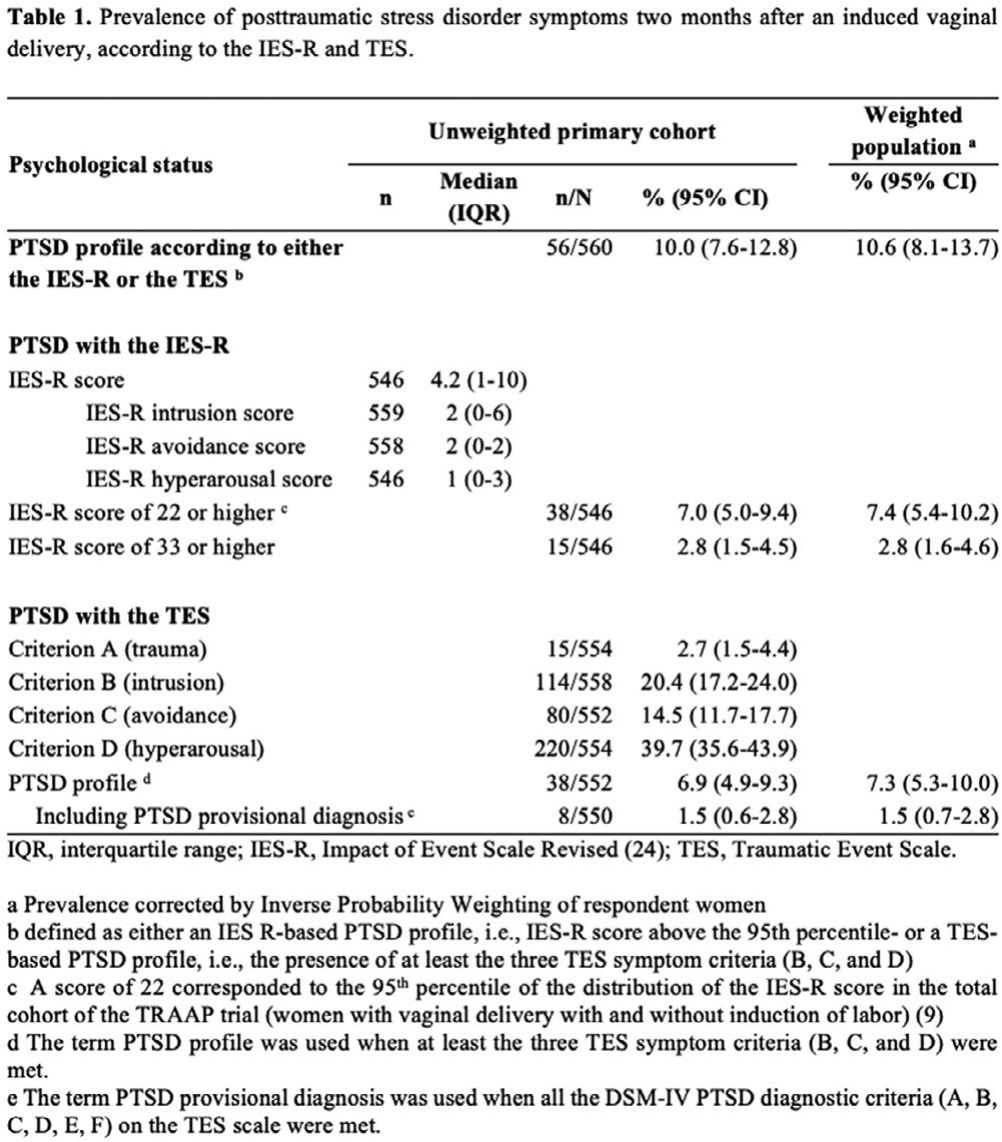

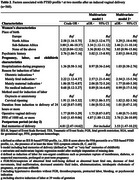

**3:30 PM – 5:00 PM**

## 1020 | **Outcomes of Macrodose Buprenorphine Inductions in Pregnancy**

Allison Kurzeja^1^; Abigail B. Clark^2^; Jessica Sisco^1^; Elaine L. Duryea^1^; Emily H. Adhikari^1^; Polly B. Cordova^3^; Jessica McNeil^3^; Amber Fisher^3^; Joshua Kern^1^; Nancy S. Onisko^1^; Aldo Andino^1^; Kurt Kleinschmidt^1^; Jessica E. Pruszynski^1^; C. Edward Wells^1^; Anne M. Ambia^1^

^1^University of Texas Southwestern Medical Center, Dallas, TX; ^2^University of Texas Southwestern Medical Center, University of Texas Southwestern Medical Center, TX; ^3^Parkland Health, Dallas, TX

**Objective**: To evaluate maternal and neonatal outcomes with a standardized, high‐dose buprenorphine induction protocol for antepartum patients with opioid use disorder (OUD).

**Study Design**: This was a retrospective cohort study of patients admitted with OUD who underwent an antepartum macrodose buprenorphine induction protocol. Records were reviewed for demographics, OUD treatment characteristics, maternal, obstetric, and neonatal outcomes. A descriptive and analytic statistical analysis was completed.

**Results**: Between September 2023 through May 2025, 72 buprenorphine inductions occurred at a median gestational age of 17 weeks. 4 (5.6%) patients underwent buprenorphine induction but were transitioned to methadone due to persistent symptoms.  The average induction dose of buprenorphine was 16mg, as per the institutional protocol (90.2%). 4 patients (5.6%) experienced precipitated withdrawal and 33 (45.8%) required additional “rescue” doses for ongoing symptoms. 47 (65.2%) remained on buprenorphine at most recent follow up. Compared to those who discontinued treatment, patients who continued had significantly fewer additional admissions for substance use disorder (0 [IQR 0–1] vs. 1 [IQR 0–2]; *p* < 0.001). Gestational age at delivery and birthweight were similar between patients who continued and discontinued their buprenorphine (38 weeks [IQR 37–39.5] vs. 38 weeks [IQR 36–40]; *p* = 0.396, 32998g [IQR 2702–3526] vs.3090g [IQR 2675–3290]; *p* = 0.218 respectively).  Of patients with delivery outcomes available, more patients continuing buprenorphine were discharged with their neonate (18 (75%) vs. 7 (39%)), though this difference was not statistically significant (*p* = 0.389).

**Conclusion**: A standardized macrodose buprenorphine induction protocol successfully initiated treatment in pregnant patients with OUD. Continued buprenorphine use was associated with significantly fewer readmissions for substance use disorder, supporting its effectiveness in this vulnerable population.

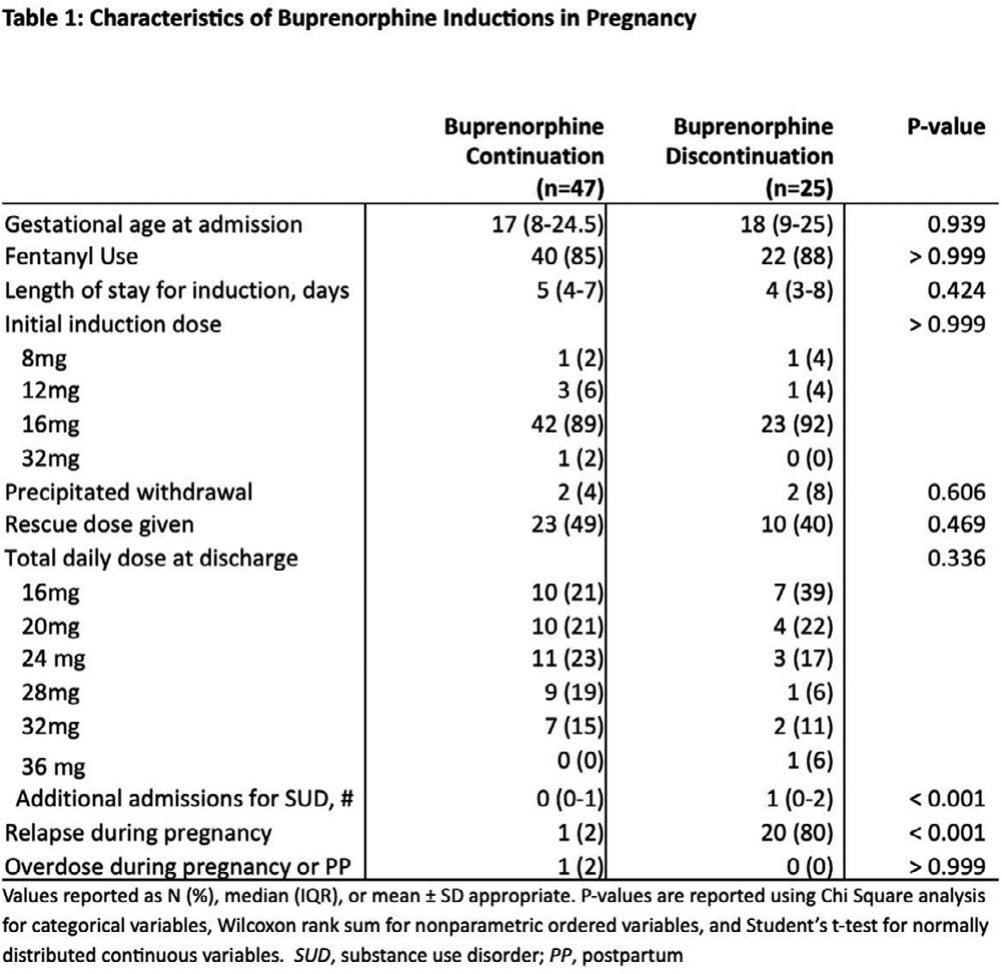

**3:30 PM – 5:00 PM**

## 1021 | **Outcomes of Elective Versus Medically‐Indicated Radiofrequency Ablation for Multifetal Reduction of Monochorionic Pregnancies**

Allison D. Perelman^1^; Sara E. Edwards^1^; Nicola F. Tavella^2^; Ariana Mills^3^; Jordan Davis^1^; Manasa G. Rao^4^; Kirema Garcia‐Reyes^3^; Angela T. Bianco^5^; Joanne L. Stone^5^; Chelsea A. DeBolt^1^

^1^Division of Maternal‐Fetal Medicine, Icahn School of Medicine at Mount Sinai, New York, NY; ^2^Division of Maternal‐Fetal Medicine, Icahn School of Medicine, Icahn School of Medicine at Mount Sinai Hospital, New York, NY; ^3^Division of Interventional Radiology, Icahn School of Medicine at Mount Sinai, New York, NY; ^4^Columbia University Irving Medical Center, New York, NY; ^5^Icahn School of Medicine at Mount Sinai, New York, NY

**Objective**: We have previously demonstrated improved pregnancy outcomes in monochorionic (MC) twins who undergo multifetal reduction (MPR) via radiofrequency ablation (RFA) for an elective indication when compared to medically‐indicated reduction, but this study was limited by small sample size. The current study aims to determine whether live birth rates differ for elective MPR (eMPR) compared to selective MPR (sMPR) via RFA in MC twins and higher order multiples in a large cohort.

**Study Design**: This was a retrospective cohort study of MC pregnancies undergoing MPR via RFA between 2008 and 2025 at a single institution. eMPR was compared to sMPR which was defined as MPR for a complication of monochorionicity or fetal anomaly. Chi‐squared and Fisher's exact tests compared proportional differences, and multivariable logistic regression models were used to estimate the likelihood of live birth.

**Results**: Of 115 patients included, there were 62 (54%) eMPRs and 53 (46%) sMPRs. 35 (66%) of the sMPRs were for MC complications and 18 (34%) for fetal anomalies. eMPRs occurred at an earlier gestational age (15 vs 18 weeks, *p* < 0.001). Many subjects were referred from outside institutions, and pregnancy outcomes were known for 50 (81%) of the elective group and 42 (81%) of the selective group. In an unadjusted analysis of those with known pregnancy outcomes, eMPR had a significantly higher live birth rate (92% vs 72%, *p* < 0.05) and lower rate of small for gestational age newborn (22% vs 5%, *p* < 0.05) (Table 2).  When adjusting for possible confounders, the likelihood of live birth remained significantly higher in the eMPR group (aOR 4.8, 95% CI 1.20–33.13).

**Conclusion**: In a retrospective cohort, elective reductions occurred at a significantly earlier gestational age. When adjusting for this, elective reductions remained significantly more likely to result in live birth when compared to selective reductions. Patients should be counseled that should they chose to pursue MPR at an earlier gestational age before complications of MC arise, they may have a higher chance of live birth.

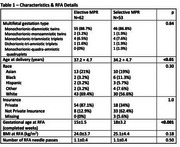

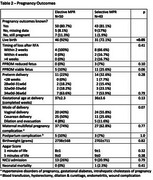

**3:30 PM – 5:00 PM**

## 1022 | **Outcomes of Non‐Medically Indicated Induction of Labor at 39 Weeks Among Births Complicated by Obesity**

Alyssa R. Hersh^1^; Bharti Garg^2^; Kristin C. Prewitt^1^; Aaron B. Caughey^2^

^1^Oregon Health & Science University, Portland, OR; ^2^Oregon Health & Science University, Oregon Health & Science University, OR

**Objective**: Given the recent increase in non‐medically indicated induction of labor (IOL) following publication of the ARRIVE trial, it is important to assess perinatal outcomes for maternal factors that influence labor physiology and outcomes, such as obesity. We sought to assess perinatal outcomes of births complicated by obesity managed with IOL compared with expectant management (EM).

**Study Design**: This was a retrospective cohort study of singleton, non‐anomalous nulliparous births between 39–42 weeks in California between 2018–2020. We included births complicated by a pre‐pregnancy body mass index of 30 kg/m2 or greater. Medical indications for IOL and planned cesarean deliveries were excluded. We examined maternal and neonatal outcomes using bivariate and multivariable analyses with a *p* value of 0.05.

**Results**: There were 23,945 births included in this analysis, of which 4346 (18%) were managed with IOL at 39 weeks. IOL was associated with lower risk for cesarean delivery (aIRR 0.94, 95% CI 0.89–0.98), large for gestational age (aIRR 0.87, 95% CI 0.79–0.95) and macrosomia (aIRR 0.62, 95% CI 0.57–0.69). There was a higher risk of operative vaginal delivery (aIRR 1.18, 95% CI 1.08–1.30).

**Conclusion**: Among nulliparous births complicated by obesity, non‐medically indicated IOL at 39 weeks is associated with lower cesarean deliveries, fewer neonates that are large for gestational age or macrosomic, and increased operative vaginal deliveries.

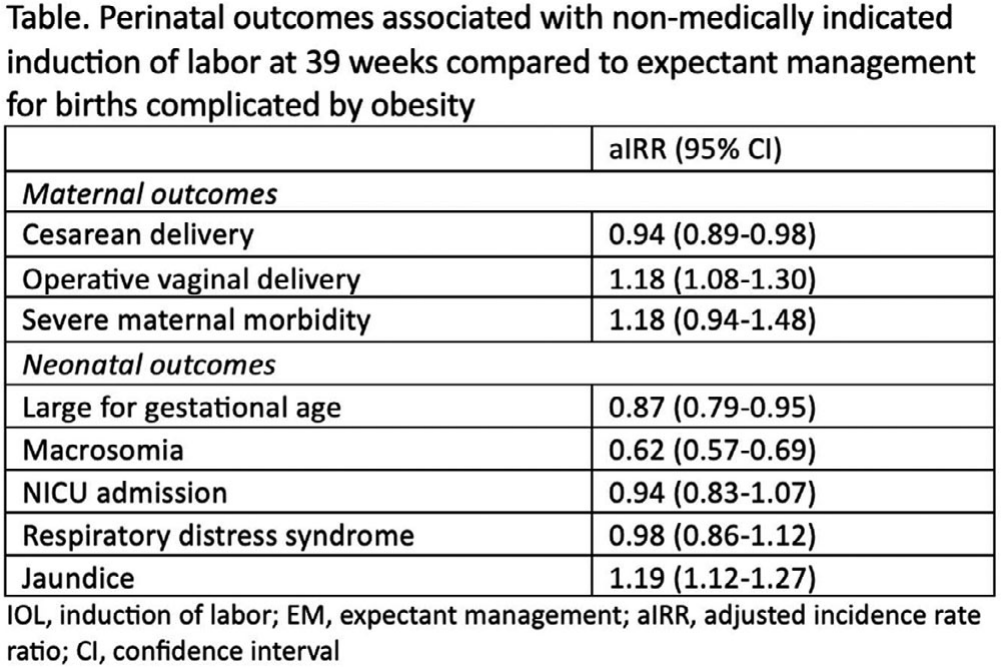

**3:30 PM – 5:00 PM**

## 1023 | **Betamethasone Administration Among Neonates at 22 Weeks Gestational Age: A Cost‐Effectiveness Analysis**

Amanda M. Baucom^1^; Robert M. Rossi^2^; Aaron B. Caughey^3^; Mark H. Eckman^4^

^1^University of Cincinnati, Cincinnati, OH; ^2^Department of Obstetrics and Gynecology Division of Maternal‐Fetal Medicine University of Cincinnati College of Medicine, Cincinnati, OH; ^3^Oregon Health & Science University, Oregon Health & Science University, OR; ^4^University of Cincinnati College of Medicine, University of Cincinnati College of Medicine, OH

**Objective**: The American College of Obstetricians and Gynecologists (ACOG) and the Society for Maternal‐Fetal Medicine (SMFM) have updated guidelines to recommend considering the initiation of betamethasone at 22 weeks' gestation. While previous studies have assessed the cost‐effectiveness of resuscitation methods at 22 weeks, the long‐term impact of betamethasone administration on neurological outcomes and survival from a cost‐effectiveness perspective remains unknown. This study aims to evaluate the cost‐effectiveness of initiating betamethasone at 22 weeks gestation, focusing on its influence on long‐term neurological outcomes and survival.

**Study Design**: A decision‐analytic model was developed using TreeAge software to compare survival rates and neurological outcomes in infants born at 22 weeks’ gestation, with and without the administration of betamethasone. Given that approximately 0.05% of deliveries in the United States occur at 22 weeks annually, the analysis was applied to a theoretical cohort of 1693 pregnant individuals. Costs were evaluated in 2025 U.S. dollars over a lifetime horizon, with effectiveness measured in neonatal and maternal quality‐adjusted life years (QALYs). Cost and utility values were derived from published literature. The incremental cost‐effectiveness ratio (ICER) was calculated as cost per QALY, using a willingness‐to‐pay threshold of $100,000 per QALY.

**Results**: In this modeled cohort, antenatal betamethasone administration resulted in an increase of 343 additional survivors. Among these, 289 infants survived with mild or no neurological impairment. In contrast, withholding betamethasone led to 300 survivors, and only 135 with mild or no neurological impairment. Overall, betamethasone administration provided an additional 2.92 QALYs per neonate compared to no administration. The total costs were $389,642,460 more with a difference of 4961 QALYs. The ICER was 78,684, which was cost effective.

**Conclusion**: In this theoretical model, antenatal betamethasone administration for infants born at 22 weeks’ gestation was found to be cost‐effective compared to no administration.

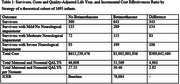

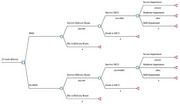

**3:30 PM – 5:00 PM**

## 1024 | **Adverse Outcomes in Individuals With Recurrent Gestational Diabetes Mellitus in Subsequent Pregnancies**

Amelia H. Gagliuso^1^; Bharti Garg^2^; Carolyn C. Green^3^; Aaron B. Caughey^2^

^1^Oregon Health and Science University, Portland, OR; ^2^Oregon Health & Science University, Oregon Health & Science University, OR; ^3^Oregon Health & Science University, Portland, OR

**Objective**: Gestational diabetes (GDM) makes up 8–10% of all annual pregnancies in the United States. A prior diagnosis of GDM in a previous pregnancy is a well‐established risk factor for recurrence in subsequent pregnancies. However, data examining maternal and neonatal outcomes specifically in the setting of recurrent GDM remains limited. The purpose of this study is to examine the risk of adverse maternal and neonatal outcomes among individuals with a current GDM diagnosis and a history of GDM in a prior pregnancy.

**Study Design**: A retrospective cohort study using linked birth certificate hospital discharge data in California from 2008–2020 was conducted to examine the association of recurrent preeclampsia with adverse maternal and neonatal outcomes. We included singleton, non‐anomalous births with gestational age of 23–42 weeks. Chi‐square tests and multivariable Poisson regression models were employed to evaluate the association between recurrent GDM and the risk of adverse outcomes when compared to those without recurrent GDM.

**Results**: Overall, among those with GDM in their first pregnancy, 56% had recurrent GDM in the subsequent pregnancy (*n* = 37,928). Within the recurrent GDM population, 46% of patients were categorized as obese, 26% as overweight, 26% as normal weight, and 1% underweight. Recurrent GDM was associated with increased risk of preterm birth (aRR 1.18, 95% CI 1.13–1.25) and preeclampsia (aRR 1.33, 95% CI 1.26–1.40). Neonates born to mothers with recurrent GDM had higher risk of NICU admission (aRR 1.18, 95% CI 1.13–1.24), large for gestational age (aRR 1.21, 95% CI 1.17–1.25), shoulder dystocia (aRR 1.24, 95% CI 1.11–1.40), and brachial plexus injury (aRR 2.36, 95% CI 1.53–3.63).

**Conclusion**: Recurrent gestational diabetes mellitus is linked to a range of adverse maternal and neonatal outcomes, including preeclampsia, cesarean delivery, and complications related to fetal overgrowth. These results emphasize the importance of early identification and individualized care for patients with a history of GDM to mitigate risks in subsequent pregnancies.

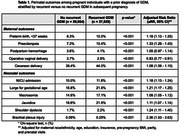

**3:30 PM – 5:00 PM**

## 1025 | **Diurnal Variability of Urine Protein‐to‐Creatinine Ratio in Singleton and Twin Pregnancies Versus 24‐Hour Proteinuria**

Amir Naeh^1^; Basel Habib Nasser^2^; Eiman Shalabna^1^; Rawia Hussein‐Aro^1^; Milana Gelman^3^; Noa Haggiag^1^; Moran Gawie‐Rotman^1^; Adi Malkoff Rabin^4^; Rinat Gabbay‐Benziv^5^

^1^Hillel Yaffe Medical center, Hadera, HaZafon; ^2^Hillel Yaffe Medical center,Hadera, Israel; Rappaport Faculty of Medicine, Technion ‐ Israel Institute of Technology, Haifa, Israel, Hadera, HaZafon; ^3^Department of Obstetrics and Gynecology, Hillel Yaffe Medical Center, Netanya, HaMerkaz; ^4^Hillel Yaffe Medical center, Binyamina, HaZafon; ^5^Hillel Yaffe Medical Center, Hillel Yaffe Medical Center, HaMerkaz

**Objective**: To evaluate the diagnostic performance and diurnal variability of the urine protein‐to‐creatinine ratio (Upcr) in detecting proteinuria among singleton and twin pregnancies.

**Study Design**: We conducted a retrospective cohort analysis of all pregnant women who underwent both Upcr testing and 24‐hour urine protein collection (24hUpc) between January 2021 and March 2024 at a single university‐affiliated medical center. Upcr samples were categorized based on collection time as daytime (6:00 AM–6:00 PM) or nighttime (6:00 PM–6:00 AM). The diagnostic accuracy of Upcr for detecting significant proteinuria, defined as 24hUpc >300 mg/day, was assessed using receiver operating characteristic (ROC) curves and area under the curve (AUC) calculations.

**Results**: Out of 13,054 deliveries during the study period, 276 cases (2.1%) had paired Upcr and 24hUpc measurements—253 (91.7%) singleton pregnancies and 23 (8.3%) twin pregnancies. Daytime samples were more prevalent (178 vs. 98). Upcr demonstrated good diagnostic performance in identifying proteinuria in both singleton (AUC = 0.835) and twin (AUC = 0.829) pregnancies. Overall, Upcr values were lower in twin pregnancies, regardless of sampling time, though twin gestations exhibited higher 24hUpc levels during daytime collections. In singleton pregnancies, daytime Upcr had superior diagnostic accuracy compared to nighttime measurements (AUC: 0.854 vs. 0.806). Conversely, in twin pregnancies, nighttime Upcr outperformed daytime values (AUC: 0.833 vs. 0.802). As expected, twins delivered at an earlier gestational age but had comparable rates of hypertensive disorders to singletons.

**Conclusion**: Upcr is a reliable tool for proteinuria assessment in both singleton and twin pregnancies. Twin pregnancies consistently show lower Upcr values, yet nighttime Upcr measurements provide better diagnostic accuracy in this group, suggesting a diurnal effect. These findings highlight the importance of considering sampling time when interpreting Upcr results, particularly in twin gestations, and warrant further research into its clinical implications.

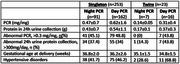

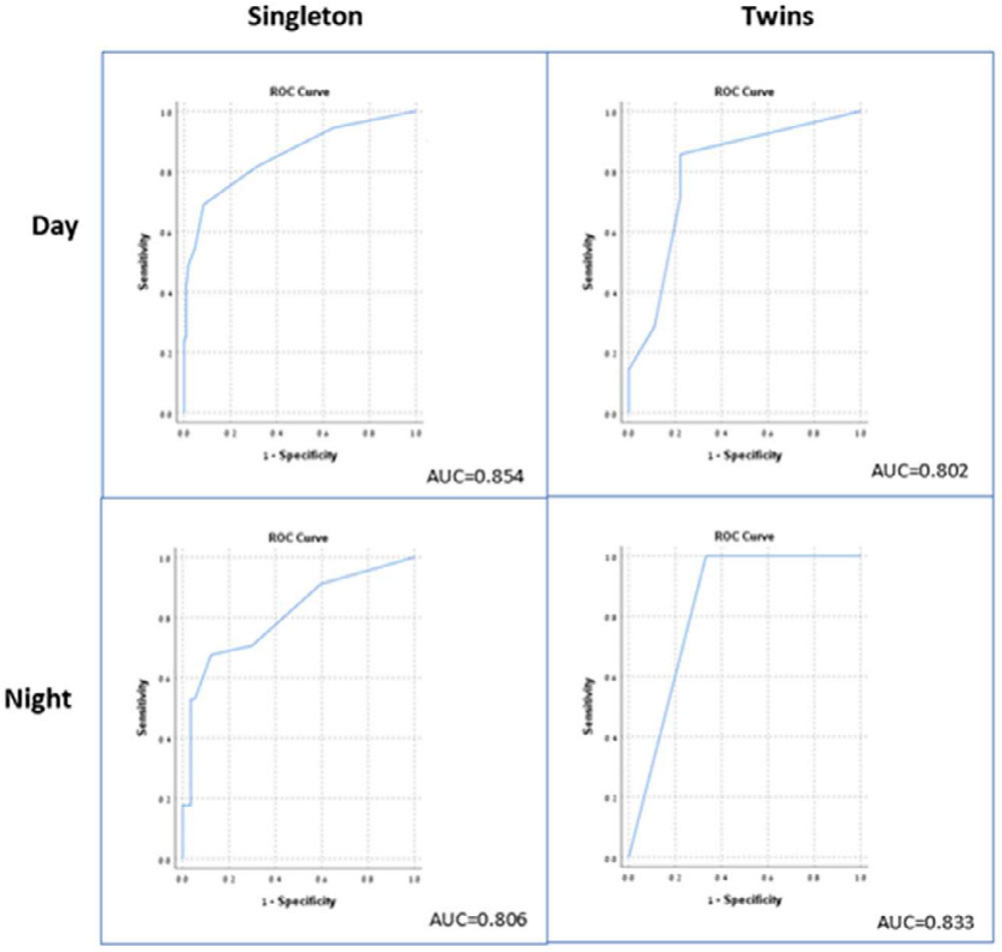

**3:30 PM – 5:00 PM**

## 1026 | **Enhancing Multispecialty Competence in Maternal Trauma Resuscitation Through Simulation‐Based Training**

Ana Collins‐Smith

University of Texas Medical Branch, Galveston, TX

**Objective**: Following the implementation of restrictive abortion legislation in Texas, healthcare providers anticipate a rise in pregnancy‐related complications, including maternal trauma. Given the rarity of severe, life‐threatening trauma during pregnancy, resident exposure to its management is limited. To address this educational gap, a multidisciplinary simulation‐based training and educational curriculum was developed to improve resident familiarity with the management of trauma in pregnancy.

**Study Design**: Residents from a single academic institution participated in a didactic lecture followed by a multidisciplinary, low‐fidelity simulation of maternal trauma. The simulation was conducted in a functioning trauma bay and included collaboration among nursing staff, residents, faculty, and medical students from pediatrics, emergency medicine, trauma surgery, anesthesia, and obstetrics. Participants completed a knowledge assessment both before and after the simulation.

**Results**: A total of 30 residents completed both pre‐ and post‐simulation assessments. Prior to the intervention, 57.2% correctly identified the components of the primary and secondary survey, 71.5% understood the modifications required for trauma resuscitation during pregnancy, and 42.9% were unaware of the preferred abdominal entry method for surgical intervention. Following the educational session and simulation, 80% of participants accurately described the primary and secondary survey components, 90% could articulate the adaptations necessary for resuscitation in pregnancy, and 86% correctly identified the optimal surgical approach in maternal trauma.

**Conclusion**: Simulation‐based training significantly improved resident knowledge and preparedness for managing maternal trauma. Low‐fidelity, interdisciplinary simulation serves as a valuable educational tool to address training gaps and may contribute to improved outcomes in the care of pregnant trauma patients.

**3:30 PM – 5:00 PM**

## 1027 | **Treating Vaginal Infections Prior to History‐Indicated Cerclage and Its Impact on PPROM and Pregnancy Prolongation**

Andy Chen; Gilad A. Gross

SSM Health/St. Louis University School of Medicine, St. Louis, MO

**Objective**: To evaluate the effect of infectious vaginitis treatment in patients undergoing history‐indicated cerclage. Gestational age at cerclage, preterm premature rupture of membranes (PPROM), gestational age at PPROM, time from cerclage to PPROM, gestational age at delivery, and time from cerclage to delivery were evaluated for patients who received treatment for bacterial vaginosis, chlamydia, trichomonas and yeast versus those who tested negative prior to cerclage.

**Study Design**: A retrospective cohort study analyzing all history‐indicated cerclages performed at a single institution between 2008–2020 (*n* = 333) was performed. Two‐sample t‐tests and ANOVA analyses were utilized to assess for statistical significance between the treated (*n* = 68) and control patient (*n* = 265) groups, and among the different vaginal infections for each study parameter, respectively.

**Results**: There was no statistically significant difference in the timing of cerclage placement between the treated and negative group (15.2 weeks vs. 14.8 weeks, *p* = 0.06). Patients who were treated for vaginal infections prior to cerclage had a higher incidence of PPROM than those who tested negative (26.5% vs 17.4%, *p* = 0.04). Treatment of vaginal infections was associated with PPROM at a later gestational age (31.5 weeks vs. 27.7 weeks, *p* = 0.01) and a longer cerclage‐to‐PPROM interval than those who tested negative (15.8 weeks vs. 12.9 weeks, *p* = 0.04). There was no statistically significant difference between the treated and negative groups in gestational age at delivery (35.9 weeks vs. 36.1 weeks, *p* = 0.42) and time from cerclage to delivery (20.8 weeks vs. 21.3 weeks, *p* = 0.24). The type of infection and the presence of co‐infection did not have any statistically significant influence on any variables in this study.

**Conclusion**: Treating vaginal infections prior to placement of history‐indicated cerclage is associated with a higher rate of PPROM, but is associated with an overall comparable or longer prolongation of pregnancy as well as reducing prematurity versus patients who tested negative for vaginal infection prior to cerclage placement.

**3:30 PM – 5:00 PM**

## 1028 | **Maternal and Fetal Outcomes and Anticoagulation Use in Pregnancies Affected by Mechanical Heart Valves**

Angela Essa^1^; Lara C. Kovell^2^; Gianna L. Wilkie^3^

^1^University of Massachusetts Chan ‐ Baystate Medical Center, Easthampton, MA; ^2^University of Massachusetts Chan School of Medicine, Worcester, MA; ^3^University of Massachusetts Chan Medical School, Worcester, MA

**Objective**: Patients with mechanical heart valves in pregnancy are at a substantially higher risk of adverse maternal and fetal outcomes. Management of patients with mechanical heart valves poses a unique challenge, and strategies for anticoagulation in these patients remain controversial. The goal of this project was to evaluate rates of adverse outcomes and types of anticoagulation used in pregnancies affected by mechanical heart valves.

**Study Design**: A query was performed using Epic Cosmos to evaluate pregnancies affected by mechanical heart valves between 12/13/2021 and 12/12/2024. Patient demographics and rates of adverse cardiac outcomes in the perinatal period, including heart failure, arrhythmia, and stroke were assessed. The rates of anticoagulation with enoxaparin and coumadin were evaluated. Perinatal outcomes, including the rate of preterm delivery (<37 weeks), cesarean delivery, and neonatal demise were also evaluated. Rare outcomes were reported as <10 to protect patient privacy.

**Results**: A total of 398 patients with 486 pregnancies were identified. The majority of patients were white (72.9%), with 11.6% Hispanic. Regarding cardiac outcomes, the presence of a mechanical heart valve in pregnancy was associated with maternal arrhythmia (54.0%), heart failure (31.9%), and stroke (10.1%). Maternal death occurred in fewer than 10 patients. The preterm birth rate was 19.3%, and the cesarean delivery rate was 30.9%. Overall, 55.8% of patients received enoxaparin and 43.2% received coumadin during the pregnancy.

**Conclusion**: The presence of a mechanical heart valve in pregnancy is associated with high rates of adverse maternal and fetal outcomes, including heart failure and stroke. Further larger studies are needed to delineate the timing and the type of anticoagulation used in pregnancy, as well as risk factors associated with worsened outcomes in this population.

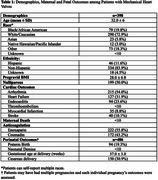

**3:30 PM – 5:00 PM**

## 1029 | **Implementation of Respectful Care Breakfasts Supports Statewide Perinatal Quality Improvement Initiative Equitable Care Efforts**

Patricia Lee King^1^; Maria Villagomez^2^; Vincent Mambo^3^; Lavisha Singh^4^; Alana Rivera^2^; Ann E Borders^2^

^1^Northwestern University Feinberg School of Medicine, Chicago, IL; ^2^Endeavor Health, Evanston Hospital, Evanston, IL; ^3^Northwestern University, Chicago, IL; ^4^Endeavor Health, Endeavor Health Evanston Hospital, IL

**Objective**: The Illinois Perinatal Quality Collaborative's (ILPQC) Birth Equity (BE) quality improvement (QI) initiative launched in July 2021 to implement actionable strategies to address birth equity across Illinois birthing hospitals. Respectful Care Breakfasts (RCB) were designed to promote discussion of respectful care experiences and opportunities to improve care with patients/families who delivered in the past year and OB clinical staff. We aim to describe hospital team progress towards implementing RCBs from their introduction mid‐initiative (Jan‐Jun 2023) through early sustainability (Jan‐Jun 2025) and identify themes from participants’ experiences.

**Study Design**: Hospitals submitted monthly data, including whether a RCB was held and the number and type of participants. Chi‐square tests were used to evaluate differences in hospitals’ characteristics of those that completed a RCB and those who did not. A post‐event survey was distributed to assess participant experiences and inform future RCBs.

**Results**: 77 hospitals participated in the BE initiative and 56 hospitals (73%) hosted at least one RCB between Jan 2023 to June 2025. Hospitals hosting a RCB increased from 5% in Jan‐Jun 2023 to 48% in Jan‐Jun 2025. No significant differences in hospital characteristics were found between those that hosted a RCB and those that did not (Table 1). Among 333 post‐event participant surveys, 96.7% of participants rated enjoyment of the RCB at 4–5 and 88.6% rated learning at 4–5 on a 5‐point scale. Patients valued open and honest dialogue and the opportunity to share experiences and feel heard. Clinical staff valued hearing patient stories, feedback and learning strategies to improve respectful care (Table 2). Personal invitations to patients from trusted providers increased patient participation and scheduling for breakfast/lunch increased clinical team participation.

**Conclusion**: The results suggest that a statewide collaborative can facilitate and sustain equitable implementation of RCBs through a QI initiative. Hospitals can use RCBs to promote respectful care improvements regardless of hospital characteristics.

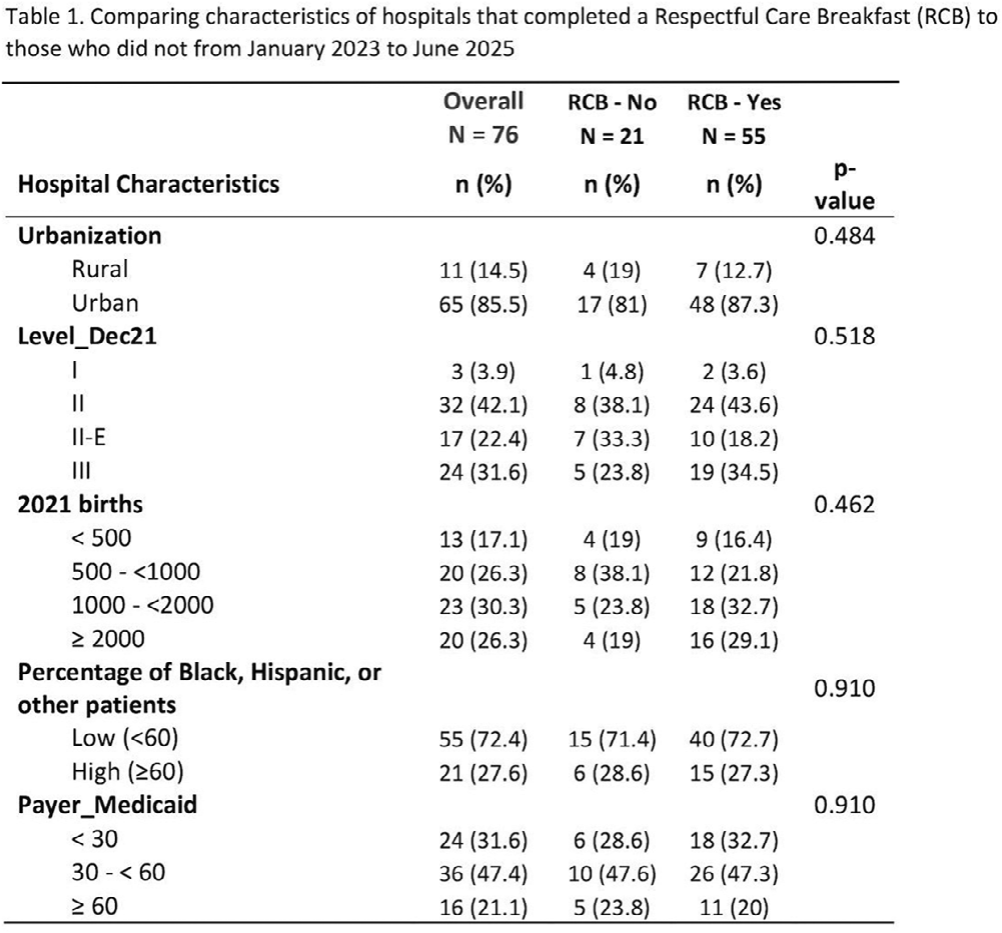

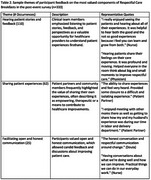

**3:30 PM – 5:00 PM**

## 1030 | **Cost‐Effectiveness of Early Fetal Anomaly Screening (11–14 Weeks) in Pregnancies Complicated by Pregestational Diabetes Mellitus**

Anna J. Bishop^1^; Amelia H. Gagliuso^2^; Lily Ben‐Avi^3^; Aaron B. Caughey^3^

^1^Oregon Health & Science University, Portland, OR; ^2^Oregon Health and Science University, Portland, OR; ^3^Oregon Health & Science University, Oregon Health & Science University, OR

**Objective**: Second trimester anomaly screening is currently the standard‐of‐care for the general population, although many congenital anomalies can already be detected in the first trimester of pregnancy via ultrasound. This results in some abnormalities being identified later in the second trimester, when pregnancy termination may no longer be an option and is associated with greater cost and risk. This study aims to investigate the cost‐effectiveness of first‐trimester anomaly screening in pregestational diabetes, which is a high‐risk condition with an elevated prevalence of fetal anomalies, by enabling earlier decisions regarding pregnancy termination or fetal intervention.

**Study Design**: We designed a decision‐analytic model using TreeAge Pro 2025 software to compare first trimester vs. second trimester ultrasound screening for a theoretical cohort of 31,347 women with pre‐gestational diabetes mellitus. The average cost for a fetal anatomy scan was $497. Primary outcomes were 1st trimester scan anomaly detection rate, total anomalies correctly detected, pregnancy loss, NICU stay, neonatal mortality, cost, and quality‐adjusted life years (QALYs). Model inputs were obtained from the literature and varied in sensitivity analyses, with a cost‐effectiveness threshold of $100,000/QALY.

**Results**: In our theoretical cohort of 31,347 pregnant persons with pregestational DM, a total of 380 more anomalies were detected in the first trimester strategy when compared to the standard‐of‐care second trimester strategy. Regarding neonatal outcomes, there were 337 less pregnancy losses, 113 less NICU stays, and 69 less cases of neonatal mortality in the 1st trimester group. These outcomes led to a $47,872,412 reduction in cost and 344 additional QALYs in the first‐trimester strategy compared to the second‐trimester strategy.

**Conclusion**: Early fetal anomaly screening for women with pregestational diabetes resulted in lower costs and higher QALYs, representing a dominant strategy that was cost‐effective.

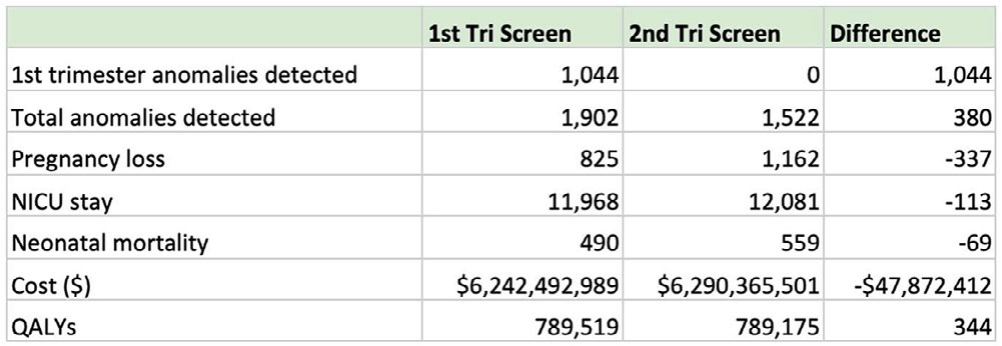

**3:30 PM – 5:00 PM**

## 1031 | **Feasible but Fleeting: Exploring Response Patterns in Remote Postpartum BP Monitoring Among Underserved Patients**

Anna Madden‐Rusnak^1^; Miriam J. Alvarez^1^; Alejandro Guevara^2^; Nandini Raghuraman^3^; George A. A. Macones^1^; Lorie M. Harper^1^; Alison G. G. Cahill^4^

^1^Department of Women's Health, Dell Medical School at the University of Texas at Austin, Austin, TX; ^2^Texas Tech University Health Sciences Center Paul L. Foster School of Medicine, El Paso, TX; ^3^Washington University School of Medicine, St. Louis, MO; ^4^Department of Women's Health, Dell Medical School at the University of Texas at Austin, Department of Women's Health, Dell Medical School at the University of Texas at Austin, TX

**Objective**: Postpartum home blood pressure (BP) monitoring is commonly used to balance the need for continued surveillance in pregnancies complicated by hypertension with the goal of timely discharge after delivery. This study explored the real‐world feasibility of twice‐daily home blood pressure monitoring in the critical first two weeks postpartum among underserved, primarily Spanish‐speaking individuals with hypertensive disorders.

**Study Design**: A prospective, observational pilot study enrolled postpartum persons diagnosed with gestational hypertension (gHTN), preeclampsia (Pre‐E), or chronic hypertension (cHTN) for remote BP monitoring. Participants self‐monitored BP twice daily via text message prompts over two weeks, with automated real‐time feedback. Response rates were calculated across the sample. A generalized linear mixed effect model assessed compliance by time of day (am or pm), week, and diagnosis.

**Results**: Seventy‐five postpartum individuals were enrolled; most were Hispanic (87%), Spanish‐speaking (72%), and publicly insured (88%). Diagnoses included gHTN (51%), Pre‐E (40%) and cHTN (9%). Eighty‐five percent submitted at least one BP reading during the study period, with a mean of 12 ± 8.4 readings each. Of 2100 text prompts, 786 (37%) received responses. Response rates dropped in Week 2 (OR = 0.38, 95% CI: 0.30–0.48, *p* < 0.001). Time of day (*p* = 0.72  ) and diagnosis (*p* = 0.77) were not significant predictors of response.

**Conclusion**: This pilot study shows the feasibility and limits of remote BP monitoring in an underserved high‐risk population. Although overall response was modest, higher initial engagement supports short‐term monitoring. Participation dropped after one week, indicating the need for brief, focused interventions. Future research should address barriers like caregiving demands, digital literacy, or post‐delivery recovery. With refinement, home BP monitoring can be a scalable way to improve postpartum care and reduce disparities.

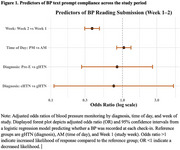

**3:30 PM – 5:00 PM**

## 1032 | **Carrier Screening Trends in Pregnancy at a Large Academic Center**

Anna Ulrey^1^; Marcia Des Jardin^2^; Jordan Garcia^1^; Lianhua Jin^2^; Dhong‐Jin Kim^3^; Lynda G. Ugwu^2^; Angela R. Seasely^4^; Akila Subramaniam^2^

^1^Department of Obstetrics and Gynecology, University of Alabama at Birmingham, University of Alabama at Birmingham, AL; ^2^Center for Research in Women's Health, University of Alabama at Birmingham, Birmingham, AL; ^3^Center for Research in Women's Health, University of Alabama at Birmingham, University of Alabama at Birmingham, AL; ^4^Fort Sanders Perinatal Center, Fort Sanders Perinatal Center, TN

**Objective**: Carrier screening in pregnancy is universally recommended for reproductive risk assessment. However, uptake is low. In 2021, our single institution partnered with a commercial lab to offer carrier screening with reflexive noninvasive fetal risk assessment at no cost to individuals with government insurance. Private paying individuals continued to have routine coverage, with out‐of‐pocket costs. As such, we sought to evaluate temporal trends in carrier screening uptake at a single center from 2018 to 2023 and variables associated with changing temporal trends.

**Study Design**: We conducted a retrospective cohort study of all patients who received prenatal care at a single institution 2018–2023. Patient demographics and clinical characteristics were collected (Table 1). The primary outcome was receipt of carrier screening. Rates were compared by calendar year using Chi‐square test for trend. Patient characteristics were compared between those who did and did not receive carrier screening (unadjusted odds ratio [OR] with 95% confidence intervals [CI]); trends in differences for characteristics over time were assessed with the Breslow Day Test (BDT).

**Results**: Of 30,570 pregnancies, 20,165 pregnancies met study criteria. Screening rates were <2% from 2018–2020 but increased four‐fold following the industry partnership to 32.4% in 2022 and 26.8% in 2023 (Figure 1; *p* < 0.0001). Patients who self‐identified as Black, were multiparous, less than 35 years of age, had government insurance, and received prenatal care at publicly funded clinics had significantly increased odds of receiving carrier screening (Table 1). Over time, all of these characteristics were significantly associated with increased receipt of carrier screening (BDT *p* < 0.0001 for all).  Of note, patients with private insurance continued to have lowest rates of carrier screening uptake over time (*p* < 0.001).

**Conclusion**: The implementation of a no‐cost industry‐supported model was associated with a rapid and sustained increase in carrier screening uptake among publicly insured and historically under‐screened populations.

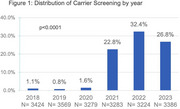

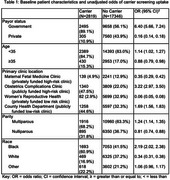

**3:30 PM – 5:00 PM**

## 1033 | **Postpartum Pain Scores in Patients Maintained on Medication for Opioid Use Disorder (MOUD)**

Anne Claire Burghard^1^; Zenobia Gonsalves^2^; Rachel Lee^3^; Francesca DeIeso‐Frechette^4^; Chaitali Korgaonkar‐cherala^5^; Lucinalda Perez Sanchez^1^; Maryam Tayyab^6^; David J. Garry^7^; Kimberly Herrera^3^; Cassandra Heiselman^3^

^1^Renaissance School of Medicine at Stony Brook University, Stony Brook, NY; ^2^Stony Brook University Hospital, Great Neck, NY; ^3^Stony Brook University Hospital, Stony Brook, NY; ^4^Renaissance School of Medicine at Stony Brook University, Renaissance School of Medicine at Stony Brook University, NY; ^5^Stony Brook Medicine, Stony Brook, NY; ^6^Renaissance School of Medicine at Stony Brook University, Hauppague, NY; ^7^Stony Brook Medicine, Stony Brook Medicine, NY

**Objective**: To determine the frequency of severe postpartum pain in patients with OUD maintained on methadone and buprenorphine.

**Study Design**: This was a single‐center retrospective cohort study of women with a history of OUD on MOUD who presented for prenatal care or delivery between January 2017 to July 2023. Patient, pregnancy, and delivery outcomes were abstracted. High dose MOUD was defined as buprenorphine >16mg and methadone >100mg. Patients were compared based on their MOUD. The primary outcome was the number of times a severe pain score (score of 7 or greater on a 10‐point scale) was reported from delivery to discharge.  Statistical analyses were performed by Mann‐Whitney U Test, chi‐square test, and multivariate logistic regression, with significance determined as *p* < 0.05.

**Results**: 419 women were included in the study. Compared to buprenorphine, more patients on methadone had not initiated prenatal care in the first trimester (*p* = 0.004), had active drug use (*p* = < 0.001), polysubstance use (*p* < 0.001), required a high dose of MOUD (*p* < 0.001) and increase on MOUD (*p* < 0.001) dose at delivery (Table 1). MOUD was not associated with a difference in postpartum pain scores across all timepoints (Figure 1), and was not an independent predictor of severe pain scores. Polysubstance abuse was an independent predictor of severe pain scores at any point postpartum (aOR 2.0, 95%CI [1.3–3.2], p‐value = 0.04). Psychiatric medication use was an independent predictor of severe pain scores on PPD#0 (aOR 1.65, 95%CI [1.1–2.5], p‐value = 0.016). Both polysubstance use (aOR 1.9, 95%CI [1.2–3.0], p‐value = 0.03) and psychiatric medication use (aOR 1.5, 95%CI [1.0–2.3], p‐value = 0.047) were independent predictors for severe pain scores on PPD#2.

**Conclusion**: While MOUD does not influence the severity of postpartum pain, differences in comorbid factors—such as polysubstance use and psychiatric medication use—may impact pain experiences.

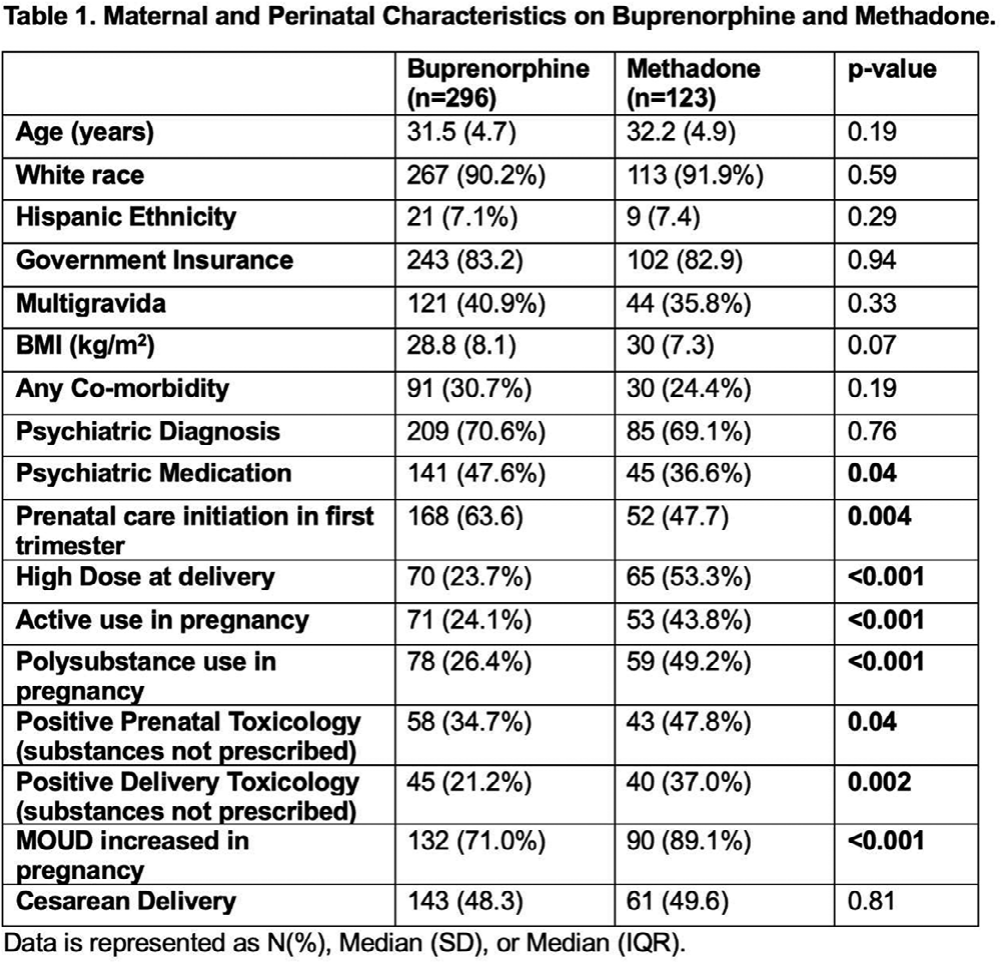

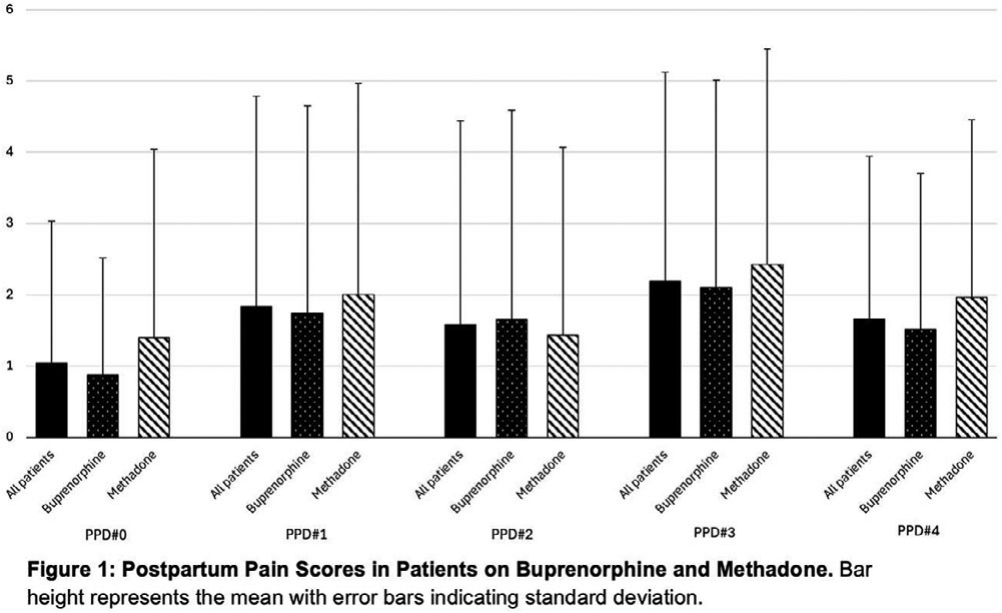

**3:30 PM – 5:00 PM**

## 1034 | **Effect of Antenatal Corticosteroids on Placental Histology in Growth‐Restricted Fetuses**

Annie Rozenblyum^1^; Julia Kim^1^; Amberly Lao^2^; Justin Gimoto^1^; Maria Gomez^1^; Soo Young Kim^1^; Poonam Khullar^3^; Patricia Rekawek^1^; Martin Chavez^1^; Lakha Prasannan^4^

^1^NYU Grossman Long Island School of Medicine, Mineola, NY; ^2^NYU Langone Hospital‐ Long Island, NYU Langone Hospital‐ Long Island, NY; ^3^Department of Pathology, NYU Grossman School of Medicine, Mineola, NY; ^4^NYU Long Island, Long Island City, NY

**Objective**: Antenatal corticosteroids (ACS) are frequently administered to patients with fetal growth restriction (FGR) due to risk of preterm delivery; however, data on the histopathologic impact of ACS exposure on the placenta in this context are limited. The objective of this study was therefore to evaluate placental pathology in pregnancies complicated by FGR and exposed to ACS.

**Study Design**: This was a retrospective cohort study of singleton pregnancies complicated by FGR with confirmed small for gestational age (SGA) neonates at a single institution from 2020 to 2024. FGR was defined as an estimated fetal weight (EFW) and/or abdominal circumference (AC) <10th percentile. Chromosomal abnormalities, major fetal anomalies, multiple gestations, delivery prior to 24 weeks, or discarded placentas were excluded. ACS exposure was defined as receiving either a complete or incomplete course of betamethasone. ANOVA and X^2^ tests with *p* < 0.05 were used.

**Results**: Among 111 pregnancies complicated by FGR, 93 were exposed to ACS and 18 were not. Those with ACS exposure were more likely to have severe FGR at time of delivery (50% vs 11%, *p* = 0.008), abnormal umbilical artery dopplers (43% vs 11%, *p* = 0.01) and deliver earlier (35.1 vs 37.7 weeks *p* < 0.001).There were no significant differences in placental findings between the two groups. Increased rates of intraparenchymal hematoma (14% vs 0%), accelerated villous maturation (14% vs 0%), fetal vascular malperfusion (18.3% vs 5.6%) and segmental avascular villi (9.7% vs 0%) were noted, though not statistically significant.

**Conclusion**: No significant differences in placental pathology were identified between FGR pregnancies with and without ACS exposure, although trends toward increased placental abnormalities were noted in the ACS group. These findings may reflect greater disease severity rather than a direct effect of ACS. Future research should stratify by timing of ACS, particularly within the second trimester, to better assess the relationship between ACS exposure and placental pathology.

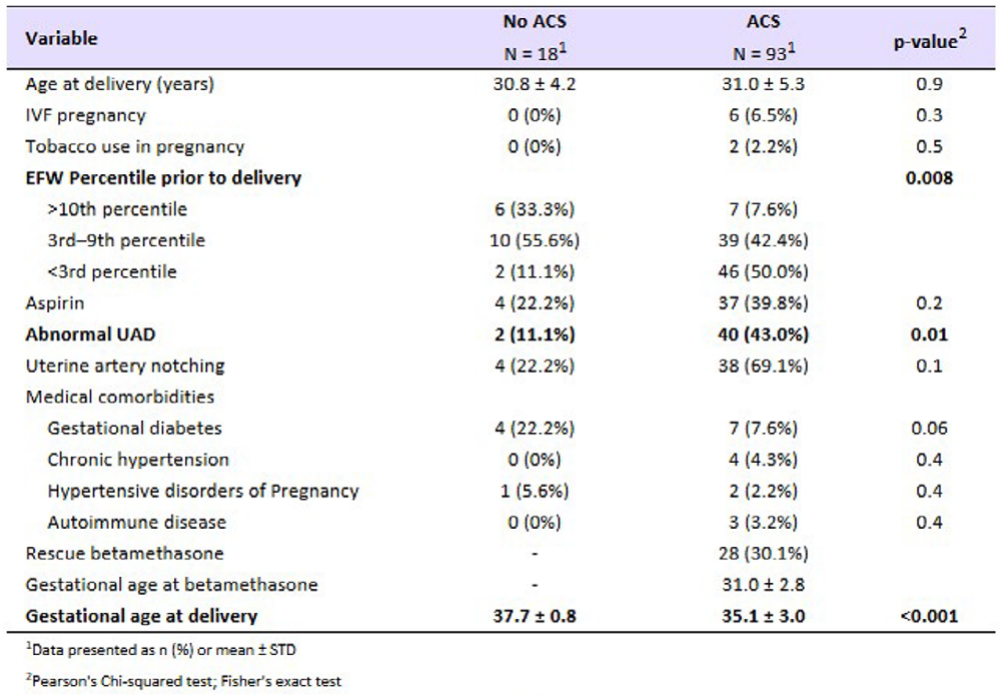

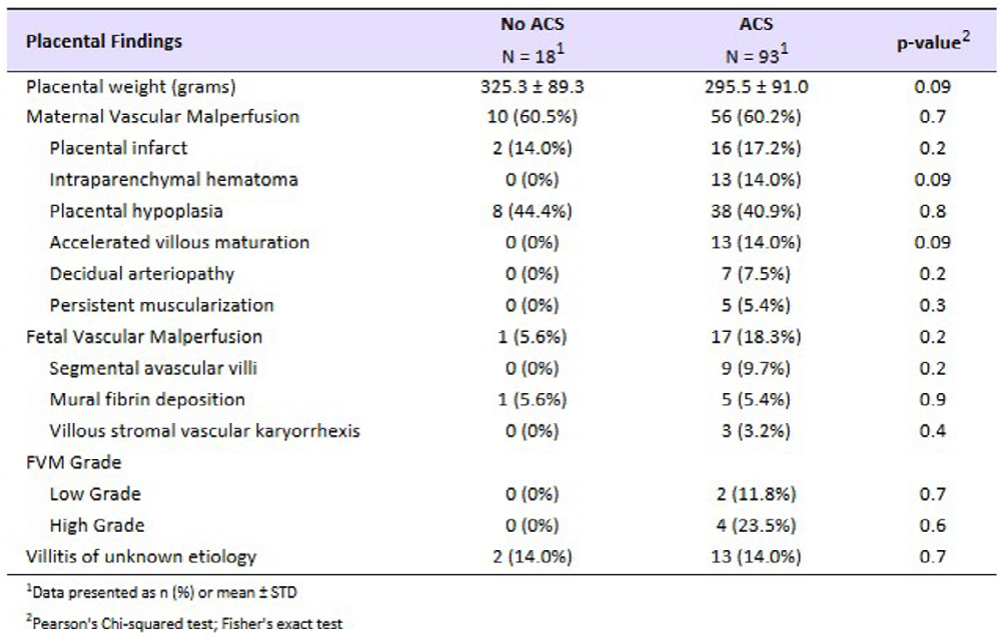

**3:30 PM – 5:00 PM**

## 1035 | **Adding Abdominal Circumference <10th Percentile to Estimated Fetal Weight <10th Percentile for Predicting Neonatal Morbidity**

Ashley Shea^1^; John Owen^2^; Katherine L. Grantz^3^; Marcia Des Jardin^1^; Kevin S. Shrestha^1^; Ashley Battarbee^1^

1Center for Research in Women's Health, University of Alabama at Birmingham, Birmingham, AL; ^2^Center for Women's Reproductive Health Department of Obstetrics and Gynecology University of Alabama at Birmingham, Birmingham, AL; ^3^Eunice Kennedy Shriver National Institute of Child Health and Human Development, National Institutes of Health, Bethesda, MD

**Objective**: SMFM and ACOG added abdominal circumference (AC) <10th %ile to estimated fetal weight (EFW) <10th %ile to diagnose fetal growth restriction (FGR); however, whether this addition improves neonatal morbidity prediction is unknown. Our objective was to determine if using AC <10th %ile or EFW <10th %ile (EFW+AC) was associated with improved prediction of composite adverse neonatal outcomes (CANO) and small‐for‐gestational‐age (SGA) at birth, compared to EFW <10th %ile alone.

**Study Design**: We performed a retrospective cohort study of pregnant patients at a tertiary care center who had a scan with complete fetal biometry <28 days before birth and delivered 1/2022–12/2023. Clinically significant anomalies and multiples were excluded. Both EFW and AC %iles were evaluated by NICHD charts. Outcomes were: 1) CANO (Table) and 2) SGA at birth. Logistic regression modeled the association of each FGR definition with outcomes and receiver operator characteristic area under the curve (AUC) compared predictive abilities.

**Results**: Of 3975 patients included, the mean GA at last ultrasound was 36.0±2.1 wks with delivery at mean GA 37.8±2.1 wks. Overall, 8.3% of patients met criteria for FGR by EFW+AC vs 7.4% by EFW alone (*p* < 0.0001); 16.5% were SGA and 29.7% had CANO. While both FGR definitions were associated with CANO (EFW+AC: RR 1.83, 95% CI 1.6–2.1; EFW alone: RR 1.85, 95% CI 1.6–2.1; *p* < 0.0001, Table), neither were good predictors of CANO (EFW+AC: AUC 0.54, 95% CI 0.53–0.55; EFW alone: AUC 0.54, 95% CI 0.53–0.55, *p* = 0.11). Both definitions had acceptable discrimination of SGA (Figure). Sensitivity analyses removing hyperbilirubinemia and hypoglycemia from CANO exhibited similar results (Table and Figure).

**Conclusion**: The addition of AC <10th %ile to EFW <10th %ile significantly increased the rate of FGR but did not improve the prediction of CANO compared to EFW alone. Given the increased use of prenatal resources to care for patients with FGR and the risk of iatrogenic premature birth, randomized clinical trials are warranted to determine if using the new expanded definition improves perinatal outcomes.

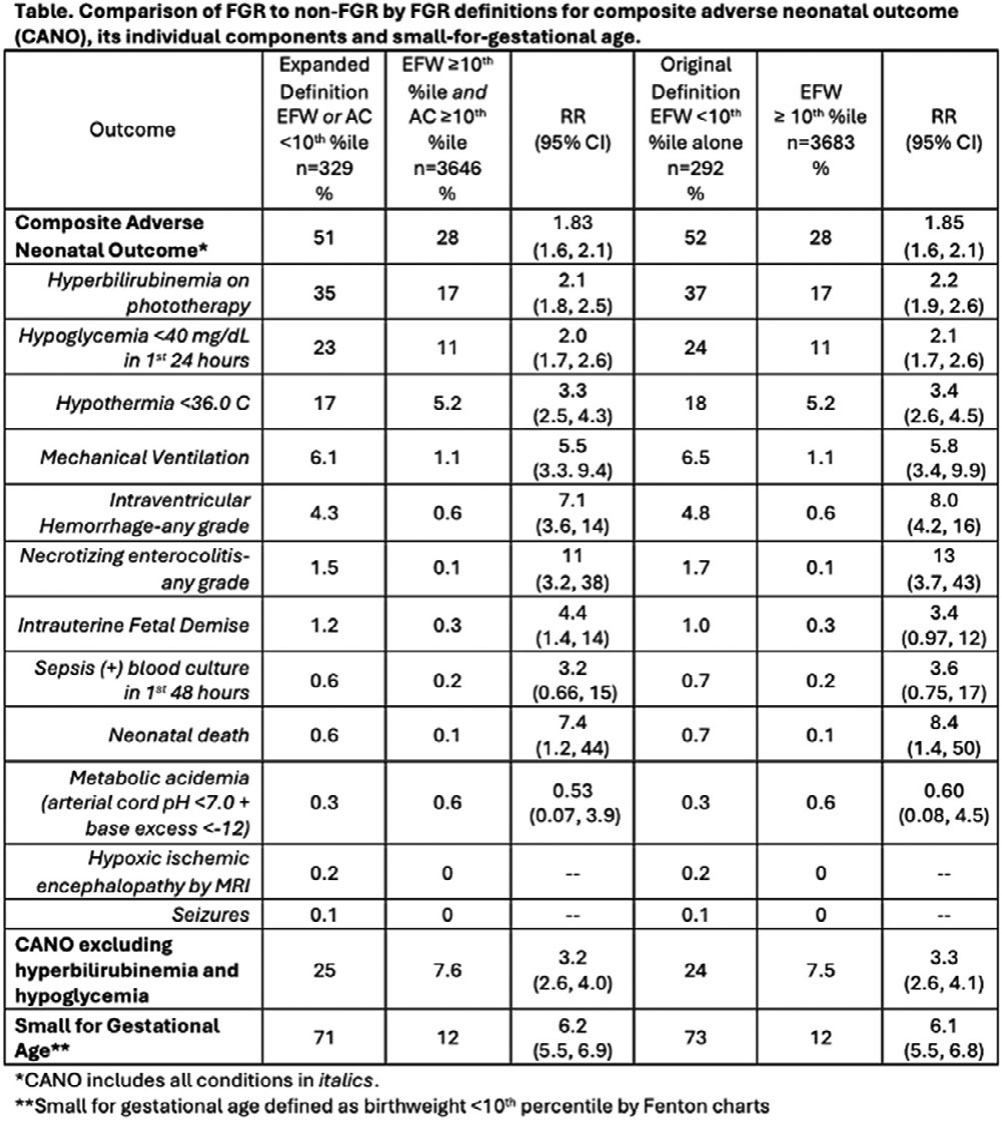

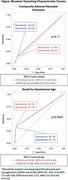

**3:30 PM – 5:00 PM**

## 1036 | **Severe Maternal Morbidity Associated With Timing and Absence of Prenatal Care**

Ashra Tugung^1^; Bo Park^2^; Nida Ali^1^; Sergio A. Karageuzian^3^; Havilah Reimche‐Vu^4^; Kriti N. Vedhanayagam^5^; Ilish Gedestad^3^; Neville Tritch^4^; Kevin H. Hu^5^; Ruofan H. Yao^5^

1Loma Linda University Children's Health | Perinatal Institute, Loma Linda, CA; ^2^California State University, Fullerton, Fullerton, CA; ^3^Loma Linda University Health, Loma Linda, CA; ^4^Loma Linda University School of Medicine, Loma Linda, CA; ^5^Loma Linda University, Loma Linda, CA

**Objective**: To examine the association between timing and absence of prenatal care and the risk of severe maternal morbidity (SMM)

**Study Design**: This population‐based, retrospective cohort study utilized the California Office of Statewide Health Planning and Development Linked Birth File with hospital discharge diagnoses coded in the International Classification of Diseases, 9th Revision, Clinical Modification (ICD‐9‐CM) between 2007 and 2012. The primary outcome was SMM, defined as any of the 25 peripartum conditions listed by the Centers for Disease Control and Prevention. Prenatal care was categorized as no care, early care (≤14 weeks gestation), or other care (  >14 weeks or unspecified). Chi‐square tests compared SMM incidence across care groups, and Cox proportional hazards models estimated risk relative to no care. A cumulative hazard plot was generated to illustrate timing of SMM events by prenatal care group.

**Results**: Of the 3,034,753 pregnancies analyzed, 44,270 (1.46%) were complicated by SMM. The highest burden occurred in pregnancies without prenatal care, with 48 of 284 affected (16.9%). By comparison, SMM was seen in 3441 of 173,605 pregnancies (1.98%) in women with other care and in 40,781 of 2,860,864 pregnancies (1.43%) in those who initiated care in the first trimester. Cox modeling demonstrated that any prenatal care was highly protective compared with none. Early care was associated with the lowest risk of SMM (HR 0.0148; 95% CI 0.0110–0.0199), and other care also conferred substantial protection (HR 0.0217; 95% CI 0.0161–0.0291; both *p* < 0.001). Cumulative hazard curves demonstrated that SMM risk rose after 30 weeks in pregnancies without prenatal care. In contrast, SMM risk stayed low throughout gestation for those receiving care, with early care providing the lowest risk.

**Conclusion**: Absence of prenatal care is strongly associated with SMM, whereas receiving any care is highly protective. Early care offers the lowest risk, with a slight advantage over other care. These findings highlight the importance of prenatal care and early engagement to prevent life‐threatening maternal complications.

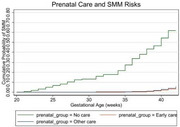

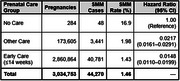

**3:30 PM – 5:00 PM**

## 1037 | **The First Two Seasons of Maternal RSV Immunization: Patients’ Acceptance, Satisfaction, and Costs**

Audra C. Fain^1^; Tina A. Nguyen^2^; Neil S Silverman^2^

^1^Department of Obstetrics and Gynecology, David Geffen School of Medicine at University of California, Los Angeles (UCLA), Los Angeles, CA; ^2^Division of Maternal Fetal Medicine, Department of Obstetrics and Gynecology, David Geffen School of Medicine at University of California, Los Angeles (UCLA), Los Angeles, CA

**Objective**: The respiratory syncytial virus (RSV) vaccine's efficacy to reduce infant RSV–related morbidity has been established, yet low rates of prenatal RSV vaccination were seen in the first 2 seasons of vaccine availability: 18% in 2023–24 and 38% in 2024–25 *(CDC RSV Vaxview)*. We sought to explore potential vaccine acceptance barriers, as well as identify factors impacting differences in patients’ attitudes and costs between the 2 seasons.

**Study Design**: An IRB‐approved protocol identified all patients in our health system receiving an RSV vaccine during either RSV seasons from 2023–25. RSV vaccination is available on‐site in all outpatient obstetric clinical areas in our system. Patients received an invitation to participate via their electronic patient portal. Data collected included demographics, other vaccinations, satisfaction metrics, billing and payment requests, and selected pregnancy outcomes.

**Results**: Among eligible patients, RSV vaccine acceptance rates surpassed national rates across seasons and showed a significant increase between the 2023 and 2024 season (Table 1). Satisfaction with on‐site vaccine availability was consistently high (4.5–4.7/5.0), with low rates of reported side effects (6–8%). Acceptability of future RSV vaccination was uniformly high across seasons. For out‐of pocket costs, 5–12% of patients received a bill after their vaccine; required payment decreased from 79% to 46% between seasons. When payment was required, 54% were responsible for > $100, similar between seasons. Preterm delivery rates were low and stable between seasons (5.8% vs 7.0%, *p* = 0.57), and were unrelated to interval between vaccination and delivery. Reported infant RSV infections declined between seasons (11.9% vs 6.6%, *p* = 0.04) but occurred significantly sooner after birth in 2024–25 (*p* < 0.001) and were more likely to result in hospitalization.

**Conclusion**: High rates of RSV vaccine acceptance and satisfaction were identified, with on‐site vaccine availability a strong factor in acceptance. Persistence of  billing and insurance concerns across seasons highlights coverage as a key barrier for vaccination.

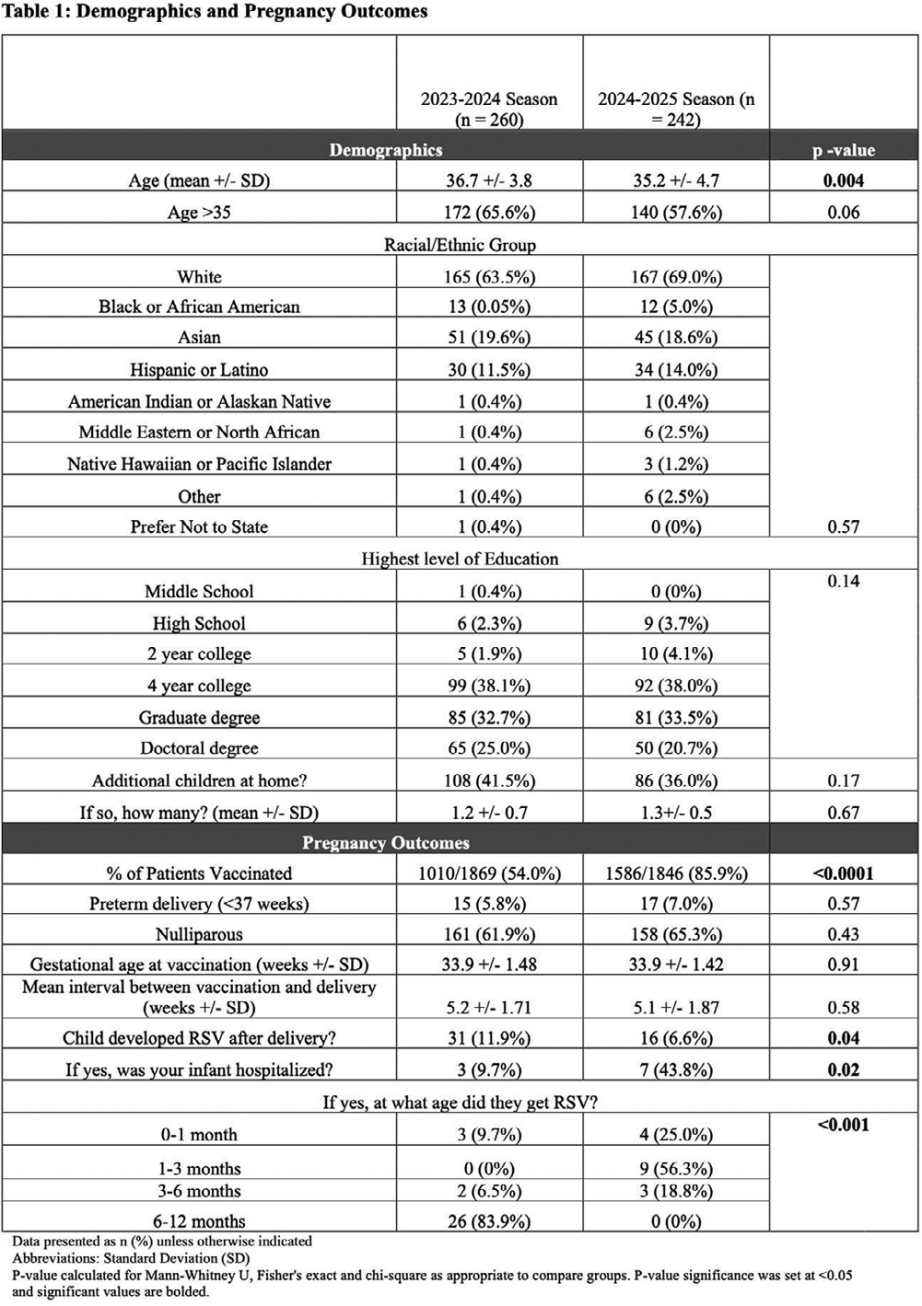

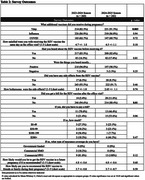

**3:30 PM – 5:00 PM**

## 1038 | **Accuracy and Readability of GPT‐4o Responses to Top Pregnancy FAQs**

Avish Arora^1^; Nawras Zayat^2^; Georgios Doulaveris^3^

1Montefiore/Albert Einstein College of Med., New York, NY; ^2^Montefiore‐Einstein, Bronx, NY; ^3^Montefiore Medical Center/Albert Einstein College of Medicine, Montefiore Medical Center/Albert Einstein College of Medicine, NY

**Objective**: To Quantify the accuracy, completeness, readability, and inter‐reviewer agreement of GPT‐4o answers to the most‐searched pregnancy questions.

**Study Design**: The 50 highest‐volume U.S. Google‐Trends pregnancy questions (April 2025) were each submitted twice to GPT‐4o (temperature 0, identical prompt). Two maternal‐fetal medicine clinicians, blinded to each other, graded the 100 answers with a four‐tier rubric: comprehensive & accurate (C‐A), accurate but incomplete, mixed correct/incorrect, completely incorrect, and recorded whether the two answers to a question differed materially. Flesch‐Kincaid grade level and Flesch Reading Ease were calculated for every answer. Cohen's κ described inter‐rater concordance.

**Results**: Of 200 individual ratings, 147 (73.5 %) were C‐A, 38 (19 %) incomplete, and 15 (7.5 %) mixed; none were incorrect. Reviewers assigned the identical grade to 67 of 100 answers (67 %), yielding κ = 0.21 (fair agreement). Unanimous judgements comprised 57 C‐A, 7 incomplete, and 3 mixed answers. The two GPT‐4o answers to a question differed materially in 37 of 50 cases (74 %), with added detail rather than contradictions accounting for divergence. Mean FKGL was 13.1 (FRE 36.5); only 4 % of answers met the ≤ 8^t^ʰ‐grade benchmark for patient education.

**Conclusion**: GPT‐4o is a safe and time‐saving starting point for obstetric FAQs: it produced zero outright errors and was rated *comprehensive & accurate* in nearly three‐quarters of clinician reviews. Yet meaningful gaps remain—one answer in three fails to satisfy both specialists, and 96 % of responses exceed an 8^t^ʰ‐grade reading level. The most effective workflow is therefore “clinician‐in‐the‐loop”: generate two iterations, merge their complementary content, embed institution‐specific guidance and red‐flag warnings, then translate the text to patient‐friendly language before dissemination. Deployed this way, GPT‐4o can standardize counselling scripts, shorten drafting time, and let MFM providers focus on nuanced care rather than repetitive education, while periodic re‐benchmarking keeps pace with evolving LLM capabilities and clinical guidelines.

**3:30 PM – 5:00 PM**

## 1039 | **Association Between the Time‐Interval of Conception After Methotrexate and the Following Pregnancy Outcomes**

Bar Rosh^1^; Joul Hadad^2^; Naomi Gronich^2^

^1^Rambam Health Care Campus, Haifa, HaZafon; ^2^Carmel Medical Center, Haifa, Hefa

**Objective**: Methotrexate is commonly used to treat ectopic pregnancy, but its teratogenicity raises concerns about the optimal time interval needed until  a following conception. Guidelines recommend a 3‐months washout period, while the manufacturer advises 6 months. Evidence on the safety of early conception is limited. In this study we aimed to assess if a 1‐month washout period following methotrexate treatment for ectopic pregnancy, is as safe as a longer interval.

**Study Design**: We conducted a retrospective cohort study of women treated with 30–150 mg methotrexate for ectopic pregnancy in Clalit Health Services hospitals (2010–2021). The following pregnancies were categorized based on the time interval between methotrexate treatment and conception. Outcomes of the following pregnancies included major congenital malformations, spontaneous abortions, preterm birth, and low birth weight. Adjusted odds ratios (ORs) were estimated using generalized estimating equations.

**Results**: The study included 711 cases. Conception occurred within 1–3 months in 38 cases, 3–6 months in 111, and >6 months in 562. Major congenital malformations were diagnosed in 71 infants, with no significant differences between groups. Although numerically higher rates of congenital malformations associated with methotrexate embryopathy, preterm birth, and low birth weight were observed in the 1–3 months group, these differences were not statistically significant (Figure 1). Adjusted ORs for congenital malformations were 0.97 (95% CI, 0.28–3.42) for 1–3 months and 1.04 (95% CI, 0.48–2.24) for 3–6 months, compared to >6 months (Table 1).

**Conclusion**: Conception 1–3 and 3–6 months after methotrexate treatment for ectopic pregnancy does not appear to increase the risk of major fetal malformations. Conception within 1–3 months may warrant further study in larger cohorts.

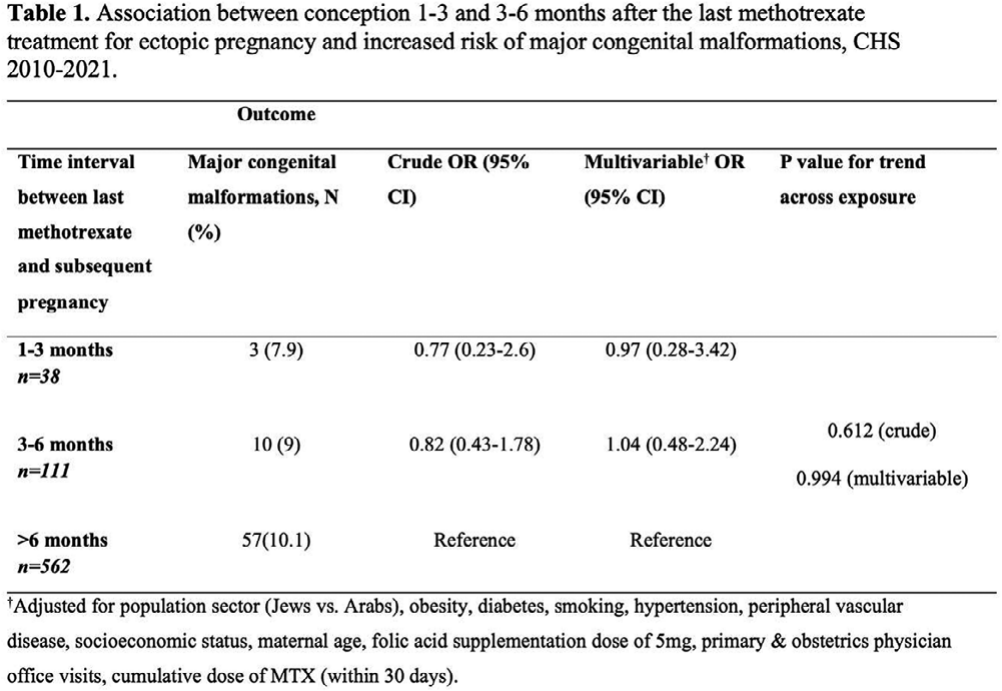

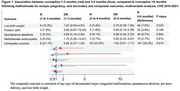

**3:30 PM – 5:00 PM**

## 1040 | **Optimizing Anatomic Site Selection for Needle Decompression and Chest Tube Placement for Pneumothorax in Pregnancy**

Beverly C. Tse^1^; Tonya Tucker^1^; Ava G. Holland^1^; Benjamin Sutton^2^; Caroline J. Berberian^3^; Poorna Balakumar^3^; Kasey McKenna^3^; Andrea D. Shields^1^

1University of Connecticut Health, Farmington, CT; ^2^Centre for Emergency Health Sciences, Spring Branch, TX; ^3^University of Connecticut School of Medicine, Farmington, CT

**Objective**: Tension pneumothorax can cause life‐threatening respiratory and cardiac complications. Prompt decompression is essential, with current guidelines favoring the 4th or 5th intercostal space (ICS) at the anterior axillary line (AAL) over the traditional 2nd ICS at the midclavicular line (MCL).  In pregnancy, physiologic changes such as diaphragm elevation and altered thoracic anatomy may influence the safety and effectiveness of these sites.  The primary objective of this study is to determine the optimal location for needle decompression and chest tube placement in pregnancy utilizing chest x‐ray (CXR) and computed tomography (CT) imaging.

**Study Design**: Imaging from pregnant patients with singleton pregnancies between 6–41 weeks were retrospectively analyzed. CXRs were used to measure diaphragm position relative to the anterior ribs and ICS at the MCL. CT scans were used to assess diaphragm position at the AAL, chest wall thickness (CWT), and distance to vital structures (DVS) at the 2nd ICS (MCL) and the 4th and 5th ICS (AAL).  ANOVA was used to assess differences across sites and trimesters.

**Results**: A total of 91 CXRs and 56 CT scans were analyzed.  The diaphragm was located between the 4th–8th ribs at the MCL and the 5th–8th ribs at the AAL, with no statistical difference when stratifying by trimester (Table 1).  Mean CWT was greatest at the 2nd ICS (MCL) and thinnest at the 5th ICS (AAL) with a statistically significant difference across sites (*p* < 0.001).  The shortest mean DVS was at the left 5th ICS (AAL) (Table 2).  On the right, the liver was in the needle path in 31% of cases at the 5th ICS, while at the 4th ICS both abdominal structures and the diaphragm were not encountered.

**Conclusion**: Needle decompression site in pregnancy requires balancing safety and procedural success. The 2nd ICS (MCL) offers the greatest DVS but may fail due to increased CWT. The 4th and 5th ICS (AAL) offer thinner chest walls but pose greater risk of organ injury, particularly on the left.  Due to the thinner chest wall and lack of abdominal structures, the 4th ICS (AAL) may be the optimal decompression site in pregnancy.

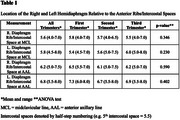

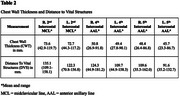

**3:30 PM – 5:00 PM**

## 1041 | **Compliance and Side Effects of Iron vs. Placebo for Non‐Anemic Iron Deficiency in Pregnancy**

Bijal Parikh^1^; Mayi Gnofam^2^; Rakasa Pattanaik^3^; Brynn S. Franz^4^; Tiffany Yang^5^; Emily Stetler^2^; Chaitali Korgaonkar‐cherala^6^; Heidi Preis^5^; David J. Garry^7^; Cassandra Heiselman^8^; Kimberly Herrera^8^

1St. Peters University Hospital, Warren, NJ; ^2^Stony Brook University, New York, NY; ^3^Beth Israel Deaconess Medical Center, Boston, MA; ^4^University of South Carolina School of Medicine–Columbia, SC, East Northport, NY; ^5^Stony Brook University, Stony Brook, NY; ^6^Stony Brook Medicine, Stony Brook, NY; ^7^Stony Brook Medicine, Stony Brook Medicine, NY; ^8^Stony Brook University Hospital, Stony Brook, NY

**Objective**: Non‐anemic iron deficiency (NAID) in pregnancy is common and a known risk factor for the development of anemia. As part of a study to examine the effects of iron supplementation on anemia rates, we evaluated the compliance and side effect profile of iron supplementation compared to placebo in pregnant individuals with NAID.

**Study Design**: This was a double‐blinded, randomized controlled trial at a single academic center from 08/2022 to 03/2024. Patients ≤20 weeks gestation with NAID, defined as low ferritin (<30 mcg/L) and normal hemoglobin (  >11g/dL) were included. Participants were randomized to receive an iron supplement or placebo pill daily and prenatal care providers were blinded to the treatment arm. Outcomes assessed compliance and side effects in the second and third trimester, based on two telephone surveys. Intention‐to‐treat analysis included chi‐square tests, and t‐tests or nonparametric equivalents.

**Results**: There were 132 patients with NAID included in the study with 64 in the treatment group and 68 in the control group. At the second trimester survey, there was no significant difference between treatment and control group regarding compliance (*n* = 39, 83.0% VS *n* = 47, 88.7%, *p* = 0.41), observance (mean = 6.4, *SD* = 1.3 vs mean = 6.5, *SD* = 1.1, *p* = 0.74) and attrition (*n* = 8, 12.5% VS *n* = 7, 10.3%, *p* = 0.72). At the third trimester survey, there were no significant group difference in compliance (*n* = 26, 61.9% VS *n* = 33, 80.5%, *p* = 0.06), observance (mean = 6.2, *SD* = 1.5 vs mean = 6.2, *SD* = 1.2, *p* = 0.93) and attrition (*n* = 8, 12.5% VS *n* = 9, 13.2%, *p* = 0.91). Participants in both groups reported similar side effects at the second and third trimester (Tables 1 and 2) except for significantly higher rates of third trimester vomiting in the treatment group compared to the placebo group (*n* = 5, 14.3% VS *n* = 0, 0%; *p* = 0.02).

**Conclusion**: Iron supplementation was generally well tolerated and had similar compliance and attrition rates compared to placebo during the second and third trimesters. Side effects were not significantly different between groups, except for a higher incidence of third trimester vomiting in the iron group.

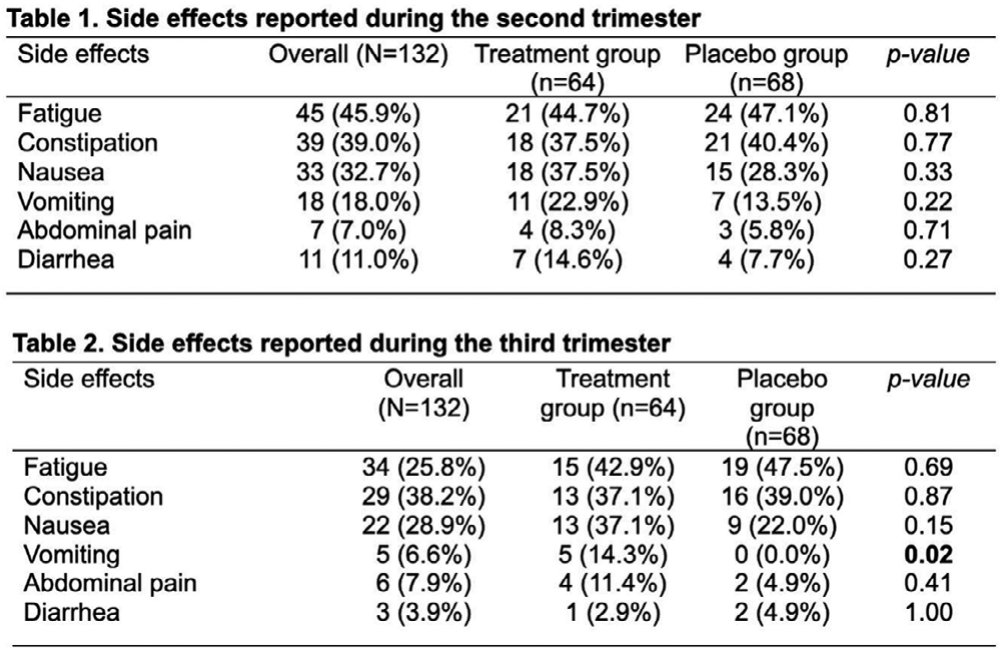

**3:30 PM – 5:00 PM**

## 1042 | **Maternal and Neonatal Outcomes Following Fetoscopic Versus Open Prenatal Surgery for Open Neural Tube Defects**

Bree A. Goodman^1^; Jagruti Anadkat^2^; Jennifer M. Strahle^1^; Jesse Vrecenak^2^; Sarah Smith^3^; Jeannie C. Kelly^3^; Nandini Raghuraman^1^; Katherine H. Bligard^2^

1Washington University School of Medicine, St. Louis, MO; ^2^Washington University School of Medicine, Saint Louis, MO; ^3^Washington University School of Medicine, Washington University School of Medicine, MO

**Objective**: Fetoscopic repair of open neural tube defects (ONTDs) is a promising alternative to open prenatal surgery via hysterotomy that may confer similar fetal benefits while allowing for vaginal delivery (VD) in the index and subsequent pregnancies. We sought to evaluate maternal and neonatal outcomes following fetoscopic versus open prenatal surgery for ONTDs.

**Study Design**: This was a retrospective cohort study of all patients who underwent prenatal ONTD repair at a single fetal center from 2020 to 2024. Fetoscopic repair with a multi‐layer closure identical to those done open via hysterotomy was offered to all patients starting in 2023.  Demographic information, ultrasound findings, and maternal and neonatal outcomes were collected and compared for open and fetoscopic cases. Primary outcomes were rate of severe maternal morbidity (SMM) as defined by the CDC and need for CSF diversion in the first year of life. Secondary outcomes were PPROM, VD rate, length of surgery for patients undergoing cesarean delivery (CD), postpartum length of stay (LOS), live birth, preterm birth, postnatal imaging findings and NICU LOS.

**Results**: Of 36 prenatal repairs, open surgery was performed in 24 (66.7%) and fetoscopic surgery was performed in 12 (33.3%). Pre‐surgical characteristics were similar between groups, with the exception of a higher rate of myeloschisis in the fetoscopic repair group (Table 1). With regards to maternal outcomes, fetoscopic repair was associated with lower rate of SMM (0.0% vs. 33.3%, *p* = 0.03), lower delivery EBL (200mL vs. 800mL, *p* < 0.001), and a shorter postpartum stay (2.2 days vs. 3.7 days (*p* < 0.001) which is likely related to a higher rate of vaginal birth in the fetoscopic repair group (66.7% vs. 0.0%, *p* < 0.001). Neonatal outcomes including preterm birth, NICU length of stay, postnatal hydrocephalus, and need for CSF diversion were not statistically different.

**Conclusion**: Fetoscopic prenatal repair of ONTD is associated with lower rates of SMM, lower EBL at delivery, and shorter postpartum stay when compared to open repair, but appears to confer similar fetal neurological benefit.

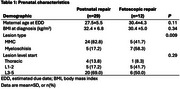

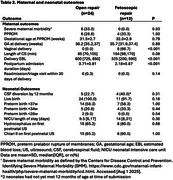

**3:30 PM – 5:00 PM**

## 1043 | **Maternal Vulnerability Index As a Predictor of Severe Maternal Morbidity in Louisiana**

Brian W. James^1^; Alexis Cates^2^; Talia Suner^3^; James D. Toppin^3^; Mariella Gastanaduy^4^; Frank B. Williams^2^

1Ochsner Health, New Orleans, LA; ^2^Ochsner Clinic Foundation, Ochsner Clinic Foundation, LA; ^3^Ochsner Clinic Foundation, New Orleans, LA; ^4^Ochsner Center for Outcomes Research, New Orleans, LA

**Objective**: Neighborhood characteristics, captured in indices such as the Area Deprivation Index (ADI) that evaluate a range of census‐level socioeconomic (SES) characteristics, have been associated with various adverse health outcomes. The Maternal Vulnerability Index (MVI) is a novel index that incorporates access to reproductive healthcare with SES. We aim to evaluate MVI and ADI as predictors of Centers for Disease Control‐defined Severe Maternal Morbidity (SMM).

**Study Design**: This is a retrospective cohort study including those receiving prenatal and delivery care in a large Louisiana health system from March 2018 to April 2023. Home address at admission was linked to a census block, which were then linked to national centile for both MVI and ADI. The primary outcome was any recorded non‐transfusion SMM. Receiver operator characteristic (ROC) curves were generated to determine the predictive value of each index, both as continuous and binary predictors (analyzed as high vulnerability/deprivation vs not with cutoffs at 75^th^ percentile).

**Results**: There were 61,901 deliveries identified, with 35% residing in high deprivation neighborhoods (table). There were 993 (1.6%) diagnosed with non‐transfusion SMM. Increasing ADI (OR 1.009, CI 1.006–1.012, *p* < 0.001) and MVI (OR 1.008, CI 1.005–1.007, *p* < 0.001) were both associated with the development of SMM, representing an increase of 0.8 to 0.9% in odds for SMM with each 1.0% increase in neighborhood deprivation. Living in a high MVI neighborhood was associated with 37% increased odds of SMM (OR 1.37, CI 1.21–1.56), compared to a 32% increase associated with living in a high ADI neighborhood (OR 1.32, CI 1.16–1.50). Neither AUC demonstrated strong discriminatory value, though MVI was statistically better than ADI (figure).

**Conclusion**: High MVI is associated with increased odds for non‐transfusion SMM, potentially informing neighborhood characteristics that can lead to maternal morbidity, including access to reproductive health care.

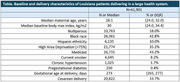

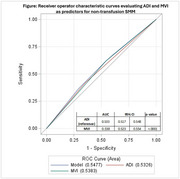

**3:30 PM – 5:00 PM**

## 1044 | **A Comprehensive Analysis of Maternal Health Policy in the State of Illinois**

Britney P. Smart^1^; Kelsey Barnick^2^; Lauren Keenan‐Devlin^3^; Marion Malcome^4^

1Sinai Urban Health Institute, CHICAGO, IL; ^2^Sinai Urban Health Institute, Chicago, IL; ^3^Endeavor Health, Evanston, IL; ^4^Loyola University Chicago, Chicago, IL

**Objective**: Policy has tremendous implications for maternal health (MH). The objective of this study was to understand the Illinois policy landscape related to MH and its alignment with policy recommendations from the March of Dimes (MOD) and the American College of Obstetrics and Gynecology (ACOG).

**Study Design**: Using the READ (Ready your materials, Extract data, Analyze data, and Distill your findings) framework, we conducted an analysis of all proposed and approved MH legislation in the state of Illinois over a 14‐year period (2010–2024).  Our team developed a search tool to identify maternal health laws using the Illinois General Assembly database. We used an iterative approach to remove duplicates, resolutions, and bills not pertinent to MH, resulting in an analytical sample that was entered into MaxQDA, coded, and reviewed for alignment or misalignment with 10 MOD and 28 ACOG maternal health policy recommendations, both of which can be found on their respective websites within their policy priorities.

**Results**: From 2010–2024, a total of 56 bills related to MH were introduced into the Illinois state legislature, and 23% (*n* = 13) were passed into law. The largest number of bills focused on maternal mortality, with 30 bills introduced and all 30 aligned with ACOG and MOD recommendations; 5 were passed into law. Abortion rights was the second most common MH topic of legislation, with 29 bills introduced, 5 bills that aligned with ACOG policy recommendations and 24 bills that were misaligned; no bills were passed into law.

**Conclusion**: In the state of Illinois, most MH legislation either focused on maternal mortality and aligned with MOD and AGOG recommendations, or focused on abortion rights and was misaligned with recommendations. Our analysis highlights the competing priorities in MH in the current political landscape and the need for ongoing advocacy for legislation that meets evidence‐based recommendations.

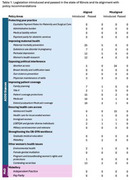

**3:30 PM – 5:00 PM**

## 1045 | **Development of an Updated Predictive Model for VBAC Success**

Bryce T. Munter^1^; Karla M. Martin^2^; Irene A. Stafford^3^

^1^Division of Maternal‐Fetal Medicine, Department of Obstetrics, Gynecology, and Reproductive Sciences, McGovern Medical School at UT Health Houston, Houston, TX; ^2^Department of Obstetrics, Gynecology and Reproductive Sciences, McGovern Medical School at UTHealth Houston, Houston, TX; ^3^Division of Maternal‐Fetal Medicine, Department of Obstetrics, Gynecology and Reproductive Sciences, McGovern Medical School at UTHealth Houston, Houston, TX

**Objective**: The commonly used vaginal birth after cesarean (VBAC) calculator does not account for the specific subtype of labor dystocia that resulted in prior cesarean delivery (CD) or include patients with multiple prior CD. To address the impact of these factors on VBAC success rates, we aimed to develop a novel predictive model incorporating these previously unevaluated variables and compare them to the current VBAC calculator.

**Study Design**: We performed a retrospective case‐control study of all women who attempted trial of labor after cesarean (TOLAC) and delivered at a large county hospital system in Houston, TX from 1/2019–1/2022. Data were collected using ICD‐10 codes and detailed chart review. Variables included maternal comorbidities, obstetric history, detailed reason for prior CD, and maternal and neonatal outcomes. A multivariate logistic ridge regression model using stepwise backward elimination identified predictors of successful VBAC with variables significant at *p* < 0.05. Results were expressed as odds ratios (OR) with 95% confidence intervals (CI) and compared to the OR used in the current MFMU VBAC calculator.

**Results**: During the study period, 3036 patients with prior cesarean delivered, of which 1160 (38%) attempted TOLAC. Among these, 393 (34%) ultimately underwent repeat CD, while 767 (66%) had successful VBAC. History of two prior CD did not decrease VBAC success odds compared to one prior CD (OR 1.1 (CI 0.66–1.75). Compared to patients who had initial CD for reasons other than labor dystocia, patients who had initial CD for active phase arrest had lowest chances of VBAC success (OR 0.02, 95% CI 0.02–0.03), followed by prior CD for failed induction (<6cm dilation, OR 0.03, 95% CI 0.02–0.03), then prior CD for arrest of descent (OR 0.1 (95% CI 0.04–0.18). Twenty patients had uterine rupture (1.7%).

**Conclusion**: These results indicate that prior labor dystocia details should be considered in VBAC prediction calculators.

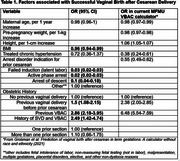

**3:30 PM – 5:00 PM**

## 1046 | **Prediction of Severe Preeclampsia Antepartum: Machine Learning Algorithms Including Social Vulnerability Data**

Carly I. Hirschberg^1^; Rachel P. Gerber^2^; Dana Clark^2^; Edgardo Molina^2^; Roberto Martin^2^; Matthew Weisberg^2^; Marek Sirendi^2^; Ipsita Chatterjee^2^; Ujwala Ravinder^2^; Matthew J. Blitz^2^; Fernando Suarez^2^; Edwidge Thomas^2^; Adriann Combs^2^; Michael Nimaroff^2^; Burton Rochelson^2^

1Montefiore Medical Center, Bronx, NY; ^2^Northwell, New Hyde Park, NY

**Objective**: To optimize machine learning models created based on large‐scale clinical and social vulnerability data to improve prediction of severe preeclampsia in the antepartum period. Additionally, we sought to determine which factors were most influential in determining risk.

**Study Design**: Multi‐center analysis of patients who received prenatal care from January 2018 to July 2025 at Northwell Health throughout the New York metropolitan area. Data collected prior to twenty weeks of pregnancy was used for the prediction models and was sourced from the electronic health record system. Three statistical learning algorithms to predict severe preeclampsia were created: (1) weighted gradient‐boosted tree model using XGBoost, (2) stacking ensemble including above model, a random forest and a regularized logistic‐regression and (3) a logistic regression baseline. Variables considered in the model include maternal characteristics, obstetric and medical history, and social vulnerability data.

**Results**: A total of 156,693 patients were used to train the algorithm, of whom 2.54% were diagnosed with severe preeclampsia during their pregnancies. The algorithms were tested on 17,411 patients. The algorithm thresholds were optimized to F1‐scores to balance precision and recall with an F1‐score of 0.105–0.187. The prediction model yielded an area under the curve of 0.631–0.749, a sensitivity of 15–24%, specificity of 92–98%, and false‐positive rate of 1.5–7.4%.  The factors most predictive of preeclampsia, which differed by model, included prior history of preeclampsia, past medical history of hypertension, and early diagnosis of diabetes in pregnancy (prior to 20 weeks).

**Conclusion**: Machine learning algorithms yielded high predictive performance for severe preeclampsia risk from data routinely collected early in pregnancy. Our model has predictive accuracy above that which is currently utilized. Additionally, specifically predicting the severe subtype of antepartum preeclampsia can assist in improved and personalized counseling at the first prenatal visit, without additional workup.

**3:30 PM – 5:00 PM**

## 1047 | **Neonatal Outcomes Among Pregnancies Complicated by Vasa Previa: A California Population‐Based Cohort Study**

Carolyn C. Green^1^; Amelia H. Gagliuso^2^; Bharti Garg^3^; Aaron B. Caughey^3^

1Oregon Health & Science University, Portland, OR; ^2^Oregon Health and Science University, Portland, OR; ^3^Oregon Health & Science University, Oregon Health & Science University, OR

**Objective**: Vasa previa is a rare condition, affecting 1/2500 pregnancies. However, it is a diagnosis associated with severely adverse maternal and neonatal outcomes. Due to this high concern for morbidity, practices around the ideal timing of delivery for vasa previa vary. The objective of this study is to examine the association between key neonatal outcomes by gestational age at the time of delivery for a contemporary cohort.

**Study Design**: This is a retrospective cohort study using California's linked vital statistics‐hospital discharge data (2008 and 2020). We included singleton births with gestational age of 32–36 weeks, among individuals with a diagnosis of vasa previa who were hospitalized before delivery. Vasa previa was identified using ICD‐9 and ICD‐10 diagnostic codes. Maternal and neonatal outcomes were compared between two consecutive weeks of gestational age using chi‐square tests and multivariate Poisson regression.

**Results**: A total of 760 cases met inclusion criteria. Rates of adverse neonatal outcomes were observed to decline with increasing gestational age. For example, the rate of RDS decreased from 61.9% at 32 weeks to 7.1% at 36 weeks. Rates of Apgar score <7 declined from 4.9% at 33 weeks to 1.9% at 35 weeks. In regression models, delivery at 35 vs. 34 weeks was associated with lower risk of RDS (aRR 0.42, 95% CI 0.30–0.59) and jaundice (aRR 0.70, 95% CI 0.60–0.81). Delivery at 36 vs. 35 weeks did not show a statistically significant difference for any of the outcomes.

**Conclusion**: Among patients hospitalized due to vasa previa before delivery, neonatal outcomes improved incrementally as gestational age advanced from 32 to 35 weeks, though no significant benefits were noted with delivery at 36 vs 35 weeks. These findings highlight the importance of balancing risks of prematurity with potential neonatal morbidity when determining optimal age at delivery.

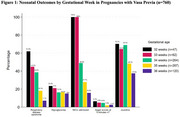

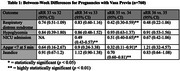

**3:30 PM – 5:00 PM**

## 1048 | **Perinatal Outcomes in Individuals With Classic Versus Mosaic Turner Syndrome: A Retrospective Cohort Study**

Casey Lower^1^; Vincent Tang^1^; Joanne N. Quiñones Rivera^1^; Srijit Paul^2^; Zhe Chen^1^; Meredith Rochon^1^

1Lehigh Valley Health Network, Allentown, PA; ^2^University of South Florida, University of South Florida, FL

**Objective**: Pregnancy in individuals with Turner syndrome (TS) is associated with an increased risk of adverse pregnancy and maternal outcomes, but most available data are from small case series that do not differentiate between classic and mosaic types. As mosaic TS may be associated with a less severe phenotype, we sought to compare maternal and perinatal outcomes for individuals with classic versus mosaic TS in a large dataset.

**Study Design**: This retrospective cohort study used Epic Cosmos, a large, de‐identified, national clinical dataset, currently with 300 million patients. Pregnancies with maternal TS identified by ICD‐10 codes and delivery outcomes were included. Maternal demographic characteristics and perinatal outcomes within 30 days of delivery were compared for those with classic vs mosaic TS.

**Results**: 2724 were identified: 1875 with classic TS and 849 with mosaic TS (Table). Those with mosaic TS were older (31.9 vs 30.5y, *p* < 0.001), more likely to be White (75.9 vs 69.3%, *p* < 0.001), had a higher prior cesarean delivery rate (29.7 vs 25.5%, *p* = 0.044), and a higher risk of maternal congenital heart disease (2.9 vs 1.3%, *p* = 0.004). Those with classic TS had a higher number of prior live births (1.04 vs 0.86, *p* < 0.001) but were also more likely to have had a prior fetal demise (10.2 vs 2.9%, *p* < 0.001). Regarding delivery outcomes, cesarean delivery rate was higher (44.6 vs 38.5%, *p* = 0.005) and birthweight was lower (3088 vs 3159g, *p* = 0.02) in the mosaic TS group; delivery outcomes were otherwise similar. Postpartum aortic dissection was rare in both groups but occurred more frequently in the mosaic TS group (0.59 vs 0.11%, *p* = 0.034). There were no maternal deaths in either group.

**Conclusion**: In this large cohort of deliveries complicated by maternal TS, pregnancy outcomes for individuals with classic TS and mosaic TS were overall favorable, although there were some phenotypic and outcome differences between groups. Given limitations in the dataset–including possible inaccurate TS classification and incomplete data capture (including conception method)–these findings should be interpreted with caution.

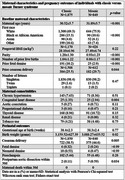

**3:30 PM – 5:00 PM**

## 1049 | **Effect of HIV Status on Cord Blood SARS CoV‐2 Seropositivity Among Pregnant Women in Zambia**

Cassandra R. Duffy^1^; Ethan Zulu^2^; Julie Herlihy^3^; Christopher J. Gill^4^; Lawrence Mwananyanda^2^; Blair J. Wylie^1^; Don Thea^4^

1Beth Israel Deaconess Medical Center, Boston, MA; ^2^Right to Care Zambia, Lusaka, Lusaka; ^3^Boston University Chobanian & Avedisian School of Medicine, Boston Medical Center, Boston, MA; ^4^Boston University School of Public Health, Boston, MA

**Objective**: Both pregnancy and HIV are known risk factors for severe COVID‐19 disease, yet data from Africa, where the majority of women living with HIV reside, are lacking. We examined whether HIV affected the incidence of SARS CoV‐2 cord blood antibodies during the first years of the pandemic in Zambia.

**Study Design**: We leveraged a prospective observational cohort of pregnant women living with and without HIV and receiving care at an urban clinic in Lusaka, Zambia. Of those with HIV, most were on CART preconception and had CD4 counts >350 cell/mm3; about half had an undetectable viral load. Banked cord blood specimens were tested for SARS CoV‐2 antibodies to spike and nucleocapsid proteins (Euroimmune ELISA, Lubeck, Germany); those positive for both were presumed to have had a maternal COVID‐19 infection. We examined the association of HIV with evidence of COVID infection in two groups: deliveries occurring during the first year of the pandemic and deliveries after vaccine introduction until study completion in November 2022.

**Results**: From April 2020 to March 2021, 317 umbilical cord blood samples (186 HIV+/131 HIV‐) were tested for SARS CoV‐2 antibodies. 28 (8.8%) showed evidence of prior COVID‐19 infection; there was no difference by HIV status (9.1 v 8.4% *p* = 0.82). Following introduction of the COVID vaccine, 618 cord blood samples (318 HIV+/300 HIV‐) were tested. Overall, 48% showed evidence of infection and 25% vaccination without infection. During this period, cord blood from women living with HIV was less likely to show evidence of infection (41.6 v 54.1%, *p* < 0.01) and more likely to show evidence of active immunity (29.8 v 19.1%, *p* < 0.01).

**Conclusion**: In a pregnant cohort with mostly well‐controlled HIV infection in Zambia, HIV status did not affect the risk of COVID infection during the first year of the pandemic. SARS CoV‐2 seropositivity rose dramatically after the first year, and pregnant women living with HIV were less likely to have evidence of prior COVID infection, possibly related to higher rates of vaccination.

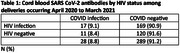

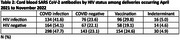

**3:30 PM – 5:00 PM**

## 1050 | **Association Between Severe Maternal Morbidity and Racism in Electronic Medical Records: A Case‐Control Mixed‐Methods Study**

Chaarushi Ahuja^1^; Kherah Rawlins^2^; Sakura Oyama^1^; Kathryn Callahan^1^; Katherine H. Campbell^3^; Olga Grechukhina^3^; Audrey Merriam^1^

1Yale School of Medicine, Yale School of Medicine, CT; ^2^Yale New Haven Health, Yale New Haven Health, CT; ^3^Division of Maternal Fetal Medicine, Yale School of Medicine, New Haven, CT

**Objective**: There are 4 forms of contemporary racism (interpersonal, internalized, institutional, and structural) in healthcare that contribute to racial inequities. Little is known about whether these forms of racism are notable in the electronic medical record (EMR). We aimed to identify evidence of racism in the EMR and determine if there was an association with severe maternal morbidity (SMM).

**Study Design**: A case control study of pregnant patients with SMM was performed in our hospital system from 2018–23. SMM cases were matched 1:1 to controls by delivery date, gestational age(GA), age, race, ethnicity, insurance status, and mode of delivery. A data dictionary containing examples was created with guidance of 2 maternal fetal medicine subspecialists. Chart review and qualitative content analysis of pregnancy records for SMM and controls was performed by 3 independent reviewers. Presence of racism within each form was determined. Clinical and sociodemographic characteristics were compared across the two groups using chi‐square or ANOVA as appropriate. Odds ratios were calculated using JMP.

**Results**: 198 cases of SMM and 198 controls were identified. Differences among the groups were noted in hypertensive disorders of pregnancy (HDP), pregestational diabetes (DM), compliance with treatment plan, and food/housing insecurity (Table 1). The odds of having evidence of racism in the EMR were higher (1.96; CI 1.3–3.0) in the SMM group compared to controls (Table 2). The relationship remained significant after adjusting for HDP, DM, gestational diabetes, chronic hypertension, age, race and GA. Among the forms of racism, odds of evidence of structural and institutional racism in the EMR were higher in the SMM group (Table 2).

**Conclusion**: This is one of the first studies to demonstrate that evidence of racism in the EMR is significantly associated with SMM. Our findings also suggest a stronger association with structural racism and SMM compared to interpersonal interactions. Further studies are needed to explore qualitative themes within these forms of racism to inform effective interventions that could decrease SMM.

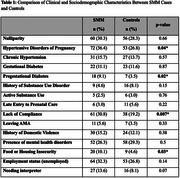

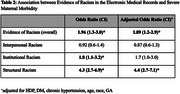

**3:30 PM – 5:00 PM**

## 1051 | **Primary Immunodeficiency Drives Severe Maternal Morbidity: A Nationally Representative Cohort Study**

Charlotte Laurel^1^; Chandler Bradley^2^; Grace D. Bosma^3^; Mary D. Sammel^4^; Jessica Galant‐Swafford^5^; Manesha Putra^2^

1University of Colorado Anschutz School of Medicine, Denver, CO; ^2^University of Colorado, Aurora, CO; ^3^University of Colorado Anschutz Medical Campus, Aurora, CO; ^4^Colorado School of Public Health, Aurora, CO; ^5^National Jewish Health, Denver, CO

**Objective**: To quantify the risk of severe maternal morbidity (SMM) associated with primary immunodeficiency (PI) during delivery hospitalizations in a nationally representative cohort. Although rare, PI includes a spectrum of genetic inherited immune disorders that may increase vulnerability to infection, autoimmunity, organ failure, and other life‐threatening peripartum complications.

**Study Design**: We conducted a retrospective cohort study using data from the National Inpatient Sample (2016–2022), a database capturing approximately 20% of hospital discharges in the United States. Delivery admissions, comorbidities, and SMM were identified through previously established ICD‐10 procedure and diagnosis. PI diagnoses were identified using ICD‐10 codes obtained from the American Academy of Allergy, Asthma & Immunology. Bivariate analyses were used to compare the prevalence of comorbidities and SMM indices between PI and non‐PI groups. Inclusion criteria for the PI group includes patients hospitalized for childbirth between 2016 and 2022 and an ICD‐10 code of PI. Exclusion criteria include missing key demographic or outcome variables needed for analysis. A multivariable binary logistic regression was used to assess the relationship of PI on SMM while controlling for the number of CI with diagnosis of PI as the primary exposure and SMM as the outcome of interest.

**Results**: A total of 5,027,079 delivery hospitalizations were identified. Diagnosis of PI was found in 1052 admissions. Several comorbidities are more common among patients with PI (Figure 1a).  SMM occurred in 10.4% of patients with PI versus 1.9% without, mostly driven by immune‐related SMM (Figure 1b).  In the logistic regression model, PI was associated with SMM even after controlling for the number of CI (OR 3.02, 95% CI 2.43–3.73, *p* < 0.001).

**Conclusion**: Patients with PI had a threefold increased odds of severe maternal morbidity even after controlling for comorbidities. These findings show a critical gap in perinatal risk stratification and underscore the need for early recognition and targeted care in this overlooked population.

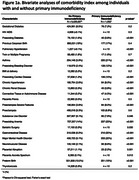

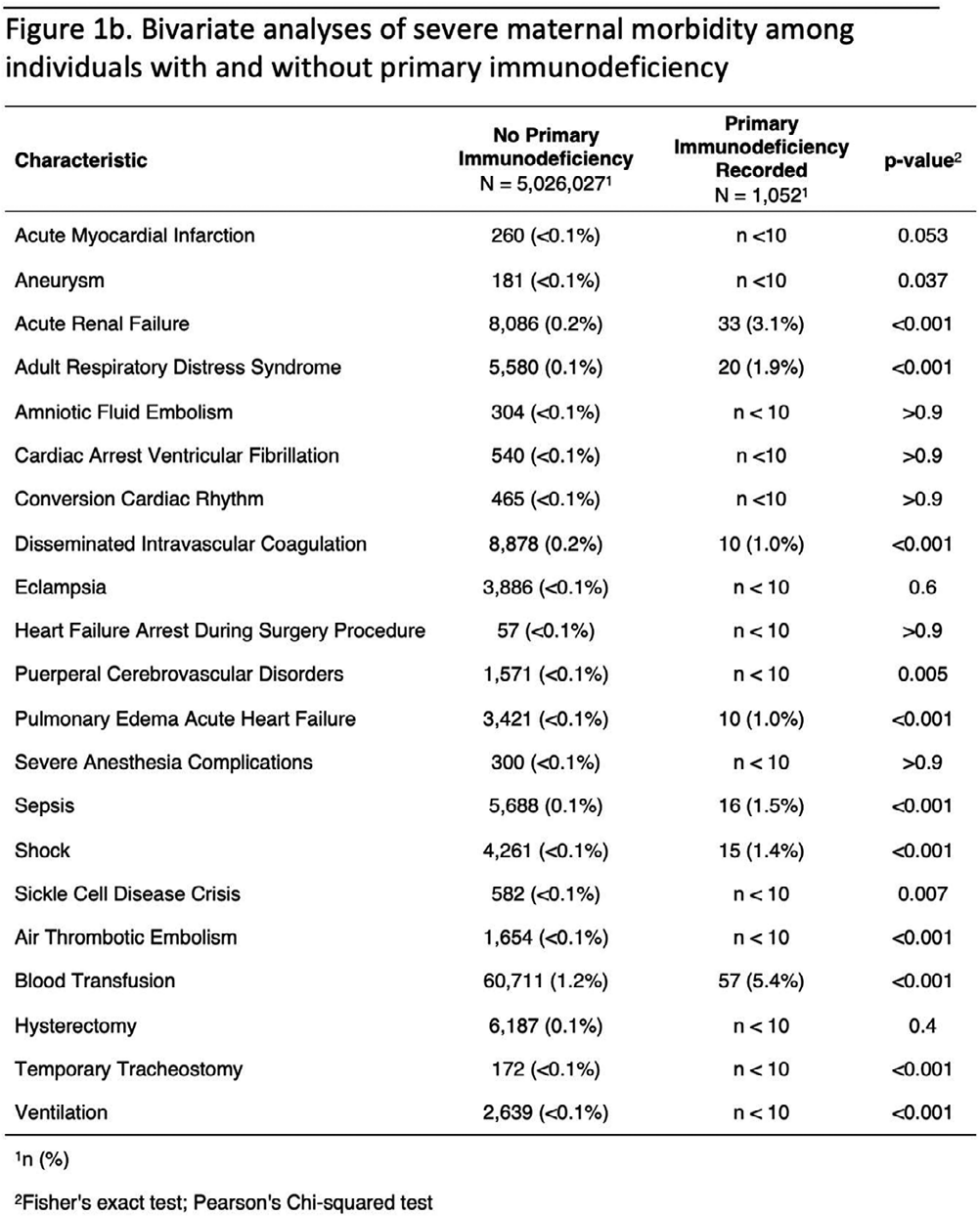

**3:30 PM – 5:00 PM**

## 1052 | **Effect of Spinal Anesthesia on the Fetal Autonomic Nervous System: An Experimental Study**

Sebastien Insubri; Louis Galan; Elise Deroubaix; Geoffroy Chevalier; Julien De jonckheere; Louise Ghesquiere; Charles Garabedian

CHU Lille, Lille, Nord‐Pas‐de‐Calais

**Objective**: Spinal anesthesia is the reference technique for scheduled cesarean deliveries. Its main risk is maternal hypotension, which may impact fetal blood gas parameters. Thus our objective was to assess the effects of spinal anesthesia on the fetal autonomic nervous system (ANS).

**Study Design**: An experimental study was conducted in pregnant ewes. Prior surgical instrumentation allowed placement of fetal arterial catheters, thoracic electrodes and cerebral electrodes to record the electroencephalogram (EEG). Spinal anesthesia was performed on day 4. Two groups were compared: intrathecal bupivacaine vs isotonic saline solution (SSI, control). Maternal and fetal hemodynamic and blood gas parameters, along with ANS and EEG data, were collected before and after spinal injection. ANS activity was assessed via fetal heart rate variability, including the Fetal Stress Index (FSI), a quantitative marker of parasympathetic tone. Brain activity was analysed both qualitatively and using quantitative markers, including Spectral Edge Frequency (SEF), defined as the frequency below which 95% of the total spectral power is concentrated.

**Results**: Fourteen spinal anesthesia were performed (8 bupivacaine, 6 SSI). Maternal hypotension occurred in 62.5% of the bupivacaine group vs none in the SSI group. Maternal systolic blood pressure was lower at 3 min (96 vs 132 mmHg, *p* = 0.004) and 5 min (89 vs 117 mmHg, *p* = 0.045). Fetal mean arterial pressure decreased at 3 min (47 vs 59 mmHg, *p* = 0.014) and 5 min (49 vs 58 mmHg, *p* = 0.016). No fetal acidosis was observed. In the bupivacaine group, a global increase in fetal ANS activity was found, including a significant rise in FSI at 3 min vs baseline (77.8 vs 55.4, *p* = 0.046), with no change in the SSI group. SEF was significantly reduced in the bupivacaine group at 10 min (6.4 vs 13.9 Hz, *p* < 0.01).

**Conclusion**: This model of spinal anesthesia in pregnant ewes induced maternal hypotension and fetal changes in hemodynamics, blood gases, ANS, and EEG activity, suggesting an adaptive response to anesthetic stress.

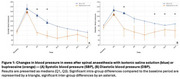

**3:30 PM – 5:00 PM**

## 1053 | **Survey of Vasa Previa Practice Patterns in the United States**

Chiara M. Corbetta‐Rastelli^1^; Asia Gandy^2^; Rachel Mundaden^3^; Molly Zeme^3^; Juan Gonzalez‐Velez^4^; Kate Swanson^2^

1UCSF, UCSF, CA; ^2^University of California, San Francisco, San Francisco, CA; ^3^UCSF, San Francisco, CA; ^4^University of California, San Francisco, Department of Obstetrics, Gynecology & Reproductive Sciences, San Francisco, CA

**Objective**: While SMFM suggests utilizing a cut off of 2cm to define vasa previa, a recent expert opinion suggested expanding vasa previa to include any fetal vessels within 5cm of the internal cervical os. Our objective was to explore maternal fetal medicine (MFM) clinician opinions and practices in light of this lack of consensus.

**Study Design**: We conducted a cross‐sectional nationwide survey of maternal fetal medicine clinicians (attendings and fellows) between June 2024 to June 2025. We distributed this survey via email listservs and the SMFM website, and used descriptive statistics for analysis.

**Results**: 182 clinicians responded to the survey. 63% of respondents were MFM fellows. Most respondents practiced in academic (76%) and urban (79%) settings. All US regions were equally represented. Over half of clinicians had a vasa previa volume of 1–2 cases per month. Most respondents (66%) define vasa previa as fetal vessels within 2cm from the cervical os, but approximately one quarter define vasa previa as vessels within 5cm (Figure 1). Management practices regarding hospital admission, antenatal surveillance, administration of betamethasone, and timing and route of delivery varied significantly (Table 1). When asked about a hypothetical case of fetal vessels overlying the cervical os in a patient with no risk factors for spontaneous preterm birth, most respondents recommend outpatient monitoring, ultrasounds every 4 weeks to monitor for vasa previa resolution, pelvic rest, and cesarean delivery at 35–36 weeks. When asked about a hypothetical case with fetal vessels 2–5 cm from the cervical os in a patient with no risk factors for spontaneous preterm birth, most recommend outpatient monitoring, ultrasounds every 4 weeks to monitor fetal vessels and no activity restriction. For this scenario, half of respondents recommend cesarean delivery at 36–37 weeks, while the other half recommend vaginal delivery and timing of delivery based on other obstetric indications.

**Conclusion**: There is a lack of consensus regarding definition and management of vasa previa among MFM clinicians in the United States.

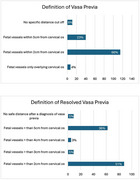

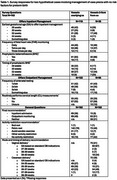

**3:30 PM – 5:00 PM**

## 1054 | **Effects of a Universal Postpartum Home Visit Program on Blood Pressure Monitoring**

Christopher L. Griffin^1^; Isabel K. Anderson^2^; Mariella Gastanaduy^3^; Meena Mishra^4^; Frank B. Williams^5^

1Ochsner Clinic Foundation, New Orleans, LA; ^2^University of Queensland ‐ Ochsner Clinical School, New Orleans, LA; ^3^Ochsner Center for Outcomes Research, New Orleans, LA; ^4^Ochsner Center of Outcomes & Health Services Research, New Orleans, LA; ^5^Ochsner Clinic Foundation, Ochsner Clinic Foundation, LA

**Objective**: Despite sustained risk for hypertension‐related complications, one‐third to half of newly delivered patients never attend a postpartum visit. We hypothesize that implementation of a universal nurse home visit model will improve rates and timeliness of postpartum blood pressure (BP) measurements.

**Study Design**: This was a retrospective cohort study of patients delivering at a single urban tertiary academic hospital in the South from 1/2024 to 1/2025. In July 2024, Family Connects (FC) was rolled out at our institution, offering delivering patients residing within city limits up to 3 home nurse visits during the postpartum period. To assess impact, we included all patients with city addresses listed at delivery admission; those delivering prior to 20 weeks gestation were excluded. July 2024 was considered a washout period and excluded from analysis. Baseline, pregnancy, and delivery characteristics were extracted from the electronic medical record, as were clinically assessed BP following delivery discharge. Primary outcome was an interrupted time series (ITS) analysis of BP assessment within 49 and 84 days of delivery. Secondary analyses evaluated time to BP assessment generating a Kaplan‐Meier curve. Alpha was set at 0.05.

**Results**: A total of 641 eligible pre‐implementation and 703 post‐implementation deliveries were identified. Cohorts were similar in age, parity, body mass index, race, ethnicity, chronic hypertension, delivery mode, and pregnancy‐associated hypertension. ITS analyses at 49 and 84 days postpartum demonstrated no increase in proportion of patients with BP assessment (Figure 1, results not shown for 84 days). Median time to first postpartum BP decreased from 28 days pre‐implementation to 25 days post‐implementation (Figure 2).

**Conclusion**: Introduction of the FC model was not associated with increased proportion of postpartum patients receiving BP assessment by 49 days postpartum, though median time to initial clinical BP was shortened following implementation.

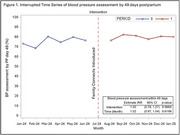

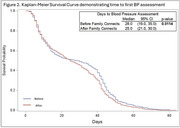

**3:30 PM – 5:00 PM**

## 1055 | **The Association of Neonatal Hypoglycemia With Childhood Obesity**

Claire E. Jensen

On behalf of the Eunice Kennedy Shriver National Institute of Child Health and Human Development (NICHD) Maternal‐Fetal Medicine Units (MFMU) Network, Bethesda, MD

University of North Carolina at Chapel Hill, Durham, NC

**Objective**: Neonatal hypoglycemia is common, associated with neonatal morbidity, and alters insulin, growth hormone, and cortisol secretion. However, its long‐term metabolic effects on offspring are understudied. We evaluated whether neonatal hypoglycemia was associated with childhood obesity.

**Study Design**: Secondary analysis of a prospective follow‐up study of children whose mothers enrolled in a trial comparing antenatal corticosteroids with placebo among pregnant women at risk for late preterm delivery. The trial did not include women with pregestational diabetes or multiple gestation. We included children ≥ 6 years old with hypoglycemia data after delivery and childhood anthropometry. The primary outcome was obesity at ages 6–11 (BMI‐for‐age‐and‐sex ≥ 95%). Secondary outcomes included additional anthropometry at follow‐up. Primary and secondary outcomes were compared between neonates with/without hypoglycemia and by persistence of hypoglycemia: 1 value <40 mg/dL (isolated) and ≥ 2 values <40 mg/dL (persistent). Additionally, we assessed for effect modification by breastfeeding status. Log‐binomial regression estimated relative risks and 95% CI for categorical outcomes. General linear models estimated mean differences and 95% CI, adjusting for potential confounders.

**Results**: Of 1211 children included, 284 (23.5%) were exposed to neonatal hypoglycemia: 197 (69.3%) persistent and 87 (30.6%) isolated. 225 children (18.6%) were obese. Neonates with any hypoglycemia were more likely to be preterm, LGA, exposed to betamethasone, or delivered by cesarean, and their mothers were older and more likely to have private insurance, GDM, and identify as White (Table 1). There was no association between any hypoglycemia and obesity (aRR 1.02; 95% CI 0.77, 1.35) or persistent hypoglycemia and obesity (aRR 1.01; 95% CI 0.73,1.39) (Table 2). Breastfeeding was not an effect modifier (*p* = 0.96).

**Conclusion**: Neonatal hypoglycemia, even when persistent, was not associated with obesity at ages 6–11. The high prevalence of obesity is consistent with national data; further research is needed to identify opportunities for prevention.

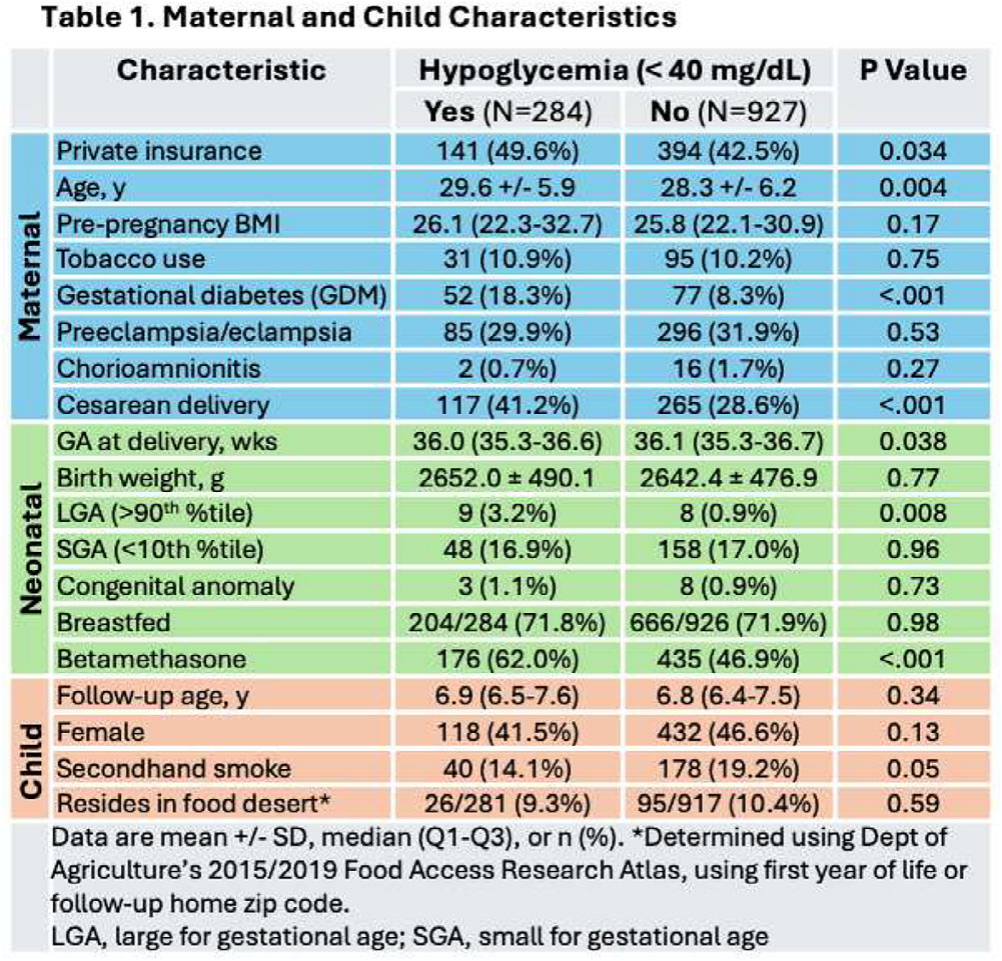

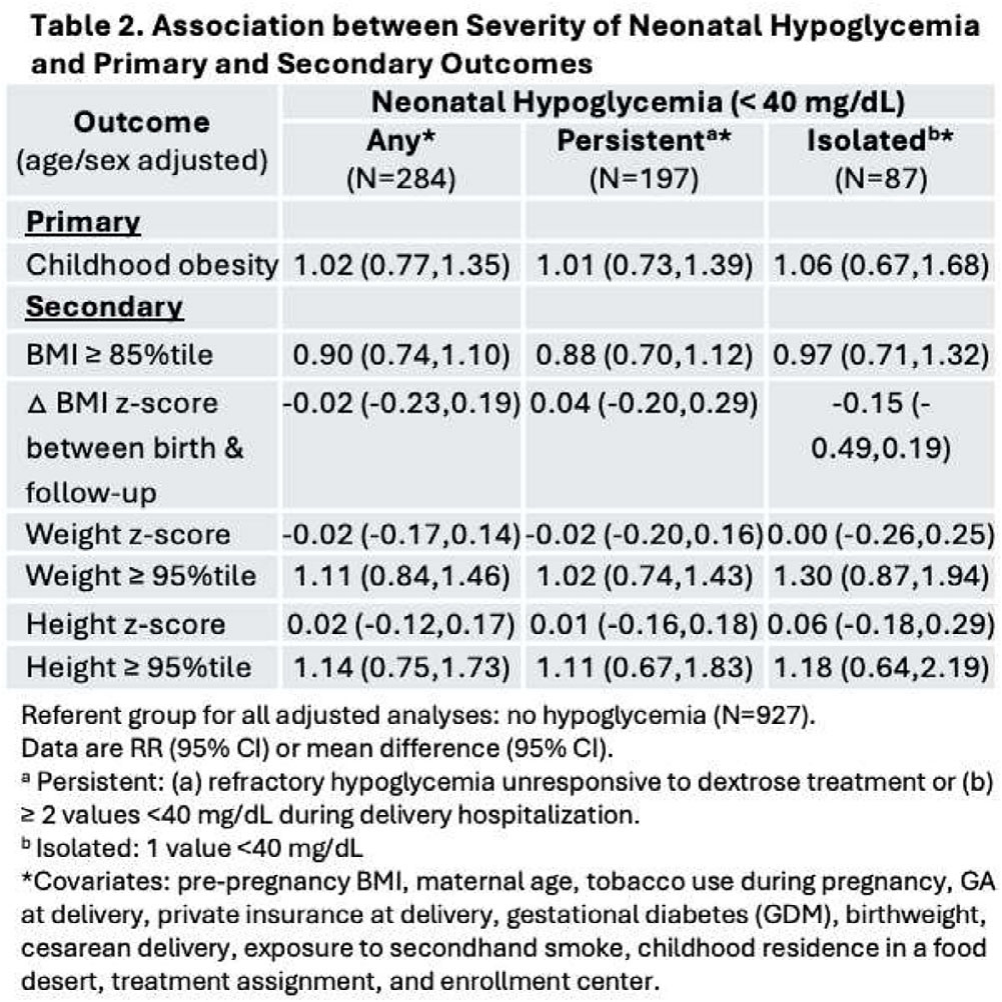

**3:30 PM – 5:00 PM**

## 1056 | **Selective Reduction in Pregnancies With Selective Fetal Growth Restriction: Systematic Review and Meta‐Analysis of Outcomes**

Claudio V. Schenone^1^; Ehsan Rojhani^2^; Nikan Zargarzadeh^2^; Faezeh Aghajani^2^; Francesco D'Antonio^3^; Asma Khalil^4^; Hiba J. Mustafa^5^; Eyal Krispin^1^; Alireza A. Shamshirsaz^1^

^1^Boston Children's Hospital, Harvard Medical School, Boston, MA; ^2^Boston Children's Hospital, Boston, MA; ^3^University of Chieti, University of Chieti, Abruzzi; ^4^St. George's University Hospital, St. George's University Hospital, England; ^5^Division of Maternal‐Fetal Medicine, Indiana University School of Medicine, Indianapolis, IN

**Objective**: To estimate the rates of co‐twin intrauterine demise (IUFD), co‐twin neonatal demise and gestational age (GA) at delivery in monochorionic (MC) pregnancies complicated by selective fetal growth restriction (sFGR) undergoing selective reduction (SR), and stratify the outcomes according to the surgical technique.

**Study Design**: We searched PubMed, Scopus, and Web of Science from inception through May 2025. We included studies reporting MC pregnancies complicated by sFGR undergoing SR with data on co‐twin IUFD or neonatal demise and GA at delivery. A random‐effect model estimated the pooled means and proportions and corresponding 95% confidence intervals (CI). X^2^ compared outcomes between pregnancies managed with radiofrequency ablation (RFA) vs. Bipolar cord coagulation (BCC). The I^2^ value assessed heterogeneity across studies.

**Results**: A total of 21 studies, including 672 pregnancies, met the inclusion criteria and were eligible for quantitative synthesis. The overall rates of co‐twin IUFD and neonatal demise were 14% (9–19) and 4% (2–7), respectively, and the GA at delivery was 35.4 weeks (34.3–36.5). Cases undergoing RFA had higher rates of co‐twin IUFD than those undergoing BCC (20%, 95% CI 13–29 vs. 8%, 95% CI 4–15) (Figure 1). Co‐twin neonatal demise rates were similar (BCC 5% 95% CI: 2–9 vs. RFA 3% 95% CI: 1–8). GA at delivery was lower in cases undergoing BCC, than those undergoing RFA (34.5 95% CI: 33.1–35.9 vs. 36.7 95% CI 35.2–38.4, respectively) (Figure 2).

**Conclusion**: The risk of co‐twin IUFD or neonatal demise in MC pregnancies complicated by sFGR who undergo SR is approximately 18%. BCC appears to be associated with lower rates of IUFD and earlier GA at delivery than RFA.

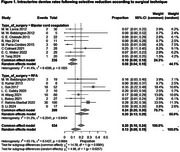

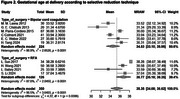

**3:30 PM – 5:00 PM**

## 1057 | **Severe Early‐Onset Preeclampsia is Associated with Compromised Placental Oxygen Signaling via HIF‐1a and Metabolic Dysregulation**

Courtney Johnson‐Gonzalez; Katherine Wilcher; Scout Bowman‐Gibson; Chandni Chandiramani; Bryce Linkous; Emily Stone; Kailey Nolan; David Dhanraj; Rose Maxwell; Thomas L. Brown

Wright State University, Dayton, OH

**Objective**: Hypoxia‐inducible factor 1 (HIF‐1α) is an oxygen‐sensitive transcription factor which has previously been found to be upregulated in placentas of preeclampsia (PE) patients, suggesting placental HIF‐1α plays a significant role in the pathogenesis of the condition when elevated beyond the first trimester. In this study, we sought to determine why HIF‐1α remains elevated in severe, early‐onset PE (sEOPE), and how its elevation leads to metabolic dysfunction in the placenta.

**Study Design**: Placentas from healthy pregnant control (*n* = 12) and severe, early‐onset preeclampsia (sEOPE) (*n* = 13) were taken at the time of delivery and processed for Western blot analysis.

**Results**: HIF‐1α was significantly elevated in sEOPE placentas. VHL, which degrades HIF‐1α, was significantly decreased. Phosphorylated AKT, which stabilizes HIF‐1α, was significantly elevated. A decrease in VHL coupled with increased AKT activity provides a possible mechanism for the sustained elevation of placental HIF‐1α. We then assessed downstream targets to determine the impact of elevated HIF‐1α. Target proteins in glycolysis were significantly elevated. An enzyme critical for B‐oxidation was significantly decreased. The master regulator of cellular energy sensing, pAMPK, was significantly elevated, suggesting low cellular energy. Low cellular energy and HIF‐1α elevation have both been shown to decrease antioxidant capacity, and likewise, we found significantly reduced GPX4 protein and decreased total GSH. A marker of endothelial dysfunction and decreased angiogenesis was also significantly elevated, providing further evidence of increased cellular dysfunction.

**Conclusion**: Severe, early‐onset preeclampsia (sEOPE) is known to result from abnormal placentation resulting in compromised placental oxygen signaling, leading to sustained elevation of placental HIF‐1α. This elevation results in a shift from oxidative phosphorylation to glycolysis, decreased cellular energy, reduced B‐oxidation, and diminished antioxidant capacity. Our results provide compelling evidence for the significance of elevated placental HIF‐1α in sEOPE pathogenesis.

**3:30 PM – 5:00 PM**

## 1058 | **Predicting Red Blood Cell Transfusion After Cesarean Delivery Using Machine Learning: An XGBoost Model**

Daniel Gabbai^1^; Itamar Gilboa^1^; Lee Reicher^2^; Yariv Yogev^3^; Emmanuel Attali^1^

1Lis Hospital for Women's Health, Tel Aviv Sourasky Medical Center, Lis Hospital for Women's Health, Tel Aviv Sourasky Medical Center, Tel Aviv; ^2^Lis Hospital for Women's Health, Tel Aviv Sourasky Medical Center, Tel Aviv, HaMerkaz; ^3^Lis Hospital for Women's Health, Tel Aviv Sourasky Medical Center Gray Faculty of Medicine, Tel Aviv University, Israel, Lis Hospital for Women's Health, Tel Aviv Sourasky Medical Center, Tel Aviv

**Objective**: To develop a predictive model for red blood cell transfusion (PRBC) following cesarean delivery (CD), aiming to enable early identification of high‐risk patients and optimize clinical management.

**Study Design**: A retrospective cohort study (2012–2024) at a university‐affiliated tertiary center included all women undergoing CD. Postpartum PRBC transfusion was defined by severe hemorrhage, symptomatic anemia with Hb 7–8 g/dL, or Hb <7 g/dL. Predictors included clinical and obstetric variables. Data were randomly split into training (70%) and testing (30%) sets. An XGBoost machine learning model was trained to predict  the need for PRBC, with performance assessed by area under the receiver operating characteristic curve (AUC). Variable importance was analyzed to identify key predictors. To assess the individual contribution of each predictor to PRBC transfusion risk, SHAP (SHapley Additive exPlanations) values were calculated.

**Results**: 1. Overall, 31,991 women underwent CD, of whom 661 (2.1%) received red blood cell transfusions.

2. The top predictors based on their relative contribution to model gain were surgery duration (18.6%), pre‐delivery hemoglobin (12.9%), maternal age (12.8%), BMI (10.0%), pre‐delivery platelet count (9.8%), newborn birthweight (9.5%), gestational age (9.0%), and gestational weight gain (GWG) (4.9%). General anesthesia contributed 2.1% to the total model gain.

3. Among CD indications, abnormal placentation was the most important predictor, followed by “other” indications, malpresentation, and abruption.

4. SHAP analysis identified surgery duration as the strongest contributor (mean SHAP: 0.54), followed by pre‐delivery hemoglobin (0.50), BMI (0.38), gestational age (0.34), and maternal age (0.31).

5. The XGBoost model achieved an AUC of 0.83 on the test set.

**Conclusion**: An XGBoost model incorporating clinical, obstetric, and surgical variables can effectively predict the need for RBC transfusion after CD. This tool may assist clinicians in identifying high‐risk patients, guiding perioperative management, and optimizing resource allocation.

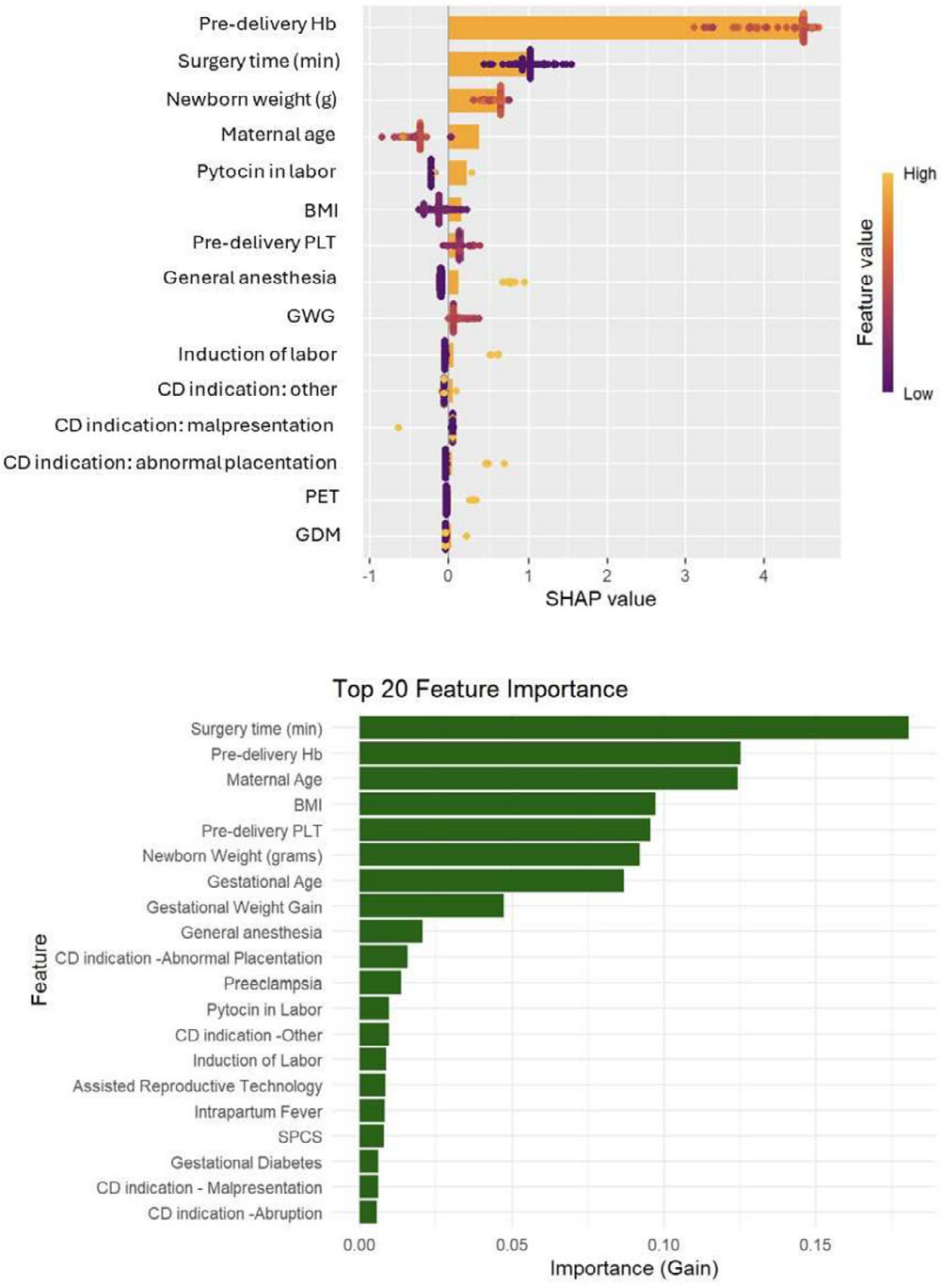

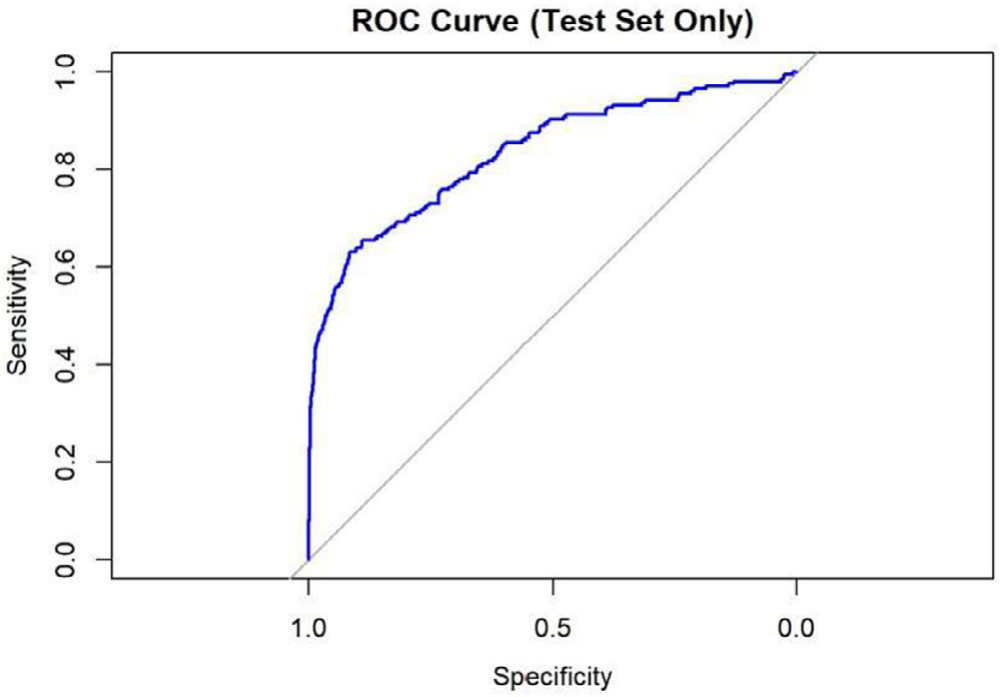

**3:30 PM – 5:00 PM**

## 1059 | **Antenatal Corticosteroid Exposure and Childhood Cancer Risk: A Population‐Based Cohort Study of Singleton Live Births**

Daniel Ling^1^; Tamar Wainstock^2^; Eyal Sheiner^3^; Gali Pariente^4^

^1^Department of Obstetrics and Gynecology, Soroka University Medical Center, Ben‐Gurion University, Beer‐Sheva, HaDarom; ^2^Department of Epidemiology, Biostatistics and Community Health Sciences, Faculty of Health Sciences, Ben‐Gurion University of the Negev, Beer Sheva, HaDarom; ^3^Soroka University Medical Center, Faculty of Health Sciences, Ben‐Gurion University of the Negev, beer sheva, HaDarom; ^4^Department of Obstetrics and Gynecology, Soroka University Medical Center, Ben‐Gurion University, beer sheva, HaDarom

**Objective**: Antenatal corticosteroids (ACS) are widely used to reduce neonatal morbidity in pregnancies at risk of preterm delivery. However, concerns persist regarding potential long‐term harms. We aimed to evaluate the association between ACS exposure before 34 weeks of gestation and the risk of childhood malignancy.

**Study Design**: A population‐based retrospective cohort study was conducted using data from a single tertiary medical center between the years 1991 and 2021. The cohort included singleton infants, whose mothers did or did not receive ACS prior to 34 weeks. Diagnoses of childhood malignancy were identified through linkage with community clinics and hospital records. Kaplan‐Meier survival curve was used to compare the incidence of long‐term malignancy between the study groups. A Cox proportional hazards model was used to control for confounders.

**Results**: The study included 196,377 singleton births, of which 3634 (1.9%) were exposed to ACS before 34 weeks. Childhood malignancy was diagnosed in 9 (0.25%) of the exposed group and in 438 (0.23%) of the unexposed group (*p* = 0.798). The cumulative incidence of malignancy did not significantly differ between groups (Log‐Rank *p* = 0.055, Figure). However, in a Cox proportional hazards model adjusting for gestational age at birth and ethnicity, ACS exposure was independently associated with a modest but statistically significant increased risk of childhood malignancy (adjusted HR: 1.42, 95% CI: 1.014–1.979, *p* = 0.041).

**Conclusion**: In our population, ACS exposure before 34 weeks was associated with a small increase in childhood malignancy risk, regardless of gestational age at birth. While ACS remains essential in managing threatened preterm birth, this association may partly reflect indication bias, as more vulnerable fetuses are more likely to receive treatment. Further research is warranted to clarify long‐term outcomes and guide clinical decision‐making.

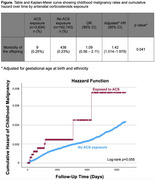

**3:30 PM – 5:00 PM**

## 1060 | **Postpartum Readmission Rates After Implementation of a Standardized Furosemide Protocol**

Daniella W. Spielman^1^; Alahni Becks^2^; Shanza Ashraf^3^; Suchitra Chandrasekaran^2^

^1^Georgia Perinatal Consultants, Atlanta, GA; ^2^Department of Gynecology and Obstetrics, Emory University School of Medicine, Atlanta, GA; ^3^Emory Healthcare, Emory Healthcare, GA

**Objective**: While the utilization of a standardized protocol for postpartum furosemide administration among subjects with hypertensive disorders of pregnancy (HDP) is suggested to reduce postpartum readmission rates, implementation of a protocol on open labor & deliveries can be challenging. We succeeded in implementing this protocol in our high risk urban cohort in a single hospital in our system. We sought to evaluate rates of postpartum readmission before and after protocol implementation.

**Study Design**: In October of 2022, a standardized protocol for postpartum furosemide administration was approved and implemented in our institutional hospital labor & delivery. This study was a retrospective chart review of postpartum patients who delivered within that hospital system and were diagnosed with HDP. The study period prior to intervention was 10/2020–10/2022 and the post was 10/2022–10/2024.

**Results**: Of a total of 18,984 patients who delivered between October 2020 and October 2024, 4,967 patients had a diagnosis of HDP (26%). The was no difference in the two groups (pre‐intervention and post‐intervention) with regards to age, race, gestational age at delivery, and HDP diagnosis [Table 1a]. The implementation of the protocol significantly reduced postpartum readmission rates within the first 7 days, 4.2% pre and 2.8% post (*p* = 0.008) [Table 1b]. Furthermore, postpartum readmission rates within 30, 42, 60 and 90 days continued to show a downward trend after the implementation of the protocol.

**Conclusion**: On October 1, 2022, a standardized furosemide administration protocol was implemented at a large urban academic hospital. Since its initiation, the rates of postpartum readmission have decreased significantly. Further studies can now be performed to look specifically as to postpartum antihypertensive medication usage, and whether furosemide decreases the need for anti‐hypertensive therapy. In addition, this study did not look at adverse events after administration of furosemide. Although thought to be rare, this should be analyzed for safety data.

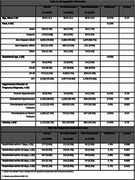

**3:30 PM – 5:00 PM**

## 1061 | **Large Language Model Inference Versus Clinician Judgment for Predicting Intrapartum Fetal Asphyxia From Cardiotocography**

Debjyoti Karmakar^1^; Erica Spessot^2^; Enes Makalic^3^; Fiona Brownfoot^4^

^1^University of Melbourne/Mercy Hospital for Women, Mercy Hospital for Women/Heidelberg, Victoria; ^2^Mercy Hospital for Women, Mercy Hospital for Women, Victoria; ^3^Monash University, Monash University, Victoria; ^4^University of Melbourne/Mercy Hospital for Women, Mercy Hospital for Women/University of Melbourne, Victoria

**Objective**: To assess whether GPT‐4V's image‐based classifications can predict fetal asphyxia

**Study Design**: We conducted a retrospective diagnostic accuracy study to evaluate GPT‐4 with Vision (GPT‐4V) for zero‐shot classification of intrapartum cardiotocography (CTG) screenshots. Prospective evaluation was not feasible due to regulatory constraints (e.g., FDA, HIPAA) and geosecurity concerns regarding data sovereignty. A proof‐of‐concept design was therefore essential to demonstrate technical feasibility, secure infrastructure integration, and baseline clinical utility. Using a balanced dataset of 1000 labor episodes (500 with asphyxia), we compared three approaches: GPT‐4V visual ratings alone (based on the Royal Australian and New Zealand College of Obstetricians and Gynaecologists [RANZCOG] and National Institute of Child Health and Human Development [NICHD] classification systems), structured clinical features alone, and a hybrid model combining both.

**Results**: The RANZCOG‐based hybrid model achieved the highest area under the receiver operating characteristic curve (AUROC 0.71; 95% CI: 0.62–0.78) and 72% sensitivity—representing a 12% absolute gain over clinician judgment at matched specificity (AUROC 0.61). GPT‐4V–only models showed limited predictive value (AUROC 0.55–0.58). For predicting clinician intervention, hybrid and clinical‐only models performed comparably (AUROC 0.83). All analyses were conducted in a geographically restricted, data‐sovereign, encryption‐enabled cloud environment compliant with Health Insurance Portability and Accountability Act (HIPAA, USA) standards.

**Conclusion**: These findings demonstrate that GPT‐4V, when integrated with structured clinical data, can offer scalable, infrastructure‐light decision support for fetal surveillance and lay essential groundwork for regulatory engagement and prospective trials of trustworthy AI‐based tools in maternal–fetal medicine.

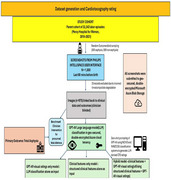

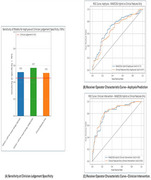

**3:30 PM – 5:00 PM**

## 1062 | **Transcerebellar Diameter as a Predictor of Gestational Age: A Retrospective Cohort Study**

Deepika Khanna^1^; Ananya S. Dewan^1^; Lauren Meiss^2^; Angie C. Jelin^3^

^1^Johns Hopkins University, Baltimore, MD; ^2^Johns Hopkins, Baltimore, MD; ^3^Johns Hopkins Hospital, Baltimore, MD

**Objective**: We evaluated the predictive accuracy of transcerebellar diameter (TCD)‐based gestational age (GA) estimation against the Hadlock equation for well‐dated pregnancies and created nomograms to estimate GA based on TCD.

**Study Design**: This retrospective cohort study utilized data from a multi‐site single academic institution between March 2022 and May 2024. We identified patients scanned between 14w0d‐38w6d. Only well‐dated scans were included (crown‐rump length <12 weeks, last menstrual period consistent with ultrasound <20 weeks, or in vitro fertilization dating). Multifetal gestations and fetal anomalies were excluded. Maternal diabetes and hypertension were analyzed as sub‐groups. For patients with multiple scans during the study period, only the final sonogram was included.

Descriptive statistical analysis examined the trend between TCD measurements and actual GA. A regression analysis was performed to create a nomogram that predicts GA based on TCD alone. Estimated GA based on TCD was compared to actual GA with Pearson's correlation analysis. GA estimated using Hadlock's equation was compared to actual GA using Pearson's correlation. Pearson's analysis was repeated for sub‐groups of maternal hypertension and diabetes.

**Results**: 220 scans were analyzed after using our selection criteria. Concordance between actual GA and estimated GA using TCD was high (r = 0.98, *p* < 0.001, mean error = 0 weeks, mean absolute error = 0.62 weeks). Concordance between actual GA and estimated GA using Hadlock's equation was also high (r = 0.98, *p* < 0.001, mean error = 0.34 weeks, mean absolute error = 0.62 weeks). Outcomes were similar for sub‐groups of maternal hypertension (*n* = 168, r = 0.97, *p* < 0.001) and maternal diabetes (*n* = 42, r = 0.92, *p* < 0.001).

**Conclusion**: Transcerebellar diameter is a highly accurate and reliable predictor of gestational age, as demonstrated by strong correlation with actual gestational age, low mean absolute error of 0.62 weeks, and lack of systemic bias (mean error of 0). TCD alone is comparable with Hadlock's equation, which utilizes several measurements when predicting gestational age.

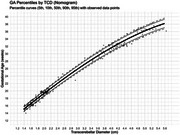

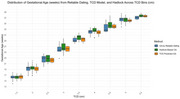

**3:30 PM – 5:00 PM**

## 1063 | **Time Threshold Between Last FHR Acceleration and Delivery Predicting Neonatal Acidemia in Low‐Risk Pregnancies**

Dikla Ben Zvi^1^; Naphtali Justman^2^; Julia Fedorkova^1^; Gilad Shahak^1^; Roee Iluz^1^; Ola Gutzeit^1^; Michael Y Divon^3^; Zeev Weiner^4^

^1^Rambam Health Care Campus, Haifa, Hefa; ^2^Rambam Medical Health Center, Haifa, Hefa; ^3^Department of Obstetrics and Gynecology, Lenox Hill Hospital–Northwell Health, New York, NY; ^4^Rambam Health Care campus, Haifa, Hefa

**Objective**: The primary objective of this study was to establish a simple, visually identifiable, and clinically relevant criterion for real‐time interpretation of fetal heart rate (FHR) monitoring by human observers, aimed at supporting timely recognition of fetal acidemia. We investigated whether the time interval from the last FHR acceleration to delivery could serve as a practical marker to guide intrapartum decision‐making.

**Study Design**: We conducted a retrospective case‐control study using delivery records from term (37–42 weeks), low‐risk singleton pregnancies at Rambam Health Care Campus. Eligible cases had vaginal or operative vaginal delivery, umbilical cord blood gas within 30 minutes, and continuous FHR monitoring from the last acceleration until birth. Neonatal acidemia was defined as umbilical pH <7.0. FHR tracings were reviewed by a blinded reader who annotated the timing of the last acceleration and terminal FHR category. Statistical analysis included ROC curve and bivariate comparisons.

**Results**: The study included 149 participants (36 acidemia, 113 controls), the groups were comparable in maternal and neonatal characteristics as well as in the intrapartum course. The incidence of category 3 fetal heart rate (FHR) tracings during labor did not differ significantly between groups (Fisher's exact test, *p* = 0.116). The mean interval from last acceleration to delivery was significantly longer in the acidemia group (186.0 vs. 75.7 minutes; *p* < 0.001). ROC analysis identified 90 minutes as the optimal cutoff (AUC 0.783).

**Conclusion**: In term, low‐risk pregnancies, a prolonged absence of FHR accelerations prior to delivery is significantly associated with neonatal acidemia. A 90‐minute threshold may assist in identifying fetuses at risk. The terminal fetal heart rate pattern and the interval from the last acceleration should be considered complementary markers of evolving neonatal compromise that may be used together to enhance clinical decision‐making and improve neonatal outcomes.

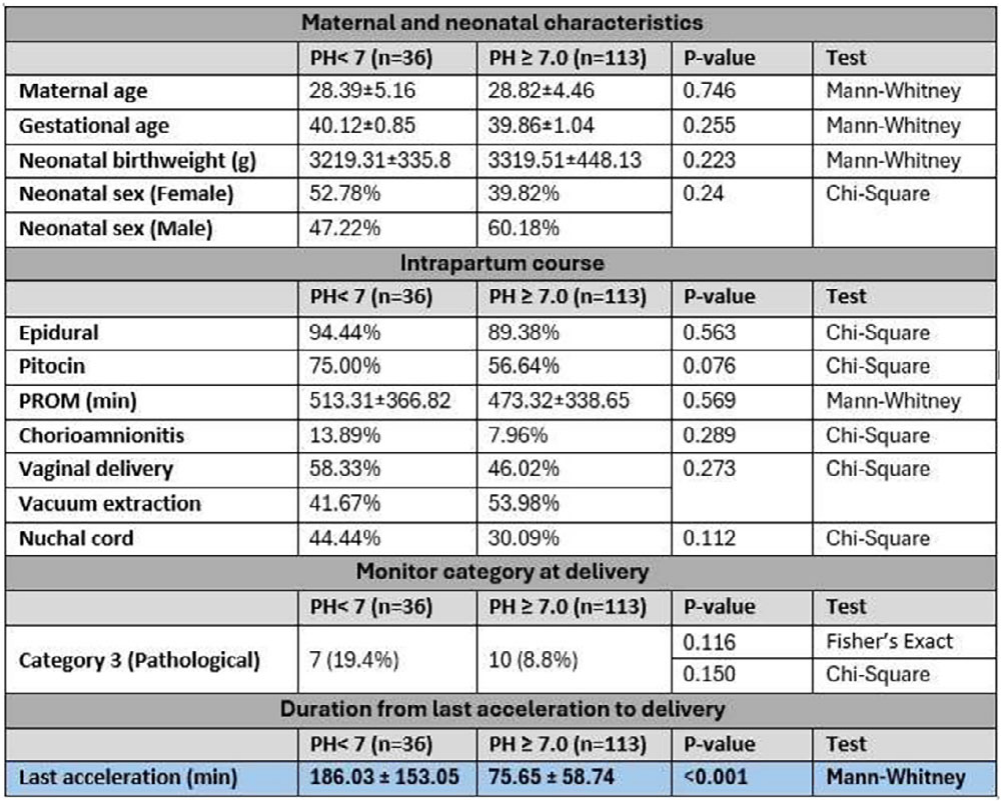

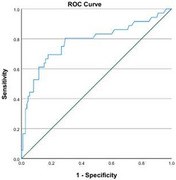

**3:30 PM – 5:00 PM**

## 1064 | **Clinical and Treatment‐Related Predictors of SGA in GDMA2 patients Treated With Metformin**

Dorit Ravid^1^; May Weinberg^2^; Reut Baram^3^; Shani Saffer^4^; Ilil Mishani‐Sagi^4^; Tal Biron Shental^5^; Sivan Farladansky Gershnabel^1^

^1^Department of Obstetrics and Gynecology, Meir Medical Center, Kfar Saba, HaMerkaz; ^2^Meir Medical Center, kfar saba, HaMerkaz; ^3^School of Medicine, University of Zagreb, Zagreb; Croatia, Kfar Saba, HaMerkaz; ^4^Meir Medical Center, Kfar Saba, HaMerkaz; ^5^Meir Medical Center, Meir Medical Center, HaMerkaz

**Objective**: Concerns have emerged regarding a possible association between metformin treatment for gestational diabetes mellitus (GDM) and increased risk of small for gestational age (SGA) neonates. While this has been reported in type 2 diabetes, data in GDMA2 pregnancies remain limited. We aimed to identify clinical and treatment‐related risk factors for SGA among metformin‐treated women with GDMA2.

**Study Design**: We conducted a retrospective cohort study of singleton GDMA2 pregnancies treated with metformin at a tertiary center. Neonates were categorized as SGA (<10th percentile) or appropriate for gestational age (AGA). Maternal, obstetric, and neonatal characteristics were compared. Multivariable logistic regression was performed to identify independent predictors of SGA. Exclusion criteria included pregestational diabetes and fetal anomalies.

**Results**: Of 397 metformin‐treated women, 46 (11.6%) delivered SGA neonates. In univariate analysis, SGA was associated with female fetal sex (62.9% vs. 28.3%, *p* < 0.01), primiparity (43.5% vs. 22.8%, *p* = 0.002), lower prior birth weight (2637g vs. 3023g, *p* = 0.019), and earlier metformin initiation (≤28 weeks: 54.3% vs. 37.1%, *p* = 0.024). In multivariable analysis, female fetal sex (aOR 0.039; 95% CI 0.004–0.373; *p* = 0.005) and lower prior birth weight (aOR 0.999; 95% CI 0.998–1.000; *p* = 0.016) were independently associated with SGA. Initiation of metformin ≤28 weeks was borderline significant (aOR 4.098; 95% CI 0.93–18.01; *p* = 0.062). Metformin dose, duration, insulin co‐treatment, weight gain, and glycemic control were not associated with SGA.

**Conclusion**: Among metformin‐treated GDMA2 pregnancies, fetal female sex and lower prior birth weight were significantly associated with SGA. Early metformin initiation showed a borderline association. These findings highlight the need for a risk‐based and individualized approach to metformin use in GDMA2.

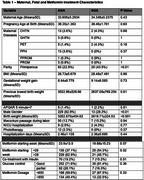

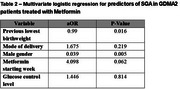

**3:30 PM – 5:00 PM**

## 1065 | **Glucocorticoid Treatment Associated With Significantly Lower Hyperemesis Gravidarum Symptoms and Emetogenic Hormone (GDF15) Levels**

Zainab H. Neemuchwala^1^; Andrew Housholder^2^; Dr. Marlena Fejzo^3^

^1^University of Southern California, University of Southern California/Los Angeles, CA; ^2^The Morning Sickness Clinic, The Morning Sickness Clinic, AL; ^3^University of Southern California, Los Angeles, CA

**Objective**: Hyperemesis Gravidarum (HG), severe nausea and vomiting of pregnancy, is associated with adverse outcomes for mother and baby and can be life‐threatening. Recently, a strong genetic link between HG and the emetogenic hormone GDF15 was discovered. Methylprednisolone has been associated with a reduction of GDF15 in an animal model. Glucocorticoids are used to treat HG when traditional antiemetics fail, and while fetal safety prior to 10 weeks’ gestation may be a concern due to potential association with oral clefts, they can be more effective in reducing symptoms and hospitalization. The mechanism of antiemetic action of glucocorticoids is unknown. We hypothesized that they may work by reducing GDF15 levels.

**Study Design**: We analyzed circulating GDF15 levels (measured by ELISA‐based assay) and HG symptoms (measured by validated HELP scoring tool) before and during treatment in 11 GA matched treatment‐resistant HG patients treated with glucocorticoids (cases) and 11 HG patients treated with other antiemetics (controls) at The Morning Sickness Clinic. A standard t‐test was used to compare means.

**Results**: Cases and controls had similar (*p* > 0.05) levels of GDF15 and HELP scores prior to treatment at ∼12 weeks’ gestation. At ∼14 weeks’ GDF15 levels had decreased in 9/11 cases and 4/11 controls. Cases had significantly greater reduction in GDF15 level (−1.1 ng/ml vs. +1.4 ng/ml, *p* = 0.03) and HELP scores (−19 vs. −11, *p* = 0.03) compared to controls.

**Conclusion**: Use of glucocorticoids in HG pregnancies is associated with a significant reduction in GDF15 levels and improved symptoms compared to traditional antiemetics. Since glucocorticoid use may be associated with risk, medications designed specifically to reduce GDF15 signaling may be favorable. Inhibitors of the GDF15 pathway reduce nausea and vomiting in animal models and are currently in clinical trials for cachexia and HG. This study is the first to show a correlation in reduction in GDF15 and medication effectiveness in reducing HG symptoms, providing clinical evidence to support development of GDF15‐based therapeutics for HG.

**3:30 PM – 5:00 PM**

## 1066 | **Improving Intrapartum Vital Sign Charting via a EHR‐Embedded Decision Support Tool**

Drew M. Hensel^1^; Megan L. Lawlor^2^; Lori M. Stevenson^3^; Amanda C. Zofkie^2^; Sydney M. Thayer^4^; Antonina I. Frolova^2^; Jeannie C. Kelly^4^; Steve Porter^5^; Susan Mann^6^; Roxane Rampersad^2^; Nandini Raghuraman^2^

^1^Washington University School of Medicine, SAINT LOUIS, MO; ^2^Washington University School of Medicine, St. Louis, MO; ^3^Barnes‐Jewish Hospital, St. Louis, MO; ^4^Washington University School of Medicine, Washington University School of Medicine, MO; ^5^Case Western Reserve University, University Hospitals MacDonald Women's Hospital, Cleveland, OH; ^6^Beth Israel Deaconess Medical Center, Harvard Medical School, Boston, MA

**Objective**: Accurate and timely documentation of maternal vital signs during labor is essential for early detection of intrapartum diagnoses, including treatable hypertension and infection. We evaluated whether riskLD, an electronic health record (EHR)‐embedded clinical decision support tool issuing real‐time alerts, improved compliance with prompt vital sign documentation.

**Study Design**: This is a pre‐post cohort study of patients admitted for delivery over a 24‐month period. Scheduled cesareans were excluded. The primary outcome was compliance with vital sign documentation, defined as ≥ 90% of vital signs documented per 4‐hour period prior to rupture of membranes (ROM) and per 2‐hour period following ROM. The secondary outcome was percent compliance with each specific vital sign documentation. Outcomes were compared between the pre‐implementation cohort (3/1/23–1/31/24) and post‐implementation cohort (2/1/24–2/28/25) using bivariate statistical tests and multivariable logistic regression. All results were stratified by compliance pre‐ROM and post‐ROM.

**Results**: 3123 patients were in the pre‐implementation cohort and 3820 were in the post‐implementation cohort. Vital sign charting compliance was higher in the post‐implementation cohort both pre‐ROM (13.6% vs 11.6%, aOR 1.17 [1.02–1.36]) and post‐ROM (57.2% vs 51.7%, aOR 1.24 [1.12–1.37]), Table 1. This difference was largely driven by improvements in maternal temperature documentation pre‐ and post‐ROM, Table 2.

**Conclusion**: Implementation of EHR‐based alerts with vital sign reminders increased compliance with vital sign documentation, highlighting the potential value of clinical decision‐support tools in optimizing labor and delivery care.

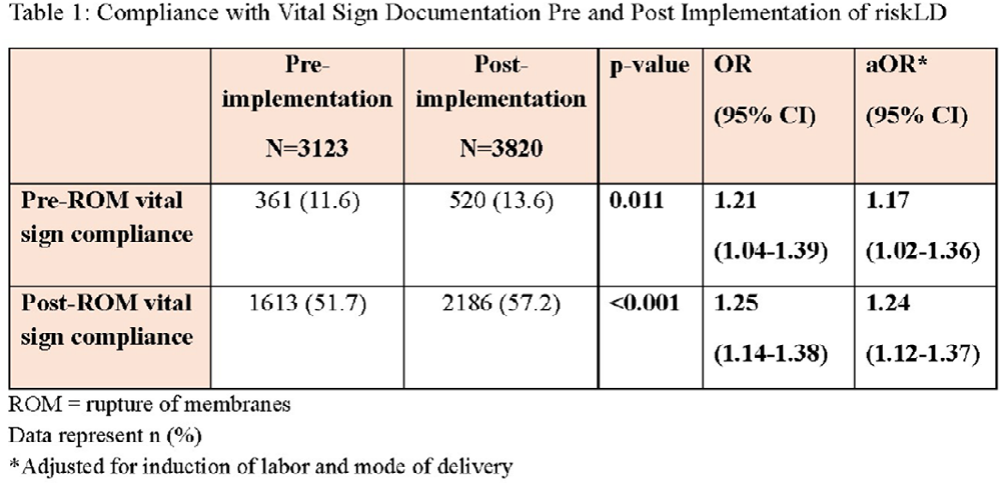

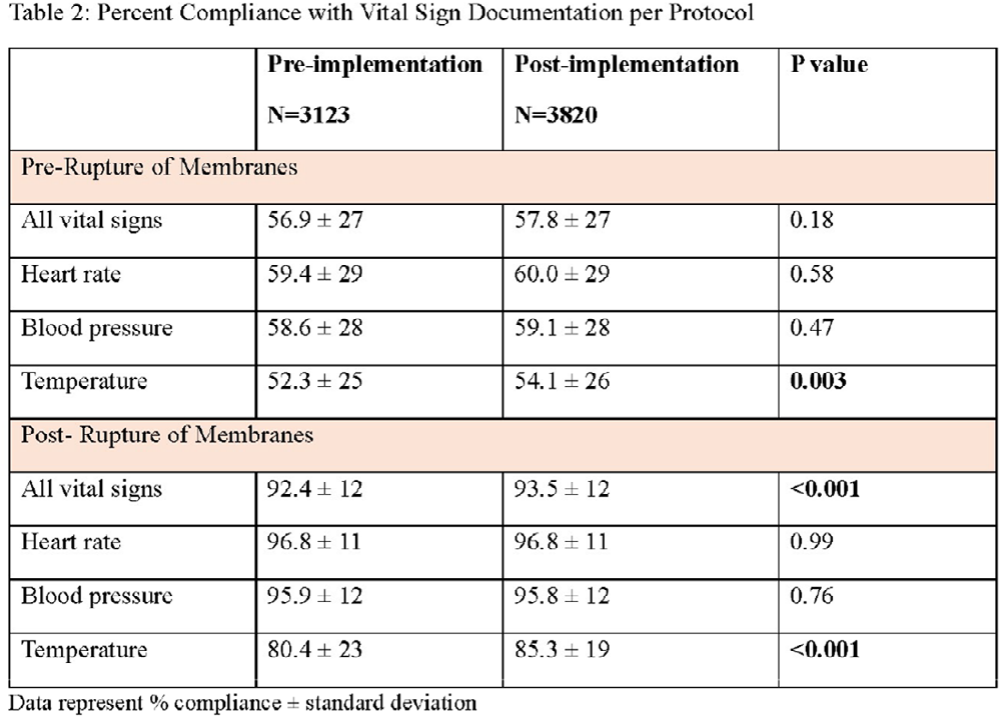

**3:30 PM – 5:00 PM**

## 1067 | **Trends in Adverse Pregnancy Outcomes Following Publication of the CHAP Trial**

E. Nicole Nicole Teal^1^; Isabel Katlaps^2^; Cynthia Gyamfi‐Bannerman^1^; Timothy Wen^1^

^1^Division of Maternal Fetal Medicine, Department of Obstetrics, Gynecology and Reproductive Sciences, University of California San Diego, San Diego, CA; ^2^Department of Obstetrics, Gynecology and Reproductive Sciences, University of California San Diego, San Diego, CA

**Objective**: In 2022, the CHAP (Chronic Hypertension and Pregnancy) trial was published demonstrating active treatment of mild chronic hypertension (CHTN) during pregnancy to a goal of <140/90 reduces a composite of adverse pregnancy outcomes (APOs) <35 weeks. We assess national trends in these APOs since CHAP's publication.

**Study Design**: This retrospective cohort study included all individuals with CHTN and birth hospitalizations from 1/2020–6/2025 identified from a national data network aggregating records from > 1600 hospitals. The primary outcome was a composite of APOs including PTB <35 weeks, superimposed preeclampsia with severe features, placental abruption/antepartum hemorrhage, and stillbirth or neonatal demise. Outcomes were compared between pre‐CHAP (1/2020–4/2022) and post‐CHAP (1/2023–6/2025), excluding 5/2022–12/2022 as a washout period following CHAP publication. Chi‐squared tests assessed outcome differences by period. Unadjusted and adjusted logistic regression models evaluated the effect of the intervention period, adjusting for maternal race, payor, pre‐delivery body mass index, multiparity, pregestational diabetes, and smoking. Sensitivity analyses were stratified by whether individuals delivered in one or both periods.

**Results**: During the study period, 322,434 births met inclusion criteria with 123,820 (38.7%) occurring in the pre‐CHAP and 197,614 (61.3%) in the post‐CHAP period. Post‐CHAP births had a higher prevalence of composite APOs (18.8% vs. 17.9%, *p* < 0.01) compared to pre‐CHAP births. PTB <35 weeks decreased significantly from 10.3% to 9.9%, while the other individual components increased significantly (Figure 1). After adjustment, births in the post‐CHAP period had a 6% higher likelihood of composite APOs (aOR 1.06, 95% CI: 1.04, 1.08) compared to those in the pre‐CHAP period (Table 1). Sensitivity analyses noted similar findings.

**Conclusion**: In this national analysis, the incidence of the CHAP APO composite increased from pre‐ to post‐CHAP but PTB <35 weeks decreased. Further investigation may elucidate opportunities for achieving the outcomes of CHAP in a real‐world setting.

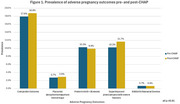

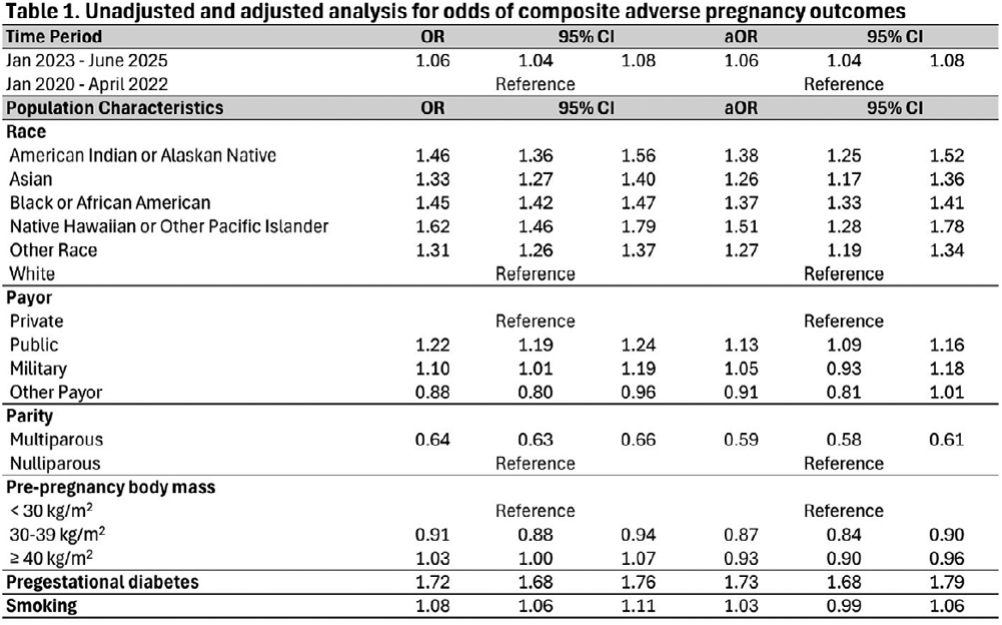

**3:30 PM – 5:00 PM**

## 1068 | **A Risk Model for Intrapartum Cesarean Delivery in Women With BMI ≤18.5**

Einat Tako^1^; Itai Atar^1^; Itamar Gilboa^2^; Daniel Gabbai^2^; Yariv Yogev^3^; Emmanuel Attali^2^

^1^Lis Hospital for Women's Health, Tel Aviv Sourasky Medical Center, Tel Aviv, Israel, Tel Aviv, Tel Aviv; ^2^Lis Hospital for Women's Health, Tel Aviv Sourasky Medical Center, Lis Hospital for Women's Health, Tel Aviv Sourasky Medical Center, Tel Aviv; ^3^Lis Hospital for Women's Health, Tel Aviv Sourasky Medical Center Gray Faculty of Medicine, Tel Aviv University, Israel, Lis Hospital for Women's Health, Tel Aviv Sourasky Medical Center, Tel Aviv

**Objective**: As scarcity of data exists regarding risk factors for intrapartum cesarean delivery (CD) in underweight women, we aimed to identify these risk factors and develop a risk prediction score in women with BMI ≤18.5.

**Study Design**: A retrospective cohort study conducted at a single university‐affiliated tertiary center included women with BMI ≤18.5 who planned a trial of labor between 2012 and 2025. Women who opted for elective CD or with nonviable fetuses were excluded. Maternal, obstetric, and neonatal characteristics were compared between women who delivered vaginally and those who underwent intrapartum CD. Risk factors were assessed using univariate and multivariate analyses. Risk prediction score was developed based on a multivariate model. Model performance was evaluated using receiver operating characteristic curve analysis.

**Results**: 1. During the study period, 147,045 women delivered at our center, of whom 8956 (6.1%) had BMI ≤18.5 and attempted a trial of labor.

2. Among them, 292 (3.3%) had intrapartum CD with the most common was non reassuring fetal heart rate (50%) followed by arrest of dilatation, descent (12.6%). Other indications included multiple pregnancy‐related indications (5.5%), failed induction (3.7%) and failed vacuum extraction (2.7%).

3. Independent risk factors included **previous CD** (OR 8.61, 95% CI 4.92–15.04), **multiple gestation** (OR 5.52, 95% CI 3.26–9.34), intrapartum **antibiotic use** (OR 4.93, 95% CI 3.81–6.39), **nulliparity** (OR 4.53, 95% CI 3.18–6.47), **induction of labor** (OR 2.49, 95% CI 1.86–3.32), **preeclampsia** (OR 2.41, 95% CI 1.15–5.06) and **maternal age >40** (OR 2.06, 95% CI 1.28–3.32).

4. Using a cutoff of 7, with sensitivity of 78% and specificity of 77%, a risk score incorporating these factors demonstrated good predictive performance with AUC of 0.83.

**Conclusion**: This study provides actionable insights into intrapartum CD risk among underweight women. The proposed prediction model offers a practical tool to support clinical decision‐making, optimize labor management and improve maternal and neonatal outcomes for underweight women.

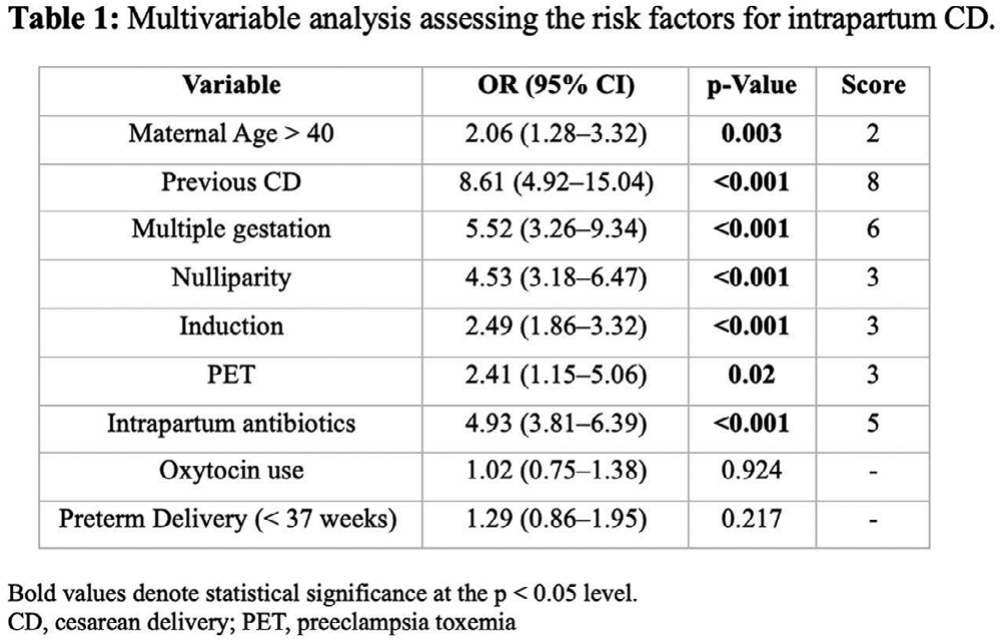

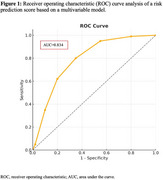

**3:30 PM – 5:00 PM**

## 1069 | **Intermittent Versus Continuous Bladder Catheterization in Epiduralized Laboring Patients: A Pilot Randomized Controlled Trial**

Elena Lands^1^; Nicole Meckes^2^; Kai Holder^3^; Jess Sciuva^2^; Francesca Facco^2^; Lauren Giugale^2^; Anna B. Binstock^4^

^1^University of Pittsbugh Medical Center Magee‐Womens Hospital, Pittsburgh, PA; ^2^UPMC, UPMC, PA; ^3^UPMC Magee‐Womens Hospital, Pittsburgh, PA; ^4^UPMC Magee‐Womens Hospital, UPMC, PA

**Objective**: Intrapartum bladder overdistension can lead to postpartum urinary retention (PUR) as well as long‐term voiding dysfunction and has not yet been assessed as the primary outcome of a trial. We performed a pilot randomized controlled trial to assess the feasibility of detecting PUR rates after intermittent versus continuous bladder catheterization in laboring patients with neuraxial anesthesia.

**Study Design**: Participants in labor with term singletons planning vaginal delivery were randomized to intermittent (IC) or continuous catheterization (CC) after epidural anesthesia initiation. Our primary efficacy outcome was urinary retention within three days postpartum. Secondarily, we assessed voiding dysfunction at 2 and 6 weeks via the UDI‐6 questionnaire, positive urine cultures within 2 weeks, and patient and nurse satisfaction. We assessed feasibility of recruitment and retention, effectiveness of randomization, and fidelity to the protocol. This pilot enrolled 10% of the planned population based on detecting a 60% difference in published rates of PUR after IC and CC. Group comparisons of PUR and UTI were assessed with logistic regression in an intention‐to‐treat fashion.

**Results**: 59 (82%) of 72 patients approached were randomized with 73% retained for follow‐up questionnaire. All patients’ outcome data were recorded appropriately. 10% of participants were catheterized at frequencies deviating from the protocol. Characteristics were similar between groups indicating effective randomization. The rate of PUR was 5% with no significant difference between CC vs. IC groups (OR 2.15, 95% CI 0.18–25.1). UTI rates were similar (OR 0.32, 95% CI 0.032–3.28), as were UDI‐6 scores at 2 and 6 weeks postpartum. Nursing satisfaction was significantly higher for CC (97 vs. 60%, *p* < 0.001).

**Conclusion**: Recruitment and retention of subjects for a randomized controlled trial comparing the effect of IC to CC on PUR is feasible. Reasons for protocol deviation were collected to guide future research. CC was better accepted by staff. A larger study may uncover a difference in PUR that would guide catheterization practices.

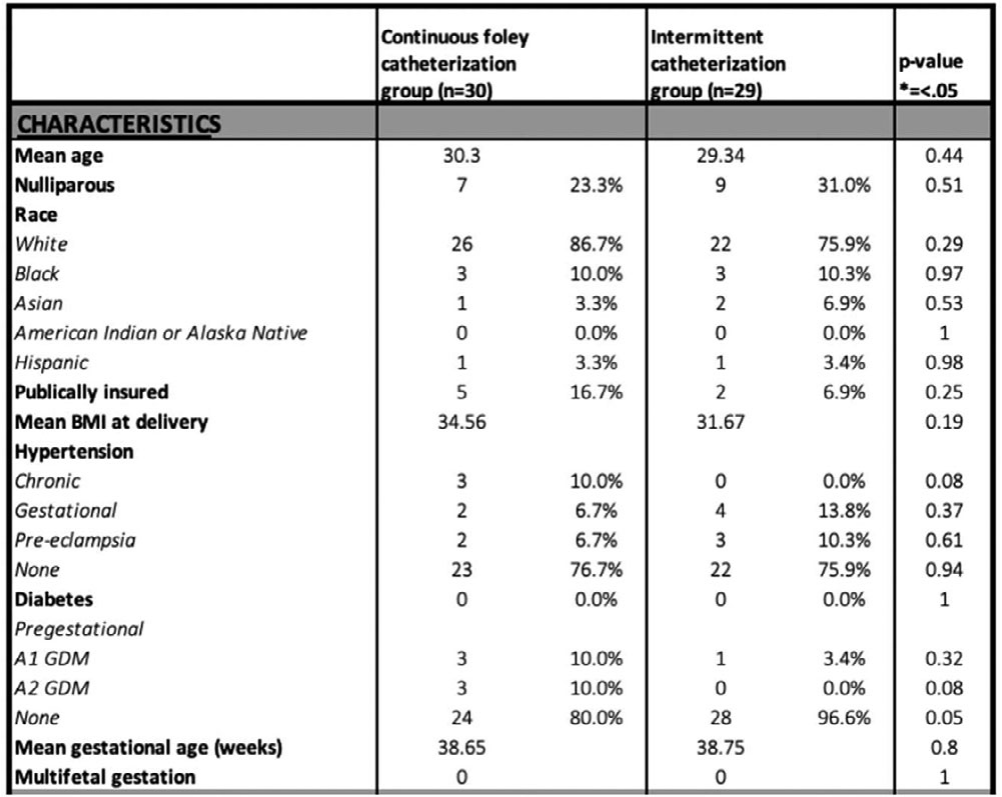

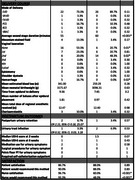

**3:30 PM – 5:00 PM**

## 1070 | **Geographic and Social Burden of InterIsland Travel for Obstetric Delivery in Hawai'i**

Elizabeth Mercer^1^; Kyle M. Ishikawa^1^; April Binari^1^; Ashley Graham^2^; Corrie Miller^1^; Hyeong J. Ahn^3^; Men‐Jean Lee^1^

^1^University of Hawaii, Honolulu, HI; ^2^Pacific Basin Telehealth Resource Center, Honolulu, HI; ^3^University of Hawaii John A Burns School of Medicine, Honolulu, HI

**Objective**: The objective of this study was to quantify the rate of pregnant patients in Hawai'i who must travel from neighbor islands to O'ahu for delivery and to measure the average distance traveled from their home ZIP code to the delivery hospital.

**Study Design**: We conducted a population‐based analysis using Hawaiʻi birth registry data from 2014 to 2024, including all hospital deliveries statewide. For each ZIP code, we calculated the rate of interisland deliveries per 1000 births and measured the driving and flight distance between the patient's home and the delivery hospital.

**Results**: As shown in Figure 1, the highest rates of interisland travel for delivery originated from Hawai'i Island (Big Island), followed by Maui and Kaua'i. Ni'ihau and Lana'i do not have delivery hospitals and only low risk, midwife attended deliveries occur on Moloka'i. Figure 2 demonstrates a wide distribution of travel distances among the 124,535 patients. On O'ahu, travel distances to delivery hospitals were comparable between short (31.5%), medium (32.5%) and long (32.9%) distances. Patients traveling from remote islands (Ni'ihau, Lana'i, and Moloka'i) accounted for 0.8% (1024), while those from other neighbor islands accounted for 2.3% (2851).  Common reasons for interisland delivery included desire for trial of labor after cesarean, anticipated delivery before 35 weeks (due to the lack of NICU services on neighbor islands), and requirement for IV insulin during labor.

**Conclusion**: Patients traveling from neighbor islands to O'ahu for delivery face significant geographic and social barriers, including housing insecurity, transportation logistics, and limited family support. Many patients must remain on O'ahu for extended periods if a NICU stay is required. Identifying ZIP codes with the highest burden of travel can inform targeted support services to reduce maternal stress, financial burden, and health inequities.

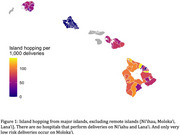

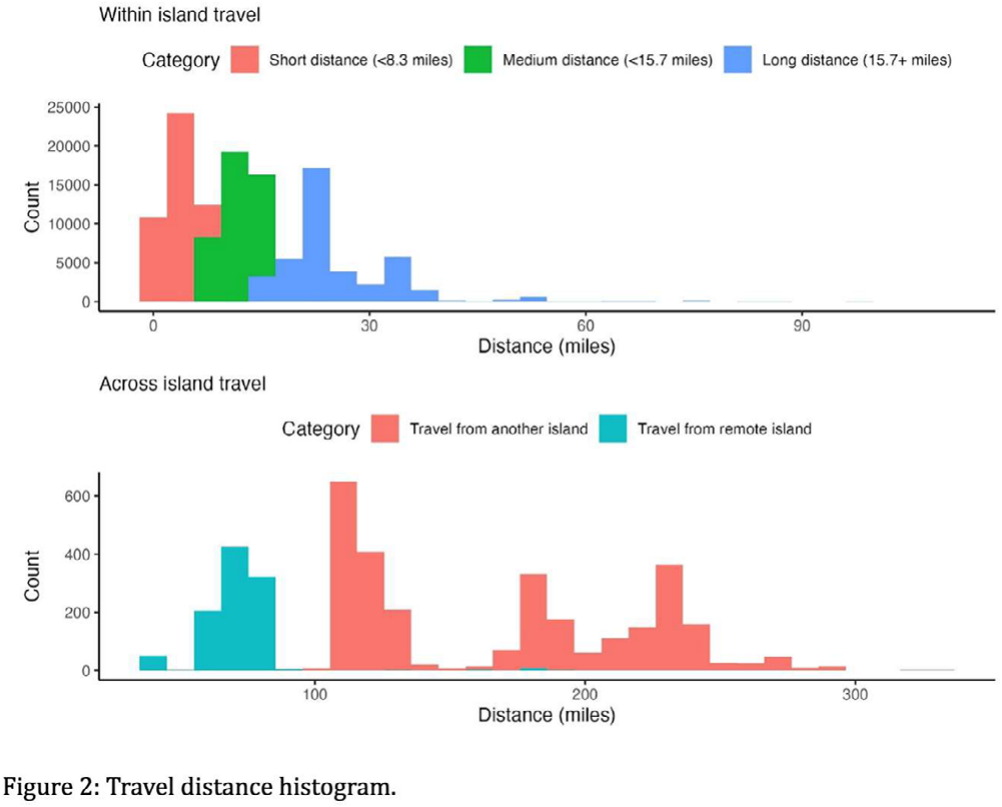

**3:30 PM – 5:00 PM**

## 1071 | **Congenital Heart Disease in Pregnancy and Severe Maternal Morbidity: A Distributed Data Network Study**

Elizabeth B. Sherwin^1^; Anna Booman^1^; Alyssa Howren^1^; Kimberlee McKay^2^; Sadaf Kazi^3^; Katie Sherwin^4^; Benjamin Martin^5^; Xiao Xu^6^; Katherine Bianco^7^; Stephanie A. Leonard^8^

^1^Stanford University, Stanford, CA; ^2^Avera Research Institute, Sioux Falls, SD; ^3^MedStar Health Research Institute, Washington, DC; ^4^Stanford Children's Health, Stanford, CA; ^5^Johns Hopkins University, Baltimore, MD; ^6^Columbia University Irving Medical Center, New York, NY; ^7^Stanford University Healthcare, Palo Alto, CA; ^8^Stanford University, Stanford University, CA

**Objective**: A growing number of pregnant people have congenital heart disease (CHD), but the limitations of traditional study designs and data sources have resulted in insufficient evidence on outcomes for these patients. We leveraged a distributed data network of electronic health record (EHR) data through the Observational Medical Outcomes Partnership (OMOP) common data model (CDM) to assess the incidence of severe maternal morbidity (SMM) among pregnant people with and without CHD.

**Study Design**: Our distributed data network study included female patients aged 12–55 who delivered at three U.S. academic healthcare institutions with EHRs converted into the OMOP CDM. Analytical code was developed at the primary study institution and then executed locally at all sites. We used OMOP standard concepts to identify CHD and SMM from structured and unstructured EHR data. Following the CDC definitions, we identified SMM indicators occurring between one month prior to delivery through 6 weeks postpartum. We categorized outcomes as SMM, non‐transfusion SMM, and cardiac SMM. We analyzed demographic characteristics and incidence of the outcomes by CHD status, by site and aggregated across sites.

**Results**: CHD prevalence was 2.0% among 149,212 deliveries across three sites. Distribution of race and ethnicity varied by site and age distribution was similar across sites (Table 1). SMM occurred in 9.0% of deliveries to people with CHD compared to 3.9% among people without CHD, and non‐transfusion SMM occurred in 6.2% vs 2.3% of deliveries, respectively. Cardiac SMM represented 50% of non‐transfusion SMM cases among people with CHD compared to 16% of cases among people without CHD (Table 2).

**Conclusion**: Our distributed network study of EHR data from three geographically diverse U.S. healthcare institutions found that 1 in 11 people with CHD had SMM compared with 1 in 26 people without CHD. This study provides novel evidence for counseling obstetric patients with CHD and lays the groundwork for expanded network studies on CHD in pregnancy.

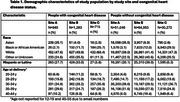

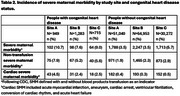

**3:30 PM – 5:00 PM**

## 1072 | **Can Maternal Height and Fetal Head Circumference Predict Labor Dystocia? An Evaluation of Diabetic Patients**

Ella Pardo^1^; Neta Mano^2^; Lior Heresco^3^; Omer Weitzner^3^; Tal Biron‐Shental^3^; Gal Cohen^3^

^1^Meir Medical Center, Meir Medical Center, Kfar Saba, Israel, HaMerkaz; ^2^Tel Aviv University, Tel Aviv University, HaMerkaz; ^3^Meir Medical Center, Kfar Saba, HaMerkaz

**Objective**: Labor dystocia (LD) is a leading cause for cesarean delivery (CD). Maternal anthropometrics are known determinants of delivery outcomes. Recent data suggest that fetal head circumference‐to‐maternal height ratio (HC/MHr) exceeding 2.09, outperforms either parameter alone in predicting LD. This study aimed to assess whether HC/MHr can predict CD for LD among patients with gestational diabetes mellitus (GDM) undergoing induction of labor (IOL).

**Study Design**: A retrospective cohort study of all GDM patients who underwent IOL between 2014 and 2023 at a tertiary hospital. All patients had pre‐IOL sonographic fetal biometry, including head circumference, biparietal diameter, femur length, and abdominal circumference. The cohort was divided into two groups based on HC/MHr: high HC/MHr (>2.09) and low HC/MHr (≤2.09). Maternal and obstetric characteristics, as well as delivery outcomes, were compared between groups. The predictive performance of HC/MHr for CD due to LD was assessed using receiver operating characteristic (ROC) curve analysis.

**Results**: Among 845 patients, 693 (82.0%) achieved vaginal delivery, while 141 (16.9%) underwent CD, 77 (9.1%) for LD. Compared to the low‐ratio group, the high HC/MHr group had higher obesity rates (43.2% vs. 33.8%, *p* = 0.019) but lower rates of nulliparity (30.7% vs. 39.1%, *p* = 0.013) and hypertensive disorders (7.2% vs. 15.1%, *p* < 0.001). Labor characteristics and delivery outcomes were similar between groups. In a multivariable analysis adjusted for potential confounders, HC/MH >2.09 was independently associated with an increased risk of CD for LD (adjusted odds ratio 1.87; 95% CI, 1.02–3.42; *p* = 0.04). However, the discriminative ability of HC/MHr was limited (AUC = 0.51).

**Conclusion**: Among GDM patients undergoing IOL, a high HC/MHr was independently associated with an increased risk of CD for LD, but had poor predictive value, limiting its utility as a screening tool.

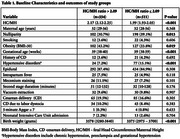

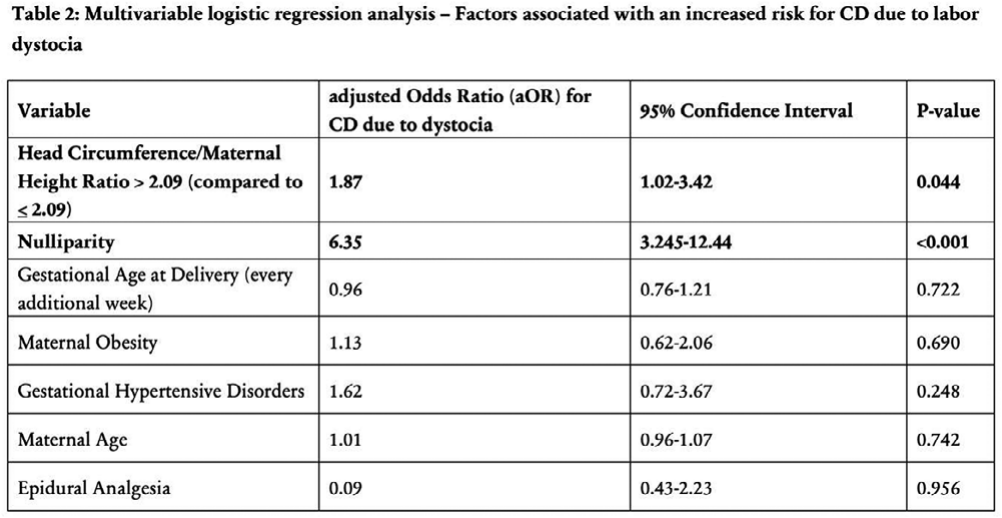

**3:30 PM – 5:00 PM**

## 1073 | **Data Missingness as a Risk Factor for Preterm Birth**

Emily Wong^1^; Tiffani Bright^1^; Melissa S. Wong^2^

^1^Cedars‐Sinai Medical Center, Los Angeles, CA; ^2^Cedars‐Sinai Health Sciences University, Los Angeles, CA

**Objective**: Complications from preterm births (PTB) remain the leading cause of death among children below 5. Predictive models of PTB aim to estimate risk to enable early, evidence‐based interventions. These models require complete or imputed data—assuming similar relationships between predictive factors and PTB across patients with complete and incomplete data—which may compromise predictive accuracy. The aim of this study is to assess missingness in data collected from a prospective cohort study and whether this impacts estimation of PTB risk.

**Study Design**: A retrospective analysis on 8955 patients from the Nulliparous Pregnancy Outcomes Study: Monitoring Mothers‐to‐Be (nuMoM2b) with documented gestational age. We identified variables previously associated with PTB or considered social determinants of health and would be available for screening at early prenatal visits (Table 1). 5133 patients had complete data across all fields in Table 1 (complete), and 3822 were missing data in one or more fields (incomplete). We estimated a logistic regression model from complete records, including all predictors in Table 1, and separate models for each predictor for complete and incomplete records.

**Results**: Incomplete data was associated with 1.6 times the odds of PTB, *p *<  .001. Among participants with complete data, obesity, tobacco use, and poverty are predictive of preterm birth while alcohol and college are protective (all *p *<  .05), consistent with previous studies (Figure 1). By contrast, each of these effects was no longer statistically significant among those with incomplete records (*p* > 0.05) suggesting missingness may attenuate these associations.

**Conclusion**: Even in a rigorously collected prospective study, data missingness persists. Our findings suggest that data missingness itself may signal an elevated PTB risk, acting as a proxy for structural determinants of health. Recognizing the implications of this inequity moving forward is critical for training models and future research should focus on mitigation strategies in parallel with implementation efforts to prioritize equitable data collection.

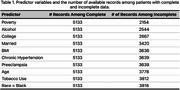

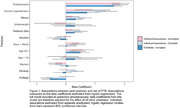

**3:30 PM – 5:00 PM**

## 1074 | **Interpregnancy Interval as a Risk Factor for Pregnancy‐Induced Hypertension**

Emma Altieri^1^; Julia Burd^2^; Candice L. Woolfolk^3^; Katherine H. Bligard^1^; Jeannie C. Kelly^3^; Nandini Raghuraman^4^; Alison G. G. Cahill^5^; Antonina I. Frolova^4^

^1^Washington University School of Medicine, Saint Louis, MO; ^2^Perelman School of Medicine, University of Pennsylvania, St. Louis, MO; ^3^Washington University School of Medicine, Washington University School of Medicine, MO; ^4^Washington University School of Medicine, St. Louis, MO; ^5^Department of Women's Health, Dell Medical School at the University of Texas at Austin, Department of Women's Health, Dell Medical School at the University of Texas at Austin, TX

**Objective**: Hypertensive disorders of pregnancy are a leading cause of maternal and fetal morbidity and mortality. This study aimed to evaluate the relationship between interpregnancy interval (IPI) and risk of maternal pregnancy‐induced hypertension (PIH).

**Study Design**: We conducted a secondary analysis of patients with one prior vaginal delivery and a subsequent singleton gestation delivered at ≥ 37 weeks between 2004 and 2015. The primary outcome was rate of PIH in the second (G2) pregnancy, categorized by IPI <2 years, 2–4 years, and > 4 years. Secondary outcomes included individual diagnoses within PIH. Results were further stratified by presence or absence of maternal obesity (BMI ≥ 30 kg/m2) in the G2 pregnancy. Multivariable regression was used to estimate odds ratios for the primary and secondary outcomes and adjust for potential confounders.

**Results**: Of the 1435 patients, 478 (33%) had an IPI of <2 years, 627 (44%) had an IPI of 2–4 years, and 330 (23%) had an IPI of > 4 years. Maternal age and race were significantly different between groups, while the prevalence of chronic hypertension and diabetes was not. Longer IPI was also associated with the development of obesity. Patients with an IPI of > 4 years had a significantly higher odds of PIH compared to those with IPI <2 years (aOR = 2.30; 95% CI 1.42–3.71; Table). A similar trend was seen for gestational hypertension (aOR = 2.29; 95% CI 1.28–4.08) as compared to those with an IPI of <2 years. When stratified by obesity, the increased odds of PIH among those with an IPI of > 4 years remained significant among patients without obesity (aOR = 3.19; 95% CI 1.35–7.51) but did not reach significance for those with obesity. However, there was no significant interaction by obesity status (p for interaction = 0.39).

**Conclusion**: Longer IPI is associated with increased risk of PIH even after adjusting for maternal age. Although the association appeared stronger among individuals without obesity, maternal obesity did not significantly modify this relationship. These findings suggest that IPI may be an important risk factor for PIH, independent of age and obesity.

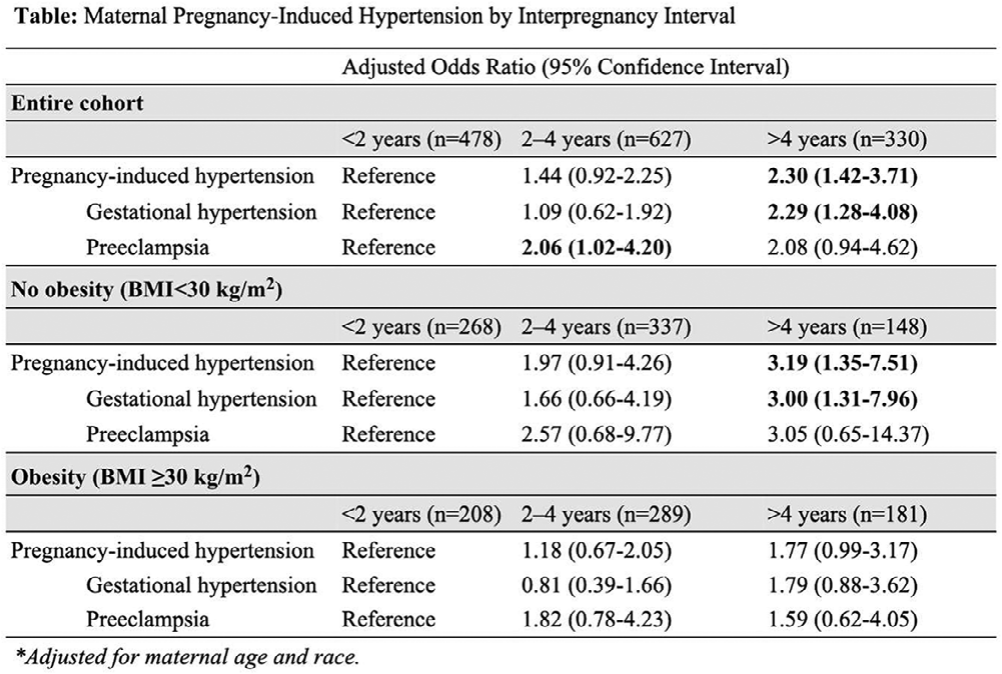

**3:30 PM – 5:00 PM**

## 1075 | **Closing the Evidence‐to‐Practice Gap: Evaluating Implementation of the CHAP Trial**

Emma Keegan^1^; Calliope O'Shea^2^; Jeanine M. Jourdain^3^; Rebecca F. Hamm^4^

^1^Pennsylvania Hospital of the University of Pennsylvania, Philadelphia, PA; ^2^Capital Health, Hopewell, NJ; ^3^CUNY School of Medicine, New York, NY; ^4^Perelman School of Medicine, University of Pennsylvania, Philadelphia, PA

**Objective**: The Chronic Hypertension and Pregnancy (CHAP) trial (Tita 2022) demonstrated improved pregnancy outcomes for patients with mild chronic hypertension (CHTN) when treating to a goal blood pressure (BP) ≤140/90, without impacting fetal growth. Here, we evaluate current adherence to this recommendation in order to identify levers for improved implementation.

**Study Design**: We conducted a single‐site, retrospective cohort study of patients with CHTN (≥2 BPs ≥140/90 at <20 weeks gestation) who delivered at ≥20 weeks between 7/1/2023–12/31/2023. Baseline characteristics and prenatal BP management were analyzed using descriptive statistics. A “missed opportunity” for BP optimization per CHAP was defined as ≥2 BPs ≥140/90 at <23 weeks gestation without medication initiation or uptitration. Patients with and without missed CHAP opportunities were compared using chi‐square or Wilcoxon rank‐sum as appropriate.

**Results**: Among 134 included patients, mean maternal age was 34(+/‐5) and median BMI was 37(IQR 32–43). 49% identified as White, 37% Black, 10% Latinx, and 2% as Asian. 25% began pregnancy on medication(s), and 35% of the cohort met criteria for a change in BP management per CHAP. When made, BP medication changes were most often made by a prenatal care provider (69%), followed by a maternal fetal medicine physician (25%). Changes occurred primarily at a prenatal care visit (69%), and secondarily on asynchronous chart review (9%) or an obstetric triage visit (6%). 17% of the overall cohort (*n* = 23) had missed CHAP opportunities. These patients were more likely to receive care in a faculty practice (as compared to resident‐run clinic), be nulliparous, and have been newly diagnosed with CHTN during pregnancy (Table).

**Conclusion**: This study highlights opportunities for improved evidence‐based BP management in pregnancy. Targeted interventions, such as electronic medical record alerts identifying eligible patients during prenatal care, may enhance adherence to guidelines, thereby improving pregnancy outcomes.

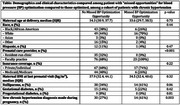

**3:30 PM – 5:00 PM**

## 1076 | **Discrimination and Generalized Anxiety in a High‐Risk Obstetrics Population**

Emmanuel O. Elijah^1^; Dana R. Canfield^2^; Rachel L. Wiley^3^; Minhazur R. Sarker^3^; Marni B. Jacobs^3^; Cynthia Gyamfi‐Bannerman^3^

^1^Department of Obstetrics, Gynecology and Reproductive Sciences, University of California San Diego, San Diego, CA; ^2^Department of Obstetrics & Gynecology  , University of Washington, Seattle, WA, Seattle, WA; ^3^Division of Maternal Fetal Medicine, Department of Obstetrics, Gynecology and Reproductive Sciences, University of California San Diego, San Diego, CA

**Objective**: Marginalized groups experience discrimination and are known to have adverse maternal mental health outcomes. We examined the relationship between self‐reported discrimination and postpartum mental health with a focus on anxiety.

**Study Design**: This is a secondary analysis of a prospective study of labor agentry. Patients were recruited from a single MFM practice from 27–34 weeks’ gestation.  At enrollment, participants completed the Everyday Discrimination Scale (EDS), a validated survey regarding daily discrimination experience. At 6 weeks postpartum, participants completed validated mental health inventories including Generalized Anxiety Disorder‐7 (GAD‐7), Edinburgh Postnatal Depression Scale (EPDS), and City Birth Trauma Scale (CBTS) to assess for symptoms of anxiety, depression, and birth trauma respectively. We compared mental health outcomes stratified by any or no reported discrimination on the EDS. Adjusted logistic regression evaluated the relationship between EDS >1 and positive GAD‐7 defined as a total score >5.

**Results**: Of 119 participants, 47 (41%) reported no discrimination, while 69 (59%) reported experiencing discrimination. Participants who spoke a language other than English at home were less likely to report experiencing discrimination (8.7% v. 27.7%, *p* = 0.014) and participants with a history of mental health disorder were more likely to report experiencing discrimination (63.8% vs 32.6%, *p* = 0.039) (Table 1). Race and ethnicity were not associated with reporting discrimination. There was no difference in rate of positive GAD‐7, EPDS, and CBTS between groups (Table 1). Unadjusted analysis showed no association between a positive GAD‐7 and reported discrimination (OR 2.155, 95% CI 0.940–4.939), and this result persisted after adjusting for language and mental health diagnosis (aOR 1.539, 95% CI 0.611–3.876).

**Conclusion**: Participants who experienced discrimination were almost twice as likely to experience postpartum anxiety, but our small sample size limited our ability to reach significance. Larger studies are needed to evaluate the impact of discrimination on postpartum mental health.

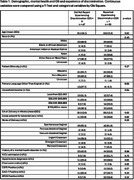

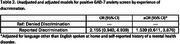

**3:30 PM – 5:00 PM**

## 1077 | **Does Ultrasound Contribute to Clinical Management of Women With Subclinical Post Partum Hemorrhage–Prospective Cohort Study**

Enav Yefet^1^; Yuri Perlitz^2^; Inbal Melamed Teretz^2^; Sami Haddad^2^; Batsheva Yaloz^2^; Samar Mari^2^

^1^Tzafon medical center, Poriya, HaZafon; ^2^Tzafon Medical Center, Poriya, HaZafon

**Objective**: Postpartum Hemorrhage (PPH) is the third leading cause for maternal death in the USA. It was demonstrated that hemoglobin (Hb) drop≥2g/dL was associated with PPH. Yet, many cases are subclinical, since overt hemorrhage did not precede the drop in Hb. The utility of ultrasound (US) in the management of the subclinical cases is not clear.

We aimed to elucidate whether US may contribute to the management of women with subclinical PPH, i.e. at least 2g Hb drop from baseline without reporting on overt bleeding.

**Study Design**: Prospective cohort trial. In our department all women perform Hb test before and several hours after delivery. Women after vaginal delivery (normal or with vacuum) without documentation of abnormal bleeding were divided, according to their postpartum Hb drop from baseline, to subclinical PPH group (Hb drop≥2 g/dL) and to a control group (Hb drop<2 g/dL) in a ratio of 1:2. Women who performed US right after delivery were excluded. The primary outcome was the rate of women with a pathological finding on US examination (abdominal or vaginal). Assuming that the rate of pathological findings in the US examination will be 30% of the women in the subclinical PPH group compared to 10% of the women in the control group, the required sample size was 44 and 88 women in the subclinical PPH and control group, respectively (5% 2‐sided alpha, 80% power).

**Results**: Characteristics and outcomes are presented in tables 1 and 2. The rate of pathological findings on US was 1 (2%) and 3 (3%) of women in the subclinical PPH and control groups, respectively (*p* = 1). US led to intervention (misoprostol and later on hysteroscopy for placental tissue removal) only in one (1%) woman in the control group (table).  The rest of the women reported on normal US examination in the 6‐weeks post‐partum visit.

**Conclusion**: In subclinical PPH, US did not contribute to the clinical management of those women.

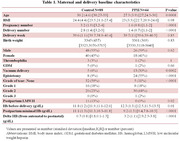

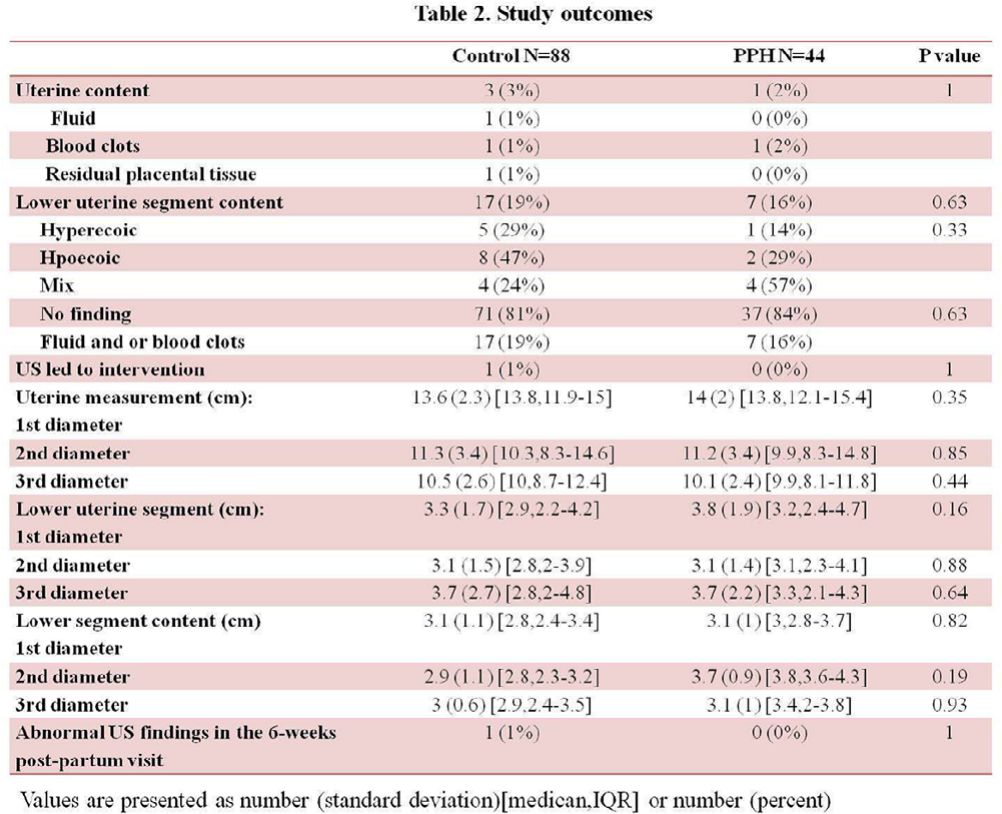

**3:30 PM – 5:00 PM**

## 1078 | **Predictors of Takotsubo Syndrome During Pregnancy and The Puerperium in the United States**

Eric K. Broni^1^; Amponsah Peprah^2^; Lisa D. Levine^3^; Imo Ebong^4^; Chike C. Nwabuo^5^; Catherine Kim^6^; Duke Appiah^7^

^1^Yale University, New Haven, CT; ^2^Kwame Nkrumah University of Science & Technology, Kumasi, Ashanti; ^3^Perelman School of Medicine, University of Pennsylvania, Philadelphia, PA; ^4^Division of Cardiovascular Medicine, UC Davis, Sacramento, CA; ^5^University of Colorado School of Medicine, Aurora, CO; ^6^University of Michigan, Ann Abor, MI; ^7^Texas Tech University Health Sciences Center, Lubbock, TX

**Objective**: Takotsubo syndrome (TS) or broken heart syndrome, is a rare cardiac condition with comparable mortality to myocardial infarction. Although few case reports highlight TS, population‐based data on TS in pregnancy are scarce. We examined the epidemiology of TS during pregnancy and the puerperium in the U.S.

**Study Design**: We analyzed 4.1 million pregnancy‐related hospitalizations in the National Inpatient Sample (NIS) from 2015 to 2021. ICD codes were used to define TS and other clinical conditions. Logistic regression models were used to calculate odds ratios (OR) and 95% confidence intervals (CI), and receiver operating characteristic (ROC) curve analysis was used to evaluate the predictors of TS. The identified parsimonious model was internally validated using 4.8 million records from the 2010–2015 NIS.

**Results**: The prevalence of TS increased from 2015 to 2021 (1.96 to 3.85 per 100,000) Figure A. Patients with TS were older (30.6 years vs 29.1 years; *p* = 0.003), used tobacco (12.9% vs 5.5%; *p* = 0.016) and had dyslipidemia (4.8% vs 0.4%; *p* = 0.023). Additionally, TS patients were more likely to have eclampsia (8.9% vs 0.1%; *p* = 0.001), postpartum hemorrhage (16.1% vs 3.9%, *p* = 0.001), anxiety disorders (17.7% vs 5.8%; *p* = 0.001), major cardiovascular events including postpartum cardiomyopathy (25.8% vs 0.1%, *p* = 0.026), in‐hospital mortality (4.0% vs 0.0%; *p* < 0.001) and longer hospital stay (9.6 vs 2.6 days; *p* = 0.026) Table. In adjusted models, predictors of TS were coronary artery disease, eclampsia, sepsis, molar pregnancy, induced abortion, postpartum hemorrhage, fetal birth defect, stillbirth, acute stress reactions and anxiety disorder. Figure B. The internally validated model had an area under the ROC curve (AUC) of 0.82 (CI:0.75–0.88).

**Conclusion**: Several clinical and obstetrical factors were predictive of TS in pregnancy and the puerperium. Future studies evaluating the mechanistic pathways driving these relations may provide a more accurate risk assessment and reduce the incidence of TS and related complications during pregnancy and the puerperium.

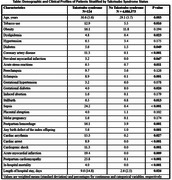

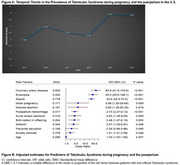

**3:30 PM – 5:00 PM**

## 1079 | **External Influences on Clinicians' Decision‐Making During Second Stage Cesarean Delivery: A Grounded Dimensional Analysis**

Erica Marion^1^; Sarah T. McMahon^1^; Janayah McClellan^1^; Anna Palatnik^2^; Melissa A. Clark^3^; Brock E. Polnaszek^1^

^1^Medical College of Wisconsin, Milwaukee, WI; ^2^Medical College of Wisconsin ‐ Milwaukee, WI, Milwaukee, WI; ^3^Brown University School of Public Health, Providence, RI

**Objective**: Second stage cesarean delivery demands rapid, critical decision‐making in complex clinical environments. This study explored external factors that influence clinicians' decision‐making in second stage cesarean delivery.

**Study Design**: Semi‐structured interviews were conducted with clinicians who opted in after completing a nationally distributed survey. Interviews inquired about labor and delivery structure, resource availability, delivery techniques, and real‐world management implementation. Interviews were recorded, transcribed verbatim, and analyzed using grounded dimensional analysis (GDA). GDA identifies variation in real‐world processes to generate a conceptual model explaining key dimensions of clinical decision‐making, such as second stage cesarean delivery management.

**Results**: A total of 41 clinicians participated and were equally represented by the four main geographic regions in the United States. Most participants were attending physicians specializing in general obstetrics and gynecology or maternal fetal medicine. Four key domains emerged that shaped clinician decision‐making during second‐stage cesarean delivery: 1) System‐level pressures (e.g., liability, institutional policy, cost‐containment), 2) Patient‐specific factors (e.g., body habitus, fetal position, urgency), 3) Peer and cultural norms (e.g., departmental expectations, informal mentorship), and Resource and real‐time constraints (e.g., personnel, equipment, OR readiness) (Figure 1). Representative quotes supporting each domain are presented in Table 1.

**Conclusion**: Second stage cesarean delivery decisions are not made in isolation. Rather, clinicians operate within a web of interrelated external influences that shape their actions in real time. The conceptual framework developed from this analysis may guide the design of standardized protocols, targeted team‐based training, and system‐level interventions to improve the safety, efficiency, and consistency of care in second stage cesarean delivery.

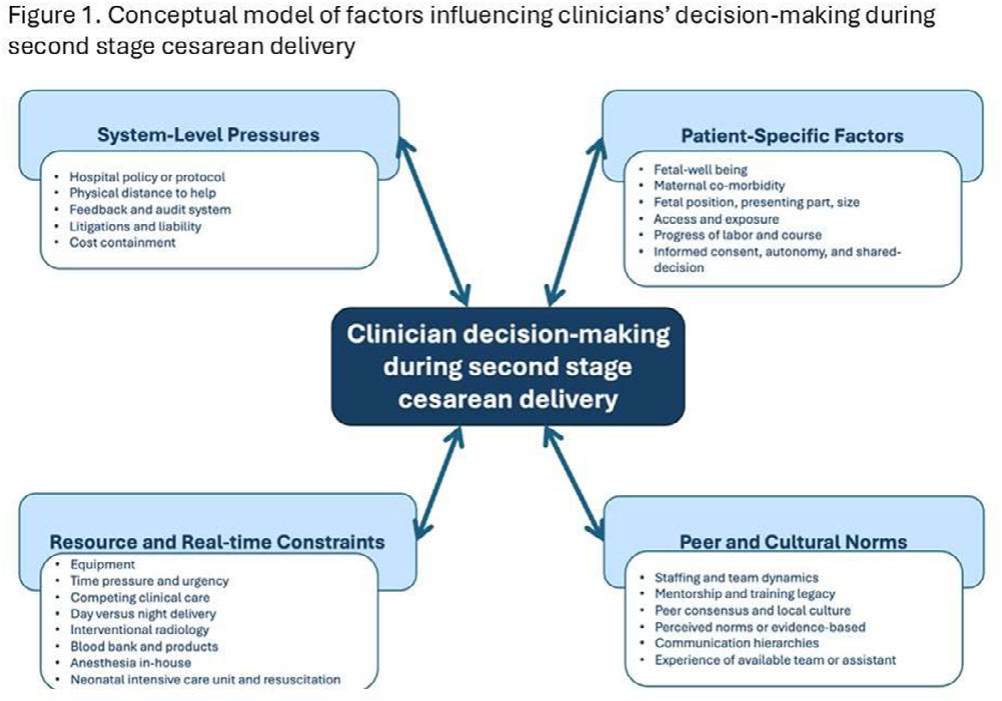

**3:30 PM – 5:00 PM**

## 1080 | **Can Early Pregnancy Glucose Predict Gestational Diabetes and Large‐for‐Gestational Age Neonates?**

Erika F. Werner^1^; Denise Scholtens^2^; Alan Kuang^3^; Patrick M. Catalano^4^; Francesca Facco^5^; Maisa Feghali^6^; William Grobman^7^; Erin S. LeBlanc^8^; Audrey Merriam^9^; Mirella Mourad^10^; Caryn E.S. Oshiro^11^; Camille E. Powe^12^; Uma M. Reddy^13^; Dwight J. Rouse^14^; Jennifer Sherr^15^; Alexandra C. Spadola^1^; Kimberly K. Vesco^8^; Lynn M. Yee^2^; Noelia Zork^13^; William L. Lowe^16^

^1^Tufts University School of Medicine, Boston, MA; ^2^Northwestern University Feinberg School of Medicine, Chicago, IL; ^3^Northwestern University, Chicago, IL; ^4^MGH REU, Boston, MA; ^5^UPMC, UPMC, PA; ^6^Magee Women's Research Institute, University of Pittsburgh, Pittsburgh, PA; ^7^Women & Infants Hospital/ Brown University, Providence, RI; ^8^Kaiser Permanente Center for Health Research, Portland, OR; ^9^Yale School of Medicine, Yale School of Medicine, CT; ^10^Division of Maternal‐Fetal Medicine, Department of Obstetrics and Gynecology, Columbia University Irving Medical Center, New York, NY; ^11^Kaiser Permanente Hawaii, Center for Integrated Health Care Research, Honolulu, HI; ^12^Massachusetts General Hospital, Boston, MA; ^13^Columbia University Irving Medical Center, New York, NY; ^14^Women & Infants Hospital of Rhode Island / Warren Alpert Medical School of Brown University, Providence, RI; ^15^Yale School of Medicine, New Haven, CT; ^16^Northwestern University, Northwestern University, IL

**Objective**: To evaluate whether data from early pregnancy oral glucose tolerance testing (OGTT) or continuous glucose monitoring (CGM) improves prediction of gestational diabetes (GDM) or large for gestational age (LGA) birthweight compared to clinical risk factors alone.

**Study Design**: In a prospective observational study at 9 US centers, adults with a singleton pregnancy and no pre‐existing diabetes underwent 75‐g OGTT and CGM placement at 10–14 wks’ gestation. Co‐primary outcomes were GDM at 24–28 wks’ gestation (Carpenter‐Coustan criteria) and LGA (birthweight > 90%). We evaluated a wide range of criteria for the 10–14 wk OGTT and CGM to develop optimal models for GDM and LGA prediction in combination with clinical factors (age, BMI, history of GDM or macrosomia). Glycemic criteria were selected to maximize model area under the ROC curve (AUC) in a training dataset from 4 centers, with data from 5 centers reserved for validation. To evaluate models incorporating OGTT or CGM + clinical factors v. clinical factors alone for identifying high‐risk individuals who may benefit from early intervention, we compared PPV at a prespecified NPV (> 1‐ prevalence/2), with prevalence set at 8.3% for GDM and 10% for LGA based on national data.

**Results**: Of 2178 participants enrolled from 2021–2024, 90.3% completed OGTTs at 24–28 wks and 95.9% had live births; 15.4% had GDM and 11.3% had LGA infants. ROC curves for optimal OGTT, CGM, and clinical factor models are shown (Figure). For GDM, both glycemic models (10–14 wk OGTT or CGM + clinical factors) improved PPV compared to clinical factors alone: at prevalence = 8.3% and NPV = > 96%, PPV was 12.2% for clinical factors, 26.5% for OGTT, and 22.5% for CGM (Table).  For LGA, neither glycemic model improved PPV compared to clinical factors alone. Validation confirmed findings.

**Conclusion**: Although adding data from 10–14 wk OGTT or CGM to clinical factors improved GDM prediction, the PPV was modest and their addition had no value for LGA prediction.  These results do not support using early pregnancy OGTT or CGM to identify individuals at high risk for subsequent GDM or LGA.

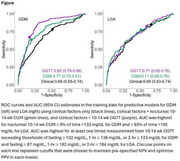

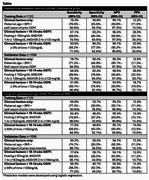

**3:30 PM – 5:00 PM**

## 1081 | **Pulsenmore Self‐Scan Proven Safe and Effective in HOLA (Home Ultrasound Assessment)–USA‐Based Multicenter Study**

Ronit Shtrichman^1^; Erkan Kalafat^2^; Lawrence D. Platt^3^; Sara Little^4^; Paola Aghajanian^5^; Shirley Platt^5^; Kris Ann Tychon^6^; Nicola F. Tavella^7^; Angela T. Bianco^8^

^1^Pulsenmore, Boston, MA; ^2^Koc University Hospital, Istanbul, Koc University Hospital, Istanbul, Istanbul; ^3^UCLA David Geffen School of Medicine and Cedars‐Sinai Medical Center, Los Angeles, CA; ^4^Maternal‐Fetal Medicine, Beth Israel Deaconess Medical Center, 4. Maternal‐Fetal Medicine, Beth Israel Deaconess Medical Center, Boston, MA; ^5^UCLA David Geffen School of Medicine, Cedars‐Sinai Medical Center, UCLA David Geffen School of Medicine, Cedars‐Sinai Medical Center, CA; ^6^Department of Maternal Fetal Medicine, Brigham and Women's Hospital, Boston, 5. Department of Maternal Fetal Medicine, Brigham and Women's Hospital, Boston, MA; ^7^Division of Maternal‐Fetal Medicine, Icahn School of Medicine, Icahn School of Medicine at Mount Sinai Hospital, New York, NY; ^8^Icahn School of Medicine at Mount Sinai, New York, NY

**Objective**: Telehealth is reshaping prenatal care by improving patient satisfaction, reducing costs, while maintaining safe clinical outcomes. Pulsenmore is a handheld ultrasound device connected to a smartphone via a mobile app, enabling pregnant women to perform scans at home (Figure 1). Scans are securely interpreted by clinicians, supporting a hybrid care model.

**Study Design**: This prospective, multicenter study evaluated the safety and efficacy of the Pulsenmore self‐operated ultrasound platform. Pregnant women (≥14 weeks) were enrolled across four U.S. centers and completed three weekly sessions. Scan types included: **App‐Guided (AG)**–self performed at home via video tutorial, **Clinician‐Guided (CG)**–home scan during a telehealth visit, and **In‐Clinic (IC)**–standard ultrasound serving as ground truth. AG and CG scans were reviewed by 10 experts assessing image quality (ACEP grading), fetal cardiac activity (FCA), amniotic fluid volume (AFV), fetal movement, breathing, and placental location.  Safety and ease of use were also evaluated.

**Results**: A total of 1370 scans (458 AG, 453 CG, 458 IC) from 162 pregnancies were analyzed. No device‐ or procedure‐related serious adverse events occurred. FCA was diagnosed in 98.5% (CG) and 95.2% (AG) of scans by ≥ 8 readers, with excellent inter‐reader reliability (Gwet's AC2: 0.805 CG, 0.846 AG).  FCA assessment success rate exceeded 95% across all visits (Figure 2). Sensitivity analyses showed no significant impact of gestational ages, BMI, or education levels on FCA assessment (*p* > 0.05). AFV, placental location, fetal movement and breathing (GW >27) assessments were comparable between Pulsenmore and IC scans. 83% of patients reported a positive experience; 93% found the device easy to use.

**Conclusion**: Pulsenmore demonstrates strong clinical evidence for safety, efficacy and usability in home‐based fetal well‐being monitoring.

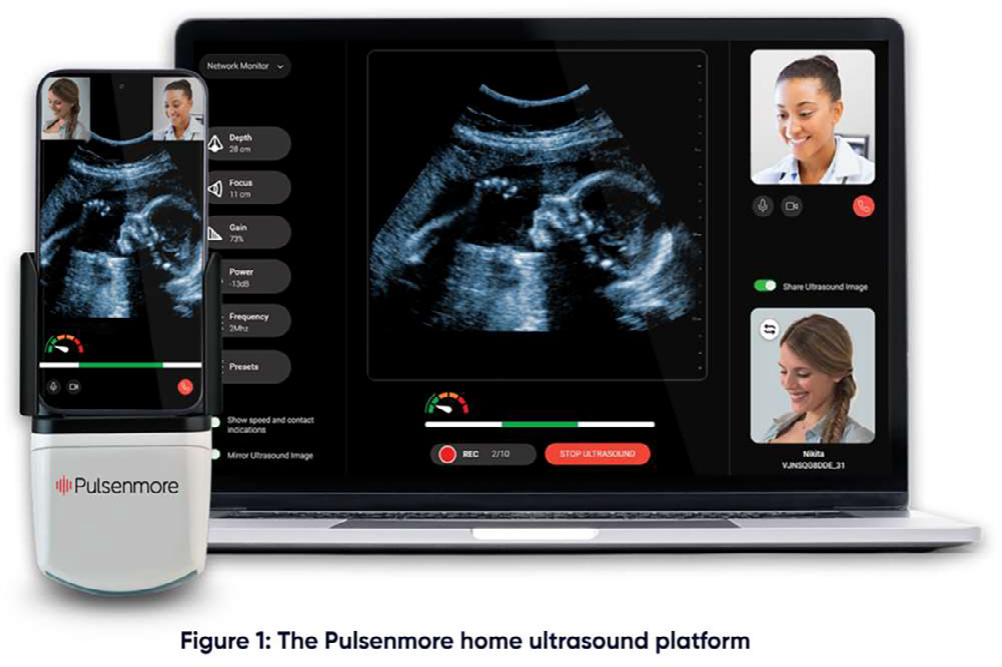

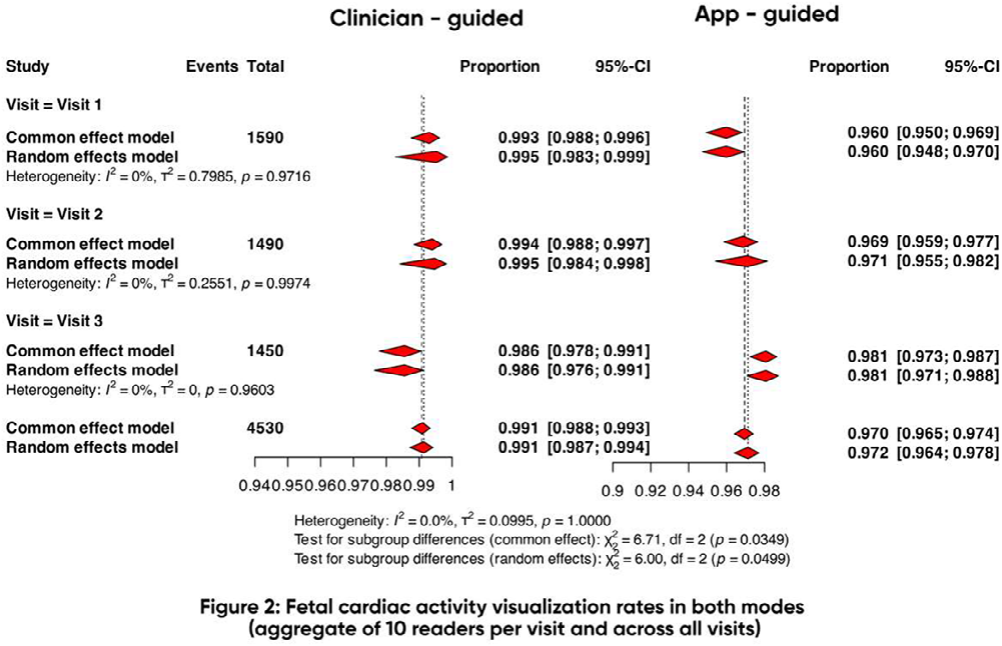

**3:30 PM – 5:00 PM**

## 1082 | **A Novel Evaluation Method for LLM Contextual Prompting in a Virtual Maternity Setting**

Robert Martorano^1^; Kathryn G. Fisher^1^; Samuel Weiss^1^; Rachelle York^1^; Madeline Perry^2^; Stacey Kallem^1^; Eva M. Luo^1^; Ian J. Hooley^1^

^1^Pomelo Care, Pomelo Care, NY; ^2^University of Pennsylvania, University of Pennsylvania, PA

**Objective**: The safety and efficacy of generative AI in clinical settings are critically dependent on the context provided to the model. Our goal was to develop and validate an evaluation framework to de‐risk and improve the quality of an AI clinical co‐pilot by simulating real‐world conditions and comparing the impact of different contextual “prompting” strategies on the quality of AI‐generated patient communications.

**Study Design**: We selected 69 historical patient conversations on preeclampsia risk, labor, and pediatric respiratory concerns. Each conversation was truncated at a key clinical turn. An LLM then generated seven responses using varied strategies, from a base model with no context to prompts using published guidelines (e.g., ACOG), conversation‐derived protocols, or retrieval‐augmented generation (RAG) with institutional protocols combined with published guidelines. Three blinded, board‐certified clinicians reviewed all 483 responses, rating each as “Best,” “Acceptable,” or “Unacceptable” (defined as clinically unsafe).

**Results**: Analysis of 490 responses revealed context was a critical determinant of safety (Table 1). Strategies grounded in curated, institutional protocols performed best, yielding the lowest ‘Unacceptable’ rate (16%) and highest ‘Acceptable’ rate (49%). In contrast, the base model produced the most ‘Unacceptable’ responses (28%). Notably, AI‐derived protocols from historical conversations alone also performed poorly (‘Unacceptable’ rate of 23%). Qualitative clinician feedback captured that unacceptable responses often failed to recognize clinical nuance or escalation needs, such as for symptoms of preeclampsia.

**Conclusion**: Clinical AI safety and efficacy are a product of rigorous context engineering, not the model alone. This framework effectively validates safe prompting strategies before live deployment. Grounding generative AI in curated, institution‐specific protocols is essential to minimize risk and is significantly safer than relying on unstructured historical conversations alone, ensuring high‐quality patient communications.

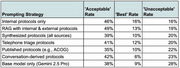

**3:30 PM – 5:00 PM**

## 1083 | **Needle Lasso Technique for Port Site Closure During Fetoscopic Spina Bifida Repair**

Farokh Demehri^1^; Claudio V. Schenone^2^; Weston Northam^3^; Alireza A. Shamshirsaz^2^; Eyal Krispin^4^

^1^Boston Children's Hospital, Bonston Children's Hospital/ Boston, MA; ^2^Boston Children's Hospital, Harvard Medical School, Boston, MA; ^3^Boston Children's Hospital, boston, MA; ^4^Boston Children's Hospital, Harvard Medical School, Bonston Children's Hospital/ Boston, MA

**Objective**: To describe the first clinical application of a needle lasso technique adapted from pediatric inguinal hernia repair (PIRS) for port site closure during fetoscopic spina bifida repair, aimed at reducing PPROM and chorioamniotic separation.

**Study Design**: We implemented the needle lasso technique in three consecutive cases undergoing fetoscopic spina bifida repair at our center—two via laparotomy‐assisted and one via mini‐laparotomy approach. Following fetoscopic access using two 3‐mm and one 5‐mm uterine cannulas, 3‐0 Prolene loop sutures mounted on 18G spinal needles were inserted transmyometrially to encircle each port site. A 3‐0 PDS suture was passed via a 20G needle on the opposite side and retrieved through the loop to form a full‐thickness port‐site stitch, anchoring the membranes to the uterine wall [Figure 1]. Each port was closed with 3–4 stitches forming triangular or square plications.

**Results**: All three pregnancies were prolonged beyond 34 weeks with no incidence of PPROM or chorioamniotic separation. The technique allowed for real‐time visualization and precise closure of the port sites, with visible approximation of the membranes to the uterine wall [Figure 2]. No maternal or fetal complications were observed.

**Conclusion**: The needle lasso technique offers a simple, effective method for port site closure during fetoscopic spina bifida repair. Adapted from a proven pediatric surgical technique, it provides membrane plication under direct visualization and may help mitigate postoperative complications such as PPROM. Integration with emerging magnetic or mechanical suture‐passing devices may further improve its reproducibility and ease of use in minimally invasive fetal surgery.

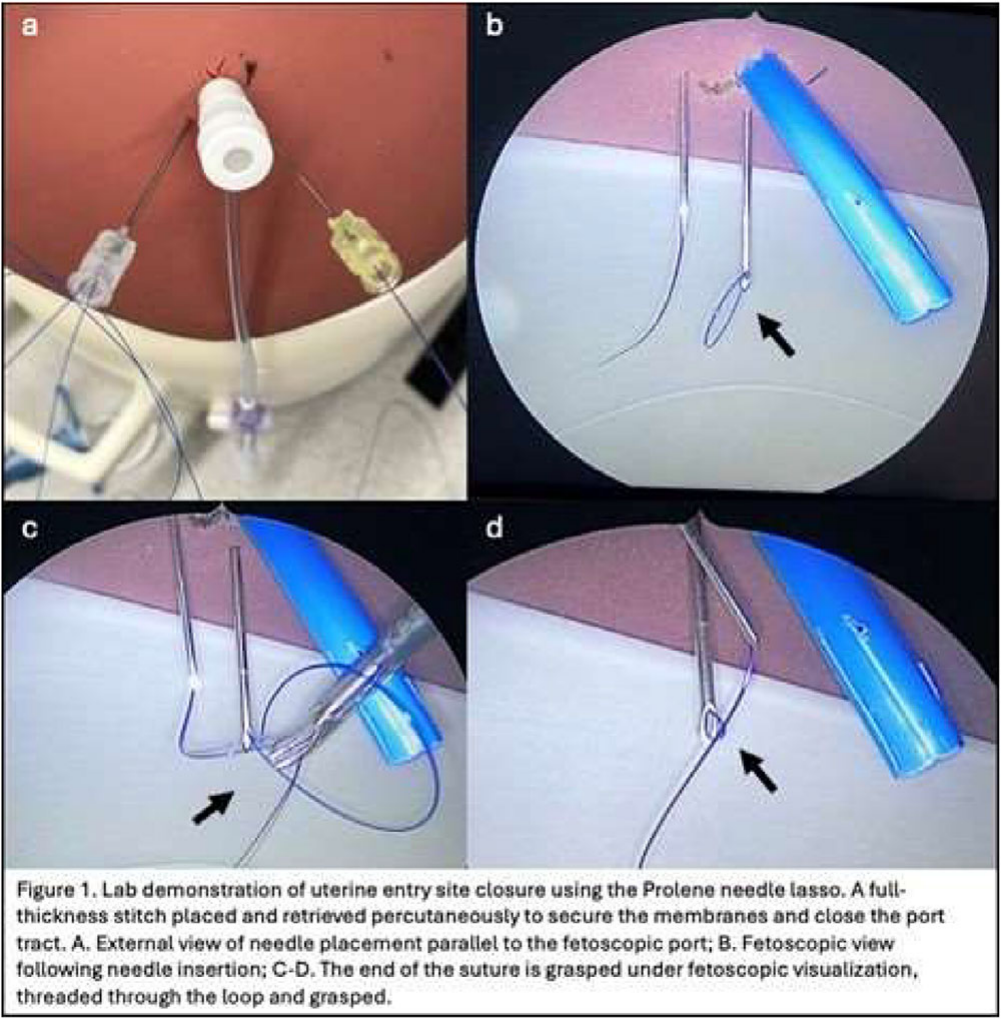

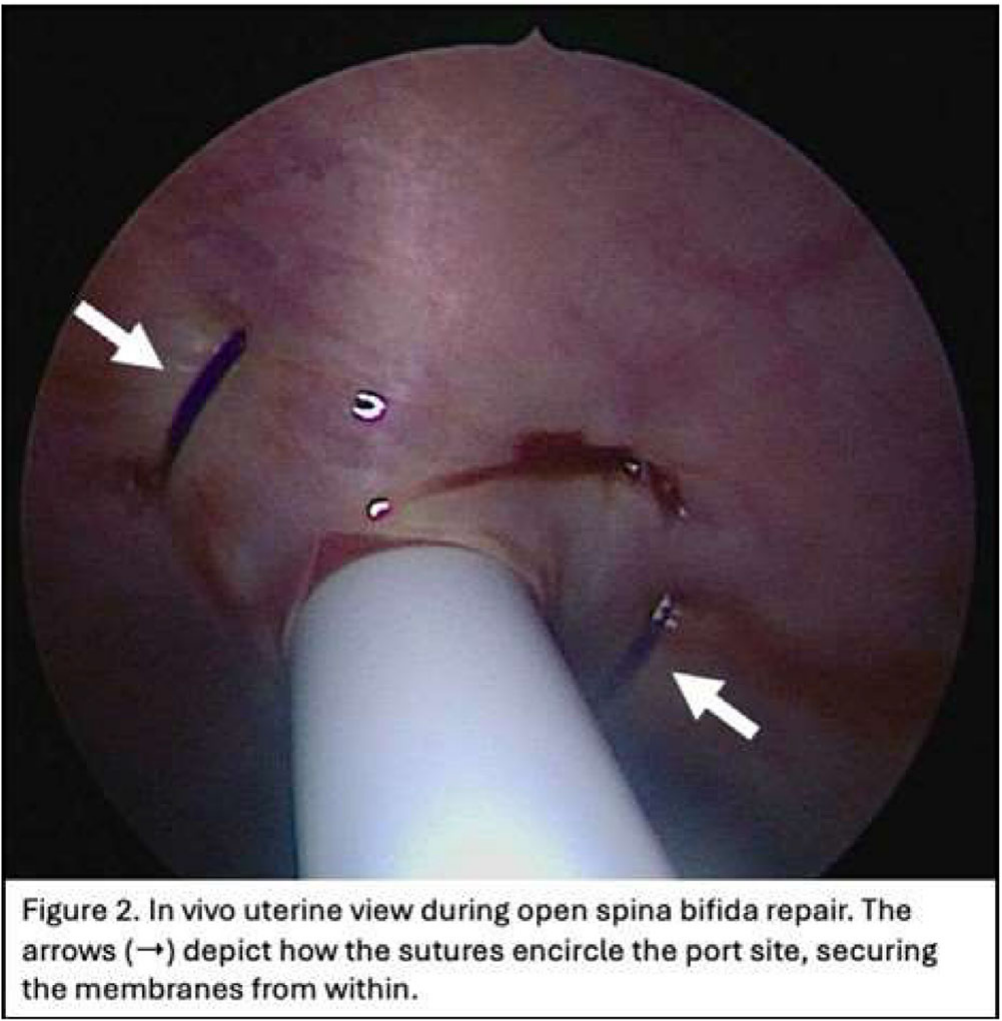

**3:30 PM – 5:00 PM**

## 1084 | **Physician Workload: A Comparative Analysis Across Maternal Fetal Medicine Ultrasound Practice Settings**

Fadi Bsat^1^; Suwan Mehra^2^

^1^SMFM Practice Management Committee, West Springfield, MA; ^2^Advocate Children's Hospital, Advocate Childrens Hospital, IL

**Objective**: To determine the median number of ultrasound examinations scheduled per Maternal Fetal Medicine (MFM) physician during an 8‐hour workday, stratified by practice ownership and size.

**Study Design**: A web‐based survey was emailed to all 2096 regular members of the Society for Maternal Fetal Medicine (SMFM) in the United States. Respondents were asked for the number of ultrasound examinations interpreted per MFM physician in an 8‐hour workday, under the assumption that the interpreting physician has no other clinical responsibilities and reviews a typical mix of studies (including fetal anatomy, fetal echocardiography, biophysical profiles, first trimester, limited and follow‐up scans). A total of 232 responses were included in the final analysis. Practices were categorized by practice ownership (university/medical school, health system, hospital with academic affiliation, community hospital, private) and by size, based on the number of clinical full‐time equivalent (cFTE) MFM physicians (large: ≥ 5 cFTE; small: <5 cFTE). Pearson's Chi‐squared test with simulated p‐value was used for statistical analysis.

**Results**: University/medical school‐owned practices reported a significantly higher median volume of 30–39 ultrasound studies interpreted per MFM physician per 8‐hour shift compared to all other practice types, which reported a median of 20–29 studies (*p* = 0.001). Furthermore, practices with ≥ 5 cFTE MFM physicians scheduled more studies per physician than smaller practices, a difference that approached statistical significance (*p* = 0.055).

**Conclusion**: Ultrasound interpretation workload among MFM physicians varies between practice settings. University/medical school‐affiliated and larger practices demonstrate a higher number of scheduled studies per physician. This survey provides important benchmarks to support workforce planning, optimize clinical efficiency, and guide resource allocation in MFM ultrasound units.

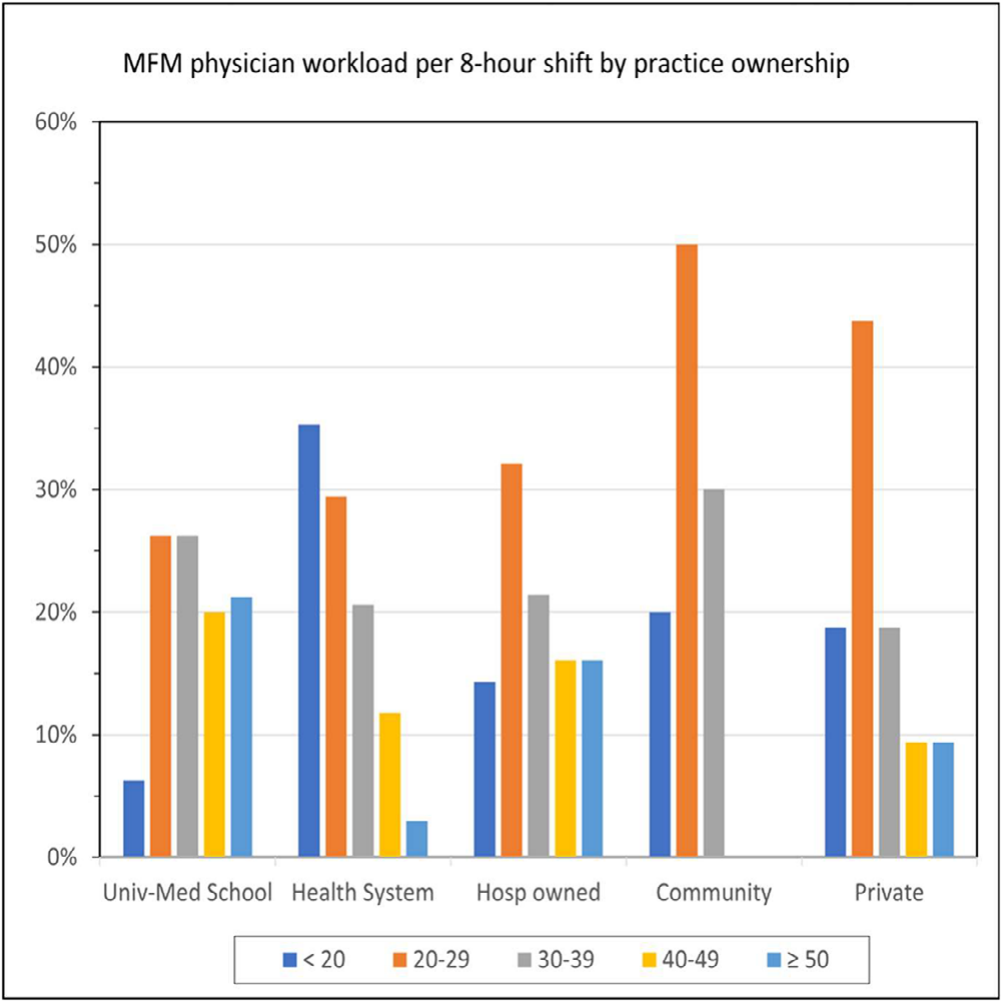

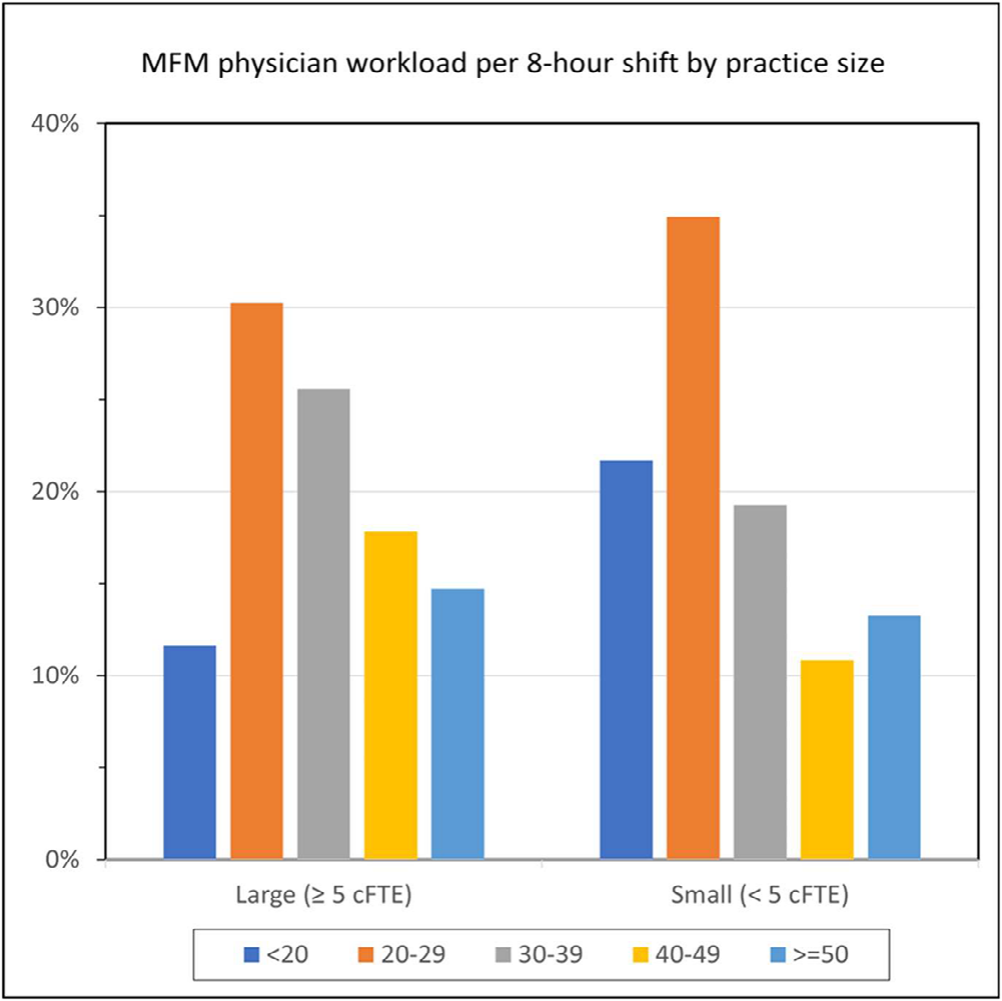

**3:30 PM – 5:00 PM**

## 1085 | **Risk Factors for PPROM after Fetoscopic Placental Laser Ablation for Twin‐Twin Transfusion Syndrome**

Felicia V. LeMoine^1^; Dejian Lai^2^; Sami Backley^1^; Jimmy Espinoza^3^; Anthony Johnson^3^; Ramesha Papanna^4^

^1^Division of Fetal Intervention, Department of Obstetrics, Gynecology and Reproductive Sciences, McGovern Medical School at UTHealth Houston, Houston, TX; ^2^Department of Biostatistics, School of Public Health at UTHealth Houston, Houston, TX; ^3^UTHealth Houston Fetal Institute; Division of Fetal Intervention, Department of Obstetrics, Gynecology and Reproductive Sciences, McGovern Medical School at UTHealth Houston, Houston, TX; ^4^University of Texas Health Science Center in Houston, McGovern Medical School, Houston, TX

**Objective**: Preterm prelabor rupture of membranes (PPROM) is a frequent complication after fetoscopic placental laser ablation (PLA) for twin‐twin transfusion syndrome (TTTS). Our center previously found no risk factors for PPROM after PLA. Here, we leveraged substantial clinical experience and the dataset of a high‐volume TTTS referral center to update these findings.

**Study Design**: A secondary analysis of a prospective cohort study was conducted on 983 PLA cases performed at our fetal center (2011–2025). Only cases of TTTS were included. Triplet or higher order gestations, monoamniotic twins, terminations, and cases with missing outcome data were excluded. Maternal and perioperative factors were compared between groups. Statistical tests included two‐sample *t* tests, χ2 test, and Fisher's exact test, where appropriate. Stepwise multiple logistic regression was used to identify clinical factors associated with PPROM.

**Results**: Of 843 cases, PPROM complicated 339 (40.2%). The median gestational age (GA) at PPROM was 190 (161, 217) days. Maternal characteristics did not differ between groups. The PPROM group had an earlier GA at PLA (138 [126,151] versus 144 [131, 157] days, *p* < 0.001), a lower rate of amnioreduction (AR; 85.3% versus 90.8%, *p* = 0.016), and a higher rate of chorion‐amnion separation (CAS; 17.4% versus 6.5%, *p* < 0.001) than the non‐PPROM group. The PPROM group delivered earlier, had higher rates of chorioamnionitis and placental abruption, and had lower rates of dual neonatal survival than the non‐PPROM group (Table 1). Stepwise logistic regression yielded a final predictive model that included Hispanic ethnicity, trocar size and entry type, laser energy, AR, cervical length (CL), donor maximum vertical pocket (D MVP), GA at PLA, total anastomoses, and CAS, with significant associations observed for CL, D MVP, GA at PLA, total anastomoses, and CAS (Table 2).

**Conclusion**: PPROM complicates 40% of fetoscopic PLA cases for TTTS and contributes to perinatal morbidity. Key risk factors include CL, D MVP, GA at PLA, total anastomoses, and CAS. Underlying mechanisms warrant further investigation.

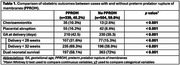

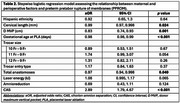

**3:30 PM – 5:00 PM**

## 1086 | **Beyond the Cesarean Rate: A Provider‐Level Composite Metric for Perinatal Quality Assessment**

Frank I. Jackson; Oladunni Ogundipe; Sarah H. Abelman; Luis A. Bracero; Matthew J. Blitz

Northwell, New Hyde Park, NY

**Objective**: To evaluate obstetrician‐level variation using a unified composite outcome, complication‐free vaginal delivery (CFVD), defined as vaginal delivery without maternal or neonatal complications, compare CFVD‐based to cesarean delivery (CD)‐based rankings, and assess correlations among CD, maternal, and neonatal complication rates among nulliparous, term, singleton, vertex (NTSV) births.

**Study Design**: This retrospective cross‐sectional study included all NTSV deliveries by obstetricians with ≥50 deliveries across 7 hospitals from January 2019 to December 2024. Maternal and neonatal complications were defined using Joint Commission criteria. For each provider, CFVD and CD rates were calculated, and rankings compared.

**Results**: Among 55,841 NTSV deliveries, 233 obstetricians met inclusion. Rankings based on CFVD showed that 108 of 233 (46.4%) shifted by >10 ranks compared to CD‐based rankings (Kendall Tau = 0.836, *p* <  .0001). Correlations showed a weak negative association between CD and neonatal complication rates (r = −0.14, *p* = 0.04), a weak positive correlation between CD and maternal complications (r = 0.14, *p* = 0.03), and a weak positive correlation between maternal and neonatal complications (r = 0.14, *p* = 0.03).

**Conclusion**: The current approach of emphasizing lowering the cesarean delivery rate, fails to account for maternal and neonatal complications and treats their outcomes independently, despite clinical interdependence. This may lead to misleading assessments of provider performance. Provider‐level assessment using a composite dyadic outcome offers a more complete view of perinatal quality. Incorporating CFVD into quality metrics may improve benchmarking and better reflect patient‐centered care.

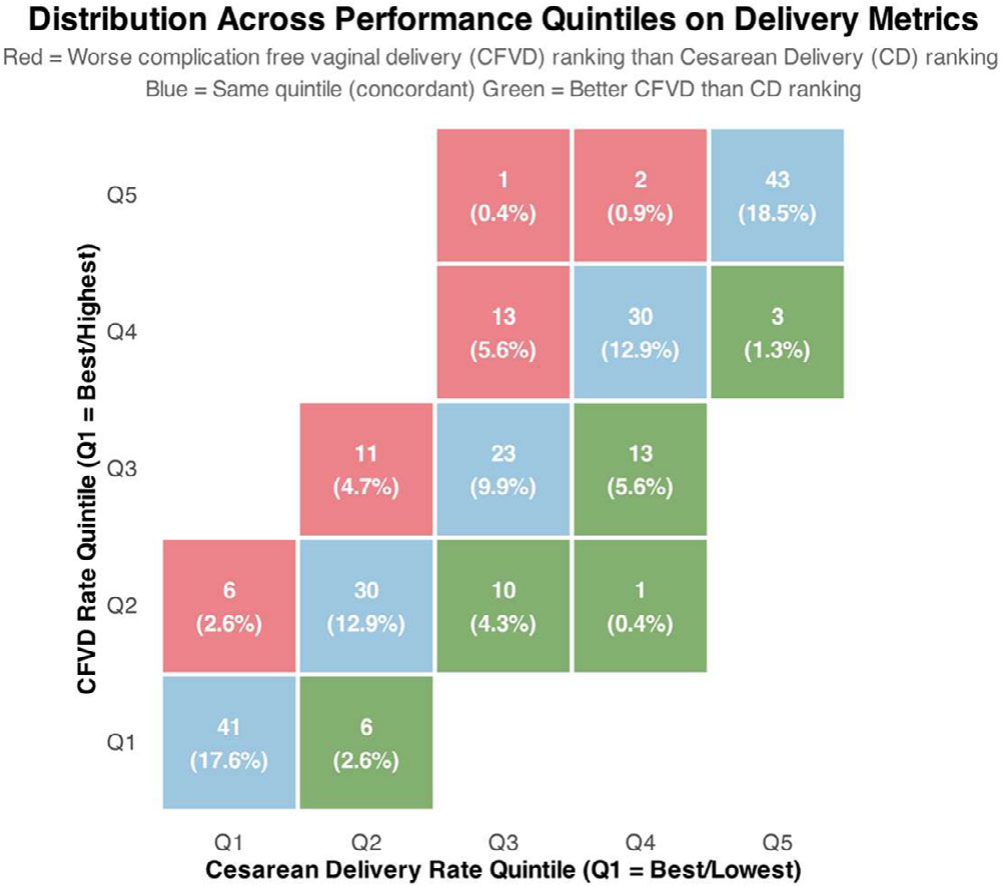

**3:30 PM – 5:00 PM**

## 1087 | **Weight Gained or Never Lost: The Relationship Between Pregnancy Weight Trajectory and Lifelong Cardiometabolic Risk**

Gabriella Braun; Mary Falstin; Bryan L. Aaron; Levi J. Anderson; Adam Baruch; Samantha Schon; Lindsay Admon; Ashley M. Hesson

University of Michigan, Ann Arbor, MI

**Objective**: As weight management options proliferate, it is crucial to understand the time course and morbidity of intra‐ and inter‐pregnancy weight fluctuation for effective intervention. We aim to characterize the relationship between weight gain during pregnancy, weight retention after pregnancy, and the development of chronic cardiometabolic conditions (CC) with and without precedent adverse pregnancy outcomes (APO).

**Study Design**: This single‐site retrospective cohort study followed the BMI trajectories of individuals with first singleton births occurring from 2014–2015, no subsequent multiple gestations, and recent intra‐institutional follow‐up. Linear models were fitted to the BMI course of each participant, both within a pregnancy and from their initial weight at first pregnancy to last recorded weight. APOs and CCs that developed after the qualifying pregnancy, and demographics, were abstracted; CC development was modeled with respect to BMI trajectories, initial pregnancy BMIs, APO occurrence, and age.

**Results**: A total of 525 patients (998 pregnancies) were analyzed (Table 1). Mean initial BMI was 29.1kg/m^2^±7.1 for qualifying pregnancies and 29.41kg/m^2^±6.8 for additional pregnancies. Initial BMI was highly correlated with APO occurrence (*p* < 0.01) and the development of multiple CCs was associated with higher initial BMI (Figure 1). Having an APO or an initial BMI≥30.1kg/m^2^ range in any pregnancy was associated with earlier age‐adjusted CC development (*p* < 0.01, Figure 1). CC development was significantly predicted by increasing initial BMI (*p* = 0.04) and age (*p* = 0.03), but not intra‐pregnancy weight slope (*p* = 0.19) or total follow‐up weight slope (*p* = 0.80).

**Conclusion**: Initial pregnancy weight, as opposed to BMI trajectory within a pregnancy or in longitudinal follow‐up, predicted the development of CCs such as diabetes and cardiovascular disease. Adverse pregnancy outcomes, though closely related to BMI, independently predict these events. Optimizing prepregnancy BMI and preventing post‐gestational weight retention should be a priority in preventing CCs in women.

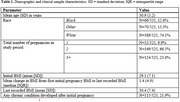

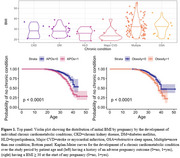

**3:30 PM – 5:00 PM**

## 1088 | **Decision‐to‐Delivery Time in Emergency Repeat Cesarean Deliveries: Every Minute Counts**

Gal Bachar^1^; Noa Frishman Martsiano^2^; Nizar Khatib^1^; Ron Beloosesky^3^; Dana Vitner^2^; Zeev Weiner^4^; Yaniv Zipori^5^

^1^Rambam Health Care Campus, Haifa, HaZafon; ^2^Rambam Medical Health Center, Haifa, Hefa; ^3^Rambam Health Care Campus, Rambam Health Care Campus, Hefa; ^4^Rambam Health Care campus, Haifa, Hefa; ^5^Rambam Health Care campus, Haifa, HaZafon

**Objective**: To evaluate the impact of decision‐to‐delivery time (DDT) on maternal and neonatal morbidity in emergency repeat cesarean deliveries (CD) following failed trial of labor after cesarean (TOLAC) for non‐reassuring fetal heart rates.

**Study Design**: This retrospective cohort study (2009–2023) included women ≥37 weeks of gestation with singleton pregnancies who underwent emergency CD during labor. Participants were divided into two groups: repeat CD after a prior cesarean (Group 1) and primary emergency CD in nulliparous women (Group 2). DDT was defined as the time (in minutes) from the decision for CD to birth. The primary outcome was a composite of maternal operative complications, including uterine dehiscence or rupture, scar extension, urinary tract injury, inverted T incision, and cesarean hysterectomy.

**Results**: Table 1 provides the demographic and secondary clinical characteristics of 2205 women analyzed. As presented in Figure 1, while the DDT times were similar between groups (12 ± 7 vs. 11 ± 7 minutes, *p* = 0.11), women in the repeat emergency CD group experienced a significantly higher incidence of composite adverse outcomes compared to those in the primary CD group (14.2% vs. 4.3%, *p* < 0.001). This difference was primarily driven by significantly higher rates of uterine dehiscence (4.7% vs. 0.1%), uterine rupture (3.1% vs. 0%), and bladder injuries (3.5% vs. 0.8%) in the repeat CD group. A subgroup analysis of repeat emergency CD cases showed significantly more adverse maternal outcomes with a median DDT of 11 minutes compared to 16 minutes (*p* = 0.008). Multivariate analysis revealed that TOLAC itself significantly increased the odds of adverse maternal outcomes (OR = 3.64, 95% CI: 2.37–5.60). Neonatal outcomes did not significantly differ between groups.

**Conclusion**: Emergency repeat CD for non‐reassuring fetal status is associated with a 3.3‐fold higher rate of composite adverse maternal morbidity. Future studies should focus on strategies to minimize maternal risks without compromising neonatal outcomes.

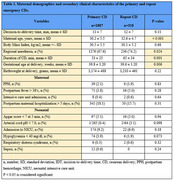

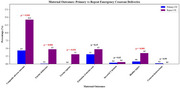

**3:30 PM – 5:00 PM**

## 1089 | **Intrapartum Glucose and Insulin Compared to Glucose Alone in Diabetic Women. A Randomized Controlled Trial**

Gali Garmi^1^; Mais Abu Nofal^2^; Noah Zafran^3^; Rana Abboud^4^; Rula Iskander^3^; Moran Levy^3^; Hanna Armaly^4^; Raed Salim^4^

^1^EMEK MEDICAL CENTER, AFULA, HaZafon; ^2^Maternal Fetal Medicine Division, Ontario Fetal Center, Department of Obstetrics and Gynaecology, Mount Sinai Hospital, University of Toronto, Toronto, ON, Canada, Toronto, ON; ^3^Emek Medical Center, Afula, HaZafon; ^4^Holy Family Hospital, Nazareth, HaZafon

**Objective**: There is currently no consensus on the optimal intrapartum protocol for maintaining maternal euglycemia and preventing neonatal hypoglycemia in diabetic pregnant women. We aimed to compare two intrapartum glycemic control protocols in terms of their impact on the incidence of neonatal hypoglycemia.

**Study Design**: A two‐site randomized controlled trial was conducted between October 2017 and July 2025. Diabetic women (pregestational or gestational) in labor with viable, singleton, term pregnancy were randomly allocated to one of two treatment protocols at a 1:1 ratio. Group 1 received an intravenous infusion of 0.9% saline and 5% glucose, along with a continuous insulin infusion. Group 2, received a similar infusion without insulin. Maternal glucose levels were monitored every 1–2 hours and both groups received additional insulin drip if glucose levels exceeded 100mg/dL. Randomization was stratified by delivery site. The primary outcome was neonatal hypoglycemia defined as a glucose level <40mg/dL. A total of 182 participants were required based on sample size calculation. The protocol was approved by the local IRBs and registered on September 2017 (ClinicalTrials.gov: NCT03273881). All participants provided informed consent.

**Results**: Overall, 182 women were randomized; 93 in Group 1 and 89 in Group 2. Baseline demographic and obstetric variables including pregestational BMI, weight gain during pregnancy, diabetes type, and rates of immediate postpartum breastfeeding were comparable between groups. No neonate in either group had a cord blood glucose level <40mg/dL. Neonatal hypoglycemia assessed from the first neonatal heelstick glucose level within the first 2 hours of birth occurred in 3 neonates (3.2%) in Group 1 and none (0%) in Group 2 (*p* = 0.25). Mean intrapartum maternal blood glucose levels, rates of cesarean delivery, macrosomia, Apgar score <7 at 5 min, and cord artery pH <7.1 were similar across both groups.

**Conclusion**: The incidence of neonatal hypoglycemia was low and did not differ significantly between the two intrapartum glycemic control protocols.

**3:30 PM – 5:00 PM**

## 1090 | **Risk of Neonatal Complications After Elevated Nuchal Translucency > 95%ile and Normal NIPT**

Genevieve Mazza^1^; Femitan Ajayi^1^; Ellie Marlor^1^; Jenny Chang^2^; Jonathan Steller^1^

^1^UC Irvine Medical Center, Orange, CA; ^2^University of California, Irvine, Irvine, CA

**Objective**: In an era of increased limitations to reproductive options, providing patients with early, accurate pregnancy counseling is imperative. Ultrasound of the fetal nuchal translucency (NT) and maternal serum noninvasive prenatal testing (NIPT) are key components of first trimester genetic screening. However, recent practice patterns have been shifting to NIPT alone, with de‐emphasis on NT scan after a low‐risk NIPT. The purpose of this study is to evaluate the rates of neonatal complications after an elevated NT ultrasound despite a low‐risk NIPT.

**Study Design**: This was a retrospective cohort study of patients who received an NT ultrasound at our center between 1/2021–12/2024. Those with an NT greater than the 95^th^ %ile and a low risk NIPT were included. The cohort was divided into two groups: NT between 95–98^th^ %ile and NT > 99^th^ %ile. The rates of neonatal complications in the entire cohort and subdivided by NT category were calculated.

**Results**: 96 subjects (54 with NT 95–98^th^%ile, 42 with NT > 99^th^%ile) met inclusion criteria. 13.5% of the entire cohort were diagnosed with > 1 neonatal structural abnormality (9.3% of those with NT 95–98%ile; 19% of those with NT > 99%ile). 4.17% required transfer to a pediatric hospital for higher level of care (0% of those with NT 95–98%ile; 9.52% of those with NT > 99%ile), and 2.08% required post‐natal surgery (0% of those with NT 95–98%ile; 4.8% of those with NT > 99%ile). Of the structural anomalies, cardiac was the most common (7.3% of the entire cohort, 1.8% of those with NT 95–98%ile; 14.3% with NT > 99%ile) followed by genitourinary. There was 1 neonatal demise in the group with NT > 99%ile.

**Conclusion**: Despite a low risk NIPT, an elevated NT > 95^th^%ile was associated with a 13.5% incidence of at least 1 neonatal structural abnormality and a 1.04% incidence of neonatal demise. These rates were even higher for those with NT > 99^th^ %ile. These elevated complication rates underscore the added value of an NT ultrasound to NIPT in pregnancy risk stratification in order to empower patients regarding their reproductive options.

**3:30 PM – 5:00 PM**

## 1091 | **Validation of the Neonatal Research Network Extremely Preterm Birth Outcome Tool**

George R. Saade

On behalf of the Eunice Kennedy Shriver National Institute of Child Health and Human Development Maternal‐Fetal Medicine Units Network, Bethesda, MD

Department of Obstetrics and Gynecology, Eastern Virginia Medical School at Old Dominion University, Norfolk, VA

**Objective**: The neonatal research network (NRN) estimator of outcomes is widely used to provide individualized counseling to patients at risk for extremely preterm birth. The calculator was based on data obtained from 2006 through 2012. Our objective was to evaluate whether the output of the calculator remains valid in a contemporary cohort.

**Study Design**: This was a secondary analysis of a randomized trial of pessary in singleton pregnancies with short cervix recruited from 2017 to 2021. All live neonates delivered from 22 0/7 through 25 6/7 weeks’ with no congential malformations were included. Those with no resuscitation after birth were excluded. The predicted survival using the NRN estimator was calculated for each neonate. The mean predicted survival for the neonates within each gestational age week and also for selected characteristics was calculated with 95% confidence intervals around the mean. The predicted survival was compared to the actual percent survival using a One‐Proportion test. A flexible calibration curve was produced using a non‐parametric (loess) smoother to assess how well the predicted survival probabilities matched the actual survival.

**Results**: Of 544 patients enrolled in the trial, 56 were included that delivered in the periviable period. The 42 neonates who survived were born later in gestation, had higher birthweight, and were more likely to have received corticosteroids compared with those who did not survive (Table 1). The actual average rate of survival per given week was slighty higher than what was predicted by the NRN model. The actual survival for those self‐reported as NH Black was slightly higher than predicted survival (87% vs. 63%, *p* = 0.01).The calibration curve showed that the calculator overestimated survival at lower actual survival rates, underestimated survival at higher survival rates, and had a strong prediction overall (ROC 0.75 95%CI 0.58, 0.87, Figure 1).

**Conclusion**: Overall  , the NRN estimator tool somewhat underestimates survival, but not to a significant degree and the tool estimates remain valid.

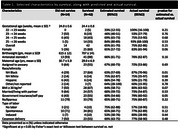

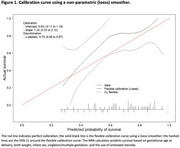

**3:30 PM – 5:00 PM**

## 1092 | **Identification of Maternal Alzheimer's Disease Risk After an Atypical Finding on SNP‐Based Prenatal cfDNA Screening**

Georgina D. Goldring^1^; Jordan Foster^2^; Anjali D. Zimmer^1^; Priyanka Arya^1^; Jeffrey T. Meltzer^1^

^1^Natera, Inc., Austin, TX; ^2^University of Arkansas for Medical Sciences, Fayetteville, AR

**Objective**: To report a case in which routine prenatal cfDNA screening identified an atypical finding (AF) on chromosome 21 (chr 21), emphasizing the importance of investigating AFs and the potential of cfDNA to reveal maternal genomic information with personal/familial health implications.

**Study Design**: Retrospective descriptive case series involving 2 sisters referred for genetic counseling (GC) following a chr 21 AF on SNP‐based prenatal cfDNA. Clinical and genetic evaluations were conducted across multiple pregnancies from 2021‐2025.

**Results**: A patient was referred for GC due to an AF on cfDNA suggestive of a copy number variant (CNV) on chr 21 of unknown origin. Maternal microarray identified a 3 Mb duplication at 21q21.2q21.3, encompassing the *APP* gene, which, when duplicated, is associated with autosomal dominant early‐onset Alzheimer's disease (ADEOAD). The patient reported no known prior genetic testing; however, family history was notable for early‐onset Alzheimer's disease. In a subsequent pregnancy, cfDNA screening again identified an AF on chr 21, this time of maternal origin, consistent with a duplication involving the APP gene.

Her sister had previously been referred for GC after an AF on cfDNA suggestive of a CNV on chr 21 of unknown origin. No follow‐up testing was performed at that time. During a subsequent pregnancy, she was referred again, and maternal microarray identified the same CNV that had previously been identified in her sister.

**Conclusion**: This case series highlights how delayed diagnoses, such as maternal APP duplication associated with ADEOAD, may stem from limitations in early recognition and follow‐up. As chr21 is routinely assessed in prenatal cfDNA screening, the incidental detection of AFs may reveal clinically relevant information for both mother and fetus. To facilitate timely diagnosis and informed reproductive decision‐making, pre‐test counseling should address the possibility of AFs, with prompt referral for GC and appropriate clinical follow‐up when they are identified.

**3:30 PM – 5:00 PM**

## 1093 | **Maternal Age at First Delivery and Future Psychiatric Morbidity of the Mother**

Gil Gutvirtz^1^; Tamar Wainstock^2^; Ruslan Sergienko^3^; Hagar Brami^4^; Eyal Sheiner^5^; Roy Kessous^6^

^1^Department of Obstetrics and Gynecology, Soroka University Medical Center, Ben‐Gurion University, Metar, HaDarom; ^2^Department of Epidemiology, Biostatistics and Community Health Sciences, Faculty of Health Sciences, Ben‐Gurion University of the Negev, Beer Sheva, HaDarom; ^3^Ben‐Gurion University of the Negev, ben gurion, HaDarom; ^4^Soroka, 0, HaDarom; ^5^Soroka University Medical Center, Faculty of Health Sciences, Ben‐Gurion University of the Negev, beer sheva, HaDarom; ^6^Soroka, BEER SHEVA, HaDarom

**Objective**: Advanced maternal age is known to be associated with higher rates of pregnancy complications. Maternal age at first delivery is rising, however, its impact on future maternal health is still under investigation. In this study, we aimed to examine a possible association between advanced maternal age at first delivery and long‐term psychiatric morbidity of the mother.

**Study Design**: A population‐based cohort analysis was conducted, including all deliveries of primigravids at a tertiary medical center, occurring between 1991 to 2021. Women were categorized in 4 groups based on age at first delivery (younger than 30 years, between 30–35, 35–40 and those over 40) and the incidence of maternal long‐term psychiatric morbidity (diagnosed in both community clinics and/or hospitalizations) was examined based on these age categories. A Kaplan‐Meier survival curve was used to assess cumulative incidence over time of psychiatric morbidity and a Cox regression model was used to control for possible confounders.

**Results**: A total of 77,786 mothers at their first delivery were included in the study. The cumulative incidence of psychiatric morbidity was higher as maternal age at first delivery was older, with the highest incidence noted for women older than 40 (Figure). However, the crude rates of psychiatric morbidity were comparable among groups (Table) and when applying the Cox proportional hazards model, controlling for ethnicity and the use of fertility treatments, and comparing to primipara women under 30 years of age as reference, older maternal ages at first delivery were not associated with an increased risk for future psychiatric morbidity of the mother (Table).

**Conclusion**: Advanced maternal age at first delivery is not a risk factor for future psychiatric morbidity of the mother.

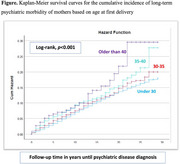

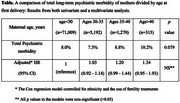

**3:30 PM – 5:00 PM**

## 1094 | **Long‐Term Ophthalmic morbidity of the Term Offspring Following Antenatal Corticosteroids Therapy Before 34 Weeks Gestation**

Gil Gutvirtz^1^; Tamar Wainstock^2^; Erez Tsumi^3^; Eyal Sheiner^4^

^1^Department of Obstetrics and Gynecology, Soroka University Medical Center, Ben‐Gurion University, Metar, HaDarom; ^2^Department of Epidemiology, Biostatistics and Community Health Sciences, Faculty of Health Sciences, Ben‐Gurion University of the Negev, Beer Sheva, HaDarom; ^3^Soroka University Medical Center, Beer‐Sheva, HaDarom; ^4^Soroka University Medical Center, Faculty of Health Sciences, Ben‐Gurion University of the Negev, beer sheva, HaDarom

**Objective**: Antenatal corticosteroids (ACS) are a cornerstone intervention in pregnancies at risk of preterm birth to reduce neonatal morbidity and mortality. However, concerns have emerged regarding potential long‐term effects including neurodevelopmental outcomes, particularly when ACS is administered but birth actually occurs at term. Data on long‐term ophthalmic morbidity following ACS exposure in term‐born offspring is limited. The present study was aimed to evaluate the long‐term ophthalmic outcomes in term‐born offspring who were exposed to ACS prior to 34 weeks’ gestation.

**Study Design**: A population‐based retrospective study of term deliveries between 1998‐2021 at a tertiary center was conducted. Long‐term ophthalmic morbidity was compared between children who were exposed to ACS prior to 34 weeks’ gestation to unexposed children, based on data from both community and hospitalization records. A Kaplan–Meier survival curve was used to compare the cumulative incidence of ophthalmic morbidity and a Cox regression model was constructed to control for confounders.

**Results**: A total of 182,626 term deliveries were included; 2093 (1.1%) children were exposed to ACS therapy prior to 34 weeks. Total ophthalmic morbidity incidence per 1000 person‐years (Table) as well as the cumulative incidence of ophthalmic morbidity over time (Figure) were higher in offspring exposed to ACS as compared with unexposed children. The Cox regression model, controlling for maternal and gestational age, ethnicity, diabetes mellitus, hypertensive disorders and cesarean delivery, found that ACS exposure is an independent risk factor for long‐term ophthalmic morbidity of the offspring (Table).

**Conclusion**: While ACS are crucial in preterm offspring, those who were exposed to ACS prematurely but born at term may suffer long‐term ophthalmic morbidity, highlighting the need for a more cautious application of ACS therapy when preterm birth is uncertain. Further studies are needed to elucidate the underlying mechanisms and to better define the long‐term safety profile of this critical therapy.

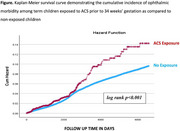

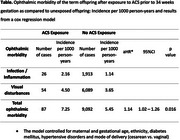

**3:30 PM – 5:00 PM**

## 1095 | **Impact of Maternal Stature on Cardiac Diastolic Load in Twin Pregnancies: A Height‐Stratified Echocardiographic Study**

Gisu Lee^1^; Jin‐Gon Bae^1^; Yu Ra Jeon^1^; SiYoung Park^1^; Sung Hun Na^2^; Young‐Han Kim^3^; Geum Joon Cho^4^; Han‐Sung Hwang^5^; Se Jin Lee^6^

^1^Keimyung University School of medicine, Daegu, Taegu‐jikhalsi; ^2^Department of Obstetrics and Gynecology, Kangwon National University, School of Medicine, Department of Obstetrics and Gynecology, Kangwon National University, School of Medicine, Kangwon‐do; ^3^Department of Obstetrics and Gynecology, Yonsei University College of Medicine, Department of Obstetrics and Gynecology, Yonsei University College of Medicine, Seoul‐t'ukpyolsi; ^4^College of Medicine, Korea University, Seoul, Seoul‐t'ukpyolsi; ^5^Konkuk University School of Medicine, Seoul, Seoul‐t'ukpyolsi; ^6^School of Medicine, Kangwon National University, Chuncheon, Kangwon‐do

**Objective**: To investigate whether shorter maternal height is associated with increased cardiac diastolic load in twin pregnancies using transthoracic echocardiographic parameters.

**Study Design**: This retrospective study analyzed 946 women with twin pregnancies who underwent transthoracic echocardiography during the third trimester. Subjects were stratified into two groups across multiple maternal height cutoffs (ranging from 155 to 170 cm). Diastolic function parameters, including mitral E‐wave velocity, E/e’ ratio, deceleration time (DT), isovolumic relaxation time (IVRT), and pulmonary artery systolic pressure (PASP) were compared between the shorter and taller groups at each cutoff.

**Results**: Across several height thresholds, women below the cutoff consistently exhibited echocardiographic signs of increased diastolic load. At the 158 cm cutoff, the shorter group showed significantly higher mitral E velocity (0.85 ± 0.25 vs. 0.79 ± 0.21 m/s, *p* = 0.001) and E/e’ ratio (7.43 ± 2.48 vs. 6.84 ± 2.15, *p* = 0.004), along with shorter deceleration time (180 ± 24.2 vs. 186.2 ± 33.6 ms, *p* = 0.026) and IVRT (80.6 ± 12.1 vs. 83.4 ± 12.5 ms, *p* = 0.006). These patterns remained consistent and statistically significant across the 157–164 cm range. PASP was also elevated in the shorter group at several cutoffs (e.g., 22.4 ± 7.2 vs. 20.8 ± 6.4 mmHg at 158 cm, *p* = 0.019).

**Conclusion**: Short maternal stature in twin pregnancies is associated with echocardiographic indicators of increased diastolic filling pressure and reduced ventricular relaxation time. These findings suggest that women with smaller body size may have limited cardiovascular adaptation to the increased circulatory load of twin gestation. Diastolic focused cardiac assessment may aid in risk stratification and management of twin pregnancies in shorter statured women.

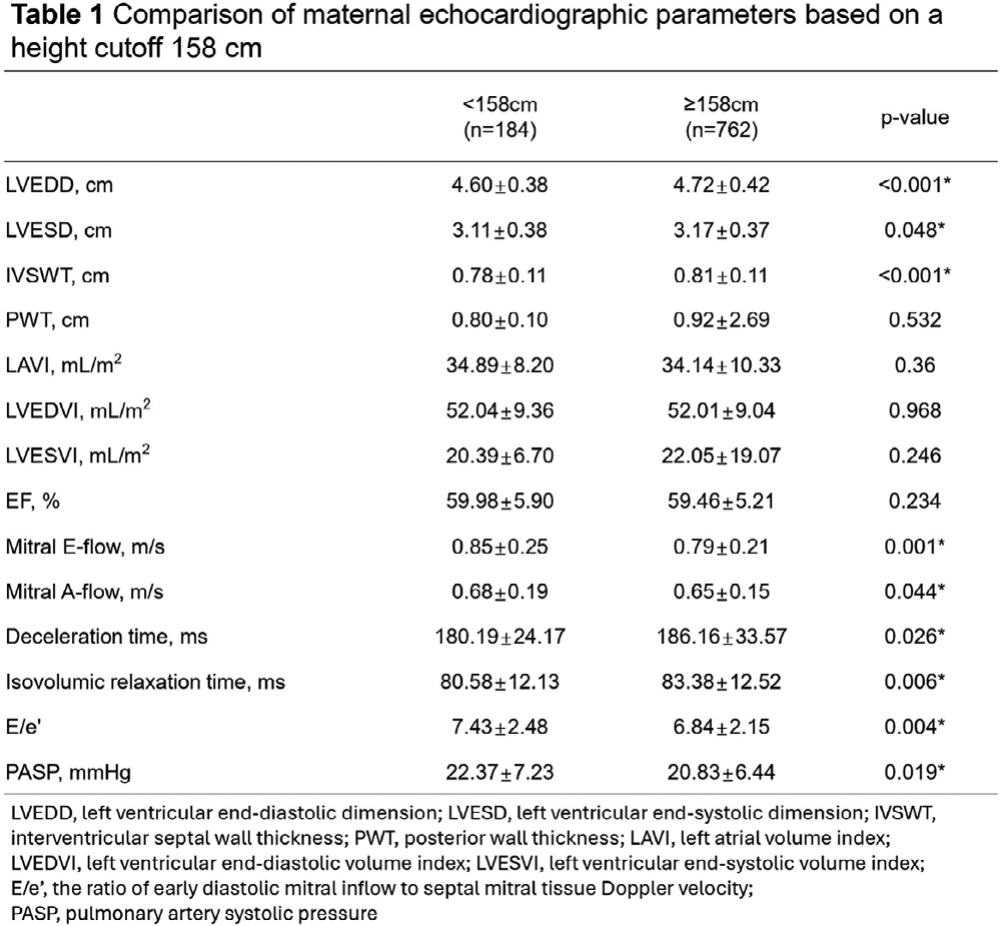

**3:30 PM – 5:00 PM**

## 1096 | **Antibody Titers Rise After Transplacental IUT: A Hypothesis‐Generating Study**

Giulia Bonanni^1^; Emily Hutson^2^; Erin E. Moise^2^; Alireza A. Shamshirsaz^3^; Kenneth J. Moise, Jr.^2^

^1^Division of Fetal Medicine and Surgery, Boston Children's Hospital Harvard Medical School, Boston Children's Hospital, MA; ^2^Department of Women's Health, Dell Medical School at the University of Texas at Austin, Austin, TX; ^3^Division of Fetal Medicine and Surgery, Boston Children's Hospital Harvard Medical School, Boston, MA

**Objective**: To evaluate whether transplacental intrauterine transfusion (IUT) increases maternal alloantibody production. We hypothesized that transplacental IUT enhances maternal exposure to fetal red blood cell (RBC) antigens, increasing the degree of alloimmunization (Figure 1).

**Study Design**: We retrospectively reviewed patients undergoing IUT for RBC alloimmunization at a single center. The primary exposure was placental location (anterior vs posterior), which informs procedural approach and risk of transplacental needle passage. To refine exposure classification, procedural videos were reviewed to confirm whether the needle traversed the placenta, enabling a second exposure definition: verified transplacental vs non‐transplacental. A titer increase was defined as any rise in antibody titer between the pre‐IUT#1 and the next available post‐IUT value. Statistical analyses included Fisher's exact test and t‐tests. Clinical relevance was assessed using Number Needed to Harm (NNH), and power calculations were performed to estimate sample size needs for future studies.

**Results**: Among 28 patients, a titer increase occurred in 7/10 (70%) anterior vs 5/18 (28%) posterior placentas (OR 5.63, 95% CI 0.86–48.5, *p* = 0.05; Δlog_2_ titer: 1.20±1.03 vs 0.22±1.31, *p* = 0.04). When reclassified by observed transplacental entry, a rise occurred in 8/14 (57%) vs 4/14 (29%) (OR 3.19, 95% CI 0.55–21.7, *p* = 0.25). NNH was 2.5 (95% CI: 1.5–35.1) for anterior placenta and 3.6 (95% CI: 1.6–9.5) for transplacental approach (Table 1).

**Conclusion**: Anterior placentation was associated with a higher risk of increase in alloantibody titer after the first IUT. A similar, though non‐significant, trend was observed with confirmed transplacental entry. Power analysis suggests 56 patients are needed to detect the observed titer difference, and 92 for a binary outcome. These findings inform IUT counseling and support the need for a larger prospective study. If confirmed, rising titers may justify adjusting the interval between first and second IUT due to the potential for more rapid decline in fetal hematocrit secondary to enhanced alloimmunization.

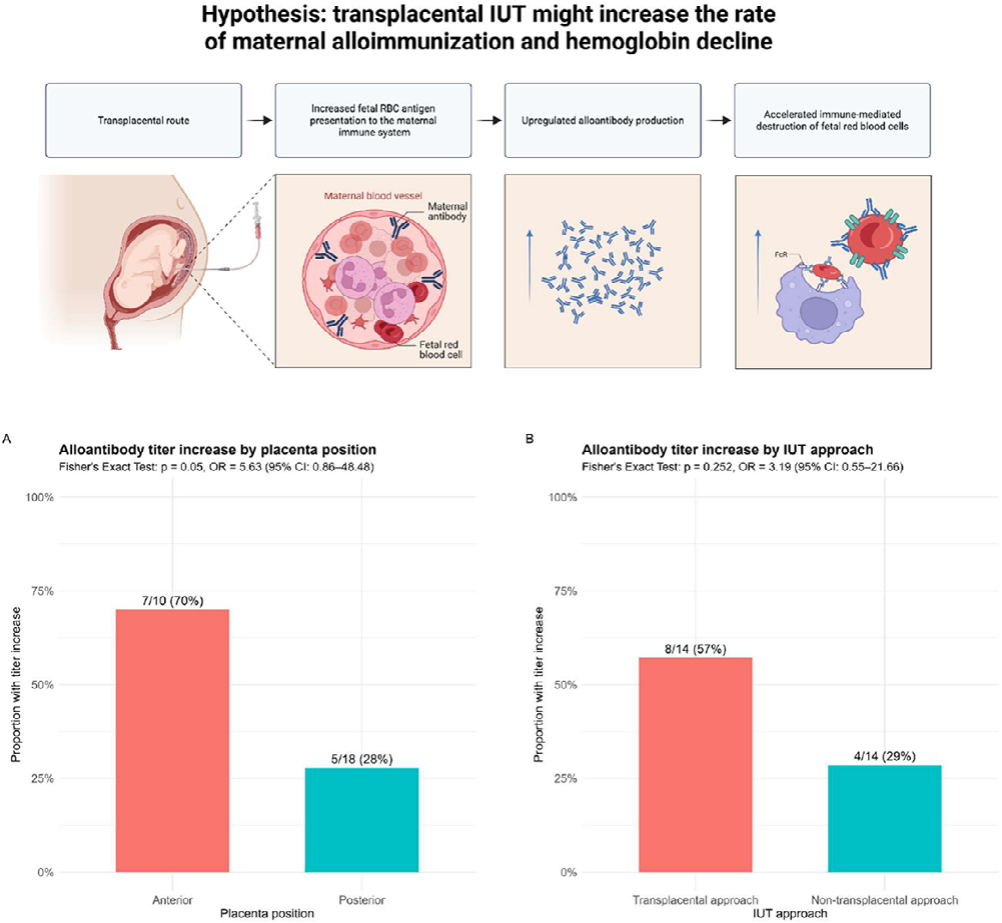

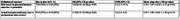

**3:30 PM – 5:00 PM**

## 1097 | **Maternal Morbidity Among Pregnant Women With Placenta Accreta Spectrum Treated Conservatively Versus Non‐Conservatively**

Grace Zhao^1^; Nefertiti OjiNjideka Hemphill^2^; Maria Fernandez^3^; Katelyn DeAlmeida^4^; Beth L. Pineles^5^

^1^Western University, London, ON; ^2^Nutrient Innovation, Nutrient Innovation, IL; ^3^Harvard, Harvard University, MA; ^4^Northwestern Medicine, Harvard University, MA; ^5^University of Pennsylvania, Philadelphia, PA

**Objective**: Placenta accreta spectrum (PAS) is a life‐threatening condition that is increasing in incidence along with cesarean rates worldwide. The current standard of management of PAS is cesarean hysterectomy. Few studies have compared maternal outcomes after cesarean hysterectomy versus a uterus‐preserving approach. Thus, this study aims to compare maternal morbidity for women with PAS who undergo non‐conservative versus conservative management.

**Study Design**: This is a retrospective cohort study using delivery hospitalization records from the 2021‐2022 National Inpatient Sample. Pregnant individuals aged 18 and older who delivered at ≥ 20 weeks’ gestation and had a documented diagnosis of PAS were included. Baseline characteristics, exposures, and outcomes were identified using ICD‐10 codes. Weighted survey methods accounting for the complex sampling design were applied. Odds ratios (OR) with 95% confidence intervals (CI) were estimated using logistic regression. A sensitivity analysis was performed in a restricted cohort of individuals diagnosed with various PAS subtypes.

**Results**: From this representative sample of 20% of all US hospitals, a weighted sample of delivery hospitalizations from 4433 women with PAS and 33,108,352 women without PAS were obtained. Of those with PAS who delivered during admission, 813 underwent cesarean hysterectomy and 960 were managed conservatively. Women with PAS who were managed conservatively had a 67% reduction in risk of developing at least one morbidity during their hospitalization (aOR 0.33, 95% CI: 0.25–0.43). Conservative management was associated with less shock (OR 0.19, 95% CI: 0.10–0.37), disseminated intravascular coagulation (OR 0.34, 95% CI: 0.21–0.55), and blood transfusions (OR 0.33, 95% CI: 0.26–0.42). This association remained statistically significant with a restricted study population limited to increta, percreta, and accreta cases who also had a placenta previa (aOR 0.40, 95% CI: 0.27, 0.60).

**Conclusion**: Conservative management of PAS is associated with significantly lower risk of maternal morbidity.

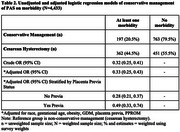

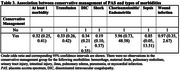

**3:30 PM – 5:00 PM**

## 1098 | **Savings From Early Detection of Hypertension Among Medicaid Pregnant Women With Chronic Conditions**

Gregory Wozniak^1^; Stavros Tsipas^2^; Alejandro Arrieta^3^

^1^American Medical Association, Chicago, IL; ^2^American Medical Association, Chicago, Armed Forces ‐ AE; ^3^FLORIDA INTERNATIONAL UNIVERSITY, Miami, FL

**Objective**: Previous research has shown that self‐measured blood pressure (SMBP) monitoring is able to detect elevated BP (BP ≥140/90 mm Hg) in pregnant women 29 days before a confirmed diagnosis at the clinic. We use this evidence to estimate the potential monetary savings of SMBP associated with increases in gestational age.

**Study Design**: To assess monetary savings of early detection of hypertension (HTN), we assume that a fraction of women with SMBP receive earlier detection of HTN, receive appropriate treatment, and delay her delivery. To estimate the gain in gestational age, we analyzed data from the CHAP Trial which assessed the effects of initiating antihypertensive treatment among pregnant women with chronic HTN targeting a BP of <140/90 mm Hg. The composite primary outcome of the trial included medically indicated preterm birth before 35 weeks’ gestation. Using two cumulative data points from the trial, we estimated the distributions of gestational age at delivery for control (*w_1_*) and intervention (*w_2_*) groups, 16.7% (31.4%) of deliveries occurred at <35 (<37) weeks and 12.2% (27.5%) of deliveries occurred at <35 (<37) weeks, respectively. Assuming a normal distribution of gestational age (in days), we fitted the *cdf*s: *w_1_* ∼N(39.00, 4.42) and *w_2_* ∼N(39.19, 4.14). For gestational ages from 23 to 40 weeks, we estimated the gain in gestational age using the fitted normal cumulative distribution functions for control and intervention groups.

**Results**: We found an overall increase of 3.6 days of gestational age by early detection of HTN and anti‐hypertensive treatment among women with chronic HTN and an estimated cost saving of $5,818 per pregnant women, equivalent to a 26.6% reduction in costs. Applying this cost reduction rate to HTN and preeclampsia costs, adjusted to Medicaid rates and inflating to 2023 US dollars, suggests a savings of $3,456 among women with chronic HTN and $7,679 among women with pre‐eclampsia/eclampsia.

**Conclusion**: SMBP monitoring during pregnancy and postpartum yields substantial economic benefits among Medicaid beneficiaries, particularly among women with chronic HTN.

**3:30 PM – 5:00 PM**

## 1099 | **Early vs. Late Cholestasis of Pregnancy: A Comparative Study of Clinical Features and Pregnancy Outcomes**

Hadel Watad^1^; Aviran Ohayon^1^; Raanan Meyer^1^; Rakeft Yoeli Ullman^2^; Shali Mazaki‐Tovi^3^

^1^Sheba Medical Center, Ramat Gan, Tel Aviv; ^2^The Sheba Medical Center, The Sheba Medical Center, HaMerkaz; ^3^Department of Obstetrics and Gynecology, Sheba Medical Center, Ramat Gan, Tel Aviv

**Objective**: To compare adverse pregnancy outcomes in intrahepatic cholestasis of pregnancy (IHCP) stratified by timing of diagnosis: Early versus Late.

**Study Design**: A retrospective cohort study of all IHCP cases at a single center (July 2014–January 2022). Pregnancies were grouped by the timing of diagnosis:1.  Early (<28 W) and 2. Late (≥28 W). Parametric and non‐parametric tests were used to compare clinical and obstetric outcomes. In addition, multivariable linear regression analysis was performed to identify factors independently associated with gestational age (GA) at delivery.

**Results**: A total of 345 individuals were included: 1. 49 with early‐onset and 2.  296 with late‐onset IHCP. Early‐onset cases had higher rates of prior preterm labors (PTL) (30.6% vs. 15.5%, *p* = 0.015). Moderate IHCP (Total Bile Acids (TBA) ≥ 40 µM) was more frequent in early‐onset cases (42.2% vs. 28.7%, *p* = 0.047), with earlier treatment initiation (median 27, IQR 24–29 vs. 34, IQR 32–35 W, *p* < 0.001) and a higher rate of rifampin use (12.2% vs. 3.4%, *p* = 0.016). All other baseline characteristics were comparable (Table 1).

Early‐onset IHCP was associated with lower GA at delivery (median 36, IQR 32.5–37 vs. 36, IQR 35–37 W, *p* = 0.013), higher rates of PTL< 34 W (26.5% vs. 6.4%, *p* < 0.001), more cesarean deliveries (49% vs. 31%, *p* = 0.014), and fewer labor inductions (49% vs. 63.9%, *p* = 0.047). Rates of PTL< 37 W, PPROM, and IUFD were comparable.

Neonates in the early‐onset group had higher NICU admissions, lower birthweights and Apgar scores, though SGA rates were similar (Table2).

On regression analysis, GA at symptom onset was positively associated with GA at delivery (B = 0.107, *p* < 0.001), while higher TBA levels (B =  – 0.016, *p* < 0.001) and twin gestation (B =  – 1.471, *p* < 0.001) were associated with earlier delivery. Other variables, including maternal age, pre‐pregnancy BMI, smoking status, primiparity, previous PTL and history of cholestasis were not significantly associated with GA at delivery.

**Conclusion**: Early‐onset IHCP is linked to more severe disease, higher rates of early PTL, and increased neonatal morbidity compared to late‐onset IHCP.

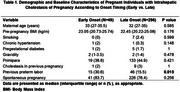

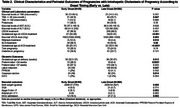

**3:30 PM – 5:00 PM**

## 1100 | **Incidence of New Recommended Criteria for Severe Maternal Morbidity in Placenta Accreta Spectrum Disorder**

Hadley Ross^1^; Mira Woods^1^; Maharajni Perla^1^; Mikayla Hernandez^1^; Sarah Chang^1^; Jose Roble^2^; Yasmin A. Lyons^1^; Kayla E. Ireland^1^

^1^UT Health San Antonio, San Antonio, TX; ^2^UT Health San Antonio, UT Health San Antonio, TX

**Objective**: Given that CDC severe maternal morbidity (SMM) definitions classify *anticipated* interventions in management of PAS as complications, *specific standardized* criteria for SMM in PAS (PAS‐SMM) have been proposed. Our goal was to understand the incidence of PAS‐SMM compared to standard SMM criteria in a contemporary cohort.

**Study Design**: We conducted a retrospective cohort study of patients with histopathology confirmed PAS managed at large academic center from January 2020‐June 2025. Severe maternal morbidity outcomes were obtained from electronic medical records. Categorical variables were compared using Chi square or Fischer's exact and continuous variables were compared with Mann Whitney U. Maternal demographics and delivery outcomes that met criteria for SMM without transfusion and hysterectomy were compared to those with PAS‐SMM (see Table 2 for criteria).  Additionally, cases with PAS‐SMM were compared to those without.

**Results**: During the study time period, 114 cases of PAS were delivered by cesarean hysterectomy. 80% had severe PAS (percreta, *n* = 92) and median gestational age at delivery was 34 [32, 34] weeks. 100% of patients were found to have SMM events by standard definition, however, when transfusion and hysterectomy were excluded, this dropped to 29% (*n* = 34) (*p* < 0.001). Using PAS‐SMM, only 20% (*n* = 23) of patients met SMM criteria. Compared to women without SMM‐PAS, PAS‐SMM patients had significantly more antenatal vaginal bleeding (*p* = 0.04), blood loss (1500 [950, 2867] vs 3835 [2342, 5500] mL, *p* = 0.001) and unscheduled cases (*p* = 0.001). No cases of immediate maternal death, acute renal failure, cardiogenic shock, acute coronary syndrome, cardiac arrest, or stroke were noted. The most common PAS‐SMM outcome was transfusion > 8units pRBCs (*n* = 10, 8%).

**Conclusion**: Traditional SMM metrics overstate morbidity in PAS due to inclusion of expected interventions like blood transfusion and hysterectomy. Application of proposed PAS‐specific SMM criteria results in a substantially lower and potentially more accurate morbidity rate.

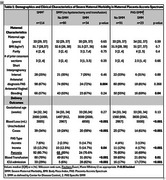

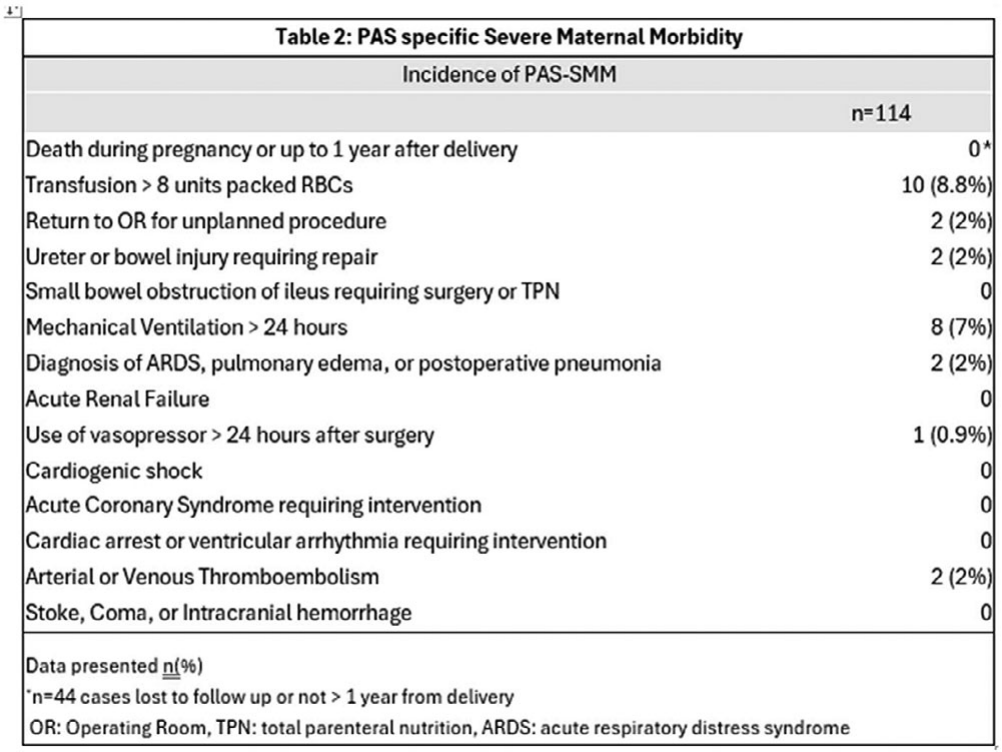

**3:30 PM – 5:00 PM**

## 1101 | **Validation and Feasibility of Communication Assessment Tool Use in Hospitalized Antepartum Patients**

Hannah Kelly^1^; Aya Bashi^1^; Audrey Aitelli^2^; Jennifer Paul^2^; Anne West‐Honart^3^; Rachel L. Wood^3^

^1^Duke University Hospital, Durham, NC; ^2^Duke University School of Medicine, Durham, NC; ^3^Division of Maternal Fetal Medicine, Department of Obstetrics and Gynecology, Duke University, Durham, NC

**Objective**: Hospitalized antepartum patients are at elevated risk for postpartum mood disorders, and effective provider communication mitigates this risk. The Communication Assessment Tool (CAT) is a validated instrument for measuring patient‐reported physician communication but has not been evaluated in the inpatient antepartum setting. We aimed to (1) assess the feasibility of the CAT in this population and (2) establish baseline CAT scores to inform future interventions.

**Study Design**: We conducted a prospective, cross‐sectional study on the labor and delivery unit of an academic medical center. English‐speaking antepartum patients admitted for ≥24 hours were eligible. Exclusion criteria included fetal demise, ICU admission, unstable medical or obstetric condition, and postpartum or recent admissions. The 15‐item CAT was administered electronically via REDCap prior to discharge or delivery. Primary outcomes included feasibility (completion rate, workflow, missing data) and baseline CAT scores (mean, % “excellent” responses). Cronbach's α assessed internal consistency. The study was deemed IRB exempt.

**Results**: Fifteen of sixteen patients completed the CAT (93.8%). Average scores ranged from 4.00 to 4.47 on a 5‐point Likert scale (Table 1), with “Showed interest in my ideas” (4.47, 53.4% “excellent”) and “Greeted me in a way that made me feel comfortable” (4.40, 53.4%) scoring the highest. The lowest‐rated question was “Encouraged me to ask questions” (4.07, 40.0%). Internal consistency was high (Cronbach's α = 0.92). Score variability was adequate (SD range 0.63–1.13).

**Conclusion**: CAT administration among inpatient antepartum patients was feasible, with strong internal consistency, sufficient score variability, and promising response rates. This pilot establishes communication benchmarks and informs design and power planning for a randomized trial of physician rounding strategies. Future steps include initiatives to improve CAT scores and evaluate whether higher scores are associated with lower rates of postpartum mood disorders in this high‐risk population.

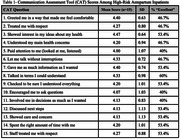

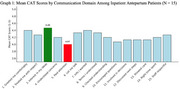

**3:30 PM – 5:00 PM**

## 1102 | Breastfeeding Intensity and Long‐Term Dyslipidemia Risk in Women With Prior Gestational Diabetes: A Mediation Analysis

Harumi Kanzawa^1^; Ichiro Yasuhi^2^; Hiroshi Yamashita^2^

^1^Nagasaki University Graduate School of Biomedical Sciences, Omura, Nagasaki; ^2^NHO Nagasaki Medical Center, Omura, Nagasaki

**Objective**: Women with prior gestational diabetes (GDM) face elevated risks of long‐term dyslipidemia. While breastfeeding is generally beneficial for postpartum metabolic health, its long‐term effects on lipid profiles in this population remain unclear. We previously showed that higher breastfeeding intensity was associated with improved lipid profiles in early postpartum. This study examined whether high‐intensity breastfeeding (HIB) in early postpartum is associated with reduced risk of elevated triglyceride‐to‐HDL (TG/HDL) ratio (> = 2.1) at 5 years, and whether early postpartum TG/HDL ratio mediates this association.

**Study Design**: We analyzed data from 521 women with prior GDM followed for 5 years at a Japanese tertiary perinatal center. Breastfeeding intensity was classified as HIB or non‐HIB during early postpartum. TG/HDL ratio was assessed at early postpartum and again at 5 years. The primary outcome was TG/HDL > = 2.1 at 5 years. Mediation analysis was performed using bootstrapping (*n* = 1000), adjusting for age, prepregnancy BMI, postpartum week, maternal weight, and fasting glucose at 5 years.

**Results**: HIB was significantly associated with lower early postpartum TG/HDL ratio (β =  – 0.16), which strongly predicted TG/HDL > = 2.1 at 5 years (β =  – 0.99) (Figure). The indirect effect of HIB on 5‐year dyslipidemia risk through early TG/HDL ratio was significant (bootstrapped 95% CI:  – 0.241 to  – 0.096). The direct effect was small and marginally positive (β = +0.11). These findings suggest that HIB indirectly reduces long‐term dyslipidemia risk by improving early TG/HDL ratio.

**Conclusion**: Among women with prior GDM, early high‐intensity breastfeeding did not directly lower 5‐year dyslipidemia risk but had a significant indirect effect via early lipid improvement. Promoting breastfeeding may serve as a metabolic window for long‐term cardiovascular risk reduction.

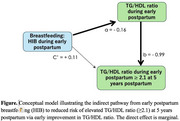

**3:30 PM – 5:00 PM**

## 1103 | **Severe Maternal Morbidity Associated With the Individual and Interactive Effects of Influenza, Asthma, and Hypertension**

Havilah Reimche‐Vu^1^; Bo Park^2^; Nida Ali^3^; Ashra Tugung^3^; Neville Tritch^1^; Kriti N. Vedhanayagam^4^; Kevin H. Hu^4^; Sergio A. Karageuzian^5^; Ilish Gedestad^5^; Ruofan H. Yao^4^

^1^Loma Linda University School of Medicine, Loma Linda, CA; ^2^California State University, Fullerton, Fullerton, CA; ^3^Loma Linda University Children's Health | Perinatal Institute, Loma Linda, CA; ^4^Loma Linda University, Loma Linda, CA; ^5^Loma Linda University Health, Loma Linda, CA

**Objective**: To evaluate the association between influenza, asthma, hypertension (HTN), and their combined interaction with the risk of severe maternal morbidity (SMM).

**Study Design**: This is a population‐based, retrospective cohort study using the California Office of Statewide Health Planning and Development Linked Birth File with hospital discharge International Classification of Diseases, 9th Revision, Clinical Modification (ICD‐9‐CM) diagnoses between 2007 and 2012. Chi‐square tests were used to determine the association between influenza, asthma, HTN, and SMM. Cox proportional hazard analysis determined the risk of SMM associated with each condition, both individually and combined. A cumulative hazard graph was made to show the timing of SMM events during pregnancy due to these conditions.

**Results**: Among the 3,076,580 individuals analyzed, 45,261 (1.47%) experienced SMM. Women who did not have influenza, asthma, or HTN were the majority (2,980,400; 98.58%), with a reference SMM rate of 1.42%.

Individuals with influenza only (*n* = 576) had an elevated SMM rate of 12.15% (HR 9.24 [95% CI: 7.31–11.69]). Those with HTN only (*n* = 14,254) had a SMM rate of 5.62% (HR 5.43 [95% CI: 5.06–5.83]). Individuals with asthma only (*n* = 80,277) showed a SMM rate of 2.30% (HR 1.48 [95% CI: 1.41–1.55]).

Comorbid conditions were associated with compounding risk. The interaction of flu and asthma (*n* = 49) resulted in a SMM rate of 16.33% (HR 14.17 [95% CI: 7.09‐28.34]). Those with influenza and HTN (*n* = 7) had an elevated SMM rate of 71.43% (HR 58.17 [95% CI: 24.20‐139.82]). Individuals with asthma and HTN (*n* = 1,016) had a SMM rate of 8.07% (HR 7.31 [95% CI: 5.89‐9.08]). The influenza, asthma, and HTN (*n* = 1) cohort was too small to associate with SMM risk.

**Conclusion**: This study shows that influenza, HTN, and asthma are all independent risk factors associated with SMM. Influenza infection had a multiplicative effect on other risk factors associated with SMM. Whereas asthma and HTN demonstrated only additive effects on SMM.

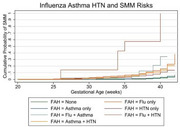

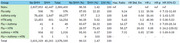

**3:30 PM – 5:00 PM**

## 1104 | **The Role of Bone Marrow‐Derived Macrophage to Prevent Preterm Birth in a Murine Model**

Hee Young Cho, N/A^1^; Ji Yeon Lee^2^; So Hee Park^3^; Mira Park^3^; Haengseok Song^3^

^1^Obstetrics and Gynecology, Seoul National University College of Medicine, Seoul, Kyonggi‐do; ^2^Department of Obstetrics and Gynecology, CHA Bundang Medical Center, CHA University, Seongnam, Kyonggi‐do; ^3^Department of Biomedical Sciences, CHA University, Seongnam, Kyonggi‐do

**Objective**: The purpose of this study was to investigate the importance of macrophages in maintaining pregnancy and preventing preterm birth (PTB) in a murine model.

**Study Design**: To induce PTB, Lipopolysaccharide (LPS) 25 ug in 100 ul PBS was injected in the middle of the first and second amniotic membranes in 16.5 dpc ICR mice. To deplete macrophages, clodronate liposomes were injected in 15.0, 15.5, and 16.0 dpc ICR mice intravenously. Macrophages in the bone marrow (BM), uterus, placenta, and decidua collected from 16.5dpc ICR mice were analyzed by fluorescence‐activated single‐cell sorting. Monocytes obtained from BM flushing were cultured to differentiate into macrophages (BMDMs), and then BMDMs were further polarized into M2‐like macrophages using IL‐4. M2‐polarized BMDM was injected in 15.0 and 16.5 dpc mice treated with LPS to rescue LPS‐induced PTB.

**Results**: The percentage of F4/80+ macrophages decreased 12 hours after the clodronate liposomes injection and PTB spontaneously occurred in 80% (4/5) of cases without LPS. F4/80+ macrophages were significantly reduced in bone marrow, uterus, placenta, and decidua of mice that received clodronate liposomes. Particularly, there was a significant decrease of F4/80+ macrophages in the BM. PTB occurred within 48 hours (4/4) in all mice in the LPS group, whereas the M2‐polarized BMDM significantly reduced PTB in pregnant mice that received LPS. Only 25% of pregnant mice (1/4) with M2 BMDM therapy suffered from LPS‐induced PTB.

**Conclusion**: We demonstrate that balanced macrophage polarity could be a critical factor to maintain pregnancy and prevent inflammation‐induced PTB in mice.

**3:30 PM – 5:00 PM**

## 1105 | **Antenatal Corticosteroids and Neonatal Outcomes in Periviable Twin Pregnancies: A National Cohort Study**

Hiba J. Mustafa^1^; Faezeh Aghajani^2^; Erez Lenchner^3^; Moti Gulersen^4^; Vincenzo Berghella^5^; Alireza A. Shamshirsaz^6^

^1^Division of Maternal‐Fetal Medicine, Indiana University School of Medicine, Indianapolis, IN; ^2^Boston Children's Hospital, Boston, MA; ^3^New York University Rory Meyers College of Nursing, New York, NY; ^4^Division of Maternal Fetal Medicine, Department of Obstetrics, Gynecology and Reproductive Science, Jefferson Health, Philadelphia, PA, Philadelphia, PA; ^5^Thomas Jefferson University, Philadelphia, PA; ^6^Boston Children's Hospital, Harvard Medical School, Boston, MA

**Objective**: To evaluate trends in antenatal corticosteroid (ACS) administration and compare neonatal outcomes in ACS‐exposed versus unexposed twin pregnancies delivered between 22 and 25+6 weeks’ gestation.

**Study Design**: We conducted a cross‐sectional study using the National Center for Health Statistics and CDC natality dataset (2016–2022). Included were liveborn twin pregnancies delivered between 22 and 25+6 weeks; singletons, higher‐order multiples, those with gestational age or ACS data missing, or outside this window were excluded. The primary outcome was neonatal survival; secondary outcomes included 10‐minute Apgar score <7, prolonged ventilation, surfactant therapy, suspected neonatal sepsis, and a composite adverse outcome (ventilation, Apgar <7, or death). Associations were estimated with multivariable logistic regression.

**Results**: Among 15,833 twin births, 6982 (44%) were exposed to ACS and 8851 (56%) were unexposed. ACS administration was associated with higher neonatal survival at 22–22+6 weeks (55.9% vs. 26.8%; aOR 3.66, 95% CI 2.95–4.55), 23–23+6 weeks (74.1% vs. 65.6%; aOR 1.53, 95% CI 1.30–1.79), and 24–25+6 weeks (85.4% vs. 82.2%; aOR 1.21, 95% CI 1.02–1.44). Odds of a 10‐minute Apgar score <7 were lower with ACS at 22–22+6 weeks (aOR 0.56, 95% CI 0.45–0.69), 23–23+6 weeks (aOR 0.84, 95% CI 0.72–0.98), and 24–25+6 weeks (aOR 0.68, 95% CI 0.58–0.80). Among neonates receiving active intervention (*n* = 9,118), the survival benefit with ACS remained significant at 22 (aOR 2.5, 95% CI 1.77–3.54) and 23 weeks (aOR 1.56, 95% CI 1.26–1.94). From 2016–2022, ACS use increased significantly at all gestational ages, most markedly at 22 weeks (from 13.5% to 42.9%), with a notable rise after the 2021 ACOG advisory.

**Conclusion**: ACS administration in periviable twin pregnancies is linked to improved survival and lower odds of adverse outcomes, especially at 22 and 23 weeks. Benefit persists among those receiving active postnatal care, supporting ACS use when intensive neonatal management is planned.

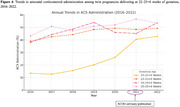

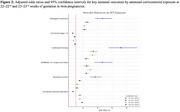

**3:30 PM – 5:00 PM**

## 1106 | **Association Between Artificial Rupture of Membranes and Labor Outcomes in Second‐Trimester Induction of Labor**

Hillary McLaren^1^; Vanya Manthena^2^; Eryn Wanyonyi^2^; Camille Johnson^2^; Laura Laursen^3^; Julie Chor^2^; Beth Plunkett^4^; Emily Barker^5^; Sloane York^3^; Jocelyn Wascher^2^; Isa Ryan^4^; Ashish Premkumar^1^

^1^University of Chicago, Chicago, IL; ^2^University of Chicago Medicine, Chicago, IL; ^3^Rush University Medical Center, Chicago, IL; ^4^Endeavor Health Evanston, Evanston, IL; ^5^Hope Clinic, Granite City, IL

**Objective**: To evaluate the association between artificial rupture of membranes (AROM) and labor duration in second‐trimester induction of labor (2T IOL). Secondary objectives included evaluating associations between AROM and morbidity.

**Study Design**: We conducted a retrospective cohort study of individuals undergoing 2T IOL at four academic hospitals from 2009–2019. Patients with twin gestations, preterm labor, cervical insufficiency, or rupture of membranes were excluded. The primary outcome was labor duration, defined as hours from first misoprostol dose to fetal expulsion. Secondary outcomes included a composite morbidity measure (suspected chorioamnionitis, blood transfusion, ICU admission, uterine rupture, readmission within 6 weeks, or death) and each complication individually. Labor duration was analyzed using linear regression and Cox survival modeling. Bivariate analysis identified no confounders, so final models were unadjusted. Secondary outcomes were assessed using regression analysis.

**Results**: Among 681 patients meeting inclusion criteria, 81 (11.9%) underwent AROM. The groups differed only by induction methods. AROM was associated with significantly longer labor (mean 16.14 vs. 13.51 hours, *p* = 0.02). In unadjusted Cox modeling, AROM was associated with increased time to delivery (HR 0.76, 95% CI 0.60–0.96), confirmed by Kaplan‐Meier analysis (log‐rank *p* < 0.001). AROM was associated with increased composite morbidity (RR 1.52, 95% CI 1.14–2.02), primarily driven by suspected chorioamnionitis (RR 1.88, 95% CI 1.22–2.89).

**Conclusion**: In patients undergoing 2T IOL, AROM was associated with an average increase in labor duration of 2.63 hours, a 52% increase in composite morbidity, and an 88% increased risk of suspected intraamniotic infection. These findings suggest AROM may contribute to prolonged labor and increased risk of infection in 2T IOL, warranting cautious clinical use.

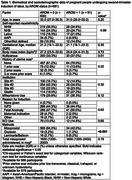

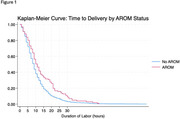

**3:30 PM – 5:00 PM**

## 1107 | **Disposition Index Better Differentiates Insulin Dynamics in Gestational Diabetes Than Matsuda or Insulinogenic Index**

Hiroshi Yamashita^1^; Sohei Hamazaki^1^; Harumi Kanzawa^2^; Ichiro Yasuhi^1^

^1^NHO Nagasaki Medical Center, Omura, Nagasaki; ^2^Nagasaki University Graduate School of Biomedical Sciences, Omura, Nagasaki

**Objective**: To examine whether the disposition index (DI), which reflects the relationship between insulin sensitivity and secretion, better characterizes underlying insulin dynamics in women with GDM compared to the Matsuda index (MI) or insulinogenic index (IGI), with the goal of developing physiologically relevant subclassifications of GDM beyond glucose‐based diagnosis alone.

**Study Design**: This was a cross‐sectional study of 488 pregnant women who underwent a 75g oral glucose tolerance test (OGTT) between 24 and 32 weeks of gestation at a tertiary perinatal center in Japan. Based on OGTT results, women were categorized as having GDM (*n* = 174) or normal glucose tolerance (*n* = 314). Three indices were calculated from the OGTT data: MI (whole‐body insulin sensitivity), IGI (early‐phase insulin secretion), and DI (IGI × MI, reflecting β‐cell compensatory capacity). We compared means between groups using t‐tests and evaluated discriminatory ability with ROC curve analysis. The area under the curve (AUC) was compared for each index.

**Results**: All three indices were significantly lower in the GDM group compared to the normal group (*p* < 0.00001 for each). Disposition index demonstrated the highest discriminatory ability for identifying GDM: AUC for DI = 0.80, compared to IGI = 0.68 and MI = 0.62 (Figure). These results confirm that DI, which integrates both insulin sensitivity and secretion, more accurately captures the pathophysiologic basis of GDM than either index alone.

**Conclusion**: Although GDM is currently diagnosed based solely on maternal glucose levels, this study highlights the potential of disposition index as a tool for pathophysiologic subclassification. DI may better reflect the balance between insulin sensitivity and secretion in GDM, which could have implications for predicting perinatal outcomes or guiding individualized care.

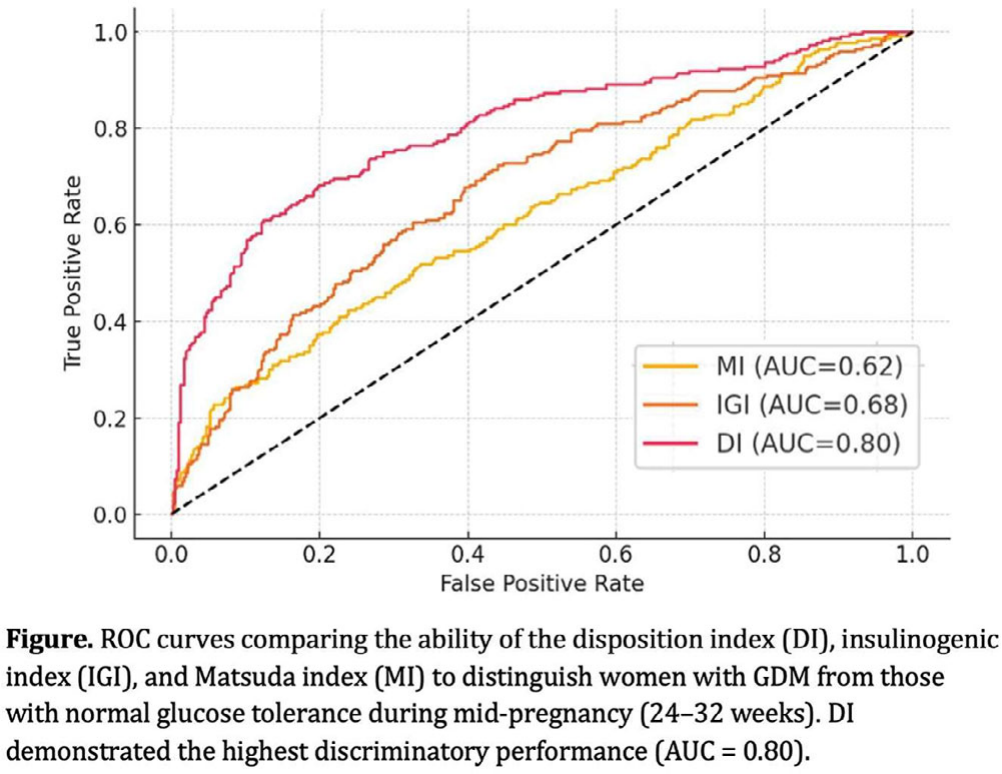

**3:30 PM – 5:00 PM**

## 1108 | **Diagnostic Outcomes of Sex Chromosome Abnormalities Detected on Noninvasive Prenatal Testing**

Ifeoma M. Ogamba‐Alphonso^1^; Annie Rozenblyum^2^; Xiwei Yang^1^; Teresa Dunn^3^; Nicole Cacace^1^; Martin Chavez^2^; Anju Suhag^4^

^1^NYU Langone Hospital ‐ Long Island, Mineola, NY; ^2^NYU Grossman Long Island School of Medicine, Mineola, NY; ^3^CytoGenX Medical Genetic Laboratories, Stony Brook, NY; ^4^NYU Langone Long Island, Mineola, NY

**Objective**: To evaluate the confirmation rate of sex chromosome abnormalities (SCA) including both sex chromosome aneuploidies and atypical sex chromosomes findings on noninvasive prenatal testing (NIPT).

**Study Design**: This retrospective cohort study included patients who underwent diagnostic testing following results of SCA on NIPT between March 2020 and June 2025. Exclusion criteria included incomplete records, contraindications to NIPT (e.g. organ or bone marrow transplant, hematological malignancy) and normal NIPT results. Medical records were reviewed for demographics, diagnostic results, and ultrasound findings. The primary outcome was the diagnostic confirmation rate, defined as the proportion of SCA on NIPT results that were subsequently confirmed by diagnostic testing (Chorionic Villi Sampling and/or Amniocentesis). Chi‐squared tests were used for categorical variables and Mann‐Whitney U test for continuous variables.

**Results**: Of 91 patients who met inclusion criteria, NIPT predicted the type of SCA in 49 (53.8%) cases, while 42 (46.2%) were not specified. Among those with a specified SCA type, the diagnostic confirmation rate was 29% for monosomy X, 67% for triple X, 80% for XXY, and 100% for XYY (*p* = 0.001). Among the unspecified SCA type, only 11 (26%) were confirmed on diagnostic testing. Maternal karyotype was more likely to be normal when the SCA was confirmed on diagnostic testing compared to when it was not (100% vs. 57%, *p* = 0.038). Maternal demographics and ultrasound findings did not differ significantly between groups.

**Conclusion**: The diagnostic confirmation rate for monosomy X was low, whereas other SCAs (XYY, Triple X, and XXY) were more frequently confirmed. The false positive cases of SCA were often linked to abnormal maternal chromosomes. These findings underscore the importance of offering diagnostic testing, including maternal karyotyping, and providing comprehensive counseling about the risk of false positives, especially in cases of monosomy X.

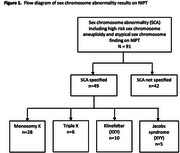

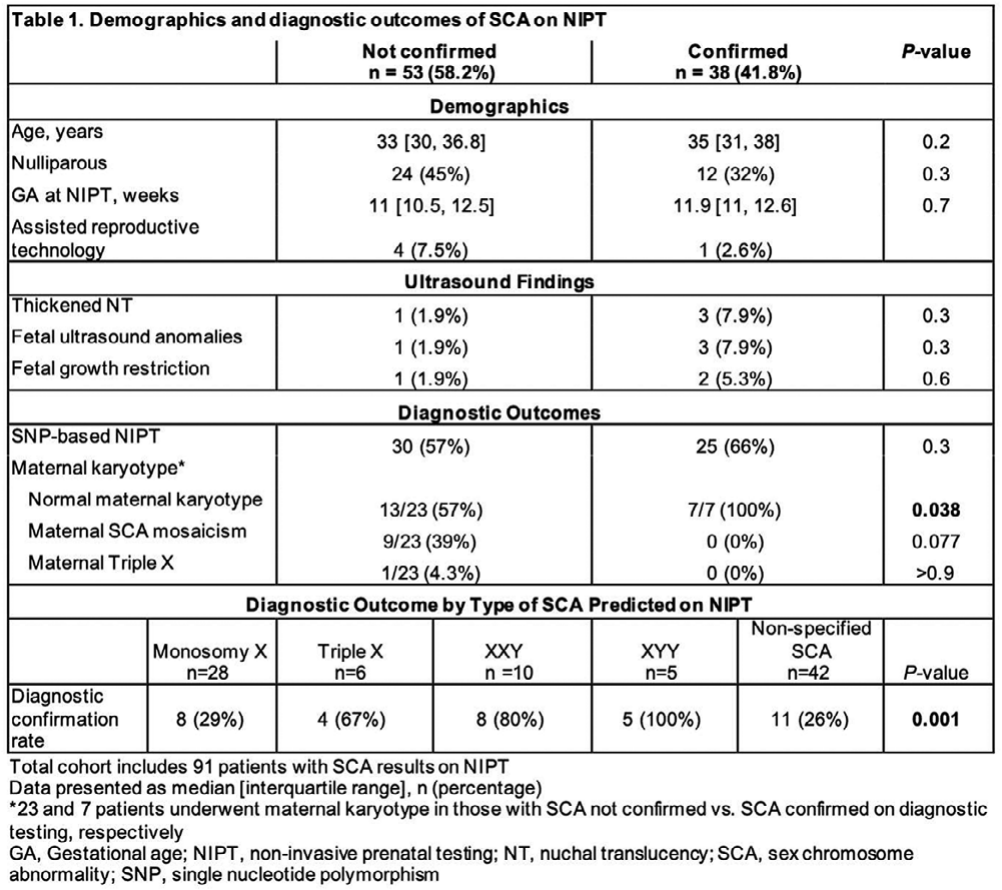

**3:30 PM – 5:00 PM**

## 1109 | **First Trimester Hemoglobin A1c Underestimates CGM‐Derived Average Glucose**

India Eaford^1^; Diana C. Soria‐Contreras^1^; Kyeisha Laurence^2^; Deepti Pant^1^; Lydia L. Shook^3^; Tanayott Thaweethai^1^; Rachel Petherbridge^1^; Veronica Tozzo^1^; John R. Higgins^1^; Camille E. Powe^4^

^1^Massachusetts General Hospital, Massachusetts General Hospital/Boston, MA; ^2^Harvard Medical School, Harvard Medical School/Boston, MA; ^3^Department of Obstetrics and Gynecology, MassGeneral Brigham; Division of Maternal‐Fetal Medicine; Harvard Medical School, Massachusetts General Hospital/Boston, MA; ^4^Massachusetts General Hospital, Boston, MA

**Objective**: Do 1^st^ trimester hemoglobin A1c (A1c) values reflect recent and preconception glucose levels measured by continuous glucose monitoring (CGM)?

**Study Design**: We conducted a retrospective study using laboratory and CGM data from pregnant individuals with pregestational diabetes (86.3% Type 1, Table 1). We included gravidas with an A1c measured at up to 14 weeks’ gestation and >33% CGM coverage in the 90 days before A1c. The primary outcome was the difference between average glucose from CGM (AG‐CGM) and the average glucose estimated from A1c (AG‐A1c) using the commonly used A1C‐Derived Average Glucose (ADAG) equation. Our primary analysis used a paired t‐test to compare the differences in AG‐CGM and AG‐A1c in the 1^st^ trimester vs. outside of pregnancy in those with available CGM and A1c data both in the 1^st^ trimester and while not pregnant (  >3 months prior to conception or >6 months postpartum). Secondary analysis compared AG‐CGM from data 90 days prior to conception to AG‐A1c in the 1^st^ trimester. In exploratory analyses, we used linear regression to examine associations between hemoglobin, pre‐pregnancy BMI, or gestational age at 1^st^ trimester A1c and the primary outcome.

**Results**: AG‐CGM prior to A1c was 14.8 mg/dL higher on average than AG‐A1c in the 1^st^  trimester (*n* = 26) vs. 7.3 mg/dL higher in the same individuals outside of pregnancy (mean pregnancy effect = 7.5 [95% CI 0.10–14.8] mg/dL, *p* = 0.047, Figure 1). AG‐CGM was 22.2 mg/dL higher on average prior to conception (*n* = 24) than AG‐A1c in the 1^st^ trimester vs. 6.9 mg/dL higher in the same individuals outside of pregnancy (mean effect = 15.3 [95% CI 6.5–24.2] mg/dL, *p* = 0.002). In exploratory analyses, there was no association between hemoglobin or pre‐pregnancy BMI and the difference between AG‐CGM and AG‐A1c. Later gestational age was associated with a lower difference between AG‐CGM and AG‐A1c (*n* = 51, β = −3.35 [95% CI −5.50, −1.20] mg/dl per week).

**Conclusion**: 1^st^ trimester A1c underestimates mean CGM glucose to a greater extent than outside of pregnancy; there is greater underestimation of preconception CGM glucose than recent CGM glucose.

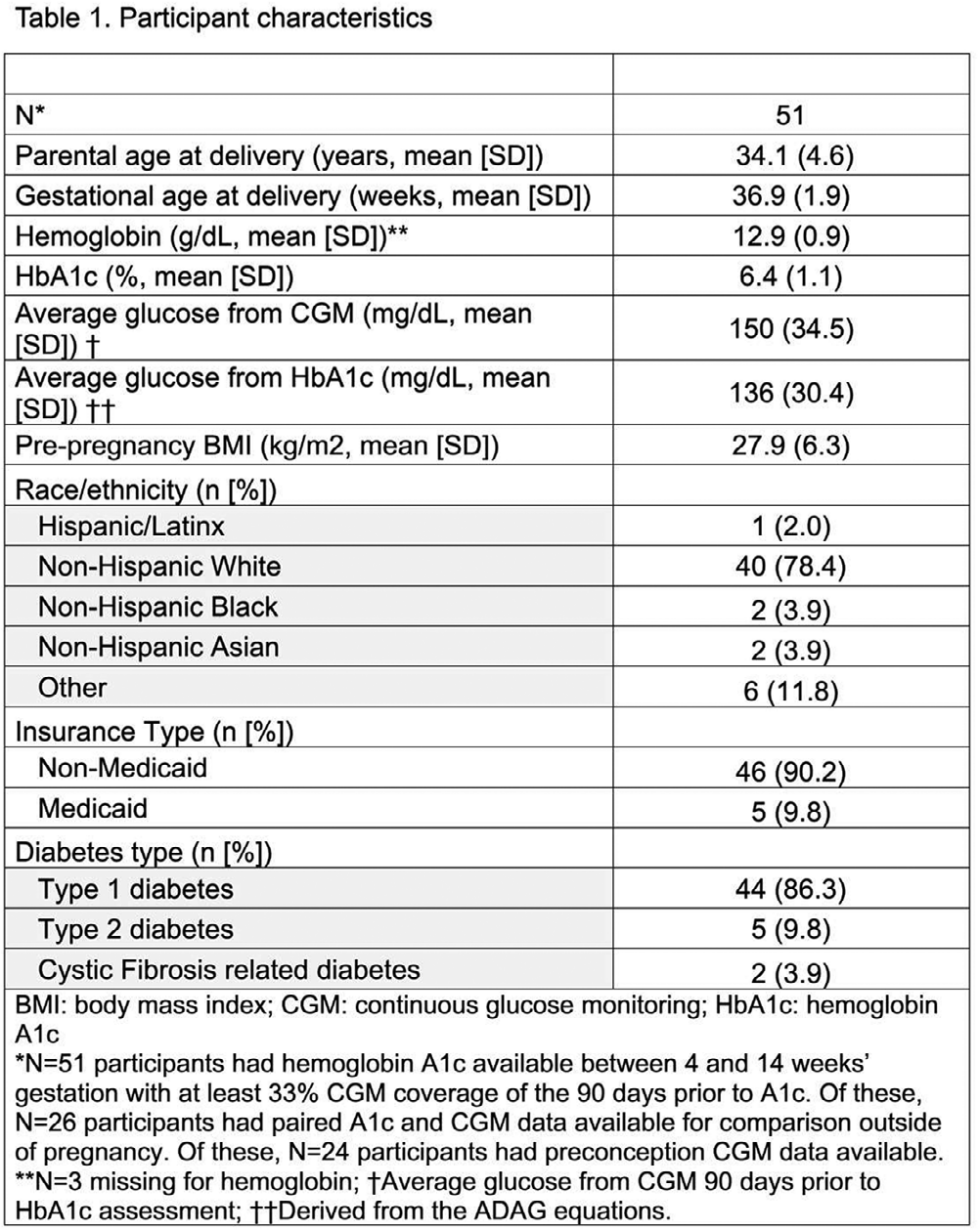

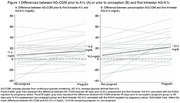

**3:30 PM – 5:00 PM**

## 1110 | **The Association of Mycoplasma Genitalium and Adverse Perinatal Outcomes in High‐Risk Pregnant Women**

Irene A. Stafford^1^; Erik Munson^2^; Sahana Kodali^3^; Mason Collie^3^; Han‐Yang M. Chen^1^; Sean C. Blackwell^1^; Damon Getman^4^

^1^Division of Maternal‐Fetal Medicine, Department of Obstetrics, Gynecology and Reproductive Sciences, McGovern Medical School at UTHealth Houston, Houston, TX; ^2^Deaprtment of Medical Laboratory Science, Marquette University, Milwaukee, WI; ^3^McGovern Medical School at UTHealth Houston, Houston, TX; ^4^Hologic, Inc, San Diego, CA

**Objective**: *Mycoplasma genitalium (M. genitalium)* is a known cause of sexually transmitted disease and genital tract pathology in women. Retrospective studies have linked the mycoplasmas to adverse maternal and neonatal outcomes. The primary aim of this prospective observational cohort study was to determine the association of *M. genitalium* with adverse perinatal outcomes in high‐risk pregnant women.

**Study Design**: After institutional approval, 500 pregnant patients with a history of pregnancy loss, cervical insufficiency (CI), or preterm birth (PTB) were enrolled between 07/23‐12/24. Vaginal swabs were tested for *M. genitalium* using the FDA‐cleared Aptima *M. genitalium* transcription‐mediated amplification (TMA) assay (Hologic Inc, San Diego, CA). Patients and providers were blinded to results prior to delivery. All obstetrical, neonatal, and infection‐related data were recorded. The primary outcome was a composite of adverse pregnancy outcomes including recurrent PTB, CI, or pregnancy loss. Secondary outcomes included two separate composites: one for adverse maternal outcomes (e.g., infectious morbidity) and another for adverse neonatal outcomes. Continuous variables were compared using t‐tests or Wilcoxon rank‐sum tests, and categorical variables with chi‐square or Fisher's exact tests. Adjusted relative risks (aRR) with 95% confidence intervals (CIs) were reported.

**Results**: Of the 500 patients enrolled, 490 (98%) had complete data. Fifty‐seven (11.6%) tested positive for *M. genitalium*. *M. genitalium* was significantly associated with prior PTB (*p* < 0.05). There was no significant difference in the composite adverse pregnancy outcome between groups (34.6% vs. 40.3%; aRR 0.80, 95% CI: 0.55–1.15) (Table 1), the composite adverse maternal outcome (7.1% vs. 14.0%; aRR 0.47, 95% CI: 0.18–1.26), or the composite neonatal outcome (45.5% vs. 42.9%; *p* = 0.72) (Table 2).

**Conclusion**: *M. genitalium* was not associated with recurrent adverse pregnancy outcomes, maternal morbidity, or adverse neonatal outcomes in this high‐risk pregnant cohort. These findings may help inform screening guidelines for pregnant populations.

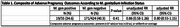

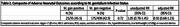

**3:30 PM – 5:00 PM**

## 1111 | **Amniotic Fluid Volume Estimation: A Novel Approach for an Old Problem**

Ceren Ünal^1^; Muhammet Atay Özten^2^; Işıl Ayhan^3^; Sebile Güler Çekiç^4^; Şeyma Hasköylü Şahin^4^; Alara Altıntaş^4^; Şevval Berfin Şahin^5^; Mert Turğal^6^; Serdar Aydın^5^; Ebru Çelik^6^

^1^Department of Obstetrics and Gynecology, Koç University Hospital, ISTANBUL, Istanbul; ^2^Department of Obstetrics and Gynecology, Division of Maternal‐Fetal Medicine, Koç University Hospital, Istanbul, Istanbul; ^3^Division of Maternal‐Fetal Medicine, Antalya City Hospital, Antalya, Antalya; ^4^Department of Obstetrics and Gynecology, Koç University Hospital, Istanbul, Istanbul; ^5^Department of Obstetrics and Gynecology, Koç University Hospital, Istanbul, Istanbul; ^6^Department of Obstetrics and Gynecology, Division of Maternal‐Fetal Medicine, Koç University Hospital, Istanbul, Istanbul

**Objective**: We aimed to quantitatively assess amniotic fluid volume(AFV) using 3D ultrasound, with accuracy determined by comparison to reference volumes obtained at cesarean delivery.

**Study Design**: In this prospective cohort of women undergoing third‐trimester ultrasound before elective cesarean, AFV was estimated by single deepest pocket (SDP), amniotic fluid index (AFI), and 3D four‐quadrant (3D4Q) techniques. Actual volumes were aspirated at delivery. Associations of AFI, SDP, and semi‐automated 3D A3D4Q with reference AFV were assessed using Spearman's correlation. Predictive formulas were derived via stepwise multiple linear regression using pocket volumes (Q1–Q4), mean Q, Qmax, AFI, and SDP; model fit used F‐ and t‐tests, and accuracy was examined by Bland–Altman analysis.

**Results**: 46 singleton pregnancies (median GA 38.4 weeks) were analyzed. Mean AFI was 12.2±4.5 cm, SDP 5.1±2.0 cm, true AFV 254±176 mL, and 3D4Q 220±155 mL. Mean Q (54±56 mL; 0–287 mL) correlated best with reference AFV (ρ = 0.88, *p* < 10^−1^⁴), followed by 3D4Q (ρ = 0.87) and Qmax (ρ = 0.76); conventional 2D indices had weaker correlation (SDP ρ = 0.62, AFI ρ = 0.55). Mean Q showed the highest correlation (ρ = 0.88, *p* < 1×10^−1^⁴), closely followed by the 3D4Q (ρ = 0.87, *p* < 1 × 10^−1^⁴). The Q max was also strongly related to true AFV (ρ = 0.76, *p* < 1 × 10^−^⁹). By contrast, conventional 2D indices had moderate associations‐ SDP: ρ = 0.62 (*p* ≈ 4×10^−^⁶), AFI: ρ = 0.55 (*p* ≈ 6×10^−^⁵). Multiple ordinary‐least‐squares regressions for candidate predictors are shown in Table 1, and the best fit was the formula comprising of measurements of all 4 quadrants and Qmax. Bland–Altman analysis showed minimal bias (∼–1 mL) for all models, with the KUGOAF‐Qmax yielding ±140 mL limits of agreement, the two‐parameter KUGOAF‐MeanQmax slightly narrower (±133 mL), and Qmax alone wider (±182 mL), indicating increased random error in the single‐predictor model.

**Conclusion**: The mean 3D volume of all quadrants had the strongest correlation with the real AFV, while 2D SDP and AFI had weaker correlations. Multi‐pocket formulas demonstrated clinically acceptable agreement within ±140 mL.

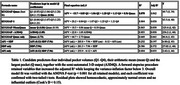

**3:30 PM – 5:00 PM**

## 1112 | **Prediction Model for Intrapartum Cesarean Delivery Among Women With Clinical Chorioamnionitis**

Itamar Gilboa^1^; Daniel Gabbai^1^; Dana Englander^1^; Yoav Baruch^2^; Yariv Yogev^3^; Emmanuel Attali^1^

^1^Lis Hospital for Women's Health, Tel Aviv Sourasky Medical Center, Lis Hospital for Women's Health, Tel Aviv Sourasky Medical Center, Tel Aviv; ^2^Lis Hospital for Women's Health, Tel Aviv Sourasky Medical Center, Tel‐Aviv, Tel Aviv; ^3^Lis Hospital for Women's Health, Tel Aviv Sourasky Medical Center Gray Faculty of Medicine, Tel Aviv University, Israel, Lis Hospital for Women's Health, Tel Aviv Sourasky Medical Center, Tel Aviv

**Objective**: Although prior studies have identified risk factors for cesarean delivery (CD) in women with clinical chorioamnionitis, no predictive model has been developed. Hence, we aimed to identify risk factors and construct a model for intrapartum CD in this population.

**Study Design**: A retrospective cohort study was conducted at a university‐affiliated tertiary center (2013–2024). The study included all singleton pregnancies ≥37 weeks undergoing a trial of labor and diagnosed with clinical chorioamnionitis, defined as intrapartum fever ≥38°C with administration of broad‐spectrum antibiotics. Exclusions: prior CD, multiple gestation, non‐viable fetuses, or CD without labor. Vaginal deliveries were compared to intrapartum CDs. Multivariable logistic regression identified predictors; model performance was evaluated using receiver operating characteristic (ROC) analysis.

**Results**: Among 119,034 deliveries during the study period, 2296 (1.9%) were diagnosed with clinical chorioamnionitis; of these, 272 (13.4%) underwent intrapartum CD.

1 Positive cultures: 8.8% in CD group vs. 8.7% in vaginal group (*p* = 0.966).

2. Risk factors for CD included: oxytocin use (OR 2.64, 95% CI 1.33–5.22), hemoglobin <10.5 g/dL (OR 2.07, 95% CI 1.23–3.48), nulliparity (OR 2.00, 95% CI 1.12–3.58), body mass index ≥25kg/m^2^ (OR 1.82, 95% CI 1.32–2.50), gestational age  ≥40 weeks (OR 1.81, 95% CI 1.31–2.50), age >35 years (OR 1.72, 95% CI 1.23–2.42), white blood cell count >16,000/µL (OR 1.53, 95% CI 1.13–2.07), meconium‐stained fluid (OR 1.45, 95% CI 1.06–1.98), gestational weight gain >15 kg (OR 1.37, 95% CI 1.02–1.85).

3. Epidural anesthesia was associated with a lower risk for CD (OR 0.21, 95% CI 0.14–0.31).

4. Main CD indications: arrest/cephalopelvic disproportion (44.1%), non‐reassuring fetal heart rate (40.8%).

5. Model performance: AUC 0.742 (95% CI 0.709–0.775; *p* < 0.001). Cutoff score of 10: 74% sensitivity, 63% specificity.

**Conclusion**: In clinical chorioamnionitis, several maternal and labor factors predicted intrapartum CD. This model supports risk stratification and may improve decision‐making.

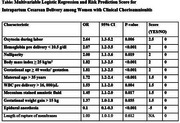

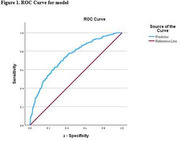

**3:30 PM – 5:00 PM**

## 1113 | **Placental Pathology Among Pregnant Individuals With Pregestational and Gestational Diabetes**

Jacklyn Locklear^1^; Shelley M. Stiltner^1^; Jill M. Maples^2^; Kimberly B. Fortner^3^; Nikki B. Zite^1^; Callie F. Reeder^4^; Jordan E. Lewis^1^; Rebecca Purvis^5^; Megan L. Young^6^; Kristina M. Carter^1^; Paul Eberts^1^

^1^University of Tennessee Graduate School of Medicine, Knoxville, TN; ^2^University of Tennessee Health Science Center COM Knoxville, Knoxville, TN; ^3^University of Tennessee College of Medicine‐ Knoxville, Knoxville, TN; ^4^UTHSC UTGSM Knoxville, Knoxville, TN; ^5^University of Tennessee Health Science Center College of Medicine‐ Knoxville, Knoxville, TN; ^6^University of Tennessee Medical Center, Knoxville, TN

**Objective**: There are many known placental pathology changes in pregnancies complicated by diabetes. While current data describe pathology changes in either gestational (GDM) or pregestational diabetics (PGDM), few compare placental pathology changes between the two groups. The aim of this study is to compare various pathological changes between patients with PGDM and GDM. We hypothesize that patients with PGDM will have increased maternal vascular lesions of the placenta compared to GDM.

**Study Design**: This retrospective study looked at the prevalence of abnormal placental pathology findings in patients that had PGDM and GDM.  Using electronic health record data from patients who delivered at our institution from January 2024‐ January 2025 (*N* = 4925), we evaluated placental pathology of women with GDM, type 1 diabetes and type 2 diabetes. Placentas were examined by a single board‐certified pathologist. Chi squared test was used to compare categorical variables between the groups.

**Results**: Of the total 4925 deliveries, 734 had diabetes and of those, 126 had placentas sent to pathology. There were no significant differences in demographics or covariates between the groups (Table). Villous fibrinoid necrosis was the most common finding in both PGDM and GDM. There were no significant changes in placental pathology between PGDM and GDM. Of the pathological changes we evaluated, only chorangiosis approached significance, being found more commonly among GDM (Figure 1).

**Conclusion**: Chorangiosis is a marker for increased concentration of capillaries in the chorionic villi, representing potential hypoxia in the placenta. A hypoxic environment for the placenta can lead not only to vascular changes but also epigenetic remodeling.  Future research focusing on the correlation between these pathologic findings and individual's metabolic profiles is planned.

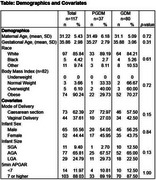

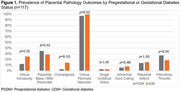

**3:30 PM – 5:00 PM**

## 1114 | **Effect of Aspirin and Early onset Fetal Growth Restriction Doppler Severity**

Jacqueline M. Powell^1^; Natalia Gontarczyk Uczkowski^1^; Scott Infusino^2^; Erin Bailey^1^; Amy Godecker^1^; J. Igor Iruretagoyena^1^

^1^University of Wisconsin School of Medicine and Public Health, Department of Obstetrics and Gynecology, Madison, WI; ^2^University of Wisconsin Hospitals and Clinics, Madison, WI

**Objective**: Early onset fetal growth restriction (FGR) is a diagnosis with significant impacts on neonatal outcomes driven by the need for preterm delivery as often is determined by Doppler severity. Preeclampsia is a known risk factor for placental insufficiency associated with early onset FGR. Given the widespread use aspirin to reduce risk of preterm preeclampsia, we aimed to determine if aspirin use was associated with a reduced risk of Doppler severity and progression of early onset FGR.

**Study Design**: We performed a retrospective cohort study at a single academic institution of patients with early onset FGR (<32 weeks) from 8/10/2016 to 4/17/2024. Chart review was performed to evaluate singleton, nonanomalous pregnancies with a diagnosis of early onset FGR and the association between aspirin use and gestational age at diagnosis, umbilical artery Doppler severity, and gestational age at delivery. The Mann‐Whitney test was used to compare the continuous variable. Categorical variables were compared using Fisher's exact test.

**Results**: A total of 255 patients were included in the study sample with the majority being non‐aspirin users (215 vs 40). Mean gestational age at diagnosis was 26.0 weeks for aspirin user compared to 26.5 for non‐aspirin users (*p* = 0.256). Aspirin use did not alter the severity of Dopplers at time of diagnosis, with the majority of patients having normal Dopplers. For those diagnosed at less than 28w, 92% of the patients on aspirin had normal umbilical artery Dopplers at diagnosis compared to 81.8% of the patients not taking aspirin (*p* = 0.181). There was no difference in rates of preterm delivery based on aspirin use, and the majority of patients delivered after 37w (67%).

**Conclusion**: Aspirin did not alter the severity of early onset FGR based on gestational age at diagnosis, severity of initial Doppler value, and gestational age at delivery. This is consistent with previous literature that aspirin is not effective at altering FGR outcomes or incidence.

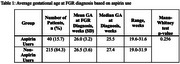

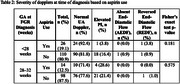

**3:30 PM – 5:00 PM**

## 1115 | **Exploring Patient Perspectives in the the PROMMO (Prelabor Rupture of Membranes: Misoprostol vs Oxytocin) Study**

Jacquelyn H. Adams^1^; Amy Godecker^2^; Kara K. Hoppe^2^

^1^University of Wisconsin School of Medicine and Public Health, Madison, WI; ^2^University of Wisconsin School of Medicine and Public Health, Department of Obstetrics and Gynecology, Madison, WI

**Objective**: Prelabor rupture of membranes (PROM) is common, yet optimal induction methods remain debated. We conducted a secondary analysis of the PROMMO trial which compared IV oxytocin (OT) to oral misoprostol (MP) for labor induction after PROM to examine participant experience and satisfaction with their allocated induction agent.

**Study Design**: This is a planned secondary analysis of a PROMMO, a randomized control trial in a single institution from 08/2020 to 05/2024. Study participants were allocated to oral MP or IV OT. Inclusion criteria included confirmed PROM, an unfavorable modified Bishop score (<7), gestational age 34w0d and 41w6d, and ability to consent in Spanish or English. We evaluated satisfaction using a modified 10‐item version of the Labor Agentry Scale and six unique questions with induction agent. All items were scored from 1 to 7, with 7 being the most positive. Statistical analysis included chi‐square or Fisher's exact tests and t‐test or Wilcoxon‐Rank Sum as appropriate.

**Results**: Of 134 trial participants, 122 completed postpartum surveys. The MP group included 60 and the OT group 62 participants. Maternal characteristics did not differ between groups. A Chronbach's alpha of 0.83 was calculated for the Labor Agentry Scale. Both groups had high median scores on the Labor Agentry Scale at 57/70 [IQR 50, 59.5] for MP and 57/70 [IQR 50, 61] for OT. There was no difference in patient satisfaction with method for MP or OT (7 [IQR 5,7  ] v 6 [IQR 4,7] *p* = 0.121). There was also no difference in feelings of convenience for MP or OT (7 [IQR 4,7  ] v 6 [IQR 4,7] *p* = 0.376). Both groups were likely to recommend their induction agent with high median scores for MP and OT (6 [IQR 4,7  ] v 6 [IQR 4,7] *p* = 0.974).

**Conclusion**: Participants in both the MP and OT groups had high median score on the modified Labor Agentry Scale and unique questions on patient satisfaction. The results of this survey demonstrate the patient experience with both MP and OT are satisfying for patients requiring labor induction in the setting of PROM.

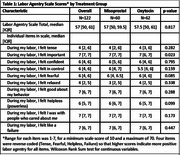

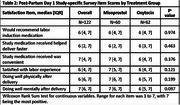

**3:30 PM – 5:00 PM**

## 1116 | **Previous Sexually Transmitted Infection as a Predictor for Maternal Syphilis Infection**

James D. Toppin^1^; Talia Suner^1^; Brian W. James^1^; Joseph R. Biggio, Jr.^2^; Frank B. Williams^3^

^1^Ochsner Clinic Foundation, New Orleans, LA; ^2^Ochsner Health, New Orleans, LA; ^3^Ochsner Clinic Foundation, Ochsner Clinic Foundation, LA

**Objective**: Syphilis infection in pregnancy and congenital syphilis rates have dramatically increased in recent years. Yet, there is little evidence to guide individualized screening protocols for high‐risk populations. Our aim was to determine if a history of a sexually transmitted infection (STI) predicts first‐time syphilis infection in pregnancy amid the current rise in congenital syphilis cases.

**Study Design**: We performed a retrospective cohort study of pregnancies receiving prenatal care in a multi‐center health‐system in the southern United States from January 2015 ‐ June 2024. Patients with prior or current syphilis were excluded. Groups were divided based upon STI history: previous STI before pregnancy or at initial prenatal visit vs no STI history. STIs included were laboratory‐confirmed chlamydia, gonorrhea, trichomoniasis, or HIV. The primary outcome was a syphilis diagnosis during pregnancy. Secondary outcome was congenital syphilis. Analysis was performed via Student's t and χ^2^ tests to calculate, *p* values, odds ratios (OR) with 95 % confidence intervals (CI).

**Results**: Among 57 543 eligible pregnancies, 7 144 (12.4 %) had prior STI exposure. Maternal age, parity, insurance status and gestational age at delivery showed statistically significant differences between groups (Table 1). Syphilis was newly identified in 258 pregnancies (0.45 %). Infection occurred in 1.4 % (97) of women with prior STI versus 0.3 % (161) of those without. Thus, an STI history increased the odds of antenatal syphilis (OR 4.3, 95 % CI 3.33–5.53; *p* < 0.001). Similarly, congenital syphilis frequency were increased, 0.00% vs 0.21% (OR 4.24, 95% CI 2.23‐8.05).

**Conclusion**: Any history of STI confers a four‐fold increase in the risk of syphilis acquisition during pregnancy. Incorporating prior STI history into early prenatal risk assessment could guide screening intervals, partner screening, and other targeted interventions to allow for early detection and treatment to help mitigate the growing burden of congenital syphilis.

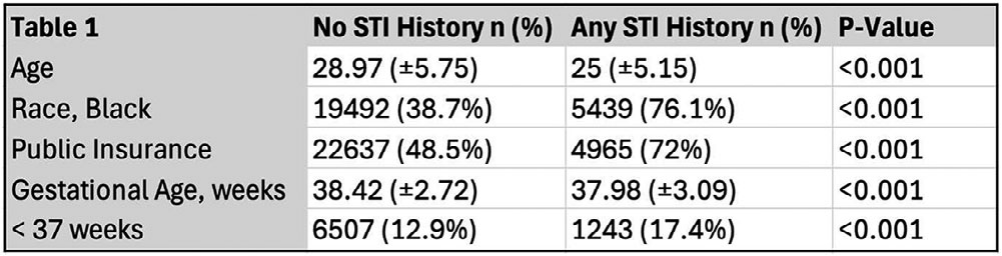

**3:30 PM – 5:00 PM**

## 1117 | **Noninvasive Fetal EEG Validation Using Auditory Event‐Related Potentials in Third‐Trimester Pregnancies**

Jasmine W. Jiang^1^; Emily Lee^2^; Juan Urrutia‐Gandolfo^3^; Molly Lucas^4^; Hayan Kwon^5^; Jose Cortes‐Briones^3^

^1^Yale School of Medicine, Yale School of Medicine, CT; ^2^Department of Obstetrics, Gynecology, and Reproductive Sciences, Yale School of Medicine, New Haven, CT; ^3^Department of Psychiatry, Yale School of Medicine, New Haven, CT; ^4^Wavelet Medical Inc., Brooklyn, NY; ^5^Department of Obstetrics and Gynecology, Yonsei University College of Medicine, Seoul, Seoul‐t'ukpyolsi

**Objective**: There are no clinically available tools for assessing fetal brain function in utero. Functional MRI and magnetoencephalography can detect fetal brain activity but require costly, non‐portable equipment and magnetically shielded rooms. We developed the first system capable of noninvasively extracting fetal electroencephalography (EEG) signals from the maternal abdomen. EEG is portable, low‐cost, and widely available, but until now, fetal EEG could not be reliably measured noninvasively.

**Study Design**: We collected abdominal EEG during non‐stress tests (NSTs) in third‐trimester pregnancies. Recordings lasted 20 minutes with randomly timed 85 dB auditory stimuli to elicit fetal neurologic responses. Our proprietary algorithm filtered non‐fetal signals and extracted fetal EEG responses. We analyzed event‐related potentials (ERPs) by examining spatial distribution, latency, and associations with gestational age and birth outcomes. Statistical significance was assessed using a nonparametric permutation test that controls for multiple comparisons.

**Results**: Fetal EEG was collected from 97 singleton pregnancies (29–39 weeks gestation). Maternal BMI ranged from 15.8–36 kg/m^2^, and maternal age from 26–47 years. All subjects had reactive NSTs. Fetal presentations included cephalic (78), breech (14), and transverse (5). ERPs were successfully extracted from all 97 fetuses. ERP heatmaps were generated for each subject, and a grand average waveform confirmed a consistent response (Figure 1). A statistically significant ERP signal was observed during the 150–800 ms post‐stimulus interval (Figure 2).

**Conclusion**: This is the first large‐scale demonstration of in utero fetal brain responses to auditory stimuli using noninvasive EEG. These findings establish the feasibility of real‐time fetal brain monitoring in clinical settings.

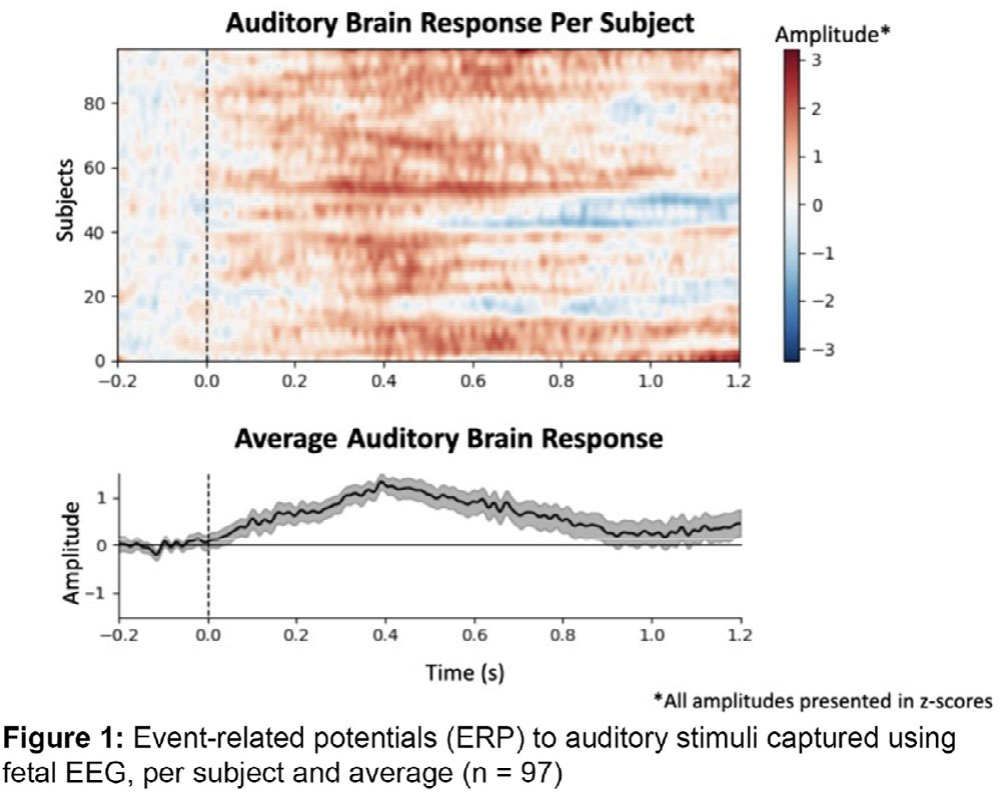

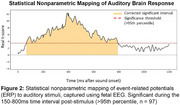

**3:30 PM – 5:00 PM**

## 1118 | **Modifiable Clinician‐Level Factors Associated With TOLAC Counseling and Management**

Jennifer Choi^1^; Rakiya Watts^2^; Maria Fisher^2^; Heidi Preis^3^

^1^Stony Brook University Hospital, Commack, NY; ^2^Stony Brook University Hospital, Stony Brook, NY; ^3^Stony Brook University, Stony Brook, NY

**Objective**: Identify modifiable clinician‐level factors associated with practice behaviors on counseling for trial of labor after cesarean (TOLAC) and management of TOLAC cases (vaginal birth after cesarean; VBAC success).

**Study Design**: Cross‐sectional survey‐based study among New York State obstetric providers (Sept 2024‐March 2025). Clinician factors included sociodemographics, professional background, childbirth beliefs, and litigation concerns. Outcomes included TOLAC recommendation frequency in low‐ and higher risk‐scenarios and TOLAC management in clinical vignettes with category II tracing randomized by race.

**Results**: The sample included 107 obstetric providers: 44 attendings (41.1%), 35 midwives (32.7%), and 28 residents/fellows (26.2%). Demographics were not associated with TOLAC counseling, but profession, specialty, employment setting, and delivery site were (Table 1). Midwives and those in academic centers recommended TOLAC more often across scenarios (*p* < 0.001); MFM more often in higher‐risk scenarios (*p* = 0.04). Stronger beliefs that birth is natural were linked to more frequent TOLAC counseling while viewing it as medical were linked to less (Table 2). Greater litigation concerns were associated with less frequent TOLAC recommendations in higher‐risk scenarios.

Overall, 24.5% managed TOLAC vignettes to VBAC. Management was similar across demographics, but hospital‐ or community‐employed providers (30.1%) were more likely than private/self‐employed (4.3%) to manage for VBAC (*p* = 0.01). Perceived institutional support was linked to higher VBAC rates (29.4% vs. 4.8%, *p* = 0.02). Providers were less likely to manage for VBAC when the vignette patient was Black (14.8%) vs. White (34.6%) (*p* = 0.02).

**Conclusion**: Provider profession, beliefs, and malpractice concerns influenced TOLAC counseling, particularly in higher risk cases. Institutional context and racial bias in clinical‐decision making was evident. Findings underscore the need for interventions including bias training, standardized counseling, and supportive institutional policies to improve equity and outcomes.

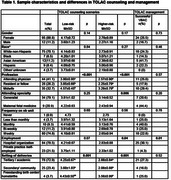

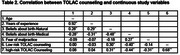

**3:30 PM – 5:00 PM**

## 1119 | **Comparing Engagement Among Postpartum Individuals With Public Versus Private Insurance in Remote Hypertension Monitoring Program**

Jennifer Okunbor^1^; Mary Marchese^1^; Allison K. Mak^1^; Rose Mahoney^1^; Scott Machado^1^; Tracy L. Jackson^1^; Nicole Konecke^1^; William Hunt^1^; Danielle Simmons^1^; Maria Mejia Castillo^1^; Methodius G. Tuuli^2^; Adam K. Lewkowitz^1^; Alisse Hauspurg^3^

^1^Women & Infants Hospital of Rhode Island / Warren Alpert Medical School of Brown University, Providence, RI; ^2^Department of Obstetrics and Gynecology, Warren Alpert Medical School at Brown University, and Women & Infants Hospital of Rhode Island, Providence, RI; ^3^Brown University, Providence, RI

**Objective**: Engagement in remote self‐measured blood pressure (SMBP) monitoring programs reduce rates of hypertension (HTN)‐related hospital readmission, emergency department (ED) visits and severe maternal morbidity (SMM).  Our aim is to examine whether insurance type is associated with differences in engagement and outcomes among SMBP program participants.

**Study Design**: Patients with hypertensive disorders of pregnancy (HDP) are provided BP cuffs and remote clinical support from a bilingual community health worker (CHW) and nurse practitioner for 6 weeks after delivery. For this analysis, participants were stratified by insurance type at enrollment: public (Medicare/Medicaid) vs private insurance. The primary outcome was engagement in the program, defined as submission of ≥1 BP within 10 days of discharge per ACOG guidelines. Secondary outcomes included total number of BP reported, a composite of HTN‐related readmission or ED presentation, frequency of SMM, and remote anti‐hypertensive medication initiation. A generalized linear model estimated relative risks (RR) after adjustment for confounding factors.

**Results**: Among 2623 participants enrolled in the SMBP program, 44.7% were publicly insured. Compared to those with private insurance, publicly insured participants were younger, less likely to identify as White, more likely to have chronic hypertension with superimposed preeclampsia.  After adjusting for these differences, engagement in the SMBP program was significantly lower in the publicly insured group compared to the privately insured group [(37.4% versus 55.8%; aRR 0.78 (0.71, 0.85)]. Publicly insured patients sent on average 4 fewer blood pressures compared to privately insured patients [11.8 (12.6) vs 19.1 (16.7); adjusted β ‐4.31 (‐6.08, ‐2.53)]. There were no differences in hypertension‐related ED visits, readmissions, or SMM between groups.

**Conclusion**: Engagement with the SMBP program was lower among publicly insured patients compared to privately insured patients. Our findings suggest the need to explore interventions in publicly insured postpartum people to support participation in these programs.

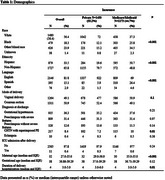

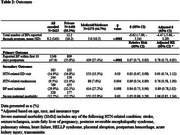

**3:30 PM – 5:00 PM**

## 1120 | **Screening on Arrival: A Decision Analysis of Universal Syphilis Screening in Pregnant Emergency Department Patients**

Jennifer Qin; Leah M. Savitsky; Catherine M. Albright

University of Washington, Seattle, WA

**Objective**: Congenital syphilis is rising sharply in the United States, with many affected birth parents receiving inadequate prenatal care prior to delivery. A recent study demonstrated universal, opt‐out syphilis screening in the emergency department (ED) identifies a substantial burden of previously undiagnosed infection. Because ED encounters may be the only point of care for some patients, we evaluated the optimal strategy of syphilis screening for pregnant patients presenting to the ED.

**Study Design**: We constructed a Markov decision model comparing universal ED screening with testing only when clinically indicated, with a base case maternal syphilis prevalence of 700/100,000 for an urban ED population. Up to four additional ED visits were modeled if the patient remained untreated and undelivered. The primary outcome was cost to prevent a case of congenital syphilis, with secondary outcomes including intrauterine fetal demise (IUFD), neonatal demise, and cases of treated syphilis. Some patients were assumed to seek routine prenatal care following an ED visit and receive testing and treatment in that setting. One‐way and probabilistic sensitivity analyses were used to assess the robustness of conclusions.

**Results**: In a theoretical cohort of 100,000 patients, universal screening for syphilis in the ED is expected to marginally decrease overall costs, identify and treat more pregnant patients with syphilis, and decrease the incidence of congenital syphilis and perinatal loss. The theoretical cost savings of preventing one case of congenital syphilis was $549,470. This strategy remains cost‐saving until the prevalence of syphilis decreases to a rate less than 10.2/100,000 people. The model was sensitive to the probability of syphilis in the population, the probability that a patient completes treatment, and the likelihood of IUFD in the setting of congenital syphilis.

**Conclusion**: Universal ED screening is a cost‐saving strategy to decrease the incidence of congenital syphilis. Given the US national maternal syphilis rate of 280/100,000 births, this strategy should be strongly considered.

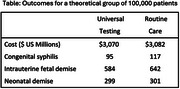

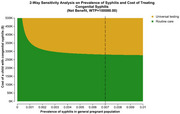

**3:30 PM – 5:00 PM**

## 1121 | **Decreasing Trends of Tobacco Use Disorder Among Pregnant People in the United States**

Jennifer Yao^1^; Angelina Lim^2^; Aoi Yamaguchi^3^; Sawa Keymeulen^4^; Joseph G. Ouzounian^5^; Koji Matsuo^6^

^1^University of Southern California, Keck School of Medicine, Los Angeles, CA; ^2^LAGMC + USC OBGYN, Los Angeles, CA; ^3^Osaka University School of Medicine, Osaka University School of Medicine, Osaka; ^4^Department of Obstetrics and Gynecology, Los Angeles General Medical Center, Los Angeles, CA; ^5^Division of Maternal‐Fetal Medicine, Department of Obstetrics and Gynecology, University of Southern California, Los Angeles, CA; ^6^Division of Gynecologic Oncology, Department of Obstetrics and Gynecology, University of Southern California, Los Angeles, CA

**Objective**: A recent analysis of the Centers for Disease Control and Prevention's National Vital Statistics System found that pregnant people with tobacco use disorder has decreased in the United States. To corroborate their findings in a different dataset, this study assessed the temporal trends of pregnant people with tobacco use disorder in the United States Agency for Healthcare Research and Quality's dataset.

**Study Design**: The cross‐sectional study queried the Healthcare Cost and Utilization Project's National Inpatient Sample, including 24,976,627 hospital deliveries from 2016 to 2022. The prevalence of tobacco use disorder among pregnant people was summarized for each year. A multivariable generalized linear model was constructed to assess temporal trends, adjusted for maternal age, race/ethnicity, primary payer, census‐level median household income, region, other substance use disorders, and mental health disorders.

**Results**: A total of 1,237,415 (5.0%) hospital deliveries had a diagnosis of tobacco use disorder. The annual prevalence of tobacco use disorder decreased by 28%, from 5.5% in 2016 to 4.0% in 2022 (adjusted rate ratio [aRR] for 2022 vs. 2016, 0.72; 95% CI, 0.71–0.72). The relative‐decrease from 2016‐2022 in tobacco use disorder was particularly high among younger people (age <25, 7.1% to 4.7%, aRR 0.63, 95%CI 0.62‐0.64; and age 25‐29, 6.3% to 4.3%, aRR 0.67, 95%CI 0.67‐0.68), those residing in regions including New England (5.2% to 3.2%, aRR 0.67, 95%CI 0.65‐0.70), the Mid‐Atlantic (4.9% to 3.3%, aRR 0.65, 95%CI 0.64‐0.66), and the East North Central region (8.4% to 6.0%, aRR 0.68‐0.70), and those with mental health conditions (anxiety disorder, 15.9% to 8.5%, aRR 0.62, 95%CI 0.61‐0.64; and those with depressive disorder, 16.4% to 9.6%, aRR 0.66, 95%CI 0.65‐0.68) (Table).

**Conclusion**: This national‐level assessment corroborated a prior study showing that pregnant people with tobacco use disorder has decreased in the United States. A near 30% decrease over a 7‐year period is significant and promising.

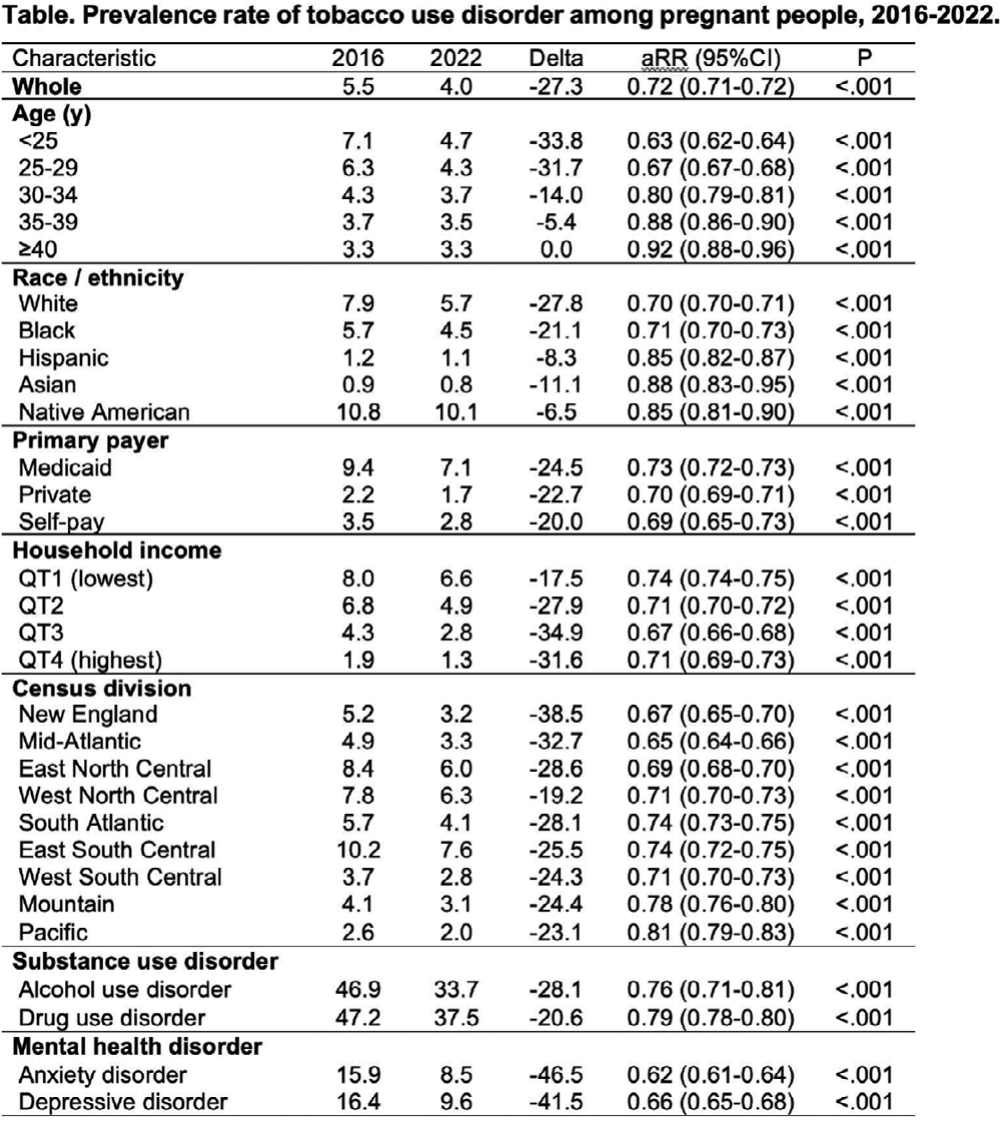

**3:30 PM – 5:00 PM**

## 1122 | **A Quality Improvement Initiative to Standardize Cord Blood Gas Collection for High‐Risk Deliveries**

Jessica S. Chung^1^; Andrea Rebisch^2^; Raven Blockwitz^2^; Isabella T. Eiler^3^; Susan Cohen^4^; Anna Palatnik^5^; Erika Peterson^1^; Brock E. Polnaszek^1^

^1^Medical College of Wisconsin, Milwaukee, WI; ^2^Froedtert Hospital, Milwaukee, WI; ^3^Indiana University School of Medicine, Indianapolis, IN; ^4^Children's Hospital of Wisconsin, Milwaukee, WI; ^5^Medical College of Wisconsin ‐ Milwaukee, WI, Milwaukee, WI

**Objective**: Umbilical cord gases are often essential for guiding neonatal management, yet national and international guidance about when and how to obtain this blood test remains variable. We aimed to improve cord gas collection rates in NICU‐attended deliveries, due to their inherent higher risk factors, with overall collection assessed as a secondary outcome.

**Study Design**: At a tertiary academic center, a multidisciplinary quality improvement (QI) initiative was implemented in December 2024 to standardize umbilical cord blood gas collection. Interventions included clinician education, training on processing and measuring, as well as audit and feedback cycles. Monthly cord gas collection metrics were tracked from September 2024 to July 2025 and are on‐going. Pre‐implementation (Sep–Nov 2024) and post‐implementation (Dec 2024–Jul 2025) rates were compared using independent t‐tests.

**Results**: Cord gas collection in NICU‐attended deliveries increased from 51% to 92% between pre‐ and post‐implementation periods (*p* < 0.001). Overall collection rates rose from 29% to 55% reflecting a 25.8‐point improvement (*p* < 0.001) (Table and Figure 1). We saw a rapid improvement sustained across eight months, and it was accompanied by strong stakeholder buy‐in. Improvements were quick, sustained across eight months, and accompanied by positive interdisciplinary feedback.

**Conclusion**: This QI project significantly and sustainably improved cord gas collection in high‐risk deliveries. These findings directly support the 2025 American Academy of Pediatrics recommendation to use postnatal cord blood sampling in neonates requiring admission and demonstrate how obstetric‐led implementation can be synergistic with neonatology priorities.

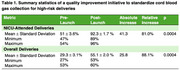

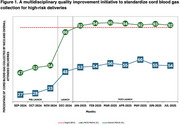

**3:30 PM – 5:00 PM**

## 1123 | **Maternal Outcomes in Forceps‐Assisted Cesarean Delivery**

Jessica D. Sisco^1^; Alesha M. White^2^; Jessica E. Pruszynski^1^; Elaine L. Duryea^1^

^1^University of Texas Southwestern Medical Center, Dallas, TX; ^2^University of Texas Southwestern Medical Center, Grand Prairie, TX

**Objective**: Maternal outcomes in cases of operative vaginal deliveries are well established. The purpose is to examine maternal outcomes in forceps‐assisted cesarean deliveries (FACD).

**Study Design**: This is a retrospective cohort study of patients delivering liveborn, singleton neonates via FACD at a single institution between January 2014 and December 2023. Patients were compared to a 1:1 random sampling of patients delivered via CD without forceps during the same study period. Maternal demographics and delivery characteristics were examined. A logistic regression adjusting for stage of labor prior to CD, indication for CD and hypertensive disorders of pregnancy was performed.

**Results**: 2021 patients were included in each cohort.  FACDs were more likely to be performed in multiparous, non‐laboring patients via low transverse hysterotomy undergoing repeat CD (all *p* < 0.001). They were less likely to have gestational hypertension (18% vs 24%, *p* < 0.001) and pre‐eclampsia (9% vs 14%, *p* < 0.001) but more likely to have overt diabetes (6% vs 4%, *p* = 0.002) (Table 1). In terms of delivery outcomes, FACDs were associated with longer extraction times (3 minutes (2‐4) vs 1 minute (1‐2), *p* < 0.001), but less likely to develop chorioamnionitis, require blood transfusion, or to undergo hysterectomy (all *p* < 0.05). No significant difference was found in rates of postpartum hemorrhage or in the overall number of products transfused. After logistic regression, only rate of hysterectomy remained significant (Table 2).

**Conclusion**: Though associated with longer extraction times, cesarean forceps were not associated with increased adverse maternal outcomes. In low‐risk CDs, forceps are reasonable tools to use to assist with delivery.

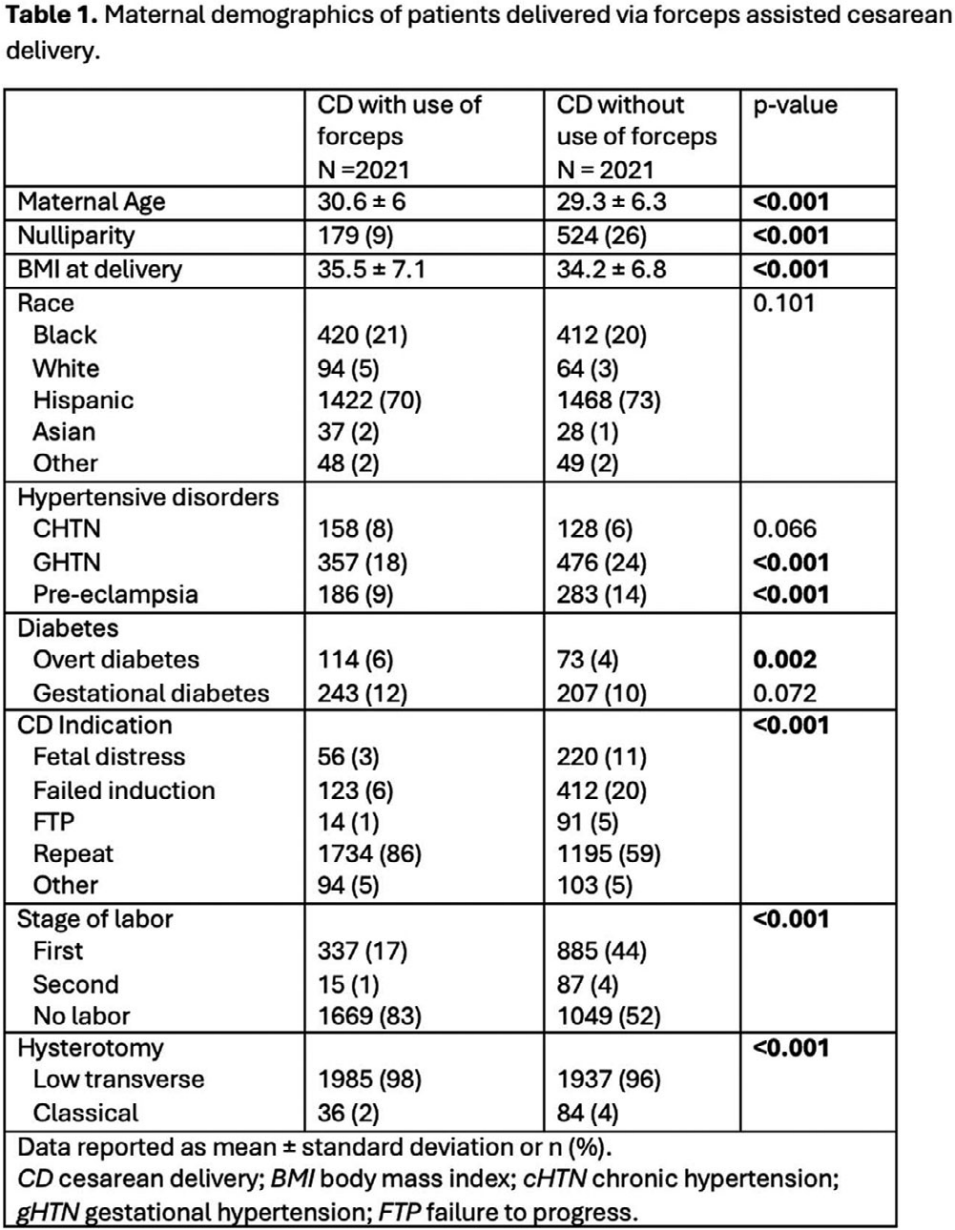

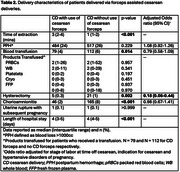

**3:30 PM – 5:00 PM**

## 1124 | **Preterm vs Full‐Term Epigenetic Age Acceleration Patterns Vary by Chorionicity in Twin Pregnancy**

Ji Hoi Kim^1^; Leejae Cho^2^; Jong Kwan Jun^3^; Jong‐Lyul Park^4^; Hwan Young Lee^2^; Seung Mi Lee^1^

^1^Department of Obstetrics and Gynecology, Seoul National University College of Medicine, Seoul, Seoul‐t'ukpyolsi; ^2^Department of Forensic Medicine, Seoul National University College of Medicine, Seoul, Seoul‐t'ukpyolsi; ^3^Department of Obstetrics and Gynecology, Ewha Womans University, Seoul, Seoul‐t'ukpyolsi; ^4^Aging Convergence Research Center, KRIBB, Daejeon, Taejon‐jikhalsi

**Objective**: Epigenetic age acceleration (EAA) is the residual from regressing DNA methylation‐based gestational age (GA) on actual GA, reflecting differences in biological aging rates. This study assessed developmental differences in preterm birth by measuring EAA using DNA methylation clocks. We compared epigenetic differences by chorionicity in monozygotic twins to examine the link between gestational age at delivery and EAA.

**Study Design**: DNA methylation and clinical data were obtained from cord blood samples of 102 monozygotic twins at our center. Among them, 26 pairs and 2 individuals from dichorionic diamniotic (DCDA) pregnancies (19 full‐term, 7 preterm and 2 individuals preterm) and 24 pairs from monochorionic diamniotic (MCDA) pregnancies (14 full‐term, 10 preterm). Zygosity was determined with sex of neonates, chorionicity, and DNA analysis. Methylation profiling was done using the Illumina EPIC v2.0 microarray. Samples were classified as preterm (<37 weeks) or full‐term (≥ 37 weeks). DNA methylation gestational age was estimated to be using Knight GA clock and Bohlin GA clock. EAA was calculated as the residuals from regressing of DNAm GA on actual GA. Differences in EAA between preterm and full‐term were analyzed by t‐tests.

**Results**: Preterm infants showed high EAA than full‐term in both chorionicity, with significance observed using Knight GA clock (*p* = 0.033). In DCDA twins, full‐term infants had low EAA while preterm showed high EAA(Knight GA clock, *p* = 0.024), whereas MCDA twins exhibited high EAA regardless of gestational age (Knight GA clock, *p* = 0.65). We examined clinical factors that might explain the EAA pattern in MCDA full‐term infants but found no differences between DCDA and MCDA groups, except for maternal age, height, and body mass index.

**Conclusion**: EAA patterns differed between preterm and full‐term infants and were significantly influenced by chorionicity. MCDA twins exhibited high EAA regardless of gestational age, suggesting that sharing placenta may cause physiological stress even at full term. The highlight the role of chorionicity in interpreting gestational age and development.

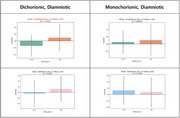

**3:30 PM – 5:00 PM**

## 1125 | **Early Initiation of Low‐Dose Aspirin Improves Outcomes in Women With Moderate‐Risk Factors for Preeclampsia**

Ji Yeon Lee^1^; Hee Young Cho, N/A^2^; Nari Kim^3^; Hyun Mee Ryu^4^

^1^CHA Bundang Medical Center, CHA University School of Medicine, CHA Bundang Medical Center, CHA University School of Medicine / Seongnam, Kyonggi‐do; ^2^Obstetrics and Gynecology, Seoul National University College of Medicine, Seoul, Kyonggi‐do; ^3^CHA Bundang Medical Center, CHA University School of Medicine, CHA Bundang Medical Center / Seongnam, Kyonggi‐do; ^4^CHA Bundang Medical Center, CHA University School of Medicine, CHA Bundang Medical Center/ Seongnam, Kyonggi‐do

**Objective**: Preeclampsia is thought to originate from abnormal placentation during implantation. Initiating low‐dose aspirin closer to this window may improve its preventive effect. Current guidelines recommend starting aspirin at 12 weeks due to limited data supporting earlier use. This study aimed to assess whether starting low‐dose aspirin before 12 weeks improves maternal and neonatal outcomes compared to starting between 12–20 weeks in women with multiple moderate‐risk factors for preeclampsia.

**Study Design**: This retrospective cohort study included singleton pregnancies between 2012 and 2025. Eligible women had ≥2 moderate‐risk factors for preeclampsia and were prescribed daily low‐dose aspirin, delivering at ≥20 weeks at our hospital. Women with high‐risk factors (e.g., prior preeclampsia, multifetal gestation, chronic hypertension, diabetes, renal or autoimmune disease) were excluded. Subjects were grouped by aspirin initiation timing: early (<12 weeks, *n* = 1,802) vs. late (12–20 weeks, *n* = 473). Multivariate analyses adjusted for maternal characteristics.

**Results**: The early group had higher rates of nulliparity (77.0% vs. 66.0%) and IVF (77.4% vs. 39.1%). Compared to the late group, the early group had lower adjusted risks for gestational hypertension (aOR 0.33, 95% CI 0.21–0.52), preeclampsia (aOR 0.30, 95% CI 0.17–0.54), gestational diabetes (aOR 0.57, 95% CI 0.40–0.80), and preterm birth <32 weeks (aOR 0.04, 95% CI 0.01–0.10). Neonatal outcomes were also improved: lower risks of 5‐min Apgar <7 (aOR 0.26, 95% CI 0.12–0.55), NICU admission (aOR 0.52, 95% CI 0.40–0.66), bronchopulmonary dysplasia (aOR 0.08, 95% CI 0.02–0.30), jaundice (aOR 0.68, 95% CI 0.53–0.87), patent ductus arteriosus (aOR 0.35, 95% CI 0.22–0.57), and retinopathy of prematurity (aOR 0.41, 95% CI 0.27–0.63).

**Conclusion**: Starting aspirin before 12 weeks in moderate‐risk pregnancies significantly reduced adverse maternal and neonatal outcomes. These results support revising guidelines to align aspirin use with the timing of placentation.

**3:30 PM – 5:00 PM**

## 1126 | **The Association Between Rural Setting and Adverse Perinatal Outcomes Among Teens**

Jill K. Kumasaka^1^; Bharti Garg^2^; Amelia H. Gagliuso^3^; Aaron B. Caughey^2^

^1^Oregon Health & Science University, Portland, OR; ^2^Oregon Health & Science University, Oregon Health & Science University, OR; ^3^Oregon Health and Science University, Portland, OR

**Objective**: Rural communities face a variety of challenges that contribute to higher maternal morbidity and mortality compared to pregnancies in urban settings. While research has shown that teens experience worse perinatal outcomes than their adult counterparts, few studies report on teens in rural settings. This study aimed to evaluate rural adolescents’ perinatal outcomes as compared to urban adolescents as well as compared to rural adults.

**Study Design**: This is a retrospective cohort study of California's linked vital statistics and hospital discharge data (2008‐2020). We included 13‐50 years old with singleton, non‐anomalous births delivered between 23‐42 weeks. Patients were stratified by rural or urban setting, which was determined using patient's residential zip code. We further categorized patients into adolescents or teens (13‐18 years old) and adults (19‐50 years old). Two separate analyses were performed. First, we compared rural adolescents with urban adolescents and then we compared rural adolescents with rural adults. Chi‐square tests and Multivariable Poisson regression analyses were used to determine risk of maternal and neonatal outcomes among rural adolescents.

**Results**: We included 402,608 births, of which 44% (*n* = 177,015) were adolescents (6.9% rural adolescents and 93.1% urban adolescents). When compared to urban adolescents, rural adolescent pregnancies had significantly increased risk of postpartum hemorrhage (aRR = 1.24, 95% CI: 1.13‐1.36).  Rural adolescents had lower risk of preterm delivery <37 weeks when compared to urban adolescents (aRR = 0.89, 95% CI: 0.81‐0.99), and rural adults (aRR = 0.86, 95% CI: 0.75‐0.98). Neonates born to rural adolescents had significantly higher risk of infant death when compared to urban adolescents (1.51, 95% CI: 1.13‐2.02) and rural adults (aRR = 1.43, 95% CI: 1.04‐1.95).

**Conclusion**: Clinicians and public health officials should be aware that both location and age may influence perinatal outcomes. Further research to elucidate how rural settings and age influence perinatal complications will be important in identifying effective interventions.

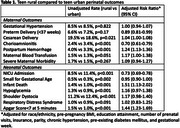

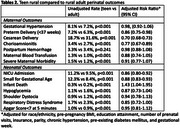

**3:30 PM – 5:00 PM**

## 1127 | **Assessing the Accuracy and Safety of ChatGPT Responses to Common Questions About Syphilis in Pregnancy**

Joe Haydamous^1^; Laura Diab^2^; Analuisa C. Mosqueda^2^; Sabrina C. DaCosta^2^; Irene A. Stafford^2^

^1^Department of Obstetrics, Gynecology and Reproductive Sciences, McGovern Medical School at UTHealth Houston, Department of Obstetrics and Gynecology, McGovern Medical School at UT Health, Houston, TX; ^2^Division of Maternal‐Fetal Medicine, Department of Obstetrics, Gynecology and Reproductive Sciences, McGovern Medical School at UTHealth Houston, Houston, TX

**Objective**: This study evaluated the accuracy, completeness, and safety of ChatGPT‐generated responses to common questions about syphilis in pregnancy, aiming to assess its role as an educational and triage‐support tool in obstetric care.

**Study Design**: Clinically relevant questions were compiled using CDC, ACOG, and WHO guidelines, as well as topics frequently discussed in patient forums. Responses were generated using GPT‐4 and evaluated by six independent experts in obstetrics, maternal‐fetal medicine, and infectious disease. Reviewers rated each response on a 5‐point Likert scale across three domains: accuracy, completeness, and safety. Each question was submitted using one of two standardized prompts simulating either patient or provider communication styles. The complete list of questions and prompts appears in Figure 1. Questions were grouped into four categories: General Knowledge, Public Health/Prevention, Treatment, and Diagnostic Interpretation.

**Results**: ChatGPT responses showed high performance across all domains, with mean scores of 4.38 for accuracy, 4.49 for completeness, and 4.47 for safety. Between 88% and 92% of evaluations received scores of 4 or higher. Public Health/Prevention questions achieved the highest overall scores, averaging 4.67 or above in each domain. General Knowledge items also performed well, especially in safety (4.79) and completeness (4.75). Treatment‐related responses maintained strong ratings across all categories (≥4.33), showing good guideline alignment. Although Diagnostic Interpretation responses were accurate (4.33) and safe (4.50), completeness was lower (4.17), with reviewers noting missing nuance in complex cases. Importantly, no unsafe or misleading content was identified by any reviewer.

**Conclusion**: ChatGPT produced safe, accurate, and generally complete responses to syphilis‐in‐pregnancy questions. Its strong performance in public health and general education supports cautious integration into prenatal counseling workflows. Enhancing diagnostic depth should be prioritized while maintaining the observed high safety profile.

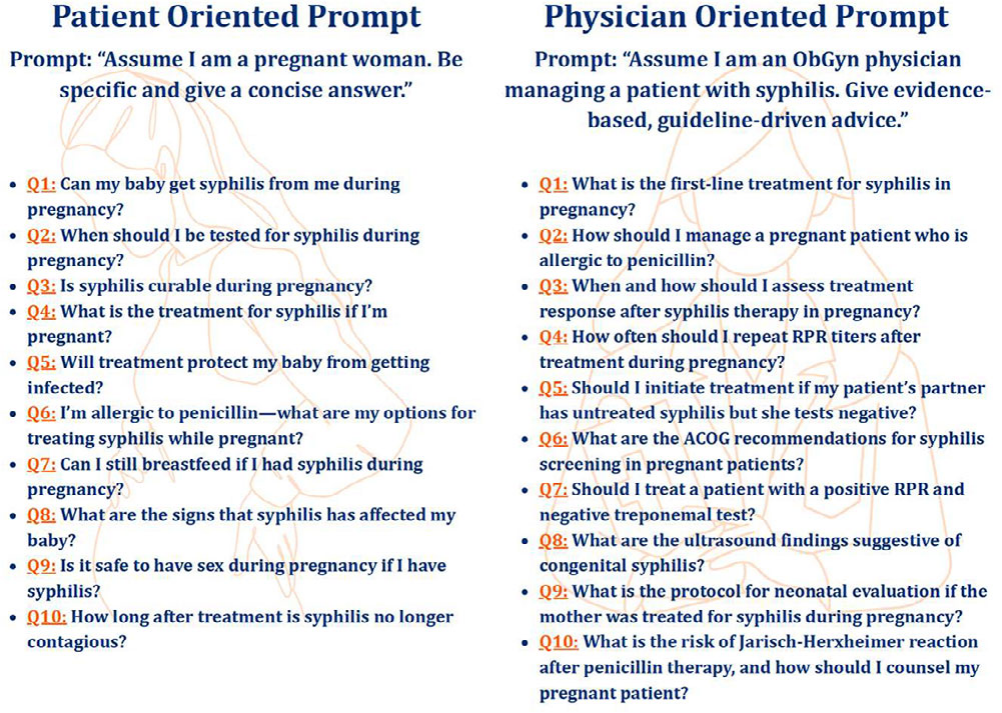

**3:30 PM – 5:00 PM**

## 1128 | **Continuous Intrapartum Fetal Lactate Monitoring: A First‐in‐Fetus Study Using a Novel Multi‐Modal Biosensor**

Jonathan M. Morris^1^; Sean Seeho^1^; Sharon McCracken^1^; Daniel L. Rolnik^2^; Scott White^3^; Deborah Fox^4^; Madeline Hawke^4^; Joao Santos^5^; Chuong Phan^6^; Anup Pollukonda^5^; Maureen Ross^5^; Ranjusha Rajagopalan^5^; Julian Shapley^5^; Arjun Kaushik^5^; Lee J. Hubble^5^; Jane Pillow^7^

^1^University of Sydney, University of Sydney, New South Wales; ^2^Monash Health, Melbourne, Victoria; ^3^King Edward Memorial Hospital, University of Western Australia, Western Australia; ^4^University of Technology, University of Technology, Sydney, New South Wales; ^5^VitalTrace, VitalTrace, Perth, Western Australia; ^6^VitalTrace, VitalTrace, Western Australia; ^7^University of Western Australia, University of Western Australia, Western Australia

**Objective**: To evaluate a novel lactate biosensor incorporated into the form factor of a fetal scalp electrode applied during human labour.

**Study Design**: Women (*n* = 10) who had experienced an uncomplicated pregnancy and in whom vaginal birth was anticipated were consented to participate in the study. The biosensor was applied to the fetal scalp when in active labor, membranes ruptured and the cervix at least 3 cm dilated. Fetal heart rate and lactate outputs from the device were blinded to caregivers and patients. The sensor was removed immediately prior to birth. Umbilical cord arterial samples were collected at the time of birth for lactate analysis. A factory calibration method was developed using fetal lamb data to predict umbilical cord lactate.

**Results**: Of the 10 sensors applied contemporaneous acquisition of fetal heart rate and lactate current was obtained from 7 fetuses. Figure 1 demonstrates representative traces following processing and demonstrate a rise in lactate throughout labor.

The mean time of sensor application was 276 minutes (range 28 minutes – 465 minutes), with intrapartum lactate measurements obtained from as early as 15 minutes. Using data from extensive preclinical testing in the fetal lamb, predicted umbilical cord lactate correlated with that obtained from blood gas analysis. Table 1 demonstrates the concordance between the predicted lactate and the umbilical cord lactate. Overall, the Mean Average Error (MAE) was 0.68 mmol/L.  User acceptability of the device was high and there were no significant adverse events noted.

**Conclusion**: This study establishes the feasibility of acquisition of continuous fetal lactate measurement and fetal heart rate with a single device. The approach has the potential to advance intrapartum care and improve neonatal outcomes. Larger clinical studies are planned.

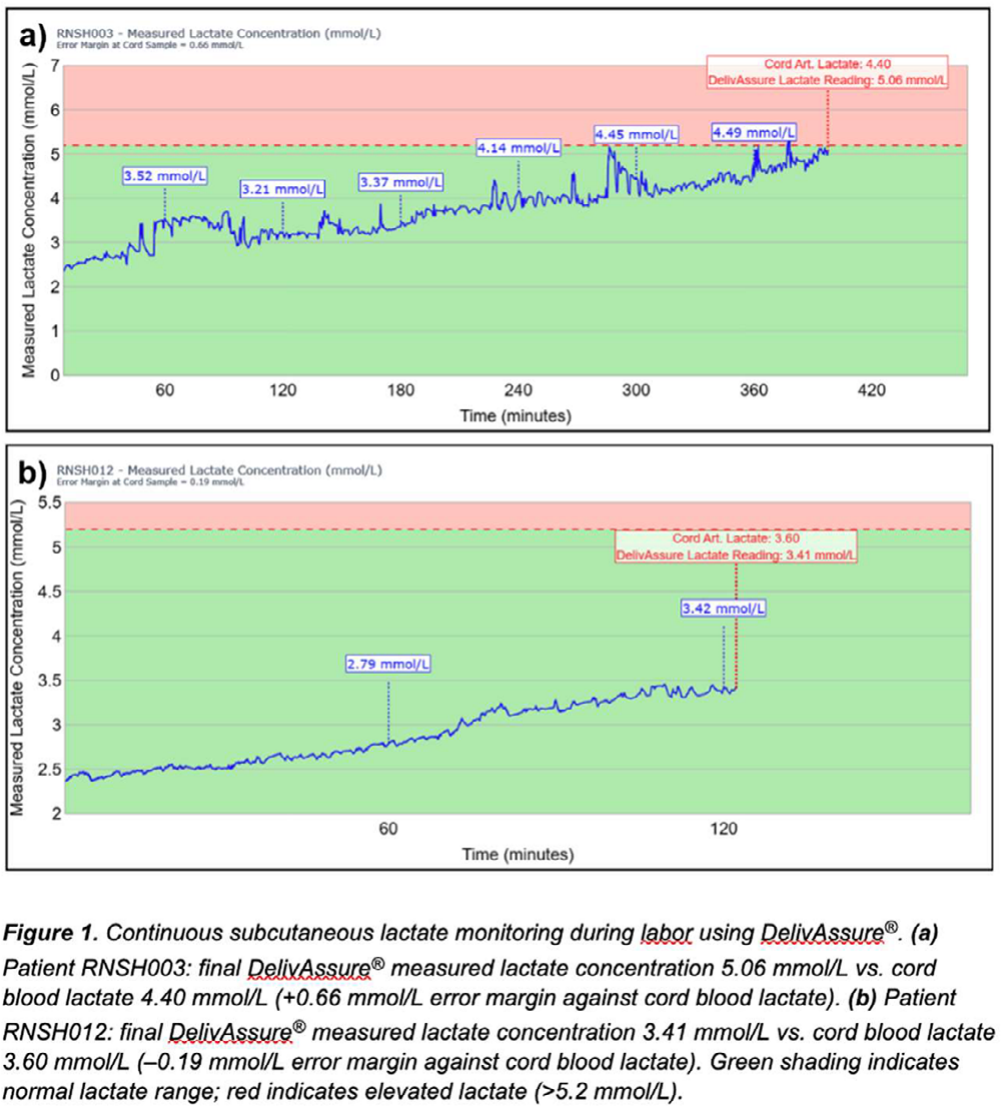

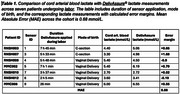

**3:30 PM – 5:00 PM**

## 1129 | **Lightening the Load: Does Concurrent Partner Screening Decrease the Clinical Genetic Counseling Burden?**

Joselle O'Brien^1^; Eliana Wachstock^2^; Emily Schlussel Markovic^1^; Itamar Futterman^3^; Robyn Weiss^1^; Jeremy Weedon^4^; Shoshana Haberman^1^; Rodney A. McLaren, Jr^1^; Tirtza Spiegel Strauss^1^

^1^Maimonides Medical Center, Brooklyn, NY; ^2^Yeshiva University, New York, NY; ^3^Maimonides Medical Center, Maimonides Health, NY; ^4^SUNY Downstate, Brooklyn, NY

**Objective**: Expanded carrier screening (ECS) has increased in use; however, rates of genetic counseling and partner screening remain low. We implemented multiple interventions to increase partner screening and thereby decrease need for genetic counseling. We compared how often genetic counseling was indicated pre‐ and post‐intervention.

**Study Design**: This was an IRB approved retrospective cohort study at an academic hospital clinic. From 1/1/2024‐2/28/24, interventions were implemented, specifically, ECS was standardized to 113 genes recommended by American College of Medical Genetics, providers were educated, and free concurrent partner screening was offered via blood draw or prepaid saliva kit. Pre‐intervention, patients heterozygous for pathogenic variants were referred for genetic counseling, while post‐intervention, couples with reproductive risk (carrier couples and X linked variants) and patients with variants whose partners were not screened were referred, and clinic providers reviewed other results. The primary outcome was the percentage of indicated post‐test genetic counseling pre‐ and post‐intervention. Secondary outcomes included uptake of indicated post‐test genetic counseling, rate of concurrent partner screening post intervention and rate of partner screening in patients with variants.

**Results**: There was a total of 444 patients; 156 pre‐intervention and 288 post‐intervention (demographics in Table 1, results in Table 2). The need for genetic counseling significantly decreased post intervention (65.4% vs 18.1%, *p* < 0.01). Uptake of post‐test genetic counseling was low but did not vary between groups. The rate of concurrent partner screening was 58% (166/288). In patients with variants, the rate of partner screening was 71% (100/141).

**Conclusion**: Standardizing ECS, educating providers, and offering free concurrent partner screening increases partner screening rates and decreases need for post‐test genetic counseling.  This may reduce the clinical burden of genetic counseling in areas with shortage. Larger studies are needed to confirm our findings.

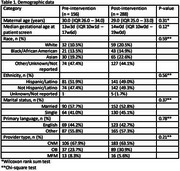

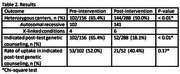

**3:30 PM – 5:00 PM**

## 1130 | **Evaluating Content Quality on Laser Ablation for Twin‐to‐Twin Transfusion Syndrome: YouTube, TikTok, and Instagram Reels**

Kai Holder^1^; Prottusha Sarkar^2^; Faith Kehinde^3^; Tiffany Deihl^4^

^1^UPMC Magee‐Womens Hospital, Pittsburgh, PA; ^2^University of New Mexico, Albuquerque, NM; ^3^University of Pittsburgh Medical Center, Pittsburgh, PA; ^4^UPMC Magee Womens Hospital, Pittsburgh, PA

**Objective**: Social media platforms are increasingly accessed by high‐risk obstetric patients seeking information on complex prenatal diagnoses. This study aimed to evaluate the quality of videos on YouTube, TikTok, and Instagram Reels as educational resources on fetal laser ablation for twin‐to‐twin transfusion syndrome (TTTS), a rare but life‐threatening complication of monochorionic twin pregnancies.

**Study Design**: A systematic search of YouTube, TikTok, and Instagram Reels was conducted using a public account and keywords related to fetal laser ablation for TTTS. For each platform, the first 60 videos per keyword were screened, and unique videos were analyzed. Each video was evaluated using an expert‐derived content score and the DISCERN instrument, a validated tool for evaluating consumer health information. Videos scoring ≥5 (content) and ≥39 (DISCERN) were classified as high quality. Descriptive statistics and intergroup comparisons were performed using Bonferroni‐adjusted Mann‐Whitney U tests.

**Results**: A total of 324 unique videos were analyzed: YouTube (*n* = 176), TikTok (*n* = 91), and Instagram Reels (*n* = 57). Mean DISCERN scores were similar between platforms and did not differ significantly: TikTok (43.20 ± 13.70), Instagram Reels (41.05 ± 12.70), and YouTube (39.66 ± 12.40); all *p* > 0.10. Mean content scores varied significantly by platform with YouTube demonstrating the highest mean content score (2.66 ± 2.95), followed by TikTok (1.26 ± 1.85) and Instagram Reels (0.62 ± 1.19); all *p*  < 0.001. Only 19 videos (5.86%) met high‐quality criteria: YouTube (*n* = 17, 9.66%), TikTok (*n* = 2, 2.20%), and Instagram Reels (*n* = 0, 0.00%).

**Conclusion**: YouTube had the highest content scores and accounted for the majority of high‐quality videos; however, high‐quality educational content on TTTS was limited across all platforms. With pregnant patients increasingly turning to social media for information on complex fetal conditions—particularly short‐form platforms like TikTok and Instagram Reels—it is critical to improve content completeness and accuracy to support informed decision‐making and timely referral for fetal therapy.

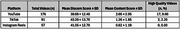

**3:30 PM – 5:00 PM**

## 1131 | **Risk of Infection Using Cervical Ripening Balloon for Premature Rupture of Membranes: Systematic Review Meta‐Analysis**

Kamran Hessami^1^; Sarah Tounsi^1^; Mark A. Turrentine^2^; Manisha Gandhi^1^

^1^Baylor College of Medicine, Houston, TX; ^2^Department of Obstetrics and Gynecology Baylor College of Medicine, Houston, TX

**Objective**: To estimate the rate of intra‐amniotic infection (IAI) in the setting of premature rupture of membranes (PROM) with usage of cervical ripening balloon (CRB) compared with oxytocin and/or prostaglandin for cervical ripening for induction of labor.

**Study Design**: A systematic review and meta‐analysis was conducted using terms related to CRB induction from database inception to 7/1/2025 and registered in Prospero (CRD420251085355). Randomized controlled trials (RCTs) and cohort studies comparing CRB with pharmacologic ripening (either prostaglandin or oxytocin) were included. The primary outcome was clinical diagnosis of IAI. The secondary outcomes were rates of cesarean delivery, maternal fever, and postpartum endometritis. The random‐effects model was used to pool the odds ratios (ORs) and the corresponding 95% confidence intervals (CIs).

**Results**: Fourteen studies (7 RCTs and 7 cohort) involving 2137 participants were included. The rate of IAI was similar between individuals undergoing ripening with CRB as compared to prostaglandin, 6.3% vs 4.4%, respectively (OR 1.23 [95% CI: 0.49, 3.09], I^2^: 61.8%) and CRB compared to oxytocin, 10.8% vs 7.3%, respectively (OR 1.69 [95% CI: 0.83, 3.43], I^2^ = 26.4%). Separate subgroup analysis based on study design (RCT vs cohort) was performed which showed similar results (Figure 1). The secondary outcomes were similar between the CRB and pharmacologic ripening groups (Figure 2).

**Conclusion**: Rates of IAI were similar in patients with PROM that underwent cervical ripening with CRB compared to pharmacologic methods. Due to the lack of adequate power to rule out a type II error in the current pooled analysis, future studies are needed before one method can be recommended over the other.

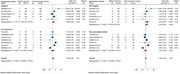

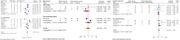

**3:30 PM – 5:00 PM**

## 1132 | **Impact of BMI Classifications and Obesity Definitions on Perinatal Outcomes Among Asian American Pregnant Patients**

Kandace Fung^1^; Apekshya Nepal^2^; Kennedi Randolph^2^; Song Hyun Kim^2^; Nazow B. Tarakai^2^; Jiayue Chen^3^; Lorna Kwan^4^; Christina S. Han^5^

^1^Charles R. Drew University of Medicine and Science; David Geffen School of Medicine at University of California, Los Angeles (UCLA), Los Angeles, CA; ^2^David Geffen School of Medicine at University of California, Los Angeles (UCLA), Los Angeles, CA; ^3^Department of Urology, David Geffen School of Medicine at University of California, Los Angeles (UCLA), Los Angeles, CA; ^4^Department of Urology, David Geffen School of Medicine at University of California, Los Angeles (UCLA), Los Angeles, CA; ^5^Department of Obstetrics and Gynecology, David Geffen School of Medicine at University of California, Los Angeles (UCLA), Los Angeles, CA

**Objective**: Asian American (AsA) populations are disproportionately affected by T2DM and increased cardiovascular risk factors. The WHO has set an international standard (IS‐BMI) obesity cut‐off at BMI ≥30 kg/m^2^ but recently recommended a different BMI cut‐off of ≥27.5 kg/m^2^ for Asian populations. This study aims to evaluate how the Asian standard BMI (AS‐BMI) threshold affects pregnancy and neonatal outcomes in AsA patients. Our hypothesis was that risk factors and pregnancy outcomes will differ in AsA pregnant individuals when using the two BMI thresholds for obesity.

**Study Design**: We conducted a retrospective case‐control study of patients at two urban academic medical centers between 01/2022 to 12/2023. Inclusion criteria were age 16‐49 years and singleton gestation.  Exclusion criteria included aneuploidy, pregestational diabetes, multiple gestations, bariatric surgery, cystic fibrosis, or chronic systemic steroid use. Comparisons were made between AsA groups with varying BMI cut‐offs, using non‐obese AsA and non‐AsA as references. Kruskal‐Wallis and Chi‐squared were used where appropriate.

**Results**: 573 deliveries were analyzed, AsA(17.3%) and non‐AsA(82.7%). The AsA group has higher mean age(*p* = 0.0032), lower early pregnancy using IS‐BMI (*p* < 0.001), and earlier GA delivery (*p* = 0.0006). Table 1 shows higher BMI cohorts using both standards having increased prevalence of macrosomia. Table 3 shows when comparing non‐AsA, both AsA cohorts have delivery at earlier GA, and AsA with AS‐BMI are also less likely to meet IOM standards(*p* = 0.0436). No significant differences in hypertensive disorders of pregnancy, degree of lacerations, or birth weight.

**Conclusion**: Use of the AS‐BMI threshold identified a broader at‐risk population with higher prevalence of individuals not being less likely to meet IOM weight gain standards even with lower initial BMI. Regardless of BMI standard, both AsA cohorts have earlier GA deliveries compared to the non‐AsA cohort with higher rates of c‐section. These findings support further investigation into risk stratifying and clinical guidance for Asian American populations.

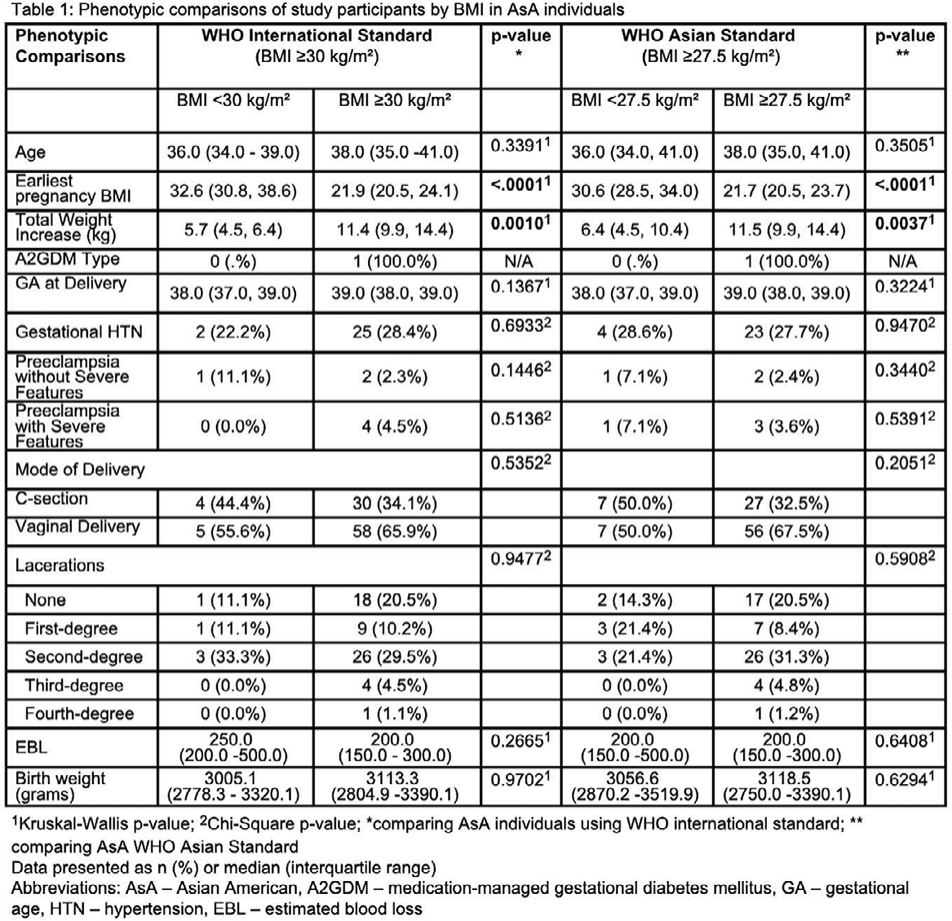

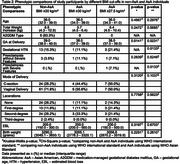

**3:30 PM – 5:00 PM**

## 1133 | **Severe Maternal Morbidity and Breastfeeding Among US Nulliparous Individuals**

Tracy Caroline Bank^1^; Jiqiang C. Wu^2^; Janet Catov^3^; Lynn M. Yee^4^; David M. Haas^5^; Rebecca B. McNeil^6^; Jessica Pippen^7^; Hyagriv Simhan^8^; Uma M. Reddy^9^; Robert M. Silver^10^; Lisa Levine^11^; George R. Saade^12^; Judith H. Chung^13^; Courtney C. Lynch^2^; William A. Grobman^14^; Kartik K. Venkatesh^15^

^1^The Ohio State University Wexner Medical Center, COLUMBUS, OH; ^2^Ohio State University Wexner Medical Center, COLUMBUS, OH; ^3^University of Pittsburgh, Pittsburgh, PA; ^4^Northwestern University Feinberg School of Medicine, Chicago, IL; ^5^Indiana University School of Medicine, Indianapolis, IN; ^6^RTI International, Triangle Park, NC; ^7^MetroHealth Medical Center, Cleveland, OH; ^8^University of Pittsburgh Medical Center, Pittsburgh, PA; ^9^Columbia University Irving Medical Center, New York, NY; ^10^Department of Obstetrics and Gynecology, University of Utah Health, Salt Lake City, UT; ^11^Hospital of the University of Pennsylvania, Philadelphia, PA; ^12^Department of Obstetrics and Gynecology, Eastern Virginia Medical School at Old Dominion University, Norfolk, VA; ^13^University of California Irvine Medical Center, Irvine, CA; ^14^Warren Alpert Medical School of Brown University, Providence, RI; ^15^The Ohio State University Wexner Medical Center, The Ohio State University Wexner Medical Center, OH

**Objective**: While certain delivery complications, such as postpartum hemorrhage, have been associated with less breastfeeding, little is known about breastfeeding following deliveries complicated by severe maternal morbidity (SMM). Therefore, we examined whether SMM at delivery hospitalization was associated with the likelihood and duration of breastfeeding among US nulliparous individuals.

**Study Design**: This was a secondary analysis from the prospective cohort Nulliparous Pregnancy Outcomes Study: Monitoring Mothers‐To‐Be Heart Health Study. The primary exposure was any SMM, based on the Centers for Disease Control and Prevention definition, and secondarily, non‐transfusion SMM. The primary outcomes were self‐reported breastfeeding initiation (yes/no) and, among those who initiated breastfeeding, its duration (<6 weeks as reference, 6 weeks to 6 months, and >6 months) assessed 6 months after delivery. Secondarily, exclusive breastfeeding and its duration were assessed. Multivariable logistic regression and ordinal logistic regression were used and adjusted for age, insurance, and pre‐pregnancy body mass index.

**Results**: Among 6762 nulliparous individuals, those who experienced SMM had similar odds of breastfeeding initiation as those without SMM (83.8% vs. 88.3%; aOR: 0.73; 95% CI: 0.46, 1.15), but among those who breastfed, SMM was associated with a shorter duration of breastfeeding (aOR: 0.50; 95% CI: 0.36, 0.68). SMM was associated with significantly lower odds of exclusive breastfeeding (76.4% vs. 85.6%; aOR: 0.60; 95% CI: 0.37, 0.98), albeit not the duration of exclusive breastfeeding (aOR: 1.06; 95% CI: 0.70, 1.64). Once transfusion was removed as a component of SMM, point estimates revealed even stronger associations with reduction in the duration of breastfeeding (TABLE).

**Conclusion**: SMM at delivery hospitalization was associated with significantly lower odds of initiating and sustaining breastfeeding. Future research is needed to understand how to better support individuals in optimizing lactation following deliveries complicated by SMM.

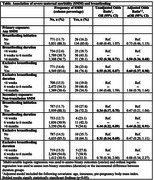

**3:30 PM – 5:00 PM**

## 1134 | **Dobbs and the Double Burden: Pregnancy Amid Intimate Partner Violence in an Abortion Restrictive State**

Katelin J. Boak^1^; Lori M. Stevenson^2^; Nandini Raghuraman^3^; Antonina I. Frolova^3^; Megan L. Lawlor^3^; Amanda C. Zofkie^3^; Sydney M. Thayer^4^; Jeannie C. Kelly^4^

^1^Washington University School of Medicine, Saint Louis, MO; ^2^Barnes‐Jewish Hospital, St. Louis, MO; ^3^Washington University School of Medicine, St. Louis, MO; ^4^Washington University School of Medicine, Washington University School of Medicine, MO

**Objective**: To assess whether maternal and neonatal outcomes changed following the Dobbs v. Jackson Women's Health Organization decision among obstetric patients referred for intimate partner violence (IPV) consultation.

**Study Design**: We conducted a retrospective cohort study of patients on an obstetric inpatient unit who were referred to the “AWARE” program, an intimate partner violence assistance program at a tertiary academic center in an abortion‐restrictive state. Patients were grouped as Pre‐Dobbs (June 2020–June 2022) or Post‐Dobbs (Nov. 2022–Sept. 2024). Demographics, injury characteristics, and outcomes were abstracted from the electronic medical record. Primary outcomes included preterm birth and a composite of perinatal complications. Secondary outcomes included NICU admission and child discharge to biological parents. Multivariable logistic regression models adjusted for relevant covariates were selected via backwards elimination.

**Results**: Of 234 AWARE consults, 130 occurred pre‐ and 104 post‐Dobbs. A greater proportion of deliveries received AWARE consults pre‐Dobbs (2% vs 1.5%, *p* = 0.009). Most patients were Black (72%) and publicly insured (79%), with high rates of substance use and mental health conditions. Injury patterns were similar across eras. Head trauma was most common (89% vs 84%). Multiple injury sites were frequent (23%) and blunt trauma was the most common mechanism. While no statistically significant differences in outcomes were observed, adverse events remained common: preterm birth (29% vs 21%), composite complications (56% vs 50%), and NICU admission (30% vs 27%). Notably, most infants were discharged to biological parents (76% vs 86%).

**Conclusion**: Although we hypothesized that abortion restriction may exacerbate the effects of IPV experienced by pregnant individuals, we did not observe significant differences in perinatal outcomes between eras. However, persistently high rates of adverse maternal and neonatal outcomes in both cohorts reflects the vulnerability of this population and the care gaps that have existed in our state even prior to the Dobbs decision.

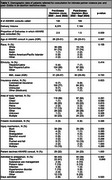

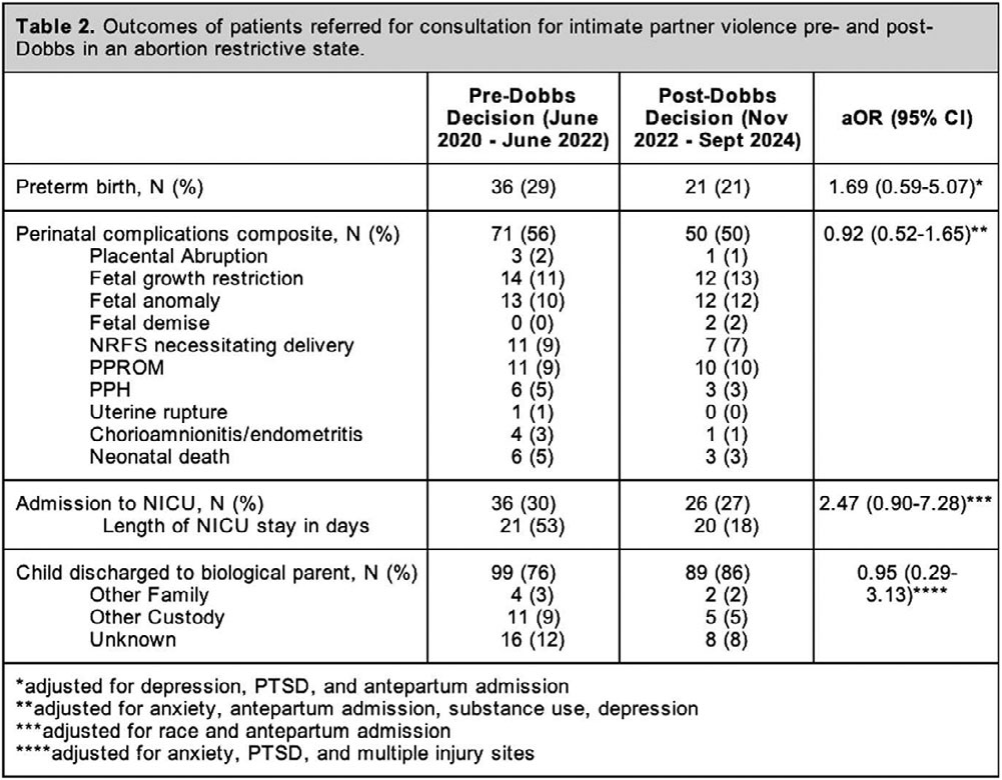

**3:30 PM – 5:00 PM**

## 1135 | **Identifying Prolonged Second Stage Labor Using Machine Learning Techniques**

Stephanie Guang^1^; Katherine L. Grantz^2^; Mathew Bruckner^3^; Advait Nene^4^; Jessica Gleason^5^; Neil Perkins^2^; Rajeshwari L. Sundaram^2^

^1^Columbia University, New York, NY; ^2^Eunice Kennedy Shriver National Institute of Child Health and Human Development, National Institutes of Health, Bethesda, MD; ^3^The Prospective Group, Inc., Fairfax, VA; ^4^Carnegie Mellon University, Pittsburgh, PA; ^5^NICHD, NIH, Bethesda, MD

**Objective**: Clinical practice of safe duration for the second stage of labor has been evolving. Assessing risk of prolonged second stage is dependent on many dynamic antepartum and intrapartum factors yet prior work used classical regression with a limited number of well‐defined variables. We used machine learning models to analyze high‐dimensional electronic health record data to predict prolonged second stage of labor.

**Study Design**: In the Consortium on Safe Labor, prolonged second stage of labor duration was defined as beyond two hours for nulliparas and one hour for multiparas with an additional hour for an epidural. Sixty‐nine risk factors extracted from prenatal and delivery electronic medical records combined with ICD–9 codes were considered. We used logistic regression and random forest to predict prolonged labor, over all deliveries, and stratified by parity. We also studied the predictive ability of models with and without Bishop's score. Partial dependence plots were used to explore the direction of association of the risk‐factors on prolonged second stage labor in this high‐dimensional setting.

**Results**: Of the 103,415 parturients assessed, 3519 (3.5%) had prolonged second stage of labor Mean second stage duration was 0.85 hours (SD 1.02). The random forest model had the best performance with area under the curve of 0.80 (95% CI 0.78‐0.81) for all deliveries, 0.81 in multiparous and 0.72 in nulliparas.(Table 1) Overall, the most important features selected in best predictive models included parity, station at full dilation, cervical examination data at admission and maternal age.(Figure) Cervical dilation on admission was the predominant prediction factor for multiparas; in contrast, there were multiple factors for nulliparas, with maternal age, admission contractions, epidural and cervical effacement leading.

**Conclusion**: These findings demonstrate utility of machine learning in predicting prolonged second stage of labor using data available to the obstetric provider. Machine learning plays a promising role in creating individualized predictions to support clinical risk assessment during labor.

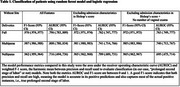

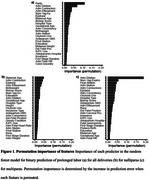

**3:30 PM – 5:00 PM**

## 1136 | **Cesarean SSI and BMI: Relationship Between the Two in the Era of Modern Antibiotic Prevention**

Katherine H. Zhu^1^; Lori M. Stevenson^2^; Heather Campbell^1^; Roxane Rampersad^3^; Amanda C. Zofkie^3^; Megan L. Lawlor^3^; Antonina I. Frolova^3^; Nandini Raghuraman^3^; Jeannie C. Kelly^4^

^1^Washington University School of Medicine, St Louis, MO; ^2^Barnes‐Jewish Hospital, St. Louis, MO; ^3^Washington University School of Medicine, St. Louis, MO; ^4^Washington University School of Medicine, Washington University School of Medicine, MO

**Objective**: Updated antibiotic regimens and prevention bundles have reduced surgical site infection (SSI) rates after cesarean delivery (CD), but SSI remains a concern. Elevated BMI has traditionally been a major risk factor, yet its impact amid evolving clinical protocols is uncertain. We sought to evaluate the association between BMI and SSI following CD in the context of modern perioperative practices.

**Study Design**: We performed a case control of all CDs at an urban tertiary care center from January 2024‐July 2025 where antibiotic dosage by BMI, azithromycin for labored CD, and redosing for extended operative time and high blood loss are standard practice. Cases were defined as patients with SSI based on National Healthcare Safety Network criteria, without exclusions for BMI >60; controls were CDs without SSI during the same period. The primary exposure was BMI. Multivariable logistic regression estimated adjusted odds ratios (aORs) for SSI. Covariates included age, quantitative blood loss, repeat CD, and other clinical factors. Stratified analyses compared scheduled versus labored CDs (after labor onset or rupture of membranes).

**Results**: We identified 32 CD with SSI and 1761 without. In unadjusted analysis, BMI 35–45 kg/m^2^ was significantly associated with higher odds of SSI (OR 2.70, 95% CI 1.07–8.22; Figure, Table). However, after multivariable adjustment with backwards selection, BMI was no longer significantly associated with SSI. Only maternal age remained in the final model, with each additional year linked to a 9.2% reduction in odds of SSI (aOR 0.91, 95% CI 0.83–0.99). In stratified analyses, BMI was not significantly associated with SSI in either scheduled or labored CD. However, younger maternal age remained significantly associated with increased SSI risk in the labored group.

**Conclusion**: In the era of updated antibiotic regimens and bundled care, BMI did not independently predict SSI risk following CD after adjusting for other factors. Younger maternal age emerged as a key risk factor, possibly reflecting social determinants affecting SSI risk.

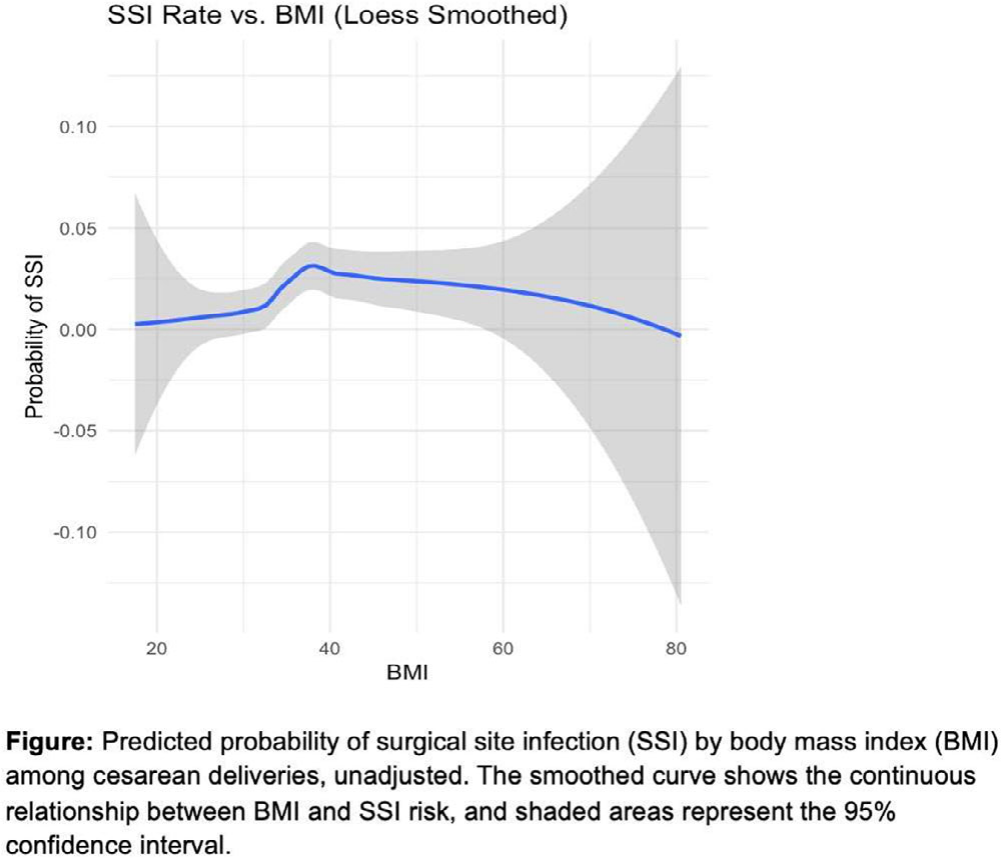

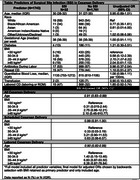

**3:30 PM – 5:00 PM**

## 1137 | **Evaluating Outcomes of Expectantly Managed CSEP in a Histologically Confirmed PAS Cohort**

Kathy Mostajeran^1^; Elizabeth Mangone^2^; Lelan McCann^1^; Pamela Garcia‐Filion^1^; Christopher Huls^1^

^1^University of Arizona College of Medicine, Phoenix, AZ; ^2^University of Arizona College of Medicine Phoenix, Phoenix, AZ

**Objective**: To compare maternal and neonatal outcomes in patients with and without 1st trimester evidence of cesarean scar ectopic pregnancy (CSEP) in a cohort with histologically confirmed placenta accreta spectrum (PAS).

**Study Design**: In this single‐center retrospective study of patients with confirmed PAS (2012–2024), patients were included if: 1) 1st trimester ultrasound images were available, or 2) an outside diagnosis of CSEP existed with referral for continuation of pregnancy. Two blinded MFM subspecialists independently reviewed all available 1st trimester images using validated criteria for categorization. Cases included those with a CSEP diagnosis either by retrospective image review or established prior to referral. Controls lacked evidence of CSEP on image review.

**Results**: Among 273 histologically confirmed PAS cases, 47 (17.2%) met inclusion criteria. CSEP was identified in 31 (66.0%): 6 (19.4%) by outside diagnosis and 25 (80.6%) by expert imaging review. Sixteen (34.0%) patients did not have a CSEP by review of available ultrasound images. Baseline demographics were similar. CSEP patients had more prior cesareans and higher gravidity and parity (*p* < 0.01).  There were no differences in gestational age at delivery, intra‐operative blood loss, transfusions, or peri‐operative morbidity between groups. Percreta was more prevalent in the CSEP group (41.9% vs 12.5%; *p* = 0.04). Live birth rates were similar between groups (93.5% vs. 93.8%; *p* = 0.98) without any significant differences in Apgar scores.

**Conclusion**: Patients with a diagnosis of CSEP in the 1st trimester did not experience greater maternal or neonatal morbidity compared to those with no evidence of CSEP in a cohort of patients with histologically confirmed PAS. These findings challenge current guidelines that strongly recommend early termination of CSEP. The study provides support for the option of expectant management in appropriately selected and counseled patients receiving care with experts in PAS. The trend toward more invasive pathology in the CSEP group underscores the importance of early identification and coordinated planning.

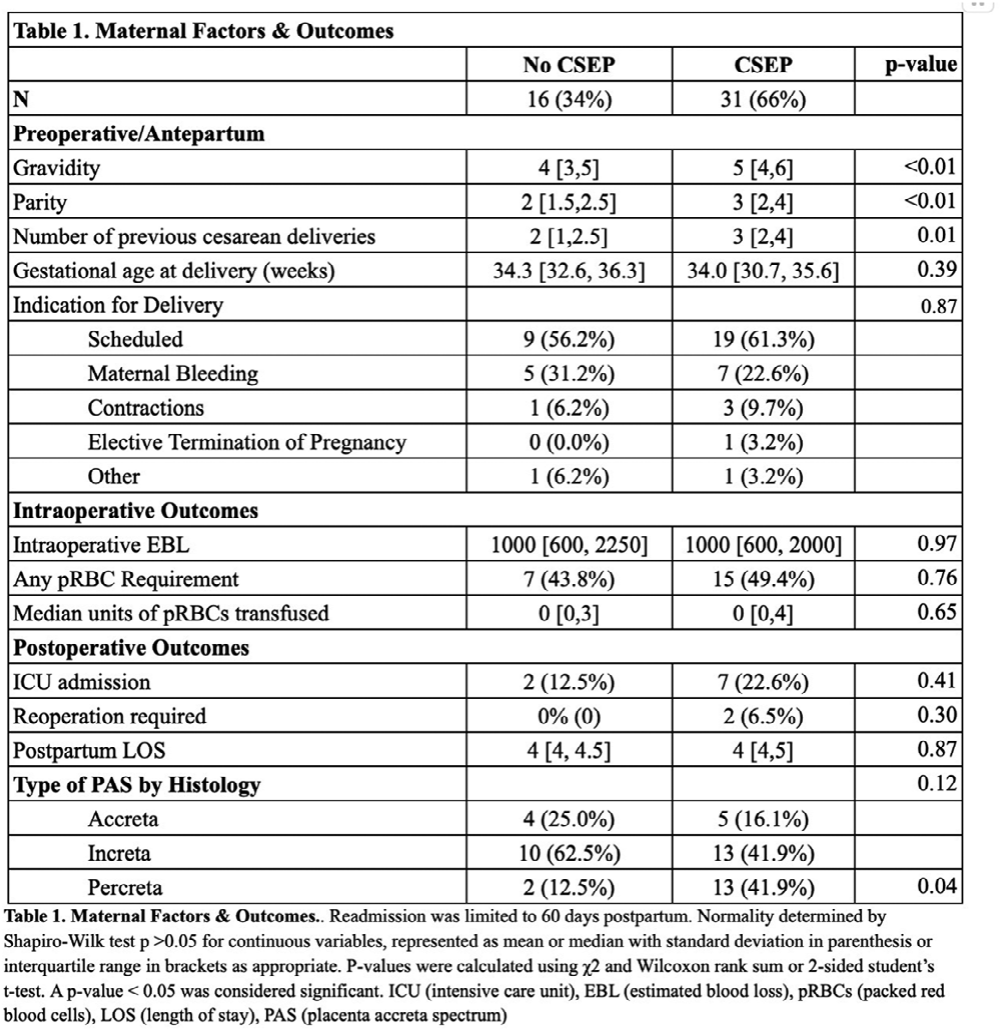

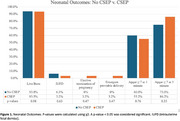

**3:30 PM – 5:00 PM**

## 1138 | **Is the Incidence of Placental Insufficiency Increased After Prenatal Surgery for Spina Bifida?**

Katie Howard^1^; Bree A. Goodman^1^; Jesse Vrecenak^2^; Jennifer M. Strahle^1^; Jagruti Anadkat^2^; Nandini Raghuraman^1^; Katherine H. Bligard^2^

^1^Washington University School of Medicine, St. Louis, MO; ^2^Washington University School of Medicine, Saint Louis, MO

**Objective**: Fetal open neural tube defects (ONTDs) affect 0.1% of pregnancies. Prenatal repair improves fetal outcomes but comes with a higher risk of preterm delivery (PTD). It is unclear if prenatal repair, or repair technique (fetoscopic or open via hysterotomy), lead to placental pathology that may increase the risk of PTD or obstetric complication. This study aims to compare pathologic markers of placental insufficiency in fetuses with repaired vs unrepaired ONTDs.

**Study Design**: This is a retrospective cohort study of pregnancies with fetal ONTDs referred to a single fetal center between 2015 and 2024. The primary outcomes were maternal and fetal malperfusion scores, which are composite measures of placental parenchymal lesions associated with placental injury related to altered uterine and intervillous blood flow. Secondary outcomes included placental weight, delivery outcomes, birthweight, umbilical artery pH, Apgar score at 1 and 5 minutes, and NICU length of stay. Outcomes were compared between cases undergoing prenatal vs postnatal repair, as well as between repair type.

**Results**: Of the 88 fetuses included, 42 underwent prenatal repair, while 46 underwent postnatal repair. Of the 42 prenatally repaired cases, 32 were repaired via hysterotomy, while 10 were repaired fetoscopically. Maternal characteristics, including age, parity, and chronic medical conditions did not differ across groups. Maternal and fetal placental malperfusion scores did not differ by prenatal vs postnatal repair cases (Table 1) or by surgical approach among prenatally repaired cases (Table 2). Gestational age at delivery was earlier in the prenatal repair group (36.1 vs 39.0 weeks, *p* < 0.01) but did not differ between type of prenatal repair (fetoscopic 35.7 vs hysterotomy 36.3 weeks, *p* = 0.70). There were no differences in placental weight percentile, birthweight percentile, or assessed neonatal outcomes amongst comparison groups.

**Conclusion**: Prenatal repair of ONTDs is not associated with placental malperfusion. This suggests that the higher rate of PTD after prenatal repair is not driven by placental insufficiency.

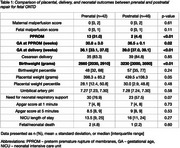

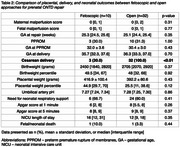

**3:30 PM – 5:00 PM**

## 1139 | **Postpartum Follow‐Up and Pregnancy Outcomes in Individuals With Diabetes: A Retrospective Cohort Study**

Rachel Nordlicht^1^; Jessica Atrio^2^; Kavita Vani^3^

^1^Albert Einstein College of Medicine, The Bronx, NY; ^2^Alice L. Walton School of Medicine, Bentonville, AR; ^3^Albert Einstein College of Medicine/Montefiore Medical Center, Bronx, NY

**Objective**: To evaluate the association of postpartum visit attendance among persons with diabetes at or before delivery in a large obstetric cohort and to explore other associated demographic factors.

**Study Design**: We conducted a retrospective cohort study of 13,874 patients who received prenatal care and delivered at Montefiore Medical Center from June 2018 ‐ May 2022. Patients were categorized by diabetes status: gestational diabetes mellitus (GDM), pre‐gestational diabetes (PGDM) and no diabetes, using ICD‐9/10 codes at delivery. The primary outcome was postpartum visit (PPV) attendance within 12 weeks. Secondary outcomes included emergency room (ER) visitation and hospital readmission within 12 weeks. Bivariate analysis compared patient characteristics and outcomes. Logistic regression adjusted for demographics, prenatal care engagement, medical co‐morbidities, delivery outcomes, and NICU admission.

**Results**: 1776 (12.9%) patients had GDM and 374 (2.7%) had PGDM. Compared to those without diabetes, patients with GDM and PGDM were older (mean age 35.3 + 5.7 vs. 36.6 + 6.3 vs. 33.3 + 6.0 years), had higher rates of preterm delivery (10.6% vs. 24.9% vs. 8.8%), cesarean delivery (42.6% vs. 62.0% vs. 34.2%), hypertensive disorders (27.8% vs. 54.6% vs. 24.5%), and NICU admissions (12.6% vs. 29.3% vs. 9.4%), all *p* < 0.01. PPV attendance was higher among patients with GDM and PGDM (59.1% vs. 70.6% vs. 53.1%, *p* < 0.01). However, after adjustment, GDM was associated with lower odds of PPV attendance (aOR 0.82, 95% CI 0.73–0.92, *p* = 0.001), and PGDM had no significant association (aOR 0.92, 95% CI 0.72–1.19, *p* = 0.54). GDM was associated with lower odds of readmission (aOR 0.74, 95% CI 0.56‐0.97, *p* = 0.03) and PGDM was associated with increased ER visits (aOR 1.33, 95% CI 1.02‐1.73, *p* = 0.03).

**Conclusion**: Despite their elevated clinical risk, patients with GDM were less likely to attend a PPV, while those with PGDM had higher rates of emergency care use. These findings highlight the need for targeted strategies to improve postpartum care engagement among patients with diabetes.

**3:30 PM – 5:00 PM**

## 1140 | **Evaluating the Relationship Between Fetal Aneuploidy, Severe Maternal Morbidity and Hypertensive Diseases in Pregnancy**

Keiko Fox^1^; Luke Enthoven^1^; Hannah E. Vincent^2^; Maeve Hopkins^3^; Manesha Putra^2^

^1^University of Colorado School of Medicine, Aurora, CO; ^2^University of Colorado, Aurora, CO; ^3^Cleveland Clinic Foundation, Cleveland, OH

**Objective**: To assess whether fetal aneuploidy (FA) is independently associated with increased frequency of severe maternal morbidity (SMM) and hypertensive diseases in pregnancy (HDP) outcomes relative to delivery of non‐aneuploid pregnancies.

**Study Design**: This cross‐sectional study utilized the most recent United States’ National Inpatient Sample dataset, which was queried for delivery admissions between 2016‐2022. Women with ICD‐10 codes for pregnancy complicated by FA (O35.1*) diagnosed on delivery admission were compared with those without. SMM was defined as presence of at least one Centers for Disease Control and Prevention severe maternal morbidity indicator. Comorbidity index (CI) was established based on prior report. Bivariate analyses were used to evaluate the association of FA and SMM and HDP. Logistic regression was performed to model the association between aneuploidy and severe maternal morbidity, controlling for the presence of CI. A second logistic regression model was made to assess the association between aneuploidy and HDP, while controlling for the presence of CI without HDP indicators.

**Results**: Of 5,038,140 deliveries, 6144 involved FA**. **The rate of SMM was similar in pregnancy with and without FA (2.2 vs. 1.9%, *p* 0.07). HDP was more common in pregnancy with FA compared to pregnancy without (14.8 vs. 12.9%, *p* < 0.001). In the logistic regression model, FA is not associated with SMM when controlled for CI (OR 1.03, 95% CI 0.96  –  1.11, *p* 0.33). When controlled for CI without HDP indicators, fetal aneuploidy is associated with higher risk of HDP (OR 1.66, 95% CI 1.53  – 1.79, *p* < 0.001).

**Conclusion**: While there is no association of FA with SMM, the analyses of this large contemporary cohort showed that FA is an independent predictor for HDP. Counseling on potential maternal risk should be considered in pregnancy with FA.

**3:30 PM – 5:00 PM**

## 1141 | **Clinician Perspectives on Antidepressant Use in Pregnancy: Unpacking Barriers and Racial Disparities in Medication Continuation**

Kelly B. Zafman^1^; Sara Kornfield^2^; Sindhu K. Srinivas^3^; Beth L. Pineles^1^; Emily S. Miller^4^; Rebecca F. Hamm^5^

^1^University of Pennsylvania, Philadelphia, PA; ^2^Hospital of the University of Pennsylvania, Hospital of the University of Pennsylvania, PA; ^3^Department of Obstetrics and Gynecology, Perelman School of Medicine, Maternal and Child Health Research Center, Philadelphia, PA; ^4^Women & Infants Hospital of Rhode Island / Warren Alpert Medical School of Brown University, Providence, RI; ^5^Perelman School of Medicine, University of Pennsylvania, Philadelphia, PA

**Objective**: Untreated maternal depression leads to significant maternal morbidity/mortality, yet more than half of patients stop antidepressants in pregnancy. Patients of color are less likely to continue medications compared to white patients. Our objective was to evaluate clinician perceptions on antidepressant use in pregnancy, with an equity focus.

**Study Design**: This two‐site, mixed‐methods (quant‐ >QUAL) study included providers of perinatal and mental health care from 2‐5/2025. All clinicians completed the Acceptability of Intervention Measure (AIM) to evaluate acceptability of patients continuing antidepressants during pregnancy (Likert; range 4‐20). Clinicians were purposively sampled by specialty/AIM score for individual interviews, which explored barriers/facilitators to antidepressant continuation and its relationship to race/equity. The Consolidated Framework for Implementation Research 2.0 and Health Equity Frameworks were used for coding.

**Results**: 147 clinicians completed the survey, including 85 (58%) OBGYNs/CNMs, 26 (18%) psychiatrists, 22 (15%) family medicine, and 14 (10%) internal medicine clinicians. Acceptability was high but differed by specialty; IM clinicians had lower AIM vs. others (15.2 ±2.6 vs 17.5 ±2.6, *p* = 0.02). All clinicians reported that patient race did not influence their counseling. In interviews (*n* = 15), facilitators of antidepressant continuation included clinician belief in medication efficacy/safety, risks of discontinuation, and prioritization of mental health. Barriers included lack of time, knowledge gaps on specific medications, and patient preconceived notions (Table 1). Clinicians were not surprised about differences in discontinuation by race, and suggested standardized counseling, culturally sensitive resources, and racially concordant care as tools to improve equity (Table 2).

**Conclusion**: This study characterized the acceptability of antidepressant use in pregnancy across clinician specialties. These data, alongside ongoing work on patient perspectives, will inform future equity‐focused strategies to promote appropriate continuation of antidepressants in pregnancy.

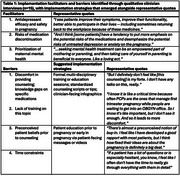

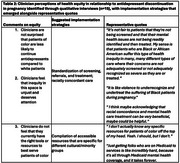

**3:30 PM – 5:00 PM**

## 1142 | **Not all Cerclages Are Equal: A High‐Volume Cohort Reveals Indication‐Driven Differences in Pregnancy Outcomes**

Keren H. Kogan^1^; Doron Kabiri^2^; Tamar Greenstein Wasser, N/A^3^; Netta Nebenzahl‐Berkovits, N/A^4^

^1^Hadassah Ein Kerem, jerusalem, Yerushalayim; ^2^Hadassah Medical Center and Hebrew University of Jerusalem, Jerusalem, Israel, Hadassah Medical Center and Hebrew University of Jerusalem, Jerusalem, Israel, Yerushalayim; ^3^The Hebrew University of Jerusalem, jerusalem, Yerushalayim; ^4^Tel Aviv University, Tel Aviv University, Tel Aviv

**Objective**: To evaluate whether pregnancy outcomes differ among women undergoing cervical cerclage based on the indication for the procedure: history‐indicated, ultrasound‐indicated, or physical examination–indicated cerclage.

**Study Design**: We conducted a retrospective cohort study of all women who underwent cervical cerclage at our institution between 2014‐2024. Women were classified according to cerclage indication: History‐indicated, Ultrasound‐indicated, or Physical examination–indicated. Baseline characteristics and pregnancy outcomes were extracted from electronic medical records. Descriptive statistics compared demographic and obstetric history variables between groups. The primary outcome was preterm birth (<37 weeks). Adjusted risk ratios (aRR) were estimated using log‐binomial regression for parity and history of prior preterm birth.

**Results**: Of 440 women, 349 (71.8%) had history‐indicated cerclage, 79 (16.3%) ultrasound‐indicated, and 12 (2.5%) physical examination–indicated cerclage, often performed in women presenting with advanced cervical dilation or bulging membranes. Median gestational age at delivery was 38.4, 37.9, and 37.6 weeks, respectively (*p* = 0.0038). Preterm birth (<37 weeks) occurred in 23.8%, 36.7%, and 41.7% of the groups (*p* = 0.031).  After adjusting for previous preterm birth history and parity, women receiving ultrasound or physical examination–indicated cerclage had a significantly increased risk of preterm birth compared with the history‐indicated cerclage group (aRR 1.53, 95% CI 1.07–2.18, *p* = 0.019). Secondary outcomes followed a similar trend, with the poorest outcomes observed in the ultrasound and physical examination indicated groups.

**Conclusion**: Indication for cervical cerclage is strongly associated with pregnancy outcomes. Women undergoing ultrasound or physical examination–indicated cerclage remain at a higher risk of preterm birth despite intervention compared with those receiving prophylactic cerclage. These findings highlight the importance of early identification of high‐risk women who may benefit from timely prophylactic cerclage placement.

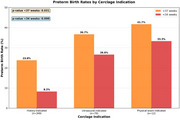

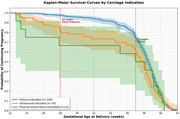

**3:30 PM – 5:00 PM**

## 1143 | **Pregnancy Outcomes in Patients With Cystic Fibrosis: A Multicenter Retrospective Study**

Jasmine A. Rosa^1^; Kimberley Lepe^2^; Sallie Canumay^3^; Bryan Oshiro^4^; Sarah Smithson^5^; Daniel Novak^6^; Leonard Soloniuk^7^

^1^University of California, Riverside, Riverside, CA; ^2^University of California, Riverside School of Medicine, Riverside, CA; ^3^Riverside University Health System, Riverside University Health System, CA; ^4^Department of Obstetrics and Gynecology, Riverside University Health System, Moreno Valley, CA, CA; ^5^Department of Obstetrics and Gynecology, Riverside University Health System, Riverside University Health System, CA; ^6^University of California Riverside, Riverside, University of California Riverside, Riverside, CA; ^7^Department of Gynecology and Obstetrics, Riverside University Health System, Moreno Valley, CA

**Objective**: Cystic fibrosis (CF) is a chronic genetic disorder resulting from a mutation of the *CFTR* gene, historically linked to reduced fertility and adverse pregnancy outcomes. As survival improves, more individuals with CF are pursuing pregnancy, though data on maternal and obstetric outcomes remain limited. We hypothesize that CF increases the risk of adverse maternal outcomes.

**Study Design**: We conducted a study using TriNetX, a large database of de‐identified electronic patient health records, compiled with the HIPAA Privacy Rule. We used the US Collaborative Network, which includes 169,411,996 patients across 67 healthcare organizations. Pregnant patients with CF were compared to a matched cohort without CF. Outcomes included preterm delivery, preeclampsia, gestational diabetes mellitus, sepsis, eclampsia, chorioamnionitis, postpartum hemorrhage, and maternal mortality. Outcomes were analyzed within 45 weeks of pregnancy diagnosis, except for mortality, which was assessed within 1 year. Propensity score matching (PSM) of demographics and comorbidities was performed. Statistical analyses included risk ratio, two‐tailed t‐test and 95% confidence intervals with a p‐value of < 0.05 defining significance.

**Results**: Average age of the cohorts were [29.7+/‐ 16] for the CF cohort and [29.8+/‐ 16.2] for the control cohort. Compared to controls, patients with CF had significantly higher rates of preterm delivery [Risk Ratio: (RR) = 1.7, (1.266, 2.266), *p* =  .0003], gestational diabetes mellitus [RR = 1.4, (1.15,1.67) *p* =  .0006], sepsis [RR = 3.3, (2.247, 4.891), *p* <  .0001], and mortality [RR = 2.9, (1.942, 4.43), *p* <  .0001]. Controls had significantly higher rates of preeclampsia [RR =  .78, (.673,  .901) *p* =  .0007]. There was no significant difference in rates of eclampsia, chorioamnionitis or postpartum hemorrhage.

**Conclusion**: Pregnant patients with CF are at increased risk for preterm delivery, gestational diabetes mellitus, sepsis, and maternal mortality, but were associated with lower rates of preeclampsia. These findings underscore the need for multidisciplinary care and close monitoring of this vulnerable population.

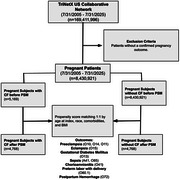

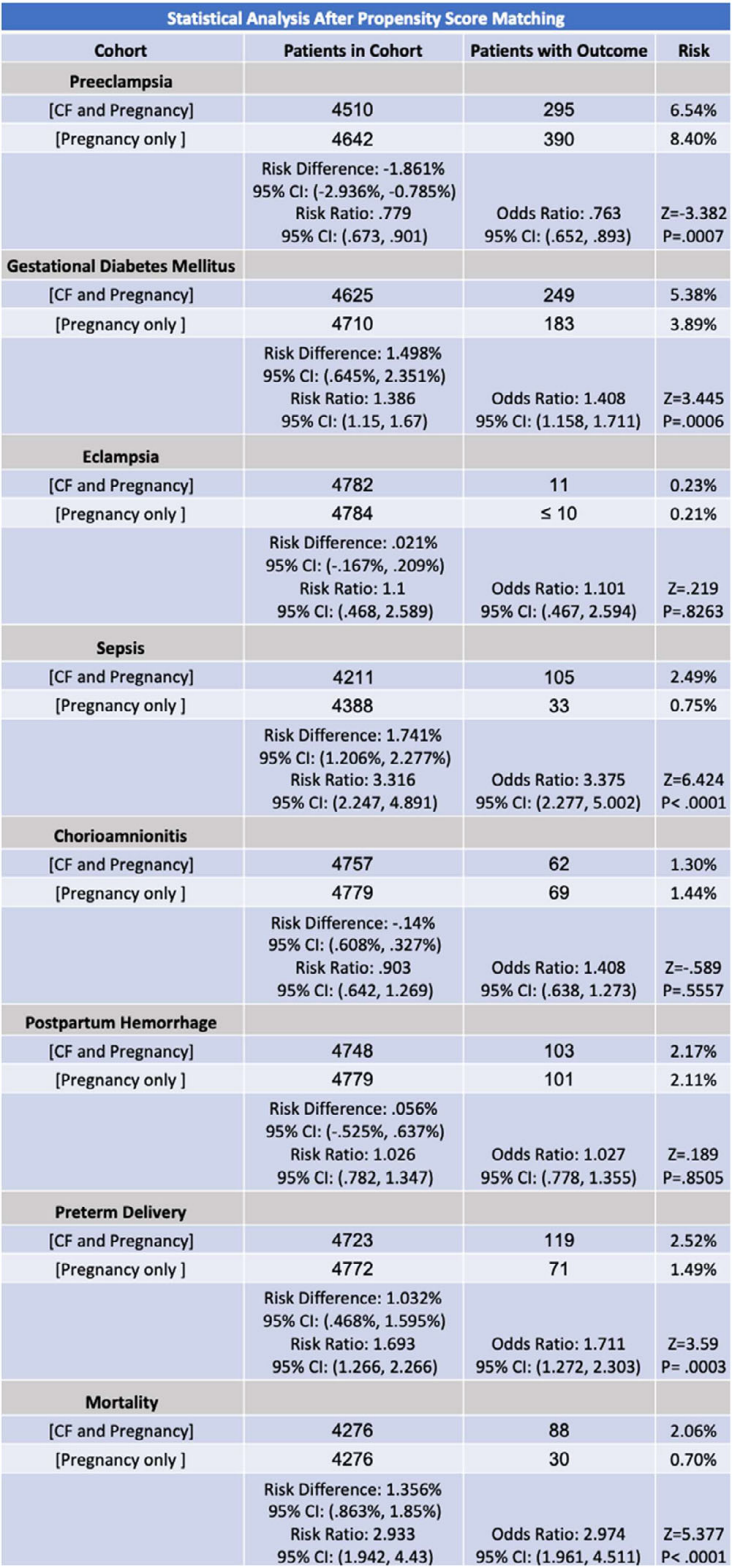

**3:30 PM – 5:00 PM**

## 1144 | **Rapid PlGF: A Pilot Study of Clinical Utility**

Kimen S. Balhotra^1^; Natalie Neff^2^; John W. Hotra^3^; Baha M. Sibai^1^; Jacqueline G. Parchem^4^

^1^Division of Maternal‐Fetal Medicine, Department of Obstetrics, Gynecology and Reproductive Sciences, McGovern Medical School at UTHealth Houston, Houston, TX; ^2^Division of Maternal‐Fetal Medicine, Department of Obstetrics, Gynecology and Reproductive Sciences, McGovern Medical School at UTHealth Houston, McGovern Medical School at UTHealth Houston/ Houston, TX; ^3^McGovern Medical School at UTHealth, Houston, TX; ^4^Division of Maternal‐Fetal Medicine, Department of Obstetrics, Gynecology and Reproductive Sciences, McGovern Medical School at UTHealth Houston, McGovern Medical School at UTHealth Houston/Houston, TX

**Objective**: Placental growth factor (PlGF) is a key biomarker of placental dysfunction. Traditional assays require centralized lab processing, delaying results. The Ronia™ platform (Research Use Only) enables rapid, point‐of‐care PlGF testing from a finger‐prick sample, with results in 30 minutes. This pilot study evaluated its feasibility in ruling out placental dysfunction by assessing associations between PlGF levels and delivery timing, indication, and outcomes in pregnancies with suspected preeclampsia (PE).

**Study Design**: We assessed PlGF in singleton pregnancies at 20–34.6 weeks with suspected PE using the Ronia™ rapid PlGF assay. Clinical outcomes were obtained by chart review. The primary outcome was medically indicated delivery for placental dysfunction within 7 or 14 days of testing. The results were stratified with preliminary concentration PlGF level cut‐offs: low = <20; indeterminate = 20–60; and normal = >60 pg/mL.

**Results**: 85 patients were enrolled: 34 had low PlGF, 9 were indeterminate, and 42 were normal. Baseline characteristics were similar. PlGF <60 pg/mL showed high sensitivity and negative predictive value (NPV) for ruling out delivery (Table 1). Within 14 days, 61.8% with low PlGF delivered, compared to 55.6% indeterminate and 16.7% normal (*p* < 0.001). Medically indicated deliveries and contraindications to expectant management were more frequent in the low vs normal group (20.6% vs 2.4%, *p* = 0.037; 29.4% vs 4.8%, *p* = 0.014). Composite delivery indication for placental dysfunction within 14 days was more common with low PlGF (50.0% vs 7.1%, *p* < 0.001). Adverse maternal/neonatal outcomes trended higher in low PlGF groups but were not statistically significant (Table 2).

**Conclusion**: In this pilot study, a novel point‐of‐care PlGF test showed high sensitivity and NPV for ruling out medically indicated delivery within 7 and 14 days with rare adverse outcomes among those with normal PlGF levels. These findings support the feasibility and potential of rapid PlGF testing to improve triaging, reduce unnecessary interventions, and better target care for high‐risk pregnancies.

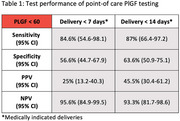

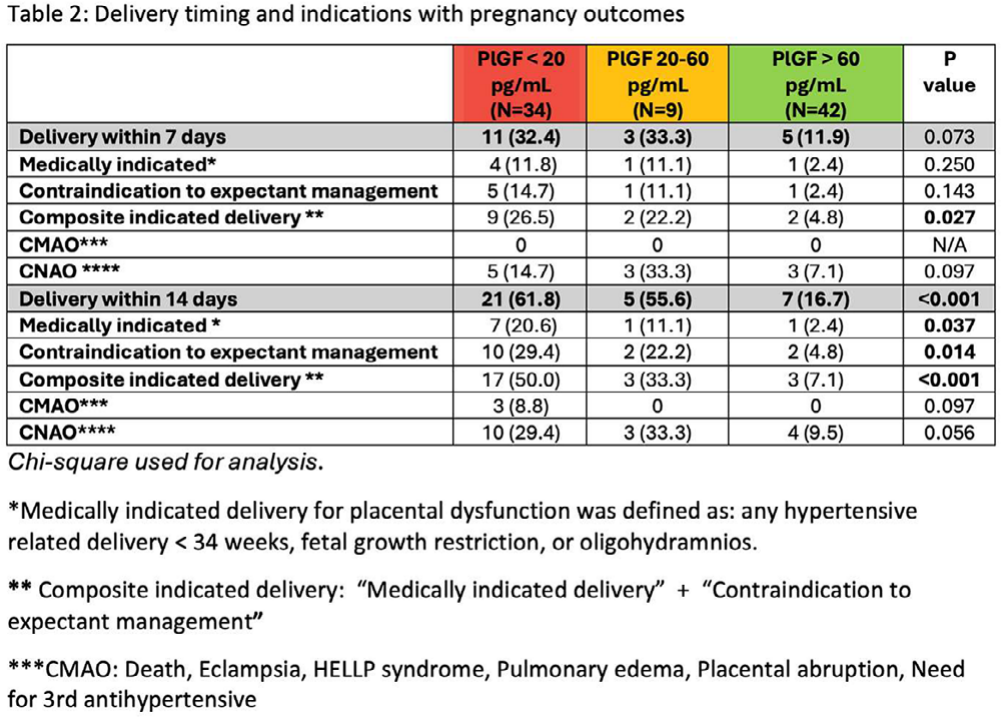

**3:30 PM – 5:00 PM**

## 1145 | **Threshold for Acute Treatment of Severe Hypertension: Before and After Quality Improvement Study**

Kristen A. Cagino^1^; Cabrina I. Becker^2^; Emily Hyde^3^; Beverly Red^2^; Christina Cortes^2^; Anthony Chartier^1^; Claudia Pedroza^4^; Chenyue Huang^5^; Suneet P. Chauhan^6^; Hector Mendez‐Figueroa^4^; Sean C. Blackwell^4^; Baha M. Sibai^4^

^1^Department of Obstetrics, Gynecology and Reproductive Sciences, McGovern Medical School at UTHealth Houston, Houston, TX; ^2^UT Houston, Houston, TX; ^3^UTSW, Dallas, TX; ^4^Division of Maternal‐Fetal Medicine, Department of Obstetrics, Gynecology and Reproductive Sciences, McGovern Medical School at UTHealth Houston, Houston, TX; ^5^McGovern Medical School at UTHealth Houston, Houston, TX; ^6^Delaware Center for Maternal‐Fetal Medicine of Christiana Care, Newark, DE

**Objective**: There is limited evidence for the optimal systolic blood pressure (SBP) threshold for acute treatment of severe hypertension (HTN) in pregnancy. Although SBP ≥ 160 mmHg is used in pregnancy, guidelines for non‐pregnant adults support SBP ≥ 180.  We compared outcomes using SBP threshold ≥ 180 mmHg versus the current threshold of ≥ 160.

**Study Design**: We performed a Quality Improvement before and after non‐inferiority study (NCT05881252) of singletons ≥ 20 weeks’ with SBP ≥ 160 mmHg at a level IV hospital. In the before group (10 months), acute antihypertensives (IV labetalol/hydralazine or rapid‐acting oral Procardia) were given for persistent SBP ≥ 160 mmHg (or DBP ≥ 110). In the after group (10 months), oral maintenance antihypertensives (labetalol or long‐acting Procardia) were given if persistent SBP between 160‐179 mmHg (DBP <110) and acute antihypertensives for persistent SBP ≥ 180 mmHg (or DBP ≥ 110). We excluded if 1) history of or active seizure, stroke (CVA), congestive heart failure (CHF), pulmonary edema, acute kidney injury (AKI) or myocardial ischemia (MI) 2) HELLP syndrome or platelets <100 x 10^9^/L or 3) neurologic symptoms. The primary outcome was a composite of CHF/pulmonary edema, CVA, AKI, or MI. Bayesian analysis was performed using a non‐inferiority margin of 4%.

**Results**: From May 2023 to February 2025, we studied 1003 individuals with severe HTN. There were fewer SBP ≥ 160 mmHg (*p* < 0.01) and a decrease in acute antihypertensives in the after group (170 per 100 individuals in the before versus 61 per 100 individuals in the after group, *p* < 0.01). The primary outcome occurred in 2.9% of the before and 2.0% of the after group (0.76 median relative risk, 95% credible interval 0.37‐1.55) with a 78% probability of risk reduction in the after group and 100% probability that the risk difference in the after group was <4%.

**Conclusion**: In our study, using a SBP threshold of ≥ 180 mmHg for acute treatment of severe HTN led to a non‐inferior rate of maternal outcomes and decreased use of IV/rapid acting oral medication. This has implications in resource utilization and healthcare burden.

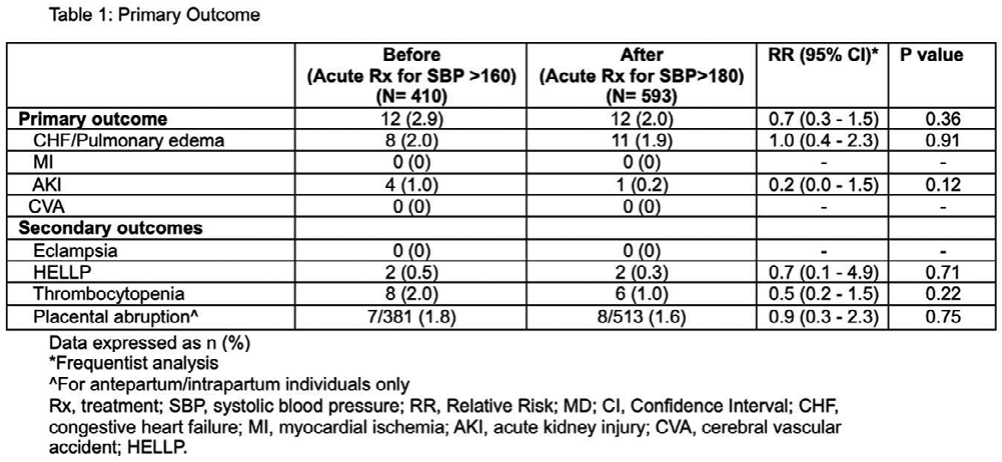

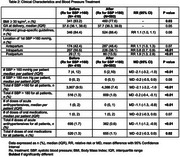

**3:30 PM – 5:00 PM**

## 1146 | **Menstrual Discs for Cervicovaginal Cellular Phenotyping: A Novel Methodology to Define Tissue‐Specific Adaptations in Pregnancy**

Lauren Anton^1^; Olha Kholod^2^; Olivia Palmer^2^; Kristin M. Klohonatz^1^; Zoe Graskin^1^; Rita Leite^1^; Britt A. Goods^2^; Kristin D. Gerson^3^

^1^University of Pennsylvania Perelman School of Medicine, Philadelphia, PA; ^2^Dartmouth College, Hanover, NH; ^3^University of Pennsylvania, Hospital of the university of Pennsylvania, PA

**Objective**: Cervical remodeling is a critical biologic process in preterm and term birth. Mechanisms underlying these tissue dynamics remain incompletely understood though likely involve changes in cervical epithelial cell subtypes across gestation. How these cell signatures differ from those outside of pregnancy is also unknown. We used self‐inserted Softdisc menstrual discs as a novel approach to profile the cellular composition of the pregnant cervix.

**Study Design**: Softdiscs were collected from non‐pregnant (NP) non‐menstruating and pregnant participants enrolled in an ongoing preterm birth study. Single cell RNA‐sequencing (scRNA‐seq) was performed on cells isolated from discs. Standard Seurat based workflows identified cell clusters and cell type enrichment (PangaloaDB and EnrichR). Supervised differential expression analysis and pathway analysis (Metascape) were performed.

**Results**: 27 discs were collected. scRNA‐seq was performed for 5 pregnant and 7 NP samples. Characteristics were similar between groups with respect to age (26.8 years, SD 2.6) and race. Mean gestational age at disc collection was 20.6 weeks (SD 2.6) for pregnant participants. After processing, 24,976 cells were isolated in total. Cell viability was ∼40% with no correlation to the sampled population. There was a 10‐fold higher cell recovery in pregnant individuals. Two large clusters and seven sub‐clusters of cells were identified (Figure 1). The majority of cells were epithelial with a smaller cluster of myeloid cells. Pathway analysis of the top upregulated genes in pregnant vs NP revealed pathways related to cytokine signaling, leukocyte influx, and bacterial defense (Figure 2).

**Conclusion**: We demonstrate feasibility of a novel noninvasive sampling methodology to define cell signatures at a critical biologic site, with increased immune activation being a hallmark of pregnancy physiology in the cervicovaginal space. Molecular phenotyping of exfoliated epithelial and immune cells in high‐risk populations using this approach may be leveraged in the future to unveil cellular regulators of premature cervical remodeling. 1R01HD114611‐01 (KDG)

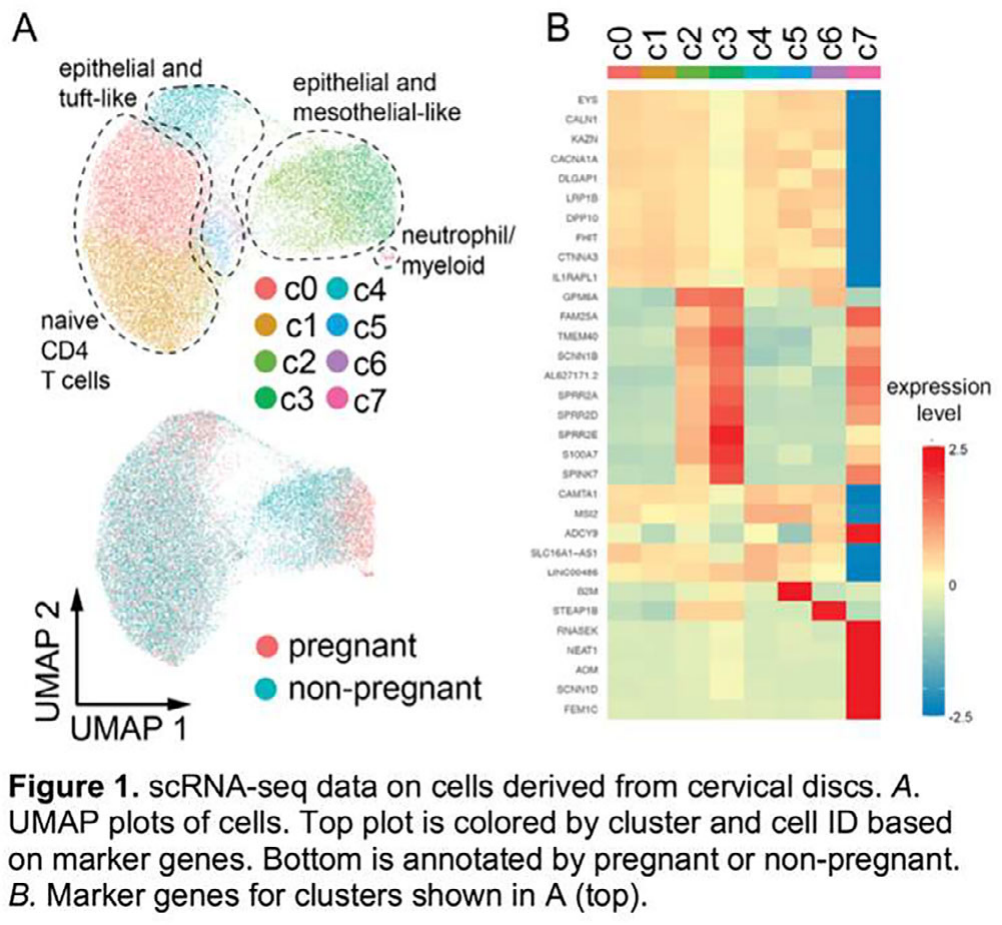

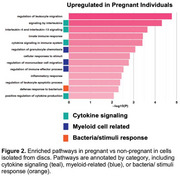

**3:30 PM – 5:00 PM**

## 1147 | **Observed Thromboelastography (TEG) Values in Healthy Postpartum Individuals Compared to Non Pregnant Individuals**

LaKeisha C. Majette^1^; Rebecca Purvis^2^; Kaitlin Warta^3^; Rachel Parker^4^; Geoffrey Slaughter^5^; William M. Johnstone, Jr.^5^

^1^Novant Health New Hanover Regional Medical Center, Wilmington, NC; ^2^University of Tennessee Health Science Center College of Medicine‐ Knoxville, Knoxville, TN; ^3^Novant Health NHRMC, Wilmington, NC; ^4^Lexington Medical Center, Columbia, SC; ^5^New Hanover Regional Medical Center, Wilmington, NC

**Objective**: Thromboelastography (TEG) is a method for assessing clotting and fibrinolysis that allows for real‐time assessment of a patient's coagulative status. We sought to evaluate the TEG profile of healthy postpartum individuals and compare values to established reference values for the non‐pregnant population, to better characterize postpartum coagulation dynamics.

**Study Design**: This was a prospective, cross‐sectional study of 25 healthy individuals in the postpartum period (≥5 weeks after delivery). TEG parameters—Reaction Time (R), Clot Kinetics (K), Clot Angle (Angle), Maximum Amplitude (MA), Lysis at 30 minutes (LY30), and Functional Fibrinogen (FF)—were obtained using the TEG 5000 analyzer (Citrated Kaolin and Functional Fibrinogen assays). Postpartum TEG values were compared to established literature‐based reference ranges for healthy non‐pregnant individuals using the Wilcoxon Signed Rank Test. Bootstrapped confidence intervals (*n* = 2,000) were simulated for further validation.

**Results**: Significant differences were observed between postpartum and non‐pregnant TEG values. R, K, and LY30 are significantly lower for postpartum individuals (R: 4.82‐6.03 versus 5.0‐10.00, *p* = < 0.0001; K: 1.36‐1.97 versus 1.0‐3.0, *p* = 0.0005; LY30 0.57‐2.60 versus 0.00‐8.00, *p* = 0.0004). Angle, MA, and FF were significantly higher for postpartum individuals (Angle 62.50‐69.88 versus 53.00‐72.00, *p* = 0.0056; MA 62.23‐66.22 versus 50.00‐70.00, *p* = 0.0008; FF 375.73‐440.48 versus 200.00‐444.90, *p* = 0.0001). Bootstrapped estimates confirmed deviation, with postpartum point estimates consistently at or near the extremes of non‐pregnant ranges.

**Conclusion**: The postpartum period is characterized by a persistent hypercoagulable state as demonstrated by sustained alterations in TEG values R, K, LY30, Angle, MA, FF compared to non‐pregnant individuals. These findings have important implications for postpartum hemorrhage management. Notably, functional fibrinogen remains markedly elevated, suggesting continued prothrombotic potential beyond delivery. Postpartum‐specific TEG reference values may be necessary for accurate assessment.

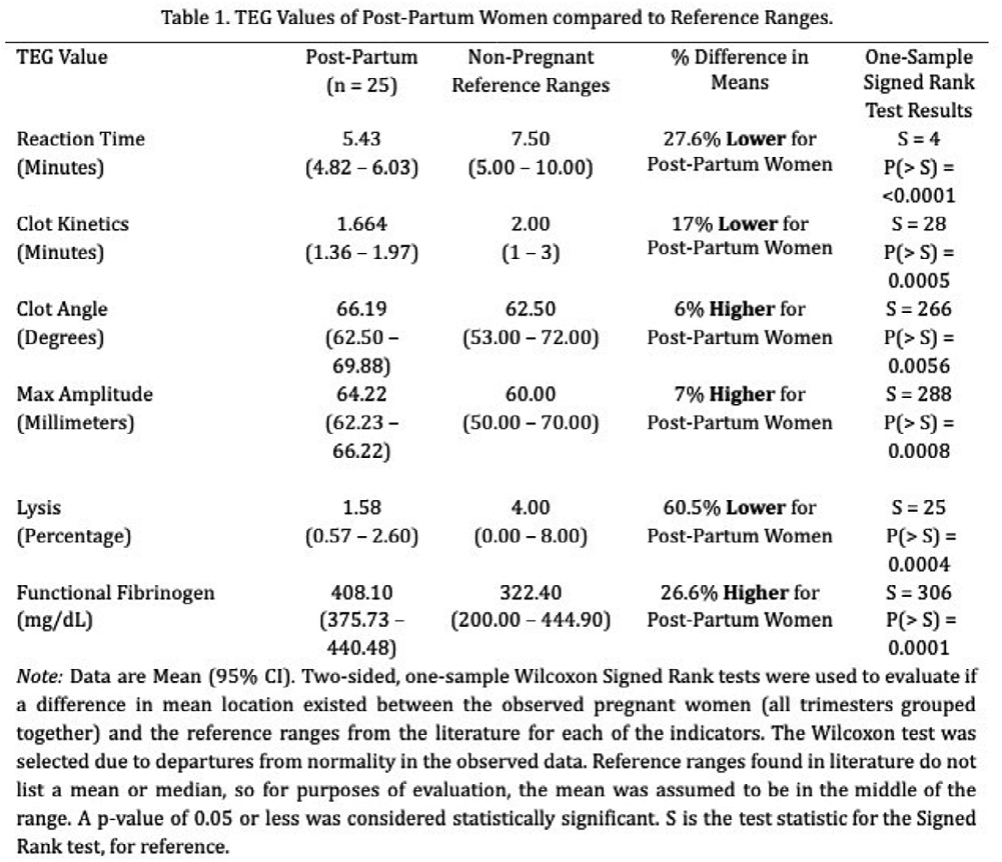

**3:30 PM – 5:00 PM**

## 1148 | **Enhanced Screening via EMR Portal for Detection of Perinatal Mood and Anxiety Disorders**

Laura M. Carlson^1^; Ellen McAlpine^2^; Anna Baker^3^; Jessica H. Britt^4^; Neha Hudepohl^1^

^1^Prisma Health, Greenville, SC; ^2^USC School of Medicine ‐ Greenville, Greenville, SC; ^3^Clemson University, Clemson, SC; ^4^Prisma Health/University of South Carolina School of Medicine Greenville, Greenville, SC

**Objective**: To evaluate whether screening via Epic MyChart patient portal each trimester and twice postpartum (enhanced screening, ES) improves detection of perinatal mood and anxiety disorders (PMADs) in pregnant and postpartum people compared to routine clinic‐based screening (RC) via a pilot trial.

**Study Design**: Pregnant people presenting for prenatal care were randomized to RC or ES in this pilot study. Inclusion criteria included English or Spanish speaking and US‐confirmed viable IUP< 14w6d. Exclusion criteria include age< 16, current psychiatric care or treatment with psychotropic medication or inability to access MyChart. ES participants received automated MyChart screening prior to prenatal visits at 12‐14, 24‐28, and 36 weeks and 2 and 4 weeks postpartum via Edinburgh Postnatal Depression Scale and Generalized Anxiety Disorder‐7 questionnaires. Results were sent to the provider for the patient's next visit; clinical care was not dictated by protocol and patients were treated and referred as clinically appropriate.

The primary outcome was identification of clinically diagnosed PMADs defined by screening measures, ICD‐10 code, initiation of SSRI or other antidepressant medication, and/or referral for psychiatric services. Secondary analyses included frequency of psychiatric referral and medication initiation.

**Results**: 133 people were enrolled, 70 in ES and 63 in RC. The population was racially and ethnically diverse; demographics were balanced (Table 1). Medical complications of pregnancy were more prevalent in RC. PMAD diagnoses were more prevalent in ES (27.5% vs 15.9%) but did not reach statistical significance (aOR 2.08 (0.98, 8.05), Figure 1). Anxiety disorders were diagnosed with increased frequency in ES vs RC (aOR 3.26 (1.11, 9.55)). Psychiatric referral and medication initiation were more common in ES though this did not reach statistical significance (aOR 2.12 (0.7, 6.49) and aOR 2.05 (0.62, 6.79) respectively).

**Conclusion**: ES results in increased detection of anxiety disorders has potential to improve detection and treatment of all PMADs. A larger RCT is warranted.

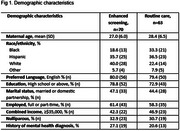

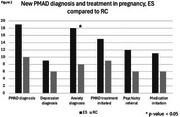

**3:30 PM – 5:00 PM**

## 1149 | **Facilitators and Barriers to Aspirin Implementation for Prevention of Preeclampsia in Nigeria**

Laura Crook^1^; Kabiru Umar Ibrahim^2^; Nura Abdulkarim^3^; Chukwuebuka F. Okoye^4^; Adaego Orji^4^; Cecilia Nartey^5^; Kamilu Karaye^2^; Kathryn J. Lindley^6^; Victor Davila‐Roman^5^; Dike Ojji^4^; Mark Huffman^5^; Zainab Mahmoud^5^

^1^Washington University in St. Louis School of Medicine, St Louis, MO; ^2^Department of Medicine, Aminu Kano Teaching Hospital, Kano, Kano; ^3^Murtala Muhammad Specialist Hospital, Kano, Kano; ^4^Cardiovascular Research Unit, University of Abuja and University of Abuja Teaching Hospital, Gwagwalada, Abuja, Federal Capital Territory; ^5^Department of Medicine, Cardiovascular Division and Global Health Center, Washington University in St. Louis, St. Louis, MO; ^6^Department of Medicine, Division of Cardiology, Vanderbilt University Medical Center, Nashville, TN

**Objective**: Nigeria has one of the highest rates of maternal morbidity and mortality globally, with hypertensive disorders of pregnancy (HDP), including preeclampsia, causing one‐third of all maternal deaths. While aspirin prophylaxis is an effective intervention for preventing preeclampsia and its subsequent morbidity and mortality, aspirin utilization in Nigeria remains unclear. This study aims to identify the key facilitators and barriers to aspirin implementation, providing insights to inform strategies to enhance its adoption.

**Study Design**: Between August 2024 and November 2024, 18 in‐depth interviews and 8 focus group discussions were conducted with healthcare workers (obstetricians, cardiologists, nurses), patients, and administrators across four tertiary hospitals in Nigeria. Data were collected in English and Hausa, transcribed, translated, and analyzed using Dedoose software. Using the updated Consolidated Framework for Implementation Research, qualitative data were analyzed for barriers and facilitators to aspirin implementation across five domains: individual, inner setting, outer setting, innovation, and implementation process.

**Results**: Identified facilitators to implementation included the multidisciplinary expertise available at these tertiary centers, motivation for change driven by the recognized severity of HDP, high capacity for research, and alignment with current practices. Barriers included limited awareness among both patients and providers, overburdened healthcare systems and providers, and financial constraints at the individual level. Implementation strategies highlighted education/training for both patients and providers, media campaigns to promote the importance of early antenatal care, increasing the accessibility of aspirin and development of nationwide guidelines around aspirin prophylaxis.

**Conclusion**: This study identified facilitators and barriers to the implementation of aspirin prophylaxis against preeclampsia and proposed implementation strategies to increase aspirin use among pregnant patients at risk of developing HDP.

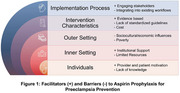

**3:30 PM – 5:00 PM**

## 1150 | **Maternal Substance Use And Altered Maternal and Fetal Immune Responses: Implications for Offspring Neurodevelopmental Risk**

Daehee Han^1^; Prabhat Upadhyay^1^; Laura Ibanez‐Pintor^2^; Olyvia J. Jasset^2^; Sophia J. Feinerman^2^; Caroline G. Bradford^2^; Oscar A. Jimenez^2^; Joshua Remland^2^; Rachel V. Yinger^2^; Amber Bausley^3^; Angela Diaz^4^; Roy H. H. Perlis^5^; Andrea G. Edlow^6^

^1^Vincent Center for Reproductive Biology, Massachusetts General Hospital; Department of Obstetrics and Gynecology, MassGeneral Brigham; Division of Maternal‐Fetal Medicine; Harvard Medical School, Boston, MA; ^2^Vincent Center for Reproductive Biology, Massachusetts General Hospital, Boston, MA; ^3^Howard University College of Medicine, Washington, DC; ^4^Indiana University School of Medicine, Indianapolis, IN; ^5^Center for Quantitative Health, MassGeneral Brigham; Harvard Medical School, Boston, MA; ^6^Department of Obstetrics and Gynecology, MassGeneral Brigham; Division of Maternal‐Fetal Medicine; Harvard Medical School, Boston, MA

**Objective**: Maternal substance use (SU) during pregnancy is known to be associated with adverse neurodevelopmental outcomes in offspring, but the mechanisms underlying this risk remain poorly understood. Here we investigated how maternal substance use is associated with altered maternal and fetal immune responses, as potential mediators of developmental risk.

**Study Design**: Maternal and cord plasma and peripheral blood mononuclear cells were collected from 68 pregnancies, including *N* = 17 pregnant women with SU (primarily opioids, marijuana and cocaine, 41% polysubstance use), matched 3:1 to uncomplicated control pregnancies (*N* = 51) for gestational age, maternal pre‐pregnancy BMI, and fetal sex. We characterized maternal and fetal T cell phenotypes and cytokine production using flow cytometry. Monocyte function was evaluated after LPS stimulation, and cytokine and chemokine concentrations in maternal and cord plasma were quantified. Maternal and fetal immune activation in SU‐exposed cases vs controls was compared using conditional linear regression.

**Results**: Increased frequency of pro‐inflammatory CD4⁺ and CD8⁺ T cells producing IFN‐γ and TNF‐a was observed in both maternal (Figure 1A) and cord (Figure 1B) blood from SU‐exposed pregnancy. Increased levels of pro‐inflammatory chemokines and adhesion molecules, such as MCP‐1 and ICAM‐1, were noted in cord plasma from SU‐exposed pregnancies (Figure 1C). Suppressed or exhausted monocyte function was noted in both maternal and cord blood of SU‐exposed pregnancies, with reduced capacity to produce IL‐6 and MIP‐1β upon stimulation (Figure 1D).

**Conclusion**: Maternal SU is associated with maternal and fetal T‐cell‐mediated immune activation, and innate immune exhaustion, consistent with patterns of immune dysregulation previously described in the setting of obesity or viral infection in pregnancy. In the large national HEALthy Brain Child Development study, we will evaluate how maternal and fetal immune dysregulation in the setting of maternal SU may contribute to adverse long‐term neurodevelopmental outcomes in children.

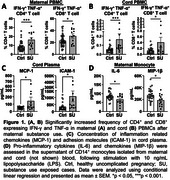

**3:30 PM – 5:00 PM**

## 1151 | **Experiences of L&D Personnel With Values Clarification Workshops Prior to Implementing Induction Abortion Services**

Laura Jacques^1^; Elise Cowley^2^; Laurie Lapp^2^; Anna Altshuler^3^; Jacquelyn Askins^2^

^1^UW Madison SMPH, University of Wisconsin Madison, WI; ^2^UW Madison SMPH, UW Madison SMPH, WI; ^3^UCSF, UCSF, CA

**Objective**: To evaluate the impact of a Values Clarification and Attitude Transformation (VCAT) workshop on labor and delivery (L&D) staff's perspectives about abortion prior to institutional abortion service expansion to L&D.

**Study Design**: We conducted a qualitative analysis of semi‐structured interviews done after VCAT workshops implemented with 192 L&D healthcare workers at a single urban, community‐based, tertiary care center in California. The workshops prompted participants to represent and reflect on diverse abortion viewpoints. 25 nurses, physicians and midwives were interviewed post‐workshop (Figure 1). Transcripts were coded and thematically analyzed.

**Results**: Four major themes emerged: (1) VCAT created space for personal reflection and perspective taking. Participants described how representing others’ views deepened understanding of their own beliefs and fostered empathy. 2) VCAT stimulated reconsideration of professional roles and the purpose of L&D in the context of abortion care. (3) Professional and personal contexts shaped participants’ engagement with the workshop. Factors like compassion fatigue from working in a hospital with many social needs, their or their friends’ abortion experiences and the perceived taboo of talking about abortion at work influenced how participants processed the experience. (4) Respect for autonomy and commitment to patient care emerged as core, shared values, even among those with differing personal beliefs.(Table 1)

**Conclusion**: VCAT workshops provided a structured, safe opportunity for L&D staff to reflect on the complex personal and professional dimensions of abortion care. The process helped participants explore value tensions, clarify their professional roles, and identify shared commitments to patient care. These findings suggest VCAT may be a valuable tool for preparing multidisciplinary perinatal teams to navigate institutional abortion care expansion with empathy, professionalism and cohesion.

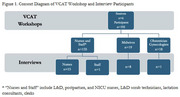

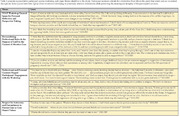

**3:30 PM – 5:00 PM**

## 1152 | **Prenatal Care Utilization in a Novel Hybrid Prenatal Care Model: Mental Health Conditions**

Laura Peyton Ellis^1^; Elizabeth Krans^2^; David M. Haas^3^; Thuy Nguyen^4^; Basil Issac^5^; Chloe Qi^4^; Amy Jiao^4^; Alex Peahl^6^

^1^UConn Health Obstetrics and Gynecology, Farmington, CT; ^2^UPMC Magee‐Womens Pregnancy and Women's Recovery Center, Pittsburgh, PA; ^3^Indiana University, Indiannapolis, IN; ^4^University of Michigan School of Public Health, Ann Arbor, MI; ^5^University of Michigan School of Economics, Ann Arbor, MI; ^6^University of Michigan Department of Obstetrics & Gynecology, Ann Arbor, MI

**Objective**: An increasing number of pregnancies are affected by mental health conditions. Evidence is lacking, however, on how to best provide high‐quality, patient‐centered prenatal care. This work evaluated the effect of the new tailored prenatal care model as described in Clinical Consensus Number 8 “Tailored Prenatal Care Delivery for Pregnant Individuals” in patients with mental health conditions.

**Study Design**: This retrospective cohort study used electronic health records from three academic hospitals that implemented tailored prenatal care during the COVID‐19 pandemic, two of which sustained changes (“intervention”), and one that did not (“control”). Mental health conditions (MH) were defined as generalized anxiety disorder and major depressive disorder diagnoses. The primary outcomes were planned (prenatal visits) and unplanned (ED/triage) utilization. The secondary outcomes were completion of recommended prenatal tests and timeliness of test completion.  Difference‐in‐difference analysis compared pregnancies from pre‐COVID Jan ‘17 to Feb’ 20 to post‐COVID Jun ‘20 to Dec’ 23 excluding a washout period (Mar to May' 20). Difference in difference in difference (DDD) coefficients were reported.

**Results**: Overall, 49,264 pregnancies were included. Anxiety and depression were common in both groups (21% intervention, 30% control). There was a similar decrease in overall visit number for both groups (no MH: ‐1.554, MH: ‐1.785; DDD: ‐0.232, *p* = 0.96) and similar increases in unplanned utilization (no MH = 0.462, MH = 0.438;  DDD: ‐0.24, *p* = 0.27). The completion of tests (no MH: 0.168, MH: 0.61, DDD: ‐0.107, *p* = 0.333) and timely completion of tests (no MH: 0.631, MH: 0.19, DDD: ‐0.612, *p* =  .002) increased in both groups, but increased less for those with MH conditions. The difference was only significant for the timely completion.

**Conclusion**: Patients with mental health conditions face challenges in accessing prenatal care. Results suggest that patients with mental health conditions may not experience the same benefits of tailored prenatal care. Future work is needed to understand the patient experience of this new model.

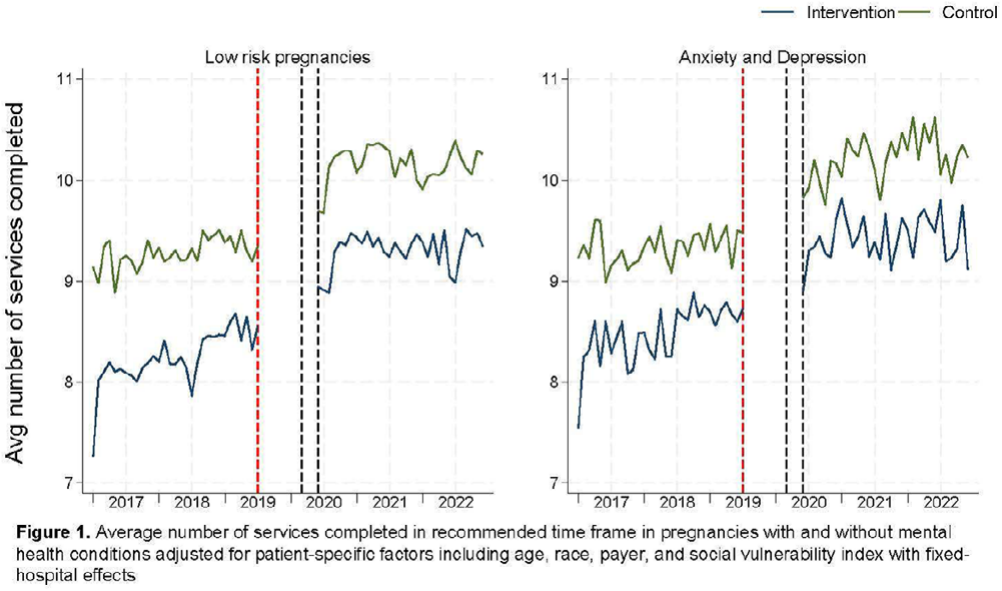

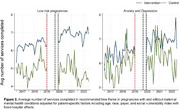

**3:30 PM – 5:00 PM**

## 1153 | **Management of Postpartum Hemorrhage Bundle–Implentation of a Novel PPH Flowsheet**

Lauren Hennis^1^; Madeleine McSpadden^1^; Maria Hankey^1^; Garret Lawhorn^1^; Megan D. Whitham^1^; Ryan Bradley^2^

^1^Virginia Tech Carilion School of Medicine, Roanoke, VA; ^2^Virginia Tech Carilion School of Medicine, roanoke, VA

**Objective**: Postpartum hemorrhage (PPH) remains the most common childbirth complication and leading cause of maternal death worldwide. Lifesaving response requires early recognition, standardized care, and rapid access to uterotonics and blood products. We implemented a quality improvement initiative focused on structured team response using a novel PPH management flowsheet and enhanced hemorrhage alert protocol.

**Study Design**: A hybrid implementation‐effectiveness evaluation using a retrospective‐prospective database included patients delivering from Jan 8, 2024, onward. The PPH Management Flowsheet was introduced June 12, 2024, followed by an alert overhaul on Mar 17, 2025. This included automatic blood bank notification, hemorrhage cart deployment, and a cultural shift in response to the “hemorrhage response”. Interrupted time series analysis assessed hemorrhage, transfusion, and uterotonic use across three epochs:

Epoch 1 (1/8/24–6/11/24): baseline

Epoch 2 (6/12/24–3/16/25): flowsheet

Epoch 3 (3/17/25–4/30/25): alert overhaul

**Results**: Among 4328 deliveries, no differences were noted across epochs in demographics, delivery mode, or preexisting conditions. PPH and severe PPH rates remained high (15% and 7.4%, respectively), with no significant changes in hemorrhage or transfusion rates. Uterotonic use trended upward post‐alert overhaul but was not statistically significant, Figure 1. There was a statistically‐significant upward trend in PPH alert usage over time (*p* = 0.026). Multi‐segmented regression analysis reveals that this is not likely attributable to a singular intervention, Figure 2.

**Conclusion**: Initial implementation suggests improved team recognition and responsiveness to PPH, evidenced by increased uterotonic use and alert activation. The intervention demonstrates progress toward a proactive, standardized hemorrhage response culture.

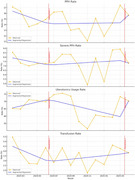

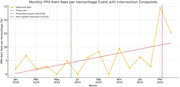

**3:30 PM – 5:00 PM**

## 1154 | **Hospital Readmission Rates for Postpartum Patients in a Remote Blood Pressure Monitoring Program**

Lauren Walheim^1^; Alejandra Barreto^2^; Lisbet S. Lundsberg^3^; Jennifer F. Culhane^3^; Nicole Spaulding^4^; Christine Coffey^4^; Elizabeth Forbes^4^; Caitlin Partridge^3^; Katherine H. Campbell^5^; Anna Denoble^6^

^1^Yale School of Medicine, Yale School of Medicine ‐ Yale University, CT; ^2^Yale School of Medicine, Lynwood, CA; ^3^Department of Obstetrics, Gynecology, and Reproductive Sciences, Yale School of Medicine, New Haven, CT; ^4^Yale New Haven Hospital, New Haven, CT; ^5^Division of Maternal Fetal Medicine, Yale School of Medicine, New Haven, CT; ^6^Yale School of Medicine, New Haven, CT

**Objective**: Hypertensive disorders of pregnancy (HDP) cause significant postpartum (PP) morbidity. Text‐message remote blood pressure (BP) monitoring (RBPM) programs show no difference in readmissions for hypertension (HTN). We aimed to compare readmission rates for HDP patients by engagement with telehealth‐based PP RBPM program.

**Study Design**: Retrospective study of PP RBPM patients from 11/1/2022‐6/30/2024. Eligibility criteria were chronic HTN or HDP. Patients receive BP cuff and scheduled telehealth BP checks, where clinicians follow protocols for BP management. Patients graduate when BP normalizes off therapy or 12 weeks PP. Patients were grouped based on RBPM engagement: 1) declined or enrolled but never attended; 2) attended ≥1 visit but did not graduate; 3) graduated. Primary outcomes were rates of readmission (within 6 weeks PP) for any indication or for HTN. Secondary outcomes were emergency department visits or observations (ED visits) for any indication or HTN. Patient demographics and comorbidites, HTN characteristics, readmissions and ED visits were extracted from the medical record. Groups were compared with chi‐square and ANOVA tests and multivariable logistic regression used to determine odds of the outcomes.

**Results**: 1160 patients were eligible. 508 (43.8%) in Group 1, 195 (16.8%) Group 2, 457 (39.4%) Group 3. HDP diagnoses and magnesium use differed among groups; there were no differences in average mean arterial pressure or rates of stage 2 HTN PP (Table 1). Readmissions occurred in 118 patients (10.2%) for any indication, 91 (7.8%) for HTN. Rates of readmission and ED visits for any indication and HTN all varied significantly between groups (Table 1). After adjusting for confounders, Group 3 had higher odds than Group 1 for readmission for any indication (aOR 3.03, 95% CI 1.90‐4.83), HTN (aOR 2.96, 95% CI 1.87‐4.76) and ED visit for HTN (aOR 3.58, aOR 1.78‐7.17). There was no significant difference between Group 2 and 1 (Table 2).

**Conclusion**: Patients fully engaging with a telehealth‐based RBPM program are readmitted and visit ED more, suggesting overall increased healthcare engagement PP.

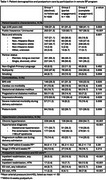

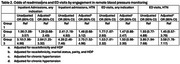

**3:30 PM – 5:00 PM**

## 1155 | **Streamlining Glycemic Control: Subcutaneous Insulin for Intrapartum Diabetes Management**

Leah M. Savitsky; Chantelle Barr; Ronit Katz; Nadine Martinez; Juliet Henderson; Tara Saleh; Leilani White; LaVone Simmons

University of Washington, Seattle, WA

**Objective**: Optimal intrapartum glycemic control for patients with gestational diabetes (GDM) and type 2 diabetes (T2DM) is uncertain. Management usually relies on initiation of intravenous (IV) insulin once a glucose threshold is met. In 2024, our unit implemented a subcutaneous (SC) insulin protocol, replacing the routine initiation of IV insulin. We hypothesized the change would not alter neonatal outcomes and would improve nursing (RN) satisfaction.

**Study Design**: This retrospective cohort quality improvement study included patients with GDM or T2DM who delivered at ≥ 36 weeks between March 2021 and June 2025. The protocol was implemented July 2024; July was excluded as a washout period. Patient demographics, maternal, and neonatal outcomes were compared before and after protocol implementation. The primary outcome was neonatal hypoglycemia or NICU admission for hypoglycemia within 24 hours of life. Secondary outcomes included IV insulin duration and RN satisfaction (3‐item Likert Survey). Modified Poisson regression with interrupted time series assessed time trends and shifts in outcomes. Multivariable logistic regression evaluated associations between insulin modality and the primary outcome. Surveys were analyzed by Wilcoxon rank‐sum tests.

**Results**: Of 995 deliveries that met inclusion criteria, 221 experienced the primary outcome. The composite rate fell from 23% pre‐intervention to 20% post (*p* = 0.225). Each pre‐intervention month was associated with a nonsignificant 0.9% increase in event rate (IRR 1.01, 95% CI 0.99‐1.02). The 65 RN survey responses pre‐intervention and 56 RN surveys post‐intervention indicated improved satisfaction with management (29.2% vs 51.8%, *p* = 0.0324).

**Conclusion**: Implementing an initial SC insulin protocol for managing intrapartum blood glucose did not lead to an increase in neonatal hypoglycemia or related NICU admissions. Nurses reported significantly higher satisfaction with the SC protocol. Initiating SC insulin at the glycemic threshold, rather than automatically starting IV insulin, was found to be a safe and preferred alternative for intrapartum glucose management.

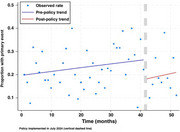

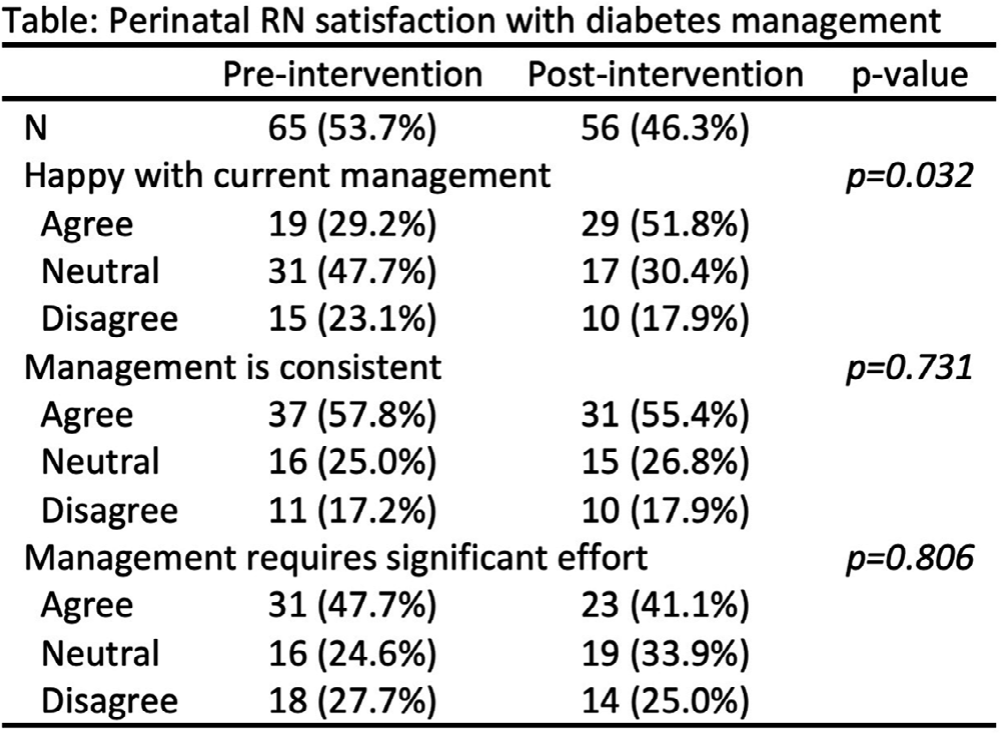

**3:30 PM – 5:00 PM**

## 1156 | **Discrepancies in Perceived Autonomy, Trust and Information Between Women and Healthcare Providers in Childbirth Decision‐Making**

Maya Ronen^1^; Lee Segev^2^; Angela Abulov^2^; Orna Tal^2^

^1^Shamir Medical Center, Shamir medical Center, HaMerkaz; ^2^Shamir Medical Center, Shamir Medical Center, HaMerkaz

**Objective**: To assess the alignment between women's preferences and healthcare providers’ perceptions regarding autonomy, trust and information preferences during decision‐making in childbirth

**Study Design**: A cross‐sectional, questionnaire‐based study was conducted between March and November 2024. Participants included postpartum or laboring women (*n* = 143) and healthcare professionals (*n* = 167; physicians, midwives, nurses). Two parallel questionnaires  of 18 questions were administered assessing trust, level of autonomy in decision‐making preferences, information sources, and communication needs. Responses were rated on 1–10 scales and compared using t‐tests and chi‐square analyses.

**Results**: Women reported greater trust in their regular OB‐GYN (8.3±2.2) and hospital physicians (8.7±1.7) than providers perceived (7.5±1.5, *p* < 0.001). Although 47.2% of women preferred shared decision‐making, which staff accurately estimated (46.9%), significant gaps were found in other domains. Staff overestimated the influence of family/friends (7.4±1.9 vs. 6.2±2.7; *p* < 0.001) and religious authority (6.6±2.3 vs. 4.0±3.1; *p* < 0.001). Women valued information from physicians (9.1±1.4) and scientific literature (6.5±2.8) more than staff assumed (8.0±1.5 and 4.7±2.5, respectively; *p* < 0.001), while staff overestimated reliance on social media (7.3±1.9 vs. 3.7±2.8; *p* < 0.001). Real‐time explanations during labor and informing birth companions were highly prioritized by women (9.2±1.5 and 9.2±1.8), again underestimated by providers (8.3±1.5 and 8.1±1.8, *p* < 0.001).

**Conclusion**: Significant gaps exist between women's preferences and healthcare providers' assumptions regarding autonomy, trust and information in childbirth decision‐making. Addressing these perception gaps may improve communication, patient‐centered care, and patients' satisfaction in obstetric settings.

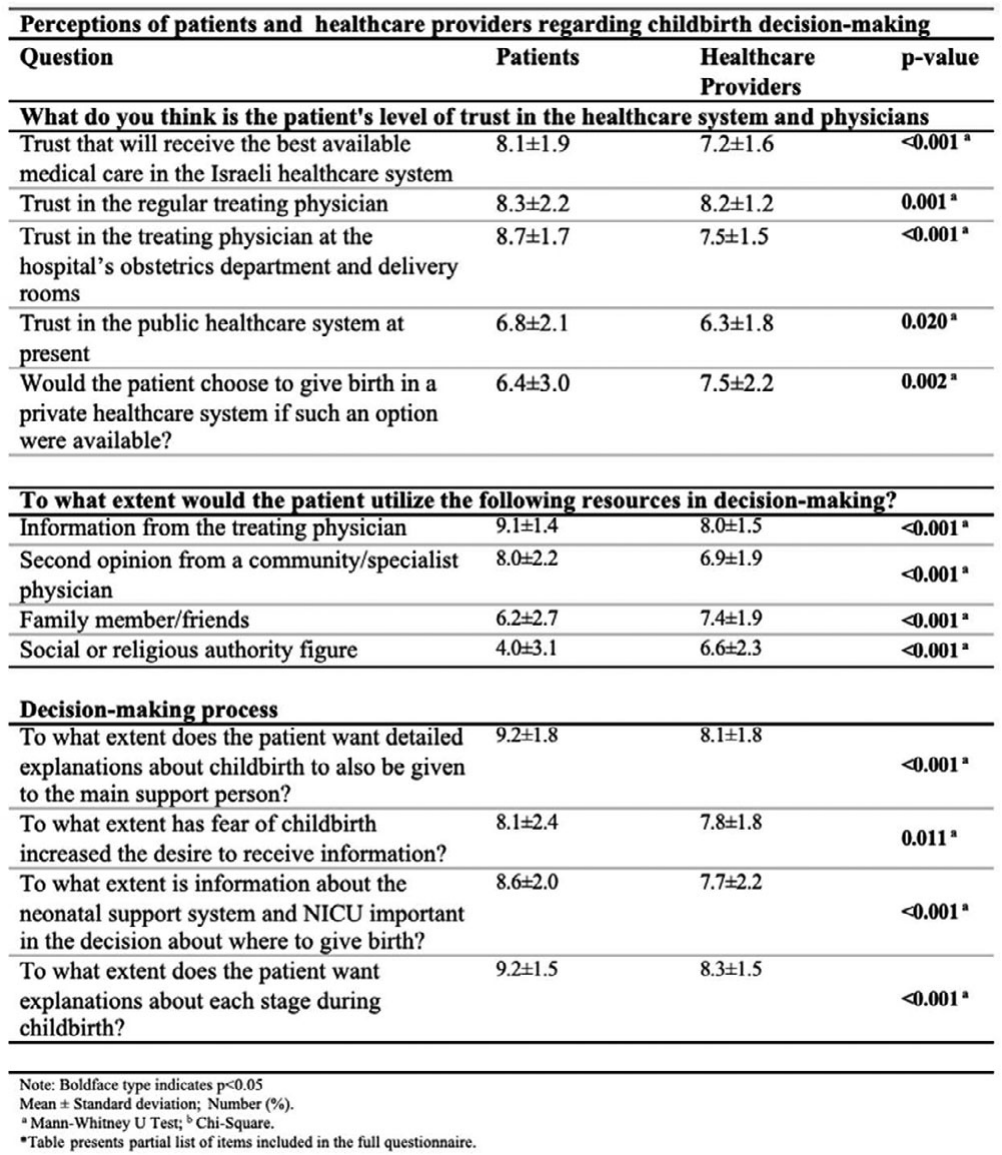

**3:30 PM – 5:00 PM**

## 1157 | **Postpartum Electrocardiogram Parameters in Patients Affected and Unaffected by Cardiovascular Dysfunctions**

Lihong Mo^1^; Arielle Yoo^2^; Gizem Akildiz^3^; Shantanu Milind Joshi^4^; Philip Strong^1^; Sonul Gupta^1^; Imo Ebong^5^; Uma Srivasta^5^; Herman Hedriana^1^; Chen‐Nee Chuah^2^

^1^Department of Obstetrics and Gynecology, UC Davis, Sacramento, CA; ^2^Electrical and Computer Engineering, UC Davis, Davis, CA; ^3^School of Medicine, UC Davis, Sacramento, CA; ^4^Computer Science, UC Davis, Davis, CA; ^5^Division of Cardiovascular Medicine, UC Davis, Sacramento, CA

**Objective**: The postpartum period involves rapid fluid shifts that, combined with preexisting cardiovascular disease or hypertensive disorders of pregnancy, increase cardiac stress. As a widely utilized cardiac assessment, we hypothesize that Electrocardiogram (ECG) may reveal critical cardiac changes in the postpartum period and serve as a screening tool prior to echocardiography. This preliminary study aims to compare trends in four ECG parameters—QTc, QRS duration, PR interval, and heart rate —from 50 weeks before to 10 weeks after birth in patients with and without cardiovascular complications.

**Study Design**: This is a retrospective case‐control study of a database that includes 26,453 ECGs on 7637 pregnant individuals aged 18–50 (2011–2024) at a single tertiary center. After excluding patients with pre‐existing cardiac morbidity or hypertensive disorders of pregnancy, 3032 ECGs from 2407 individuals were included to establish normative trends. Ten individuals with heart failure (pre‐existing or peripartum cardiomyopathy) (Figure 1) and 13 patients with preeclampsia who had ECGs from ≥2 gestational periods (pre‐pregnancy, during pregnancy, or postpartum) were separately analyzed (Figure 2).

**Results**: In unaffected pregnancies, PR‐interval, QRS duration, and QTc remained stable. In contrast, patients with heart failure exhibited elevated QRS duration, QTc, and PR interval throughout the pregnancy and a blunt drop in QTc was seen at birth (Figure 1). Patients with preeclampsia demonstrated elevated QTc at birth compared to the unaffected cohort, while an increase of QTc at birth compared to the weeks leading up to birth (Figure 2).

**Conclusion**: Distinct ECG patterns were observed in patients with heart failure and preeclampsia compared to those with unaffected pregnancies. Notably, QTc and QRS duration were elevated throughout gestation in patients with cardiac complications, with abnormal QTc responses around the time of delivery. These findings suggest that serial ECG monitoring may offer early signals of peripartum cardiovascular stress and warrant further investigation as a potential screening tool.

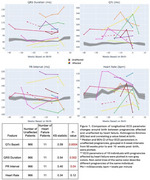

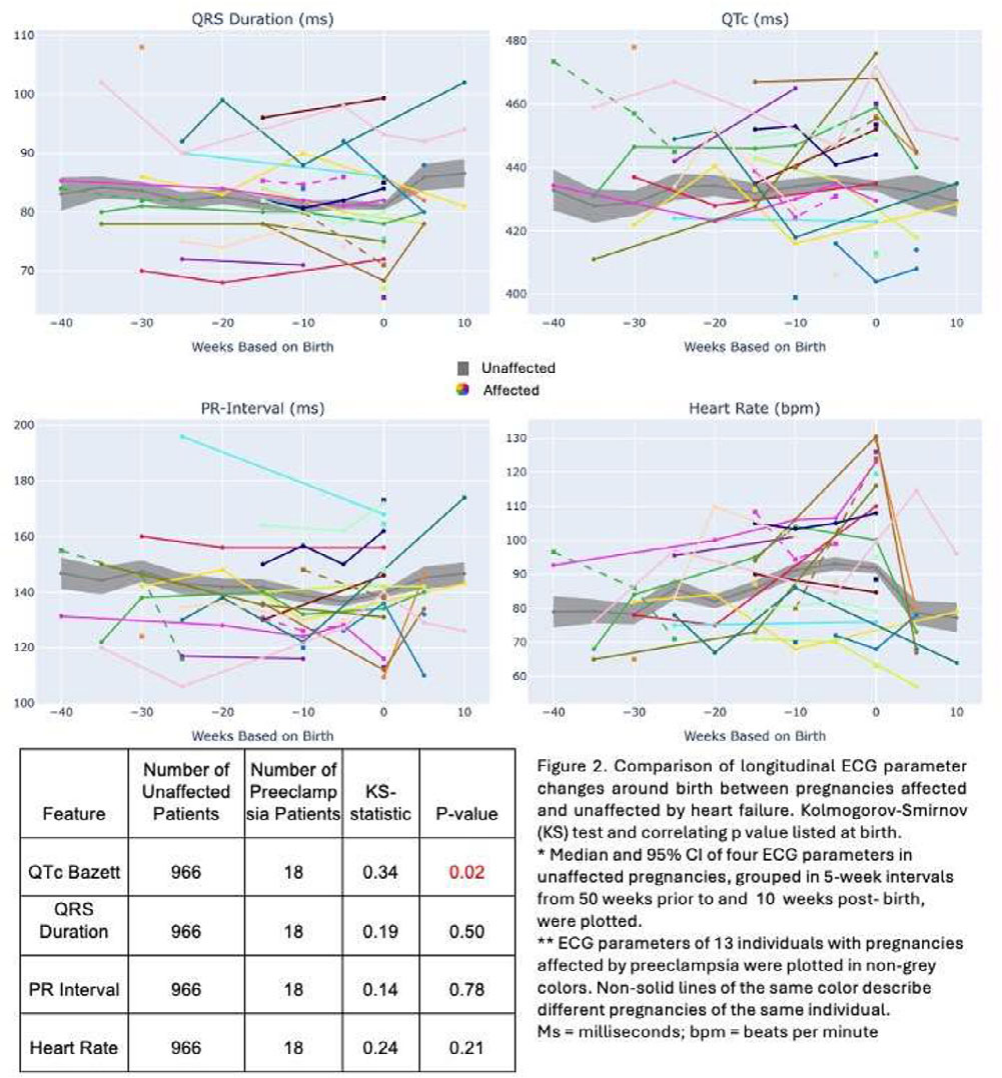

**3:30 PM – 5:00 PM**

## 1158 | **Incidence and Risk Factors associated With Funisitis in Patients With Cesarean Surgical Site Infections**

Lillian Boettcher^1^; Janice Wong^2^; Sara I. Jones^3^; Ysaac Zegeye^3^; Aya Bashi^4^; Jennifer B. Gilner^3^; Jeffrey A. Kuller^3^; Sarah K. Dotters‐Katz^5^

^1^Department of Obstetrics and Gynecology, Duke University School of Medicine, Duke University School of Medicine/Durham, NC; ^2^UCSD Department of Obstetrics and Gynecology, UCSD, CA; ^3^Duke University School of Medicine, Durham, NC; ^4^Duke University Hospital, Durham, NC; ^5^Division of Maternal Fetal Medicine, Department of Obstetrics and Gynecology, Duke University, Duke University/Durham, NC

**Objective**: Surgical site infection (SSI) following cesarean confers significant maternal morbidity. Funisitis, acute inflammation of the umbilical cord, is a histologic finding of neutrophils within Wharton's jelly and is indicative of fetal inflammatory response. This study explores the relationship between funisitis and SSI, attempting to assess if funisitis can be used as a risk stratifying factor for wound complications.

**Study Design**: IRB‐approved retrospective cohort study of patients who delivered via cesarean and developed SSI at a single tertiary care facility between 4/1/2013 and 11/2/2019. Exposure of interest was histologic funisitis on pathologic analysis of the placenta. Demographic data, maternal history, intrapartum information, and wound infection details were abstracted from EMR. Primary outcome was factors associated with funisitis in the SSI population. Secondary analyses assessed rates of severe SSI (i.e. re‐operation in OR), need for wound washout, debridement, readmission, and readmission complications (ICU, sepsis, etc.). Bivariate statistics analyzed the data. Regression models controlled for confounders.

**Results**: 618 patients met inclusion criteria, but placental pathology available in only 274 cases. The prevalence of funisitis was 23.0% (*n* = 63). The primary outcome identified intraamniotic infection, cesarean in labor, and azithromycin administration as significantly associated with funisitis (Table1).

Patients with funisitis were more likely to require surgical management of SSI (31.7% vs 14.2%), wound washouts at bedside (20.6% vs 12.3%), management in hospital setting (84.1% vs 28.5%), and readmission (53.2% vs 28.5%) (all *p* < 0.05, Table 2). This remained true for all after controlling for confounders (Table 2).

**Conclusion**: Funisitis is prevalent in patients who develop surgical site infections and is associated with more resource‐heavy and complex wound management needs. Funisitis likely represents a more significant pathogen load in the intraamniotic cavity at the time of delivery. This information may help risk stratify patients at risk for cesarean wound complications.

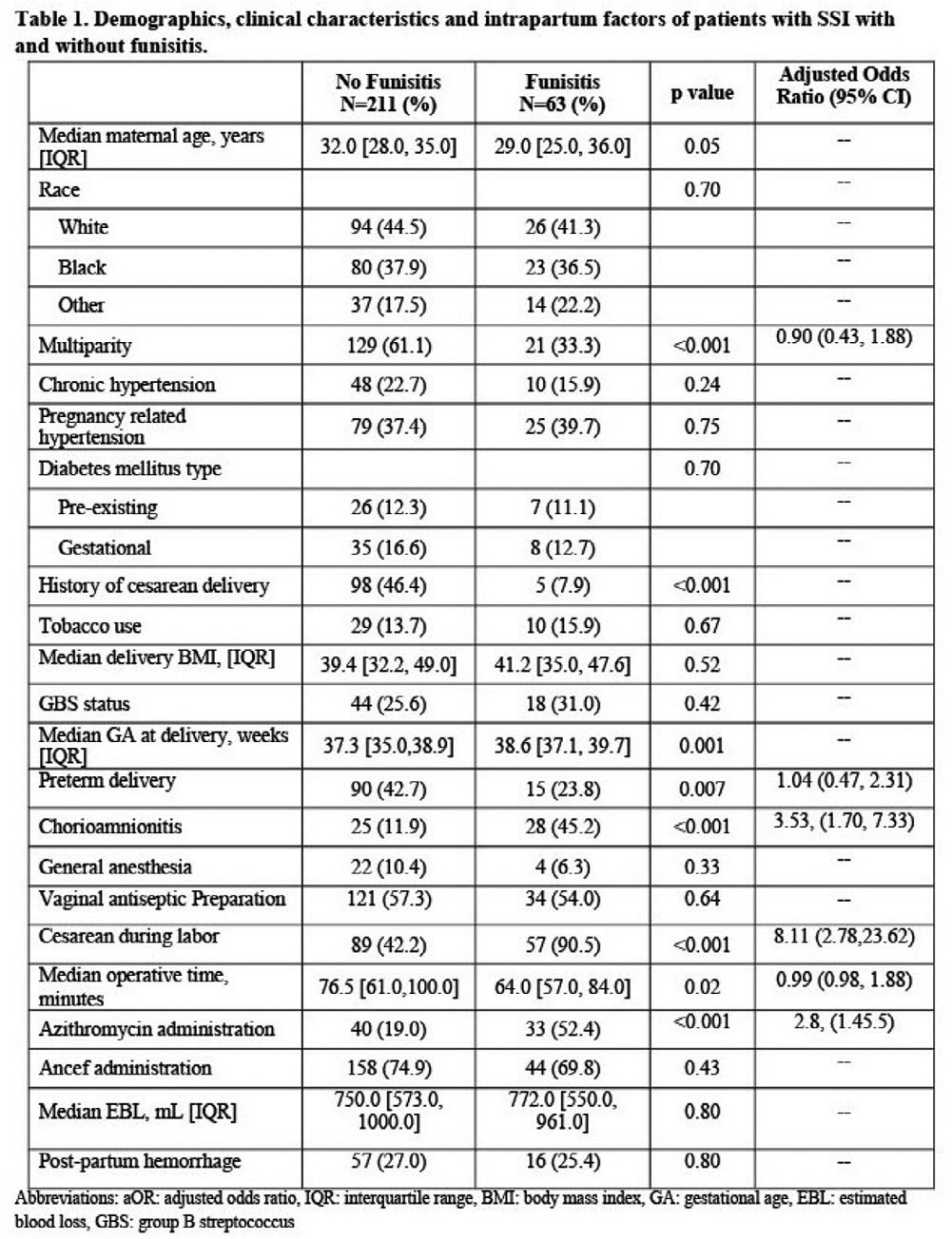

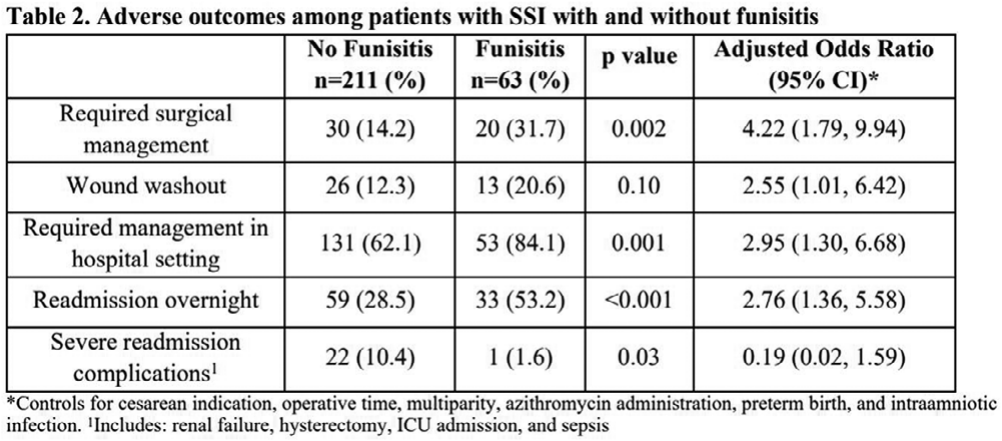

**3:30 PM – 5:00 PM**

## 1159 | **Maternal Adverse Childhood Experiences and Breastfeeding in Low‐Come Pregnant Women: The CRADLE Study**

Lilly Picklesimer^1^; Veronica H. Mize^2^; Jessica H. Britt^2^; Lu Zhang^3^; Moonseong Heo^4^; Amy H. Crockett^2^

^1^The Ohio State University, The Ohio State University, OH; ^2^Prisma Health/University of South Carolina School of Medicine Greenville, Greenville, SC; ^3^Clemson University, clemson, SC; ^4^Clemson University, Clemson, SC

**Objective**: Adverse childhood experiences (ACE) measures traumatic experiences like abuse, neglect and household disfunction. Higher scores are associated with chronic disease, mental health problems and risky health behaviors like substance use disorders. Evidence on the association between ACE and breastfeeding (BF) is inconsistent and largely based on mixed populations. Data from low‐income cohorts are limited. We examined the relationship between ACE and BF initiation and continuation in a well‐defined cohort of low‐income pregnant women.

**Study Design**: Secondary analysis of the Cradle randomized clinical trial evaluating group prenatal care. Participants completed surveys of demographics, health behaviors, measures of ACES, Perceived Stress and Shift and Persist scales. Medical records were abstracted for BF initiation at discharge and at 6 weeks postpartum. Participants were divided between low (0‐3) and high (≥4) ACE scores. T‐tests and chi‐square tested differences between groups. Multivariable logistic regression adjusted for race and maternal age.

**Results**: 2255 participants were included. Most had low ACE scores (1796, 79.7%); 459 (20.4%) had high ACE scores. Participants with high scores were older (26.0 vs 25.3 years), less likely to be Black (28.8% vs 43.6%), more likely to speak English (90.0% vs 82.8%), more likely to drink alcohol during pregnancy (7.3% vs 3.4%) and smoke before (48.2% vs 34.5%) and during pregnancy (27.2% vs 16.4%), *p* < 0.01 for all. Participants with high scores demonstrated higher perceived stress scores (6.29 vs 4.74, *p* < 0.01) and fewer coping skills (3.4 vs 3.5, *p* < 0.01). Participants with high scores demonstrated higher rates of BF initiation (aOR 1.58, 95% CI 1.21‐2.07) and at 6 weeks (aOR 1.49, 95% CI 1.15‐1.95).

**Conclusion**: Higher ACE scores were associated with higher rates of BF despite other negative health behaviors, higher stress and fewer coping skills in a low‐income population. Future studies should explore the contributing factors of success in this group that can be leveraged for improving other health behaviors for patients with high ACE scores.

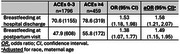

**3:30 PM – 5:00 PM**

## 1160 | **Endothelial Dysfunction and Complement Dysregulation in Growth Restricted Fetuses in the Absence of Preeclampsia**

Lina Youssef^1^; Marta Palomo^2^; Alex Ramos^2^; Maria Borrell^3^; Killian Vellvé^3^; Francesca Crovetto^3^; Eduard Gratacós^4^; Maribel Diaz‐Ricart^2^; Fatima Crispi^5^

^1^BCNatal–Barcelona Center for Maternal‐Fetal and Neonatal Medicine (Hospital Clínic de Barcelona and Hospital Sant Joan de Deu), IDIBAPS, University of Barcelona, Barcelona, Catalonia; ^2^Hematopathology, Centre Diagnòstic Biomèdic (CDB), Hospital Clínic de Barcelona, Institut d'Investigacions Biomèdiques August Pi i Sunyer (IDIBAPS), Barcelona, Catalonia; ^3^BCNatal–Fetal Medicine Research Center (Hospital Clínic de Barcelona and Hospital Sant Joan de Déu), Institut d'Investigacions Biomèdiques August Pi i Sunyer (IDIBAPS), Barcelona, Catalonia; ^4^Hospital Clínic de Barcelona, BARCELONA, Catalonia; ^5^Hospital Clínic de Barcelona, BARCELONA, Catalonia

**Objective**: To assess fetal endothelial biomarkers and complement system in fetal growth restriction (FGR) in the absence of preeclampsia.

**Study Design**: We recruited 47 singleton pregnancies prospectively (15 cases of normotensive FGR and 32 controls). Soluble markers (sTNFR1, sVCAM‐1, sICAM‐1, sVWF, and terminal complement complex (sC5b‐9)) were analyzed in fetal cord blood samples. Moreover, VCAM‐1, ICAM‐1, VWF, VE‐Cadherin, endothelial nitric oxide synthase (eNOS), radical oxygen species (ROS) and C5b‐9 deposits were evaluated on cultured endothelial cells.

**Results**: Increased sTNFR1 and sICAM‐1 concentrations were observed in FGR fetuses compared to controls (2.7 ± 0.5 vs. 1.5 ± 0.3 ng/mL and 143.9 ± 12.4 vs. 114.8 ± 6.8 ng/mL respectively, *p* < 0.05) with similar results of sVCAM‐1, sVWF and sC5b‐9. Endothelial cells exposed to fetal sera showed significantly incremented expression of ICAM‐1, VWF, and VE‐Cadherin with similar expression of VCAM‐1, eNOS and ROS in FGR compared to controls. Significantly less C5b‐9 deposits on endothelial cells were induced by FGR fetal plasma vs. controls (fold change 0.16 ± 0.11 vs. 0.48 ± 0.13, *p* < 0.01).

**Conclusion**: High concentrations of circulating endothelial adhesion molecules and low levels of C5b9 deposits on endothelial cells suggest endothelial damage and complement dysregulation in FGR fetuses. More research is warranted to study the impact of FGR on fetal vascular health and innate immunity.

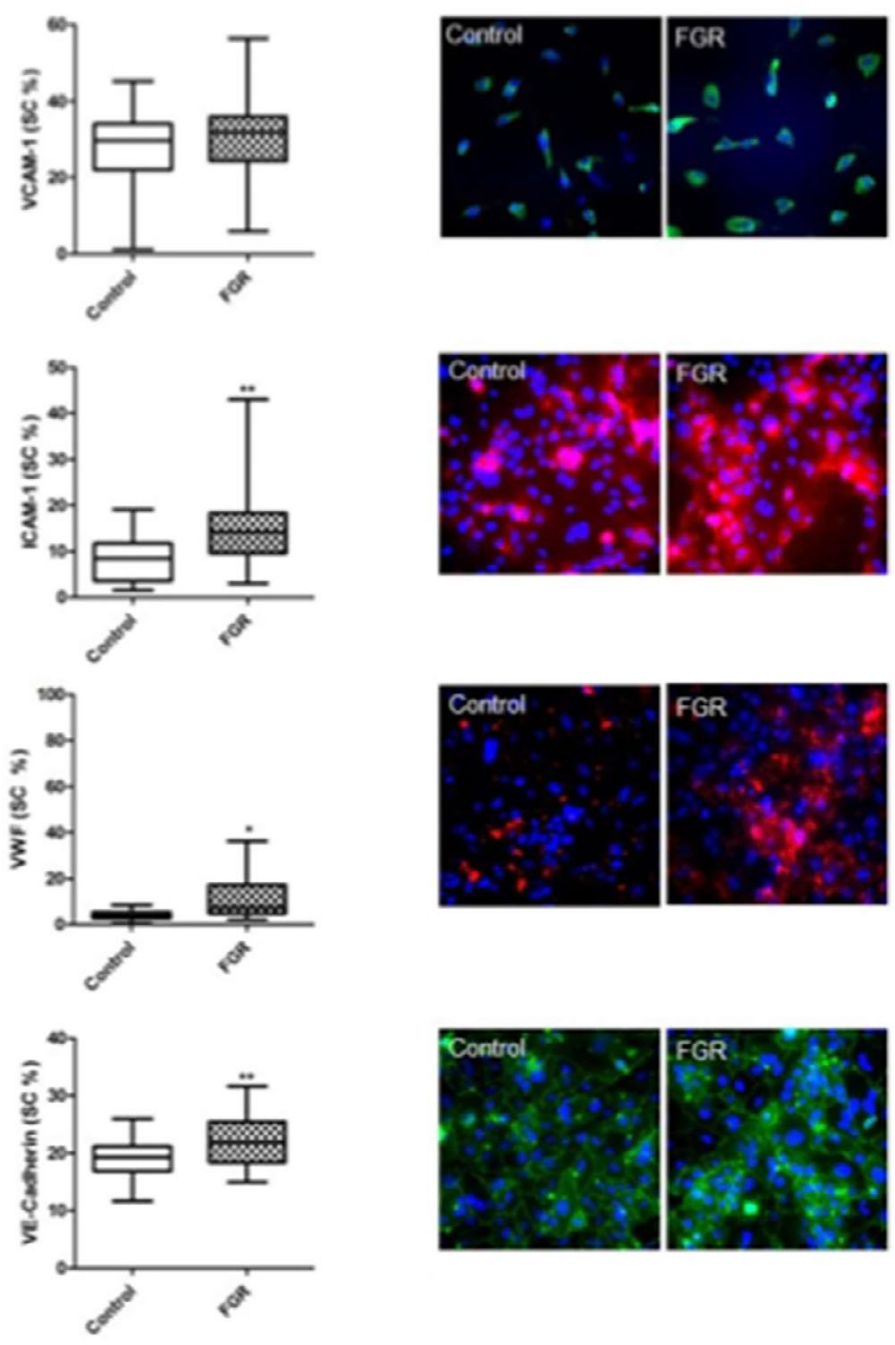

**3:30 PM – 5:00 PM**

## 1161 | **Hyperglycemia and Metformin Differentially Modulate Inflammatory Protein Cargo in Placental Extracellular Vesicles**

Lior Heresco^1^; Itzhak Amoyal^2^; Tal Biron‐Shental^1^; Tali Zitman‐Gal^2^; Michal Kovo^3^

^1^Meir Medical Center, Kfar Saba, HaMerkaz; ^2^Nephrology Laboratory, Meir Medical Center, Kfar Saba, HaMerkaz; ^3^Shamir Medical Center, Israel

**Objective**: Placental extracellular vesicles (EVs) are mediators of maternal‐fetal communication. This study aimed to investigate the impact of hyperglycemia‐a known inflammatory stressor, on placental EV protein content, with and without metformin treatment, using the ex‐vivo human cotyledon perfusion model. We focused on key inflammatory and oxidative stress‐related proteins within EVs: the inflammasome‐associated Caspase‐1, and the redox and inflammatory regulators thioredoxin‐interacting protein (TXNIP), thioredoxin (TRX), suppressor of cytokine signaling 3 (SOCS3), and signal transducer and activator of transcription 3 (STAT3).

**Study Design**: Term placentas from elective cesarean deliveries were perfused for 180 minutes with M‐199 culture medium under three conditions: normoglycemia (control, *n* = 5; glucose 100 mg/dL), hyperglycemia (*n* = 6; glucose 400 mg/dL), and hyperglycemia with metformin (*n* = 5; 1.0 µg/mL). Placental viability was confirmed by stable maternal/fetal flow rates, fetal artery inflow pressure, reservoir pH, and hCG secretion. EVs were isolated from maternal perfusate via differential ultracentrifugation. Western blotting confirmed trophoblastic origin (PLAP‐positive) and minimal exosomal contamination (CD9/CD63‐negative), and was used to quantify expression of the target proteins

**Results**: EVs were successfully isolated from 16 perfusion experiments. Hyperglycemia significantly increased EV expression of Caspase‐1, TRX, and SOCS3 (fold change [FC] vs. control: 1.7, 2.1, and 1.2, respectively; *p* < 0.05). Metformin reduced Caspase‐1 and TRX expression (FC: 0.6 and 0.7, respectively) and increased expression of TXNIP, STAT3, and SOCS3.

**Conclusion**: Hyperglycemia modifies the inflammatory and oxidative stress‐related protein cargo of placental EVs. Metformin modulates this response, exerting both anti‐inflammatory and regulatory effects on specific EV proteins. These findings suggest a complex and selective influence of metformin on placental inflammatory signaling pathways.

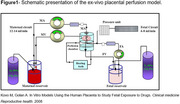

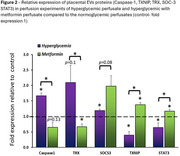

**3:30 PM – 5:00 PM**

## 1162 | **Treatment Outcomes in Pyelonephritis During Pregnancy Following Practice Change From Ampicillin and Gentamicin to Ceftriaxone**

Lisa R. Thiele^1^; Sana Jaleel^2^; Donald D. McIntire^2^; Emily H. Adhikari^2^; Elaine L. Duryea^2^; Catherine Y. Spong^2^

^1^UT Southwestern, Dallas, TX; ^2^University of Texas Southwestern Medical Center, Dallas, TX

**Objective**: To evaluate the impact of a practice change from a combined regimen of ampicillin and gentamicin (amp/gent) to single agent ceftriaxone for pyelonephritis in pregnancy.

**Study Design**: This retrospective cohort study examined outcomes for pregnant patients treated before and after a change in treatment protocol for pyelonephritis at our institution from amp/gent to ceftriaxone as first‐line therapy. Treatment is standardized using electronic order sets and written protocols. Data including demographics, laboratory data, medical comorbidities, and characteristics of pyelonephritis treatment and admissions were examined.

**Results**: Of 153 treated for antepartum pyelonephritis, 93 were treated during the Amp/Gent protocol (1/1/2022‐12/31/2022) and 60 during the Ceftriaxone protocol (12/5/2024‐5/5/2025). Patients in the ceftriaxone cohort had more pronounced leukocytosis (15.1 ± 4.0 vs.13.7 ± 4.3, *p* = 0.04) and higher maximum heart rate (128.6 ± 17.4 vs. 120.2 ± 17.9, *p* = 0.005), Table. There was no significant difference in the duration of IV antibiotic therapy or length of stay between cohorts. There was a significant decrease in the number of doses given (8.9 vs 4.5, *p* = < 0.001) in the ceftriaxone cohort. E. Coli was the predominant pathogen, followed by Klebsiella and ESBL. Pathogenic organisms in both cohorts were more susceptible to Ceftriaxone (88‐100%) than Ampicillin (43%‐50%) or Gentamicin (50‐100%), Figure.

**Conclusion**: Transitioning from dual (amp/gent) to single agent (ceftriaxone) therapy as primary regimen for treatment of pyelonephritis in pregnancy was associated with fewer doses of antibiotics without effect on length of stay or duration of antibiotic therapy. The difference in severity of illness, as measured by leukocytosis and max temperature, limits interpretation of the effect of the protocol change.

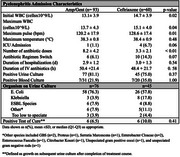

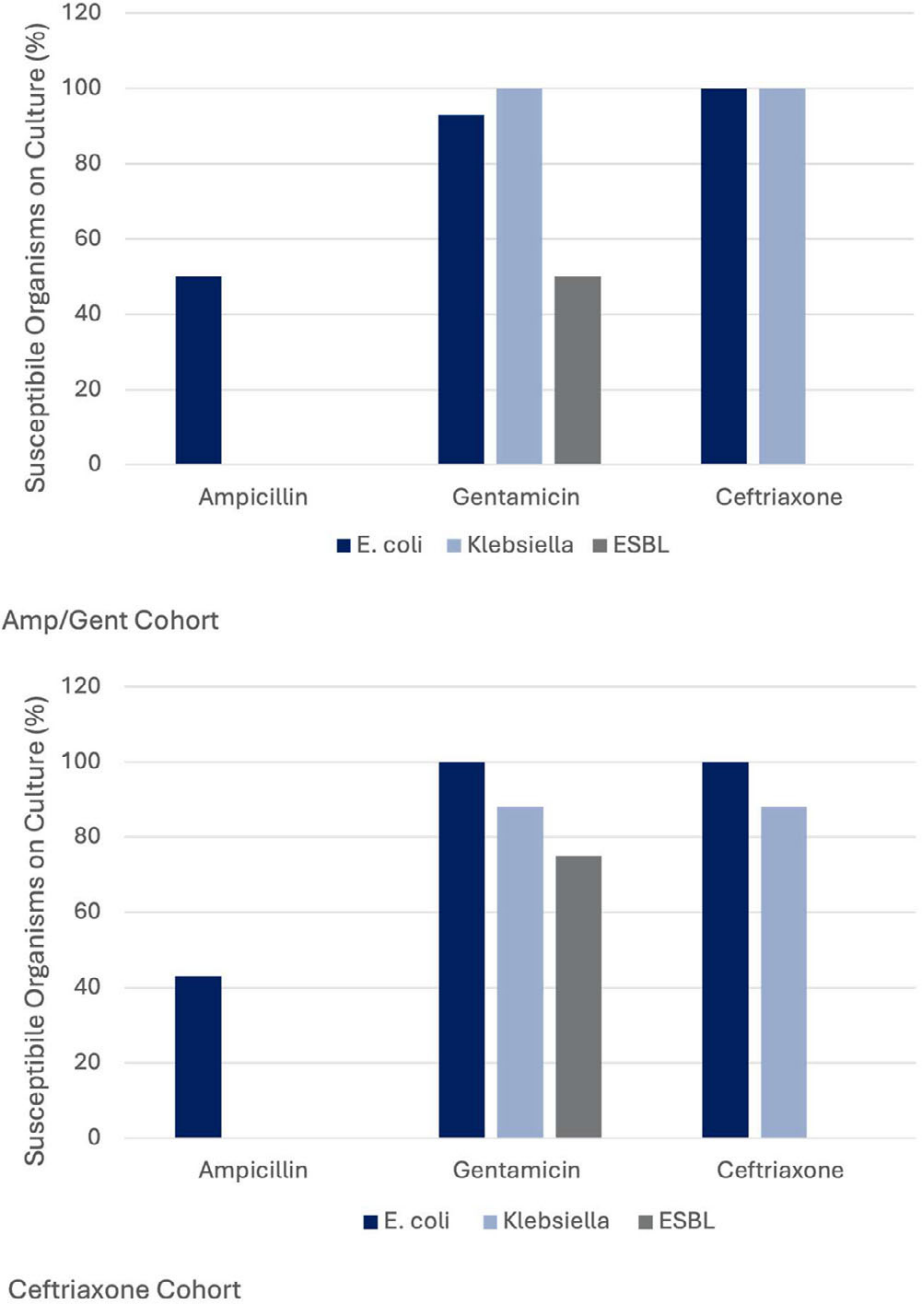

**3:30 PM – 5:00 PM**

## 1163 | **Reducing Postpartum Readmissions: Development of a Risk Model Based on Maternal and Peripartum Factors**

Lital Shaham^1^; Aya Kook^1^; Abraham Tsur^1^; Liat Toderis^2^; Ronen Loebstein^2^; Michal J. Simchen^1^

^1^Department of Obstetrics and Gynecology, Sheba Medical Center, Sheba Medical Center, HaMerkaz; ^2^ADAMS Center, Sheba Medical Center, Sheba Medical Center, HaMerkaz

**Objective**: To identify maternal and peripartum risk factors for postpartum hospital readmission and to develop a clinically applicable risk score for early identification of high‐risk patients.

**Study Design**: This retrospective cohort study included 132,850 women who delivered at Sheba Medical Center between 2011 and 2023. Data were extracted from a perinatal database. Readmission was defined as re‐hospitalization within 30 days postpartum. Maternal, obstetric, and neonatal characteristics were compared between patients readmitted and those not. Independent predictors were identified using multivariate logistic regression, and a risk score was developed based on regression coefficients.

**Results**: Among 838 women (0.63%) readmitted postpartum, patients were older, had higher BMI, and more comorbidities such as chronic hypertension (7.2% vs. 1.1%), renal disease (4.3% vs. 1.3%), and pregestational diabetes (3.7% vs. 1.2%) (all *p* < 0.001). Cesarean delivery (78.0% vs. 29.1%), preeclampsia (17.4% vs. 4.6%), and preterm birth (32.1% vs. 11.4%) were significantly more common. Infections were the leading cause of readmission (51.2%), mainly surgical site infections and endometritis, followed by preeclampsia (15%), PPH (6%), and VTE (2.5%).

Subgroup analyses linked infection‐related readmissions to postpartum fever. Preeclampsia‐related cases were more common with chronic hypertension, obesity, and older maternal age. VTE‐related readmissions were associated with placental abruption and fetal growth restriction.

Six independent predictors were identified: chronic hypertension, preeclampsia, preterm birth, cesarean/instrumental delivery, postpartum fever, LMWH use, and blood transfusion. The risk model stratified patients into low, moderate, and high‐risk groups, with readmission probabilities from 0.16% to 69.6% (Figure 1).

**Conclusion**: Postpartum readmission is associated with comorbidities and peripartum complications. A simple risk score allows early identification of high‐risk women and may facilitate aquality improvement intervention  .

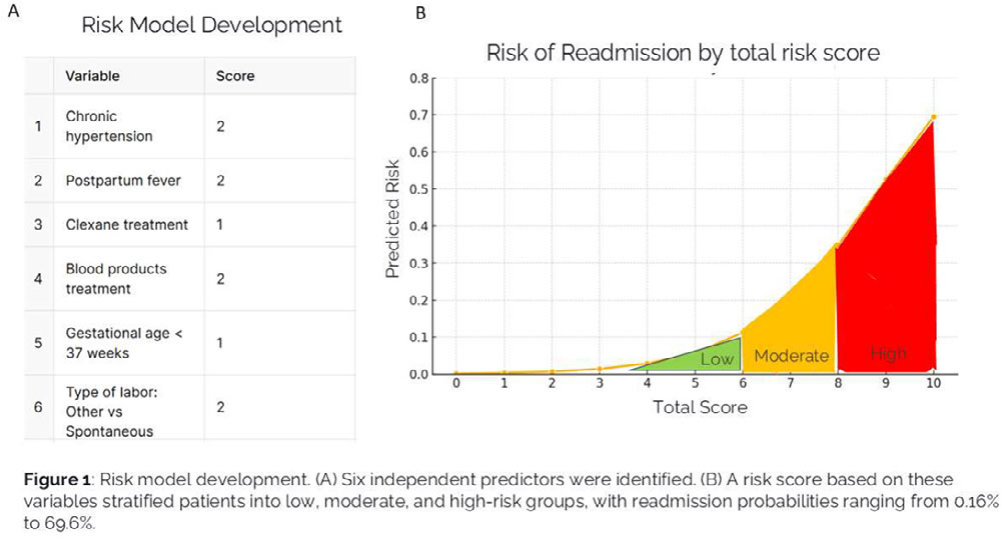

**3:30 PM – 5:00 PM**

## 1164 | **Comprehensive Single‐Cell Profiling of the Maternal‐Fetal Interface in Type 1 Diabetes**

Paola A. Lopez‐Zapana^1^; Rachelly Normand^2^; Daehee Han^3^; Olyvia J. Jasset^1^; Laura Ibanez‐Pintor^1^; Douglas A. Lauffenburger^4^; Alexandra‐Chloé Villani^5^; Andrea G. Edlow^6^; Lydia L. Shook^7^

^1^Vincent Center for Reproductive Biology, Massachusetts General Hospital, Boston, MA; ^2^Krantz Family Center for Cancer Research, Massachusetts General Hospital; Harvard Medical School, Boston, MA; ^3^Vincent Center for Reproductive Biology, Massachusetts General Hospital; Department of Obstetrics and Gynecology, MassGeneral Brigham; Division of Maternal‐Fetal Medicine; Harvard Medical School, Boston, MA; ^4^Department of Biological Engineering, Massachusetts Institute of Technology, Cambridge, MA; ^5^Center for Immunology and Inflammatory Diseases, Krantz Family Center for Cancer Research, Massachusetts General Hospital, Boston, MA; ^6^Department of Obstetrics and Gynecology, MassGeneral Brigham; Division of Maternal‐Fetal Medicine; Harvard Medical School, Boston, MA; ^7^Department of Obstetrics and Gynecology, MassGeneral Brigham; Division of Maternal‐Fetal Medicine; Harvard Medical School, Massachusetts General Hospital/Boston, MA

**Objective**: Individuals with type 1 diabetes (T1D), an autoimmune disorder with insulin deficiency and metabolic dysregulation, have increased risk of placental dysfunction and adverse pregnancy outcomes. The molecular mechanisms underlying pregnancy morbidity in T1D are poorly understood. We examined placental gene programs at the single‐cell level in T1D pregnancies.

**Study Design**: We collected 19 placental samples from T1D pregnancies (*N* = 10) and healthy controls (*N* = 9), balanced for fetal sex. We profiled maternal and fetal cells using 10x Genomics single‐cell RNA sequencing. Genetic demultiplexing determined maternal/fetal cell origin. Differentially expressed genes (DEGs) between T1D and controls were identified across cell subclusters (threshold: fold change >1.5, FDR < 0.1). Pathway analysis (Ingenuity) was performed on subclusters with ≥50 DEGs.

**Results**: Of 114 trophoblast, immune, and stromal subpopulations identified, 35 were dysregulated by T1D (Figure 1). Villous cytotrophoblasts (VCTs), maternal CD14+ monocytes, fetal S100A8+ macrophages (Hofbauer subset), and fetal endothelial cells were most impacted. Dysregulated canonical pathways included upregulated oxidative phosphorylation and mitochondrial function in VCTs, altered activation patterns in macrophages, and dysregulated IL‐4/13 and JAK‐mediated cytokine signaling in fetal endothelial cells, indicating fetal inflammation (Figure 2A). Multiple disease functions were upregulated in fetal endothelial cell subtypes: cell survival, leukocyte adhesion/migration, and angiogenesis (Figure 2B). Glucose metabolism disorder pathways were upregulated in maternal and fetal macrophage subtypes.

**Conclusion**: Examination of the maternal‐fetal interface at the single‐cell level revealed novel immune and metabolic gene pathways impacted by T1D. These findings suggest an impact of maternal T1D across the maternal‐fetal interface that may have implications for offspring cardiometabolic programming.

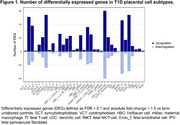

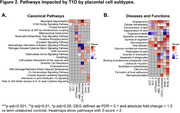

**3:30 PM – 5:00 PM**

## 1165 | **Maternal Exposure to Heavy Metals and Risk of Recurrent Preterm Birth: A Prospective Cohort Study**

Maayan Hagbi Bal^1^; Shimrit Yaniv Salem^2^; Eyal Sheiner^3^; Israel Yoles^4^; Noa Dina Israel‐Tov^5^; Iris Shoam^5^; Itai Hazan^6^; Noam Tomasis Damri^6^; Doron Bergman^5^; Ron Rosenbaum^5^; Eyal Haimov^6^; Ruslan Sergienko^7^; Ilana Shoham Vardi^6^; Tamar Wainstock^4^

^1^Icahn School of Medicine at Mount Sinai, Beer sheva, HaDarom; ^2^Faculty of Health Sciences, Joyce & Irving Goldman Medical School at Ben‐Gurion University, Beer sheva, HaDarom; ^3^Soroka University Medical Center, Faculty of Health Sciences, Ben‐Gurion University of the Negev, beer sheva, HaDarom; ^4^Ben‐Gurion University of the Negev, Beer sheva, HaDarom; ^5^Ben‐Gurion University of the Negev, Ben Gurion, HaDarom; ^6^Ben‐Gurion University of the Negev, Beer Sheva, HaDarom; ^7^Ben‐Gurion University of the Negev, ben gurion, HaDarom

**Objective**: With increasing industrialization and urbanization, concerns have arisen about the impact of environmental exposures, including heavy metals, on pregnency outcomes. Some studies suggest associations between elevated metal levels and adverse outcomes such as preterm birth (PTB), but evidence remains limited and inconsistent, especially regarding recurrent PTB. Given that prior PTB is a known risk factor for subsequent PTB, our study aimed to assess whether maternal exposure to heavy metals, especially Selenium (Se), is associated with recurrent PTB to identify potentially modifiable environmental risk factors.

**Study Design**: We conducted a prospective cohort study at a tertiary center and affiliated clinics, enrolling pregnant women with prior spontaneous singleton PTB (IRB#0390‐20‐SOR). Maternal blood was collected at two time points to assess concentrations of 10 trace elements: chromium (Cr), cobalt (Co), nickel (Ni), copper (Cu), zinc (Zn), arsenic (As), Se, cadmium (Cd), mercury (Hg), and lead (Pb). Participants completed sociodemographic questionnaires. Multivariable models assessed associations between metal levels and recurrent PTB, adjusting for clinical and socioeconomic factors.

**Results**: Among 267 women enrolled, 247 (92.5%) completed follow‐up, with 22.3% experiencing recurrent PTB. Women with Se above the 75th percentile had a 2.4‐fold increased risk of recurrence (OR = 2.37, 95% CI:1.26–4.46, *p* = 0.01). Multivariable models confirmed the association (adj.OR = 1.016 per unit, 95% CI:1.01–1.03, *p* = 0.03; adj.OR = 2.68 for >75th percentile, 95% CI:1.38–3.91, *p* = 0.004). Linear mixed‐effects models showed higher Se in women with recurrence (mean difference = 6.29µg/L, 95% CI:1.37–11.20, *p* = 0.013). First‐trimester Se was strongly associated with recurrence (adj.OR = 3.245, 95% CI:1.343–7.840, *p* = 0.009); no associations were found in later trimesters.

**Conclusion**: Higher maternal Se in early pregnancy is linked to recurrent PTB, with no associations seen for other metals. Findings highlight a possible gestational timing effect and suggest further research is needed before Se supplementation in pregnancy is recommended.

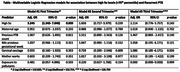

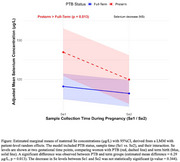

**3:30 PM – 5:00 PM**

## 1166 | **Hypoglycemia on Gestational Diabetes Diagnostic Testing and Obstetric Outcomes**

Maayan Ormianer^1^; Sharon Alpern^2^; Hadar Rosen^3^; Doron Kabiri^4^; Genyah Aharon‐Hananel^1^; Lauren H. Yaeger^5^; Joshua Isaac Rosenbloom^1^

^1^Hadassah Medical Center and Hebrew University of Jerusalem, Jerusalem, Israel, Jerusalem, Yerushalayim; ^2^Hadassah Medical Center, Ein Kerem Campus, Jerusalem, Israel, Jerusalem, Yerushalayim; ^3^Hadassah Medical Center and Hebrew University of Jerusalem, Jerusalem, Israel, Hadassah Medical Center Ein Kerem Campus, Jerusalem, Israel, Yerushalayim; ^4^Hadassah Medical Center and Hebrew University of Jerusalem, Jerusalem, Israel, Hadassah Medical Center and Hebrew University of Jerusalem, Jerusalem, Israel, Yerushalayim; ^5^Washington University School of Medicine, Washington, PA

**Objective**: Although high values on glucose screening tests are diagnostic of gestational diabetes, there is controversy in the literature as to the meaning of low values on the tests. Therefore, we performed a systematic review and meta‐analysis to investigate whether post glucose test hypoglycemia is related to maternal and neonatal adverse outcomes.

**Study Design**: A comprehensive search was performed by a medical librarian for literature in any language through March 2025 that reported perinatal outcomes in pregnant patients with low values on gestational diabetes screening or diagnostic tests.  All retrieved titles and abstracts were independently reviewed by two researchers and data were extracted.  Random effects meta‐analysis was used where appropriate.

**Results**: The search resulted in 2596 unique titles of which 63 were deemed potentially eligible and 20 were ultimately included.  Study sample sizes ranged from 200‐128,999 participants and most studies were retrospective.  There was heterogeneity in the types of tests used (50g, 75, and 100g glucose tests), definitions of hypoglycemia, and definitions of outcomes.  However, overall, hypoglycemia on a gestational diabetes test was associated with an increased risk of growth restriction (OR 1.46, 95% CI 1.27, 1.68) and NICU admission (OR 1.21, 95% CI 1.00, 1.47), and a lower risk of macrosomia (OR 0.68, 95% CI 0.55, 0.85). When stratifying by type of glucose screen (50g, 75g, or 100g,) results stayed consistent.

**Conclusion**: Hypoglycemia on gestational diabetes screening may be associated with a number of adverse neonatal outcomes. In particular, it is associated with IUGR, suggesting that hypoglycemia may be a marker of placental dysfunction.  Clinicians should consider monitoring patients with hypoglycemia on gestational diabetes testing with serial growth scans. However, due to heterogeneity in study design and variable definitions, a large prospective study is needed to further elucidate the significance of this common clinical conundrum.

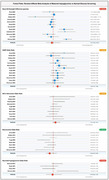

**3:30 PM – 5:00 PM**

## 1167 | **Risk Factors of Extreme Preterm Birth After Physical Exam Indicated Cerclage**

Mackenzi R. McHugh^1^; Grace Spencer^2^; Tetsuya Kawakita^3^

^1^EVMS Department of Obstetrics and Gynecology, Macon & Joan Brock Virginia Health Sciences at Old Dominion University, Eastern Virginia Medical School/Norfolk, VA; ^2^Eastern Virginia Medical School at Old Dominion University, EVMS OBGYN, Virginia Health Sciences at Old Dominion University, VA; ^3^Eastern Virginia Medical School Department of Obstetrics and Gynecology, Macon & Joan Brock Virginia Health Sciences at Old Dominion University, Norfolk, VA

**Objective**: To identify risk factors associated with extreme preterm birth (<28 weeks) in patients undergoing physical exam‐indicated cerclage.

**Study Design**: This was a retrospective cohort study conducted among patients who underwent physical exam‐indicated cerclage at an academic institution from 2014 to 2022. The primary outcome was delivery timing (<28 weeks’ gestation or ≥28 weeks’ gestation). We examined risk factors including maternal demographics, gestational age at cerclage, and the degree of cervical dilation before cerclage. Multivariable logistic regression was used to calculate adjusted odds ratios (aOR) with 95% confidence intervals (95% CI). We also examined the association between the time from cerclage placement to delivery and the degree of cervical dilation using the Kaplan‐Meier curve.

**Results**: A total of 117 patients were included, with 31.6% (*n* = 37) delivering <28 weeks and 68.4% (*n* = 80) delivering ≥28 weeks. There were no statistically significant differences between groups in maternal demographics, genitourinary infection, and gestational age at cerclage placement (Table 1). Cervical dilation at the time of cerclage was significantly greater among those who delivered before 28 weeks (median 2.0 cm vs. 1.0 cm, *p* = 0.006). Compared to cervical dilation 1 cm, cervical dilation 2, 3, and 4‐5 cm were associated with increased odds of delivery <28 weeks (aOR 1.51 [95% CI 0.50–4.60] for 2 cm; aOR 8.82 [1.91–40.79] for 3 cm; and aOR 12.17 [2.21–67.01] for 4–5 cm). In time‐to‐delivery analyses, more than half of patients with 4–5 cm dilation delivered within 2 weeks of cerclage placement (Figure 1).

**Conclusion**: Greater cervical dilation at physical exam–indicated cerclage is independently associated with markedly increased odds of delivery before 28 weeks. Patients presenting with 4–5 cm dilation have the shortest latency, with most delivering within 2 weeks despite cerclage. Cervical dilation should be emphasized in prognostication, risk stratification, and counseling at the time of cerclage placement.

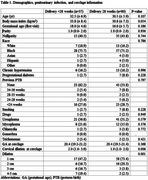

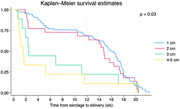

**3:30 PM – 5:00 PM**

## 1168 | **Venture Capital‐Backed Pregnancy Health Startups and the Maternal Health Crisis**

Madeline F. Perry^1^; Kristan A. Scott^2^; Diana Montoya‐Williams^2^; Sindhu K. Srinivas^3^; Scott A. Lorch^2^; David Grande^4^

^1^Hospital of the University of Pennsylvania, Philadelphia, PA; ^2^Children's Hospital of Philadelphia, Philadelphia, PA; ^3^Department of Obstetrics and Gynecology, Perelman School of Medicine, Maternal and Child Health Research Center, Philadelphia, PA; ^4^Perelman School of Medicine, Philadelphia, PA

**Objective**: The US has significant disparities in maternal morbidity and mortality. In response to this crisis, there has been public and private investment in maternal health. While venture capital (VC) investing in pregnancy health can drive innovation, limited research exists. We aim to describe VC‐backed pregnancy health startups and assess how they address recognized problems in maternal health.

**Study Design**: This was a content analysis of VC‐backed pregnancy health startups. Included companies were founded between 1/1/2014‐12/31/2022, headquartered in the US and directly related to pregnancy health. We used financial databases Pitchbook and Crunchbase to identify startups and obtain descriptive characteristics. Dual‐coder content analysis was performed using company websites to assess business model, mention of health equity and maternal mortality, acceptance of insurance and/or Medicaid, and healthcare category. Kappa for content analysis ranged from 0.67‐0.94 (median 0.82), demonstrating strong agreement.

**Results**: 172 companies were identified. During the study period, $1.8 billion was raised for VC‐backed pregnancy health startups. Annually, there was a 63% increase in startups founded, with the greatest annual increase among virtual and hybrid wrap around care companies (Figure). We identified 8 healthcare categories for pregnancy health startups (Table). The content analysis further demonstrated: 1) few companies had any focus on health equity or maternal mortality, 2) 65% of companies advertised directly to consumers, 3) 42% of companies providing goods/services typically covered by insurance were covered by Medicaid. Virtual/hybrid wrap around care was the most common type of company (35%), but had below average total capital raised (Table).

**Conclusion**: VC backed startups are filling gaps in pregnancy care delivery–rather than pharmaceuticals and devices as is most common in VC. Startups have the potential to address the maternal health crisis and contribute needed innovation, but few focus on low‐income populations or health equity. Importantly, rigorous evaluation of startup outcomes is needed.

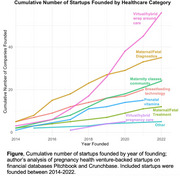

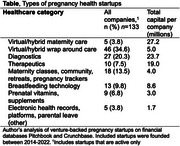

**3:30 PM – 5:00 PM**

## 1169 | **Cumulative Impact of Multiple Severe Features of Preeclampsia and Maternal Morbidity**

Madison Calvert^1^; Hannaneh Mirmozaffari^2^; Maya Patel^2^; Jaye Boissiere^3^; Sally Kuehn^3^; Brooke Schroeder^3^; Matt Fuller^3^; Marie‐Louise Meng^3^; Johanna Quist‐Nelson^2^; Kim Boggess^4^

^1^University of North Carolina School of Medicine, Chapel Hill, NC; ^2^University of North Carolina, Chapel Hill, Chapel Hill, NC; ^3^Duke University School of Medicine, Durham, NC; ^4^University of North Carolina at Chapel Hill, Chapel Hill, NC

**Objective**: PEC with severe features (PEC‐SF) is defined by severe hypertension and/or signs of end‐organ dysfunction and increases risk of cardiovascular disease. The specific acute risks for patients with multiple severe features remain unknown. We hypothesize that compared to having 2 or fewer severe criteria, >2 criteria is associated with higher odds of maternal postpartum morbidity.

**Study Design**: This retrospective cohort study included patients with antepartum PEC‐SF defined by ACOG who delivered at two academic medical centers from 2016‐17. The exposure was number of severe features present at delivery admission. The primary outcome was severe maternal morbidity (SMM) defined by the CDC at delivery hospitalization. A multivariable logistic regression model generated odds ratios (ORs) and 95% confidence intervals (CI) for the associations of number of severe features (<2, 3 or 4‐6) with SMM, adjusting for chronic hypertension, diabetes, obesity (BMI >30) and renal disease (reference group: patients with < 2 features).

**Results**: In the cohort, 774 were diagnosed with PEC‐SF. 685 (88.5%) patients had < 2 severe features, 65 (8.4%) had 3 and 24 (3.1%) had 4‐6. Having 3 severe features was associated with higher risk of transfusion‐related SMM (OR 1.17, 95% CI 1.1‐1.25) and non‐transfusion SMM (OR 1.12, 95% CI 1.05‐1.19) compared to < 2 severe features. 4‐6 severe features conferred greater risk of transfusion‐related (OR 1.55, 95% CI 1.38‐1.73) and non‐transfusion SMM (OR 1.57, 95% CI 1.41‐1.73) (Figure 1). Blood product transfusion at time of delivery was the most frequent SMM observed across all groups. Most common non‐transfusion SMM were acute renal failure, DIC, eclampsia, pulmonary edema/heart failure, and need for ventilation (Figure 2).

**Conclusion**: In patients with PEC‐SF, > 2 severe criteria confer incremental SMM risk at delivery, emphasizing the importance of identifying all severe features at diagnosis. Patients with > 2 severe criteria should deliver at a higher level of maternal care prepared to respond to SMM events.

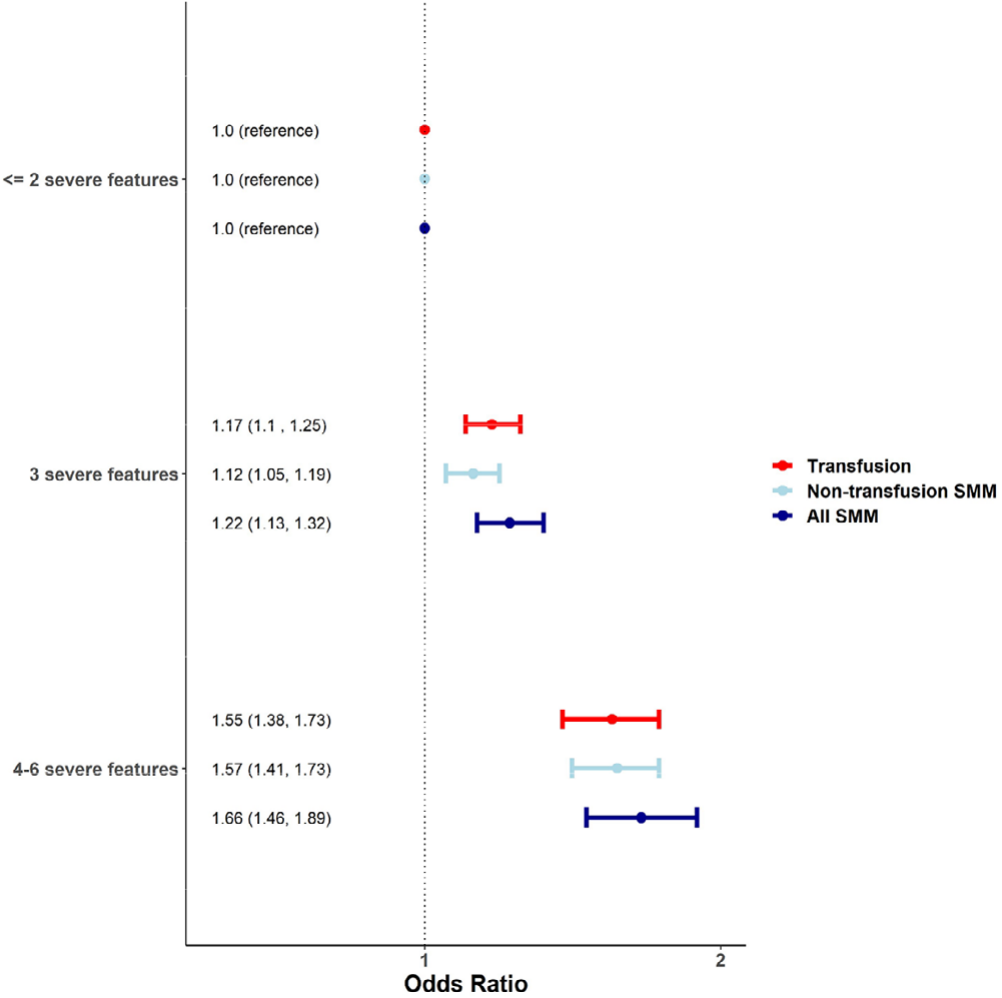

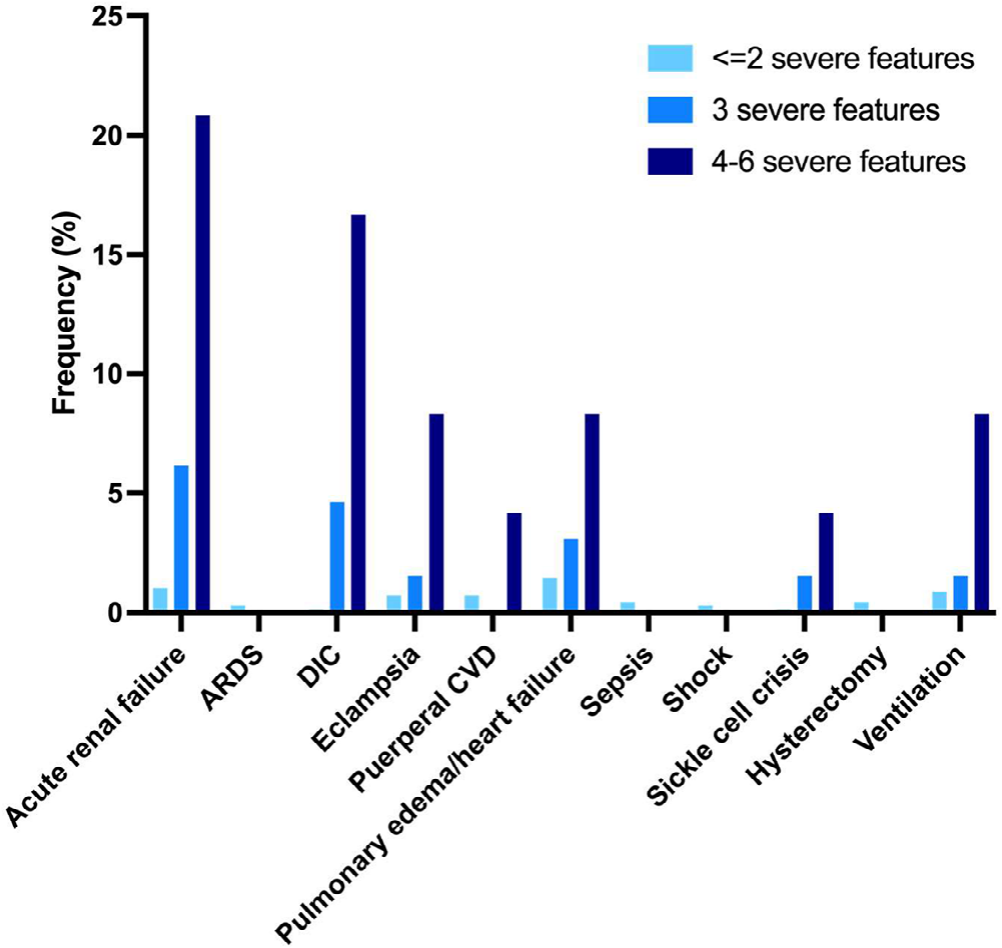

**3:30 PM – 5:00 PM**

## 1170 | **Comparing Continuous Glucose Monitoring and Blood Glucose Monitoring Among Pregnant Individuals With Type 2 Diabetes**

Manasa G. Rao^1^; Sbaa Syeda^2^; Chloe Porigow^3^; Emily Hansman^4^; Shai Bejerano^5^; Hooman A. Azad^6^; Uma M. Reddy^5^; Jacqueline Lonier^4^; Noelia Zork^5^

^1^Columbia University, New York, NY; ^2^Johns Hopkins, Baltimore, MD; ^3^Albert Einstein College of Medicine, New York, NY; ^4^Columbia University Medical Center, New York, NY; ^5^Columbia University Irving Medical Center, New York, NY; ^6^Columbia University, Columbia University, NY

**Objective**: While using continuous glucose monitoring (CGM) in pregnancy may improve outcomes in those with Type 1 diabetes, the benefits of CGM in pregnant patients with Type 2 diabetes (T2DM) is lacking. The objective of our study was to assess maternal and neonatal outcomes with use of CGM versus blood glucose monitoring (BGM) among pregnant individuals with T2DM.

**Study Design**: This is a retrospective cohort study (2017‐2023) of pregnant individuals with T2DM receiving maternal‐fetal medicine (MFM) and endocrinology care at a single institution and managed with either CGM or BGM. The primary outcome was the percentage achieving HbA1c <6.0% by delivery. Secondary outcomes included maternal and neonatal measures related to glycemic control. Multivariable logistic regression accounting for prenatal care provider team (MFM with or without endocrine co‐management), body mass index, gestational weight gain, and comorbid conditions was conducted.

**Results**: Of 79 patients, 28 (35%) used CGM and 51 (65%) used BGM during pregnancy. A significantly greater number of CGM users were comanaged by MFM and endocrine. Otherwise, baseline characteristics did not vary significantly between groups. In the adjusted analysis, the percent achieving HbA1c <6.0% was not significantly different between groups. In addition, there were no significant differences in maternal outcomes including development of hypertensive disorders, diabetic ketoacidosis, polyhydramnios, preterm labor, chorioamnionitis, wound complications, or hospital readmission (Table). Neonatal outcomes, including gestational age at delivery, birth weight, congenital anomalies, hypoglycemia, respiratory distress syndrome, transient tachypnea, hyperbilirubinemia, sepsis, demise, and NICU admission were similar between groups.

**Conclusion**: Use of CGM was not associated with improvement in glycemic control or in maternal and neonatal outcomes compared to use of BGM in this small cohort of pregnant individuals with T2DM. Larger studies are needed to assess if CGM use is beneficial in this population.

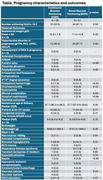

**3:30 PM – 5:00 PM**

## 1171 | **Recent Trends in Prenatal Genetic Screening for Aneuploidy at a Large Academic Center**

Marcia Des Jardin^1^; Jordan Garcia^2^; Lianhua Jin^1^; Dhong‐Jin Kim^3^; Lynda G. Ugwu^1^; Angela R. Seasely^4^; Akila Subramaniam^1^

^1^Center for Research in Women's Health, University of Alabama at Birmingham, Birmingham, AL; ^2^Department of Obstetrics and Gynecology, University of Alabama at Birmingham, University of Alabama at Birmingham, AL; ^3^Center for Research in Women's Health, University of Alabama at Birmingham, University of Alabama at Birmingham, AL; ^4^Fort Sanders Perinatal Center, Fort Sanders Perinatal Center, TN

**Objective**: Genetic screening for aneuploidy is recommended for all patients, but ACOG does not recommend one standard approach.  Given rapid uptake of non‐invasive prenatal testing (NIPT), we sought to evaluate recent rates of specific aneuploidy screening test uptake.

**Study Design**: We performed a retrospective cohort study of all patients who received prenatal care at a single center 2018‐2023. Patient demographics, location of clinical care, and aneuploidy screening methods (NIPT versus quad screen) were ascertained. NIPT was a send‐out lab, while quad screen was done in‐house. Of note, combined/first trimester serum screening was discontinued by our in‐house lab June 2023, and thus not included for analysis. The primary outcome was NIPT uptake; secondary outcome was uptake of quad screen. Outcomes were compared by each calendar year. Secondary analysis evaluated patient characteristics of those who did and did not receive NIPT (odds ratio [OR]s with 95% confidence intervals [CI]) with temporal variations in NIPT uptake evaluated using the Breslow‐Day test (BDT).

**Results**: Of 30,570 pregnancies during the study period, 20,165 met study criteria. There was a significant increase in the rate of NIPT uptake from 8.6% to 32.2% with reciprocal decrease in quad testing (*p* < 0.001, Figure 1). Patients who received NIPT were more likely to have government insurance (OR 2.72 [95% CI 2.51, 2.95]), be ≥35 years old (OR 2.18 [2.01, 2.37]), receive care at the publicly funded high‐risk clinics (OR 3.00 [2.78, 3.22]), be multiparous (OR 1.36 [1.27, 1.47], and self‐identify as Black race (OR 2.15 [2.00, 2.30]). Over time, the odds of NIPT significantly differed amongst all these characteristics (BDT *p* < 0.05) except parity (*p* = 0.07). NIPT receipt continued to be higher amongst those with government insurance and of Black race over the study period, while NIPT uptake became similar based on age (Figure 2).

**Conclusion**: We demonstrate decreasing traditional serum screening and increasing NIPT uptake for aneuploidy screening. Of note, race, payor type, and clinical location are highly correlated with NIPT receipt over time.

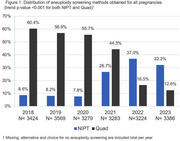

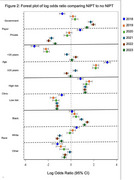

**3:30 PM – 5:00 PM**

## 1172 | **Obstetric Outcomes Following Cerclage Placement in Multiparous Patients With and Without a Prior Preterm Birth**

Marja McLaughlin^1^; Habibatu Ighile^2^; Suzanne O'Nan^2^; Sarah A. Wernimont^2^; Katelyn M. Tessier^3^; Martina S. Burn^2^

^1^University of Minnesota, Mendota Heights, MN; ^2^University of Minnesota, Minneapolis, MN; ^3^University of Minnesota Masonic Cancer Center, University of Minnesota, Minneapolis MN, MN

**Objective**: Obstetric history is a known risk factor for preterm birth (PTB). Our aim was to investigate whether a previous term delivery decreases the odds of PTB among multiparous patients undergoing cerclage placement.

**Study Design**: This was a retrospective cohort study of multiparous patients with a singleton gestation who underwent cerclage placement between 16‐24 weeks gestation due to cervical dilation (≥1cm) or significant cervical shortening (<10mm) between 2011‐2024 in an academic health system. Individuals were categorized by obstetric history based on prior term birth or any prior PTB. The primary outcome was spontaneous PTB <32 weeks. Secondary outcomes included gestational age at delivery, living status at birth, neonatal intensive care unit (NICU) admission, and NICU length of stay. Bivariate analyses and multivariable logistic regressions were performed.

**Results**: Among 67 identified individuals, 39 (58%) had no history of PTB and 28 (42%) had a history of PTB. Those with a prior term birth were more likely to have a shorter cervix [median (IQR) 6.2mm (0, 10.0) vs. 17.0mm (8.0, 20.6), *p* < 0.001], increased cervical dilation [median (IQR) 1.0cm (1.0, 2.2) vs. 1.0cm (1.0, 1.0), *p* = 0.009] and prolapsing membranes (44% vs 4%, *p* < 0.001) at the time of cerclage placement (Table 1). There was no difference in rates of PTB <32 weeks among patients without a PTB history compared to patients with a PTB history (21% vs. 14%, *p* = 0.512; OR 1.55, 95% CI 0.43‐6.37) (Table 2). Even after adjusting for baseline differences in cervical length at time of cerclage placement, there remained no difference in odds of PTB <32 weeks between patients without and with a prior PTB (aOR 0.88, 95% CI 0.2‐4.15). Furthermore, there were no differences in any secondary outcomes between the two groups (Table 2).

**Conclusion**: Among multiparous patients who underwent cerclage placement, a history of a prior term birth was not protective against PTB <32 weeks. This study highlights that once the process of cervical shortening/dilation occurs, the risk of PTB does not differ based on prior obstetric history.

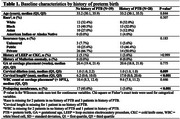

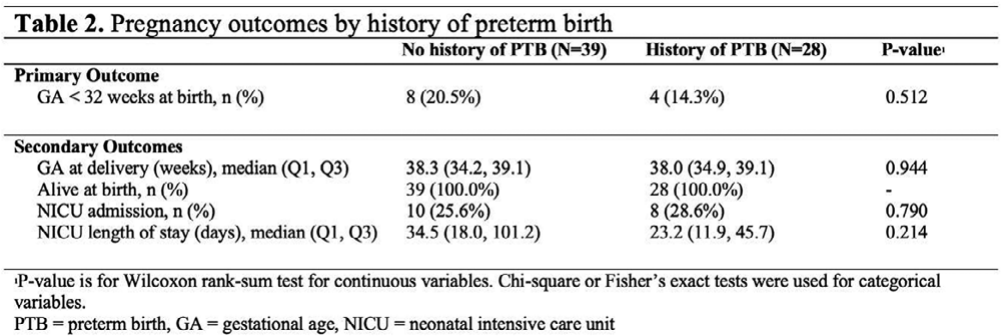

**3:30 PM – 5:00 PM**

## 1173 | **Gastrointestinal Morbidity Following Antenatal Corticosteroid Exposure in Preterm Birth: A Long‐Term Perspective**

Mark Volevich^1^; Tamar Wainstock^2^; Eyal Sheiner^3^; Gali Pariente^4^

^1^Department of Obstetrics and Gynecology, Soroka University Medical Center, Beer sheva, HaDarom; ^2^Department of Epidemiology, Biostatistics and Community Health Sciences, Faculty of Health Sciences, Ben‐Gurion University of the Negev, Beer Sheva, HaDarom; ^3^Soroka University Medical Center, Faculty of Health Sciences, Ben‐Gurion University of the Negev, beer sheva, HaDarom; ^4^Department of Obstetrics and Gynecology, Soroka University Medical Center, Ben‐Gurion University, beer sheva, HaDarom

**Objective**: Antenatal corticosteroid (ACS) therapy reduces neonatal morbidity and mortality in preterm infants. However, the long‐term effects of ACS exposure on gastrointestinal (GI) health remain unclear, and existing evidence on potential risks is limited. We investigated the long‐term GI outcomes of preterm offspring who were exposed to ACS prior to 34 weeks of gestation.

**Study Design**: This population‐based retrospective cohort study included all singleton preterm deliveries at a tertiary medical center between 1991 and 2021. Long‐term GI morbidity was compared between premature infants exposed and non‐exposed to ACS before 34 weeks’ gestation, based on combined databases from community and hospitalization records. Kaplan–Meier survival curve was used to compare the cumulative incidence of GI morbidity and a Cox regression model was constructed to control for confounders.

**Results**: A total of 13,580 premature infants met the inclusion criteria of which 1538 (11.3%) received ACS prior to 34 weeks. The overall GI morbidity rate (132.6 vs. 44.5 per 1000 person‐years; *p* < 0.001, Table) as well as the cumulative incidence over time (Figure) were significantly higher in ACS exposed children as compared to unexposed children. In a Cox regression model, controlling for maternal and gestational age, diabetes mellitus, hypertensive disorders and cesarean delivery, ACS exposure remained an independent risk factor for long‐term GI morbidity (adjusted HR 1.24; 95% CI 1.18–1.30; *p* < 0.001).

**Conclusion**: While ACS therapy is pivotal for reducing immediate neonatal morbidity, our findings raise concern for increased long‐term risk of GI morbidity extending into adolescence. However, the possibility of indication bias cannot be excluded. These findings underscore the need for further research to better understand the underlying mechanisms driving these associations.

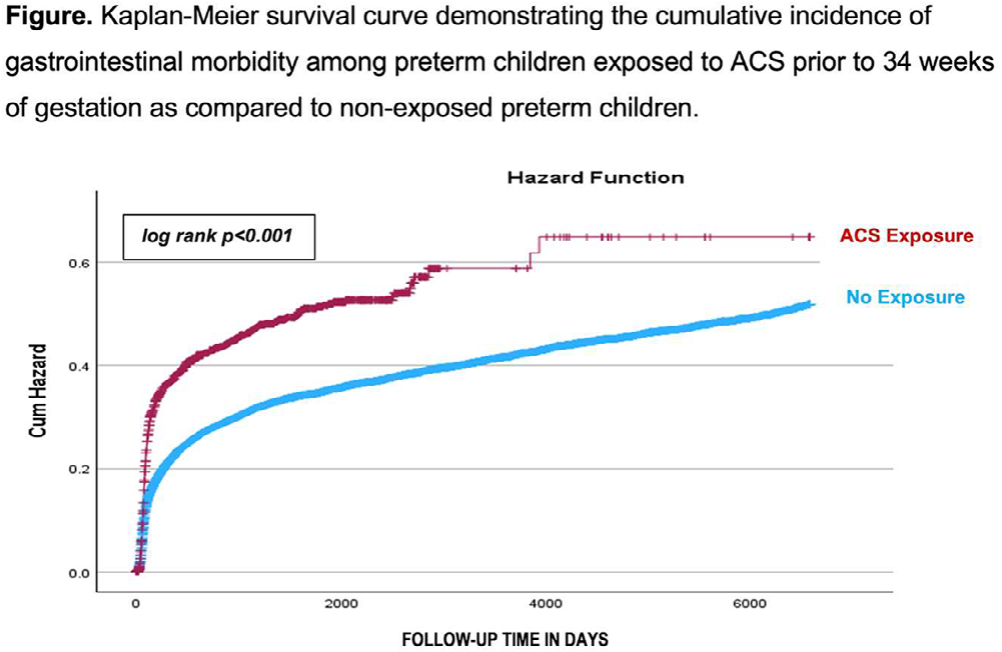

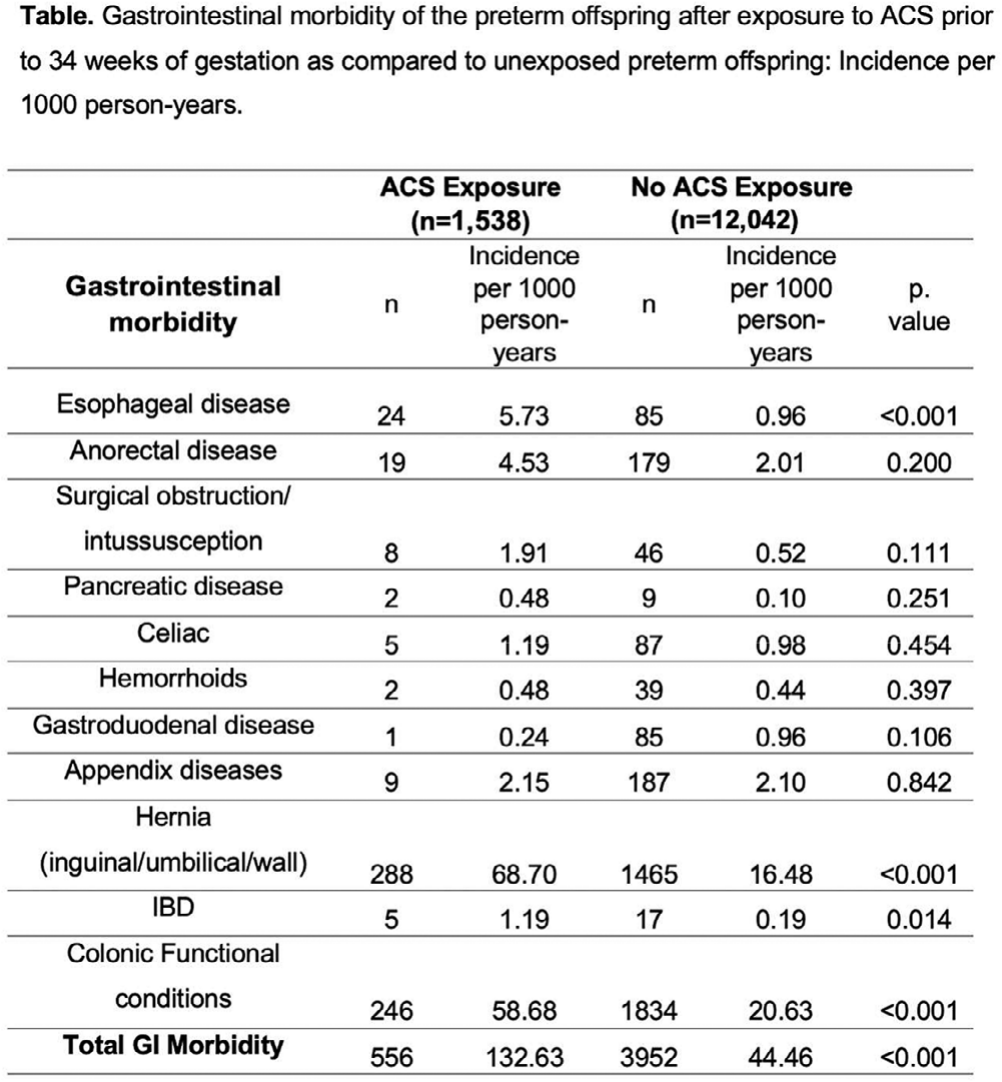

**3:30 PM – 5:00 PM**

## 1174 | **Differences in Engagement and Outcomes in a Postpartum Hypertension Monitoring Program by Infant NICU Admission**

Mary Marchese^1^; Jennifer Okunbor^1^; Allison K. Mak^1^; Scott Machado^1^; Tracy L. Jackson^1^; Nicole Konecke^1^; William Hunt^1^; Crystal F. Ware^1^; Danielle Simmons^1^; Maria Mejia Castillo^1^; Methodius G. Tuuli^2^; Alisse Hauspurg^3^; Adam K. Lewkowitz^1^

^1^Women & Infants Hospital of Rhode Island / Warren Alpert Medical School of Brown University, Providence, RI; ^2^Department of Obstetrics and Gynecology, Warren Alpert Medical School at Brown University, and Women & Infants Hospital of Rhode Island, Providence, RI; ^3^Brown University, Providence, RI

**Objective**: Remote self‐measured blood pressure (SMBP) monitoring programs improve outcomes in the postpartum period among individuals with hypertensive disorders of pregnancy (HDP). However, whether those findings are impacted by infant neonatal intensive care unit (NICU) admission is unknown. Our objective is to examine if infant NICU admission is associated with differences in engagement and outcomes among SMBP program participants.

**Study Design**: Postpartum individuals with HDP at our tertiary hospital are offered enrollment in our remote SMBP program. For this analysis, participants were stratified by those with infants admitted to the NICU or to the standard newborn nursery. The primary outcome was engagement in our SMBP as assessed by the number of participants reporting at least one blood pressure (BP) measurement within the first 10 days postpartum. Secondary outcomes included total number of BP measurements reported through the program, HTN‐related Emergency Department (ED) visit or readmission, initiation of antihypertensive medication, and severe maternal morbidity (SMM). Relative risks were calculated after adjusting for confounders.

**Results**: Among 2556 patients enrolled in the SMBP, 27.7% had infants admitted to the NICU. Compared to those with infants in the nursery, those with infants in the NICU delivered at earlier gestational ages and were more likely to have severe preeclampsia, superimposed preeclampsia, and require ICU admission after delivery. After adjusting for these factors, there was no significant difference in the primary outcome of participants reporting a BP measurement within 10 days postpartum (aRR 0.99, 95% CI 0.89 ‐1.10). There were no significant differences in secondary outcomes including HTN‐related ED visits, readmissions, or SMM.

**Conclusion**: These findings suggest that even though those with infants in the NICU have more severe presentations of HDP and other competing priorities in the postpartum period, SMBP engagement is similar and may mitigate postpartum hypertension‐related morbidity in this population.

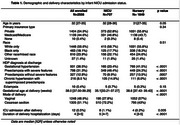

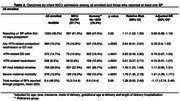

**3:30 PM – 5:00 PM**

## 1175 | **Association of Prior Adverse Pregnancy Outcomes With Health‐Related Social Needs: The BUSY‐BP Study**

Marya Rana^1^; Katherine E. Economy^2^; Kaitlyn E. James^3^; Lauren Belak^4^; Victoria Viscosi^4^; Jaiden Busso^4^; Imuetiyan Eweka^4^; Amy Sarma^4^; Camille E. Powe^4^; Michael C. C. Honigberg^4^

^1^Harvard Medical School, Boston, MA; ^2^Brigham and Women's Hospital, Boston, MA; ^3^Department of Obstetrics and Gynecology, MassGeneral Brigham; Division of Maternal‐Fetal Medicine; Harvard Medical School, Boston, MA; ^4^Massachusetts General Hospital, Boston, MA

**Objective**: Adverse pregnancy outcomes (APOs) are associated with increased risk for postpartum complications, future APOs, and chronic disease, highlighting the importance of follow‐up care. However, health‐related social needs (HRSNs)–the social and economic needs that influence health–can pose barriers to accessing care. Here, we investigated whether APOs, including hypertensive disorders of pregnancy (HDP), gestational diabetes (GDM), preterm birth (PTB, <37 weeks), or low birthweight (LBW, <2.5 kg), are associated with HRSNs.

**Study Design**: We included parous participants in the BUSY‐BP study of Black and/or Latina women aged 18‐65 years. Participants completed the Centers for Medicare and Medicaid Services Accountable Health Communities screening tool, which assesses 13 domains of HRSNs (Table 1). Cumulative burden of HRSNs was calculated as the sum of domains of need for each participant (range 0‐13). The primary exposure was any prior APO, and the primary outcome was a composite HRSN score ≥7 (top quartile of HRSN scores). Logistic regression tested the association of APO history with HRSNs, adjusted for age, years since last pregnancy, and parity.

**Results**: Among 1231 participants (mean [SD] age 40.1 [10.7] years, 794 [64.5%] Black, 642 [52.2%] Latina),  521 (42.3%) reported ≥1 previous APO (219 [17.8%] HDP, 149 [12.1%] GDM, 195 [15.8%] LBW, 288 [23.4%] PTB). Prevalence of prior APOs increased with a higher cumulative burden of HRSNs (Figure 1). Compared with no previous APOs, history of APOs was associated with 1.67‐fold odds of a high HRSN score (i.e., ≥7 domains of need; adjusted OR 1.67, 95% CI 1.22‐2.31, *p* = 0.002). Educational needs were most strongly associated with prior APOs, and among APOs, HDP and GDM were associated with the greatest number of HRSNs (Table 1).

**Conclusion**: APO history was associated with greater burden of HRSNs, highlighting APO occurrence as a potential opportunity for intervention to address both medical and social factors associated with adverse health outcomes.

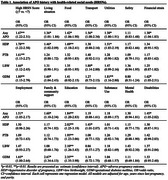

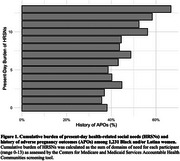

**3:30 PM – 5:00 PM**

## 1176 | **Development and Validation of a Screening Tool Incorporating Fetal Indicators to Predict Severe Maternal Morbidity**

Matthew J. Blitz^1^; Insaf Kouba^2^; Frank I. Jackson^1^; Alejandro D. Alvarez^1^; Dimitre Stefanov^1^; Dawnette Lewis^1^; Adriann Combs^1^; Michael Nimaroff^1^; Burton Rochelson^1^

^1^Northwell, New Hyde Park, NY; ^2^Johns Hopkins All Childrens, St. Petersburg, FL

**Objective**: To evaluate whether fetal factors independently predict severe maternal morbidity (SMM) and to compare the predictive performance of fetal, maternal, and combined models for SMM risk stratification.

**Study Design**: This retrospective cohort study included 110,319 patients delivering across a large health system from 2019–2023. To ensure independent observations, only each patient's first delivery was analyzed. The dataset was randomly divided into training, validation, and test subsets (*n* = 36,773 each). Fetal predictors included oligohydramnios, polyhydramnios, fetal growth restriction, large‐for‐gestational‐age (LGA), abnormal umbilical artery Dopplers, and fetal anomaly. Maternal predictors were based on the obstetric comorbidity index (OB‐CMI). SMM was defined using CDC criteria. Model performance was evaluated in the test dataset based on the area under the receiver operating characteristic curve (AUC‐ROC) derived from logistic regression. Three models were evaluated: (1) maternal factors, (2) fetal factors alone, and (3) combined maternal + fetal model. ROC contrast tests compared AUC performance.

**Results**: Fetal factors were independently associated with SMM, but predictive performance was limited (AUC 0.54; 95% CI, 0.53–0.55). Only polyhydramnios and LGA showed significant associations. The maternal model (OBCMI) performed better (AUC 0.65; 95% CI, 0.64–0.67), while the combined maternal + fetal model had the highest performance (AUC 0.69; 95% CI, 0.68–0.70). ROC contrast testing confirmed that the combined model significantly outperformed both fetal‐only and maternal‐only models (*p* < 0.001 for both).

**Conclusion**: Fetal factors contribute independently to SMM risk but offer limited predictive value on their own. Integrating fetal and maternal data improves model performance, supporting combined risk stratification.

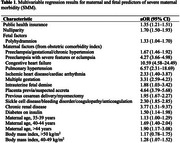

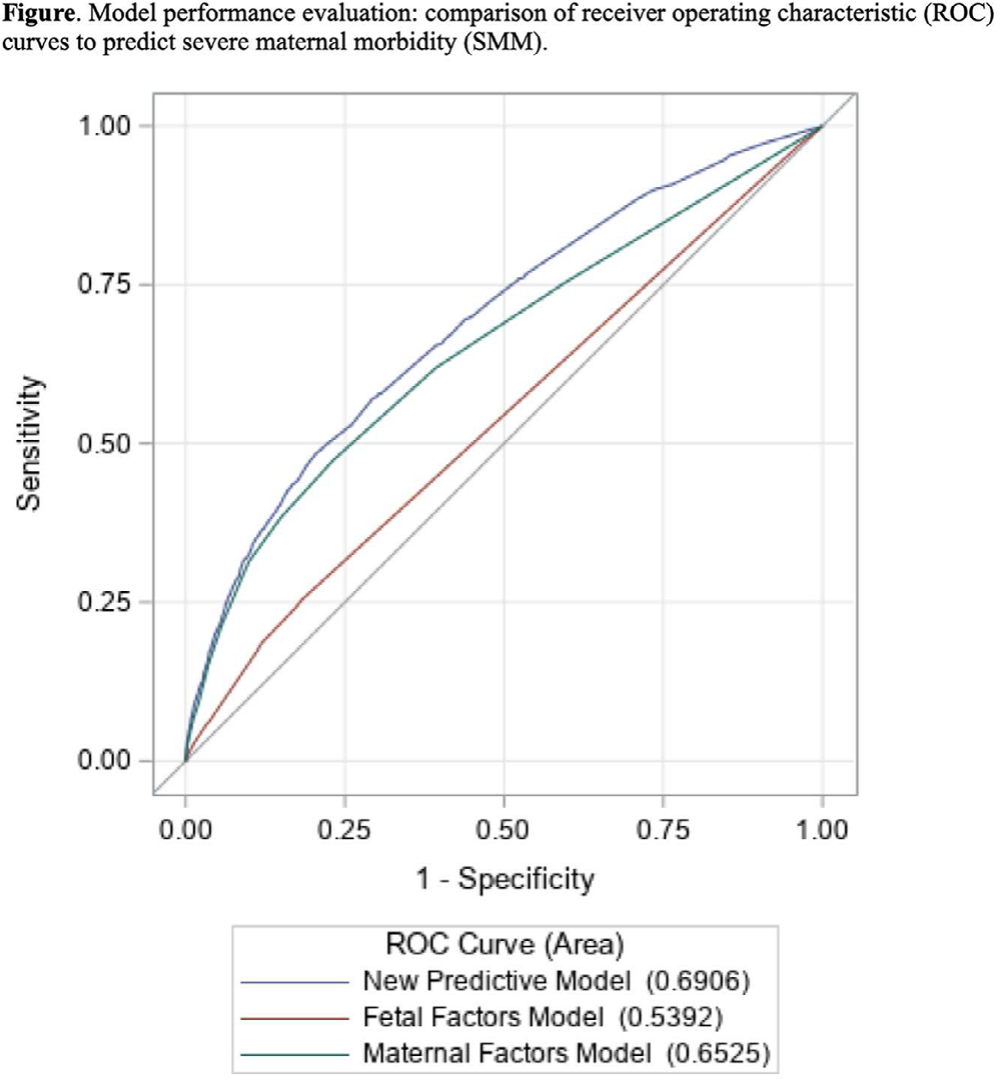

**3:30 PM – 5:00 PM**

## 1177 | **BMI Effects on Placenta Accreta Spectrum Grade Severity**

Maxwell Oberlander^1^; Rodney A. McLaren, Jr^1^; Tirtza Spiegel Strauss^1^; Henri M. Rosenberg^2^; Rebecca H. Jessel^3^; Alexa L. Cohen^4^; Georgios Doulaveris^5^; Pe'er Dar^5^; Meghana A. Limaye^6^; Emily Schlussel Markovic^1^; Joselle O'Brien^1^; Angela T. Bianco^7^; Shoshana Haberman^1^; Scott Chudnoff^1^; Itamar Futterman^8^

^1^Maimonides Medical Center, Brooklyn, NY; ^2^Icahn School of Medicine at Mount Sinai, Icahn School of Medicine at Mount Sinai, NY; ^3^Division of Maternal‐Fetal Medicine, Department of Obstetrics and Gynecology, NYU Grossman School of Medicine, New York, NY; ^4^University of Miami, Miami, FL; ^5^Montefiore Medical Center/Albert Einstein College of Medicine, Montefiore Medical Center/Albert Einstein College of Medicine, NY; ^6^Division of Maternal‐Fetal Medicine, Department of Obstetrics and Gynecology, NYU Grossman School of Medicine, NYU Langone, NY; ^7^Icahn School of Medicine at Mount Sinai, New York, NY; ^8^Maimonides Medical Center, Maimonides Health, NY

**Objective**: Recent evidence suggests that obesity affects placental development, leading to adverse pregnancy outcomes. Whether obesity affects the pathophysiology of placenta accreta spectrum (PAS) is unknown. Thus, the objective of this study was to evaluate if maternal pre‐pregnancy body mass index (BMI) correlates with increased PAS grade severity.

**Study Design**: A multicenter retrospective cohort study of histopathologically confirmed PAS cases 1/1/2013 ‐ 6/30/2022 at five New York academic institutions. Obstetrical and demographic variables were collected. The cohort was divided into two groups, obese (BMI ≥30 kg/m^2^) and non‐obese (BMI <30 kg/m^2^). Univariable analysis was performed comparing variables using chi square test for categorical and Student t‐test/Wilcoxon rank sum for continuous data. An ordinal regression analysis was then performed adjusting for statistically significant obstetrical and demographic variables between the two groups, comparing median PAS grade. Finally, we divided the cohort by PAS grades and compared the median BMI in each group.

**Results**: A total of 401 cases of PAS were collected. Of these, 166 had a documented histopathological grade of PAS. There were 26 obese patients and 140 non‐obese. Demographics are presented in Table 1. The proportion of Black and Hispanic individuals was greater in the obese group (30.8% vs. 14.5% and 11.5% vs 5.1%, respectively, *p* = 0.025). Individuals in the obese group had a significantly greater number of prior cesarean sections [2 (1,3) vs. 1 (1,2), *p* = 0.045]. There were no other differences between the two groups in social and obstetrical variables. For the primary outcome, there were no differences in median PAS grade between the obese and non‐obese groups [1 (1,2) vs 1 (1,2) *p* = 0.67, regression coefficient ‐0.35, 95% CI (‐0.94‐0.22]. Additionally, there were no differences in incidence of obesity and median BMI among the different grades of PAS (Table 2).

**Conclusion**: There were no differences in both severity of PAS disease and incidence of PAS grade between obese and non‐obese patients with histopathologically confirmed PAS.

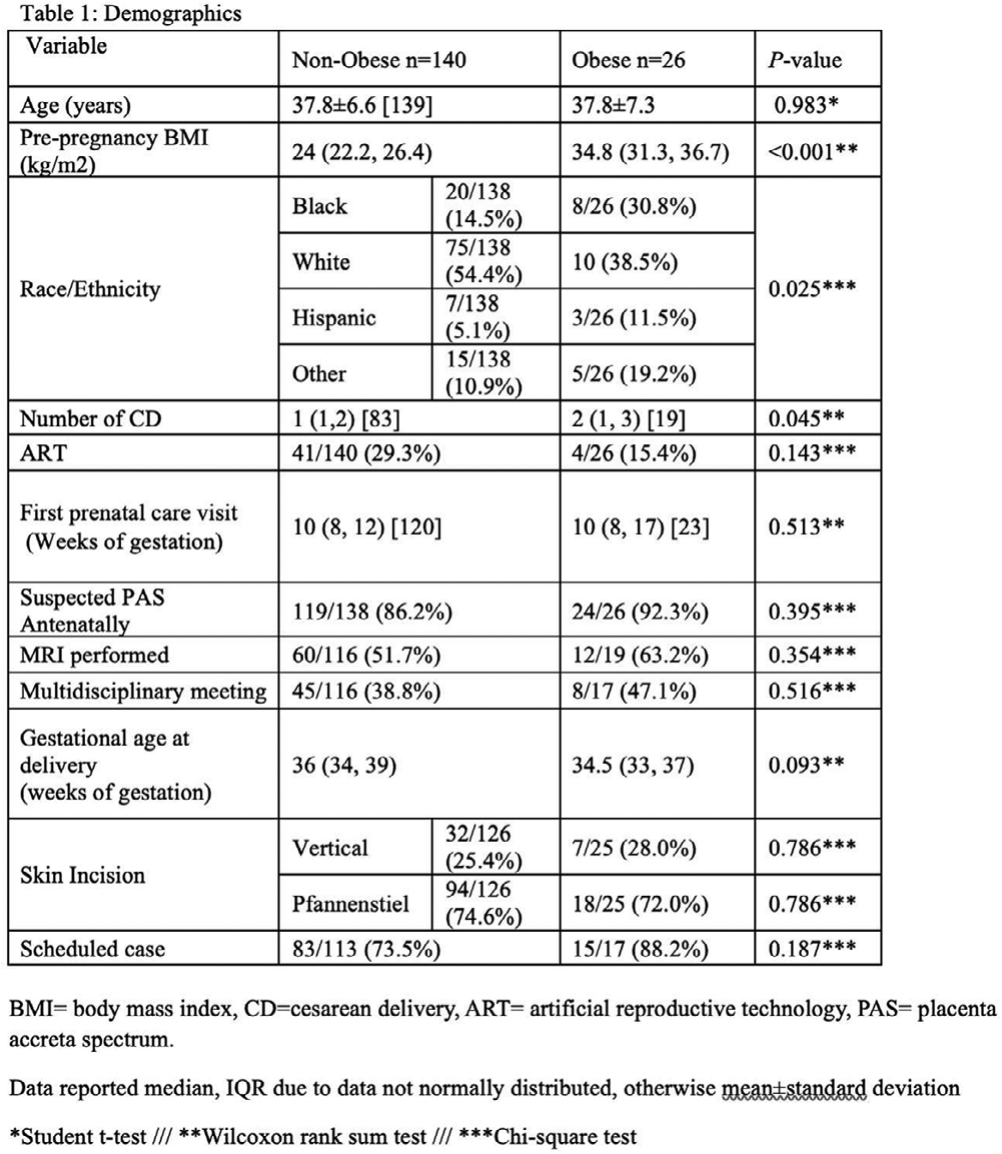

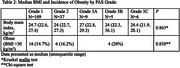

**3:30 PM – 5:00 PM**

## 1178 | **Resolved Fetal Growth Restriction Is Not Risk‐Free: An Analysis of Maternal Factors and Neonatal Outcomes**

Megan R. Ansbro^1^; Marissa A. Hand^1^; Caroline Pennacchio^2^; Hannah E. Bowers^1^; Lydia DeAngelo^3^; Kirat Sandhu^1^; Maeve Hopkins^4^; Ahmed Ahmed^5^; Amol Malshe^5^

^1^Cleveland Clinic Foundation, Cleveland Clinic Foundation, Cleveland, OH; ^2^University of Pittsburgh Medical Center, University of Pittsburgh Medical Center, PA; ^3^Cleveland Clinic Foundation, Cleveland Clinic, Cleveland, OH; ^4^Cleveland Clinic Foundation, Cleveland, OH; ^5^Cleveland Clinic Foundation, Cleveland Clinic Foundation, OH

**Objective**: To identify maternal factors linked to fetal growth restriction (FGR) resolution and compare neonatal outcomes between resolved and persistent FGR at delivery

**Study Design**: We performed a retrospective cohort study of FGR pregnancies delivered at Cleveland Clinic from January 1, 2010 to July 31, 2022 (IRB# 21‐1064). Using EPIC and REDCap, we included cases with ultrasound‐estimated fetal weight (EFW) or abdominal circumference (AC) <10th percentile. We defined persistent FGR as EFW/AC remaining <10th percentile throughout gestation. Resolved FGR had final EFW/AC ≥10th percentile. We excluded pregnancies <24 weeks, multifetal gestations, fetal anomalies, or genetic disorders. We analyzed maternal contributors to resolution using logistic regression and point‐biserial correlation. Neonatal outcomes were compared with t‐tests and chi‐square. Delta heatmaps identified associations across variables.

**Results**: Among 763 FGR pregnancies, 254 remained persistent and 145 resolved. Higher maternal BMI at delivery and increasing parity were significantly associated with FGR resolution. Gestational diabetes and hypertensive disorders showed no such association (Figure 1). Cesarean delivery for non‐reassuring fetal status occurred in 11.8% of persistent vs. 9% of resolved cases (*χ^2^* = 0.39, *p* = 0.53). Persistent FGR neonates trended toward lower 10‐minute Apgar scores and longer NICU stays (25 vs. 18 days, *p* = 0.086). Intubation and CPAP durations did not differ significantly between groups.

**Conclusion**: In this large cohort, higher maternal BMI and parity predicted FGR resolution. However, resolved FGR neonates experienced similar risks to those with persistent FGR, including longer NICU stays and comparable respiratory support needs. Resolution by ultrasound alone may not reflect full physiologic recovery. These findings underscore the importance of continued antenatal surveillance and tailored perinatal management, even in pregnancies where FGR appears to resolve.

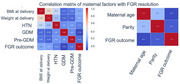

**3:30 PM – 5:00 PM**

## 1179 | **Revisiting Nuchal Translucency Thresholds for Predicting Fetal Anomalies in Low Risk Cell‐Free DNA**

Melissa E. Chambers^1^; Luis Torres^1^; Aparna Murali^1^; Alexandra Shambayte Lopez^1^; Lorna Kwan^2^; Thalia Mok^3^

^1^Division of Maternal Fetal Medicine, Department of Obstetrics and Gynecology, David Geffen School of Medicine at University of California, Los Angeles (UCLA), Los Angeles, CA; ^2^Department of Urology, David Geffen School of Medicine at University of California, Los Angeles (UCLA), Los Angeles, CA; ^3^David Geffen School of Medicine at University of California, Los Angeles (UCLA), Los Angeles, CA

**Objective**: Nuchal translucency (NT) is a well‐established ultrasound marker for aneuploidy. Since implementation of cell‐free DNA (cfDNA), use of the NT measurement has decreased. This study aims to investigate the role of NT in a low risk cfDNA cohort for prediction of fetal outcomes.

**Study Design**: Retrospective cohort study of pregnancies with NT measurement 3 mm or greater between 11 to 14 weeks gestation and a low risk cfDNA result from January 2012 to June 2024. Primary outcome was development of a composite major abnormal ultrasound (US). Secondary outcomes included abnormal diagnostic testing and composite abnormal soft US marker. Univariate analysis was performed using Chi‐square or Fisher‐exact to assess outcomes by NT measurements. Multivariate logistic regression calculated adjusted odds ratios (aOR). Area under the curve (AUC) from receiver operating characteristic (ROC) curves were compared to assess the optimal cutoff for NT to predict a major abnormal US.

**Results**: 211 pregnancies had a low risk cfDNA but an increased NT 3 mm or greater. 30 (14.2%) had a composite major abnormal US and 11 (5.2%) had a composite abnormal soft US marker. Diagnostic testing was performed in 82 (40.0%) patients, and abnormal diagnostic testing results were found in 12 (14.8%). The median NT measurement for abnormal US was greater than normal US (3.9 mm, (IQR 3.5, 6.4) vs 3.3 mm (IQR 3.1, 3.6); *p* < 0.01). Increased NT was associated with significantly higher rates of composite abnormal US (*p* < 0.01). NT of > 4.5 mm compared to 3.0‐3.4 mm had a significantly increased risk of fetal anomalies (aOR 32.5, 95% CI 8.7‐121.4). NT threshold of 3.3‐3.8 mm and 4.5 mm had significantly higher AUC than 3.1 mm on ROC curves (Figure 1).

**Conclusion**: Only 14% of pregnancies with low risk cfDNA and increased NT had a composite abnormal US, but higher NT measurement was associated with a significantly increased risk of abnormal US. The first trimester ultrasound and NT remain valuable components of prenatal screening, but a higher threshold for increased NT measurement may be considered when evaluating risk of fetal anomalies.

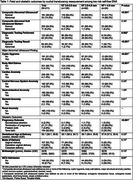

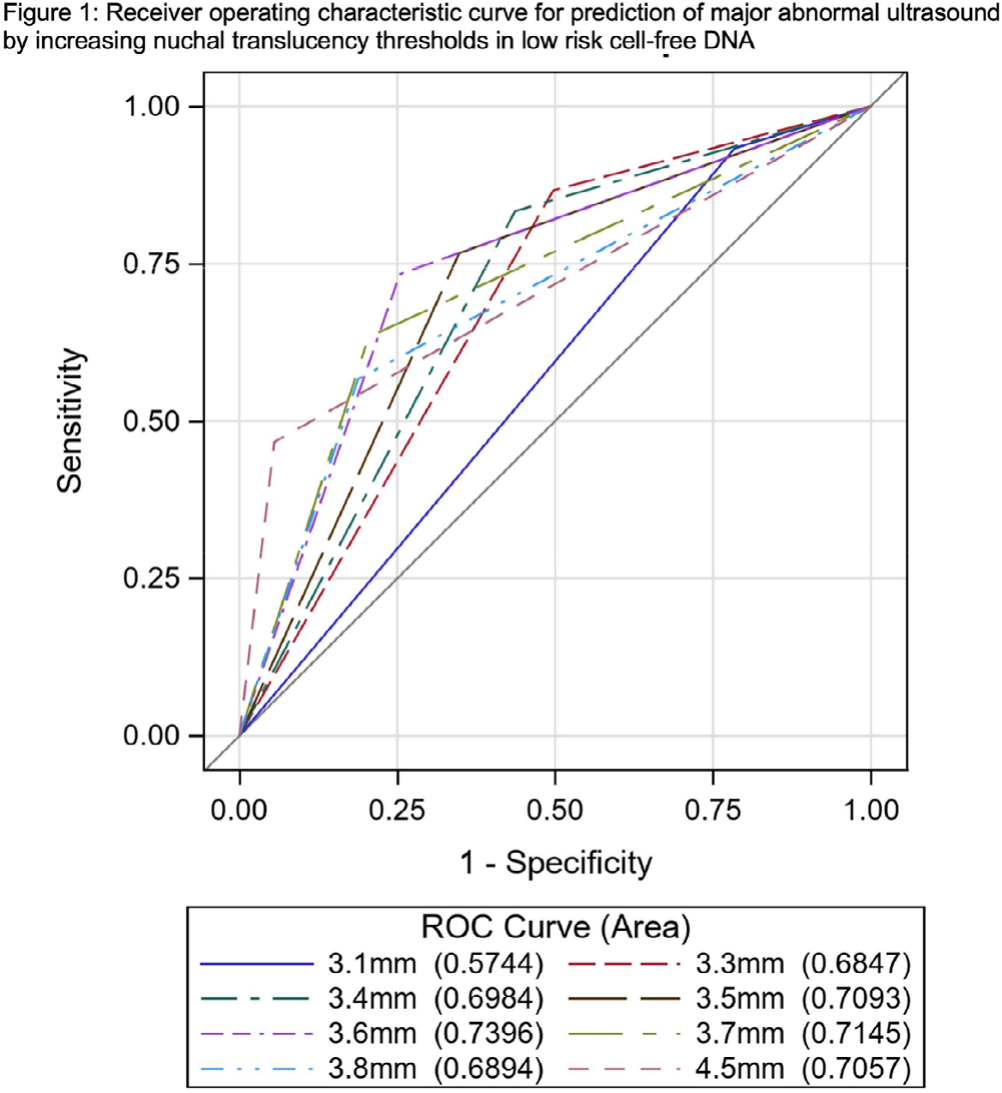

**3:30 PM – 5:00 PM**

## 1180 | **Single Versus Seven‐Day Treatment Dosing for Metronidazole in Pregnancies Complicated by Trichomonas Vaginalis Infections**

Daniel J. Martingano^1^; Melody Boafo^2^; Andrea Rivera^3^; Ashley Nguyen^2^; Marwah Al‐Dulaimi^4^; Ginny Zizhen Liu^5^; Jaina Diaz‐Kelly^2^; Kristen Henry^2^; Kelly Mondey^2^; Brenna M. Regan^6^; Gloria Otoo^2^; Kristen Cohen^7^; Eddie Santana^8^; Angelo Oduro^1^; Jacqueline Marecheau^2^

^1^St. John's Episcopal Hospital‐South Shore / Lake Erie College of Osteopathic Medicine, St. John's Episcopal Hospital‐South Shore / Far Rockaway, NY; ^2^St. John's Episcopal Hospital‐South Shore, St. John's Episcopal Hospital‐South Shore / Far Rockaway, NY; ^3^Wyckoff Heights Medical Center, Wyckoff Heights Medical Center / Brooklyn, NY; ^4^St. John's Episcopal Hospital‐South Shore, St. John's Episcopal Hospital‐South Shore, NY; ^5^William Carey University, William Carey University, MS; ^6^William Carey University College of Osteopathic Medicine, William Carey University / Hattiesburg, MS; ^7^RWJBarnabas Health Trinitas Regional Medical Center, RWJBarnabas Trinitas Regional Medical Center / Elizabeth, NJ; ^8^Good Samaritan University Hospital / NYIT, Good Samaritan University Hospital / West Islip, NY

**Objective**: Trichomonas vaginalis (TV) commonly affects pregnant women with an increasing incidence and has been linked to several adverse birth outcomes. This study sought compare the effectiveness of different dosing regimens for metronidazole (MT) in the treatment of TV in 2^nd^ and 3^rd^ trimester pregnancies.

**Study Design**: We conducted a multi‐center, prospective observational study from 7/2022 to 7/2025 and included all pregnant women diagnosed with TV from 20 0/7 to 40 0/7 weeks‐gestation receiving monotherapy antibiotic treatment with oral MT. Drug regimens considered include a single dose of 2000mg (SD) or a 7‐day course of 500mg twice daily (WD). Choice of regimen was determined by physician preference. Primary outcomes included need for additional medication doses, positive test‐of‐cure (performed at 4 weeks post‐treatment), ascending infection (Fitz‐Hugh‐Curtis Syndrome), sepsis, early preterm delivery defined at <34 0/7 weeks‐gestation, late preterm delivery defined at 34 1/7 to 36 6/7 weeks‐gestation, preterm prelabor rupture of membranes (PPROM) defined at <37 0/7 weeks‐gestation, and fetal growth restriction defined as EFW <10^th^ percentile, as discrete events. Patients were excluded if they received additional antibiotics within a week before TV diagnosis, received treatment IV or IM, had preexisting immunologic conditions, or had concurrent sexually transmitted infections.

**Results**: The study included 1127 patients, with 473 receiving SD and 654 receiving WD. Baseline demographic factors were not significantly different. Patients receiving SD had lower rates of positive test‐of‐cure (21.9% v. 36.2% *p* = 0.003), and need for additional medication (19.4% v. 35.1% *p* = 0.004), with a 33% (RR = 0.67, 95% CI 0.41‐0.90, *p* = 0.022) and 29% (RR = 0.71, 95% CI 0.43‐0.86, *p* = 0.030 decreased risk in confounder adjusted models, respectively. Patients receiving WD had increased rates of PPROM (6.7% v. 4.9% *p* = 0.010), and a 19% (RR = 1.19, 95% CI 1.03‐1.44, *p* = 0.030) increased risk in adjusted models.

**Conclusion**: Single‐dose metronidazole is a reasonable and possible superior dosing choice for pregnancies complicated by TV.

**3:30 PM – 5:00 PM**

## 1181 | **External Validation of Predictive Models for Postpartum Readmission for Hypertension Management**

Meralis V. Lantigua‐Martinez^1^; Steven Friedman^2^; Justin S. Brandt^1^; Erinn M. Hade^2^; Christina A. Penfield^3^

^1^Division of Maternal‐Fetal Medicine, Department of Obstetrics and Gynecology, NYU Grossman School of Medicine, New York, NY; ^2^Division of Biostatistics, Department of Population Health, NYU Grossman School of Medicine, New York, NY; ^3^Division of Maternal‐Fetal Medicine, Department of Obstetrics and Gynecology, NYU Grossman School of Medicine, New York City, NY

**Objective**: Prediction models for the risk of postpartum readmission for hypertension management (PRHM) have been developed but require external validation. We externally validated and compared the performance of two published risk prediction models by Venkatesh et al. (Model #1) and McLaren et al. (Model #2) in a large cohort.

**Study Design**: Using a large observational cohort of all deliveries at a New York City health system between 1/2020‐6/2024, we externally validated Model #1 and Model #2. We defined PRHM as a readmission within 42 days of delivery in which rapid acting antihypertensive medications (i.e. IV labetalol, IV hydralazine, or immediate release nifedipine) were administered. Both models include maternal age, delivery encounter blood pressure data points, and preeclampsia diagnosis; Model #1 further includes parity, birthweight, and delivery mode (race neutral) while Model #2 includes race/ethnicity (race‐conscious) beyond the initial set. We evaluated each model through recalibration in the large, logistic recalibration, and model estimate revision. Model discrimination was assessed with the c‐statistic and calibration with the Brier statistic and E‐average.

**Results**: Of 41,216 deliveries, 535 (1.3%) resulted in PRHM. Those readmitted were more often older, had higher diastolic blood pressure prior to discharge, had a diagnosis of severe preeclampsia, and delivered by cesarean section (Table 1). We found moderate performance of both models for predicting PRHM, with c‐statistics ranging from 0.75‐0.77 (Table 2) and acceptable calibration when the prediction model was allowed to estimate a model intercept slope for our cohort.

**Conclusion**: In this large, diverse cohort, two published models for predicting PRHM demonstrated moderate discrimination and calibration. Performance improved with model updating, underscoring the need for tailoring prediction tools to the target population before clinical implementation. In line with other models and calculators that have excluded race without degrading performance, the race‐neutral model performed well.

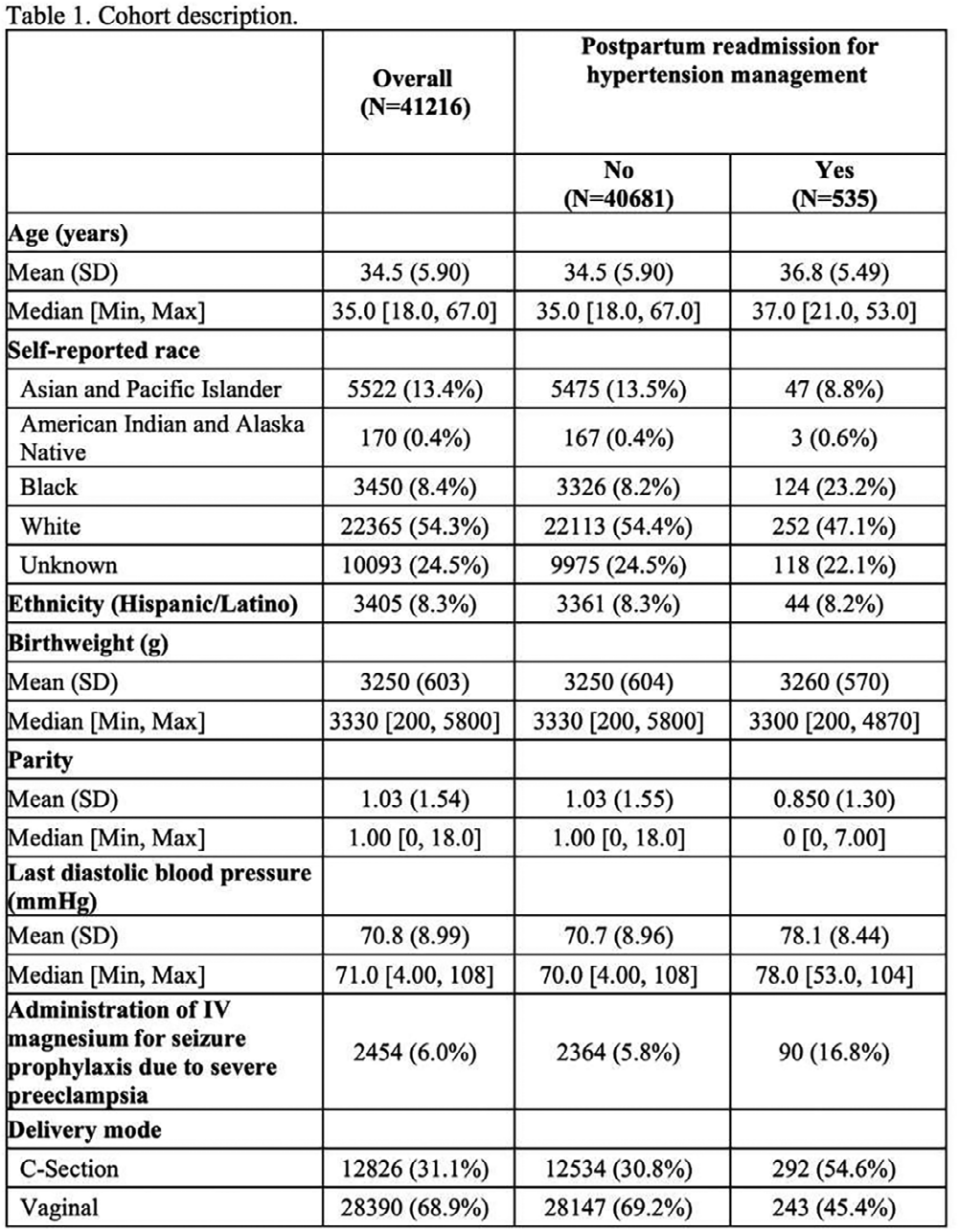

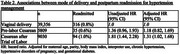

**3:30 PM – 5:00 PM**

## 1182 | **Adverse Childhood Experiences and Postpartum Healthcare Utilization Following Cesarean Birth: A Pilot Prospective Cohort Study**

Metabel T. Markwei^1^; Noor H. Joudi^2^; Janet Hurtado^1^; Samantha L. Simpson^1^; Nidhee S. Reddy^1^; Jordan J. Burgess^2^; Elizabeth B. Sherwin^3^; Stephanie A. Leonard^4^; Miriam Schultz^1^; Brendan Carvalho^1^; Pervez Sultan^1^; Katherine Bianco^1^; Danielle M. Panelli^1^

^1^Stanford University Healthcare, Palo Alto, CA; ^2^Stanford University Healthcare, Stanford University Healthcare, CA; ^3^Stanford University, Stanford, CA; ^4^Stanford University, Stanford University, CA

**Objective**: Adverse childhood experiences (ACEs) affect over 60% of U.S. adults and are linked to chronic disease, opioid use, and increased healthcare utilization in non‐pregnant populations. Yet, their impact during the postpartum period—a time of heightened vulnerability—is poorly understood. We evaluated the association between ACEs and postpartum emergency department (ED) visits or hospital readmissions within six weeks of cesarean birth.

**Study Design**: We analyzed data from a prospective cohort study of patients aged 18–55, literate in English or Spanish, who delivered a singleton liveborn infant via low‐transverse cesarean under neuraxial anesthesia at a single tertiary care center (2023–2024). ACE scores (0–10) were collected using a validated tool and categorized by trauma subtype (abuse, neglect, household dysfunction). ACEs were modeled using two cutoffs: >1 ACE versus none, and >4 ACEs versus <4, in accordance with prior literature. The primary outcome was any postpartum ED visit or hospital readmission within 6 weeks of cesarean birth. Modified Poisson regression was used to estimate risk ratios (RR), adjusting for confounders.

**Results**: Among 129 participants, 41% reported ≥1 ACE, and 19% had a postpartum ED visit or readmission. Postpartum ED visits or readmissions were more common among those with ACEs compared to those without (36% vs 8%, Figure 1). Compared to participants without ACEs, those with ≥1 ACE had an increased risk of ED visit or readmission (aRR 5.50, 95% CI 2.41–12.6). Similarly, those with a high ACEs burden (≥4) had increased risk (aRR 3.28, 95% CI 1.55–6.93, *p* = 0.002), compared to those with <4 ACEs (Table 1). All ACE trauma subtypes were independently associated with increased risk of postpartum ED visit or readmission (Table 1).

**Conclusion**: ACEs were strongly associated with increased postpartum ED visits and hospital readmissions following cesarean birth. Trauma‐informed care programs that incorporate routine ACE screening into peripartum care may help identify those at risk of postpartum complications and mitigate preventable postpartum hospital encounters in this population.

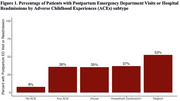

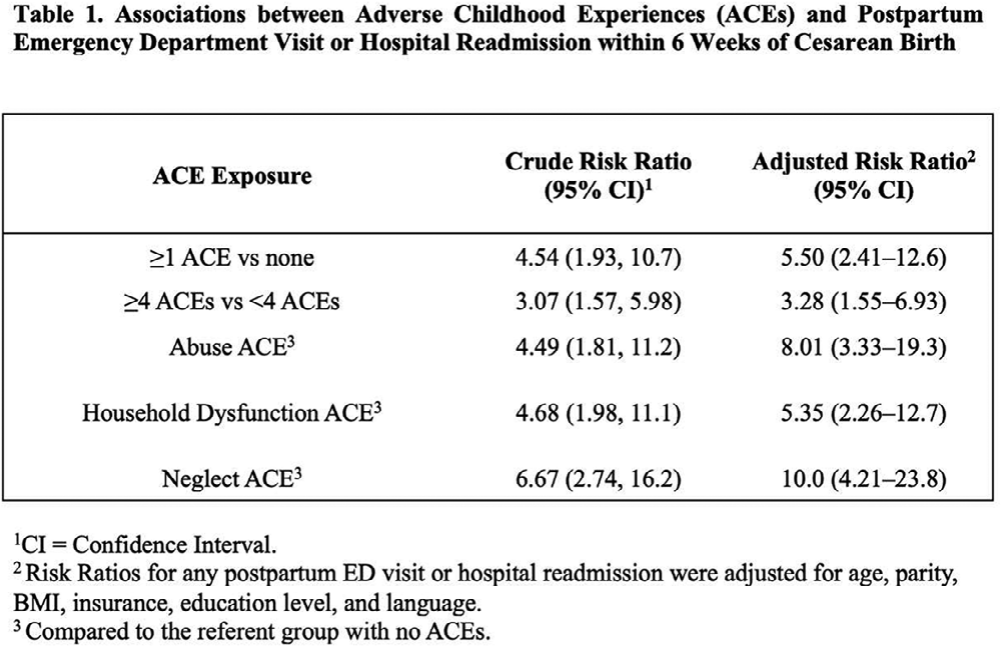

**3:30 PM – 5:00 PM**

## 1183 | **Fasting Blood Glucose (FBS) Targets in GDMA1: A Clinical Practice Comparison**

Mia A. Heiligenstein^1^; Sophia Tully^2^; Xiteng Yan^3^; Thomas Owens^4^; Ceyda Oner^5^; Shelly Thai^5^; Jo Hsuan Lee^6^; Guillaume Stoffels^7^; Leslie Rebarber^5^; Tata Coblic Zelter^8^; Casey Seiden^9^; Jennifer Lam‐Rachlin^6^; Nathan S. Fox^3^; Andrei Rebarber^10^; Zainab Al‐Ibraheemi^11^; Lois Brustman^3^

^1^Mount Sinai West, Astoria, NY; ^2^Mount Sinai West, New York, NY; ^3^Icahn School of Medicine, Mount Sinai West, New York, NY; ^4^Icahn School of Medicine at Mount Sinai, Atlanta, GA; ^5^Mount Sinai West, New York, NY; ^6^Icahn School of Medicine at Mount Sinai, Icahn School of Medicine at Mount Sinai, NY; ^7^Icahn School of Medicine at Mount Sinai, New York, NY; ^8^Mayanei HaYeshua Medical Center, New York, NY; ^9^MFMA, New York, NY; ^10^Icahn School of Medicine, Mount Sinai West, Mount Sinai West, NY; ^11^Mount Sinai West, New York City, NY

**Objective**: No standard FBS threshold exists to start hypoglycemic agents in GDM.While ACOG recommends targeting FBS< 95 mg/dL, some clinicians use lower thresholds based on the HAPO study linking lower FBS with fewer adverse outcomes.This study compares adverse outcomes in GDMA1 patients managed by two diabetic programs with different FBS thresholds to initiate hypoglycemic therapy.

**Study Design**: Retrospective review from 2018‐2024 compared GDMA1 patients‐defined as those not on hypoglycemic agents at delivery‐in two urban diabetic programs.Both used similar diagnostic criteria and began management with diet and lifestyle changes.Practice #1(PR#1) initiated hypoglycemic agents when >50% FBS values were≥95 mg/dL, while Practice #2(PR#2) used a lower threshold of ≥90 mg/dL.Both defined postprandial control as< 50% of 2hr postprandial glucose< 120 mg/dL or 1hr< 140 mg/dL.Primary outcomes were a composite of neonatal adverse events(hypoglycemia, NICU admission, large for gestational age[LGA], and polyhydramnios);secondary outcomes included cesarean section(CS) rates and birth weight.Multivariable logistic regression adjusted for confounders;odds ratios with 95% confidence intervals(CI) were calculated.

**Results**: 194 women were treated in PR#1 and 921 in PR#2.Groups differed in age, race, gestational age at diagnosis, history of GDM, and gravidity(Table 1).Mean gestational age at delivery was similar.There was no significant difference in composite neonatal outcomes(*p* = 0.073).However, CS rates were significantly higher in PR#1(Table 2).There were significant differences in NICU admissions, LGA, and polyhydramnios(Table 2):PR#1 had nearly 4‐fold higher odds of LGA and over 10‐fold higher odds of polyhydramnios but 66% lower odds of NICU admission compared to PR#2.

**Conclusion**: Managing GDMA1 with a lower FBS goal(<90 mg/dL)was associated with improved perinatal outcomes compared to <95 mg/dL.Differences in patient baseline demographics and the retrospective design may limit external validity.Our findings suggest the need for an RCT to determine the optimal FBS value to be used in the treatment of GDM.

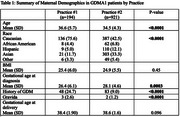

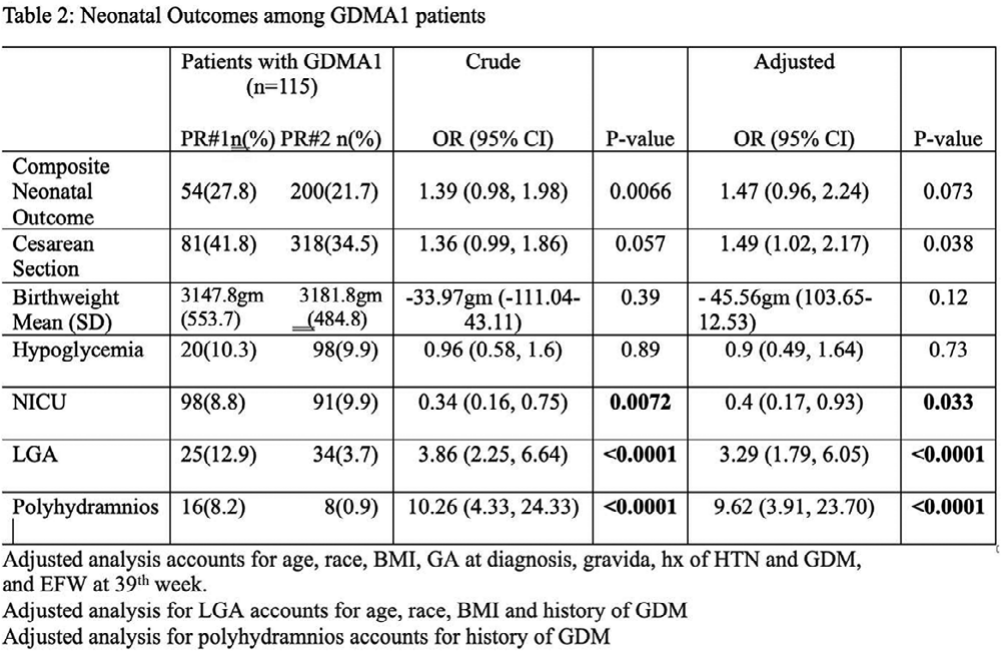

**3:30 PM – 5:00 PM**

## 1184 | **Trophoblast Injury Post in Vitro Percussion Trauma**

Michael J. Paidas^1^; Emily M. West^2^; Anna R. Speciale^2^; Rajalakshmi Ramamoorthy^2^; Aleezeh Shaikh^2^; Ganapathi Kandasamy^2^; Anis Ahmad^2^; Nayab Ahmad^2^; Edna Porter^2^; Arumugam R. Jayakumar^2^

^1^Univ Miami Miller School of Medicine, Univ Miami/ Miami, FL; ^2^University of Miami Miller School of Medicine, Miami, FL

**Objective**: Trauma is the leading cause of non‐obstetrical maternal death and is associated with adverse pregnancy outcomes, including placental abruption and fetal demise.   Although placental injury is a recognized consequence of maternal trauma, its direct effects on trophoblast function are not well understood. This study aims to investigate the cellular effects of percussion trauma on human trophoblasts in vitro.

**Study Design**: Primary cultures of human placental extravillous trophoblasts (HTR‐8/SVneo) were subjected to fluid percussion trauma (FPI, 5 atm pressure; *n* = 6 Control; *n* = 6 Trauma), and were analyzed 48 h after trauma, which had been validated in our traumatic brain injury models. Cell viability was assessed with a fluorometric assay. Immunofluorescence and Western blot analyses were performed to measure the levels of mitochondrial markers (Translocase of the Outer Mitochondrial membrane (TOM20), Cytochrome C Oxidase subunit 1 (COX1), Voltage‐Dependent Anion Channel 1 (VDAC1), Succinate Dehydrogenase Complex subunit A (SDHA) and the antiangiogenic factor Soluble Fms‐Like Tyrosine kinase‐1 (sFlt‐1). Data were analyzed using the Mann‐Whitney test.

**Results**: Trophoblasts exposed to trauma showed a 30% decrease in cell viability compared to controls (*p* < 0.0043) (Figure 1 A & B). Immunofluorescence revealed significantly higher levels of sFlt‐1 (Figure 1C) and SDHA (Figure 1D) (42% and 59%, respectively; *p* = 0.001) in the injured cells, indicating a cellular stress response. Western blot analysis showed a twofold decrease in TOM20 levels (53%, *p* < 0.001) and a slight reduction in VDAC1 (9%), while COX1 levels remained unchanged (Figure 2 A‐D).

**Conclusion**: Mechanical trauma leads to significant mitochondrial changes and cellular stress in human trophoblasts, evidenced by decreased viability, reduced expression of TOM20 and VDAC1, and increased levels of sFlt‐1 and SDHA, indicating altered anti‐angiogenic signaling. These patterns are similar to those observed in hypoxia‐related placental disorders. This may explain how percussion trauma contributes to adverse pregnancy outcomes.

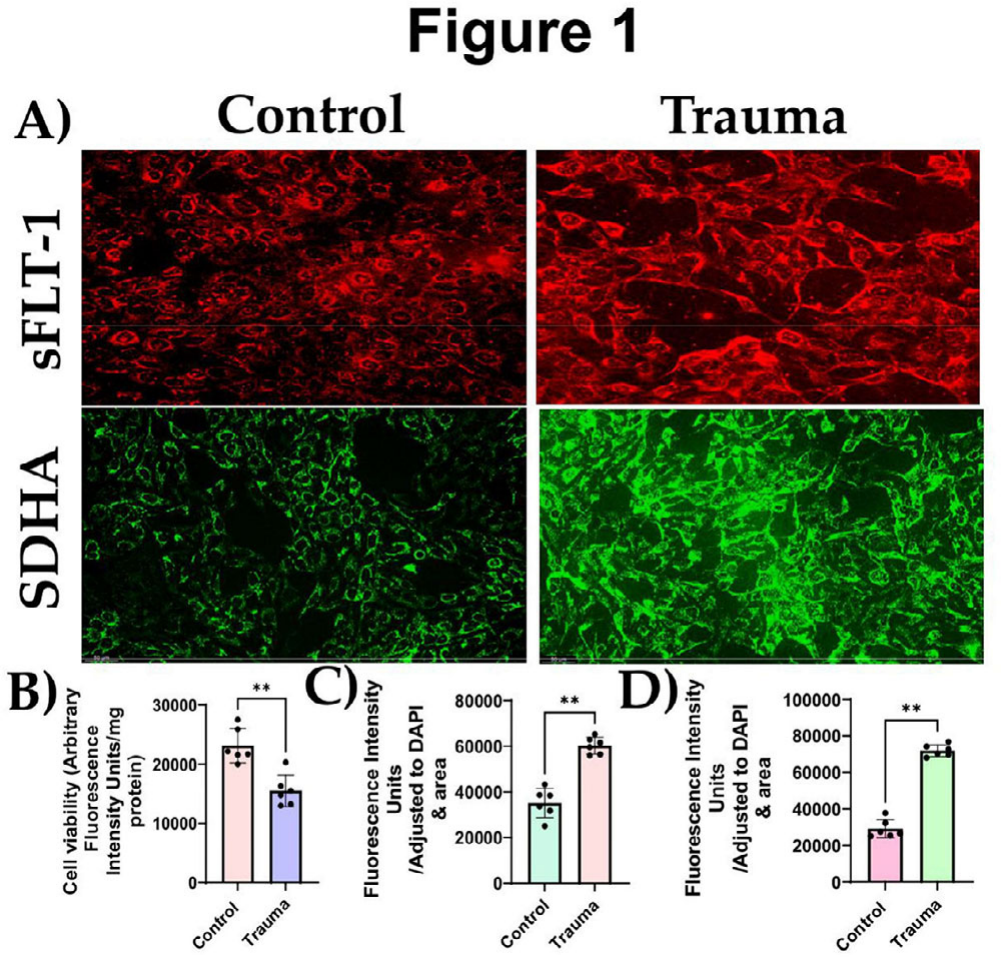

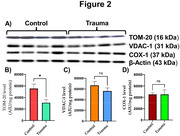

**3:30 PM – 5:00 PM**

## 1185 | **Aspirin Dose and Risk of Postpartum Hemorrhage: A Retrospective Cohort Study**

Michelle DeMeo^1^; Natalia Gontarczyk Uczkowski^2^; Amy Godecker^2^; Janine S. Rhoades^1^; Michael Beninati^1^; Erin Bailey^2^

^1^University of Wisconsin Madison School of Medicine and Public Health, Madison, WI; ^2^University of Wisconsin School of Medicine and Public Health, Department of Obstetrics and Gynecology, Madison, WI

**Objective**: To evaluate whether aspirin at 81 mg or 162 mg daily, compared to no aspirin, is associated with postpartum hemorrhage (PPH) and total blood loss (TBL) in patients receiving aspirin for preeclampsia prevention.

**Study Design**: This retrospective cohort analysis included 30,941 singleton pregnancies delivered ≥ 24 weeks at a single academic tertiary care center 2015 ‐ 2024. Patients were analyzed by aspirin exposure: no aspirin, 81 mg, or 162 mg daily, initiated between 12‐28 weeks. The primary outcome was PPH (blood loss ≥ 1000 mL); secondary outcome was TBL, stratified by mode of delivery. Multivariate logistic and linear regression models adjusting for race, BMI, magnesium sulfate, anticoagulation use, and delivery year to account for prescribing trends were developed.

**Results**: PPH occurred in 27.1% of the no aspirin, 19.2% in the 81 mg, and 15.8% in the 162 mg groups respectively (*p* < 0.001). Adjusted odds of PPH were significantly lower in aspirin groups compared to no aspirin (81 mg: aOR 0.836, CI 0.725‐0.965; 162 mg: aOR 0.779, CI 0.670‐0.960). Stratified analysis showed this effect was limited to vaginal deliveries, where both doses were associated with ∼53% lower odds of PPH (81 mg: aOR 0.462, CI 0.351‐0.608; 162 mg: aOR 0.469, CI 0.353‐0.623). Among cesarean deliveries, no significant association was found (81 mg: aOR 1.17, CI 0.97‐1.40; 162 mg: aOR 0.92, CI 0.75‐1.12). Linear regression showed significantly lower TBL in aspirin groups among vaginal births (*p* < 0.001), but not among cesarean or operative vaginal births.

**Conclusion**: Both aspirin doses were associated with reduced PPH risk and lower TBL among vaginal deliveries. This could be due to aspirin's anti‐inflammatory effects, which may mitigate bleeding risk during labor, where inflammation plays a greater role. This protective effect did not extend to cesarean births, where hemorrhage is likely more affected by surgical factors. Regardless, these findings support the safety of aspirin at both 81 mg and 162 mg and may guide dose selection for preeclampsia prevention.

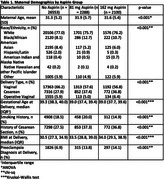

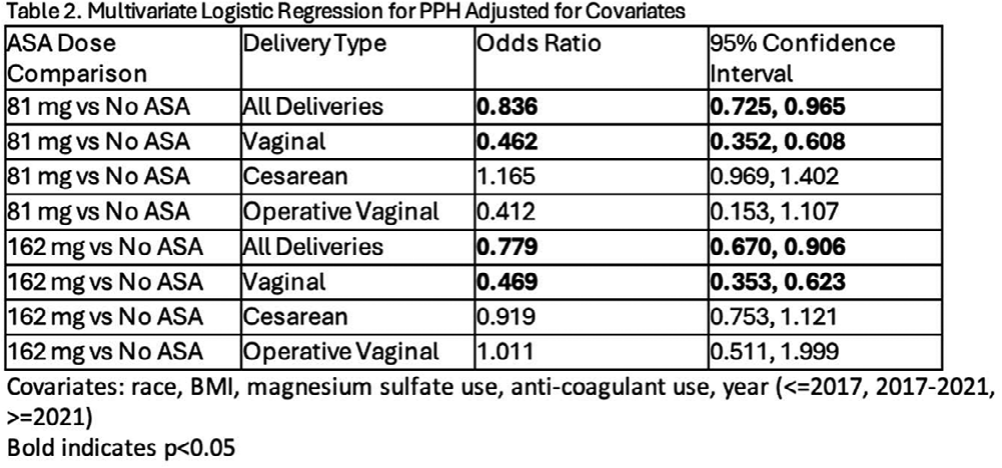

**3:30 PM – 5:00 PM**

## 1186 | **Cesarean Rate Reduction at Scale Is Possible With Intensive Hospital Quality Improvement Support**

Michelle Moniz^1^; Althea Bourdeau^2^; Lisa Kane Low^2^; Jourdan E. Triebwasser^2^; Daniel Almirall^2^; Jessi Ems^2^; Carey Simpson^2^; Paige M. Anderson^3^; Neil Simmerman^4^; Nathalee Harris^5^; Abigail Ramseyer^6^; LeTishia Hill^7^; Molly J. Stout^2^

^1^University of Michigan, University of Michigan, MI; ^2^University of Michigan, Ann Arbor, MI; ^3^University of North Carolina, Charlotte, NC; ^4^Henry Ford Health System, West Bloomfield, MI; ^5^Hurley Medical Center, Flint, MI; ^6^Sparrow Health, Lansing, MI; ^7^Bronson Hospital, Kalamazoo, MI

**Objective**: Large‐scale efforts have had mixed success at reducing the U.S. nulliparous, term, singleton vertex (NTSV) cesarean rate. We evaluated average and hospital‐specific cesarean rate trends during exposure to a “stepped up” quality improvement (QI) support intervention involving BASE (low‐intensity QI support), followed by BASE + LEAD (moderate‐intensity QI support).

**Study Design**: We conducted an observational analysis of NTSV births from 1/2022‐12/2024 in the all‐payer registry of the Obstetrics Initiative (OBI), a statewide quality collaborative funded by Blue Cross Blue Shield of Michigan and Blue Care Network as part of the BCBSM Value Partnerships program. Hospitals with cesarean rates above the Joint Commission's reporting threshold of 30% (*n* = 20) received “BASE,” OBI's standard QI support model (i.e., clinician webinars, monthly hospital performance reports, online toolkit, small financial incentive for hospital participation) in Stage 1 (Month 1‐12), followed by BASE + LEAD, OBI's moderate‐intensity support model (i.e., BASE + consultation between an external QI specialist and maternity unit leaders to develop and execute a site‐customized QI plan) in Stage 2 (Month 13‐24). A generalized linear mixed Poisson model analyzed monthly NTSV cesarean rates during Stage 1 & 2, on average and by hospital.

**Results**: Among *n* = 22,467 NTSV births, the cesarean rate fell 5.0% on average (*p* < 0.001), from 33.4% at month 1 to 31.7% at month 12 to 28.4% at month 24, representing 298 averted cesareans (**Figure 1**). Response to the intervention varied: 18/20 hospitals showed an accelerated cesarean rate decrease in Stage 2 vs. Stage 1, but 2/20 did not, and 5/20 hospitals’ cesarean rates remained above 30% at Month 24, indicating a need for sustained or additional intervention.

**Conclusion**: Cesarean rate reduction is possible at scale using stepped‐up QI support. Future work should identify (i) which hospitals are more or less likely to require augmentation with LEAD at month 12, and (ii) whether additional supports (beyond LEAD) are needed, to achieve, accelerate, or sustain safe cesarean rate reduction.

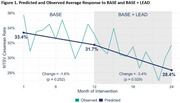

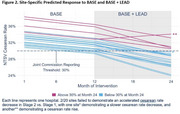

**3:30 PM – 5:00 PM**

## 1187 | **Can we Mitigate Environmental Influences on Relapse Patterns in Pregnant Persons With Substance use Disorder?**

Michelle R. Petrich^1^; Enbal Shacham^2^; Stephen Scroggins^3^; Justine Keller^1^; Sophia Izhar^4^; Sathvika Janga^5^; Maanasa Devabhaktuni^5^; Niraj R. Chavan^6^

^1^Saint Louis University/SSM Health, Saint Louis University/St. Louis, MO; ^2^Saint Louis University/College for Public Health and Social Justice, Saint Louis University/St. Louis, MO; ^3^Saint Louis University/College for Public Health and Social Justice, Saint Louis University/St. Louis, MO; ^4^Saint Louis University/School of Medicine, Saint Louis University School/St. Louis, MO; ^5^Saint Louis University/School of Medicine, Saint Louis University/St. Louis, MO; ^6^Saint Louis University, Saint Louis, MO

**Objective**: We sought to evaluate the impact of social vulnerability on relapse patterns among pregnant persons with substance use disorder (SUD) treated at a comprehensive perinatal SUD program with co‐located obstetric, behavioral health and wrap‐around psychosocial support services.

**Study Design**: We conducted a retrospective cohort study of mother‐infant dyads who received prenatal care at our specialized perinatal SUD center over a 10‐year span from 1/2014 to 3/2023. Given social vulnerability index (SVI) has a relative value, SVI scores were recalculated, per CDC/ATSDR documentation, from raw 2022 5‐Year ACS values and ranked within respective states. Demographic characteristics, substance use behavior and relapse patterns were abstracted from electronic records and compared across subgroups with and without the primary outcome–perinatal (antepartum and/or postpartum) relapse. Significant bivariate associations were determined at the a = 0.05 level using chi‐square test for categorical variables and Mann‐Whitney U test for numeric variables.

**Results**: Of 1047 pregnancies, 831 participants were geocoded across 416 census tracts traversing 2 contiguous states. 517 (49%) patients were noted to have a perinatal relapse. Participants’ SVI score within their respective census tracts is shown in Figure 1. Although prior substance use histories were similar, current behaviors differed: opioid use in conjunction with benzodiazepines (*p* <  .001), stimulants (*p* =  .004), and tobacco (*p* <  .001) as well as current tobacco use (*p* =  .002) were associated with relapse. Patients who engaged in behavioral health counseling (*p* =  .02) and received treatment by a perinatal psychiatrist (*p* <  .001) were less likely to have a relapse (Table 1).

**Conclusion**: Although our population was exposed to varying levels of social vulnerability, our data suggest the influence of environmental stressors on relapse may be mitigated by participation in a comprehensive perinatal SUD program. Addressing determinants of social vulnerability through an integrated healthcare delivery model is critical for establishing perinatal health equity and reducing relapse.

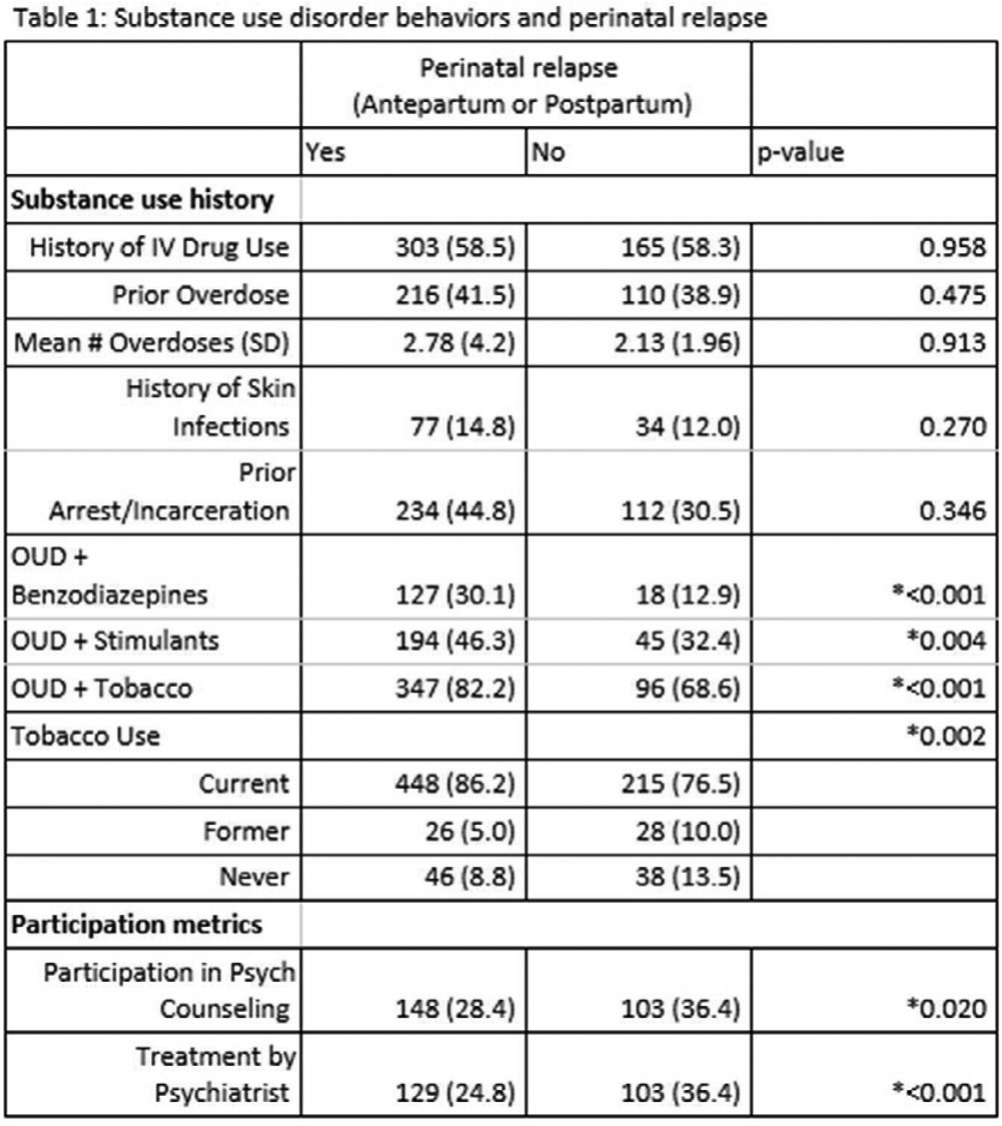

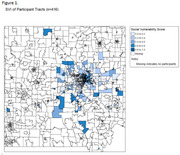

**3:30 PM – 5:00 PM**

## 1188 | **Genotype‐Phenotype Correlations in Prenatal Skeletal Dysplasias**

Michelle J. Wang^1^; Cassandra R. Duffy^1^; Michael H. Duyzend^2^; Ronald J. Wapner^3^

^1^Beth Israel Deaconess Medical Center, Boston, MA; ^2^Boston Children's Hospital, Boston, MA; ^3^Division of Maternal‐Fetal Medicine, Department of Obstetrics and Gynecology, Columbia University Irving Medical Center, New York, NY

**Objective**: While the genetic mechanisms of many skeletal dysplasias are well‐established, the relationship between these mechanisms and phenotype variation is poorly understood. This retrospective case series explores whether dysplasias caused by similar biologic or genomic effects exhibit prenatal phenotypic heterogeneity.

**Study Design**: A retrospective case series of prenatal skeletal dysplasias were identified over three years across five Fetal Sequencing Consortium centers and an additional center. All cases included had prenatal duo or trio genome sequencing and resulted in a live birth.  Data abstracted included fetal biometric measurements [femur length (FL), abdominal circumference (AC), thoracic circumference (TC)], additional imaging studies, and genetic results.

**Results**: Eleven cases were identified and listed in descending  FL Z‐scores in Table 1.  Figure 1 demonstrates the ultrasound findings based on the underlying gene function. Long bone changes (FL/AC< 0.16, bowing, fractures), often the first feature of skeletal dysplasia detected on ultrasound, were seen across all gene functions. Thoracic findings (TC/AC< 0.83, pulmonary hypoplasia, short ribs), associated with more severe disease, were seen in variants in fibroblast growth factor receptor signaling (FGFR1, FGFR3) and collagen synthesis (COL1A1, COL1A2).  Ciliary signaling pathway gene variants (ECV2, KIAA0586, DYNC2H1) were associated with more non‐skeletal anomalies. All variants affecting cartilage formation (CSGALNACT1, ACAN) and DNA repair (POLE) had milder phenotypes.

**Conclusion**: There is significant phenotypic variation among skeletal dysplasias when stratified by genetic mechanisms and fetal phenotype alone appears to be poorly predictive of underlying mechanisms. Exome or genome sequencing should be considered for suspected skeletal dysplasias due to the phenotypic and molecular heterogeneity of these conditions. Future studies should incorporate functional analyses to elucidate the molecular mechanisms driving severe congenital skeletal disease, especially as in‐utero fetal therapies are developed.

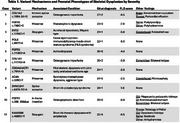

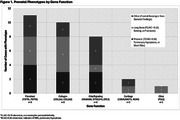

**3:30 PM – 5:00 PM**

## 1189 | **What's Trending on #PostpartumHypertension on TikTok ‐ a Descriptive Study**

Minhazur R. Sarker^1^; Emma Jacobs^2^; Rachel L. Wiley^1^; Ukachi N. Emeruwa^3^

^1^Division of Maternal Fetal Medicine, Department of Obstetrics, Gynecology and Reproductive Sciences, University of California San Diego, San Diego, CA; ^2^Department of Obstetrics, Gynecology and Reproductive Sciences, University of California San Diego, San Diego, CA; ^3^University of California San Diego, San Diego, CA

**Objective**: Postpartum hypertension accounts for a significant proportion of hypertension‐related deaths, highlighting the importance of understanding the community perception of this pathology. We aimed to evaluate content using #PostpartumHypertension (#pphtn) on TikTok.

**Study Design**: This was a cross‐sectional study of the top 100 English videos by play count using #pphtn on TikTok. Videos were generated using the TikTok Data Extractor by Apify. The primary outcome was the number of videos relevant to the hashtag. The secondary outcomes included pre‐specified themes (e.g. declining interventions, distrusting providers, seeking advice/community). A Global Quality Score (GQS) for each video was assigned; GCS is a validated tool rating medical content from 1 (low, patient use discouraged) to 5 (high, medically useful). Medical claims content was evaluated using mDISCERN–a validated scale to assess the accuracy of medical information. Additional metrics were collected for video characteristics.

**Results**: Among the top 100 videos using #pphtn, 67 were relevant, suggesting the hashtag is not appropriately utilized. Most creators were women, located in the United States, and without available credentials, suggesting likely lay‐individuals (Table 1). With respect to video type, most were personal experience or in the “other” category (Table 2). The median [interquartile range] GQS was 1 [1,2] suggesting that these videos were typically of limited medial usefulness (Table 2). Only 5 (7.5%) videos made medical claims of which 2 were evidence based and 3 were not. While 65.7% of videos expressed themes of fear, almost all videos had a supportive comments section, suggesting a caring community within this hashtag. Additional characteristics are noted in Table 2.

**Conclusion**: Our findings suggest that #PostpartumHypertension TikTok content is of low usefulness. Despite a supportive community, there is a consistent theme of fear and minimal healthcare provider presence. Creation of higher quality, evidence‐based content on postpartum hypertension may improve the tone of the community.

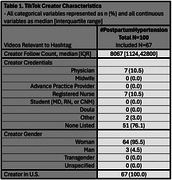

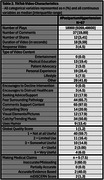

**3:30 PM – 5:00 PM**

## 1190 | **Placental CD20+B Cells From Preeclamptic Women Contribute to Neurovascular Dysfunction in Rat Model of PE**

Miriam Hankins; Nathan Campbell; Jane J. Border; Murphy Lemon; E. Hawthorne Cleveland; Baoying Zheng; Alexandra Demesa; Evangeline Deer; Rachael Morris; Richard R. Roman; Babbette LaMarca

University of Mississippi Medical Center, Jackson, MS

**Objective**: Preeclampsia (PE), new‐onset hypertension in pregnancy, is associated with immune activation, B cells secreting agonistic autoantibodies to the angiotensin II type 1 receptor (AT1‐AA), and neurological complications. We have shown AT1‐AA to contribute to neurovascular dysfunction during pregnancy and we know that it is produced postpartum up to 7 years, suggesting a memory B cell immune response. Therefore, we hypothesize that CD20+ B cells cause hypertension and neurovascular dysfunction that allows for transmission of high blood pressure to the brain via AT1‐AA.

**Study Design**: Three hundred thousand placental CD20+ B Cells from normal pregnant (NP) or PE patients were isolated at delivery and injected i.p. into nude athymic rats on gestational day (GD) 12. Mini‐osmotic pumps containing the AT1‐AA inhibitor peptide, ‘n7AAc’, were inserted on GD14. Between GD14 and GD19 memory and learning were assessed with a Barnes maze.  On GD19, blood pressure (MAP) and cerebral blood flow (CBF) autoregulation were measured with laser doppler flowmetry (LDF). A one‐way ANOVA was used for statistical analysis.

**Results**: MAP increased in PE B cell recipients (123±2 mmHg, *n* = 8) compared to NP recipients (106±3 mmHg, *n* = 11). MAP was lowered with ‘n7AAc’ to 110±3 mmHg (*n* = 6). AT1‐AA activity was elevated with PE B cells to 14±2 ΔBPM, *n* = 6 compared to NP recipients (2±1 ΔBPM, *n* = 5) and ‘n7AAc’ treated PE recipients (1±2 ΔBPM, *n* = 6). Baseline CBF was significantly higher in PE recipients (569±63 LDF, *n* = 3) compared to NP recipients (252±13 LDF, *n* = 3) or ‘n7AAc’ treated PE recipients (317±58 LDF, *n* = 3). CBF autoregulation was improved in ‘n7AAc’ treated PE recipients (43±4% increased transmission, *n* = 3) compared to PE recipients (66±10% increased transmission, *n* = 3). Over five days, PE recipients were slower to escape the Barnes maze than NP B cell recipients or ‘n7AAc’ treated PE B cell recipients.

**Conclusion**: CD20+ B cells from PE women contribute to hypertension, AT1‐AA and neurovascular and cognitive dysfunction during pregnancy supporting a role for B cells producing AT1‐AA in the pathophysiology of PE.

**3:30 PM – 5:00 PM**

## 1191 | **Growth Velocity vs Doppler Flow: Rethinking Surveillance in Growth‐Restricted Fetuses**

Mohammad Salameh^1^; Joshua Brunton^2^; Megan E. Branda^1^; Kylie Cooper^1^; Regan Theiler^1^

^1^Mayo Clinic, Rochester, MN; ^2^Virginia Commonwealth University, Richmond, VA

**Objective**: Current delivery recommendations for growth‐restricted fetuses rely heavily on umbilical artery (UA) Doppler indices, which are well validated in cases with absent or reversed end‐diastolic flow. However, management of fetuses with preserved forward flow remains largely guided by expert opinion. Growth velocity may be an important tool in growth restricted fetuses. We aimed to compare the predictive performance of growth velocity with UA systolic/diastolic (S/D) ratio in identifying adverse outcomes.

**Study Design**: This retrospective cohort study included pregnant patients aged 18–45 who delivered between 2012–2022. Exclusions included congenital anomalies and multiple gestations. Growth velocity was calculated as the weekly change in estimated fetal weight percentile between 26–32 and 32–36 weeks. The final UA S/D ratio was used. The primary outcome was a composite of neonatal morbidity and severe adverse perinatal events. Secondary outcomes included neonatal morbidity events, severe adverse perinatal events, neonatal intensive care unit admission, and respiratory complications. Univariate logistic regression and receiver operator curve analyses were used.

**Results**: Of 324 patients with growth velocity data, 189 had UA Doppler data. Among them, 29 (15.3%) had an S/D ratio >95th percentile; none had absent or reversed end‐diastolic flow. Baseline characteristics were similar across groups. There was no significant correlation between growth velocity and S/D ratio (r = ‐0.05, *p* = 0.50). The area under the curve (AUC) for the S/D ratio in predicting the primary outcome was 0.48 (95% CI: 0.39‐0.57), while growth velocity showed improved discrimination with an AUC of 0.61 (95% CI: 0.53‐0.70) for growth velocity only and 0.62 (95% CI: 0.55–0.68) for the full cohort; see Figure 1. A similar pattern was observed across secondary outcomes.

**Conclusion**: In growth‐restricted fetuses with forward UA flow, growth velocity appears to outperform the UA S/D ratio in predicting adverse outcomes. These findings support growth velocity as a valuable tool to enhance fetal risk stratification and guide delivery decisions.

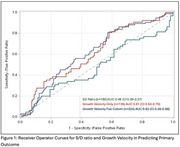

**3:30 PM – 5:00 PM**

## 1192 | **Postpartum Readmissions and Associated Complications**

Monica H. Rodriguez^1^; Robert Boccaccio^1^; Justin Solomon^2^; Michele L. McCarroll^3^

^1^Medical City Arlington, Arlington, TX; ^2^Graduate Medical Education, Arlington, TX; ^3^Medical City Arlington, Medical City Arlington, TX

**Objective**: Despite advances in obstetric care, the postpartum period remains a critical phase requiring improvement. This study aims to evaluate the incidence, timing, and predictors of postpartum hospital readmissions within 30 days of delivery.

**Study Design**: This retrospective study evaluated postpartum readmission data across Texas HCA Healthcare hospitals using Clinical Classifications Software Refined (CCSR) codes, which aggregate International Classification of Diseases, 10th Revision (ICD‐10) diagnosis codes. Statistical analyses were performed using SPSS v28.0.

**Results**: Among 169,231 total deliveries, 56,012 were excluded due to missing data or other criteria, yielding a final sample of 113,219 cases. Among these, 13,334 patients were readmitted within 30 days postpartum. Average age was 29.5 years (±5.9). Delivery types were 52.1% vaginal and 47.9% cesarean, with average estimated blood loss of 122.1 mL (±271.7). Among 30‐day readmissions, 62.3% were due to pregnancy‐related conditions (PRC), 8.3% to genitourinary disorders, and 9.1% to ill‐defined symptoms. Within PRC, the most common diagnoses were other specified puerperal complications (14.1%), severe/unspecified pre‐eclampsia (9.8%), and postpartum hemorrhage (3.1%). Average time to readmission was 9 days (±7.9). Binary logistic regression identified significant predictors of readmission (all *p* <  .001): Black race (OR = 1.3672, 95% CI: 1.2741–1.4670,); obesity (OR = 1.2798), psychiatric diagnosis (OR = 1.3351), diabetes (OR = 1.1295), and hypertensive disorders (OR = 1.7622).

**Conclusion**: Postpartum complications requiring readmission can disrupt early mother‐infant bonding and contribute to increased maternal morbidity. This study highlights racial disparities and influence of comorbid conditions in high‐risk populations, showing that upstream prenatal intervention can directly affect downstream outcomes. Findings also support ACOG guidance on early postpartum follow‐up within three weeks, a critical time to reduce maternal readmission, improving postpartum outcomes.

**3:30 PM – 5:00 PM**

## 1193 | **Iron Deficiency Anemia in Pregnancy and Perinatal Outcomes**

Monica Sosa^1^; Brett D. Einerson^2^; Rupam Das^1^; Muyuan Zhang^3^; JJ Horns^1^; Ann M. Bruno^1^

^1^University of Utah, Salt Lake City, UT; ^2^Department of Obstetrics and Gynecology, University of Utah Health, Salt Lake City, UT; ^3^University of Utah, University of Utah, UT

**Objective**: The U.S. Preventative Services Task Force identifies insufficient evidence to make recommendations on screening and treatment of iron deficiency anemia (IDA) in pregnancy. We aimed to evaluate the association between IDA in pregnancy and postpartum and perinatal outcomes in a modern obstetric cohort.

**Study Design**: Repeated cross‐sectional analysis of individuals aged 13‐50 years with pregnancy and delivery data at > 20 weeks’ gestation available in the MarketScan Commercial Insurance Research Database from 2011‐2021. The exposure was IDA, identified by International Classification of Diseases insurance claims codes. Maternal and neonatal outcomes included severe maternal morbidity, postpartum hemorrhage, postpartum depression, blood transfusion, neonatal IDA and NICU admission. Baseline characteristics were compared between those with and without IDA in pregnancy or through 6 weeks postpartum. Multivariable logistic regression models estimated the association between IDA and outcomes. A Cox proportional hazard model estimated the association between IDA and neonatal IDA.

**Results**: Of 1,713,293 individuals included, 110,719 (6.5%) had IDA. Individuals with IDA were more likely to be older and have a comorbid health condition (Table 1). Maternal IDA compared with no IDA was associated with increased odds of severe maternal morbidity (4.85% vs 0.1%, aOR 50.2, 95% CI 47.4–53.1), postpartum hemorrhage (3.81% vs 0.08%, aOR 52.5, 95% CI 49.3–56.0), postpartum depression (3.90% vs 0.09%, aOR 42.8, 95% CI 40.3–45.4), and blood transfusion (2.06% vs 0.03%, aOR 76.5, 95% CI 69.0–84.9; Table 2). Maternal IDA was associated with an increased risk of neonatal iron deficiency anemia (42.37% vs 0.34%  HR 168.6, 95% CI 163.8‐173.4).

**Conclusion**: Consistent with prior studies, we found maternal IDA in a modern national cohort was strongly associated with adverse maternal and neonatal outcomes. Additional research is needed to inform national guidelines on IDA screening and treatment in pregnancy.

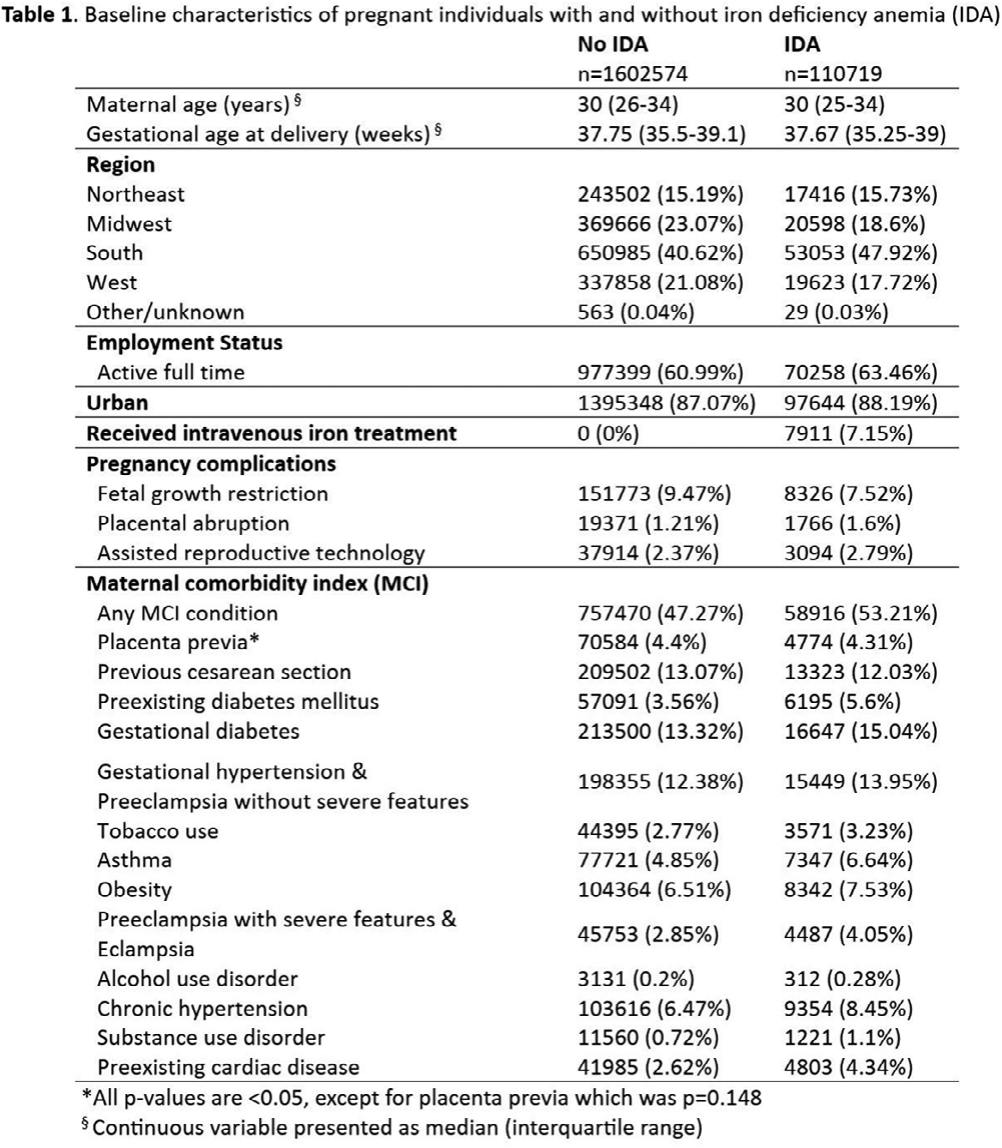

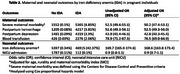

**3:30 PM – 5:00 PM**

## 1194 | **Impact of Zygosity on Perinatal Outcomes in Dichorionic Diamniotic Twin Pregnancies**

Morgan Steelman^1^; Johanna A. Suskin^2^; Erica Glaser^2^; Annika Hsu^3^; Lois Brustman^2^; Jennifer Lam‐Rachlin^4^; Andrei Rebarber^5^; Nathan S. Fox^2^; Simi Gupta^2^

^1^Icahn School of Medicine at Mount Sinai Hospital, Icahn School of Medicine at Mount Sinai Hospital, NY; ^2^Icahn School of Medicine, Mount Sinai West, New York, NY; ^3^Mount Sinai, New York, NY; ^4^Icahn School of Medicine at Mount Sinai, Icahn School of Medicine at Mount Sinai, NY; ^5^Icahn School of Medicine, Mount Sinai West, Mount Sinai West, NY

**Objective**: Single Nucleotide Polymorphism (SNP) Noninvasive prenatal testing (NIPT) allows determination of zygosity in twin pregnancies. Prior studies used NIPT to explore associations between monozygosity and adverse neonatal outcomes regardless of chorionicity. Our aim was to compare perinatal outcomes between SNP based NIPT‐confirmed monozygotic and dizygotic twins in only dichorionic diamniotic (DCDA) twin pregnancies.

**Study Design**: We conducted a single‐center, IRB‐approved retrospective cohort study of DCDA twin pregnancies with NIPT results between 2021 and 2025. Patients were excluded if data were missing for zygosity or outcomes of interest. Baseline characteristics were compared by zygosity using t‐tests or chi‐square/Fisher's exact tests, as appropriate. Logistic regression models estimated the association between zygosity and perinatal outcomes, with odds ratios adjusted for maternal age and mode of conception.

**Results**: Of the 60 patients included, 10 had monozygotic pregnancies (16.7%) and 50 had dizygotic pregnancies (83.3%). The odds of congenital anomaly were 7.90 times higher in monozygotic pregnancies compared to dizygotic pregnancies (95% CI, 1.43–43.55). The odds of genetic anomaly were 18.86 times higher in monozygotic pregnancies (95% CI, 1.56–228.46). No neonatal deaths or miscarriages were observed in either group. There were no statistically significant differences in rates of preterm birth, NICU admission, or other perinatal outcomes.

**Conclusion**: Monozygotic DCDA twin pregnancies were associated with significantly higher odds of congenital and genetic anomalies compared to dizygotic twins. It is unclear at this time whether monozygotic twinning increases the risk of genetic anomalies or whether preexisting genetic abnormalities may predispose embryos to monozygotic splitting. These findings underscore the importance of utilization of SNP‐based NIPT testing to determine zygosity of DCDA twin pregnancies. Monozygotic DCDA twins may benefit from the routine offer of diagnostic testing and detailed anatomic screening.

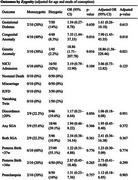

**3:30 PM – 5:00 PM**

## 1195 | **Prior Preeclampsia or Chronic Hypertension Present Distinct Molecular Subtypes in Hypertensive Disorders of Pregnancy**

Morten Rasmussen^1^; Elaine P. Gee^1^; Rupsa Boelig^2^; Daniel Kiefer^3^; Pamela Simmons^4^; George R. Saade^5^; Antonio F. Saad^6^; Ebony Carter^7^; Vincenzo Berghella^8^; Ai‐ris Y. Collier^9^; Antonina I. Frolova^10^; Esther Park‐Hwang^11^; Luis D. Pacheco^12^; Elizabeth F. Sutton^4^; Kara M. Rood^13^; Joseph R. Biggio, Jr.^14^; Cynthia Gyamfi‐Bannerman^15^; Arun Jeyabalan^16^; Michal Elovitz^17^; Carrie Haverty^1^; Thomas McElrath^1^; Alison Moe^1^

^1^Mirvie Inc, South San Francisco, CA; ^2^Thomas Jefferson University, Thomas Jefferson University, PA; ^3^Celebration OB/GYN, Orlando, FL; ^4^Woman's Hospital, Baton Rouge, LA; ^5^Department of Obstetrics and Gynecology, Eastern Virginia Medical School at Old Dominion University, Norfolk, VA; ^6^Inova Fairfax Hospital, Falls Church, VA; ^7^University of North Carolina at Chapel Hill, Chapel Hill, NC; ^8^Thomas Jefferson University, Philadelphia, PA; ^9^Beth Israel Deaconess Medical Center, Boston, MA; ^10^Washington University School of Medicine, St. Louis, MO; ^11^Multicare Health System, Tacoma, WA; ^12^University of Texas Medical Branch, Galveston, TX; ^13^The Ohio State University, Columbus, OH; ^14^Ochsner Health, New Orleans, LA; ^15^Division of Maternal Fetal Medicine, Department of Obstetrics, Gynecology and Reproductive Sciences, University of California San Diego, San Diego, CA; ^16^UPMC Magee‐Womens Hospital, Pittsburgh, PA; ^17^Nuttall Women's Health, Philadelphia, PA

**Objective**: Hypertensive disorders of pregnancy (HDP) are major causes of maternal and fetal morbidity and mortality. Previously, elevated *PAPPA2* expression in cfRNA was identified as predictive of preeclampsia (PE) in a dose‐dependent manner, with an exceptionally strong effect size among individuals without pre‐existing high risk factors. Here we evaluate *PAPPA2* expression based on HDP and gestational age (GA) at delivery in those with chronic hypertension (CHTN) or prior PE.

**Study Design**: Whole blood and medical information were collected from a diverse group of 5399 study participants collected across 11 clinical sites and direct‐to‐participant recruitment. Spontaneous preterm births were excluded. cfRNA was extracted and analyzed by sequencing to generate gene counts. Robust linear regression evaluated *PAPPA2* dependence on delivery GA by preterm PE status.

**Results**: In individuals with chronic hypertension there was no significant correlation between *PAPPA2* expression level and gestational age at delivery regardless of the occurrence of either preterm PE (*n* = 27, R^2^ = 2.8%, *p* = 0.4, Fig1) or a pregnancy where preterm PE did not occur (*n* = 281, R^2^ = 0%, *p* = 0.85, Fig1). However in individuals with a history of PE, a significant correlation with *PAPPA2* was observed in those who developed preterm PE (*n* = 29, R^2^ = 16.6%, *p* = 0.028, Fig1), but in those who did not develop preterm PE the relationship was not significant (*n* = 249, R^2^ = 1.1%, *p* = 0.09, Fig1). This is similar to the previously reported effect among individuals without independent high risk conditions who develop preterm PE (*n* = 87, R^2^ = 12.2%, *p* < 0.001, Fig1).

**Conclusion**: The molecular patterns observed for *PAPPA2* in individuals without pre‐existing risk is replicated in individuals with prior PE who go on to have repeated PE. The molecular pattern is different in superimposed PE where no elevation in *PAPPA2* was observed. These findings support multiple etiologies of HDP and the potential for personalized treatments paths in the future.

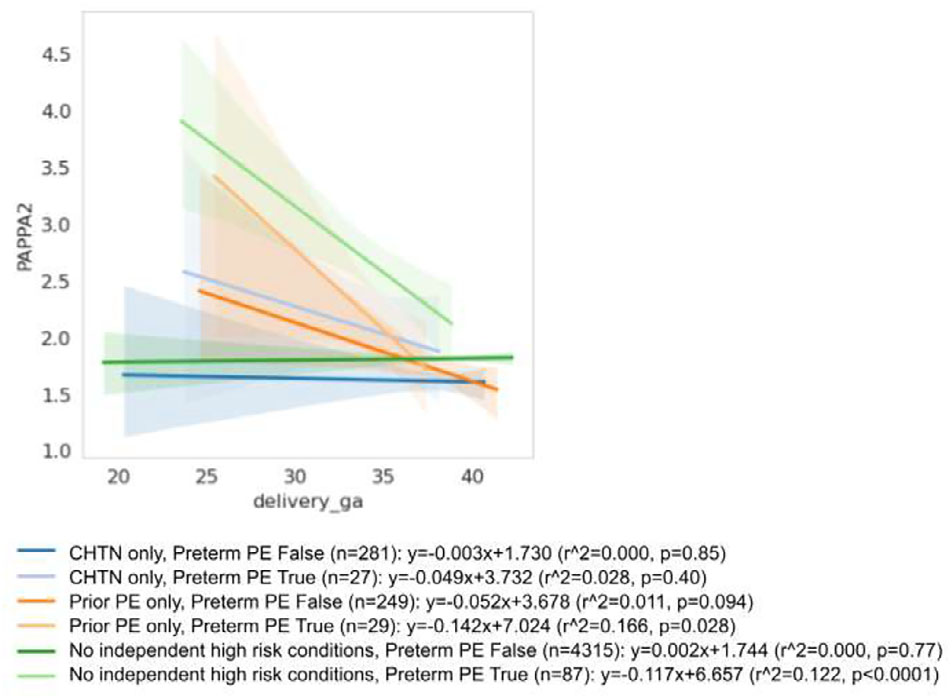

**3:30 PM – 5:00 PM**

## 1196 | **Post‐ARRIVE Trial Surge in 39 and 40‐Week Inductions Among Low‐Risk Nulliparous Patients Nationwide**

Moti Gulersen^1^; Itai Hazan^2^; Erez Lenchner^3^; Marlee Hirsch^4^; Ashley Zimmermann^5^; Vincenzo Berghella^6^; Eran Bornstein^5^

^1^Thomas Jefferson University, PHILADELPHIA, PA; ^2^Ben‐Gurion University of the Negev, Beer Sheva, HaDarom; ^3^New York University Rory Meyers College of Nursing, New York, NY; ^4^Northwell, New York, NY; ^5^Northwell, New Hyde Park, NY; ^6^Thomas Jefferson University, Philadelphia, PA

**Objective**: To assess temporal trends in labor induction (IOL) at term among low‐risk nulliparous patients following the publication of the ARRIVE trial in August, 2018.

**Study Design**: Retrospective analysis of the Centers for Disease Control and Prevention Natality Live Birth database (2016‐2023). This low‐risk cohort included nulliparous, term (≥37 0/7 weeks of gestation), singleton, vertex (NTSV), in‐hospital births. Pregnancies with fetal congenital/chromosomal anomalies, pregestational and gestational diabetes, and hypertensive disorders were excluded. We calculated annual IOL rates for each gestational week and conducted an interrupted time series (ITS) analysis for births between August 2016 and August 2021, adjusting for seasonality using harmonic terms. Subgroup analyses by gestational age, maternal age, body mass index, race and ethnicity, and insurance type were performed.

**Results**: Of the 6,665,202 births included, IOL occurred in 2,262,239 (33.9%). The rate of IOL at 39 weeks has been rising steadily over the study period, with the most striking increase noted in the first 2.5 years post‐ARRIVE (Figure 1). ITS analysis showed a significant immediate level increase at 39 weeks (IRR  =  1.09; 95% CI: 1.06–1.13; *p*  < 0.001), along with a significant acceleration in monthly trend (*p* < 0.001). Overall term IOL rates increased from 28.3% in 2016 to 38.4% and in 2023. In addition, these trends were noted in several patient characteristics evaluated (Figure 2).

**Conclusion**: Although rates of IOL in low‐risk NTSV have been on the rise throughout the past decade, the most striking increase occurred in the 2.5 years immediately post ARRIVE trial publication, particularly among patients at 39 and 40 weeks of gestation. These findings suggest real‐world adoption of ARRIVE‐based IOL practices nationwide. Furthermore, similar practice patterns were noted across all patient characteristics evaluated.

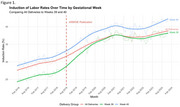

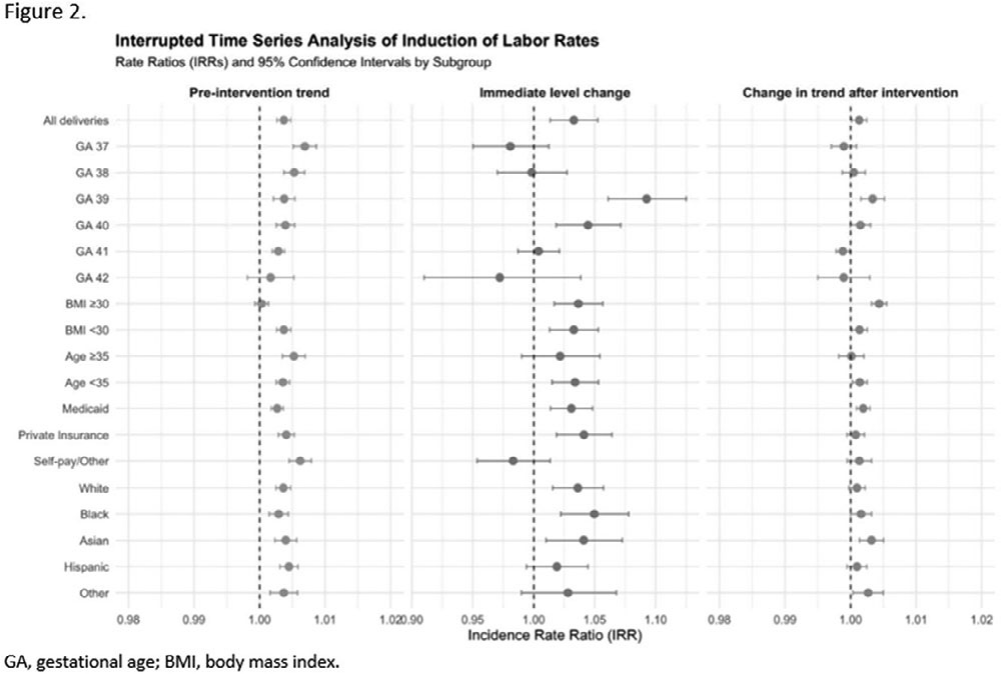

**3:30 PM – 5:00 PM**

## 1197 | **Epigenetic Variations Associated With Smoking in Pregnant Individuals With Chronic Hypertension**

Mudit Vaid^1^; Amit Patki^2^; Jeff M. Szychowski^2^; Hemant Tiwari^2^; Alana Jones^3^; Javier Velez^4^; Ryan Ryan Irvin^3^; Alan T. Tita^5^; Akila Subramaniam^5^; Bertha A. Hidalgo^3^

^1^Department of Obstetrics and Gynecology, University of Alabama at Birmingham, Birmingham, AL; ^2^Department of Biostatistics, University of Alabama at Birmingham, Birmingham, AL; ^3^Department of Epidemiology, University of Alabama at Birmingham, Birmingham, AL; ^4^Heersink School of Medicine, University of Alabama at Birmingham, Birmingham, AL; ^5^Center for Research in Women's Health, University of Alabama at Birmingham, Birmingham, AL

**Objective**: Smoking‐associated DNA methylation is well‐characterized in the general population, but data in pregnancy are sparse. Given the increased risk of hypertensive disorders of pregnancy (HDP) in women with chronic hypertension (CHTN), and paradoxically decreased rates of HDP in smokers, we aimed to investigate smoking‐induced epigenetic changes in gravidas with CHTN.

**Study Design**: We performed a secondary analysis of the CHAP Epigenetics Study. Blood samples collected at time of randomization from the original CHAP trial comparing antihypertensive treatment vs. non‐treatment in pregnant women with mild CHTN at <23 weeks’ gestation were utilized. Baseline genomic DNA was profiled for methylation levels using the Illumina EPIC v2.0 array. Participants were categorized as smokers or non‐smokers based on self‐report. Epigenome‐wide association study (EWAS) analyses were performed using linear regression models adjusted for age, treatment group, ancestry, cell proportions, BMI, and alcohol use to identify cytosine‐phosphate‐guanine (CpGs) sites associated with smoking. Differentially methylated regions (DMRs) for smoking were also identified using the coMethDMR package in R.

**Results**: 1392 individuals [157 (11.3%) smokers and 1235 non‐smokers] were analyzed (Table 1). We identified **149 CpG sites** significantly associated with smoking (*p* < 4.91×10^−^⁸). Of these, 84 mapped to 55 genes enriched in known smoking‐related loci (*AHRR, GFI1, MYO1G, PRSS23*); 99 (∼66%) were previously reported, while 50 were novel (Table 2). Additionally, we identified 22 smoking‐related DMRs (FDR< 0.009), each comprising 3–10 CpGs in close genomic proximity. Four CpGs from the DMR analysis overlapped with the EWAS (cg12303084, cg05445320, cg11427534, cg09662411), indicating greater biological relevance.

**Conclusion**: Smoking in pregnant women with mild CHTN is associated with distinct methylation signatures. These findings represent potential biomarkers to understand the relationships between smoking, CHTN, and HDP and may inform future interventions for mitigating adverse pregnancy outcomes in this high‐risk group.

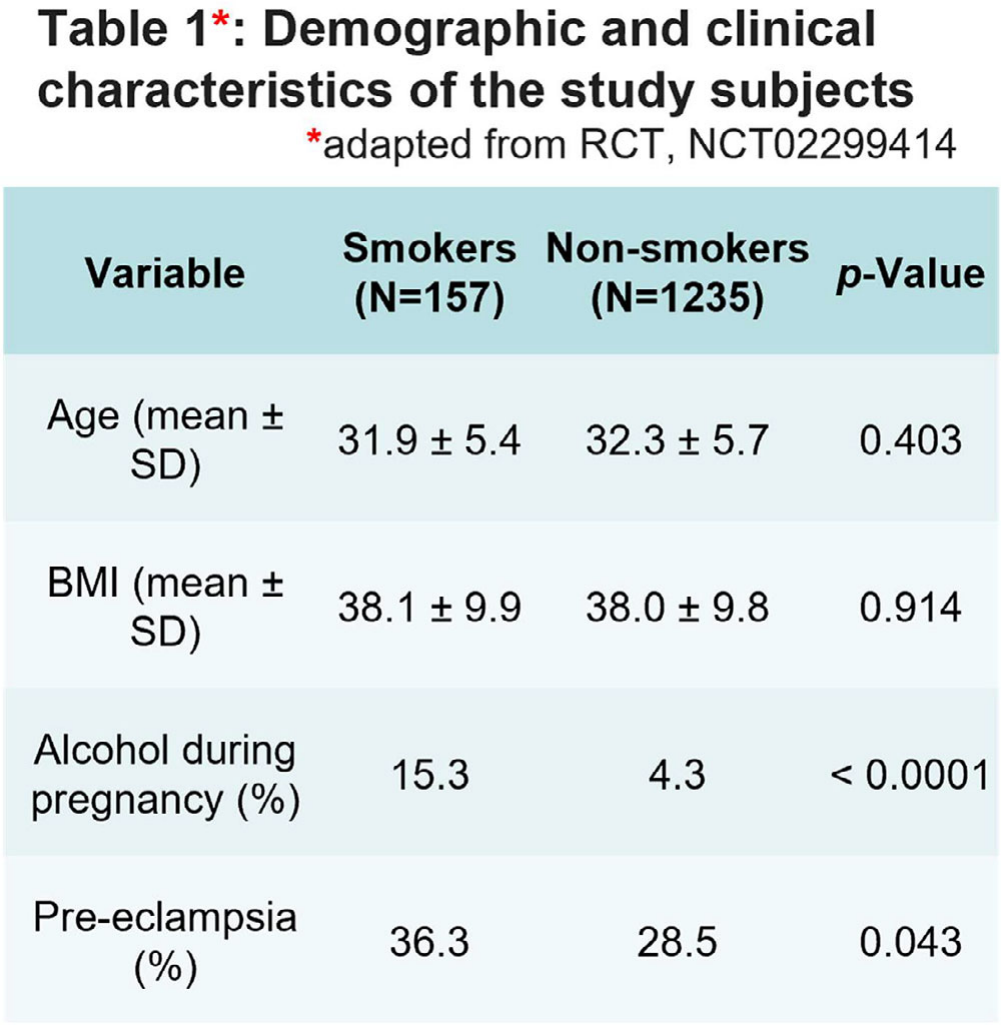

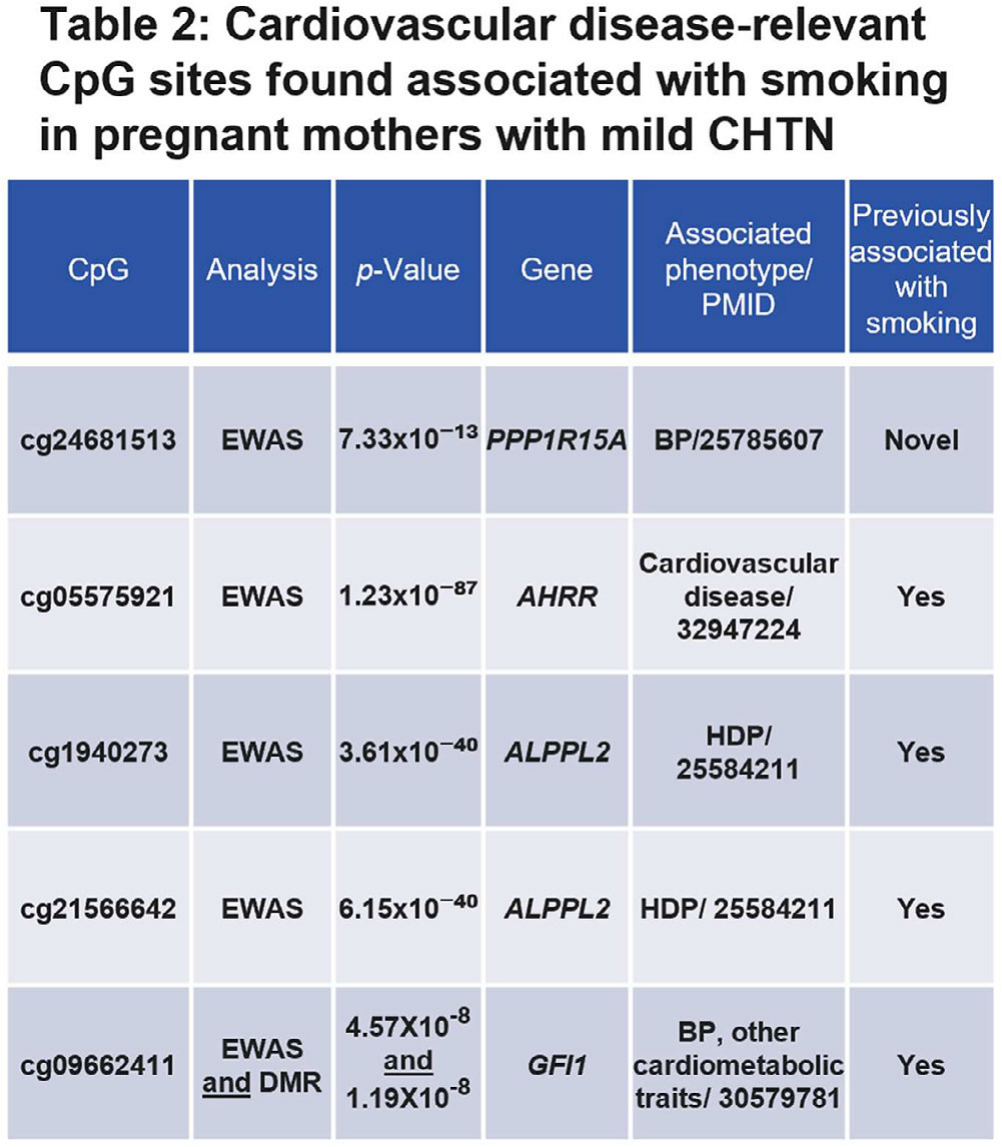

**3:30 PM – 5:00 PM**

## 1198 | **Toxicity Associated With Maternal Antiarrhythmic Therapy for Treatment of Fetal Tachyarrhythmias: A Case Series**

Mugdha V. Mokashi^1^; Samanvi Kanugula^2^; Lynn M. Yee^2^; Stephanie A. Fisher^2^

^1^Northwestern Medical Center, Chicago, IL; ^2^Northwestern University Feinberg School of Medicine, Chicago, IL

**Objective**: To evaluate maternal toxicities associated with oral antiarrhythmic therapy for management of fetal tachyarrhythmias.

**Study Design**: We conducted a retrospective case series of pregnant people with a fetal tachyarrhythmia diagnosis treated at a large academic medical center between January 2010 and September 2022. We reviewed cases of singleton gestations with an obstetric ultrasound or fetal echocardiography confirming a diagnosis of fetal tachyarrhythmia and who delivered ≥24 weeks’ gestation. Data on maternal oral antiarrhythmic treatment (digoxin, flecainide, sotalol, and/or amiodarone), side effects, monitoring for potential drug toxicity (i.e. electrocardiograms, laboratory surveillance), and neonatal outcomes were abstracted from medical records.

**Results**: Of 42 patients found to have fetal tachyarrhythmia, 22 (52.3%) received maternal antiarrhythmic therapy. The majority had fetal supraventricular tachycardia (54.5%), 9 had fetal atrial flutter (40.9%), and 1 (4.5%) had non‐sustained fetal atrial tachycardia. Of these, nearly half (*N* = 10) patients developed maternal side effects with concern for drug toxicity requiring therapy modification. The most commonly reported side effects were severe nausea and emesis (*N* = 8). Several patients had maternal electrocardiogram changes (*N* = 5), including AV block and T wave abnormalities, attributed to digoxin; one patient had QRS prolongation secondary to flecainide. Less common therapy‐altering side effects included lethargy, presyncope, visual hallucinations, and transaminitis (Figure).

**Conclusion**: Although maternal treatment of fetal tachyarrhythmias is often indicated for fetal benefit, in this cohort, 45% of individuals required alteration of therapy due to concern for maternal toxicity. Our findings emphasize the need for standardized maternal monitoring guidelines and prompt therapeutic adjustments to balance fetal treatment efficacy with maternal safety.

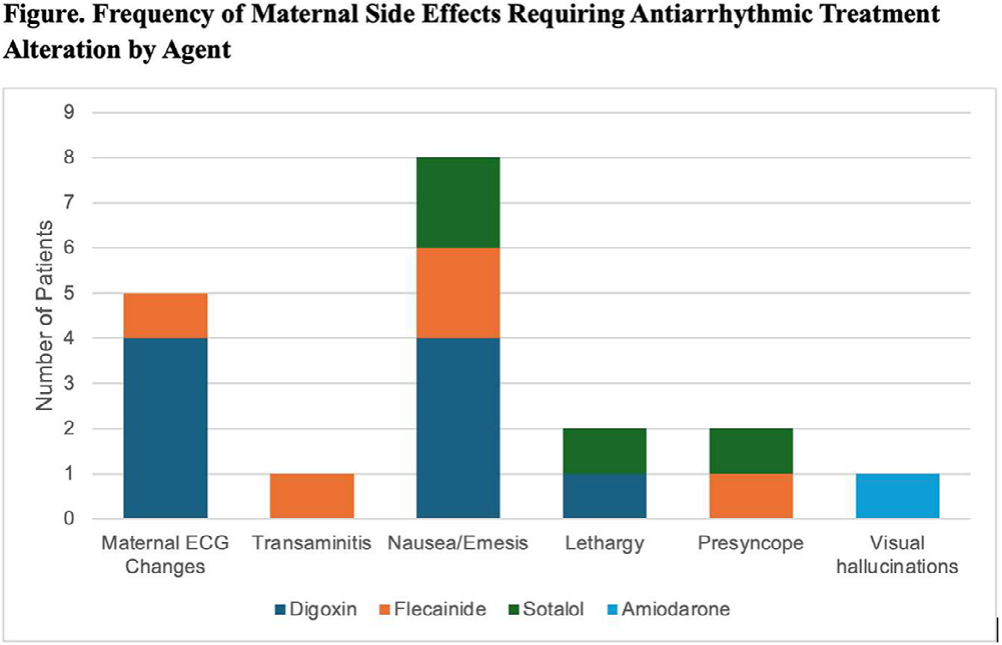

**3:30 PM – 5:00 PM**

## 1199 | **cfDNA and Adverse Outcomes With Severe Fetal Growth Restriction**

Nasim C. Sobhani^1^; Franchesca Liao^2^; Sucheta Bhatt^2^; Kan Nobuta^2^; Louise C. Laurent^3^; Mary E. Norton^4^

^1^UCSF, UCSF/San Francisco, CA; ^2^Illumina Inc, Illumina Inc, CA; ^3^UCSD, UCSD, CA; ^4^University of California, San Francisco, San Francisco, CA

**Objective**: In many cases of fetal growth restriction (FGR), the underlying cause is unknown. Confined placental mosaicism (CPM), including of rare autosomal trisomies (RAT), is reported to account for up to10% of cases. This study examined the utility of prenatal cell‐free DNA (cfDNA) screening via whole‐genome sequencing (WGS) to identify CPM of RAT at diagnosis of unexplained severe early onset FGR. We also assessed the association of total cfDNA concentration and fetal fraction with FGR‐related adverse outcomes.

**Study Design**: This prospective, multicenter study enrolled singletons with unexplained severe FGR <34 weeks, defined as estimated fetal weight (EFW) ≤5%ile, EFW ≤10%ile with abnormal umbilical artery Dopplers, or EFW ≥2 weeks below expected if <24 weeks. Those with an identifiable etiology for FGR were excluded. Blood samples were collected at FGR diagnosis, and cfDNA screening was performed using Illumina's Research Use Only pipeline with analysis of all chromosome aneuploidies and copy number variants ≥7Mb. cfDNA sample metrics were collected from the laboratory, and clinical outcomes were obtained from the medical record.

**Results**: A total of 68 cases were enrolled at mean 30 ± 3 weeks. Mean maternal age was 34 ± 6.8 years, mean BMI was 28 ± 8.3 kg/m^2^, and the cohort was 25% Asian, 6% Black, 22% Hispanic, and 35% non‐Hispanic White. No cases of aneuploidy, including RAT, were identified. Total cfDNA concentration was significantly higher in cases that developed a hypertensive disorder of pregnancy (HDP) (518.4 vs 110.5 pg/uL, *p* = 0.004) and in cases that delivered preterm (539.1 vs 105.5 pg/uL, *p* = 0.04). There were no differences in fetal fraction (FF) in those who developed HDP, while FF was significantly lower in those who delivered preterm (17.4% vs 22.5%, *p* = 0.001).

**Conclusion**: In this cohort of unexplained severe early onset FGR, no cases of RAT were identified via WGS of cfDNA. Significantly higher total cfDNA was seen in those with more severe placental dysfunction. This adds to existing literature linking cfDNA concentration, placental dysfunction, and adverse outcomes.

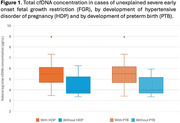

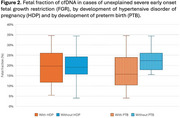

**3:30 PM – 5:00 PM**

## 1200 | **Gestational Age at Cervical Length Measurement and the Risk of Preterm Birth in Twin Pregnancies**

Natalie Kongkham^1^; Arietta Vayenas^1^; Liran Hiersch^2^; Jon F. Barrett^3^; Nir Melamed^4^

^1^Sunnybrook Research Institute, Toronto, ON; ^2^Lis Hospital for Women's Health, Tel Aviv Sourasky Medical Center Gray Faculty of Medicine, Tel Aviv University, Israel, ISRAEL, Tel Aviv; ^3^McMaster University, Hamilton, ON; ^4^Sunnybrook Health Sciences Center, Toronto, ON

**Objective**: Sonographic cervical length (CL) is a well‐established predictor of spontaneous preterm birth (sPTB) in twin pregnancies. However, its predictive accuracy may vary by the gestational age (GA) at the time of CL measurement. Earlier screening may yield higher positive predictive value (PPV), as early cervical shortening is associated with higher sPTB risk, but lower sensitivity, since some patients only experience shortening later in gestation. Data on the week‐specific predictive accuracy of CL in twin pregnancies are limited. Therefore, this study aimed to assess the effect of GA at CL measurement on the predictive accuracy of CL for sPTB in twin pregnancies and to develop week‐specific risk estimates to facilitate patient counseling.

**Study Design**: This was a retrospective study of twin pregnancies followed at a single center (2012‐2024). CL was routinely measured every 2‐3 weeks from 16‐18 weeks to 28‐30 weeks. For each 2‐week interval between 16‐29 weeks, we evaluated the discriminative ability of CL for predicting sPTB <32 weeks using the area under the ROC curve (AUC), and described its predictive accuracy at the fixed threshold of ≤25mm using sensitivity, specificity, and positive and negative predictive values.

**Results**: A total of 1760 patients who underwent 7392 CL measurements met the study criteria. The AUC of CL for sPTB <32 weeks increased with GA at measurement, from 0.636 (0.565‐0.706) at 16‐17 weeks to 0.830 (0.741‐0.919) at 28‐29 weeks. Sensitivity increased from 10.3% to 69.0% while PPV declined from 41.2% to 12.9% as GA at measurement increased from 16‐17 weeks to 28‐29 weeks (Figure 1). Week‐specific sPTB risk estimates by CL were developed (Figure 2).

**Conclusion**: In twin pregnancies, the predictive performance of CL for sPTB varies by GA at measurement. These findings can inform patient counseling based on CL and GA at CL measurement.

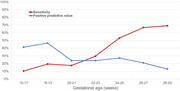

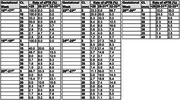

**3:30 PM – 5:00 PM**

## 1201 | **The Invisible Weight: Trauma Exposure and Early Gestational Weight Gain**

Natalie E. Poliektov^1^; Mariana Rocha^2^; Kaitlyn K. Stanhope^3^; Alicia Smith^1^; Abigail Lott^2^; Vasiliki Michopoulos^2^; Suchitra Chandrasekaran^1^

^1^Department of Gynecology and Obstetrics, Emory University School of Medicine, Atlanta, GA; ^2^Department of Psychiatry, Emory University School of Medicine, Atlanta, GA; ^3^Rollins School of Public Health, Rollins School of Public Health, GA

**Objective**: Increased early gestational weight gain (eGWG), weight gained in the 1^st^ & 2^nd^ trimesters, is associated with adverse pregnancy outcomes (APOs), including hypertensive disorders of pregnancy (HDP). Prior research has established links between trauma exposure & weight dysregulation outside of pregnancy. However, the impact of trauma on eGWG remains poorly understood. Given the known risks of increased eGWG, we evaluated the association between trauma exposures & eGWG.

**Study Design**: We performed a secondary analysis of a prospective longitudinal cohort study investigating associations between trauma exposures & APOs at an urban safety net hospital. Childhood trauma (CT), Post‐Traumatic Stress (PTSD), Lifetime Stress (LS) and Adult Trauma (AT) were captured using validated trauma exposure questionnaires (Table 1). Weight, systolic & diastolic blood pressure (BP) were recorded in each trimester. eGWG was calculated as (*final 2^nd^ trimester weight‐initial 1^st^ trimester weight)*. BP nadirs, calculated as (*final* 2*^nd^ trimester BP‐initial 1^st^ trimester BP)*, were used as markers for hypertensive risk. Linear regression models were conducted to investigate associations between trauma exposure scores & eGWG, adjusting for confounders.

**Results**: Consistent with prior studies, eGWG in our cohort (*N* = 328) was associated with a blunted systolic BP nadir, independent of age, body mass index (BMI), & chronic HTN *(β = 0.24, 95% CI 0.03‐0.44, p = 0.026)*, increasing the risk for HDP. CT *(β = 0.17, 95% CI 0.05‐0.29, p = 0.006)*, PTSD *(β = 0.18, 95% CI 0.02‐0.33, p = 0.026)* & LS *(β = 0.95, 95% CI 0.10‐1.81, p = 0.03)* scores were associated with eGWG when controlling for BMI. There was no association between AT scores & eGWG *(β = 1.17, 95% CI ‐0.40‐2.74, p = 0.14)* or between any trauma scores & 3^rd^ trimester weight gain (*p* >0.05).

**Conclusion**: Our data demonstrate that CT, PTSD & LS are associated with increased eGWG, suggesting that pre‐pregnancy trauma exposure influences gestational metabolic regulation. Further studies need to explore underlying mechanisms & test trauma‐informed interventions to optimize eGWG & improve maternal health outcomes.

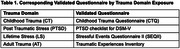

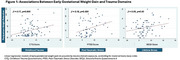

**3:30 PM – 5:00 PM**

## 1202 | **Three Phenotypes of Maternal Heart Rate Adaptation During the First Postpartum Year**

Natalie M. Williams^1^; Teresa Lillis^2^; Devon Hansen^3^

^1^Washington State University Elson S. Floyd College of Medicine, Kennewick, WA; ^2^Rush University Medical Center, Chicago, IL; ^3^Washington State University Sleep and Performance Research Center, Spokane, WA

**Objective**: Wearable data offers unique windows into perinatal cardiovascular adaptation. Previous studies show heart rate (HR) decreases after delivery and stabilizes by 3‐12 months postpartum. However, daily HR patterns across the preconception–postpartum arc remain undescribed. We leveraged two years of wearable HR data to determine whether distinct adaptation trajectories emerged across the postpartum.

**Study Design**: Twenty‐four primiparous women (26‐43 y) contributed daily HR data from 12 months pre‐conception through 12 months postpartum and completed surveys capturing subsequent pregnancies. Each participant's mean HR across three preconception months defined their baseline. Daily HR “difference scores” (daily HR minus baseline) were cumulatively summed to generate HR‐change curves. Data‐driven clustering identified groups with similar curves.

**Results**: HR curves during preconception oscillated around baseline; most participants showed monotonic increases across pregnancy. Postpartum, three trajectory groups emerged (Figure 1): “Sustained‐Low” (*n* = 9) rapidly declined following birth and remained below baseline throughout the postpartum year; “Recovery‐to‐Plateau” (*n* = 9) declined rapidly then plateaued by ∼6 months; “Rebound‐High” (*n* = 5) showed initial decline then subsequent HR increase beginning ∼6 months postpartum. Every “Rebound‐High” participant conceived again within 6–9 months postpartum; no additional postpartum pregnancies occurred in other groups.

**Conclusion**: Daily wearable HR data uncovered three distinct postpartum adaptation phenotypes. Most notably, short interpregnancy interval (IPI <12 months) showed complete association with a rebound pattern, with every participant conceiving again within 6–9 months, showing renewed HR elevation ∼6 months postpartum. This represents the first continuous monitoring evidence that IPI timing correlates with distinct cardiovascular adaptation patterns. These findings warrant further investigation into the relationship between HR trajectory phenotypes and maternal health outcomes.

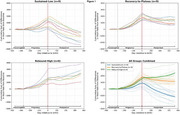

**3:30 PM – 5:00 PM**

## 1203 | **Scheduled IV Ketorolac After Cesarean Delivery and Effects on Opioid Use: A cost Effectiveness Analysis**

Neha Mishra^1^; Lily Ben‐Avi^2^; Amelia H. Gagliuso^3^; Aaron B. Caughey^2^

^1^Oregon Health and Science University, Oregon Health and Science University/Portland, OR; ^2^Oregon Health & Science University, Oregon Health & Science University, OR; ^3^Oregon Health and Science University, Portland, OR

**Objective**: Inadequate pain control after cesarean section has been associated with a greater risk of opioid use and postpartum depression. With cesareans being among the most common surgeries performed, it is imperative to use a pain control strategy that reduces opioid use while achieving adequate pain control. The use of opioid‐sparing multimodal analgesia has been increasing over the past decade, with opioids being primarily utilized for breakthrough pain. The American College of Obstetricians and Gynecologists (ACOG) recommends scheduled NSAIDs as first‐line postoperative analgesia. However, the optimal type, dose, and timing of administration have not yet been defined. In this study, we evaluated the cost‐effectiveness of scheduled IV ketorolac versus placebo in reducing the risk of developing an opioid use disorder (OUD), overdose, and opioid‐related death.

**Study Design**: We constructed a decision‐analytic model to compare outcomes between receiving IV ketorolac and IV normal saline. Our theoretical cohort included 1,161,896 individuals, reflecting the number of cesareans performed in 2023. Outcomes were the development of an OUD, overdose, opioid‐related death, costs, and quality adjusted life years (QALYs). We used a willingness‐to‐pay threshold of $100,000/QALY. Model inputs were derived from the literature and assessed through sensitivity analyses.

**Results**: In our cohort, the use of IV ketorolac was associated with 6739 fewer cases of OUD, 439 fewer cases of having an opioid‐related overdose, and 11 fewer cases of opioid‐related death. Prescribing IV ketorolac was cost‐effective with an ICER of $531.93/QALY. Once variability was incorporated into our model inputs via a Monte Carlo simulation, we found that IV ketorolac was cost‐effective and cost‐saving in 86% of the samples.

**Conclusion**: In our study, prescribing IV ketorolac was a cost‐effective strategy to improve outcomes and minimize the adverse events associated with opioid prescription. Adopting a standard of prescribing IV ketorolac post‐cesarean delivery would be advantageous for both recovery of patients and the health system.

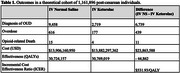

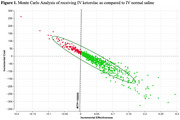

**3:30 PM – 5:00 PM**

## 1204 | **Addressing Perinatal Health Disparities Through Institution‐Wide Offering of Elective Inductions of Labor After 39 Weeks**

Nicola F. Tavella^1^; Erinma Ukoha^2^; Sara E. Edwards^3^; Toni Stern^2^; Kimberly Glazer^2^; Angela T. Bianco^2^

^1^Division of Maternal‐Fetal Medicine, Icahn School of Medicine, Icahn School of Medicine at Mount Sinai Hospital, New York, NY; ^2^Icahn School of Medicine at Mount Sinai, New York, NY; ^3^Division of Maternal‐Fetal Medicine, Icahn School of Medicine at Mount Sinai, New York, NY

**Objective**: Sociostructural barriers yield disparate perinatal outcomes. Identifying broad policies that reduce health disparities can inform targeted interventions. The Social Vulnerability Index (SVI) quantifies neighborhood‐level sociostructural adversity. We examined if expanding access to elective inductions of labor (IOL) post‐ARRIVE trial changed SVI‐based perinatal disparities.

**Study Design**: This was a retrospective study of all nulliparous patients with term, singleton, vertex gestations who delivered at a large urban US hospital from January 2013—December 2022. We excluded patients with scheduled cesarean deliveries (CD). Data were abstracted from electronic medical records. Addresses were geocoded to 2020 US Census tracts and SVI values (0‐1). SVI was dichotomized as high (≥ 0.75) or not high (< 0.75). The primary outcome was IOL. Secondary outcomes were CD, chorioamnionitis, and NICU admission. Our institution's policy change allowed elective IOL for everyone ≥ 39 weeks’ gestation. A difference‐in‐differences analysis compared SVI‐based disparities in pre‐change (2013‐2017) and post‐change (2018‐2022) cohorts. Adjusted, clustered linear probability models estimated absolute differences in likelihood, comparing (1) all individuals pre‐ and post‐change, and (2) an interaction term representing the pre/post change disparity.

**Results**: We included 21,854 patients; 22% had high SVI. Patients with high SVI disproportionately identified as non‐White, lacked private insurance, and had greater rates of hypertension and NICU admission (*p* < 0.001). IOL rates did not differ by SVI (Table 1). IOL was more likely in the post‐change period (9.0[7.5,10.5]). CD (‐3.8[‐5.2,‐2.4]) and NICU admission (‐1.5[‐2.4,‐0.7]) were less likely in the post‐change period. The disparity in chorioamnionitis decreased post‐change (‐0.5[‐0.9,‐0.02]; Table 2).

**Conclusion**: Rates of CD and NICU admission decreased post‐policy change. However, SVI‐based disparities were mostly unchanged. To meaningfully close perinatal health disparities, policies should target sociostructural barriers.

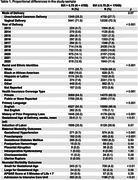

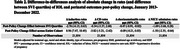

**3:30 PM – 5:00 PM**

## 1205 | **Accuracy of Diagnosis Codes in Administrative Data for Measuring Shoulder Dystocia**

Nicole Krenitsky^1^; Yongmei Huang^2^; Karthik Natarajan^3^; Nishanth Parameshwar Pavinkurve^3^; Timothy Wen^4^; Alexander M. Friedman^2^; Jason D. Wright^3^; Mary E. D'Alton^2^; Dena Goffman^5^; Uma M. Reddy^3^; Xiao Xu^3^

^1^NYP/Columbia, New York, NY; ^2^Division of Maternal‐Fetal Medicine, Department of Obstetrics and Gynecology, Columbia University Irving Medical Center, New York, NY; ^3^Columbia University Irving Medical Center, New York, NY; ^4^Division of Maternal Fetal Medicine, Department of Obstetrics, Gynecology and Reproductive Sciences, University of California San Diego, San Diego, CA; ^5^Columbia University, New York, NY

**Objective**: Research on shoulder dystocia often utilizes International Classification of Diseases (ICD) diagnosis codes to identify this obstetric emergency. However, the accuracy of ICD codes for measuring shoulder dystocia has not been validated. Potential misclassification can hinder understanding of its prevalence and risk factors and thus limit ability to develop interventions. This study aimed to examine the accuracy of ICD diagnosis codes in administrative data for measuring shoulder dystocia.

**Study Design**: This is a retrospective cohort study of all patients with vaginal delivery of a singleton, vertex gestation from 1/1/2016 to 12/31/2024 at a large, academic health system. Shoulder dystocia was identified in two ways: 1) based on ICD diagnosis codes (ICD‐9: 660.4*/ICD‐10: O66.0) in administrative data, and 2) medical chart abstraction by trained staff in a departmental obstetrics database. The latter was used as the reference standard for our evaluation. Shoulder dystocia identified based on ICD diagnosis codes was compared against the reference standard to determine its sensitivity, specificity, and positive and negative predictive values.

**Results**: The overall sample included 30,774 patients (characteristics summarized in Table 1). Among them, shoulder dystocia was identified in 717 (2.3%) deliveries based on ICD codes and 708 (2.3%) based on chart abstraction. ICD codes had a sensitivity of 93.1% and specificity of 99.8% in identifying shoulder dystocia, along with a positive and negative predictive value of 91.9% and 99.8%, respectively (Table 2).

**Conclusion**: In this first study to evaluate the accuracy of administrative codes for shoulder dystocia, ICD‐9 and ‐10 codes performed with high specificity and relatively high sensitivity at the study institution. Further evaluation in other data sources is warranted in order to support the broader use of these codes for research purposes.

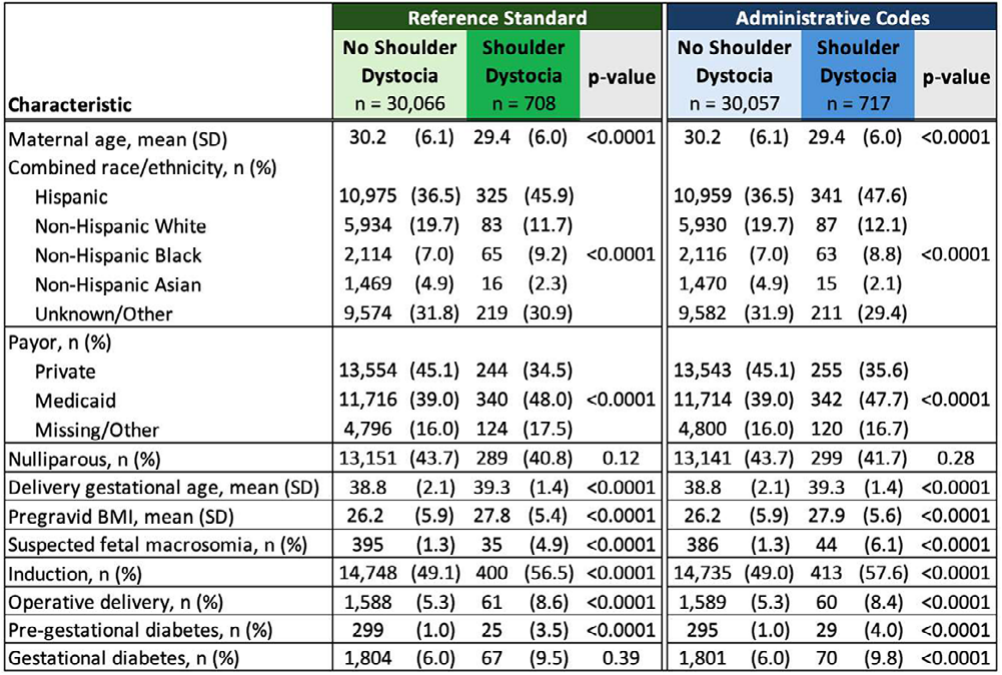

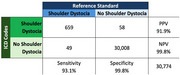

**3:30 PM – 5:00 PM**

## 1206 | **Gestational Age and Indications for Delivery in Prenatally Diagnosed Gastroschisis: A 23‐Year Retrospective Cohort Study**

Nikan Zargarzadeh^1^; Giulia Bonanni^1^; Enaja Sambatur^2^; Kjersti M. Aagaard^3^; Alireza A. Shamshirsaz^4^

^1^Boston Children's Hospital, Boston, MA; ^2^Southeast Health Medical Center, Southeast Health Medical Center, AL; ^3^HCA, Houston, TX; ^4^Boston Children's Hospital, Harvard Medical School, Boston, MA

**Objective**: The optimal timing and indication for delivery in gastroschisis remain subjects of ongoing debate. This study evaluates clinical characteristics and postnatal outcomes associated with varying indications for preterm delivery in this population.

**Study Design**: This IRB‐approved retrospective cohort study included pregnancies with prenatally diagnosed gastroschisis delivered at 34–38⁶⁄₇ weeks at a single center (2000–2023). Deliveries were grouped by indication: (1) bowel concern (e.g., dilation, wall thickening, echogenicity), (2) non‐reassuring fetal status defined by obstetric indications (NRFS), and (3) preterm labor (PTL). Neonatal outcomes—closure timing, surgical needs, hospital length of stay, sepsis, feeding initiation, and complex gastroschisis rate—were compared using Kruskal‐Wallis and Fisher's exact tests, with pairwise comparisons against the bowel concern group.

**Results**: A total of *n* = 83 infants met inclusion criteria: 22 delivered due to bowel concern, 36 for NRFS, and 25 for PTL. Age at abdominal wall closure was significantly earlier in the NRFS and PTL groups compared to the bowel concern group after adjusting for gestational age at delivery (adjusted *p* = 0.013 for NRFS; *p* = 0.025 for PTL). However, there were no significant differences across groups in number of surgical interventions, LOS, incidence of sepsis, timing to enteral feeding initiation, or type of surgical repair.

**Conclusion**: Although bowel‐related concerns prompted earlier delivery, this strategy did not translate into improvements in key clinical outcomes. In contrast, infants delivered for spontaneous or fetal‐status‐related reasons had comparable or even more favorable postnatal trajectories. These findings call into question the clinical utility and favorable prognostic value of bowel appearance alone as an indication for preterm delivery in gastroschisis and highlight the need for more evidence‐based delivery criteria.

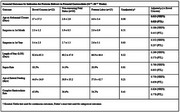

**3:30 PM – 5:00 PM**

## 1207 | **Planned Versus Unplanned Cesarean Delivery for Placenta Previa: Balancing Neonatal Risks and Maternal Morbidity**

Nirit Maliyanker^1^; Lital Shaham^2^; Michal Fishel Bartal^3^; Noa Gonen^4^; Avishag Abecassis^5^; Michal Axelrod^6^

^1^Sheba Medical Center, Ramat Gan, HaMerkaz; ^2^Department of Obstetrics and Gynecology, Sheba Medical Center, Sheba Medical Center, HaMerkaz; ^3^Division of Maternal‐Fetal Medicine, Department of Obstetrics, Gynecology and Reproductive Sciences, Sheba Medical Center, Tel Aviv University, Israel, Ramat Gan, HaMerkaz; ^4^Wolfson Medical Center, Holon, HaMerkaz; ^5^Department of Obstetrics and Gynecology, Sheba Medical Center, Ramat Gan, HaMerkaz; ^6^Sheba Medical Center, Sheba Medical Center, HaMerkaz

**Objective**: Individuals with placenta previa undergoing unplanned cesarean delivery (CD) may have worse outcomes than those with planned CD. We compared maternal and neonatal outcomes between planned and unplanned CD.

**Study Design**: We conducted a retrospective cohort study (2011‐2015) at a single tertiary center, including singleton pregnancies with placenta previa.  Suspected placenta accreta spectrum cases were excluded. Primary outcomes were composite maternal and neonatal morbidity. Maternal composite included postpartum fever, hemorrhage, transfusion, surgical intervention (CT drainage, relaparotomy), ICU admission, hospitalization >6 days or readmission. Neonatal composite included Apgar scores <7 at 5min, cord pH < 7.1, NICU admission, RDS, TTN, hypoglycemia, or sepsis. Significant clinically relevant variables were adjusted for using logistic regression models.

**Results**: Of 144,601 deliveries, 422 (0.3%) met inclusion criteria; 274 (64.9%) had a planned CD and 148 (35.1%) an unplanned CD. Groups were similar in baseline. Unplanned CD was associated with more antepartum bleeding (70.3% vs. 28.5%, OR 5.94, 95% CI 3.83–9.22). Gestational age was lower in the unplanned group (35.4 vs. 37.3 weeks, *p* < 0.001), with increased use of general anesthesia (41.9% vs. 8.4%, OR 7.87).

Maternal morbidity was higher following unplanned CD (34.5% vs. 12.0%, aOR 3.23, 95% CI 1.80–5.81, *p* < 0.001), even after adjustment. Neonatal morbidity was also higher (62.2% vs. 24.1%) but not significant  after adjustment for gestational age (aOR 1.037, 95% CI 0.57‐1.89, *p* = 905).

**Conclusion**: About one‐third of individuals with placenta previa undergo unplanned delivery before 37 weeks, independently increasing maternal morbidity. Neonatal complications were more frequent but largely driven by earlier gestational age at birth. These findings highlight the value of anticipatory planning, especially in cases with antepartum bleeding, to reduce the likelihood of urgent cesarean and improve maternal outcomes without compromising neonatal safety.

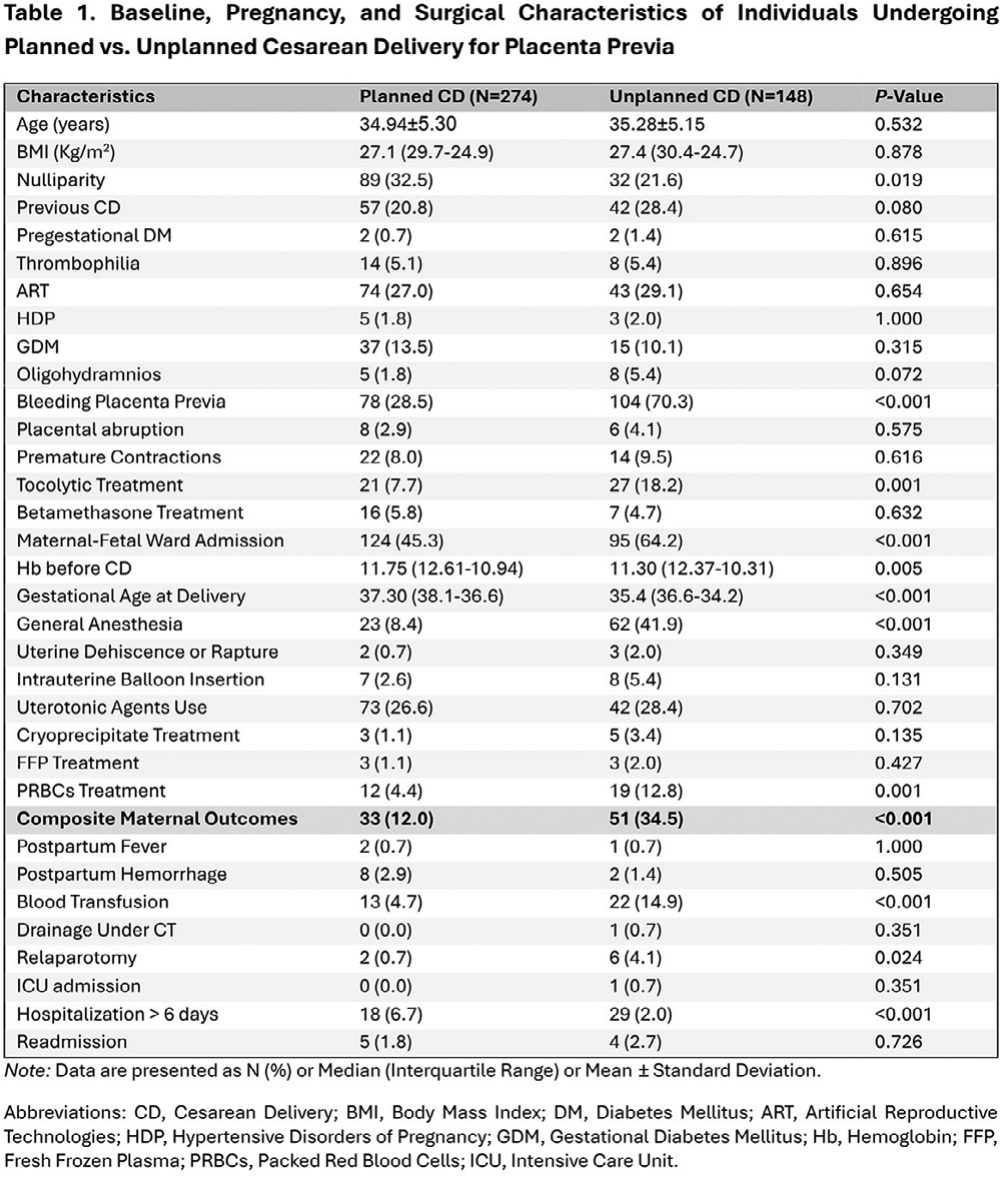

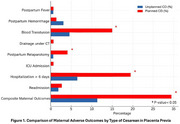

**3:30 PM – 5:00 PM**

## 1208 | **Food Deserts and Maternal Blood Pressure: A Hidden Risk for Gestational Hypertension**

Noor K. Al‐Shibli^1^; Elizabeth C. Rhodes^2^; Erin Ferranti^3^; Anne L. Dunlop^4^; Suchitra Chandrasekaran^5^

^1^Emory University School of Medicine, Emory University School of Medicine, GA; ^2^Rollins School of Public Health, Emory University, Atlanta, GA; ^3^Nell Hodgson Woodruff School of Nursing, Dept of Gynecology & Obstetrics, Emory University, Nell Hodgson Woodruff School of Nursing, Dept of Gynecology & Obstetrics, Emory University/ Atlanta, GA; ^4^Department of Gynecology and Obstetrics, Division of Research, Emory University School of Medicine, Dept of Gynecology & Obstetrics, Division of Research, Emory University School of Medicine/ Atlanta, GA; ^5^Department of Gynecology and Obstetrics, Emory University School of Medicine, Atlanta, GA

**Objective**: Food deserts (FD), regions with limited access to nutritious/affordable foods, are emerging threats to cardiometabolic health in non‐pregnant states. In pregnancy, the burden of metabolic diseases, including hypertensive disorders of pregnancy (HDP) is rising. Given the known links between social determinants of health & cardiometabolic outcomes, we aimed to examine the association between residence in a FD & the development of HDP.

**Study Design**: Using our institutional obstetric database, we performed a retrospective cohort study of deliveries from 2016‐2023 with documented zip‐code & clinical/demographic data. FD status was assigned by zip‐code of primary residence according to the U.S. Department of Agriculture (USDA) definitions. HDP was a composite of gestational hypertension (GHTN), preeclampsia with severe features (PRESF) & without severe features (PREWOSF). Kruskal Wallis & Chi square tests were performed to compare continuous & categorical variables, respectively. Poisson regression models, controlling for age, race, body mass index (BMI), & chronic hypertension (CHTN) were used to estimate incidence risk ratios (IRR).

**Results**: Among *N* = 21,065 pregnancies, 7160 (34.0%) lived in a FD. Compared to non‐FD residents, those in FDs had a higher BMI *(30.2±7.9 vs 29.5±7.2, p < 0.001)* & were younger *(29.1±6.1 vs 29.8±6.1, p < 0.01). * FD residents had higher rates of HDP *(31.5% vs 29.0%, p < 0.01)*, GHTN *(19.25% vs 17.3%, p = 0.002)*, & CHTN *(11.4% vs 9.2%, p < 0.001)*. Rates of PREWOSF *(p = 0.098)* or PRESF *(p = 0.068)* did not differ significantly between groups. While FD residence was not significantly associated with increased risk of HDP after adjustment, *(IRR 1.05, 95% CI 0.98‐1.18, p = 0.14)*, it was associated with a 1.1‐fold higher risk of developing GHTN *(IRR 1.1, 95% CI 1.01‐1.18, p = 0.02)*.

**Conclusion**: Living in a FD is associated with an increased risk of GHTN, though not with HDP overall. Our findings underscore the need for future studies to explore mechanisms linking neighborhood‐level inequities to maternal cardiometabolic health & develop interventions to advance perinatal health equity & outcomes.

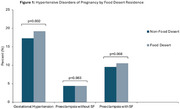

**3:30 PM – 5:00 PM**

## 1209 | **A Decade of Diabetes in Pregnancy: National Trends and Disparities by Access to Care**

Oladunni Ogundipe; Frank I. Jackson; Sarah H. Abelman; Luis A. Bracero; Matthew J. Blitz

Northwell, New Hyde Park, NY

**Objective**: Diabetes in pregnancy is associated with increased maternal morbidity and requires intensive surveillance. Access to care may influence both the identification and management of diabetes. This study examined national trends in diabetes diagnoses in pregnancy over the past decade, with a focus on geographic disparities related to healthcare access.

**Study Design**: This was a retrospective cohort study using 2014 and 2023 U.S. National Vital Statistics data from all 50 states and Washington, DC. Nulliparous singleton pregnancies were included. We identified regions with limited healthcare access using the March of Dimes classification, which stratifies areas as full, moderate, low access, or maternity care deserts based on access to obstetric hospitals or birth centers, presence of obstetric clinicians, and proportions of uninsured patients. Logistic regression was used to evaluate changes in rate of diagnosis from 2014 to 2023, with *p* < 0.05 considered significant. Analyses were performed using R version 4.4.1.

**Results**: There were 1,471,031 nulliparous singleton births in 2014 and 1,402,230 nulliparous singleton births in 2023. Nationally, the rate of diabetes diagnosis increased from 6.0% in 2014 to 8.5% in 2023. When evaluating states with the highest proportion of maternity care deserts (ND, SD, OK, MO, NE, AR), rates of diabetes diagnosis were less than in states with more access to care (5.8% vs 6.0% respectively, OR 0.95, 95% CI 0.92‐0.98). Similarly, in 2023, diagnosis rates were fewer in areas with high concentrations of maternity health deserts than in states that had fewer maternity health deserts (8.4% vs 8.6%, OR 0.97, 95% CI 0.95‐1.00).

**Conclusion**: Diabetes diagnoses during pregnancy have increased nationally over the past decade, reflecting either rising prevalence or improved screening. However, persistently lower diagnosis rates in states with limited maternity care access raise concerns about underdiagnosis. Targeted efforts to improve detection and management in underserved regions are urgently needed.

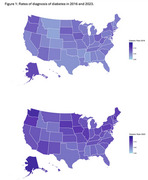

**3:30 PM – 5:00 PM**

## 1210 | **Nomogram for Predicting Shoulder Dystocia After Term Vaginal Delivery**

Or Bercovich^1^; Daniela Chen^2^; Yuval Neeman^3^; Yossi Geron^4^; Ohad Houri^5^; Ophir Blickstein^1^; Yael Rosental^6^

^1^Helen Schneider Hospital for Women, Rabin Medical Center, Petach Tikva, Israel, Helen Schneider Hospital for Women, Rabin Medical Center, Petach Tikva, HaMerkaz; ^2^Helen Schneider Hospital for Women, Rabin Medical Center‐Beilinson Hospital, Petach Tikva, Helen Schneider Hospital for Women, Rabin Medical Center‐Beilinson Hospital, Petach Tikva, HaMerkaz; ^3^Rabin Medical Center, Petach Tikva, HaMerkaz; ^4^Helen Schneider Hospital for Women, Rabin Medical Center, Petah Tikva, HaMerkaz; ^5^BCNatal Fetal Medicine Research Center, BARCELONA, Catalonia; ^6^‘Rabin medical center’, Rabin medical center/ Petach tikwa, HaMerkaz

**Objective**: To develop a predictive model and nomogram for shoulder dystocia following term vaginal delivery.

**Study Design**: This retrospective cohort study was conducted at a single tertiary care center. We included all vaginal deliveries at ≥37 weeks of gestation (either normal vaginal delivery [NVD] or vacuum extraction [VE]) between July 2012 and December 2023. Shoulder dystocia was the primary outcome and was defined as a ≥1‐minute delay in the delivery of the shoulders following the head, or the requirement of specific maneuvers. The full multivariate logistic regression included variables such as age, BMI, parity, diabetes (pregestational and gestational), gestational age, mode of delivery, clinical fetal weight estimation, oxytocin use, second‐stage duration, neuraxial analgesia and newborn gender. The stepwise model, selected based on the Akaike Information Criterion, retained the variables parity, clinical weight estimation, mode of delivery (NVD or VE), gestational diabetes mellitus A2 (GDM‐A2), and the pregestational diabetes. A nomogram was constructed based on the final logistic regression model. Internal validation and calibration were performed.

**Results**: Out of 74,146 eligible term vaginal deliveries, 278 cases (0.4%) of shoulder dystocia were identified. The final logistic regression model showed that increased parity (*p* < 0.001), higher clinical fetal weight estimation (*p* < 0.001), and vacuum‐assisted delivery (*p* < 0.001) were independently associated with higher risk of shoulder dystocia. The presence of GDM‐A2 and pregestational diabetes showed positive trends but did not reach statistical significance. Their respective predicted probabilities are represented in the nomogram (Figure 1).

**Conclusion**: This nomogram provides an individualized tool for estimating the risk of shoulder dystocia in term vaginal deliveries. Its use may support clinical decision‐making and intrapartum planning.

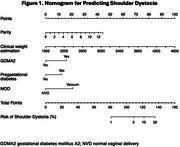

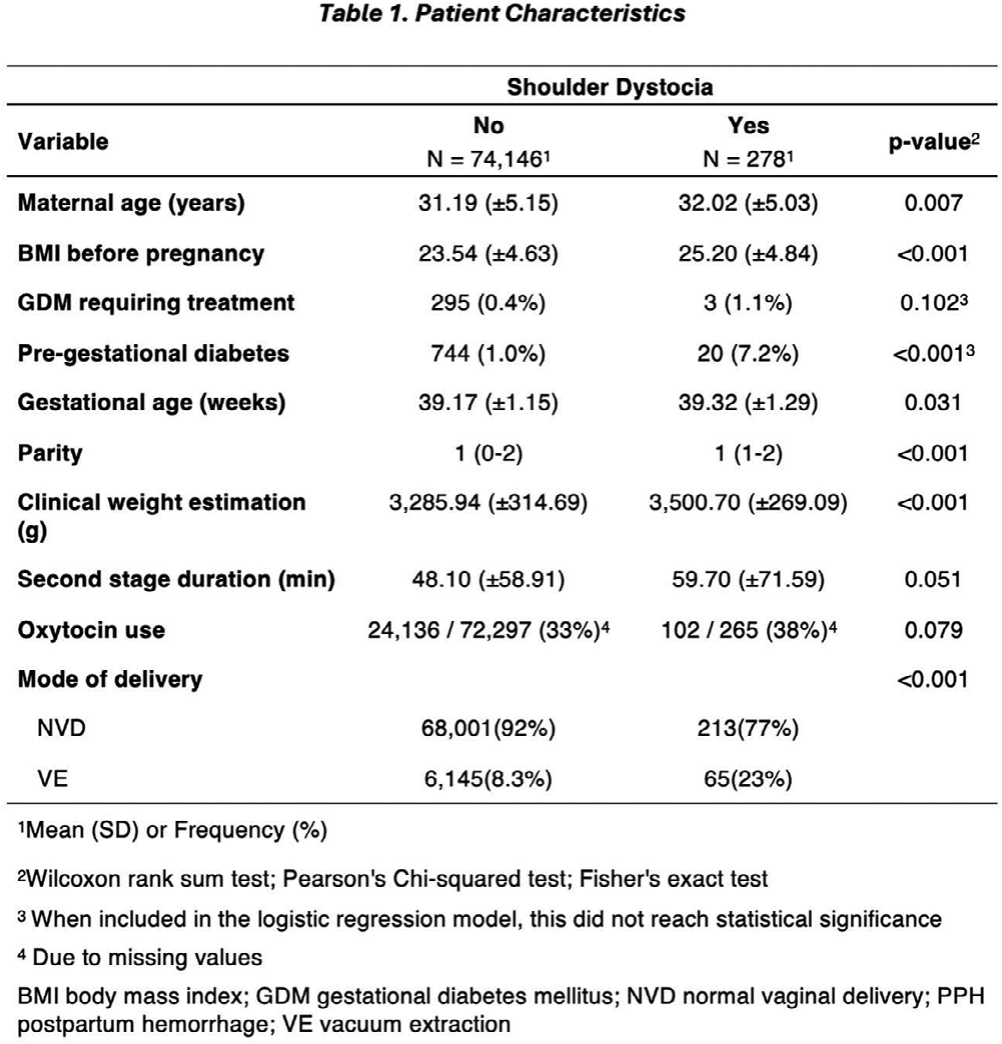

**3:30 PM – 5:00 PM**

## 1211 | **Breathing new Life Into Gestational Diabetes Diagnosis: Pilot Study of a Novel Non‐Invasive Breath Test**

Oren Barak^1^; Maya Oberman^2^; Tal Schiller^3^; Amir Shafat^4^; Inbal Avrahami^2^; Ziv Tsafrir^5^; Alon Ben‐Arie^5^; Edi Vaisbuch^2^

^1^Kaplan Medical Center, Rehovot, HaMerkaz; ^2^Kaplan Medical Center, Kaplan Medical Center, HaMerkaz; ^3^Edith Wolfson Medical Center, Holon, HaMerkaz; ^4^School of Pharmacy and Medical Sciences, University of Galway, Galway, Galway; ^5^kaplan medical center, Rehovot, HaMerkaz

**Objective**: To evaluate the feasibility and diagnostic accuracy of exogenous glucose oxidation, as measured by an oral ^13^C‐glucose breath test for diagnosing gestational diabetes mellitus (GDM).

**Study Design**: This single center prospective pilot study included individuals with singleton pregnancies scheduled for a 100‐gram oral glucose tolerance test (OGTT) before 33 weeks’ gestation. Participants ingested 100g ^13^C‐labelled glucose after an overnight fast. Venous glucose was obtained before ingestion and 1, 2, and 3 hours after. GDM was diagnosed with ≥2 abnormal values. Breath samples were collected every 15 minutes for 3 hours and analyzed by GC‐isotope‐ratio mass spectrometry. Percent dose recovered (PDR) was calculated from the breath enrichment of ^13^CO_2_ multiplied by assumed CO_2_ production rates, so that the fraction of oxidized ^13^C‐glucose (tracer) represented the fraction of the ingested glucose (tracee) metabolized to CO_2_. Feasibility outcomes were recruitment and test completion. Diagnostic accuracy was assessed using the area under the receiver operating characteristic (ROC) curve (AUC). Pearson correlation coefficient was used to calculate the agreement between glycemia and exogenous glucose oxidation. The study protocol was approved by the institutional review board committee.

**Results**: All 20 participants completed 3 hours of breath‐test sampling; no adverse events occurred. Patients diagnosed with GDM (*n* = 3) had a lower, yet not reaching significance, median PDR at 3 hours (PDR3h) than in those without GDM [9.4 (7.5‐10.3) vs 10.6 (9.6‐13.5)]. PDR3h discriminated GDM with an AUC 0.75 (95 % CI 0.33‐1.00), (Figure 1). A threshold of PDR3h <9.4% yielded 67% sensitivity and 82% specificity. PDR3h correlated inversely with OGTT glucose AUC (r  =  ‐0.48, r^2^  =  0.23, *p*   =  0.03), (Figure 2).

**Conclusion**: The ^13^C‐glucose breath test was readily completed and allowed quantification of ingested glucose metabolism. PDR3h showed a moderate ability to identify GDM and was inversely correlated with OGTT. These findings support exploring this novel non‐invasive diagnostic approach in larger cohorts.

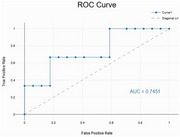

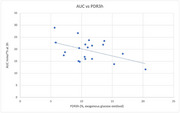

**3:30 PM – 5:00 PM**

## 1212 | **First‐in‐Human Study of a Novel Medical Device in PPROM**

Kathryn D. Wine, N/A^1^; Ross McQuivey^2^; Osvaldo Reyes^3^

^1^Novocuff, Inc., Danville, CA; ^2^Novocuff, Inc., Mountain View, CA; ^3^Hospital Santo Tomas, Panama City, Panama

**Objective**: Preterm prelabor rupture of membranes (PPROM) is associated with significant maternal and neonatal morbidity and mortality and therapeutic options are limited. Safety and feasibility of the investigational Novocuff Cervical Control System (CCS) for the treatment of PPROM were evaluated.

**Study Design**: A prospective, single‐arm study of the Novocuff CCS was completed at a single center in Panama from September 2023 to March 2024. Gravidas aged ≥ 18 years, with PPROM between 24‐34 weeks gestational age (GA), were eligible. Exclusions included suspected intra‐amniotic infection (IAI), signs of labor, vaginal bleeding, cervical length <15mm, cervical dilation > 1cm, and untreated pelvic/vaginal infection. After ≥ 48 hours of PPROM, eligible gravidas were consented and treated with the Novocuff CCS until up to 34 weeks GA. Standard PPROM expectant management, including corticosteroids and latency antibiotics, were provided. The primary outcome was a composite safety measure, including IAI within 1 week of device placement, chorioamnionitis, and device‐related cervicovaginal injury. Secondary outcomes included latency, amniotic fluid index (AFI) measurements, and participant/investigator satisfaction. Participants and their infants were followed until hospital discharge, with a 2‐week postpartum follow‐up visit. Descriptive statistics were used to present outcomes.

**Results**: Thirteen gravidas met study criteria (Table 1) and the device was successfully placed in every case. The primary endpoint success rate was 100%, with no IAI, chorioamnionitis or cervicovaginal injury. The mean PPROM latency was 24.08 days (median 17.0, min 8, max 65 days, Figure 1). AFI returned to normal from oligohydramnios or remained normal in all cases where AFI was measured post‐device placement. The investigator reported the device easy to use, and all participants reported they would use it again.

**Conclusion**: Results provide preliminary evidence that the Novocuff CCS is feasible and safe, with potential effectiveness in PPROM management. A larger comparative study is warranted.

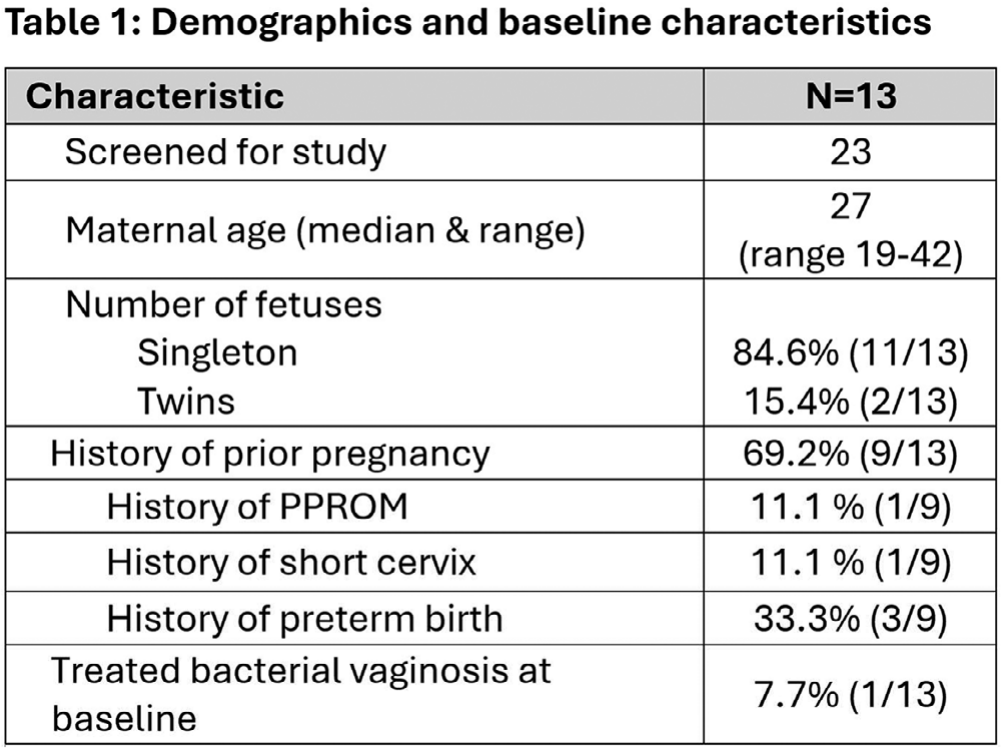

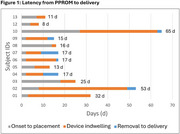

**3:30 PM – 5:00 PM**

## 1213 | **Oxytocin Dosing During TOLAC to Minimize the Uterine Rupture Risk: A Systematic Review and Meta‐Analysis**

Pierpaolo Nicolì^1^; Moti Gulersen^2^; Jordan Beacham^3^; Hila Hochler^4^; Alessandra Familiari^5^; Anna Locatelli^6^; Cynthia Abraham^7^; Ettore Cicinelli^1^; Amerigo Vitagliano^1^; Vincenzo Berghella^8^

^1^Unit of Obstetrics and Gynecology, Department of Interdisciplinary Medicine (DIM), University of Bari “Aldo Moro”, Policlinico of Bari, Piazza Giulio Cesare 11, 70124, Bari, BA, Italy, Bari, Puglia; ^2^Division of Maternal Fetal Medicine, Department of Obstetrics, Gynecology and Reproductive Science, Jefferson Health, Philadelphia, PA, Philadelphia, PA; ^3^Division of Maternal‐Fetal Medicine, Department of Obstetrics and Gynecology, Sidney Kimmel Medical College of Thomas Jefferson University, Philadelphia, PA, Philadelphia, PA; ^4^Laniado Medical Center, Netanya, Israel, Natanya, HaMerkaz; ^5^Department of Women and Child Health, Women Health Area, Fondazione Policlinico Universitario Agostino Gemelli, IRCCS, Università Cattolica del S. Cuore, Rome, Italy, Rome, Lazio; ^6^School of Medicine and Surgery, University of Milano‐Bicocca, Milan, Lombardia; ^7^Hofstra University ‐ Northwell Health System, Staten Island University Hospital, Staten Island, NY, New York City, NY; ^8^Thomas Jefferson University, Philadelphia, PA

**Objective**: To assess which oxytocin protocol, in terms of initial dose, increase amount, timing to next increase, and max dose, may minimize the uterine rupture (UR) risk during trial of labor after cesarean (TOLAC).

**Study Design**: PubMed, Embase, and Clinicaltrials.gov were searched up to September 21, 2024. RCTs and nonrandomized studies evaluating the association between oxytocin for induction and/or augmentation and UR in TOLAC vs. spontaneous TOLAC were eligible. Eligible studies had to report at least one oxytocin protocol component (initial dose, increase amount, timing to next increase, or max dose). The primary outcome was UR. Effect measures (odds ratios [ORs] with 95% confidence intervals [95% CI]) were calculated using a random‐effects model and pooled via meta‐analyses. Oxytocin exposure was categorized into three analyses: any use (induction and/or augmentation), induction only, and augmentation only. Subgroup analyses assessed differences based on initial dose (≤ 2 vs > 2 mU/min), increase amount (≤ 2 vs > 2 mU/min), timing to next increase (<30 vs ≥ 30 min), and max dose (≤ 20 vs > 20 mU/min). Sensitivity analyses were performed by excluding studies with <1,000 patients.

**Results**: Twenty‐one observational studies including 51,025 TOLAC patients were analyzed. Oxytocin was associated with increased UR risk in any use (OR, 1.80 (95% CI, 1.23‐2.63), *p* = 0.003, I^2^ = 0%) and augmentation only (OR, 1.87 (95% CI, 1.05‐3.33), *p* = 0.03, I^2^ = 0%). While initial dose and increase amount were not associated with UR, oxytocin administered at intervals <30 min (any use [sensitivity analysis] OR, 1.88 [95% CI, 1.01‐3.51], *p* = 0.047, I^2^ = 0%) and at a max dose > 20 mU/min (any use OR, 2.17 [95% CI, 1.28‐3.70], *p* = 0.004, I^2^ = 0%; augmentation only OR, 2.79 [95% CI, 1.03‐7.54], *p* = 0.04, I^2^ = 0%) was associated with increased UR risk (Table).

**Conclusion**: Oxytocin dosing during TOLAC was associated with increased UR risk. Specifically, the risk was raised with dose escalation intervals <30 min and max doses > 20 mU/min, while no clear relationship emerged for initial dose or increase amount. RCTs are needed to confirm the safest regimen.

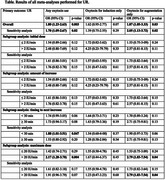

**3:30 PM – 5:00 PM**

## 1214 | **Intra‐Cesarean Myomectomies Are Safe and Practical Procedures**

Eli H. Bernstein^1^; Yael Baumfeld^2^; Yael Feinstein^3^; Offer Erez^4^; Polina Schwarzman^5^

^1^Baystate Medical Center, Easthampton, MA; ^2^Soroka University Medical Center, Soroka medical center, HaDarom; ^3^Schneider Children's Medical Center of Israel, Petach Tikva, Tel Aviv; ^4^Department of OB/GYN, Soroka University Medical Center, Ben Gurion University of the Negev, Be'er Sheva, HaDarom; ^5^Soroka University Medical Center, Soroka University Medical Center, HaDarom

**Objective**: To determine whether myomectomy during cesarean section is a safe and

Practical procedure.

**Study Design**: A retrospective cohort study was conducted at a tertiary medical university

Center. The study population included women with a documented myomectomy during

Cesarean delivery (CD) performed between 1999 and 2018.These were compared with

Women who had a standard CD. The control group was composed of patients who had

CD before and after index cases of intra‐cesarean myomectomy. We collected and

Compared the obstetric characteristics and perinatal outcomes, as well as intraoperative

And postoperative outcomes of the target population.

Data were retrieved from computerized database via electronic health records of the

Patients. Cases that lacked detailed reports of surgery and delivery were excluded. Data

Were analyzed using a chi‐square test for categorical variables and one‐way ANOVA for

Continuous variables. *p* < 0.05 was considered statistically significant.

**Results**: Seventy‐four women were included in the study group and 144 in the control

group. No significant difference was noted in the demographic characteristics of the two

groups. In the myomectomy group, mean maternal age was higher; obstetric history

Showed lower parity rates; a higher rate of assisted reproduction treatments; and mean

Length of surgery was longer (50.31±15.93 minutes versus 31.42±17.25 minutes, *p* <

0.001). No other significant differences were noted in intraoperative and immediate

Postoperative outcomes. Blood loss during surgery, reoperations, Intensive Care Unit

(ICU) admission rates, emergency room visits, and readmission rates did not differ

Significantly.

**Conclusion**: Based on our results, while intra‐cesarean myomectomy requires longer

Operative time than a standard cesarean section, it does not increase the risk of

Complications either during or after the procedure.

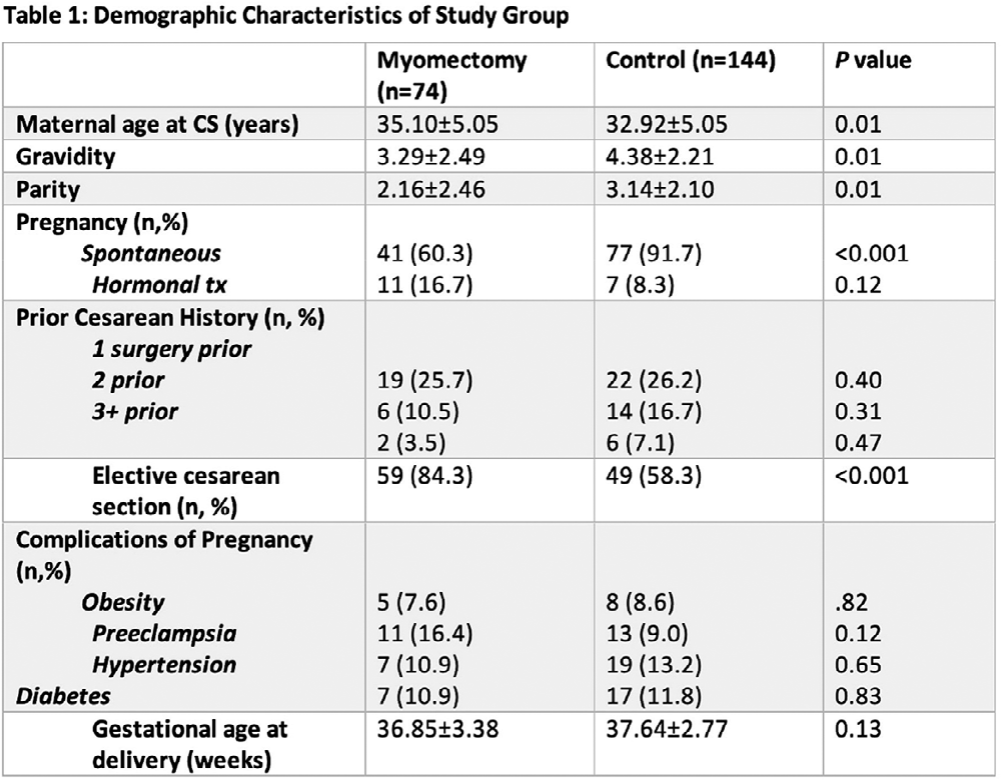

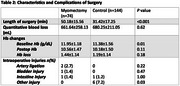

**3:30 PM – 5:00 PM**

## 1215 | **Prophylactic Antibiotics for Women With PROM >18 Hours: A cost Effectiveness Analysis**

Prakrunya Subhasree Badrinarayan^1^; Aaron B. Caughey^2^

^1^Oregon Health & Science University, Portland, OR; ^2^Oregon Health & Science University, Oregon Health & Science University, OR

**Objective**: Prelabor rupture of membranes (PROM) increases the risk of chorioamnionitis and worsens perinatal outcomes. For PROM >18 hours, this risk is even higher. Historically, such patients received antibiotic prophylaxis, but this has declined in the GBS screening era. A recent study re‐examined prophylaxis in this population. We evaluated the cost‐effectiveness of giving antibiotics (ampicillin‐gentamicin) vs. expectant management in patients who were GBS negative with PROM >18 hours.

**Study Design**: We built a decision‐analytic model in TreeAge Pro comparing prophylactic ampicillin‐gentamicin to expectant management. The theoretical cohort included 255,000 U.S. patients with term singleton pregnancies, negative GBS, and prolonged PROM. Outcomes included chorioamnionitis, postpartum endometritis, uterine atony, blood transfusion, neonatal early‐onset sepsis (nEOS), neonatal death, neonatal neurodevelopmental delay (nNDD), maternal acute kidney injury (AKI) from gentamicin, costs, and quality‐adjusted life years (QALYs). The willingness‐to‐pay threshold was $100,000/QALY. QALYs were discounted at 3%. Inputs were literature‐based and tested with univariate sensitivity analyses.

**Results**: In our cohort of 255,000 individuals, ampicillin‐gentamicin prophylaxis led to fewer cases of chorioamnionitis (2,550 vs. 22,950), endometritis (4,514 vs. 4932), uterine atony (14,747 vs. 16,407), blood transfusion (5,164 vs. 5651), nEOS (61 vs. 135), neonatal deaths (1,436 vs. 1443), and nNDD (17,725 vs. 17,728) compared to expectant management. However, gentamicin use resulted in 56 more maternal AKI cases. This reduction in adverse outcomes with prophylactic antibiotics led to lower costs, making it a dominant, cost‐effective strategy. Sensitivity analyses showed antibiotics remained cost‐effective as long as there is a 12% reduction in chorioamnionitis risk in the prophylaxis group vs the expectant management group.

**Conclusion**: Treating PROM >18 hours with ampicillin‐gentamicin reduced chorioamnionitis and improved outcomes, making it a cost‐effective strategy. This approach warrants reconsideration in clinical practice.

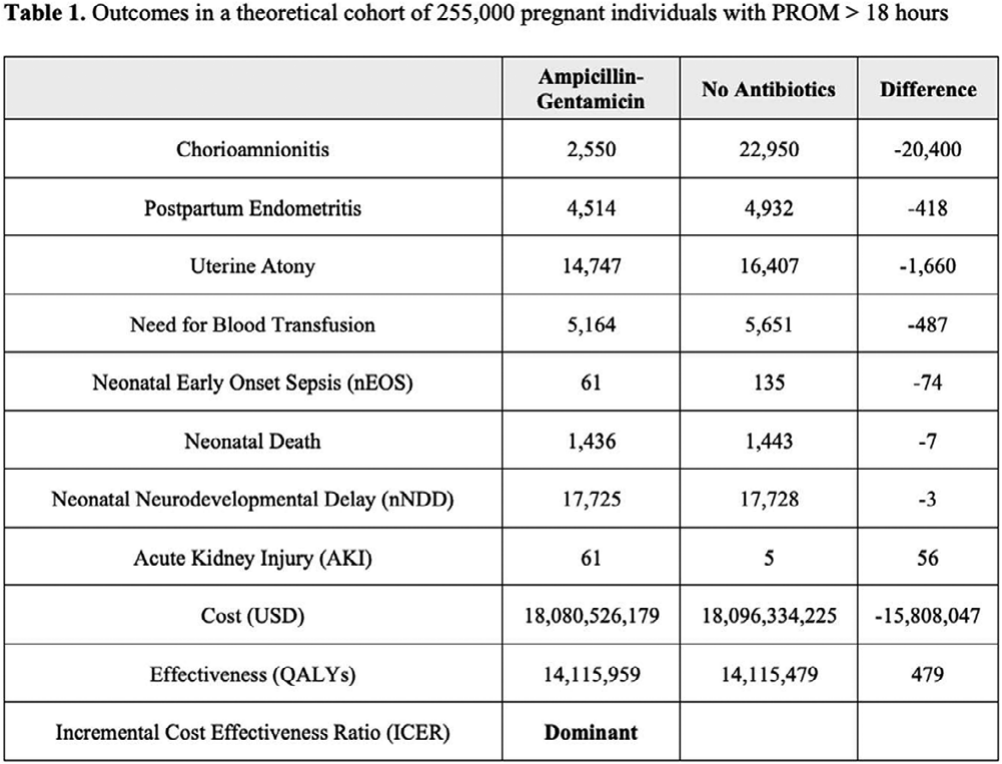

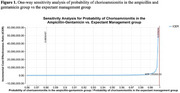

**3:30 PM – 5:00 PM**

## 1216 | **Management and Outcomes of Spontaneous Twin Anemia Polycythemia Sequence (TAPS)**

Praveen Ramesh^1^; Alexa Henderson^2^; Stephen P. Emery^1^; Tiffany Deihl^3^

^1^UPMC Magee‐Womens Hospital, Pittsburgh, PA; ^2^UPMC Magee Women's Hospital, University of Pittsburgh Magee Women's Hospital, PA; ^3^UPMC Magee Womens Hospital, Pittsburgh, PA

**Objective**: Optimal antenatal management of spontaneous twin anemia‐polycythemia sequence (TAPS) remains uncertain. We reviewed management and outcomes at a single center.

**Study Design**: We conducted a retrospective cohort study of monochorionic diamniotic twins diagnosed with spontaneous TAPS (delta MCA‐PSV ≥ 0.5) between 2019–2025. Management included selective feticide, expectant management, intrauterine transfusion (IUT) ± partial exchange transfusion (PET), laser therapy, laser + transfusion, and delivery. The primary outcome was obstetric complications. The secondary outcome was diagnosis‐to‐birth interval.

**Results**: Eighteen pregnancies met inclusion, with 94% (*n* = 17) Stage II or greater, and 17% (*n* = 3) with concomitant Stage II‐III twin‐to‐twin transfusion syndrome (TTTS). Initial management included laser (39%, *n* = 7), IUT± PET (33%, *n* = 6), expectant (11%, *n* = 2), selective feticide, laser+ IUT, or delivery (each 6%, *n* = 1). Median gestational age at diagnosis was 22w3d. Obstetric complications included worsening TAPS and/or development of other monochorionic pathology (56%, *n* = 10), IUFD of one twin (11%, *n* = 18), PPROM, placental abruption and delivery for non‐reassuring fetal status (each 6%, *n* = 1). Median (IQR) diagnosis‐to‐birth interval was 43 days (20–98). The interval was 143 days for selective feticide, 98 for laser (78–102), 81 for expectant management (58–104), 15 for IUT±PET (6–34), and 22 for laser + transfusion. After adjusting for GA at diagnosis, the median interval was 11.3 weeks longer with laser than IUT±PET (*p* = 0.009). When excluding those with concomitant TTTS, the interval remained greater than IUT±PET (6 weeks) but was not statistically significant (*p* = 0.25). Overall survival to discharge in this cohort was 94% (33/35).

**Conclusion**: Laser yielded the longest diagnosis‐to‐birth interval among non‐feticide options, though accounted for the only unintended fetal losses. Progression of TAPS or emergent monochorionic pathology was the leading complication, primarily after IUT±PET. Given small sample sizes, larger multicenter studies are needed to determine optimal management of spontaneous TAPS.

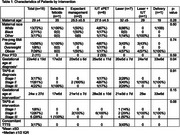

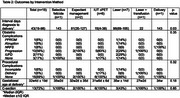

**3:30 PM – 5:00 PM**

## 1217 | **Peripartum Cardiomyopathy: Impact of Delivery Mode on Severe Maternal Morbidity and Cardiopulmonary Outcomes**

Prisca C. Diala^1^; Vanessa Kirschner^2^; Jiayue Chen^3^; Lorna Kwan^4^; Jeannette Lin^5^; Yalda Afshar^6^

^1^Department of Obstetrics and Gynecology, David Geffen School of Medicine at University of California, Los Angeles (UCLA), Los Angeles, CA; ^2^David Geffen School of Medicine at University of California, Los Angeles (UCLA), LOS ANGELES, CA; ^3^Department of Urology, David Geffen School of Medicine at University of California, Los Angeles (UCLA), Los Angeles, CA; ^4^Department of Urology, David Geffen School of Medicine at University of California, Los Angeles (UCLA), Los Angeles, CA; ^5^Division of Cardiology, Department of Medicine, Ahmanson/UCLA Adult Congenital Heart Disease Center, David Geffen School of Medicine at University of California, Los Angeles (UCLA), Los Angeles, CA; ^6^David Geffen School of Medicine at University of California, Los Angeles (UCLA), Los Angeles, CA

**Objective**: To evaluate the impact of delivery mode on severe maternal morbidity (SMM) and cardiopulmonary outcomes in peripartum cardiomyopathy (PPCM).

**Study Design**: This was a retrospective cohort study of hospitalized patients with antepartum diagnosis of PPCM using the National Inpatient Sample who delivered in the United States between 2016 and 2020. The primary outcome was a composite of SMM, rigorously defined and stratified by mode of birth (planned or unplanned/intrapartum). Secondary outcomes included rates of major adverse cardiopulmonary events. Multivariate regression models were adjusted for key demographic and clinical confounders.

**Results**: Among 3250 hospitalized patients with PPCM, nearly 60% underwent a trial of labor (TOL), yet 66% ultimately had a cesarean birth (Figure 1). There was no significant difference in composite SMM between intended vaginal birth or planned cesarean birth (aOR: 1.14; 95% CI: 0.79‐1.63, Figure 2). However, when analyzed by actual mode of birth, cesarean birth, planned or unplanned, was associated with a two‐fold increase in SMM compared to vaginal birth (aOR: 1.76; 95% CI: 1.22‐2.55, Figure 2). Rates of adverse cardiopulmonary outcomes, including arrhythmia, cardiogenic shock, and pulmonary embolism, did not differ by mode of birth (*p* >0.05).

**Conclusion**: In a large nationwide contemporary cohort of delivery outcomes in PPCM, our findings challenge the reflexive use of cesarean birth in this population. Cesarean birth, regardless of intent (planned or unplanned/intrapartum) confers a two‐fold increase in SMM compared to vaginal birth. These findings provide evidence to support a trial of labor in appropriately selected patients with PPCM and highlight the need for individualized multi‐disciplinary delivery planning to improve maternal outcomes for a vulnerable obstetrical population.

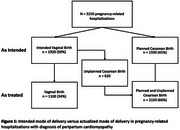

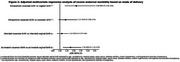

**3:30 PM – 5:00 PM**

## 1218 | **Pregnancy Outcomes in Primary Biliary Cholangitis and Primary Sclerosing Cholangitis: A National Cohort Study**

Priyanka Gaur^1^; Tatyana Kushner^2^; Emma J. Leipsner^3^; Kate Johnson^4^

^1^Division of Maternal‐Fetal Medicine, Department of Obstetrics and Gynecology, Weill Cornell Medicine, New York, NY; ^2^Tatyana Kushner, New York, NY; ^3^Weill Cornell Medical College, Philadelphia, PA; ^4^Weill Cornell Medical College, New York, NY

**Objective**: Primary sclerosing cholangitis (PSC) and primary biliary cholangitis (PBC) are rare, cholestatic liver diseases. Current understanding of how these diseases affect pregnancy is limited, and counseling is often extrapolated from intrahepatic cholestasis of pregnancy (ICP). We leveraged a large, national dataset to evaluate the independent influence of PBC and PSC on pregnancy outcomes.

**Study Design**: We performed a population‐based retrospective study of pregnant persons ages 12‐55 from 2000‐2025 using the TriNetX research network with electronic health record data from 106 U.S. health care organizations. A PSC and PBC pregnant cohort were compared to a non‐PSC and non‐PBC pregnant cohort, respectively. Primary outcomes were ICP, preterm delivery, postpartum hemorrhage, preeclampsia, gestational diabetes, cesarean section, fetal growth restriction, and stillbirth. A 1:1 propensity score match (PSM) balanced demographics and comorbid conditions, and multivariable logistic regression models were applied.

**Results**: We identified 5,070,078 pregnant participants of which 480 (0.009%) had PBC and 292 (0.006%) had PSC (Table 1). Nearly 40% of both PBC and PSC cohorts were prescribed ursodeoxycholic acid. PBC patients were on average older and more likely to have metabolic risk factors and develop advanced liver disease within 1 year of pregnancy (all *p* < 0.05). PSC patients were more likely to have inflammatory bowel disease (*p* < 0.001). After PSM, those with PSC had increased odds of ICP compared to those without PSC (aOR 3.4, 95% CI 1.6‐7.0) (Figure 1). There were no significant differences in outcomes among those with PBC compared to those without PBC. The occurrence of stillbirth was too low to calculate.

**Conclusion**: Pregnancy outcomes in patients with PBC and PSC are overall favorable. Pregnant individuals with PBC and PSC had similar risk profiles compared to unaffected pregnancies, apart from a significantly increased risk of ICP in the PSC cohort. These findings support the use of ursodeoxycholic acid and antenatal surveillance in pregnancy given that the risks appear most associated with ICP.

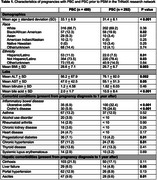

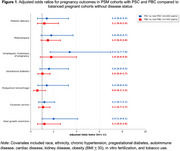

**3:30 PM – 5:00 PM**

## 1219 | **MR Radiomics and Surgical Outcomes in Pregnant Patients at Increased Risk for Placenta Accreta Spectrum**

Quyen N. Do^1^; Yin Xi^2^; Matthew A. Lewis^2^; David M. Owen^2^; Alesha M. White^3^; Naseem Uddin^2^; Baowei Fei^4^; Catherine Y. Spong^5^; Christina L. Herrera^5^

^1^UT Southwestern Medical Center, Dallas, TX; ^2^UT Southwestern Medical Center, UT Southwestern Medical Center, TX; ^3^University of Texas Southwestern Medical Center, Grand Prairie, TX; ^4^The University of Texas at Dallas, The University of Texas at Dallas, TX; ^5^University of Texas Southwestern Medical Center, Dallas, TX

**Objective**: To evaluate if placental radiomic features in pregnancies with increased risk of Placenta Accreta Spectrum (PAS) correlates with surgical outcome.

**Study Design**: We conducted a prospective MRI study of pregnancies with increased risk for PAS. Inclusion criteria consisted of first trimester ultrasound findings indicating low uterine segment implantation and a history of cesarean delivery. Eligible participants underwent MRI at two time points: 16w0d‐19w6d (Visit 1) and 24w0d‐27w6d (Visit 2). Volumetric placental regions of interest (ROIs) were manually delineated on T2W and apparent diffusion coefficient (ADC) images. Radiomic features, including first order and texture features, were extracted from the segmented placenta in accordance with the Image Biomarker Standardization Initiative (IBSI) guidelines using the PyRadiomics package. Medical records were reviewed to determine surgical outcomes, and patients were grouped according to the outcome of hysterectomy. Patient demographics were compared between the two outcome groups using T‐tests and Fisher's Exact test. Univariate receiver operating characteristic (ROC) curve analysis was performed for each radiomic feature to evaluate their predictive power for surgical outcomes. Statistical significance was determined using false discovery rate (FDR)‐adjusted p‐values, with an adjusted p‐value < 0.05 considered significant.

**Results**: Of 49 patients meeting inclusion criteria, 18 required cesarean hysterectomy (Table 1). Seventeen radiomic features from T2W and ADC images were significantly associated with hysterectomy after FDR correction, with the calculated placenta location in the uterus the most predictive (Table 2). Number of prior cesarean delivery, estimated blood loss, and pathologic PAS grading were significantly different between the two groups.

**Conclusion**: Radiomic features effectively predict surgical outcome in women at risk of PAS. These findings underscore the potential of MR‐based radiomics as a non‐invasive tool for risk stratification and preoperative planning in PAS.

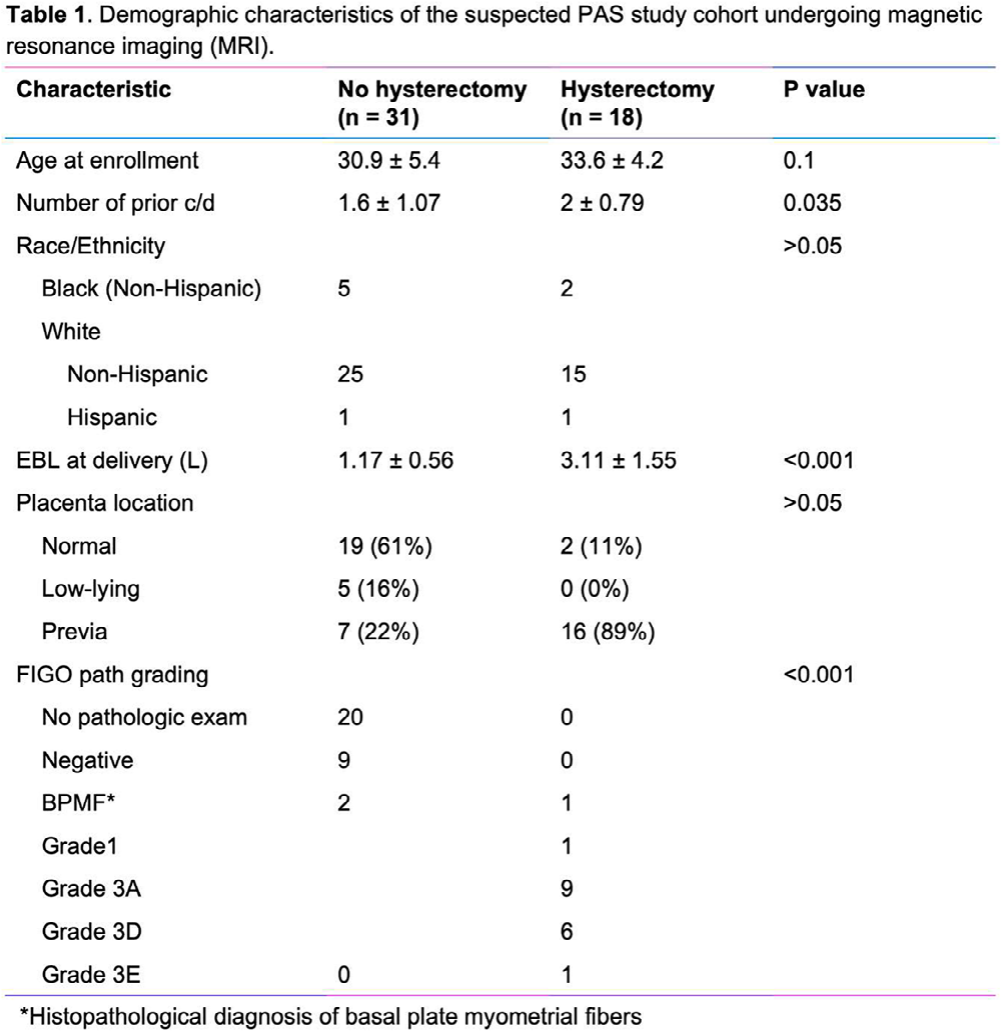

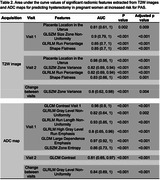

**3:30 PM – 5:00 PM**

## 1220 | **Novel Approach to a Computational Evaluation of the Waveform of Umbilical Artery Dopplers**

Rachel P. Gerber; David Krantz; Burton Rochelson

Northwell, New Hyde Park, NY

**Objective**: To develop a novel, quantitative method of evaluating waveform shapes of umbilical artery Dopplers (UAD) in fetuses with early diagnosis of fetal growth restriction (FGR). Our goal was to use shape analysis of waveforms to better predict which fetuses will ultimately develop Doppler abnormalities.

**Study Design**: This was a test of concept study, evaluating the UAD waveforms in 10 patients diagnosed with early onset FGR, 5 of which were diagnosed with absent end diastolic flow during pregnancy, 5 of which never developed Doppler abnormalities. The image recorded of the UAD wave from at the time of diagnosis of FGR was analyzed in two ways: the slope ratio and the descending curve index (DCI). The curves generated during ultrasound exam were digitized using WebPlotDigitizer 5.2 (Automeris LLC, California, USA). Three wave forms within each image were averaged to account for variability. The slope ratio is the ratio of the descending slope to the ascending slope. The DCI is the ratio of the area under the descending curve to the area if the velocity decreased at a constant rate (Figure 1). Calculations and statistical analysis were performed in R 4.4.2.

**Results**: On average, the 5 patients who developed absent end diastolic flow during the pregnancy, had a lower slope ratio and DCI. Analysis of variance of the 3 readings per subject, showed that the between‐subject variance represented 53% and 17% of total variance for slope ratio and DCI, respectively.

**Conclusion**: UAD are widely used as a component of fetal surveillance in pregnancies complicated by FGR. Current methods of analysis only include systolic and diastolic velocities, and the shape of the Doppler wave form remains unanalyzed. There may be significant information in further evaluation of the shape of the curve between systolic and diastolic velocities. We hypothesize that detailed quantitative waveform analysis at the time of diagnosis of FGR may improve prediction of those fetuses who will develop Doppler abnormalities. Additional studies on determining predictive accuracy and improved measurement repeatability are planned.

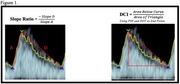

**3:30 PM – 5:00 PM**

## 1221 | **Perinatal Mental Health on TikTok: What Does Medical Content Look Like?**

Rachel L. Wiley^1^; Sierra Adkins^2^; Shayna Weinstein^3^; Dana R. Canfield^4^; Jerasimos Ballas^1^; E. Nicole Nicole Teal^1^; Minhazur R. Sarker^1^

^1^Division of Maternal Fetal Medicine, Department of Obstetrics, Gynecology and Reproductive Sciences, University of California San Diego, San Diego, CA; ^2^Kirk Kerkorian School of Medicine at the University of Nevada, Las Vegas, Las Vegas, NV; ^3^New York Medical College, Valhalla, NY, Valhalla, NY; ^4^Department of Obstetrics & Gynecology  , University of Washington, Seattle, WA, Seattle, WA

**Objective**: Social media is widely used for information, especially on sensitive topics like perinatal mental health; however, the educational value is unclear.

**Study Design**: This was a cross‐sectional analysis of publicly available TikTok videos using the hashtags #postpartumdepression (#PPD), #maternalmentalhealth (#MMH), and #postpartumanxiety (#PPA). We used the Apify TikTok Data Extractor to collect top‐performing videos by engagement. Videos were assessed using the Global Quality Score (GQS), a validated tool rating health content from 1 (low, patient use discouraged) to 5 (high, medically useful). Additional information on video elements and medical concepts was collected. Videos making medical claims beyond personal experience were further evaluated using the mDISCERN tool, which assesses the reliability of online health content.

**Results**: Of the 192 #PPD, 162 #PPA, and 216 #MMH videos identified, 175 (91.2%), 159 (98.1%), and 110 (50.9%) were relevant, respectively (Table 1). Most videos were created by women (70.7%) without professional credentials (74.1%) and shared personal experiences (71.8%). Common features included catchy music and visual enhancements (Table 2). Median GQS scores were 3 (interquartile range of 2–3 for #PPD and #PPA; 2–4 for #MMH), suggesting limited to potentially misleading educational value. Of those that addressed medical topics, the majority were supportive of diagnosis and treatment (Table 2). Of the 57 videos making medical claims, 57.9% (*n* = 33) accurately described symptoms, and 47.4% (*n* = 27) included accurate treatment information. While only 8.3% of all videos were commercial in nature, 28.1% of those making medical claims were associated with product promotion.

**Conclusion**: Engaging qualified medical voices are needed in the perinatal mental health space, as the educational quality of maternal mental health content on TikTok is currently limited, with a risk of misinformation and commercialization. The dominance of personal narratives may indicate a social benefit other than education, which needs further investigation.

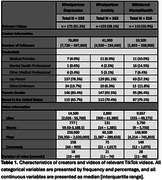

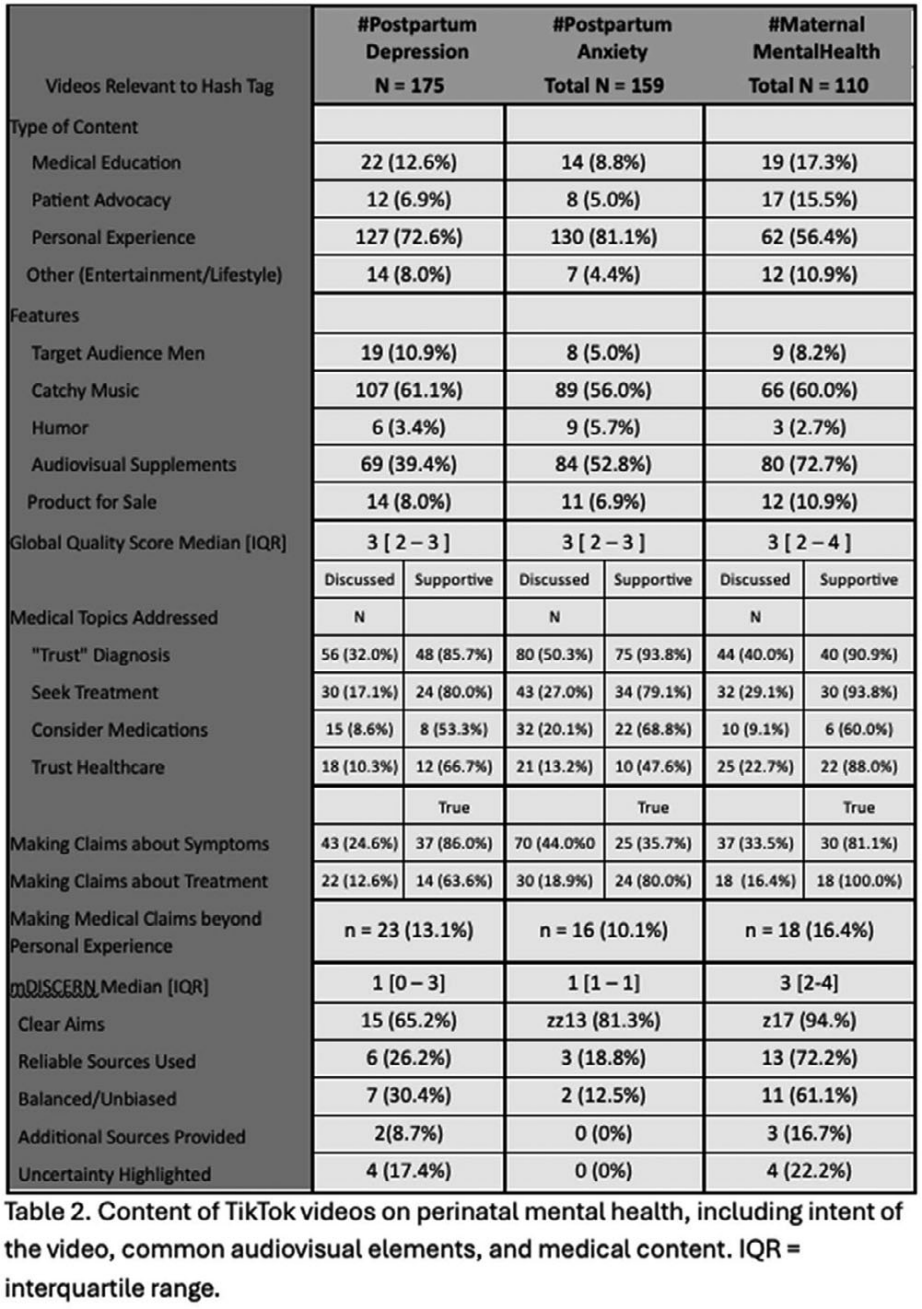

**3:30 PM – 5:00 PM**

## 1222 | **Measuring What Matters: The Clinical Utility of Quantitative vs. Estimated Blood Loss in Postpartum Hemorrhage**

Raina A. Meka^1^; Bridget C. Huysman^2^; Jeannie C. Kelly^2^; Nandini Raghuraman^3^; Roxane Rampersad^3^; Megan L. Lawlor^3^; Antonina I. Frolova^3^

^1^Washington University School of Medicine, Washington University School of Medicine/ St. Louis, MO; ^2^Washington University School of Medicine, Washington University School of Medicine, MO; ^3^Washington University School of Medicine, St. Louis, MO

**Objective**: Postpartum hemorrhage (PPH) is a major contributor to maternal morbidity and mortality worldwide. Imprecise estimation of blood loss significantly contributes to preventable PPH‐related deaths. Although quantitative blood loss estimation (QBL) is more accurate than visual estimation (EBL), its impact on adverse clinical outcomes remains unclear. This study compares the predictive value of EBL and QBL for postpartum transfusion, the leading cause of maternal morbidity in the United States.

**Study Design**: We conducted a retrospective cohort study of all deliveries at a tertiary care center between January 2024 and June 2025. Only individuals with both EBL and QBL recorded were included. The correlation between EBL and QBL was assessed using Spearman correlation. Receiver operating characteristic (ROC) curves were used to compare the predictive value of EBL and QBL for postpartum transfusion. Subgroup analyses were performed based on mode of delivery.

**Results**: Among 5544 individuals, 3526 (63.6%) met inclusion criteria. Of those included, 145 (4.1%) received a transfusion during admission. The median EBL was 350 mL (IQR 200‐700 mL), and the median QBL was 435 mL (IQR 237‐800 mL). There was a strong correlation between EBL and QBL, with Spearman's rho of 0.86 (*p* < 0.001; Figure 1). PPH defined as blood loss >1000 mL was identified significantly more frequently by QBL than by EBL (15.7% vs 11.7%, *p* < 0.001). However, EBL and QBL did not differ in their ability to predict transfusion (area under the curve (AUC) 0.90 for both, *p* = 0.52; Figure 2). Subgroup analysis revealed consistent predictive value of QBL and EBL across delivery modes (AUC 0.91 for vaginal delivery for both, *p* = 0.37; AUC 0.81 for EBL and 0.80 for QBL for cesarean section, *p* = 0.81)

**Conclusion**: While QBL identifies PPH more frequently than EBL, both methods are similarly strong predictors of the need for transfusion in the postpartum period. Further research is needed to evaluate the clinical utility of QBL versus EBL in reducing maternal morbidity, assessing additional maternal outcomes, and exploring the cost implications of implementing QBL.

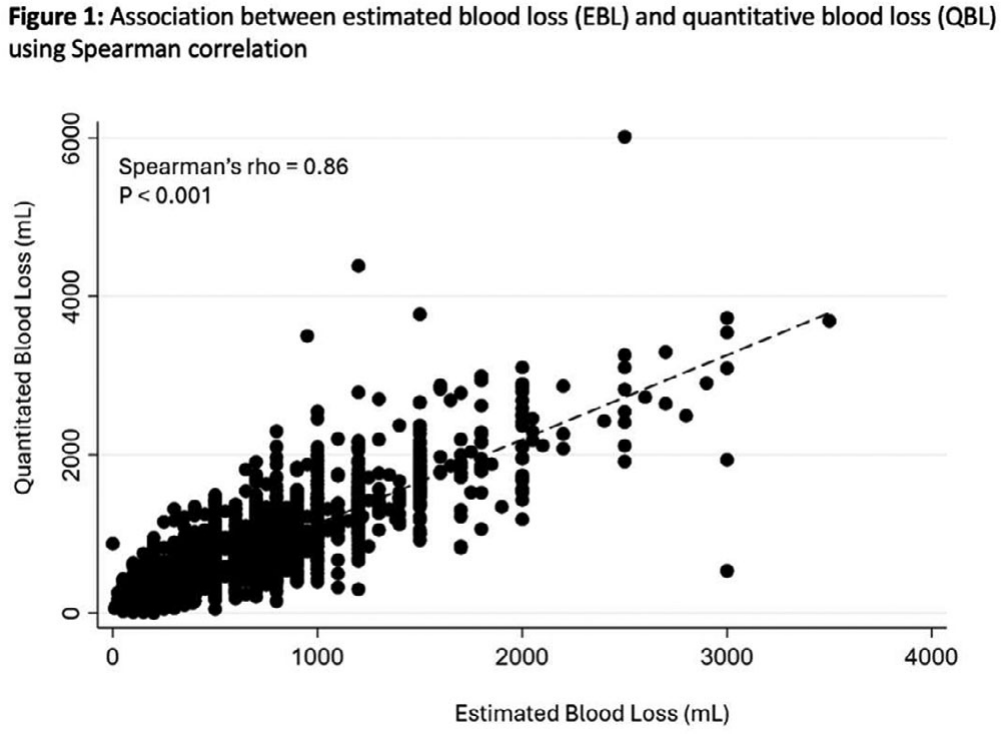

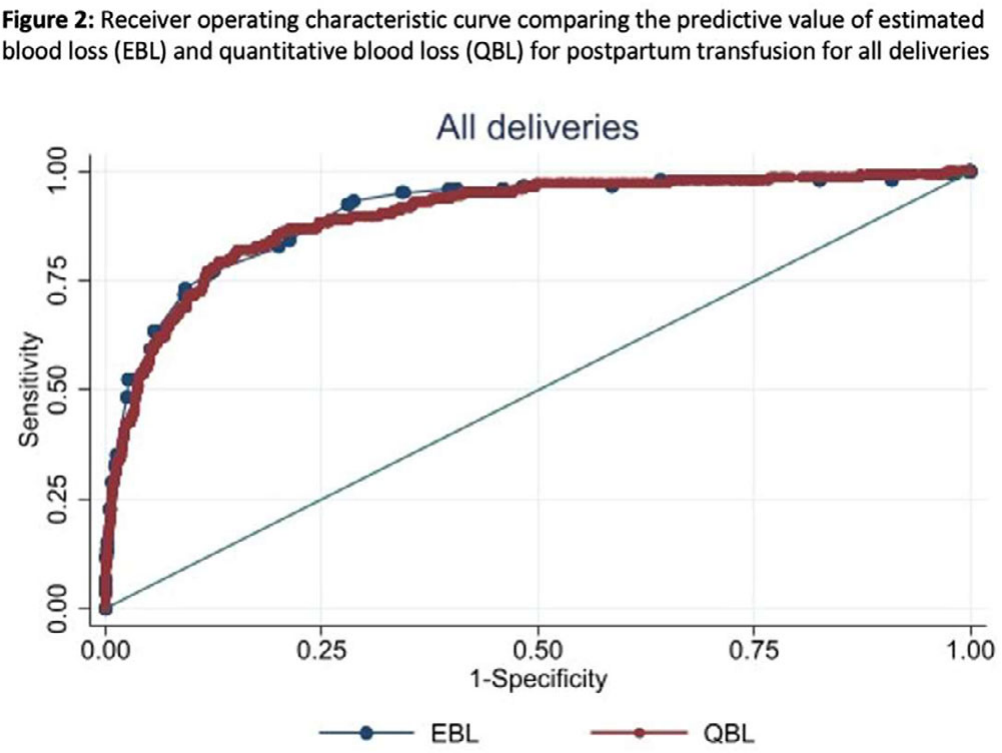

**3:30 PM – 5:00 PM**

## 1223 | **Venovenous Anastomoses and Laser for Twin‐Twin Transfusion Syndrome (TTTS): A Sequential Trial Subanalysis**

Ramen H. Chmait^1^; Lisa M. Korst^2^; Arlyn S. Llanes^1^; Andrew H. Chon^3^; Martha A. Monson^4^; Eftichia V. Kontopoulos^5^; Ruben A. Quintero^5^

^1^Keck School of Medicine of USC, University of Southern California, Keck School of Medicine of USC, University Of Southern California, CA; ^2^Childbirth Research Associates, North Hollywood, CA; ^3^Oregon Health & Science University, Portland, OR; ^4^Intermountain Healthcare, Salt Lake City, UT; ^5^The Fetal Institute, Miami, FL

**Objective**: To assess the role of venovenous anastomoses (VV) in donor twin survival after laser surgery for twin‐twin transfusion syndrome (TTTS).

**Study Design**: This is a post hoc analysis of data from the Sequential Trial, a randomized controlled trial (RCT) in which TTTS patients were randomized to sequential vs selective laser surgery. A nested trial randomized patients with superficial anastomoses (ie, arterioarterial [AA] or VV) to ablation of these first (before the principal procedure) vs last (at the conclusion of the laser surgery). Here, in bivariate analyses, we examined donor survival in those with VV vs the control group that had no superficial anastomoses (“No SA,” ie, only arteriovenous communications). We identified patient and surgical characteristics predictive of donor survival for inclusion in multiple logistic regression models, including testing the nested RCT assignment for those with VV to the “Laser First” vs the “Laser Last” groups.

**Results**: Of the 642 RCT patients, 64 (10%) had at least 1 VV (range 1‐3). Donor live birth in the No SA vs VV groups, respectively, was 421/466 (90.3%) vs 48/64 (75.0%), *P* =  .001. Of the 64 patients with a VV, 28 (43.8%) were randomized to the Laser First group and 36 (56.3%) to the Laser Last group. The proportions of donor twins born alive for the No SA, Laser First, and Laser Last groups, respectively, were 421/466 (90.3%) vs 23/28 (82.1%) vs 25/36 (69.4%), *P*<  .001. In a multiple logistic regression model for donor survival, the VV Laser Last group had a lower odds of survival (aOR 0.24 [0.10‐0.58], *P* =  .002) vs the No SA group (Table). A similar model for donor survival that split the VV group into VV only and VV+AA showed that both Laser Last groups (VV only and VV+AA) had lower odds of donor survival (aOR 0.23 [0.06‐0.90], *P* =  .035 and aOR 0.24 [0.08‐0.74], *P* = 0.013, respectively) vs the No SA group (Table).

**Conclusion**: Compared with TTTS patients without superficial anastomoses, patients with VV had worse donor twin survival if the superficial anastomoses were lasered last, at the conclusion of the surgery.

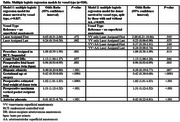

**3:30 PM – 5:00 PM**

## 1224 | **Cervical Shortening After Normal Midtrimester Screening: Clinical Implications for Preterm Birth Prediction**

Raneen Abu Shqara^1^; Maisa Assy^2^; Lior Lowenstein^2^; Maya Frank Wolf^2^

^1^Galilee Medical Center, Nahariya, HaZafon; ^2^Galilee Medical Center, Naharyia, HaZafon

**Objective**: Cervical length (CL) screening in the midtrimester is an established method for predicting spontaneous preterm birth (sPTB). However, the clinical implications of cervical shortening diagnosed after a normal midtrimester screening remain unclear. We aimed to examine whether cervical shortening identified at 24.0–34.0 weeks gestation is associated with an increased risk of sPTB, and whether obstetric history modifies this risk.

**Study Design**: This retrospective cohort study included 500 singleton pregnancies with normal midtrimester CL who were later diagnosed with cervical shortening (<25 mm) at 24.0–34.0 weeks gestation at a tertiary medical center between March 2020 and May 2025. Patients were stratified into three groups: CL <10 mm, 11–15 mm, and 16–25 mm. The primary outcome was sPTB <37 weeks. Multivariable logistic regression and receiver operating characteristic (ROC) curve analyses were used to evaluate predictive performance. sPTB rates were further analyzed by obstetric history and gestational age at diagnosis.

**Results**: sPTB <37 weeks occurred in 75.0% of patients with CL <10 mm, 52.5% with CL 11–15 mm, and 25.0% with CL 16–25 mm (*p* < 0.001). In multivariable models, both CL < 10 mm and 11–15 mm were independently associated with sPTB (adjusted odds ratios [aOR] 8.19 and 3.19, respectively; *p* < 0.001 for both). Among patients with a CL of 21–25 mm, sPTB rates differed significantly by obstetric history: 26.9% in those with a history of sPTB, 21.8% in nulliparas, and 12.8% in those with a history of term birth (*p* = 0.041) (Figure 1). ROC analysis identified optimal gestational age‐specific CL cutoffs of 22 mm (area under the curve [AUC] 0.69) at 24.0–27.6 weeks, 20 mm (AUC 0.62) at 28.0–31.6 weeks, and 18 mm (AUC 0.72) at 32.0–34.0 weeks. A significant increase in the rate of sPTB was observed below these cutoff values (Figure 2).

**Conclusion**: Cervical shortening after a normal midtrimester screening was associated with sPTB. Obstetric history modified this risk, particularly in patients with less severe shortening.

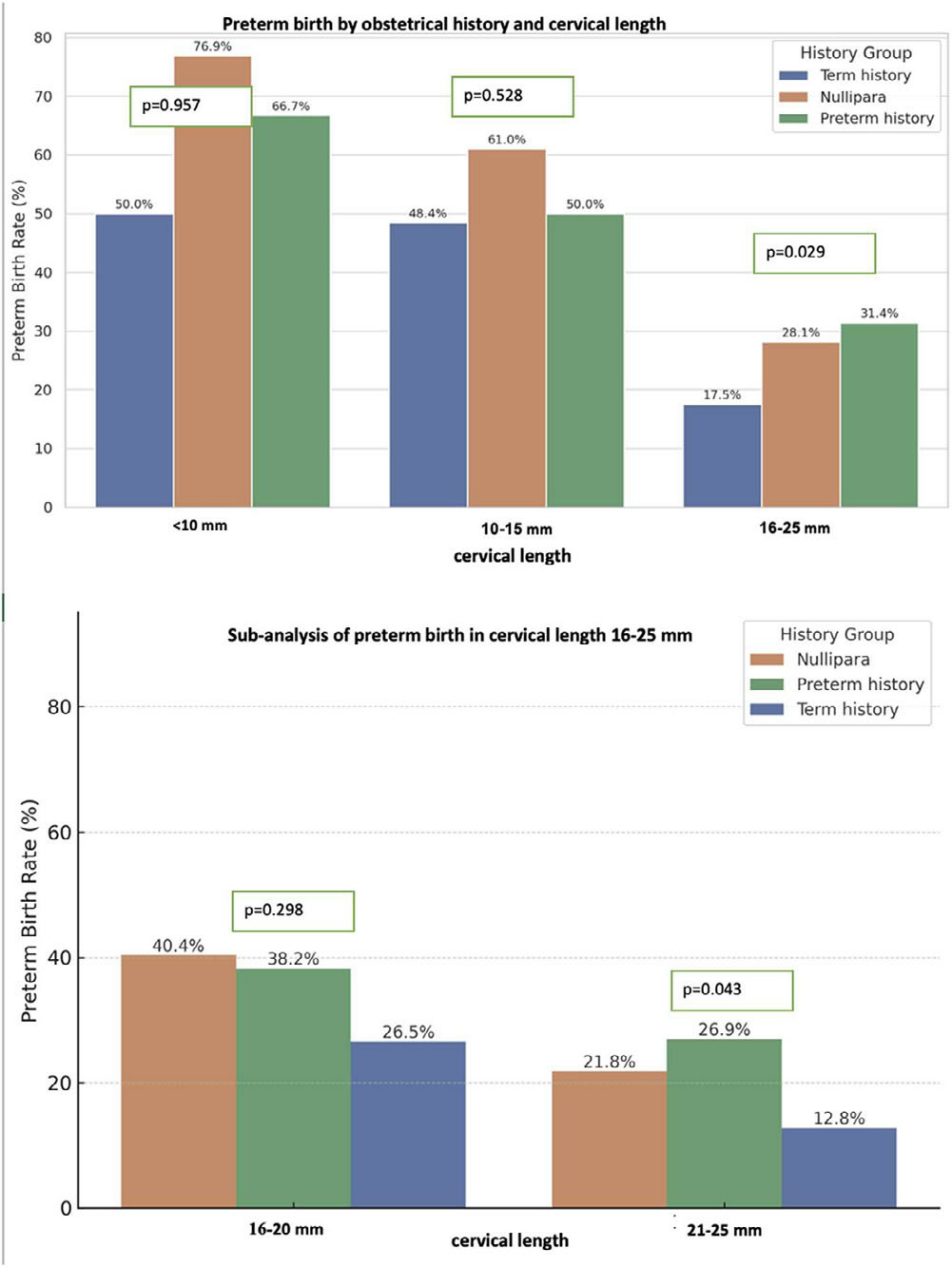

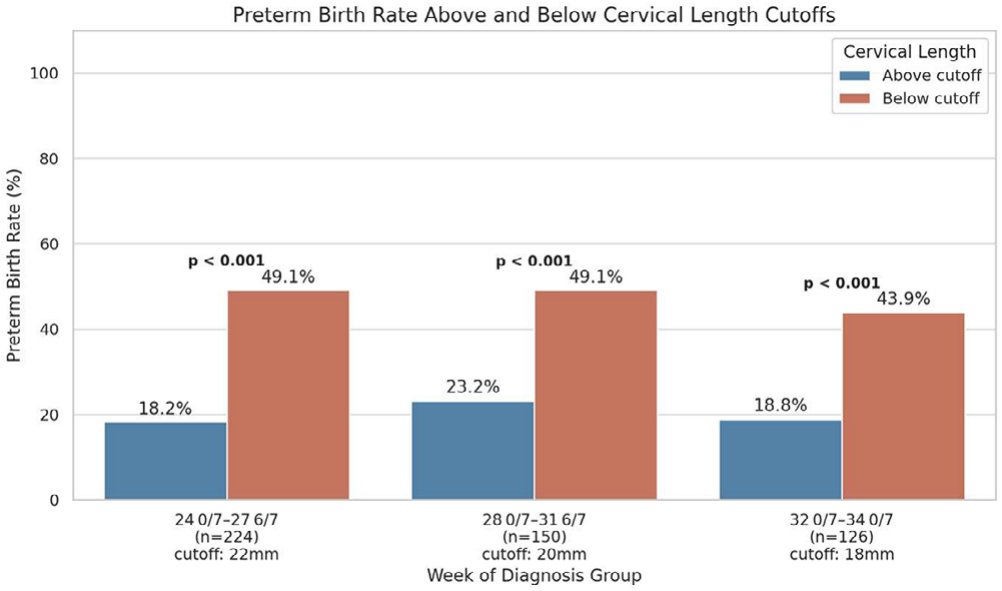

**3:30 PM – 5:00 PM**

## 1225 | **Ultra‐Processed Food Consumption Is Associated With Higher Risk of Adverse Pregnancy Outcomes**

Raven Hall^1^; Suzette Rosas‐Rogers^2^; Amy Y. Pan^3^; Melodee Liegl^1^; Alyssa M. Hernandez^3^; Anna Palatnik^2^

^1^Medical College of Wisconsin, Milwaukee, WI; ^2^Medical College of Wisconsin, MILWAUKEE, WI; ^3^Medical College of Wisconsin, Medical College of Wisconsin, WI

**Objective**: To examine the association between ultra‐processed food (UPF) consumption and the occurrence of adverse pregnancy outcomes (APOs).

**Study Design**: This was a secondary analysis of the Nulliparous Pregnancy Outcomes Study: Monitoring Mothers‐to‐Be. Patients were excluded if they experienced pregnancy loss <20 weeks or had missing data from the Food Frequency Questionnaire (FFQ). Foods from the FFQ were classified using the NOVA scale, which groups items by processing level: category 1 foods are unprocessed/minimally processed, category 2 contains processed culinary ingredients (e.g. oils, butter, sugar), category 3 contains processed foods (e.g. canned vegetables, cheeses, bread) and category 4 comprised of ultra‐processed foods (industrial ready‐to‐eat items with additives). The daily percentage of UPF intake was calculated by dividing kilocalories of category 4 foods by total daily energy intake. Univariate and multivariable analyses explored the relationship between increasing UPF intake and APOs, including preterm birth, preeclampsia, gestational diabetes (GDM), and small for gestational age (SGA).

**Results**: A total of 6041 participants were included in the analysis. The sample was predominantly White (75.5%) and not Hispanic (83.1%), and a majority had commercial insurance (71.8%). UPF comprised an average of 54% (±14%) of the daily total intake among participants (Table 1). Increasing total UPF intake was significantly associated with preterm birth, SGA, and preeclampsia (Table 2). While UPF intake was not significantly associated with overall GDM incidence, it was significantly associated with a higher risk of GDMA2 among all patients with GDM. After adjusting for education level and chronic hypertension, every 10% increase in daily UPF intake was significantly associated with preeclampsia (aOR 1.08, 95% CI 1.02‐1.14) and GDMA2 (aOR 1.25, 95% CI 1.02‐1.52).

**Conclusion**: On average, more than half of participants’ daily diet was composed of UPF. Increasing UPF intake was associated with higher rates of preeclampsia and GDMA2.

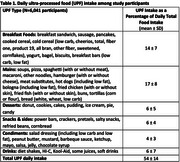

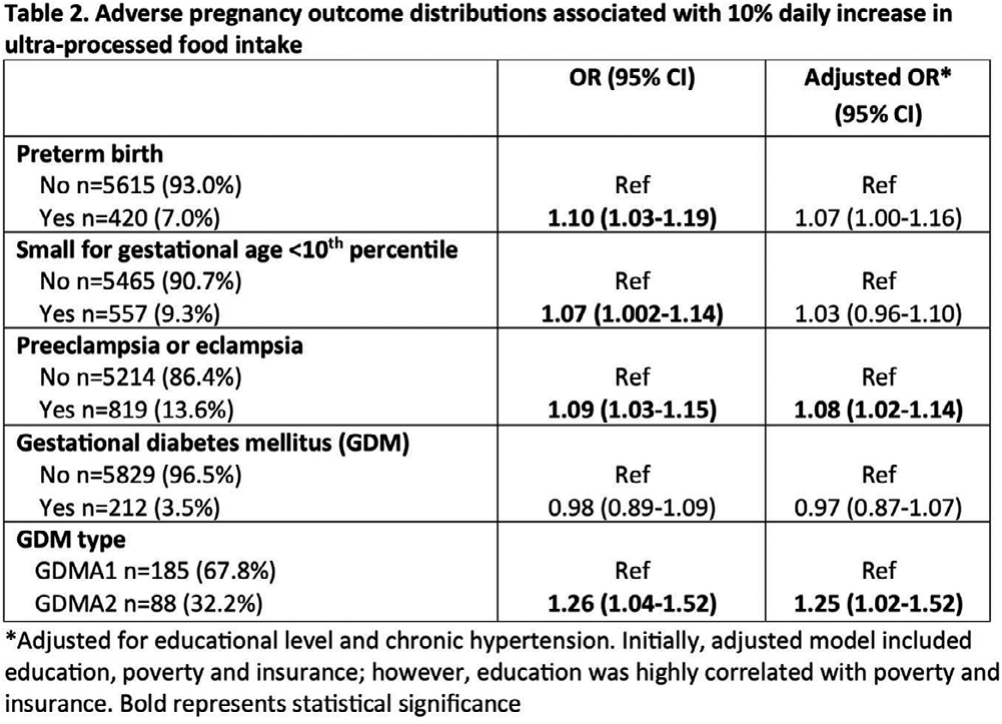

**3:30 PM – 5:00 PM**

## 1226 | **Weighing the Risks: Maternal Morbidity and Neonatal Outcomes After Bariatric Surgery**

Rebecca Boughton^1^; Jenna Mullen^2^; Hilary Devine^3^; Arthi Subramanian^3^; Ciara McNulty^3^; Patrick Dicker^4^; Zara Molphy^5^; Sean Daly^6^; Sharon Cooley^7^

^1^Department of Obstetrics and Gynaecology, Royal College of Surgeons Ireland, Dublin 1, Dublin; ^2^The Royal College of Surgeons in Ireland, Dublin, Dublin; ^3^The Rotunda Hospital Dublin, Ireland, The Rotunda Hospital, Dublin, Ireland, Dublin; ^4^Department of Obstetrics and Gynaecology & Department of Public Health and Epidemiology, Royal College of Surgeons in Ireland, Royal College of Surgeons in Ireland, Dublin; ^5^Royal College of Surgeons in Ireland, Dublin, Dublin; ^6^Rotunda Hospital, Dublin, Dublin; ^7^Rotunda Hospital Dublin & Royal College of Surgeons in Ireland, Rotunda Hospital Dublin & Royal College of Surgeons in Ireland, Dublin

**Objective**: Obesity is a growing crisis and bariatric surgery is a proven, effective solution. However, there is limited international guidance on management of pregnancy following bariatric surgery. This study aimed to evaluate outcomes in this cohort.

**Study Design**: This retrospective cohort study included 119 patients with a history of bariatric surgery who delivered between January 2022 ‐ July 2024 at Europe's largest obstetric unit responsible for 9000 births annually. Bariatric surgery included Gastric Band, Sleeve Gastrectomy and Gastric Bypass. Data were collected from electronic patient records and described.

**Results**: The most commonly performed procedure was a Sleeve Gastrectomy (74%, *n* = 88). Nearly half (48%, *n* = 57) of pregnancies were booked within 18 months post‐surgery. Prenatal complications included high rates of Gestational Diabetes (77%, *n* = 92) and Hyperemesis Gravidarum (26%, *n* = 30). Nutritional deficiencies were common with 78% (*n* = 82) of patients’ vitamin D deficient at booking. 36% (*n* = 36) of patients had low ferritin levels worsening to 69% (*n* = 69) by 28 weeks' gestation.

58% (*n* = 51) of patients had induction of labor compared to a baseline rate of 39% at our institution. Caesarean section rate was 42% (*n* = 50) of which 21% (*n* = 25) were performed as an emergency (Table 1). Birth centiles progressively decreased with more invasive bariatric surgical procedures: gastric band > sleeve > bypass. Birthweight was significantly lower following gastric bypass compared to gastric band (*p* = 0.046, Wilcoxon test). (Figure 1).

**Conclusion**: This study highlights the importance of pre‐operative counselling and post‐operative nutritional management in this demographic. There is a clear association of bariatric surgery on maternal morbidity shown by the increased rates of Gestational Diabetes and Hyperemesis Gravidarum. Additionally, the observed differences in birthweight centiles among surgery types suggest that surgical choice may influence neonatal outcomes. This warrants further investigation into the long‐term implications of bariatric surgery on maternal and fetal health.

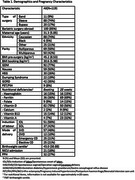

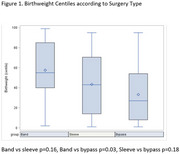

**3:30 PM – 5:00 PM**

## 1227 | **Association of Immediate Neonatal Morbidity With Long‐Term Pulmonary and Neurocognitive Outcomes**

Rebecca Horgan

On behalf of the Eunice Kennedy Shriver National Institute of Child Health and Human Development (NICHD) Maternal‐Fetal Medicine Units (MFMU) Network, Bethesda, MD

Eastern Virginia Medical School at Old Dominion University, Norfolk, VA

**Objective**: Studies addressing preterm birth often focus on immediate neonatal outcomes but there are limited data on their association with long‐term outcomes. Our objective was to evaluate the relationship between neonatal morbidity measures and pulmonary and neurocognitive outcomes in children.

**Study Design**: Secondary analysis of a prospective follow‐up study of children (median age 7 years) of mothers at high risk for late preterm birth who were randomized to corticosteroids versus placebo. Children with pulmonary and neurocognitive outcomes were included. Immediate neonatal resuscitation, defined as any intervention in the first 30 minutes other than blow‐by oxygen, as well as the neonatal morbidity index (NMI) with and without NICU length of stay (LOS) were evaluated as exposures. The NMI is a validated composite score based on the number of specific events: respiratory distress syndrome, bronchopulmonary dysplasia, grade III/IV intraventricular hemorrhage, periventricular leukomalacia, proven sepsis, or necrotizing enterocolitis. The primary outcomes were: (1) pulmonary impairment (abnormal spirometry and/or asthma diagnosis with medication use), and (2) neurocognitive delay (DAS‐II General Conceptual Ability score <85). Associations were evaluated using relative risk (RR) and Cochran‐Armitage trend tests, with a sensitivity analysis among children born preterm.

**Results**: Among 1194 children with a median GA at delivery of 36 weeks, NMI ≥ 1 (see Table footnote) was not associated with long‐term pulmonary or neurocognitive impairment. However, NMI incorporating NICU LOS was associated with neurocognitive impairment at the highest NMI score (3) and with progressively increasing risk of pulmonary impairment (Tables 1 & 2). Need for immediate neonatal resuscitation was not associated with neurocognitive impairment (Table 2). Sensitivity analysis of children born preterm showed similar findings.

**Conclusion**: The NMI incorporating NICU LOS, but not NMI alone, is associated with long‐term pulmonary, but not neurocognitive, impairment, supporting its potential as a surrogate predictor for pulmonary outcomes.

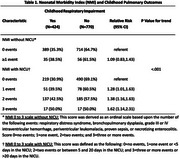

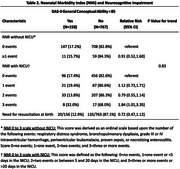

**3:30 PM – 5:00 PM**

## 1228 | **Assessment of Machine Learning Models for First‐Trimester Detection of Fetal Congenital Heart Defects**

Rebecca Horgan^1^; Jonathan Colen^2^; Daniel Lersch^3^; Lana El‐Kassis^1^; Achyut Paudel^4^; Alfred Abuhamad^5^; Heather Richter^6^; Malachi Schram^3^

^1^Eastern Virginia Medical School at Old Dominion University, Norfolk, VA; ^2^Old Dominion University, Joint Institute on Advanced Computing for Environmental Studies, Norfolk, VA; ^3^Thomas Jefferson National Accelerator Facility, Newport News, VA; ^4^Old Dominion University, Joint Institute on Advanced Computing for Environmental Studies., Norfolk, VA; ^5^Eastern Virginia Medial School at Old Dominion University, Norfolk, VA; ^6^Hampon Roads Biomedical Research Consortium, Norfolk, VA

**Objective**: To evaluate machine learning (ML) models for early detection of congenital heart defects (CHD) using first‐trimester ultrasound.

**Study Design**: We retrospectively analyzed de‐identified grayscale and color Doppler ultrasound cine loops obtained between 11 to 13+6 weeks’ gestation from our institution's database between 2018 and 2024. The dataset included 8820 clips from 927 patients, 56 of whom had confirmed CHD. To evaluate the potential of ML for early CHD detection, we tested three model types. The first approach used visual pattern analysis, extracting key imaging features from each clip to train a classification model. The second involved deep learning models trained directly on the raw ultrasound images to identify abnormalities. The third applied a general‐purpose medical AI model (MedGemma), which interpreted each clip using knowledge derived from a broad range of medical imaging data. Each model produced a final prediction for each patient by summarizing results across all available clips. All models were evaluated on patients not used during training to ensure unbiased performance.

**Results**: The best‐performing model was a support vector machine (SVM) that analyzed key visual patterns from each ultrasound clip. It achieved an area under the ROC curve (AUC) of 0.84. At 90% specificity, the model detected 62% of CHD cases, with sensitivity increasing to 81% at 70% specificity (Table 1, Figure 1). In comparison, deep learning models trained directly on the ultrasound images underperformed, with AUC values up to 12% lower. The general‐purpose medical foundation model (MedGemma) demonstrated poor accuracy, misclassifying 74% of normal cases as CHD, underscoring the limitations of non‐specialized AI in early fetal cardiac screening (Table 1, Figure 1).

**Conclusion**: Machine learning models demonstrate potential as adjunctive tools for the early detection of CHD. With further optimization and validation in larger, diverse populations, ML‐based approaches may enhance prenatal screening protocols.

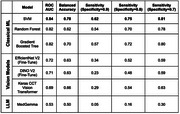

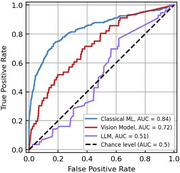

**3:30 PM – 5:00 PM**

## 1229 | **Cardiometabolic Health Indices as Predictors of Gestational Diabetes**

Rebecca Purvis^1^; Jacklyn Locklear^2^; Destiny Pearson^3^; Callie Tucker^4^; John Michael Mills^5^; Jaclyn Van Nes^6^; Nikki B. Zite^2^; Callie F. Reeder^7^; Kimberly B. Fortner^8^; Jill M. Maples^9^

^1^University of Tennessee Health Science Center College of Medicine‐ Knoxville, Knoxville, TN; ^2^University of Tennessee Graduate School of Medicine, Knoxville, TN; ^3^Baptist Memorial Hospital‐Memphis Department of OB/GYN, Memphis, TN; ^4^Univeristy of Tennessee‐ Knoxville, Knoxville, TN; ^5^University of Kentucky College of Medicine, Bowling Green, KY; ^6^University of Tennesse Medical Center‐ Knoxville Graduate School of Medicine, Knoxville, TN; ^7^UTHSC UTGSM Knoxville, Knoxville, TN; ^8^University of Tennessee College of Medicine‐ Knoxville, Knoxville, TN; ^9^University of Tennessee Health Science Center COM Knoxville, Knoxville, TN

**Objective**: The rate of gestational diabetes mellitus (GDM) diagnosis in pregnancy is rising and traditional models only quantify glucose values for diagnosis without considering insulin resistance (IR) or other markers of cardiometabolic (CM) health. Multiple markers of IR and CM health exist, including triglyceride glucose index (TyG), Homeostatic Model Assessment for Insulin Resistance (HOMA‐IR), and Adipose Tissue Insulin Resistance Index (Adipo‐IR), in addition to BMI, blood lipids, and lactate. As insulin resistance itself likely plays a large role in development of GDM but also pregnancy and neonatal outcomes, we sought to understand if an association exists between these markers and development of GDM in our population.

**Study Design**: This cohort was derived from two prospective cohort studies of metabolic health in pregnant individuals through 22 weeks gestation at a single regional academic medical center. Fasting venous blood draws were performed. Differences between groups (those that developed GDM and those that did not) were assessed using T‐test and Mann‐Whitney U, as appropriate, and binary logistic regression was used to assess which factors predicted subsequent GDM diagnosis.

**Results**: A total of 52 individuals were included in our analysis, with 22 (42.3%) developing GDM during pregnancy (Table). Maternal age, BMI, TyG, lactate, insulin, triglycerides, HOMA‐IR, free fatty acids, VLDL, and Adipo‐IR were all significantly different between those who did and did not develop GDM.  Fasting glucose and individual lipid parameters were not significantly different between those who did and did not develop GDM. When controlling for maternal age and pre‐pregnancy BMI, TyG and adipoIR both remained significant predictors of GDM (*p* < 0.01).

**Conclusion**: Despite a relatively small sample, TyG and other CM indices were better predictors of GDM than early fasting glucose alone, even when controlling for maternal age and BMI,. Future studies with a larger sample should focus on specific metabolic indices and their thresholds for risk assessment of developing GDM.

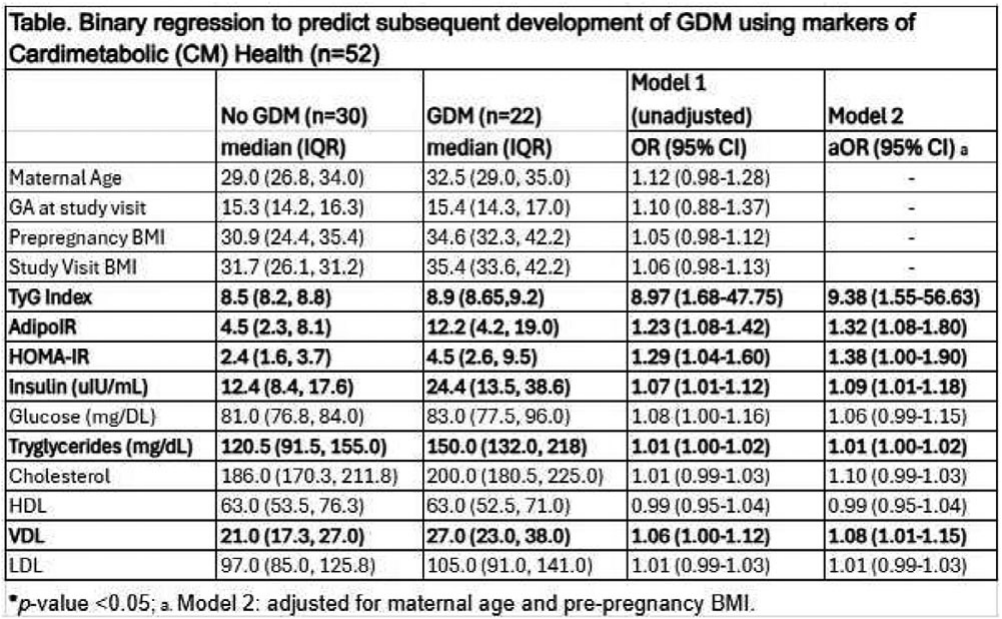

**3:30 PM – 5:00 PM**

## 1230 | **Evaluating Anti‐MBG Antibody (H3L2) As a Therapeutic Agent and Marinobufagenin As a Biomarker in Preeclampsia**

Regan Rivers^1^; Michelle N. Harris^2^; Audrey Wise^1^; Ram R. Kalagiri^3^; Jessica C. Ehrig^4^; Kelsey R. Kelso^2^; Niraj Vora^5^; Diana Villazana‐Kretzer^5^; Mohammad N. Uddin^6^; Ahmed Pantho^7^; Thomas Kuehl^8^

^1^Baylor Scott and White, Baylor Scott and White/ Temple, TX; ^2^Baylor Scott & White Health ‐ Baylor College of Medicine, Temple, TX; ^3^Baylor Scott and White Health, Te, TX; ^4^Baylor Scott & White Medical Center‐Temple, Temple, TX; ^5^Baylor Scott & White Medical Center, Temple, TX; ^6^Texas A&M Health University College of Medicine, Temple, TX; ^7^Orion Institute for Translational Medicine, Temple, TX; ^8^Texas A&M College of Medicine, Bryan, TX

**Objective**: Preeclampsia (preE) affects 3‐10% of pregnancies and is a leading cause of maternal and fetal morbidity and mortality. Recent data support the involvement of marinobufagenin (MBG), a cardiotonic steroid, in preE.

We aimed to understand the effects of anti‐MBG antibody (H3L2) on outcomes in a PreE rat model, and to evaluate trends in MBG levels in PreE patients to understand its role and timing as a biomarker.

**Study Design**: Six groups of pregnant rats (*n* = 10 per group) were studied: 1) Normal pregnant rats (NP), 2) Pregnant rats given Anti‐MBG H3L2 intraperitoneally daily from gestation day (GD) 10‐20, 3) Pregnant rats injected with DOCA (12.5 mg initially, followed by weekly injections of 6.5 mg) and given 0.9% saline as drinking water, 4) DOCA/saline‐treated rats also given anti‐MBG in intraperitoneal doses twice weekly of 10 mg/kg, or 5) 2 mg/kg, or 6) 0.5 mg/kg, from GD10‐20. Blood pressure (BP) and plasma samples were analyzed. Results are shown in Figure 1. A total of 76 women were recruited from the Obstetrical service at Baylor Scott and White Hospital in the first trimester and serum MBG was analyzed at four time points throughout pregnancy. Serum levels of MBG in the 21 patients who developed PreE were compared to levels in those who did not. Results are shown in Table 1.

**Results**: The anti‐MBG antibody normalized BP, proteinuria, and the number of abnormal pups in DOCA‐treated rats in a dose‐dependent manner. Serum levels of marinobufagenin (MBG) were significantly elevated in women who developed PreE, with notable rise observed in the second trimester. These findings suggest anti‐MBG therapy could mitigate PreE symptoms and MBG could be useful as a biomarker for the disease.

**Conclusion**: The anti‐MBG antibody (H3L2) demonstrated dose‐dependent efficacy in normalizing BP and mitigating the effects of DOCA treatment in the rat model. Elevated MBG levels in women with PreE suggest its utility as a serum biomarker for the disease. These findings support the potential of anti‐MBG therapy as a targeted treatment for PreE.

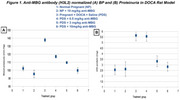

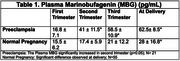

**3:30 PM – 5:00 PM**

## 1231 | **App‐Based Immediate Discharge vs. Standard‐Discharge Process: Effects on Time to Departure, Safety, and Satisfaction**

Rima Nassra^1^; Marian Matanis^1^; Nidal Haj^1^; Tasnim Asli^1^; Hiyam Yamin^1^; Yuri Perlitz^1^; Enav Yefet^2^

^1^Tzafon Medical Center, Poriya, HaZafon; ^2^Tzafon medical center, Poriya, HaZafon

**Objective**: Maternal and fetal medicine units are often overwhelmed. As medical assessment is prioritized over preparing discharge letters, the resulting delay leads to patient dissatisfaction, increased costs, and staff burden. In our unit, a mobile health application (app) provides access to lab results and discharge letters, allowing immediate discharge without waiting for a printed letter.

We aimed to compare app‐based immediate discharge with the standard discharge process regarding time to departure, safety, and patient satisfaction.

**Study Design**: A randomized controlled trial conducted in 2025 at a university‐affiliated medical center. Hospitalized pregnant women expected to be discharged before delivery were randomized to app‐based or standard discharge. Randomization was based on discharge day in an alternating pattern (Sunday–Thursday), with the order reversed weekly.

The standard group received a printed discharge letter before leaving. The app group received one‐page with recommendations and contact details, and were discharged home. The full discharge letter was later accessible via the app. Patients were instructed to contact the department if the letter did not appear within 24 hours.

The primary outcome was time from discharge decision to actual physical departure. A phone interview was conducted within one week to assess satisfaction, experience, and understanding.

**Results**: Sixty women were included per group. Patient characteristics are shown in Table 1. Time from discharge decision to physical departure was significantly shorter in the app group (0.8±0.8 vs. 2.2±1.3 hours, *p* < 0.0001). Satisfaction scores were higher with app‐based discharge. The recommendations of the single‐page summary and full letter were consistent. All app‐group patients successfully accessed their discharge letter.

**Conclusion**: App‐based immediate discharge significantly reduced time to departure and improved patient satisfaction without compromising safety.

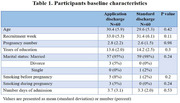

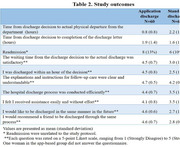

**3:30 PM – 5:00 PM**

## 1232 | **Sources of Information for Foley Balloon use During Induction of Labor Impacts Its use**

Robert E. Jones^1^; Elizabeth Bloom^2^; Kimberley Campbell^1^; Anna M. Modest^1^; Sarah E. Little^3^

^1^Beth Israel Deaconess Medical Center, Boston, MA; ^2^Beth Israel Deaconess Medical Center, BOSTON, MA; ^3^Beth Israel Deaconess Medical Center, Newton, MA

**Objective**: Patients presenting to the labor and delivery (L&D) unit often have strong opinions about using a foley balloon (FB) for induction of labor (IOL) due to concern for significant discomfort. This study examines what sources shape patient perceptions of FB use and how these perceptions influence FB utilization.

**Study Design**: Patients ≥ 18 years old presenting for IOL with intact membranes were surveyed at the start of induction prior to FB placement. The survey asked whether patients had heard of a FB before, where they heard about it, and whether the sources portrayed it positively or negatively.

**Results**: Over a 2‐month period, 366 patients underwent an IOL, 183 (50%) completed the survey. Of these, 95 (52.9%) underwent FB placement. Most (72.1%) had heard of a FB and the main information sources included obstetric providers (56.1%), social media/internet (43.2%), and family/friends (39.4%) (Figure 1). Of those reporting a perspective, positive views were most common among those citing obstetric providers (88.5%) and midwifes/doulas (100%), compared to social media/internet (8.7%) and family/friends (25%). FB use was similar regardless of prior awareness (51.5% vs. 52.9%). Patients citing obstetric providers had higher FB use compared to those citing social media (56.7% vs. 49.1%). This difference increased when comparing FB use in those citing obstetric providers without social media/internet vs. social media/internet without obstetric providers (55.8% vs. 38.5%).

**Conclusion**: Obstetric providers, social media/internet, and family/friend are the most common sources of FB information. Social media/internet is associated with negative perceptions and decreased FB use, while obstetric providers are linked to positive views and increased use.  These findings underscore the critical role obstetric providers play in promoting evidence‐based care, given the known benefits of a FB for IOL.

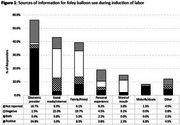

**3:30 PM – 5:00 PM**

## 1233 | **Birthweight Discordancy and Its Direction: Impact on Cesarean Risk During Attempted Vaginal Twin Delivery**

Roie Alter^1^; Inbal Akavian^1^; Shlomo Yahalomy^2^; Joshua Isaac Rosenbloom^3^; Aharon Tevet^2^; Hadar Rosen^1^

^1^Hadassah Ein‐karem hospital, Obstetrics and gynecology department, Jerusalem, Jerusalem, Yerushalayim; ^2^Department of Obstetrics and Gynecology, Hadassah Hebrew University Medical Center, Ein Kerem, Jerusalem, Yerushalayim; ^3^Hadassah Medical Center and Hebrew University of Jerusalem, Jerusalem, Israel, Jerusalem, Yerushalayim

**Objective**: To assess how birthweight discordancy influences the risk of urgent cesarean delivery during an attempted vaginal twin birth, and whether this risk varies based on discordancy direction, specifically, whether the larger twin is presenting (Twin A) or non‐presenting (Twin B).

**Study Design**: This retrospective cohort study included all diamniotic twin pregnancies at ≥32 weeks’ gestation with a cephalic‐presenting first twin who underwent a trial of labor at a tertiary medical center between 1/2019‐4/2025. Discordancy was defined as |Twin A–Twin B| ÷ larger twin birthweight >20%. Discordancy direction was categorized as “positive” (Twin B > Twin A) or “negative” (Twin A > Twin B). An SGA twin was defined as <10th percentile by the Fetal Medicine Foundation birth weight charts. The Pearson correlation coefficient was employed to measure the linear correlation between discordancy and the mode of delivery.

**Results**: Of 1017 women attempting vaginal twin delivery, 755(74.2%) achieved successful vaginal delivery of both twins, while 249 (24.5%) required cesarean delivery. Birthweight discordancy was associated with a significantly increased cesarean delivery rate compared to concordant twins (41.9% vs 23.7%, *p* < 0.001). When Twin A was larger, the cesarean delivery risk increased 87% (RR = 1.87, 95% CI: 1.34‐2.61, *p* < 0.001), whereas when Twin B was larger, risk was reduced by 39% though not statistically significant (RR = 0.61, 95% CI: 0.35‐1.07, *p* = 0.085). Among discordant pairs, cesarean delivery was three times more likely when the presenting twin was larger (RR = 3.07, 95% CI: 1.70‐5.53, *p* < 0.001). Nearly all discordant cases involved at least one SGA infant.

**Conclusion**: Birthweight discordancy increases the likelihood of urgent cesarean delivery in twin pregnancies undergoing a trial of labor. The increased risk may be influenced by the high rate of SGA fetuses in discordant pairs. This risk is notably higher when the presenting twin is the larger of the pair. Potential explanations include mechanical complications during labor or provider concern about the smaller second twin wellbeing during labor.

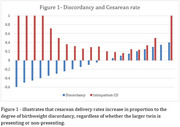

**3:30 PM – 5:00 PM**

## 1234 | **Trisomy 21 Fetal Growth: 18 Years of Sonographic Experience and Outcome Data**

Ronan Daly^1^; Jessie Amick^2^; Patrick Dicker^3^; Adam Roche^4^; Sean Daly^5^; Fergal D. Malone^6^; Sieglinde Mullers^7^

^1^Royal College of Surgeons Ireland ‐ Rotunda Hospital, Dublin, Dublin; ^2^GW SMHS, GW SMHS, DC; ^3^Department of Obstetrics and Gynaecology, Royal College of Surgeons in Ireland, Dublin, Ireland., Royal College of Surgeons in Ireland, Dublin; ^4^Trinity College Dublin, Dublin, Dublin; ^5^Rotunda Hospital, Dublin, Dublin; ^6^Department of Obstetrics and Gynaecology, Royal College of Surgeons in Ireland, Dublin, Ireland., Dublin, Dublin; ^7^Rotunda Hospital, Rotunda Hospital, Dublin

**Objective**: Fetal growth restriction (FGR) commonly features in Trisomy 21 (T21) but it is unclear if this reflects a separate pathology. As most T21 cases are terminated in the US, few data exist to guide clinicians on expected fetal growth. The different approach of our country offers opportunity for data on fetal growth in expectantly managed T21 cases. We reviewed serial fetal growth of ongoing T21 pregnancies using standard biometry (Hadlock‐3 formulae).

**Study Design**: Prenatally diagnosed T21 cases were identified from a prospectively collected registry with serial biometry extrapolated from Viewpoint 5® (abdominal circumference (AC), head circumference (HC), femur length (FL) and estimated fetal weight (EFW)). Postnatal diagnoses were included where third trimester biometry was recorded. Maternal demographics, pregnancy outcomes and placental histology were obtained. Resulting scatter plots for T21 pregnancies were generated.

**Results**: A total of 114 cases of T21 (105 prenatal and 9 postnatal) were identified over an 18‐year period (2006‐2023). Outcome data are presented in Table 1. On compilation of growth centiles, a substantial proportion of EFW trended below the 50th percentile for gestation, particularly from 28 weeks (Figure 1). AC remained consistent in trajectory throughout pregnancy but all other growth parameters fell below the 50th percentile (Table 1). Umbilical artery Doppler abnormalities were seen in 19% (22/114) of pregnancies. Only 34% of pre‐delivery EFWs and 31% of birthweights were below the 10th percentile for gestation. 30 placentas underwent histological examination with diverse abnormalities found in the majority, of which only one third demonstrated typical features of uteroplacental disease (Table 1).

**Conclusion**: T21 pregnancies demonstrate unique growth patterns in‐utero. Resulting centiles generated for T21 pregnancies in this study differ from those of euploid singleton norms, unexplained by uteroplacental disease alone. Customized prenatal centiles for T21 should be validated in future studies to inform the care of expectantly managed T21, particularly where FGR is co‐existing.

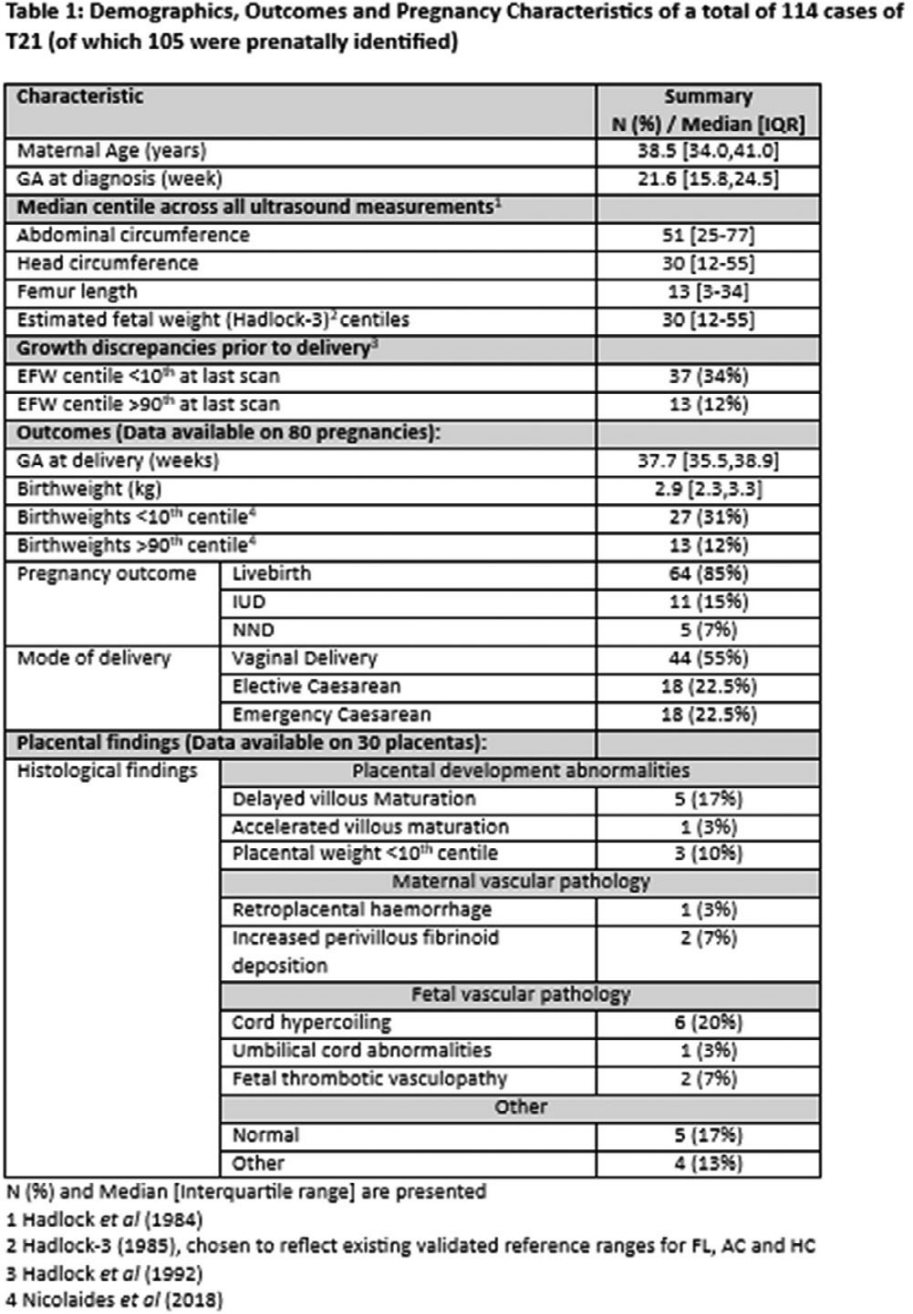

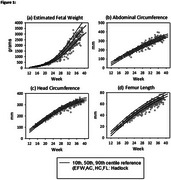

**3:30 PM – 5:00 PM**

## 1235 | **Missile Exposure and Obstetric Outcomes: A Retrospective Study on Preterm Birth and Low Birth Weight**

Roy Bitan^1^; Racheli Magnezi^2^; Roza Shperling‐Berkovitz^3^; Uri Amikam^4^

^1^Lis Hospital for Women's Health, Tel Aviv, Tel Aviv; ^2^Department of Management, Health Systems Management Program, Bar Ilan University, Ramat Gan, Israel, Ramat Gan, HaMerkaz; ^3^Lis Hospital for Women's Health, Tel Aviv Sourasky Medical Center, ichilov, HaMerkaz; ^4^Lis Hospital for Women's Health, Tel Aviv Sourasky Medical Center, Tel Aviv, Tel Aviv

**Objective**: To examine whether varying levels of missile exposure during armed conflict are associated with differences in obstetric outcomes, specifically preterm birth (PTB), and low birth weight (LBW) (<2,500 grams).

**Study Design**: A retrospective cohort study utilizing data from nine university‐affiliated hospitals in Israel. Women who gave birth between October 7, 2023, and April 7, 2024, were included. In October 2023, an armed conflict commenced in Israel. Based on the number of missile alerts in their area of residence during this period, parturients were classified into three exposure groups: low (0–5 missiles) (less than once a month), medium (6–30 missiles), and high (  >30 missiles) (more than once a week). The rates of PTB (<37 gestational weeks) and LBW (<2,500 grams) were compared between groups. Multivariate logistic regression adjusted for confounders.

**Results**: Overall, 10,392 women were included: 3212 in the low‐exposure group, 4525 in the medium‐exposure group, and 2655 in the high‐exposure group. Women in the low‐exposure group were significantly younger and had lower socioeconomic status compared to the other groups (*p* < 0.001); preexisting comorbidities were comparable. Regarding obstetric outcomes, no statistically significant differences were found between groups in the rates of PTB or LBW. However, in multivariate logistic regression analysis adjusting for maternal age, parity, socioeconomic status, and maternal comorbidities, the association between high missile exposure and PTB approached statistical significance (adjusted odds ratio 1.25, 95% Confidence Interval 0.98–1.60; P‐value = 0.077).

**Conclusion**: Despite the assumption that higher missile exposure increases stress, no significant differences in PTB or LBW were found across exposure levels. This may reflect the Israeli healthcare system's resilience and preparedness during wartime, maintaining prenatal, mental health, and obstetric care even in heavily affected areas. Further research is needed on long‐term developmental outcomes and personalized stress responses in pregnancy.

**3:30 PM – 5:00 PM**

## 1236 | **Initiation or Continuation of oral Labetalol Versus Extended‐Release Nifedipine in Expectant Management of Severe Preeclampsia**

Ryan D. Farias^1^; Christopher S. Ennen^1^; Adam J. Schettler^1^; Ella Frazier^2^

^1^University of Virginia School of Medicine, Charlottesville, VA; ^2^University of Virginia School of Medicine, University of Virginia School of Medicine, VA

**Objective**: To compare maternal and neonatal outcomes among patients with preeclampsia with severe features managed expectantly with oral labetalol or extended‐release nifedipine.

**Study Design**: This is a retrospective cohort study of patients admitted for expectant management of preeclampsia with severe features <34 weeks and initiated or continued on oral labetalol or extended‐release nifedipine. Exclusion criteria included patients already on both medications at time of admission, and any contraindication to expectant management within 24 hours. The primary outcome was pregnancy latency. Secondary outcomes included need for early delivery, initial agent dose increase, addition of second agent, number of acute treatment episodes of severe hypertension, indications for early delivery, birth weight, and one‐ and five‐minute APGARs.

**Results**: Baseline characteristics are shown in Table 1. Overall, comparing labetalol vs nifedipine, there was no difference in median latency period [4 (5) vs 5 (6.25)] days *p* = 0.28), need for early delivery (87% vs 79% *p* =  .09), initial agent dose increase (73% vs 73% *p* = 0.79), addition of second agent (39% vs 30% *p* = 0.13), and median number of acute treatment episodes [3 (3) vs 3 (3.5) *p* = 0.83]. A higher proportion of patients in the nifedipine group required early delivery for elevated liver enzymes (26% vs 7% *p* =  .001) and/or for elevated creatinine (15% vs 5% *p* =  .026). There was also a trend towards a higher proportion of patients in the labetalol group requiring early delivery for pulmonary edema (10% vs 3% *p* =  .078). Results are summarized in Table 2.

**Conclusion**: Initiation or continuation of oral labetalol vs nifedipine did not significantly impact maternal or neonatal outcomes. However, patients initially on nifedipine were more likely to experience elevated liver enzymes and/or elevated creatinine as an indication for delivery. There was a trend towards the development of pulmonary edema with labetalol. These findings suggest potential differences in disease trajectory by antihypertensive choice and warrant further prospective investigation.

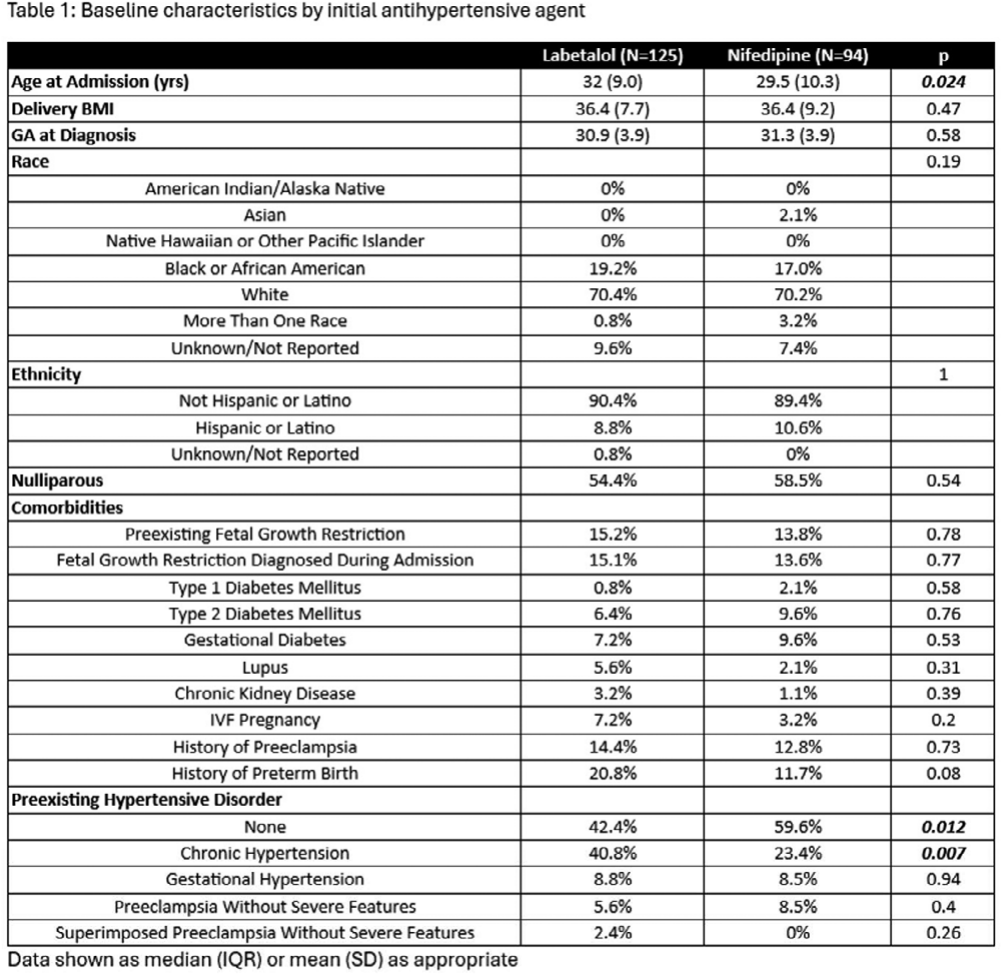

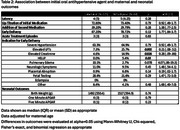

**3:30 PM – 5:00 PM**

## 1237 | **Psychosocial Stressors and Prenatal Inflammation in the Chicago Perinatal Origins of Disease Cohort**

Samantha P. Gottlieb^1^; Sarah L. Kaplan^2^; Frances Kincaid^3^; Estefania Espinosa^4^; Kennedi Williams^4^; Jiafeng Li^5^; Madeline R. Meirhaeghe^6^; Abigail L. Aron^7^; Mary Akel^5^; Tonia Branche^5^; Leena B. Mithal^8^; Stephanie A. Fisher^9^

^1^Ann & Robert H. Lurie Children's Hospital of Chicago, Lurie Children's Hospital, Chicago, IL, IL; ^2^Ann & Robert H. Lurie Children's Hospital of Chicago, Atlanta, GA; ^3^Northwestern University, Northwestern University, IL; ^4^Northwestern University, Chicago, IL; ^5^Ann & Robert H. Lurie Children's Hospital of Chicago, Chicago, IL; ^6^Ann & Robert H. Lurie Children's Hospital of Chicago, LIncolnshire, IL; ^7^Ann & Robert H. Lurie Children's Hospital of Chicago, Ann & Robert H. Lurie Children's Hospital of Chicago, IL; ^8^Ann & Robert H. Lurie Children's Hospital; Northwestern University Feinberg School of Medicine, Chicago, IL; ^9^Northwestern University Feinberg School of Medicine, Chicago, IL

**Objective**: To evaluate the association of antenatal psychosocial stressors with inflammatory biomarkers in pregnancy.

**Study Design**: Secondary analysis of data from the prospective Chicago Perinatal Origins of Disease (CPOD) cohort of pregnant people with singleton gestations at a large academic center. Exposures were participant scores on the Patient Health Questionnaire‐9 (PHQ‐9) and Generalized Anxiety Disorder‐7 (GAD‐7) at 8‐24 weeks (Table 1), and the Perceived Stress Scale (PSS‐10) and the Everyday Discrimination Scale (EDS) at 18‐36 weeks (Table 2), completed via electronic survey. Outcomes were log‐transformed concentrations of plasma inflammatory cytokines (IL1β, IL2, IL6, IL8, IL10, IFNγ, TNFα) and cytokine‐induced cell adhesion molecules (ICAM1, VCAM1), measured via Meso Scale Discovery immunoassay. Student's t‐tests compared mean differences in biomarker concentrations, and linear regression models generated beta (ß)‐coefficients adjusted for pre‐pregnancy body mass index.

**Results**: Of 118 enrolled participants, 99 completed the above psychosocial stress screeners and provided a plasma sample, with 22% and 26% identifying as Black or Hispanic, respectively. Median gestational age at plasma sample collection was 28.7 weeks’ gestation (IQR 26.1, 32.4). PHQ‐9 (mean difference 0.73 pg/mL; p‐value 0.02) and GAD‐7 scores ≥5 (mean difference 0.64 pg/mL; p‐value 0.03), indicating at least mild depressive and/or anxiety symptoms, were associated with higher IL6 concentrations, and a PHQ‐9 score ≥5 (vs. <5) was associated with higher ICAM1 concentrations. When assessed as continuous measures, higher PSS scores were associated with lower IL1ß (adj. ß ‐0.57; 95% CI ‐0.03, ‐1.12) and IL10 (adj. ß ‐0.52; 95% CI ‐0.05, ‐0.98). Higher EDS scores were associated with lower IFNγ (adj. ß ‐0.031; 95% CI ‐0.01, ‐0.05).

**Conclusion**: In the CPOD cohort, anxiety and depressive symptoms were positively associated with the pro‐inflammatory IL6, while greater perceived stress and discrimination in pregnancy were associated with suppression of anti‐inflammatory IL10 and IFNγ, a regulator of anti‐inflammatory processes.

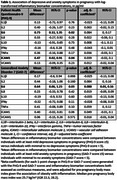

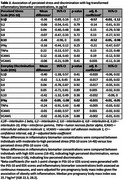

**3:30 PM – 5:00 PM**

## 1238 | **Trends in Twin Mode of Delivery Over Time**

Samantha Ocampo^1^; Alyssa R. Hersh^1^; Bharti Garg^2^; Aaron B. Caughey^2^

^1^Oregon Health & Science University, Portland, OR; ^2^Oregon Health & Science University, Oregon Health & Science University, OR

**Objective**: About 3% of all births in the United States are twin gestations. This number has been gradually rising largely due to an increase in the use of assisted reproductive technology. In 2008, approximately 75% of twins were delivered by cesarean birth. The objective of this study was to identify trends in the mode of delivery of twin gestations in a large US‐based cohort from 2008 to 2020.

**Study Design**: We performed a retrospective cohort study of twin pregnancies that delivered between 36 and 39 weeks in California between 2008 and 2020. Pregnancies were then stratified by parity, history of cesarean birth and presentation of the second twin. We graphically examined trends in mode of delivery and logistic regression analyses was used to determine odds of cesarean with increase in birth year.

**Results**: 56,562 twin pregnancies were identified. The cesarean birth rate across all time periods was 83.7% for all twins, 73.1% for vertex‐vertex presentation and 88.7% for vertex‐breech presentation. With increase in birth year, odds of cesarean decreased in nulliparous (OR = 0.98; 95% CI: 0.97‐0.99) and multiparous without prior cesarean (OR = 0.97; 95% CI: 0.96‐0.98) (Figure 1). When stratified by presentation of the second twin this trend was significant, except for nulliparous pregnancies with vertex‐breech presentation (Figure 2).

**Conclusion**: The twin cesarean birth rate in California is decreasing over time. This trend was strongest in vertex‐vertex presenting twins, but also demonstrated in vertex‐breech presentation. The most notable decrease was in multiparous patients with no prior cesarean and vertex‐vertex presentation which went from 59.1% in 2008 to 42.2% in 2020. Ongoing efforts to decrease cesarean birth rates are possible contributors to this finding and emphasize the importance of prenatal counseling on mode of delivery for twin gestations.

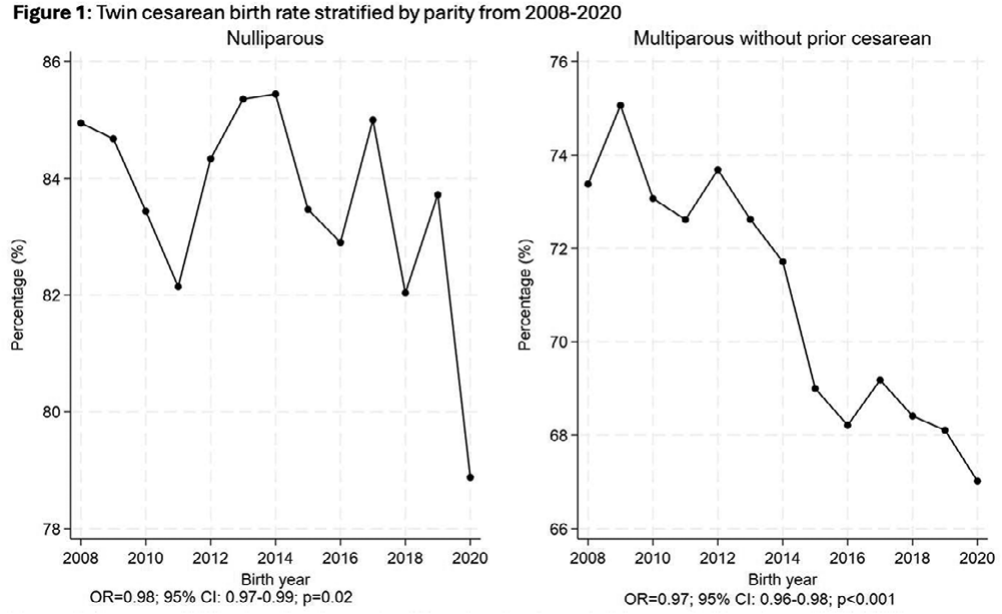

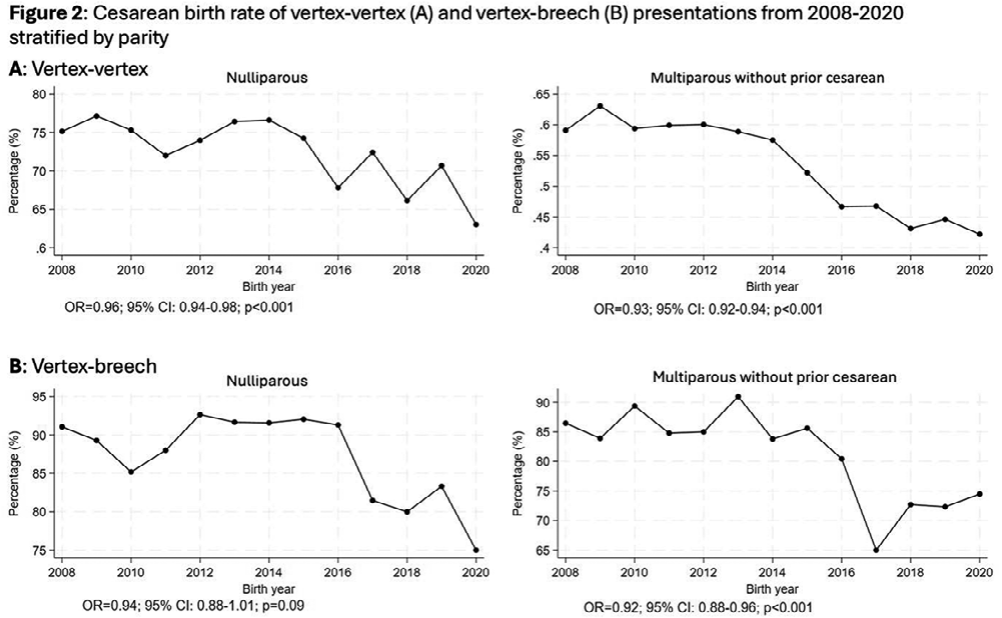

**3:30 PM – 5:00 PM**

## 1239 | **Long‐Term Cardiovascular Outcomes in Term‐Born Children Exposed to Antenatal Corticosteroids: A Population‐Based Cohort Study**

Sapir Ellouk^1^; Tamar Wainstock^2^; Eyal Sheiner^3^

^1^Department of Obstetrics and Gynecology, Soroka University Medical Center, Ben‐Gurion University, HaDarom; ^2^Department of Epidemiology, Biostatistics and Community Health Sciences, Faculty of Health Sciences, Ben‐Gurion University of the Negev, Beer Sheva, HaDarom; ^3^Soroka University Medical Center, Faculty of Health Sciences, Ben‐Gurion University of the Negev, beer sheva, HaDarom

**Objective**: Antenatal corticosteroids (ACS) are well established in reducing morbidity and mortality among preterm infants. However, the impact of ACS exposure on children born at term remains less clear. This study aimed to evaluate long‐term cardiovascular outcomes in term‐born offspring exposed to ACS.

**Study Design**: We conducted a population‐based retrospective cohort study of singleton term deliveries between 1998 and 2021. Long‐term cardiovascular morbidity was compared between term‐born offspring exposed to ACS before 34 weeks of gestation and unexposed offspring, using linked community and hospitalization databases. The cumulative incidence of cardiovascular morbidity was assessed with Kaplan–Meier survival curves, and the Cox proportional hazards model was used to adjust for potential confounders

**Results**: Among 182,626 term deliveries, 2093 offspring (1.1%) were exposed to ACS before 34 weeks of gestation. The overall incidence of cardiovascular morbidity per 1000 person‐years was significantly higher in the exposed group (Table), with a greater cumulative incidence over time (log‐rank 4.42; *p* = 0.036) (Figure). However, after adjustment for maternal age, gestational age, ethnicity, diabetes mellitus, hypertensive disorders, and mode of delivery, ACS exposure was not significantly associated with cardiovascular morbidity (adjusted hazard ratio [aHR], 1.15; 95% CI, 0.98–1.35; *p* = 0.068).

**Conclusion**: Exposure to antenatal corticosteroids before 34 weeks in pregnancies that reached term was not associated with increased long‐term cardiovascular morbidity in offspring after adjustment for confounders. These findings support the cardiovascular safety of ACS use in pregnancies that ultimately result in term delivery.

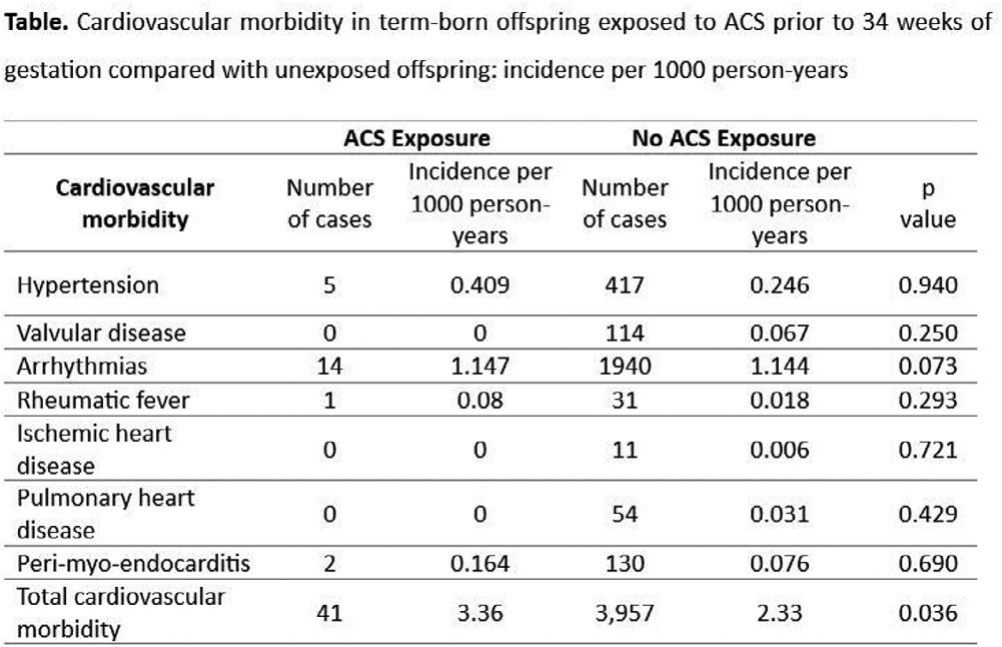

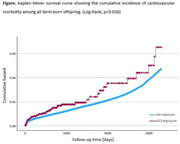

**3:30 PM – 5:00 PM**

## 1240 | **Maternal Body Mass Index and Neonatal Respiratory Outcomes Following Antenatal Corticosteroid Administration**

Sara E. Edwards^1^; Nicola F. Tavella^2^; Grace M. DiGiovanni^3^; Sunidhi Singh^3^; Camila Johanek^3^; Sarriyah Hanif^3^; Gabriele Baptiste^4^; Dayana Mirzaliev^1^; Monica J. Patel^1^; Alexandra N. Mills^3^; Daniel Katz^3^; Chelsea A. DeBolt^1^; Angela T. Bianco^3^

^1^Division of Maternal‐Fetal Medicine, Icahn School of Medicine at Mount Sinai, New York, NY; ^2^Division of Maternal‐Fetal Medicine, Icahn School of Medicine, Icahn School of Medicine at Mount Sinai Hospital, New York, NY; ^3^Icahn School of Medicine at Mount Sinai, New York, NY; ^4^SUNY Downstate Health Sciences University, Brooklyn, NY

**Objective**: Prior studies demonstrate improved preterm neonatal outcomes with administration of antenatal corticosteroids (ACS). However, no studies have investigated the interaction of this fixed dose medication with maternal weight or body mass index. Our study assessed the association of maternal weight and body mass index with neonatal outcomes after ACS administration. We hypothesized that steroids would have reduced efficacy at improving neonatal respiratory outcomes in cases of maternal obesity.

**Study Design**: This was a retrospective cohort study of all patients delivering at a single academic institution in New York City from January 1, 2013 ‐ December 31, 2023. Patients were included if they received two doses of betamethasone prior to delivery and had available maternal weight and height data. Patients were excluded if they received > 2 doses, did not deliver at our institution, received ACS at > 37 weeks, or experienced a stillbirth. We assessed electronic medical records for clinical and demographic data. Our primary outcome was a neonatal respiratory composite (transient tachypnea of the newborn, respiratory distress syndrome, use of CPAP for > 12 hours, oxygen for > 24 hours, intubation, or surfactant). Multivariable logistic regression models controlling for potential confounders examined association of maternal body mass index and weight with neonatal outcomes.

**Results**: 2638 patients met inclusion criteria. Of these, 850 had obesity (Table 1). Patients with obesity were more likely to have diabetes, hypertension, chorioamnionitis, large for gestational age fetuses, and deliver at earlier gestational ages (Table 1). After controlling for potential confounders, neonates of patients with obesity had significantly decreased rates of intubation. We found no other differences in neonatal outcomes by maternal weight or BMI (Table 2).

**Conclusion**: Although obese patients receiving ACS were less likely to experience neonatal intubation, other neonatal respiratory outcomes following ACS administration did not differ significantly by maternal weight or BMI.

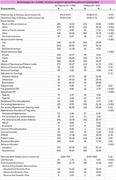

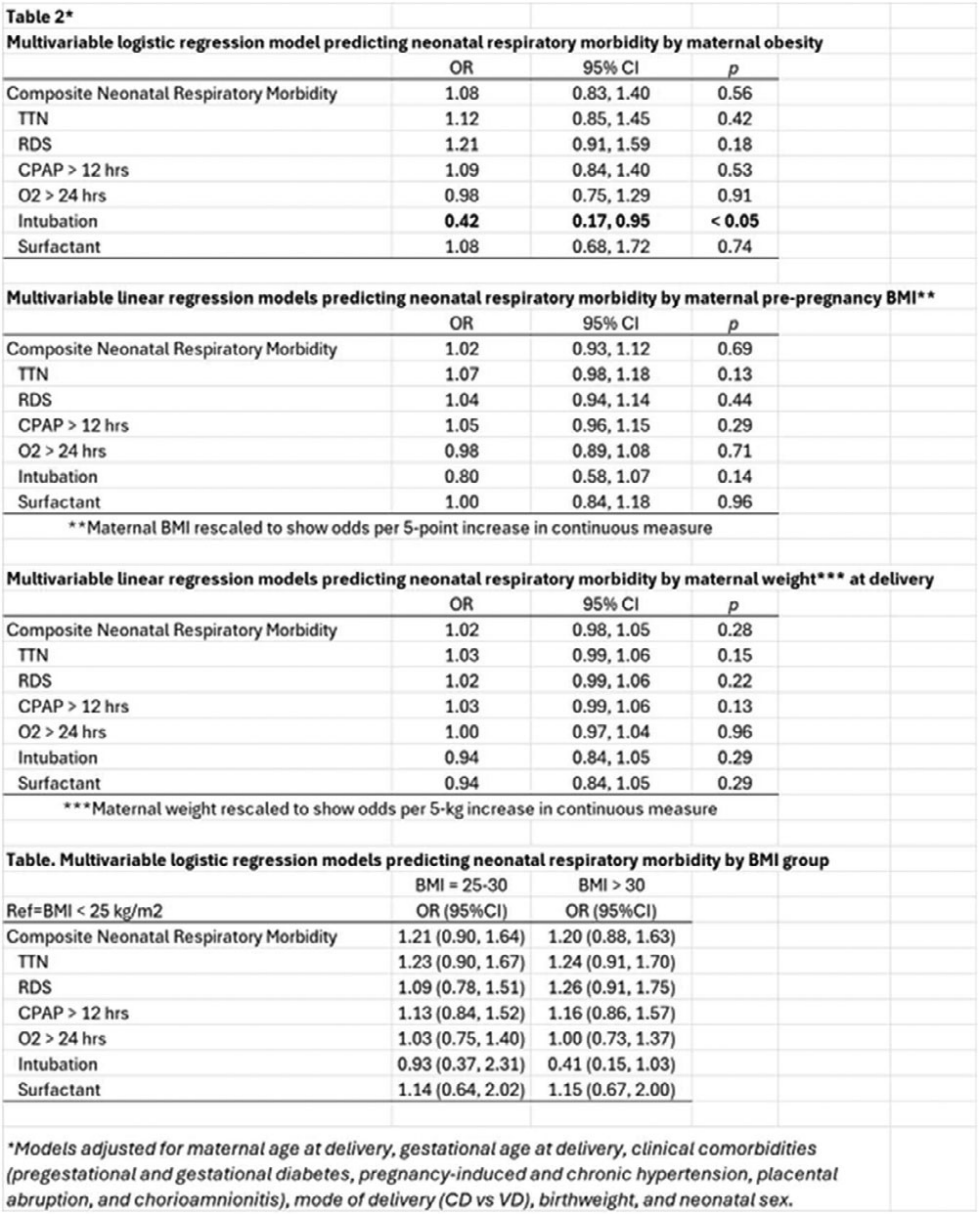

**3:30 PM – 5:00 PM**

## 1241 | **Characteristics and Preventability of Severe Maternal Morbidity Before and After Implementation of SMM‐Targeting Interventions**

Sara Giuliano^1^; Katherine H. Campbell^2^; Lisbet S. Lundsberg^1^; Jennifer F. Culhane^1^; Heather S. Lipkind^3^; Colleen Sinnott^2^; Olga Grechukhina^2^

^1^Department of Obstetrics, Gynecology, and Reproductive Sciences, Yale School of Medicine, New Haven, CT; ^2^Division of Maternal Fetal Medicine, Yale School of Medicine, New Haven, CT; ^3^New York‐Presbyterian/Weill Cornell Medicine, New York, NY

**Objective**: Severe maternal morbidity (SMM) is rising in the US. Institutional review of SMM cases has the potential to improve obstetric care, yet there is limited evidence supporting its efficacy. As a quality improvement project here we assess the impact of implementation of new policies and care pathways that stem from the SMM review process on SMM characteristics and preventability.

**Study Design**: This is a retrospective cohort study of SMM cases (defined by the ACOG/SMFM criteria) managed at a single tertiary referral center from 9/1/2015, when the SMM review started, to 4/1/2024. SMM cases were compared across two time periods: before and after June 1, 2019, marking the start of SMM review‐based interventions. Patient data and event details were manually extracted from EMR and reviewed by a multidisciplinary team. Consensus on ability to alter outcome (“strong” with definitive opportunities to alter outcome, “possible” with some opportunity to alter outcome and “none” with no opportunity to alter outcome) was reached.  The presence of various patient, provider and system‐level factors was also assessed. Bivariate analysis was performed to compare the two groups using chi‐square or t‐tests.

**Results**: A total of 304 SMM cases were reviewed: 130 cases before and 175 after 6/1/2019. Patient characteristics and etiologies of SMM were comparable across both groups (Table 1). In the post‐intervention group, there were fewer cases with strong (13.2 vs 17.7%) or possible (37.4% vs 46.9%) opportunity to alter outcome (*p* = 0.049). Additionally, provider factors, issues with management hierarchy, staff education, policies and procedures, team communication and equipment were significantly less commonly identified as contributors to SMM in the post‐intervention group (Table 2).

**Conclusion**: Implementation of SMM‐targeting interventions based on a rigorous institutional SMM review process was significantly associated with less frequent identification of provider and system factors as contributors and an overall significant reduction in preventable and potentially preventable SMM cases.

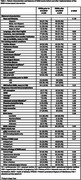

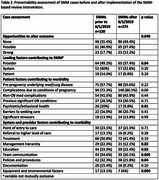

**3:30 PM – 5:00 PM**

## 1242 | **Cost‐Effectiveness of a Multivessel Embolization Strategy for Management of Placenta Accreta Spectrum: A Decision‐Analytic Model**

Sara I. Jones^1^; Aya Bashi^2^; Evan Myers^3^; Lillian Boettcher^4^; Anthony E. Melendez Torres^5^; Jennifer B. Gilner^1^; Amanda M. Craig^1^

^1^Duke University School of Medicine, Durham, NC; ^2^Duke University Hospital, Durham, NC; ^3^Duke University, Durham, NC; ^4^Department of Obstetrics and Gynecology, Duke University School of Medicine, Duke University School of Medicine/Durham, NC; ^5^Division of Maternal Fetal Medicine, Department of Obstetrics and Gynecology, Duke University, Durham, NC

**Objective**: To estimate the cost‐effectiveness of our institution's surgical strategy using multivessel embolization (MVE) followed by immediate hysterectomy for managing placenta accreta spectrum (PAS) compared to non‐MVE approaches.

**Study Design**: We developed a decision‐analytic model in TreeAge Pro 2025 to compare a standardized MVE‐based strategy with immediate hysterectomy for PAS to alternative immediate hysterectomy approaches using a healthcare system perspective. Primary outcome was cost‐effectiveness of MVE‐based strategy. Primary exposure was prenatal suspicion of high‐grade PAS with planned immediate hysterectomy. Costs included operative time, surgical approach, length of stay, complications, and reoperation/readmission. Estimates were obtained from institutional data and the literature, and all parameters characterized as distributions. The model was run probabilistically using a 10,000‐trial microsimulation (sampling from the age distribution of PAS), and 5000 samples from each of the input parameters. We used willingness‐to‐pay threshold of $100,000 per life‐year gained. Additional deterministic sensitivity analyses included the relative risk (RR) of prolonged hospital stay (1.0–2.0) with alternative approaches and costs associated with use of resuscitative endovascular balloon occlusion of the aorta (REBOA) ($0–$25,015, with the low vs high range corresponding to probabilities from no vs all patients undergoing REBOA).

**Results**: In the base case, MVE had lower mean total costs than alternatives ($32,704 [95% CI: $32,544–$32,864] vs $48,089 [95% CI: $47,979–$48,198]), with similar discounted life expectancy (25.49 years). Higher OR costs ($19,191 vs $11,513) were offset by fewer >5‐day hospitalizations (11.9% vs 23.9%). MVE was cost‐saving in 97.9% of simulations, and cost‐effective in 88.3% of simulations.

**Conclusion**: Higher OR costs associated with an MVE‐based strategy for high grade PAS may be more than offset by shorter associated hospital length of stay. Future analyses adjusting for potential patient heterogeneity and using larger sample sizes are needed.

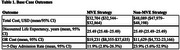

**3:30 PM – 5:00 PM**

## 1243 | **Forceps Use in Flux: Trends in Assisted Vaginal Delivery Across the United States**

Sarah H. Abelman; Frank I. Jackson; Oladunni Ogundipe; Luis A. Bracero; Matthew J. Blitz

Northwell, New Hyde Park, NY

**Objective**: To evaluate national and state‐level trends in forceps‐assisted vaginal delivery (FAVD) in the United States over the past decade.

**Study Design**: This was a retrospective cohort study using 2014 and 2023 National Vital Statistics data from 48 states to assess FAVD rates in nulliparous term singleton vertex (NTSV) births. Connecticut and Rhode Island were excluded due to missing operative delivery data. For each year, the proportion of total deliveries that were operative vaginal deliveries (OVDs) was calculated, as was the proportion of OVDs performed via forceps. States were ranked by FAVD rate for each year. Change in rank order from 2014 to 2023 was assessed using Kendall's Tau for ordinal association, with *p* < 0.05 considered significant. Analyses were performed using R version 4.4.1.

**Results**: There were 1,225,588 NTSV births in 2014, of which 13,161 (1.1%) were FAVD (17.7% of 74,428 total OVDs). There were 1,177,636 NTSV births in 2023, of which 9388 (0.8%) were FAVD (15.6% of 60,268 OVDs). Overall, there was a 27.3% decrease in the rate of FAVD, and an 11.9% decline in the proportion of OVDs that were done via forceps. Utah (3.3%), Wyoming (3.3%), and New Jersey (3.2%) were the states with the highest rates of FAVD in 2014, and rates declined in almost all states by 2023 (Figure 1). However, 4 states had increased rates of FAVD (California, Massachusetts, Maine, and New Hampshire). There was a statistically significant shift in rankings (Kendall Tau = 0.440, *p* < 0.001) for the 48 included states (Figure 2).

**Conclusion**: While FAVD rates have declined nationally among NTSV births over the past decade, state‐level trends varied, with a significant change in rank order. These findings suggest evolving regional practice patterns and may inform future efforts to standardize training and access to operative vaginal delivery techniques.

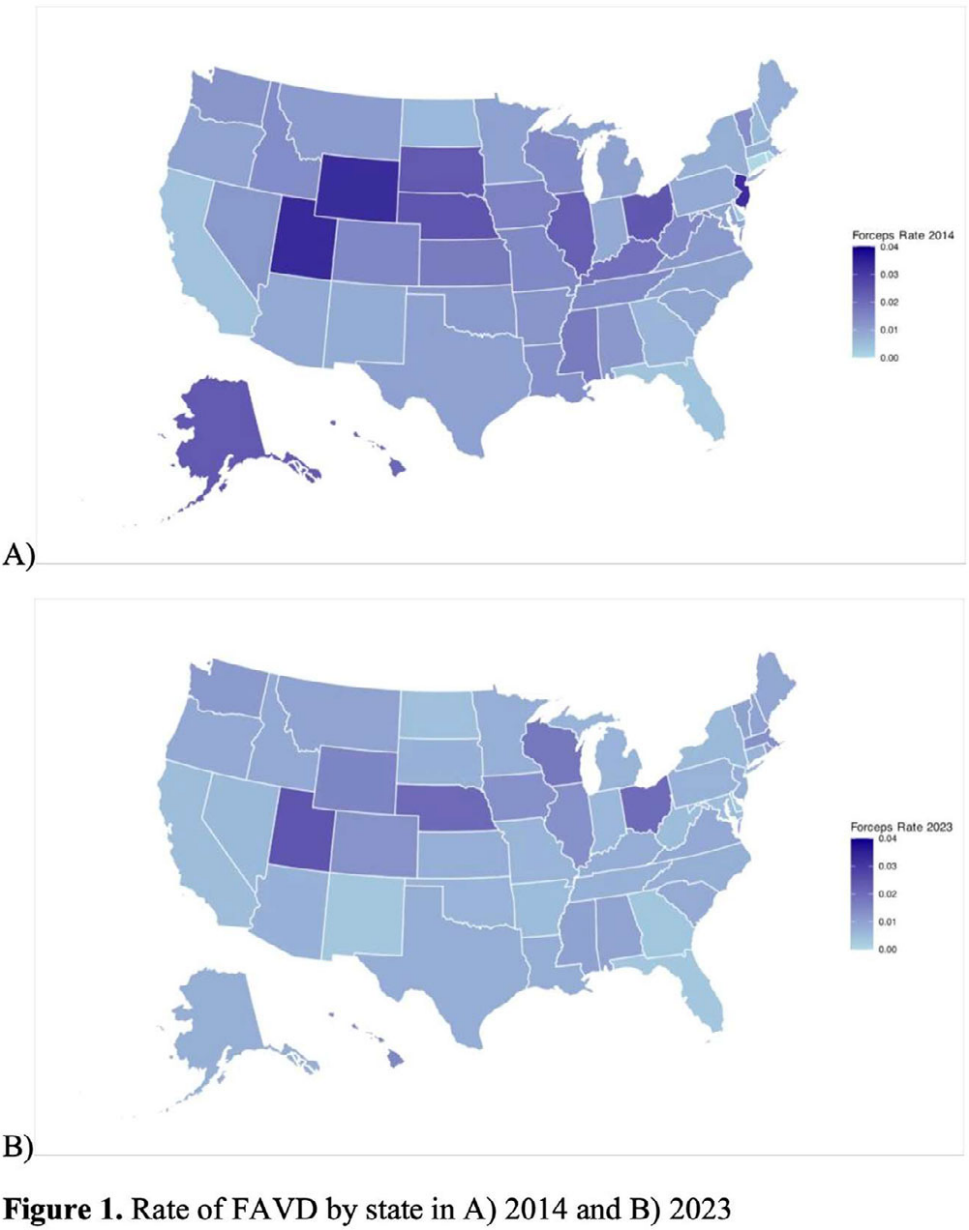

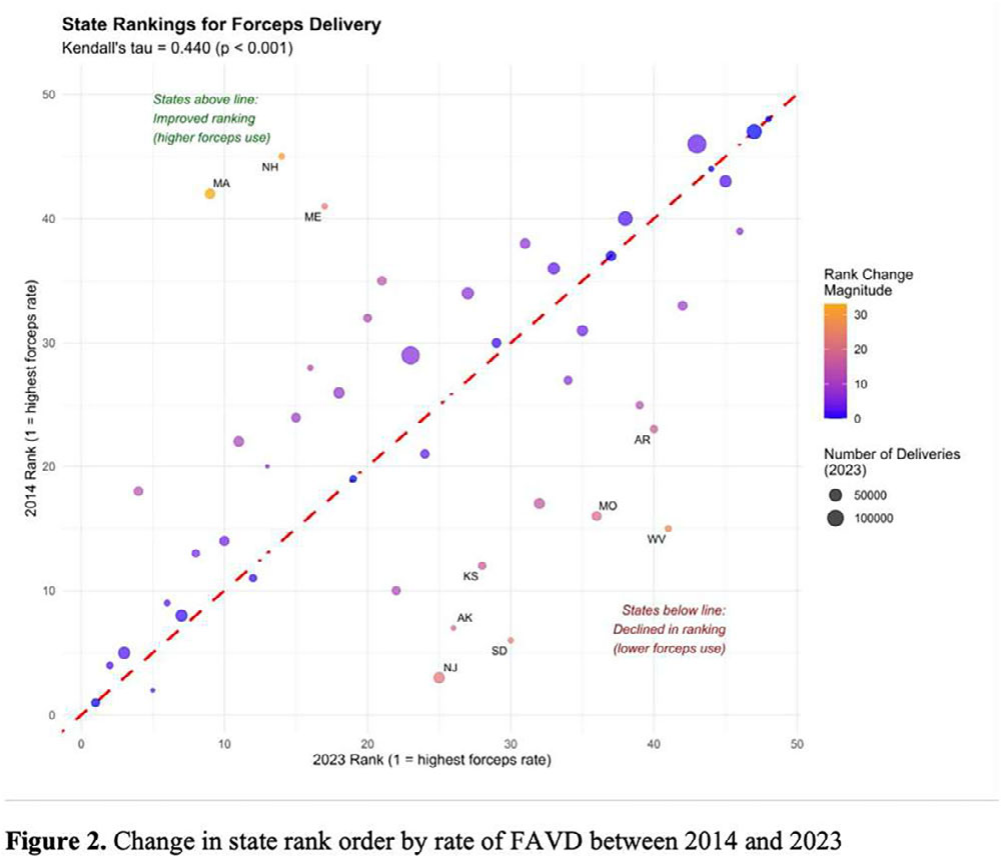

**3:30 PM – 5:00 PM**

## 1244 | **Early‐Onset Severe Superimposed Preeclampsia: Diagnostic Criteria and Severe Maternal and Neonatal Morbidity**

Sarah H. Abelman^1^; Yossi Bart^2^; Frank I. Jackson^1^; Michael T. Xie^3^; Zakaria Doughan^4^; Joe Haydamous^5^; Ahmed Zaki Moustafa^6^; Baha M. Sibai^2^; Matthew J. Blitz^1^

^1^Northwell, New Hyde Park, NY; ^2^Division of Maternal‐Fetal Medicine, Department of Obstetrics, Gynecology and Reproductive Sciences, McGovern Medical School at UTHealth Houston, Houston, TX; ^3^Donald and Barbara Zucker School of Medicine at Hofstra/Northwell, Bay Shore, NY; ^4^Department of Obstetrics and Gynecology, McGovern Medical School at UT Health, Houston, Houston, TX; ^5^Department of Obstetrics, Gynecology and Reproductive Sciences, McGovern Medical School at UTHealth Houston, Department of Obstetrics and Gynecology, McGovern Medical School at UT Health, Houston, TX; ^6^Division of Maternal‐Fetal Medicine, Department of Obstetrics, Gynecology and Reproductive Sciences, McGovern Medical School at UTHealth Houston, University of Texas ‐ Houston, TX

**Objective**: Chronic hypertension with superimposed preeclampsia is a diagnostic challenge. This study assessed whether the criteria used for diagnosis are associated with adverse maternal and neonatal outcomes.

**Study Design**: This retrospective cohort study included pregnancies complicated by superimposed preeclampsia with severe features diagnosed between 23 0/7 and 33 6/7 weeks’ gestation from 2019‐2024 across two health systems. Multiple gestations and fetal demises were excluded. The primary exposure was diagnostic criteria used, categorized as: 1) Severe‐range blood pressure (BP) only, 2) Severe‐range BP and severe lab or clinical criteria as defined by ACOG, and 3) Mild‐range BP plus severe lab or clinical criteria. Maternal outcomes included composite severe maternal morbidity (SMM) and non‐transfusion SMM. Neonatal outcomes included composite severe neonatal morbidity (SNM), small for gestational age (SGA), neonatal intensive care unit (NICU) stay >1 month, hypoglycemia, and 5‐minute Apgar < score 7. Multivariable logistic regression was used, adjusting for age ≥35, body mass index class, nulliparity, diabetes, gestational age at delivery, and race/ethnicity. Fetal growth restriction was adjusted for in the models for neonatal outcomes (excluding SGA). Analyses were performed in R 4.3.1.

**Results**: Among 431 pregnancies, diagnosis was based on only severe‐range BP in 80.3%, severe‐range BP plus lab/clinical criteria in 14.2%, and mild‐range BP plus lab/clinical criteria in 5.6%. Composite SMM did not differ by group, but non‐transfusion SMM was higher in those with severe‐range BP plus lab/clinical findings (*p* = 0.013). In 426 pregnancies with available neonatal outcomes, 53 (12.4%) delivered before 28 weeks. Neonates born to those with severe‐range BP plus lab/clinical criteria had higher odds of hypoglycemia (*p* = 0.01). No differences were observed in SNM, SGA, NICU length of stay, or 5‐minute Apgar.

**Conclusion**: In early‐onset superimposed preeclampsia with severe features, diagnosis with severe range BP plus lab/clinical criteria was associated with increased non‐transfusion SMM and neonatal hypoglycemia.

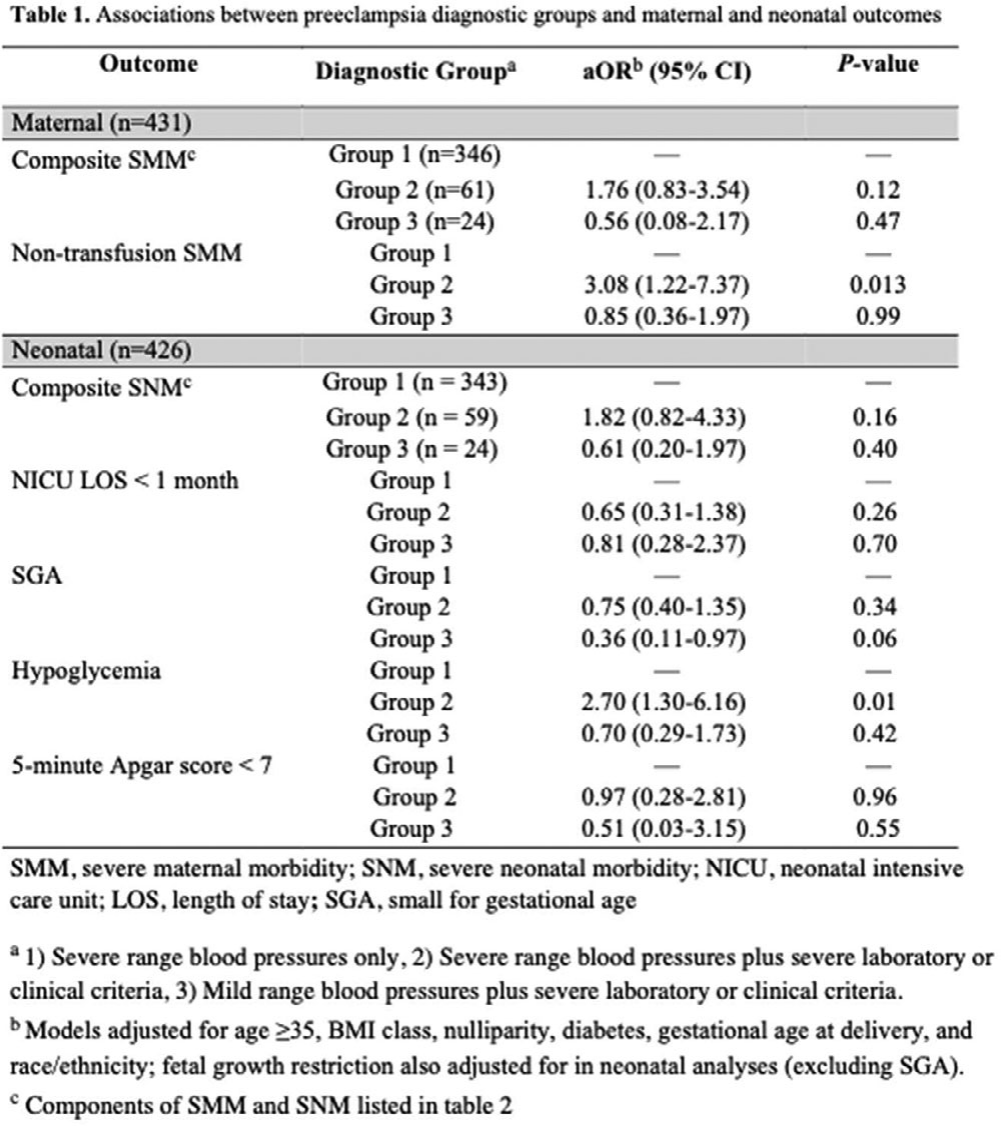

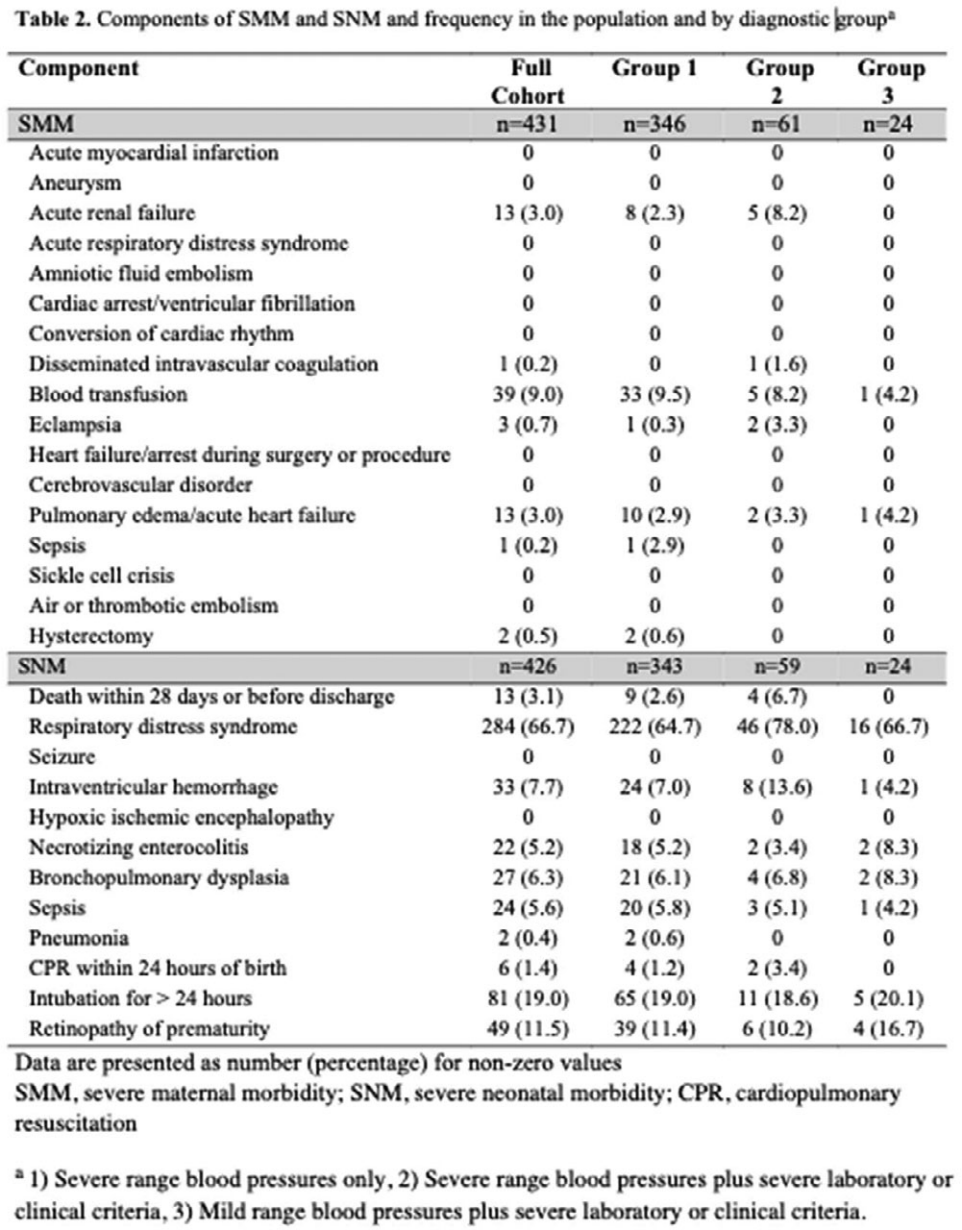

**3:30 PM – 5:00 PM**

## 1245 | **Changes in Cardiac Morphometry in Fetal Lower Urinary Tract Obstruction**

Sarah Araji^1^; Betul Yilmaz Furtun^1^; Sara Al‐Haddad^1^; Michael D. Jochum, Jr.^1^; Roopali V. Donepudi^2^; Magdalena Sanz Cortes^2^; Jessian L. Munoz^3^; Cara Buskmiller^4^; Brian A. Burnett^1^; Michael A. Belfort^1^; Ahmed A. Nassr^2^

^1^Baylor College of Medicine, Houston, TX; ^2^Baylor College of Medicine, Baylor College of Medicine, TX; ^3^Texas Children's Hospital, Texas Children's Hospital, TX; ^4^Baylor College of Medicine, Austin, TX

**Objective**: Fetal lower urinary tract obstruction (LUTO) can lead to oligohydramnios, pulmonary hypoplasia, and eventual renal failure. It is also frequently associated with abnormal cardiac findings on prenatal imaging, including cardiomegaly, ventricular hypertrophy, and pericardial effusion. Some studies have additionally reported small left‐sided cardiac structures. This study aimed to characterize detailed fetal echocardiographic features in LUTO and compare them with gestational age (GA)‐matched controls, adjusting for estimated fetal weight (EFW), with an emphasis on quantitative cardiac dimensions.

**Study Design**: This retrospective cohort study included fetuses diagnosed with isolated LUTO who underwent at least one fetal echocardiogram at our institution from 2013 to 2024. Fetuses with associated chromosomal abnormalities or additional major anomalies were excluded. Controls were 1:1 GA‐matched fetuses with normal cardiac anatomy. Quantitative cardiac measurements were compared between groups and adjusted for EFW. Logistic regression analysis was used to assess cardiac variables as predictors of LUTO, reported as adjusted odds ratios (ORs) with 95% confidence intervals (Figure 1).

**Results**: Among 121 LUTO fetuses, qualitative findings included pericardial effusion (27%), right ventricular hypertrophy (16.5%) and left ventricular hypertrophy (14.8%). Compared with controls, LUTO fetuses had significantly higher cardiothoracic ratio and smaller cardiac structures, including the mitral and tricuspid valves, aortic and pulmonary valves, and branch pulmonary arteries (all *p* < 0.01). These differences remained significant after EFW adjustment.

**Conclusion**: In addition to previously reported findings, LUTO fetuses demonstrate global cardiac morphometric changes, affecting both the left and right‐sided structures. These may reflect impaired diastolic filling or decreased venous return. Our findings suggest a potential role for serial fetal echocardiography—especially post‐intervention—to monitor evolving cardiac physiology and guide prenatal counseling.

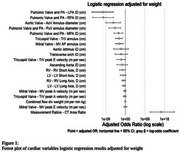

**3:30 PM – 5:00 PM**

## 1246 | **Do First Stage Uterine Contractility Measures Predict Second Stage Duration?**

Sarah Y. Cohen^1^; Candice L. Woolfolk^2^; Jeannie C. Kelly^2^; Brianna Bradley^1^; Lori Atwood^2^; Nicole McCandless^1^; Cookie Van^1^; Paige Wilder^1^; Antonina I. Frolova^3^; Nandini Raghuraman^3^

^1^Washington University School of Medicine, Saint Louis, MO; ^2^Washington University School of Medicine, Washington University School of Medicine, MO; ^3^Washington University School of Medicine, St. Louis, MO

**Objective**: National guidelines base first stage arrest on contraction adequacy, defined as ≥200 Montevideo units (MVUs). However, little is known about whether contraction adequacy or other contraction features predict second stage outcomes. We assessed the relationship between first stage uterine contractility measures and second stage duration.

**Study Design**: This was a secondary analysis of a prospective cohort of singleton pregnancies at ≥37 weeks’ gestation with intrauterine pressure catheters (IUPC) placed at time of amniotomy. This analysis only included vaginal deliveries. Contraction data, including peak pressure, MVUs, frequency, duration, tachysystole, and resting tone were collected by research nurses in 10‐minute epochs for the 1 hour prior to complete dilation. Linear and logistic regression models were used to test the association between these contraction parameters and second stage duration as well as second stage ≥1.5 hours.

**Results**: Of 190 patients with IUPC data and vaginal deliveries, 92 (48%) were nulliparous and 31 (16%) had a second stage ≥1.5 hours. After adjusting for parity and infant weight, higher peak pressure and greater MVUs were significantly associated with shorter second stage. Each 1 mmHg increase in peak pressure was associated with a 1.8‐minute decrease in second stage (β = –0.03 hr, 95% CI–0.06 to–0.01, *p * = 0.01), and each 1 MVU increase was associated with a 0.6‐minute reduction (β = –0.01 hr, 95% CI–0.01 to–0.001, *p * = 0.03) (Table). Peak pressure and having at least one 10‐minute segment of adequate MVUs were each significantly associated with a lower odds of second stage ≥1.5 hours (Figure). Other contraction features including frequency, duration, resting tone, and tachysystole were not significant.

**Conclusion**: Peak contraction pressure and MVUs in the first stage were modestly associated with second stage duration. In contrast, contraction frequency, duration, resting tone, and tachysystole were not. These findings highlight the need to further study measures of contraction strength, rather than frequency, during second stage management.

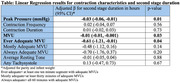

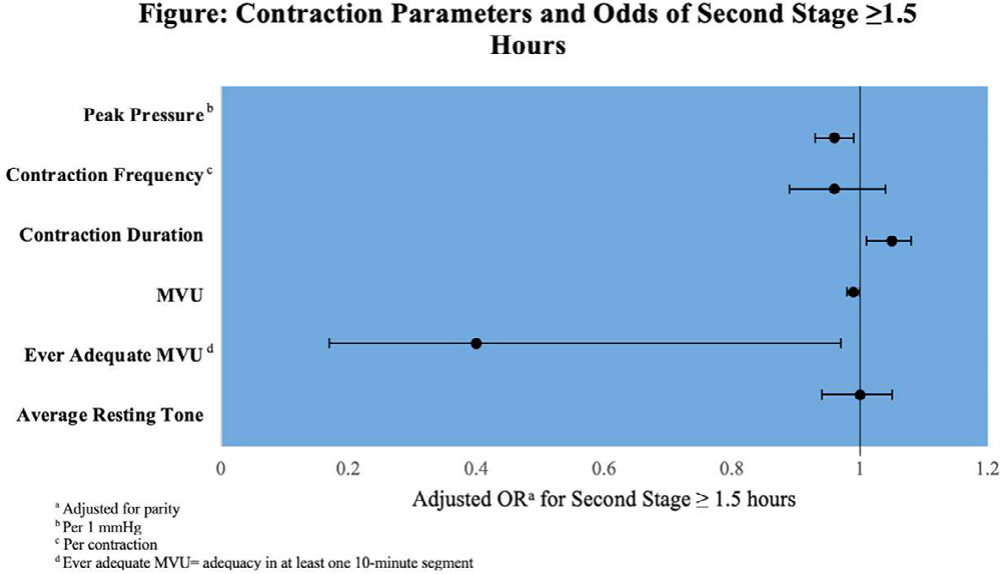

**3:30 PM – 5:00 PM**

## 1247 | **Gut Microbial Metabolite and Fractional Excretion Profiles in Pregnancies With and Without Hypertensive Disorders**

Sarah C. Graves^1^; Uma Perni^2^; Stanley Hazen^3^; Adina R. Kern‐Goldberger^4^; Jodi Meridieth^2^; Megan R. Ansbro^5^; Xinmin Li^2^; Deepthi Mallela^6^; Madeline Joquico^2^; Cara D. Dolin^7^

^1^VCU School of Medicine, VCU Health, Richmond, VA; ^2^Cleveland Clinic, Cleveland, OH; ^3^Cleveland Clinic, Cleveland, OH; ^4^Cleveland Clinic Foundation, Cleveland Clinic Foundation, OH; ^5^Cleveland Clinic Foundation, Cleveland Clinic Foundation, Cleveland, OH; ^6^Cleveland Clinic Lerner Research Institute, Cleveland Clinic, OH; ^7^Cleveland Clinic, Clevelend, OH

**Objective**: Evaluate proatherogenic and cardioprotective gut microbial metabolites and fractional excretion (FE) across pregnancy in individuals developing hypertensive disorders of pregnancy (HDP) compared to those without.

**Study Design**: Prospective cohort study enrolling patients ≥ 18 years with singleton pregnancy between 10‐14 weeks’ gestation. Paired maternal blood and urine were collected at 10‐14 weeks (visit 1), 24‐28 weeks (visit 2), and at time of delivery (visit 3). Clinical variables including pregnancy outcomes were collected. Assays for gut microbial metabolites, trimethylamine‐N‐oxide (TMAO), phenylacetylglutamine (PAG), indole acetic acid (IAA), and indole‐3‐propionic acid (I3PA), were performed using isotope dilution liquid‐tandem mass spectrometry (LC‐MS/MS) methods. Metabolite levels and FE ratios were summarized and compared across gestation between individuals who developed HDP and those who did not.

**Results**: Serum PAG did not differ between groups for any time point and increased from visit 1 to 3 in both groups. Serum TMAO did not differ between groups for any time point. There was a nonsignificant trend of increasing TMAO at visit 3 in the HDP group, while nonHDP decreased slightly. IAA trended lower in visit 1 and visit 3 and was significantly lower in visit 2 in HDP. I3PA trended lower in visit 1 and 2 for HDP and was significantly lower at visit 3. I3PA seemed to decrease more in HDP than nonHDP. FE of PAG, TMAO, and IAA decreased across gestation. I3PA could not be detected.

**Conclusion**: Lower levels of cardioprotective metabolites I3PA and IAA were observed among those who developed HDP, particularly in mid and late pregnancy. Proatherogenic metabolites TMAO and PAG did not differ by HDP status. FE of metabolites declined across gestation, suggesting gestational changes in renal handling, microbiome or host metabolism. This highlights potential early microbial signatures of vascular dysfunction and underscore the need to further study the gut‐vascular axis in HDP.

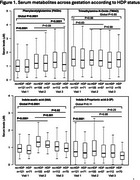

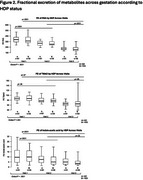

**3:30 PM – 5:00 PM**

## 1248 | **Early‐Life Parenting Patterns: Understudied, Potentially Modifiable Risk Factors for Adverse Obstetric Outcomes**

Sarah Heerboth^1^; Ashlyn Tolbert^2^; Emily Gascoigne^2^; Carmen Beamon^3^; Akila Subramaniam^4^; Annie Dude^2^; Rebecca Fry^2^; Tracy A. Manuck^2^

^1^University of North Carolina ‐ Chapel Hill, UNC Chapel Hill, Chapel Hill, NC; ^2^University of North Carolina, Chapel Hill, NC; ^3^WakeMed, Raleigh, NC; ^4^Center for Research in Women's Health, University of Alabama at Birmingham, Birmingham, AL

**Objective**: Early adverse childhood experiences are associated with long‐term health and OB outcomes, but the impact of recalled parental bonding is poorly understood. We quantified the impact of parenting patterns on adverse OB outcomes.

**Study Design**: Secondary analysis of a prospective cohort enriched for spontaneous PTB risk. Multiparous patients with singleton gestations enrolled <22 weeks, reported whether they had maternal/paternal parental figures < age 17, and completed maternal ± paternal Parental Bonding Instruments (PBI) as applicable. PBI scores quantified patient‐recalled care and protection parenting styles.  Parental exposures = absent parental figure, high care, high overprotection, and “optimal parenting” (high care/low overprotection). The primary outcome was the Cumulative Obstetric Risk Index (CORI), designed to reflect the frequency and severity of OB outcomes by considering the % of pregnancies delivered preterm, the earliest pregnancy delivery GA, and number of pregnancies complicated by preeclampsia and SGA. CORI scores range 0–12; higher scores reflect worse obstetric histories. Associations between parenting patterns and CORI scores (and its components) were assessed using linear regression.

**Results**: Of 436 participants, 22% reported an absent parent; 337 completed both PBIs. Mean CORI score was 4.9. Those with care and protection scores corresponding to “optimal parenting” from both maternal and paternal figures had lower CORI scores and fewer adverse OB outcomes (Table). Paternal overprotection and absence were linked to higher CORI scores, earlier deliveries, and increased SGA risk. In adjusted models, paternal overprotection remained significantly associated with higher CORI scores (β = 0.82, 95% CI 0.13–1.51, *p* = 0.015).

**Conclusion**: Recalled parenting patterns, particularly paternal overprotection, are novel and under‐recognized contributors to OB risk, underscoring the long‐term impact of early‐life psychosocial exposures. Promoting positive parenting through targeted support and education may alter these potentially modifiable patterns and improve OB outcomes.

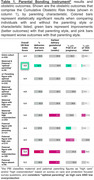

**3:30 PM – 5:00 PM**

## 1249 | **Timing of Diabetes Diagnosis and Perinatal Outcomes: A Secondary Analysis of the MOMPOD Trial**

Liza Hill^1^; Kim Boggess^2^; Ashley N. Battarbee^3^

^1^University of Alabama at Birmingham, Birmingham, AL; ^2^University of North Carolina at Chapel Hill, Chapel Hill, NC; ^3^Center for Research in Women's Health, University of Alabama at Birmingham, Birmingham, AL

**Objective**: ACOG and ADA recommend screening for undiagnosed pregestational diabetes (DM) at the first prenatal visit, but it is unclear if outcomes differ based on timing of DM diagnosis. Our objective was to compare outcomes in pregnant patients with type 2 DM diagnosed before pregnancy and in early pregnancy.

**Study Design**: Secondary analysis of a randomized controlled trial of metformin for insulin‐treated type 2 DM in pregnancy. We included participants with data on diagnosis timing, initial HbA1c and the neonatal composite (Table 2). Baseline characteristics and outcomes were compared between those with DM diagnosed before pregnancy versus during pregnancy <23 weeks. We used HbA1c‐matched conditional regression, propensity score matching and multivariable logistic regression to adjust for confounding.

**Results**: Of 686 eligible participants, 428 were included in the HbA1c‐matched cohort: 111 (26%) diagnosed during pregnancy and 317 (74%) before pregnancy. Mean HbA1c was 7.2±1.6% (range 4.8‐12.4%) with no difference between groups after matching (Table 1). Compared with diagnosis before pregnancy, participants diagnosed during pregnancy enrolled at a later GA and had lower baseline insulin doses (Table 1). The neonatal composite was similar between groups (58% v 69%; aOR 0.61, CI 0.37‐1.01). Diagnosis during pregnancy was associated with lower odds of low birthweight and NICU admission (Table 2). Similar findings were observed with propensity score matching (1^0^ outcome aOR 0.66, CI 0.43‐1.00), traditional multivariable regression (aOR 0.62, CI 0.37‐1.02) and after restricting to patients diagnosed during pregnancy by HbA1c or fasting glucose (aOR 1.01, 95% CI 0.53‐1.90).

**Conclusion**: In a large, diverse cohort we found that neonatal morbidity was similar among those with preexisting DM and DM diagnosed in early pregnancy after accounting for baseline HbA1c, underscoring the importance of DM screening in early pregnancy. Although low birthweight and NICU admission may be less common with DM diagnosed in pregnancy, patients may benefit from similar counseling and care as those diagnosed preconception.

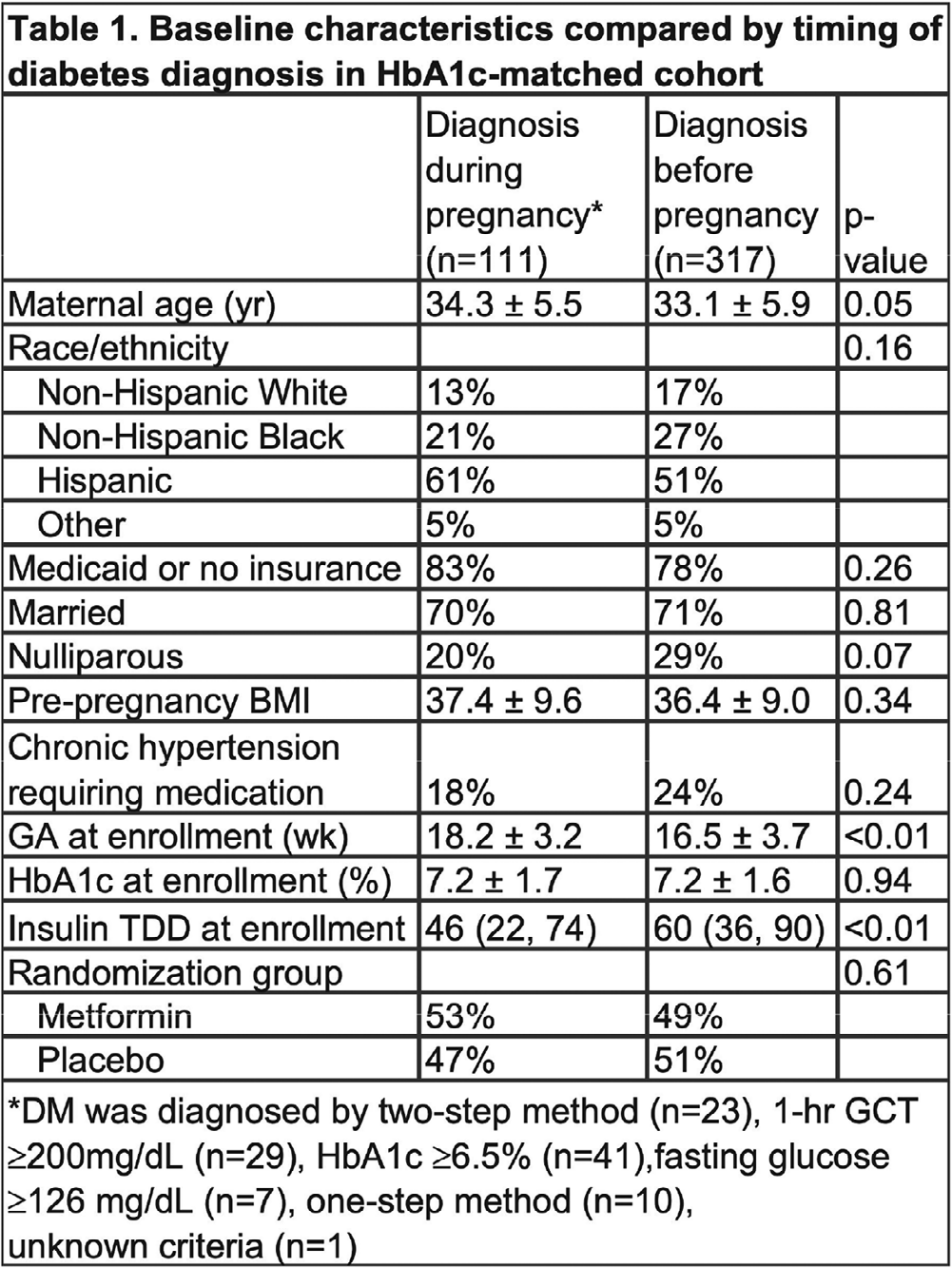

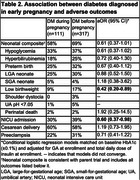

**3:30 PM – 5:00 PM**

## 1250 | **Vaginal Birth Following Second Stage Arrest Cesarean: A Worthwhile Trial?**

Sarah Martinez^1^; Avery Cox^2^; Bridget M. Donovan^3^; Michelle J. Wang^4^; Anna M. Modest^4^; Cassandra R. Duffy^4^

^1^Beth Israel Deaconess Medical Center, Beth Israel deaconess Medical Center, MA; ^2^Beth Israel Deaconess Medical Center, Beth Israel Deaconess Medical Center, MA; ^3^University of Virginia, University of Virginia, VA; ^4^Beth Israel Deaconess Medical Center, Boston, MA

**Objective**: Cesarean (CS) in the second stage of labor may impact future reproductive health, including increasing risk of preterm birth.  We sought to assess subsequent pregnancy outcomes after second stage CS compared to CS for breech presentation.

**Study Design**: This is a retrospective cohort study of nulliparous patients undergoing a CS from 2017 to 2020 with a subsequent pregnancy at our institution. We compared outcomes in those undergoing CS for second stage arrest to those with CS for breech presentation. Baseline demographic data and delivery characteristics were abstracted. Main outcomes were mode of delivery and length of second stage in a subsequent pregnancy. Secondary outcomes included preterm birth, other delivery characteristics, and neonatal outcomes.

**Results**: 180 patients with second‐stage CS and 35 patients with breech CS were included. Among subsequent livebirths, those with primary CS in the second stage were less likely to have a vaginal birth after cesarean (VBAC) (22.2% vs. 39.4% *p* = 0.04) (Table). There was no difference in median length of the second stage in subsequent VBACs (66 vs. 77 min, *p* = 0.26). Among those with successful VBAC, patients with prior second stage CS were more likely to present in spontaneous labor (86.5 vs 53.8%, *p* = 0.01); this trend was also seen among repeat cesareans but was not significant (26.2 vs. 15.0%, *p* = 0.51). For those who underwent repeat CS, patients with a  prior second stage CS had greater estimated blood loss (median EBL 750 mL vs 600 mL, *p* = 0.003). There were no differences in rates of preterm birth, markers of cervical insufficiency, or neonatal outcomes between groups.

**Conclusion**: We found that about 1 in 5 patients with initial CS for second stage arrest went on to have a vaginal birth. While rates of VBAC were lower in this group compared to initial CS for breech, there were higher rates of spontaneous labor and no difference in second stage length.

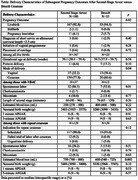

**3:30 PM – 5:00 PM**

## 1251 | **Risk Stratification Model Identifies Unplanned Deliveries <34 Weeks in Placenta Accreta Spectrum**

Sarah T. Mehl^1^; Edgar A. Hernandez‐Andrade^1^; Syed Hashmi^2^; Karla M. Martin^3^; Sandra Sadek^3^; Zakaria Doughan^4^; Elias Kassir^1^; Ioana Bondre^5^; Ahmed Zaki Moustafa^6^; Sean C. Blackwell^1^; Baha M. Sibai^1^; Farah H. Amro^1^

^1^Division of Maternal‐Fetal Medicine, Department of Obstetrics, Gynecology and Reproductive Sciences, McGovern Medical School at UTHealth Houston, Houston, TX; ^2^Professor, Director Bioinformatics Core, Pediatrics Research Center, Department of Pediatrics, McGovern Medical School UTHealth Houston, Houston, TX; ^3^Department of Obstetrics, Gynecology and Reproductive Sciences, McGovern Medical School at UTHealth Houston, Houston, TX; ^4^Department of Obstetrics and Gynecology, McGovern Medical School at UT Health, Houston, Houston, TX; ^5^Division of Gynecology Oncology, Department of Obstetrics, Gynecology and Reproductive Sciences, McGovern Medical School at UTHealth Houston, Houston, TX; ^6^Division of Maternal‐Fetal Medicine, Department of Obstetrics, Gynecology and Reproductive Sciences, McGovern Medical School at UTHealth Houston, University of Texas ‐ Houston, TX

**Objective**: Optimal timing of delivery in pregnancies complicated by placenta accreta spectrum (PAS) remains challenging. Neonatal benefit of prolonging pregnancy must be weighed against risk of maternal morbidity with emergent delivery. Our objective was to create a risk stratification model for prediction of unplanned delivery <34 weeks in pregnancies complicated by PAS.

**Study Design**: Retrospective cohort study of pregnancies with suspected PAS by antenatal ultrasound from 2015–2025 and delivered within our hospital system. Pregnancies were evaluated in two groups: unplanned delivery <34 weeks and planned delivery ≥ 34 weeks. Data was categorized and modified Poisson regression models were utilized for crude and adjusted incidence risk ratios. A subset of predictor variables was utilized to create a risk scoring system with binary variables adding to a total score ranging from 0 to 7. Pregnancies were stratified into low (score< 2), moderate (score = 2), or high risk (score >2) for unplanned delivery <34 weeks. Receiver operator characteristic (ROC) analysis was utilized to assess predictive ability of this risk stratification model.

**Results**: Among 131 pregnancies with suspected PAS, adjusted models identified a significantly increased risk of unplanned delivery <34 weeks in pregnancies BMI >30, Hispanic ethnicity, cervical length <3cm in third trimester, ultrasound suspicion percreta, admission for preterm labor (Table 1). A predictive model was developed for risk stratification using variables listed in Table 2. This model resulted in a ROC area under the curve (AUC) of 0.79 (95% CI: 0.69–0.89), with 81% of cases correctly classified at the threshold of “high risk” and a specificity of 93%. Pregnancies classified as high risk were 2.5x more likely (RR: 2.47, 95% CI: 1.17‐5.21) to have an unplanned preterm delivery compared to low‐risk pregnancies.

**Conclusion**: Our model had an AUC of 0.79 and correctly identified 81% of unplanned preterm <34 week deliveries, with a low false negative rate. This predictive ability holds significant clinical value when faced with this life‐threatening obstetric condition.

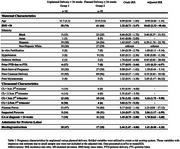

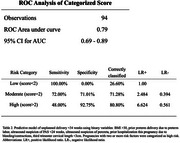

**3:30 PM – 5:00 PM**

## 1252 | **Conservative Management With Planned Placental Removal Versus Hysterectomy for Antenatally Suspected Low‐Grade Placenta Accreta Spectrum**

Sarah E. Miller^1^; Rishi B. Chopra^2^; Hope Y. Yu^3^; Ved Topkar^1^; Nicola Perlman^4^; Daniela A. Carusi^5^

^1^Brigham and Women's Hospital, Boston, MA; ^2^Brigham and Women's Hospital, Fremont, CA; ^3^Department of Obstetrics and Gynecology, MassGeneral Brigham; Division of Maternal‐Fetal Medicine; Harvard Medical School, Bostton, MA; ^4^University of California San Francisco, San Francisco, CA; ^5^Department of Obstetrics and Gynecology, MassGeneral Brigham; Division of Maternal‐Fetal Medicine; Harvard Medical School, Boston, MA

**Objective**: Uterine conservation for placenta accreta spectrum (PAS) may involve placental retention to avoid placental extraction morbidity which often confers an increased risk of delayed hemorrhage and infection. We describe a cohort of suspected lower‐grade PAS patients managed with planned placental removal and availability of uterine artery embolization (UAE) compared to planned hysterectomy.

**Study Design**: Between 2016‐2024 a cohort of patients with radiographic suspicion of low‐grade PAS (lacked signs of Percreta/Grade 3 PAS) underwent conservative management with availability of UAE at a single tertiary hospital. Outcomes were compared to those with similar radiologic and clinical criteria who underwent planned hysterectomy without placental removal. Those with singleton pregnancies >24w GA with clinically confirmed PAS were analyzed.

**Results**: We identified 73 eligible patients, 18 planning conservative management (CM) and 55 undergoing planned hysterectomy (PH). Demographics were similar between groups aside from parity, prior c‐sections and BMI (Table 1). Delivery outcomes (Table 2) were similar including hemorrhage requiring RBC transfusion ≥4 units, procedure times and general anesthesia use. There were no delayed hysterectomies or readmissions for infection in the CM group. The placenta was entirely removed in 78% of CM cases and partially removed during delivery in 17% of CM and 29% of PH cases (*p* < 0.01). Uterine retention was successful in 89% of CM cases with UAE performed in 67% of cases. Six of 18 CM patients (33%) had at least one subsequent pregnancy, 83% of which resulted in a viable birth. Rates of recurrent hemorrhage and PAS were both 67%, and there was one hysterectomy.

**Conclusion**: In patients with radiographically suspected lower‐grade PAS, uterine conservation with placental removal was successful in 89% of clinically confirmed PAS patients with 67% having peri‐delivery UAE. Delivery outcomes were similar when compared to hysterectomy. Antenatally planned peripartum embolization may facilitate the safe execution of uterine conservation with placental removal in a subset of PAS patients.

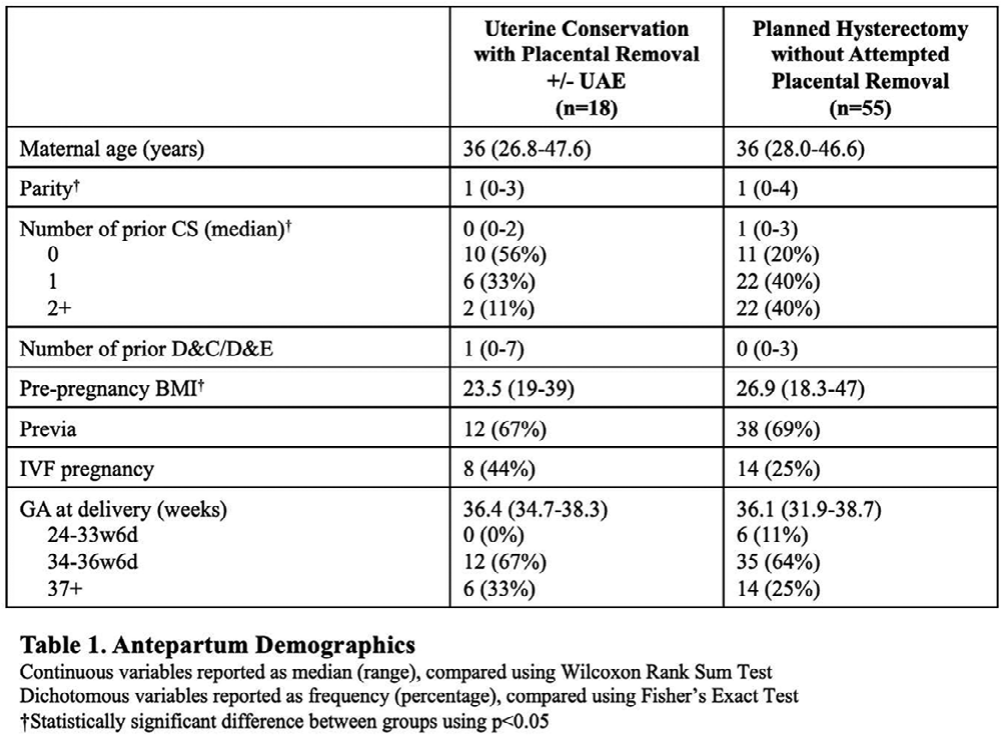

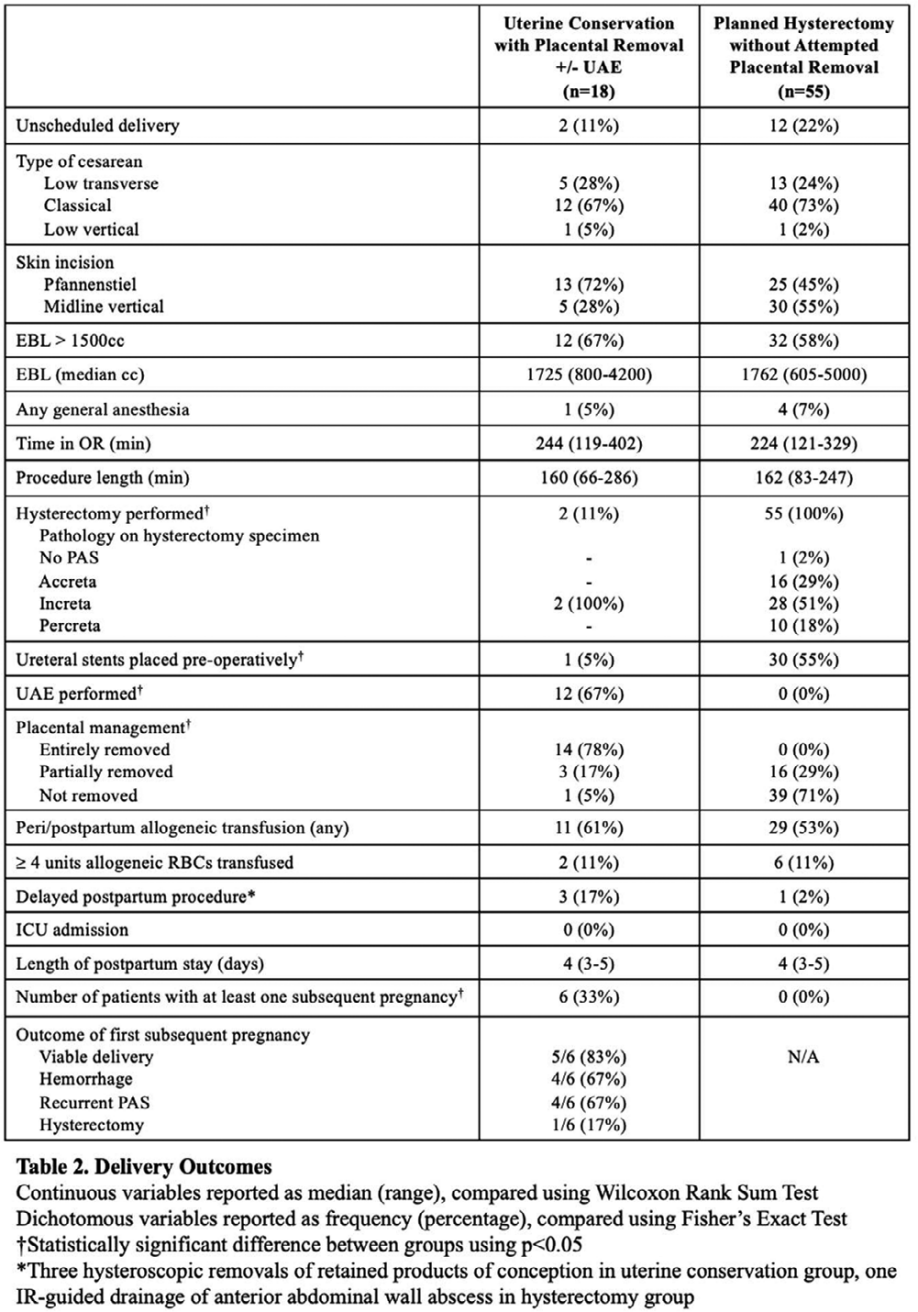

**3:30 PM – 5:00 PM**

## 1253 | **Relationship Between Continuous Glucose Sensor Metrics and Adverse Neonatal Outcomes in Individuals Without Diabetes**

Sarah Nazeer^1^; Rafael Bravo^2^; Claudia Pedroza^2^; Baha M. Sibai^2^; Sean C. Blackwell^2^; Michal Fishel Bartal^2^

^1^Cleveland Clinic Lerner College of Medicine, Cleveland Clinic Lerner College of Medicine, OH; ^2^Division of Maternal‐Fetal Medicine, Department of Obstetrics, Gynecology and Reproductive Sciences, McGovern Medical School at UTHealth Houston, Houston, TX

**Objective**: The data regarding normal glucose values using a continuous glucose monitoring (CGM) in people without diabetes in pregnancy is limited. We aimed to evaluate the relationship between CGM metrics in individuals without diabetes and adverse neonatal outcomes.

**Study Design**: This was a secondary analysis of the PRECISE trial in which participants were randomly assigned at 24–30 weeks’ gestation to either 7‐day CGM or the routine, 2‐step GDM screening method; the intervention group also had blinded CGM placed (NCT05430204). This analysis included individuals without GDM who had complete CGM data. We examined adverse outcomes including large for gestational age (LGA), need for IV/PO glucose, and respiratory distress (RDS), using the Youden index to determine cutoffs for various CGM metrics: %TAR >140 mg/dL, %TAR >130 mg/dL, mean glucose, glucose coefficient of variation (CV), and the 1‐hour glucose challenge test (GCT).

**Results**: Of 814 participants, 644 (79%) met inclusion criteria. Most were obese, identified as non‐white, and 8% had preexisting hypertension (Table). The average glucose was 104.2 mg/dL (±11.5) with 90.7% of cohort within the time in range. The entire cohort was above range 5.6% (Table 1) of the time. AUCs for LGA were TAR >140 and >130mg/dL, 0.59; mean glucose, 0.58; CV, 0.49; TAR >5%, 0.53; TAR >10%, 0.56; 1‐hour GCT, 0.49. AUCs for RDS: TAR >140, 0.58; TAR >130, 0.57; mean glucose, 0.55; CV, 0.59; TAR >5%, 0.56; TAR >10%, 0.53; GCT, 0.54. AUCs for IV/PO glucose need: TAR >140, 0.50; TAR >130, 0.50; mean glucose, 0.51; CV, 0.54; TAR >5%, 0.53; TAR >10%, 0.51; GCT, 0.46 (Figure).

**Conclusion**: In this secondary analysis, neither CGM metrics nor the 1‐hour GCT reliably predicted adverse neonatal outcomes. Larger studies are needed to determine the optimal GDM screening examination

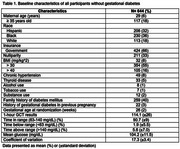

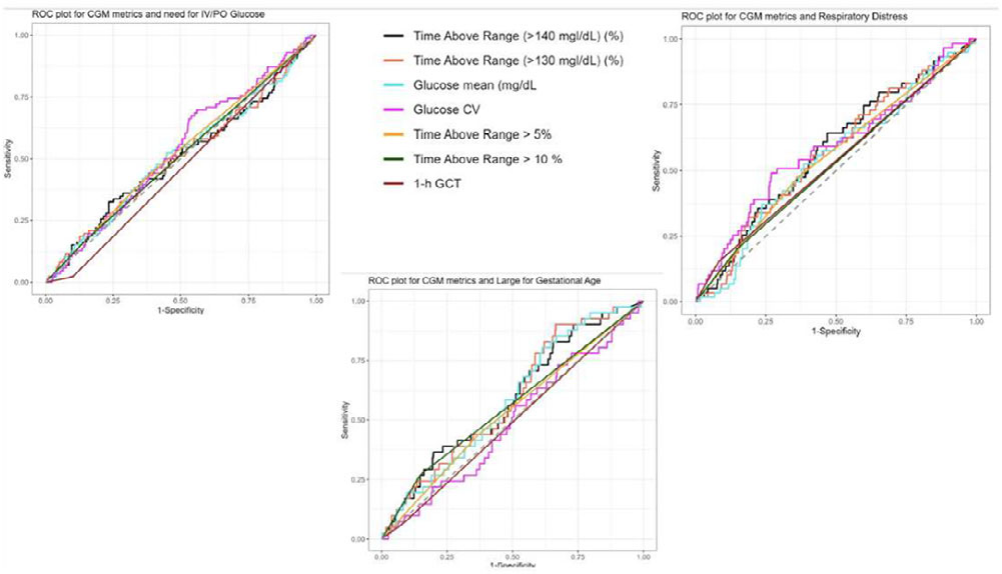

**3:30 PM – 5:00 PM**

## 1254 | **Routine Third‐Trimester Ultrasonography in Low‐Risk Pregnancies: Examining Detection of Abnormalities in a High‐Risk Population**

Zeeraq Rana^1^; Brianna Dawson^2^; Ryan Uchimura^1^; Sarah D. Smithson^1^

^1^Riverside University Health System, Moreno Valley, CA; ^2^University of California Riverside School of Medicine, Riverside, CA

**Objective**: Current guidelines from the American College of Obstetricians and Gynecologists (ACOG) recommend monitoring fetal growth and amniotic fluid status in low‐risk pregnancies through serial fundal height measurements beginning at 24 weeks gestation. Ultrasound is not routinely performed after the second‐trimester anatomy scan unless a discrepancy in fundal height raises clinical concern. This study aims to evaluate the utility of implementing routine third‐trimester ultrasounds in low‐risk pregnancies as a strategy to improve detection of fetal abnormalities and potentially enhance perinatal outcomes in an underserved, high‐risk population.

**Study Design**: At our MFM clinic, universal 3^rd^ trimester ultrasounds were implemented for all patients in 2022. A retrospective study was performed of all singleton pregnancies in the study period that underwent a 2^nd^ trimester anatomy ultrasound and a 3^rd^ trimester repeat between 28‐32 weeks gestation. Patients were excluded if a 3^rd^ trimester ultrasound or serial growth scans would have been indicated due to maternal risk factors, fetal risk factors, abnormalities on 2^nd^ trimester ultrasound, etc. The remaining routine 3^rd^ trimester ultrasounds were reviewed for clinically significant abnormalities.

**Results**: Of 743 routine 3^rd^ trimester ultrasounds, 123 (16.6%) revealed new relevant findings. The most frequent findings were large abdominal circumference with normal estimated fetal weight (31.7%), macrosomia (27.6%), and intrauterine growth restriction (22.8%). Other detected anomalies included cardiac (4.9%), umbilical/placental (6.5%), cranial (2.4%), amniotic fluid (2.4%), extremity (1.6%), and intra‐abdominal (1.6%).

**Conclusion**: Routine 3^rd^ trimester ultrasounds in low‐risk pregnancies detected clinically significant abnormalities in over 1 in 6 patients. Early detection of fetal growth and structural anomalies in this underserved population may inform care decisions and improve outcomes. These findings add to the growing body of evidence that supports the clinical value of integrating routine 3^rd^ trimester scans in settings with a high risk patient population.

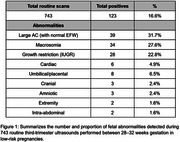

**3:30 PM – 5:00 PM**

## 1255 | **HIV and Pregnancy Outcomes: An Updated Retrospective Cohort Analysis**

Sarena Hayer^1^; Bharti Garg^2^; Jamie O. Lo^2^; Aaron B. Caughey^2^; Irina Cassimatis^2^

^1^Cedars‐Sinai Health Sciences University, Los Angeles, CA; ^2^Oregon Health & Science University, Oregon Health & Science University, OR

**Objective**: While human immunodeficiency virus (HIV) in the United States (US) has declined, HIV in pregnancy remains a relevant clinical concern. Historically, pregnancies complicated by HIV‐positive status have been associated with adverse perinatal outcomes. Despite improvements in screening and timely treatment, recent data on perinatal outcomes among pregnant individuals living with HIV remain limited. The objective of this study is to examine the association between HIV‐positive status in pregnancy and adverse outcomes in a contemporary cohort.

**Study Design**: This is a retrospective cohort study using linked vital statistics and hospital discharge data for births in California (2008‐2020). We included HIV‐positive pregnant individuals with a singleton gestation who delivered between 23 and 42 weeks. HIV‐positive status was identified using ICD‐9 (0.42x) and ICD‐10 (B20) codes. Chi‐square tests and multivariable Poisson regression models were used to assess risk of outcomes.

**Results**: Compared to those without HIV, HIV‐positive pregnant individuals (*n* = 552) were more likely to be 35 years or older (29.5% vs. 20.5%; *p* < 0.001) and non‐Hispanic Black (27.7% vs. 4.9%; *p* < 0.001). They also had less prenatal care (5.0% vs. 2.4%; *p* < 0.001), attained high school or less education (64.9% vs. 42.8%; *p* < 0.001), and were more likely to be publicly insured (84.1% vs. 51.6%; *p* < 0.001). On multivariate regression, HIV‐positive status was associated with increased risk of placental abruption (aRR = 1.87, 95% CI 1.10, 3.18), postpartum hemorrhage (aRR = 2.19, 95% CI 1.61, 2.96), and severe maternal morbidity (aRR = 3.72, 95% CI 2.68, 5.17). HIV‐positive status was also associated with increased risk of preterm birth <37 weeks (aRR = 1.94, 95% CI 1.61, 2.34), neonatal intensive care unit (NICU) admission (aRR = 1.86, 95% CI 1.60, 2.16), and respiratory distress syndrome (aRR = 2.25, 95% CI 1.68, 3.01).

**Conclusion**: Despite changes to the HIV landscape in the US, HIV‐positive status remains associated with increased risk of perinatal outcomes.

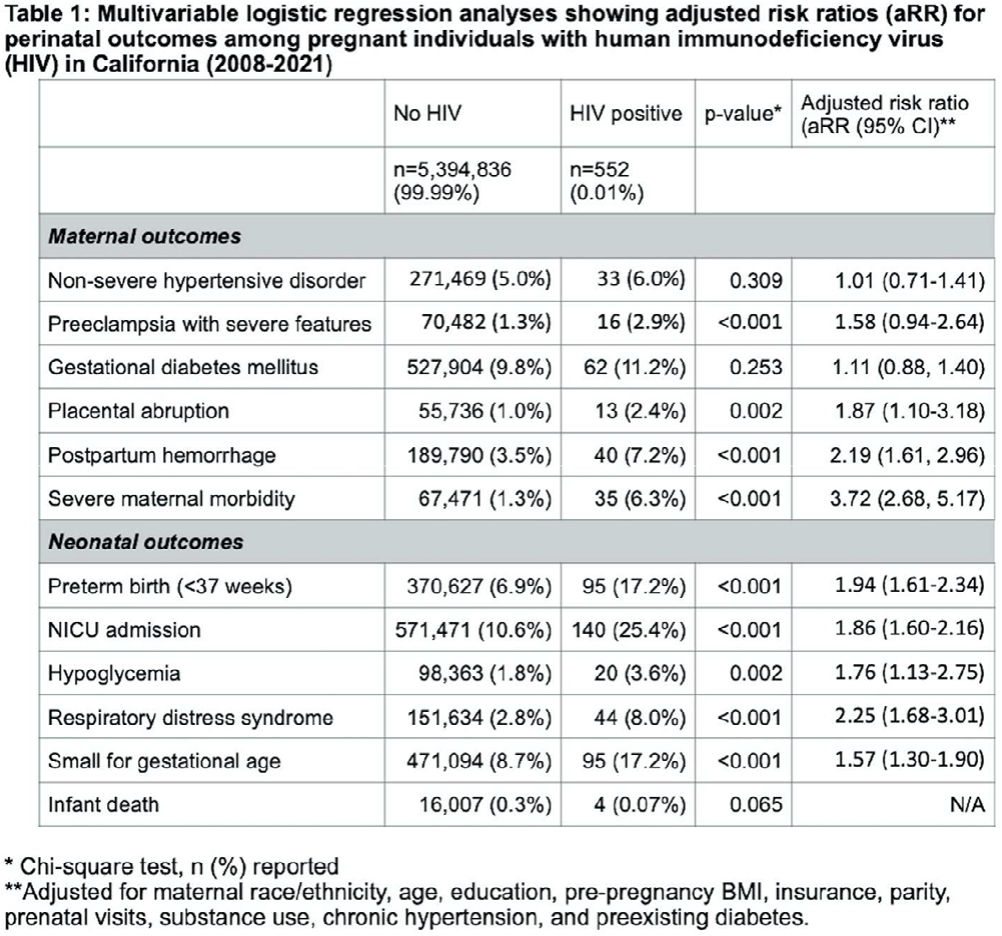

**3:30 PM – 5:00 PM**

## 1256 | **Fetal Heart Tracings With and Without Uterine Rupture During Trial of Labor After Cesarean**

Sharon Stortz^1^; Megan Stevenson^2^; Rachel D. Seaman^1^; Leslie Tseng^3^; Austin Oberlin^1^; Lauren Walheim^4^; Lisbet S. Lundsberg^3^; Jennifer F. Culhane^3^

^1^Yale School of Medicine, New Haven, CT; ^2^Yale New Haven Hospital, New Haven, CT; ^3^Department of Obstetrics, Gynecology, and Reproductive Sciences, Yale School of Medicine, New Haven, CT; ^4^Yale School of Medicine, Yale School of Medicine ‐ Yale University, CT

**Objective**: To evaluate if temporal differences in fetal heart rate tracing (FHT) during trial of labor after cesarean (TOLAC) can indicate impending uterine rupture (UR).

**Study Design**: ICD codes were used to identify TOLAC UR cases and controls of singleton patients in a multi‐hospital system from 2013–2025. Medical eligibility for TOLAC, intent to TOLAC and presence of ≥ 60 minutes of FHT for analysis was confirmed by chart review. Obstetricians blinded to maternal characteristics and UR outcome reviewed and quantified FHTs using standardized definitions per the American College of Obstetricians and Gynecologists. FHTs were reviewed in four 10‐minute time periods: admission, 60, 30 and 10 minutes prior to delivery.  Either chi‐square or Fisher's exact test, as appropriate, were used to compare patient attributes and FHT characteristics across the UR and control groups.

**Results**: Of 142 UR cases, 77 were included and compared to 70 controls (total analytic cohort *n* = 147). There was no significant difference in mean gestational age at delivery.  UR cases were less likely to be advanced maternal age (*p* =  .003) or have a previous vaginal delivery (*p* =  .006) (Table 1).  There were no significant differences in FHT measures on admission or at 60 minutes prior to delivery between groups. At 30 minutes, UR cases were more likely to have late decelerations (*p* =  .02) compared to controls. At 10 minutes prior to delivery, cases were more likely to have an abnormal baseline (*p* =  .006) and less contractions (*p* =  .006) (Figure 1).

**Conclusion**: FHT differences preceding UR in patients undergoing TOLAC may be subtle and dynamic. Notably, there was no difference in variable decelerations across cases and controls, contrary to common teachings. Cases were more likely to have late decelerations at 30 minutes prior to delivery, but this difference did not persist in the final 10 minutes of labor. Our observed UR FHT characteristics during the final 10 minutes of labor, possibly indicating loss of uterine tone and fetal distress, suggest clinically significant FHT changes may not be evident until uterine rupture occurs.

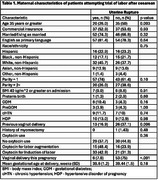

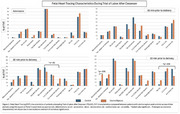

**3:30 PM – 5:00 PM**

## 1257 | **Statewide Prevalence of Screening for Unmet Social Needs Across Prenatal Clinics and Associated Clinic Characteristics**

Shayla Parthasarathy^1^; Jessica Yaser^2^; Rinat Tal^1^; Rachel Clark^3^; Briaa Baldwin^4^; Annise Reynolds^5^; Caitlyn Clock^5^; Michelle Moniz^4^; Jourdan E. Triebwasser^5^; Lisa Kane Low^5^; Alex Peahl^6^

^1^University of Michigan Medical School, Ann Arbor, MI; ^2^University of Michigan Medical Center, Ann Arbor, MI; ^3^Trinity Health Ann Arbor, Trinity Health Ann Arbor, MI; ^4^University of Michigan, University of Michigan, MI; ^5^University of Michigan, Ann Arbor, MI; ^6^University of Michigan Department of Obstetrics & Gynecology, Ann Arbor, MI

**Objective**: A new American College of Obstetricians and Gynecologists’ Clinical Consensus for Tailored Prenatal Care recommends a comprehensive needs assessment at the beginning of pregnancy, including systematically screening for unmet social needs with a standardized tool. We describe the rates of systematic social needs screening in a sample of Michigan prenatal clinics, and the clinic characteristics associated with systematic screening.

**Study Design**: Prenatal clinics in Michigan were identified through web searches and snowball sampling. Semi‐structured surveys were completed with a clinical and administrative lead for 58 clinics to assess their clinic practices for tailored prenatal care components, including systematic screening for unmet social needs. We assessed characteristics of clinics that did and did not use systematic screening, including clinic structure (Federally Qualified Health Centers, outpatient clinics linked to hospitals, and private practices) and clinic patient volume, using Fisher's exact tests of comparison.

**Results**: Of the 58 clinics represented, 51 (51/58, 88%) screened for unmet social needs, with 42 (42/58, 72%) utilizing a standardized screening tool. Clinic structure was associated with systematic screening for unmet social needs (*p* = 0.004), with higher rates for Federally Qualified Health Centers (FQHCs) and clinics linked with hospitals. Clinic patient volume was not associated with systematic screening (*p* = 0.9).

**Conclusion**: Many prenatal clinics in Michigan systematically screen patients for unmet social needs. Some clinics, such as FQHCs and outpatient clinics linked to hospitals, may have more robust infrastructure for completing systematic screening. Further work is needed to identify context specific supports needed to implement social needs screening for tailoring prenatal care.

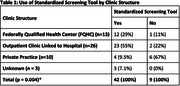

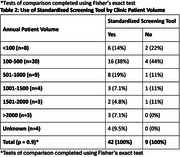

**3:30 PM – 5:00 PM**

## 1258 | **Single Umbilical Artery: Marker of Risk or a Benign Variant? A Long‐Term Neurologic Follow‐Up**

Shayna Miodownik^1^; Gil Gutvirtz^2^; Tamar Wainstock^3^; Eyal Sheiner^4^

^1^Soroka University Medical Center, Soroka University Medical Center, HaDarom; ^2^Department of Obstetrics and Gynecology, Soroka University Medical Center, Ben‐Gurion University, Metar, HaDarom; ^3^Department of Epidemiology, Biostatistics and Community Health Sciences, Faculty of Health Sciences, Ben‐Gurion University of the Negev, Beer Sheva, HaDarom; ^4^Soroka University Medical Center, Faculty of Health Sciences, Ben‐Gurion University of the Negev, beer sheva, HaDarom

**Objective**: The clinical significance of a single umbilical artery (SUA) remains a subject of ongoing investigation. SUA is recognized as a potential marker for chromosomal and congenital abnormalities. However, even when SUA is an isolated finding, its relationship with perinatal morbidity remains inconclusive, and data on the long‐term outcomes for the offspring is also limited. In this study, we evaluated the association between isolated SUA and long‐term neurologic morbidity in the offspring.

**Study Design**: A retrospective cohort study including singleton deliveries between 1991‐2021 was performed. Fetuses with chromosomal or congenital abnormalities were excluded. Children with SUA were analyzed against children without SUA using neurologic morbidity data from ambulatory and hospitalization records, up to 18 years of age. A Kaplan‐Meier survival curve was used to compare cumulative neurologic morbidity incidence. A Cox regression model was used to estimate the risk for long‐term neurologic morbidity, controlling for confounders.

**Results**: A total of 232,476 singleton pregnancies were included; SUA was identified in 136 of pregnancies. Offspring with SUA had higher rates of long‐term neurologic morbidity as compared to offspring without a SUA (Table). However, the cumulative incidence of neurologic morbidity over time was comparable between the groups (Figure), and applying the Cox regression model that controlled for maternal and gestational age, diabetes mellitus, hypertensive disorders, mode of delivery and small for gestational age neonates, reassured that SUA was not associated with a higher risk for offspring neurologic morbidity (aHR = 1.11, 95% CI 0.81–1.52; *p* = 0.503).

**Conclusion**: Although SUA was associated with a higher unadjusted rate of neurologic morbidity, this association disappeared after controlling for confounders. The cumulative risk over time was similar in both groups, suggesting that isolated SUA is not an independent predictor of adverse long‐term neurologic outcomes.

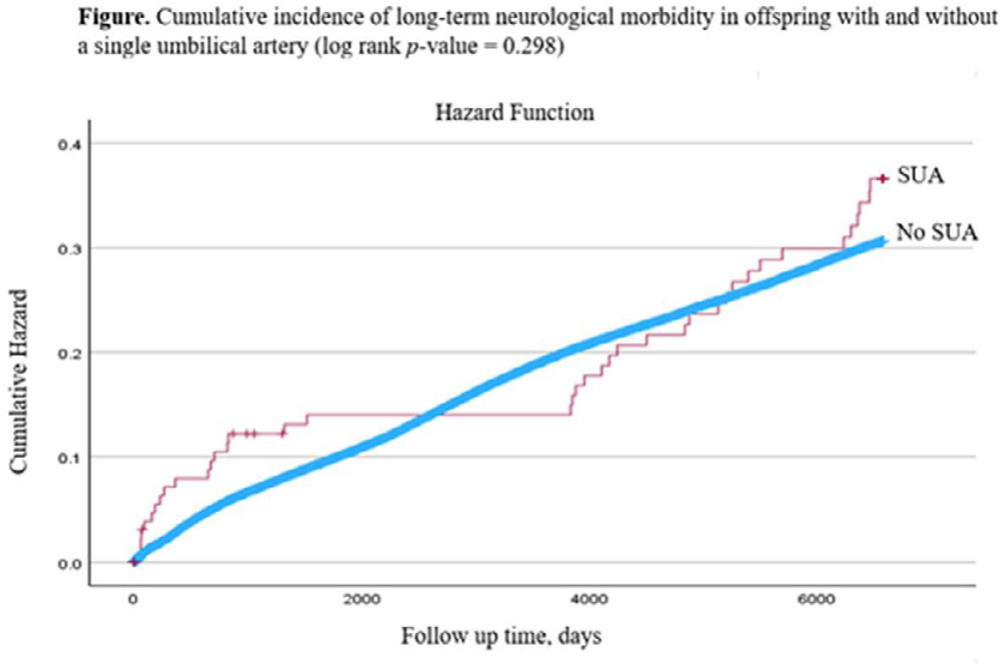

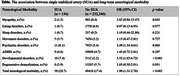

**3:30 PM – 5:00 PM**

## 1259 | **Vaping During Pregnancy: Implications for Maternal and Fetal Health**

Anna Harb^1^; Megan L. Young^2^; Jill M. Maples^3^; Jordan E. Lewis^1^; Shelley M. Stiltner^1^; Heather Moss^1^

^1^University of Tennessee Graduate School of Medicine, Knoxville, TN; ^2^University of Tennessee Medical Center, Knoxville, TN; ^3^University of Tennessee Health Science Center COM Knoxville, Knoxville, TN

**Objective**: As electronic vapor product (EVP) use becomes increasingly prevalent—particularly among younger populations—it is often perceived as a safer alternative to tobacco, despite limited data on its perinatal effects. This study aimed to assess whether EVP and/or tobacco exposure is associated with maternal factors such as psychiatric disorders and breastfeeding intentions as well as perinatal complications such as fetal growth restriction, preterm delivery, and premature rupture of membranes (PPROM), with the goal of informing clinical counseling and public health recommendations.

**Study Design**: This retrospective cohort study included pregnant individuals who delivered at the University of Tennessee Medical Center between June 2023‐April 2024. Electronic health and delivery record data was collected on EVP use, tobacco use, and maternal and neonatal outcomes. Variables included any history of psychiatric disorders, premature rupture of membranes (PPROM), preterm birth, fetal growth restriction, and breastfeeding plan on admission. Differences in maternal and fetal outcomes by tobacco and EVP usage were tested using chi square analysis. Multivariable logistic regressions were used to examine the associations between tobacco or EVP usage and fetal growth restriction, preterm delivery, and PPROM after adjusting for covariates.

**Results**: Differences in maternal fetal outcomes between the patients who did not use smoked tobacco or EVP, smoked tobacco only, EVPs only, or both are presented in Table 1. After controlled for multiple factors, EVP use increased the odds of PPROM, while smoking tobacco increased the odds of FGR, and both EVP and tobacco use increased the odds of preterm delivery (Table 2).

**Conclusion**: Among pregnant patients who reported tobacco and/or EVP usage, there are multiple associations with adverse maternal and fetal outcomes. EVPs should not be recommended as a safe alternative to tobacco use in pregnancy.

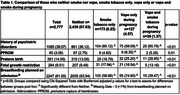

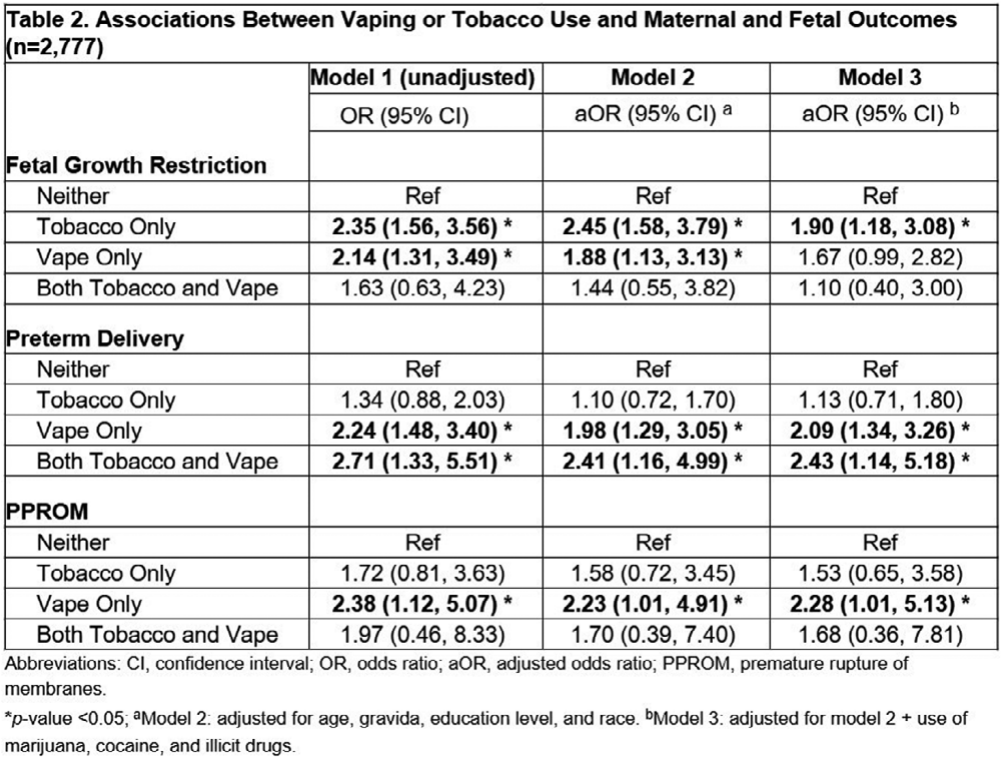

**3:30 PM – 5:00 PM**

## 1260 | **Beyond the Threshold: Clinical Implications of Subclinical Hypothyroidism in Pregnancy**

Shir Zagdon Keidar^1^; Nitzan Burrack^2^; Merav Fraenkel^3^; Uri Yoel^4^; Tamar Eshkoli^5^

^1^Soroka University Medical Center, Ben‐Gurion University of the Negev, Beer sheva, HaDarom; ^2^Clinical Research Center, Soroka University Medical Center; Faculty of Health Sciences, Ben‐Gurion University of the Negev, Beer sheva, HaDarom; ^3^Endocrinology Unit, Soroka University Medical Center; Faculty of Health Sciences, Ben‐Gurion University of the Negev, Be'er Sheva, Israel, Beer sheva, HaDarom; ^4^Endocrinology Unit, Soroka University Medical Center; Faculty of Health Sciences, Ben‐Gurion University of the Negev, Beer‐Sheva, Israel, Beer sheva, HaDarom; ^5^Soroka University Medical Center, Faculty of Health Sciences, Ben‐Gurion University of the Negev, Beer sheva, HaDarom

**Objective**: To evaluate pregnancy complications in women with subclinical hypothyroidism (mean TSH 4–10 mIU/L) using a cross‐sectional study.

**Study Design**: Data from 95,632 live birth pregnancies (2013–2022) were analyzed using the Clalit Health Services (CHS) database. The study included CHS‐insured women aged ≥18 years with available TSH results during pregnancy. Subclinical hypothyroidism was defined as a mean TSH between 4 and 10 mIU/L without a diagnosis of overt hypothyroidism. The euthyroid reference group consisted of women with TSH <4 mIU/L and no history of hypothyroidism or levothyroxine use. Women with subclinical hypothyroidism were matched to euthyroid controls using propensity score‐based matching, adjusting for maternal age, ethnicity, socioeconomic status, IVF, recurrent pregnancy loss, and smoking. Pregnancy complications were compared using descriptive statistics, univariate analysis, and quasi‐Poisson regression.

**Results**: The final analysis included 8697 subclinical hypothyroid and 86,935 euthyroid pregnancies with similar baseline characteristics. Women with subclinical hypothyroidism had slightly higher rates of severe preeclampsia (1.5% **vs**. 1.1%, *p* < 0.001), cesarean delivery (21.2% **vs**. 18.7%, *p* < 0.001), preterm birth (8.3% vs 7.3%, *p* < 0.001), assisted delivery (6.3% **vs**. 5.1%, *p* < 0.001), and low birth weight (9.3% **vs**. 7.9%, *p* < 0.001). No significant differences were observed in rate of gestational hypertension, gestational diabetes, or postpartum hemorrhage. In multivariable analysis, subclinical hypothyroidism was associated with a modestly increased risk of pregnancy complications (IRR 1.07, 95% CI 1.04–1.11; *p* < 0.001).

**Conclusion**: Subclinical hypothyroidism was associated with a small but statistically significant increase in adverse pregnancy outcomes, supporting the need for heightened surveillance and individualized clinical consideration during pregnancy.

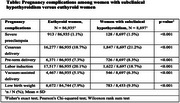

**3:30 PM – 5:00 PM**

## 1261 | **Safe Delivery of a Nanoparticle‐Based Therapy to the Pulmonary Endothelium in a Rodent Model**

Shivani Manikandan^1^; Braxton Forde^1^; Josephine Kemp^2^; Vladimir Kalinichenko^3^; Fatemeh Kohram^3^; Alan Kenny^4^

^1^University of Cincinnati College of Medicine, Cincinnati, OH; ^2^Center for Fetal and Placental Research, Cincinnati Children's Hospital Medical Center, Cincinnati, OH; ^3^University of Arizona College of Medicine, Phoenix, AZ; ^4^Cincinnati Children's Hospital Medical Center, Cincinnati, OH

**Objective**: We have previously shown that Polyethylenimine‐(5) myristic acid/ poly(ethylene glycol)‐oleic acid/cholesterol (PEI‐PEG) nanoparticles target pulmonary endothelium in adult mice and in culture. We sought to determine if PEI‐PEG nanoparticles could be used to deliver a targeted therapy in utero without disruption of normal lung development. Such a therapy could provide a breakthrough for in utero therapies for congenital pulmonary disorders as well as single gene disorders of the lungs.

**Study Design**: Dylight 647‐labeled plasmids were covalently linked to PEI‐PEG nanoparticles. These nanoparticles were subsequently injected (25 uL per fetus, total of 6 fetuses) intraperitoneally into the fetuses of wild type pregnant rodents at gestational age E17.5, with un‐injected fetuses as controls. At E20.5, fetuses were imaged via In Vivo Imaging System (IVIS) for signs of the fluorescent nanoparticles, and lung tissues were harvested for examination via flow cytometry and immunofluorescent staining. Pathologic evaluation and grading were performed.

**Results**: IVIS revealed that intrafetal intraperitoneal injections resulted in nanoparticle localization to the fetal lungs with a >10 million‐fold increase in epifluorescence over background in treated fetuses (*p* < 0.001). Immunofluorescent staining confirmed significant uptake of the nanoparticles in the fetal lung tissue (Figure 1B) without any disruption of normal fetal lung development seen on histological evaluation (Figure 1C). Flow cytometry confirmed the results, with 52% of pulmonary endothelial cells of injected fetuses having 647 fluorescence (5 injected fetuses, range 43.5%‐62.1% vs 6 controls, range 0.0‐0.2%, *p* < 0.001) vs 0% of controls.

**Conclusion**: PEI‐PEG successfully delivers fluorescent plasmids into the fetal lungs without disruption of normal fetal lung development and is a promising platform for treating pulmonary disorders in‐utero.

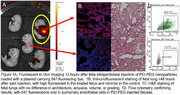

**3:30 PM – 5:00 PM**

## 1262 | **Decidual Transcriptome Profiling Unravels Key Dysregulated Genes in Cesarean Scar Ectopic Pregnancy**

Shuai Zeng; Lian Chen; Jiangxue Qu; Hai Jiang; Yangyu Zhao

Peking University Third Hospital, Beijing, Beijing

**Objective**: Cesarean scar ectopic pregnancy (CSEP), characterized by embryo implantation within a previous caesarean section (CS) scar, represent a rare yet increasingly prevalent condition. A critical concern is its frequent progression to placenta accreta spectrum (PAS) disorders, though the underlying mechanisms remain poorly understood. Emerging evidence suggests that aberrant decidualization in CSEP may create a permissive microenvironment for excessive extravillous trophoblast (EVT) invasion, thereby bridging CSEP to PAS pathogenesis. The precise molecular signatures in CSEP‐derived decidua tissues remain uncharacterized. Elucidating the decidual‐pathology‐driven mechanisms in CSEP could not only reveal early molecular triggers of PAS but also provide critical insights for risk stratification: identifying CSEP cases with potential for continued pregnancy versus those requiring intervention, and paving the way for targeted therapies to mitigate PAS progression.

**Study Design**: We performed total RNA sequencing on decidual tissues obtained from 6 CSEP and 12 normal pregnancy cases at 6‐9 weeks of gestation. Differential expressed genes (DEGs) were identified, followed by qRT‐PCR validation.Functional enrichment analysis (GO, KEGG) and immune infiltration analysis were conducted.

**Results**: Comparative transcriptomics identified 117 upregulated and 662 downregulated DEGs in decidua. Upregulated transcripts were prioritized for pathway analysis, revealing significant enrichment in TNF and chemokine signaling pathways (Figure 1). Immune infiltration analysis implicated macrophages, neutrophils and dendric cells as key players, with hub genes (*CCL2, CCL3, EGR1, CYR61, JUNB, FOS, JUN*) exhibiting pronounced upregulation in CSEP, as confirmed by qRT‐PCR (Figure 2).

**Conclusion**: Our study delineates the unique transcriptional landscape of CSEP decidua, uncovering dysregulated inflammatory and immune‐reflect pathways that may drive CSEP pathogenesis and its progression to PAS. These findings provide novel molecular insights and potential therapeutic targets for mitigating adverse outcomes in high‐risk pregnancies.

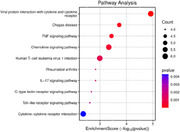

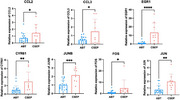

**3:30 PM – 5:00 PM**

## 1263 | **Social Determinants of Health and Birth Outcomes After Implementation of a Systematic Screening Program**

Silvia Carlotta Rosalia^1^; Martina Pavia^1^; Eleonora Cartabia^1^; Laura Pozzer^1^; Nadia Burgassi^2^; Antonella Cromi^1^

^1^University of Insubria, Varese, Lombardia; ^2^Filippo Del Ponte Hospital, Varese, Lombardia

**Objective**: To assess the association between birth and perinatal outcomes and Social Determinants of Health (SDoH), identified through a universal SDoH screening implemented in the context of a quality improvement project.

**Study Design**: This was a case‐control study conducted at a tertiary level maternity hospital within a publicly funded healthcare system. Assessment of 14 SDoH domains, integrated into the Electronic Health Records, was systematically performed at admission for delivery. Cases were defined as patients with a positive SDoH screening for one or more domains. The two women who delivered immediately after each case, with a negative SDoH screening, constituted the control group. Logistic regression was used to estimate the independent association between SDoH and maternal and neonatal outcomes, controlling for pre‐specified demographic and clinical risk factors.

**Results**: Of the total screened patients, 234 (2.9%) had a positive SDoH screening and constituted the case group. The most frequently reported SDoH belonged to the domain of individual‐level social relationships and living conditions (Figure 1). Patients with a positive SDoH screening were more likely to be younger, have a migration history, identify differently than non‐Hispanic White and have comorbidities. Compared with controls, patients who screened positive for SDoH had significantly higher rates of preeclampsia, preterm birth, fetal growth restriction, cesarean delivery, postpartum blood transfusion, newborn admission to the neonatal unit (Figure 2). Multivariable analysis showed that a positive SDoH screening is an independent predictor of preterm birth <34 weeks (OR 5.0, 95% CI 2.3‐11.6) and admission to the neonatal unit (OR 3.0, 95% CI 1.7‐5.4).

**Conclusion**: After implementation of a systematic SDoH screening program in a maternity hospital, we demonstrated an association between a positive screening and adverse birth outcomes. As this occurs in a context ensuring equitable access to maternity care for all women, interventions should target other factors in the complex pathways that contribute to differences in outcomes.

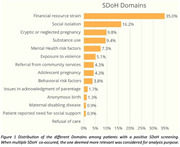

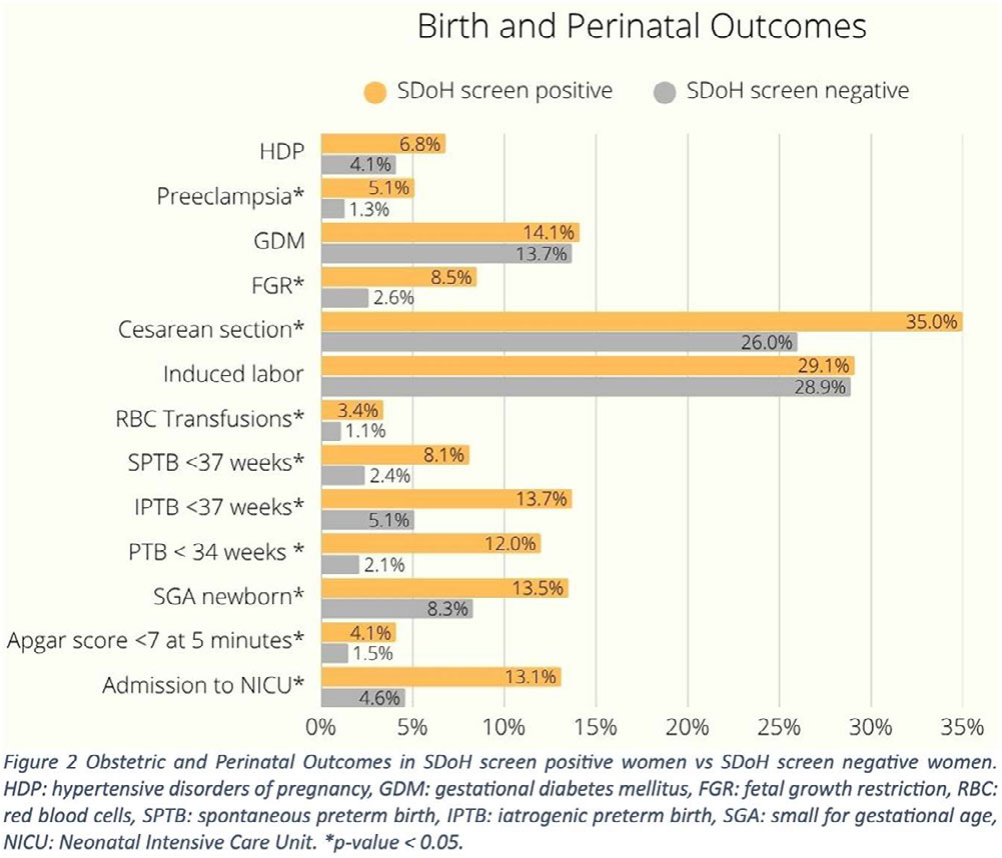

**3:30 PM – 5:00 PM**

## 1264 | **Comparison of Gestational Diabetes Treatment Modalities and Associated Placental Histopathology**

Smrithi Chanjeevaram^1^; Jessica S. Chung^2^; Elizabeth Sapiro^3^; Joy Chung^2^; Lien M. Weiss^2^; Meredith O. Cruz^2^; Brock E. Polnaszek^2^; Anna Palatnik^4^

^1^Medical College of Wisconsin, Wauwatosa, WI; ^2^Medical College of Wisconsin, Milwaukee, WI; ^3^Medical College of Wisconsin, Medical College of Wisconsin, WI; ^4^Medical College of Wisconsin ‐ Milwaukee, WI, Milwaukee, WI

**Objective**: The association between GDM treatment and placental histopathology remains unclear. We aimed to describe placental histopathology in patients with gestational diabetes mellitus (GDM) by diet, oral, or insulin treatment modality.

**Study Design**: We conducted a retrospective cohort study of patients with GDM who delivered at a single academic medical center (2019 to 2023) with available placental histopathology for review. Patients were categorized by treatment modality: diet, oral agents (metformin or glyburide), or insulin. Those with multiple gestation, placenta accreta spectrum, or absence of placental pathology were excluded. Placental histopathology reports were abstracted for findings per College of American Pathologists (CAP) guidelines, including placenta weight, maternal and fetal vascular malperfusion (MVM and FVM), and infection/inflammation. Maternal demographics and pregnancy outcomes were assessed. Chi‐square and t‐tests were used for statistical comparisons.

**Results**: Of 459 patients with GDM and placental histopathology, 49.0% were managed with diet, 5.9% with oral agents, and 45.1% with insulin. Insulin‐controlled patients were more likely to be older, have obesity, chronic hypertension, and earlier GDM diagnosis (all *p* < 0.01). However, no statistically significant differences were observed across treatment groups in placental weight (*p* = 0.22), MVM (*p* = 0.58), FVM (*p* = 0.26), or infection/inflammatory lesions (*p* = 0.34). MVM was common across all groups, with >60% of placentas showing at least one MVM lesion, regardless of treatment modality. Retroplacental hemorrhage was more frequent in the oral group (7.4%, *p* = 0.03) compared to others, but other lesion subtypes were similarly distributed.

**Conclusion**: Placental histopathologic findings did not differ significantly by GDM treatment modality. The high prevalence of MVM across all groups suggests that GDM itself—rather than treatment modality—may be the principal driver of placental changes. These findings reinforce the need for further research into GDM‐mediated placental pathology and its implications for maternal and neonatal outcomes.

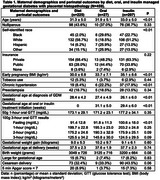

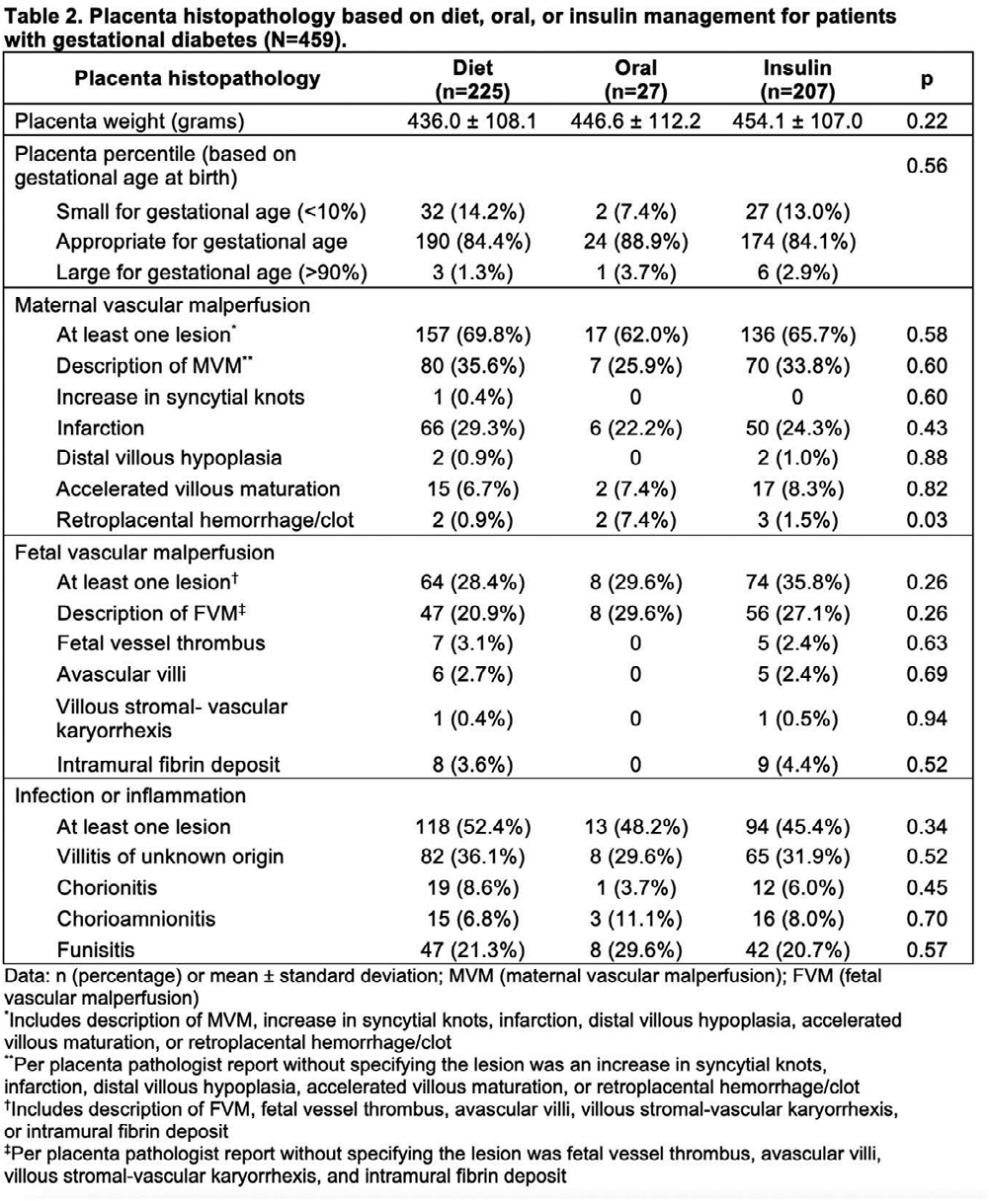

**3:30 PM – 5:00 PM**

## 1265 | **Postpartum Spontaneous Diuresis and Risk of Hospital Return in Preeclampsia With Severe Features**

Sonya Fabricant^1^; Madeline Ponzio^2^; Naomi H. Greene^3^; Natalie A. Bello^4^; Mariam Naqvi^2^

^1^Cedars‐Sinai Medical Center, Los Angeles, CA; ^2^Department of Obstetrics and Gynecology, Cedars‐Sinai Medical Center, Los Angeles, CA; ^3^Department of Obstetrics & Gynecology, Cedars‐Sinai Medical Center, Los Angeles, CA; ^4^Department of Cardiology, Cedars‐Sinai Medical Center, Los Angeles, CA

**Objective**: To evaluate the association between postpartum urine output (UOP) and hospital return within 28 days among individuals with preeclampsia with severe features.

**Study Design**: This was a retrospective cohort study of deliveries at a quaternary care center from 2018‐2023. Eligible individuals were identified based on diagnosis of a hypertensive disorder of pregnancy plus receipt of postpartum magnesium sulfate. Exclusion criteria were pre‐existing renal disease, acute renal failure, pulmonary edema, heart failure, and receipt of diuretics during delivery admission. The primary outcome was hospital return (emergency department, triage or readmission) within 28 days of discharge. The primary exposure was UOP in the first 24 hours postpartum, analyzed in quartiles based on the sample distribution: Q1 (≤ 135.4 mL/hr), Q2 (135.4–209.2 mL/hr), Q3 (209.2–284.2 mL/hr), and Q4 (  > 284.2 mL/hr). Bivariate analyses was used to compare fluid intake and output by hospital return status. Multivariable logistic regression was performed to estimate the association between UOP quartile and hospital return, adjusting for covariates. Sensitivity analysis used weight‐adjusted UOP (mL/kg/hr), also in quartiles.

**Results**: Of 1819 eligible individuals, 175 (10.6%) returned to the hospital within 28 days. Those with a hospital return had lower total UOP in the first 24 hours postpartum (4.8 vs 5.3 L, *p* = 0.01). Total intravenous fluid intake did not differ significantly between groups. Hospital return rates varied significantly by UOP quartile, with lower rates observed in the highest quartile (*p* = 0.04; Table 1). After adjusting for covariates, patients in the highest UOP quartile had 57% lower adjusted odds of hospital return compared to those in the lowest quartile (aOR 0.43, 95% CI 0.26–0.72, *p* = 0.001; Table 2). Using weight‐adjusted UOP (mL/kg/hr) yielded consistent findings, with an aOR of 0.43 (95% CI 0.26–0.71; *p* = 0.001) for the highest vs lowest quartile.

**Conclusion**: Among individuals with preeclampsia with severe features, higher UOP in the first 24 hours postpartum was associated with lower odds of hospital return.

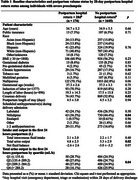

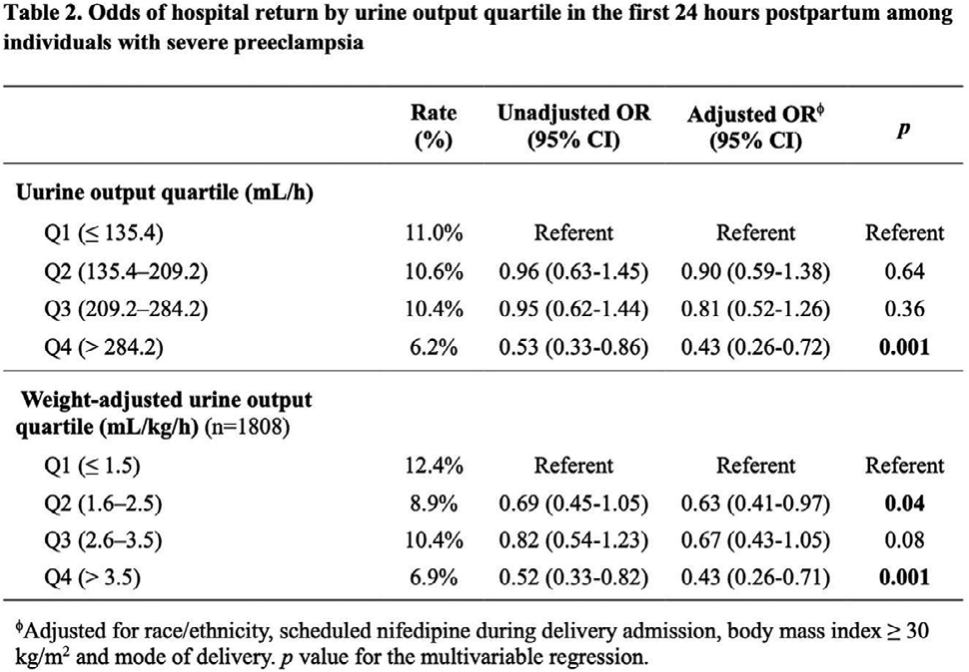

**3:30 PM – 5:00 PM**

## 1266 | **Perinatal Outcomes Associated With Bupropion Use in Pregnant Persons With Methamphetamine Use Disorder**

Sophia Izhar^1^; Michelle R. Petrich^2^; Justine Keller^2^; Niraj R. Chavan^3^

^1^Saint Louis University/School of Medicine, Saint Louis University School/St. Louis, MO; ^2^Saint Louis University/SSM Health, Saint Louis University/St. Louis, MO; ^3^Saint Louis University, Saint Louis, MO

**Objective**: We sought to evaluate perinatal outcomes associated with bupropion use in pregnant persons with Methamphetamine Use Disorder treated at a comprehensive perinatal substance use disorder (SUD) program.

**Study Design**: We conducted a retrospective cohort study of mother‐infant dyads who received prenatal care at our specialized perinatal SUD center over a 10‐year span from 01/2024 to 03/2023. Demographic characteristics, substance use patterns, psychiatric history, and maternal and neonatal outcomes were abstracted from electronic records and compared across 2 groups—pregnancies with antepartum bupropion exposure and those without. Bupropion exposure included prescribing in the context of established and off‐label indications, including perinatal mood disorders as well as tobacco and methamphetamine use disorder. Chi‐squared or Fisher's exact tests were used to compare categorical variables, as appropriate. Statistical significance was set at p≤.05.

**Results**: Of 1047 pregnancies reviewed, 121 (12%) were complicated by DSM‐V defined Methamphetamine Use Disorder. Of this subset, 19 (16%) were exposed to bupropion. The most frequent dose prescribed was 150mg (63%) and initiation peaked in the second trimester (53%). Although 68% of indications involved mood disorders, in 42% the benefit of improving methamphetamine cravings was cited. In exposed patients, 3 (16%) had an estimated date of delivery (EDD) prior to 2021, and 16 (84%) had an EDD after 2021. Maternal characteristics were similar regarding severity of amphetamine use disorder, substance use disorder history, and psychiatric history (Table 1). Patients with bupropion exposure had similar rates of maternal relapse, overdose, tobacco use, and infant drug screen results (Table 2).

**Conclusion**: The increase in bupropion exposure in this cohort since 2021 mirrors practice changes pertaining to use of bupropion for treatment of methamphetamine use disorder in the non‐pregnant literature. Further research in a larger cohort is warranted to fully examine the therapeutic potential of this intervention in the context of perinatal methamphetamine use disorder.

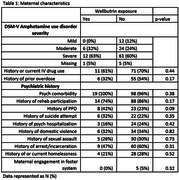

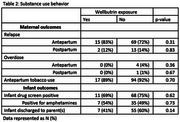

**3:30 PM – 5:00 PM**

## 1267 | **Phenotyping Glucose, Physical Activity, and Sleep Patterns Using Wearables in Gestational Diabetes**

Souptik Barua^1^; Jose Zavala^2^; Carina Flaherty^1^; Samika Hariharan^1^; Brianna Bhoopsingh^1^; Delphina Maldonado^2^; Ifeoma Ogamba‐Alphonso^2^; Anne‐Marie Wise^2^; Hye J. Heo^2^

^1^NYU Grossman School of Medicine, New York, NY; ^2^NYU Grossman Long Island School of Medicine, Mineola, NY

**Objective**: Limited data exists on the impact of physical activity and sleep on dysglycemia in pregnancies with gestational diabetes mellitus (GDM).  We evaluated the feasibility of using continuous glucose monitors (CGM) and accelerometers to phenotype participants with and without GDM during pregnancy and early postpartum.

**Study Design**: We conducted a prospective cohort study of pregnant (26‐32 wks) and postpartum (4‐12 wks) patients with and without GDM who wore an accelerometer and a CGM for 14 days. Metrics included days of device usage and user acceptability via the 100‐point System Usability Scale (SUS) survey. Kruskal‐Wallis and post‐hoc Dunn's tests were used to identify differences amongst the four groups.

**Results**: 24 participants enrolled in the study (11 pregnant GDM, 3 postpartum GDM, 5 pregnant without GDM, and 5 postpartum without GDM) (Table 1). CGM and accelerometry usage during day and sleep hours was assessed (Table 1). On the SUS survey, participants scored the accelerometer 68±27 points and CGM 70±27 points out of 100, with 91% of participants having a net positive opinion (SUS >50) of both devices (Figure 1).

There were significant differences in steps, sleep duration, and time above 140 mg/dL (all *p* < 0.05; Table 1) between groups. Postpartum controls had more steps (11624 [11252,12235] vs 7910 [7239,9259]; *p* = 0.03) and lower sleep duration (4.7 [4.6,5.1] hrs vs 7.2 [6.5,7.6] hrs; *p* = 0.01) than pregnant GDM participants. Postpartum GDM group had a higher time above 140 mg/dL than controls (3.4 [2.4,9.9]% vs 0 [0,0]%; *p* = 0.03), indicating possible unresolved dysglycemia. 86% of participants walked over 7000 steps daily, while only 41% achieved recommended 7 hours of sleep. CGM data revealed all participants spent more than the recommended 70% of time in euglycemia.

**Conclusion**: Simultaneous use of accelerometry and CGM was acceptable for participants with or without GDM during pregnancy and early postpartum. These devices can enhance understanding of exercise, sleep, and glycemia relationships, aiding management of individuals at highest risk of adverse outcomes.

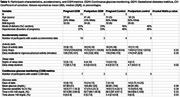

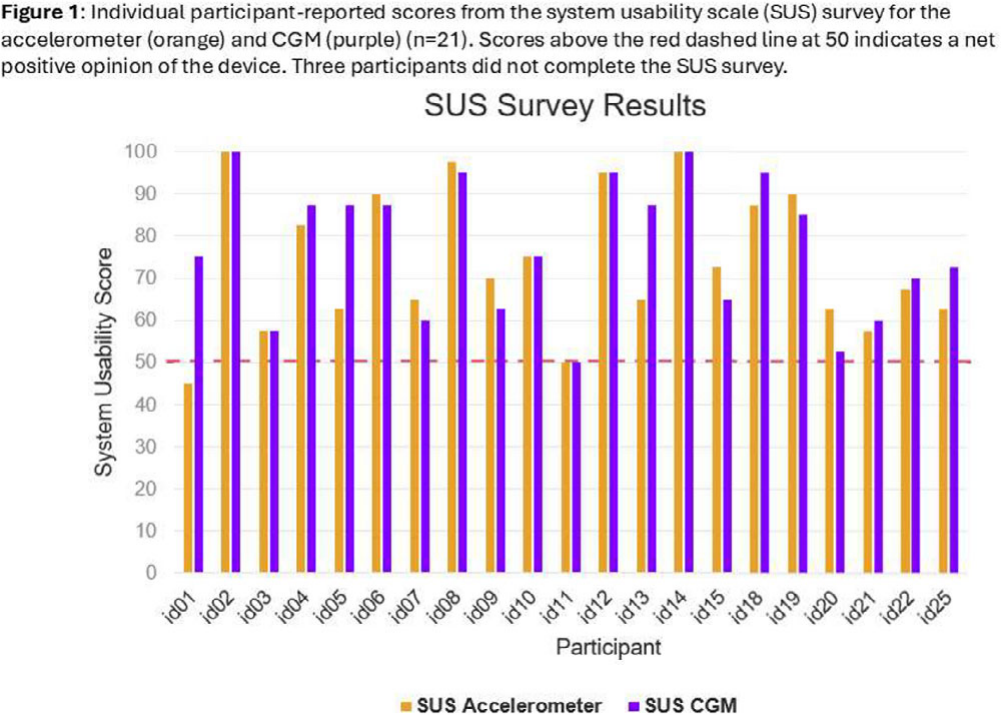

**3:30 PM – 5:00 PM**

## 1268 | **Adverse Childhood Experiences Are Associated With Maternal Morbidity and Social Vulnerability**

Spencer C. Darveau^1^; Sarah J. Weingarten^2^; Aarushi Bhan^3^; Maya Jordan^4^; Layla Kaiden^1^; Corrina M. Oxford‐Horrey^1^

^1^NewYork Presbyterian‐Weill Cornell Ob/Gyn, New York, NY; ^2^Division of Maternal and Fetal Medicine, Department of Obstetrics and Gynecology, New‐York Presbyterian Hospital‐Weill Cornell, Department of Obstetrics and Gynecology, New‐York Presbyterian Hospital‐Weill Cornell, NY; ^3^Case Western Medical School, Cleveland, OH; ^4^Touro College of Osteopathic Medicine, New York, NY

**Objective**: Adverse childhood experiences (ACEs), including abuse, neglect, and household dysfunction, have been linked to adverse pregnancy outcomes. We aimed to evaluate whether high ACE scores were associated with increased risk of adverse obstetric and neonatal outcomes and psychosocial vulnerability in an urban obstetric cohort.

**Study Design**: This prospective cohort study enrolled pregnant patients ≥18 years old who delivered at ≥20 weeks gestation from 2023–2024. Participants completed the 10‐item ACE Questionnaire. Demographics, delivery characteristics, and clinical outcomes were extracted from the electronic medical record. Clinical outcomes were based on the Center for Disease Control's indicators of Severe Maternal Morbidity (SMM), including ICU admission, blood product transfusion, vasoactive medication use, and obstetric complications. ACE scores were categorized as low (0–3) or high (≥4), and descriptive and comparative analyses were performed using Fisher's exact and Mann‐Whitney U tests.

**Results**: Among 133 patients, 24 (18%) had high ACE scores. Demographics are shown in Table 1. Compared to those with low ACE scores, these patients were more likely to identify as non‐white (*p* = 0.012), Hispanic or Latino (*p* < 0.001), have lower income (*p* = 0.003), be unmarried (*p* = 0.003), have public insurance (*p* < 0.001), and carry a psychiatric diagnosis (*p* = 0.044). Maternal and neonatal outcomes are shown in Table 2. Rate of postpartum ICU‐ admission was significantly higher in the high ACE group (12.5% vs 0.9%, *p* = 0.019). Newborn complications and NICU admission rates did not significantly differ between groups.

**Conclusion**: In this cohort, higher ACE scores were associated with increased risk of ICU admission after delivery and multiple social and psychiatric risk factors. These findings support further exploration of ACE‐informed perinatal screening and tailored care strategies to address cumulative stress exposure and potentially improve outcomes.

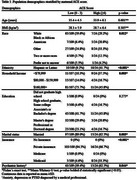

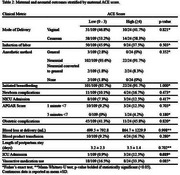

**3:30 PM – 5:00 PM**

## 1269 | **Postpartum Management and Transition‐of‐Care for Patients With Type 2 Diabetes**

Alexandra Schroeder^1^; Spencer R. Pope^2^; Dorothea Mostello^2^

^1^Saint Louis University Division of Maternal Fetal Medicine, Saint Louis University Department of Maternal Fetal Medicine, MO; ^2^Saint Louis University, Saint Louis Univeristy/Saint Louis, MO

**Objective**: We performed a multiphase quality improvement project that assessed the need for access to post‐pregnancy care for patients with Type 2 Diabetes (T2DM) and implemented a protocol to facilitate scheduled postpartum diabetes follow‐up. We also aimed to standardize postpartum hospital management of T2DM to support this transition of care.

**Study Design**: In phase 1, we performed an anonymous survey of patients with T2DM on their experiences and perceptions regarding access to diabetes care outside of pregnancy. In phase 2, we established postpartum follow up for diabetes care antepartum by ascertaining whether the patient had a primary care provider (PCP) or endocrinologist, providing referrals as needed, and using a Smartphrase to document the status of the process. We also implemented a hospital‐based protocol to standardize postpartum inpatient care for patients with T2DM by assessing need for intravenous insulin in the immediate postpartum period, monitoring glycemic control after delivery, and establishing a postpartum medication regimen.

**Results**: Our survey of 32 patients found that over 90% of our patients understood the importance of managing diabetes outside of pregnancy, yet fewer than 60% followed with a PCP regularly prior to pregnancy. Approximately 25% did not have a PCP and identified cost as a serious barrier to their healthcare. From our Phase 2 intervention, we observed an improvement in documented PCP/Endocrinologist follow‐up or referral from pre‐intervention (*N* = 28) of 53.6% to post‐intervention (*N* = 24) of 91.7% (*p* < 0.01). Additionally, we found improvement in both insulin use in the first 24 hours (21.4% vs 73.9%, *p* < 0.01) and prevention of metformin use in the first 24 hours (45.5% vs 8.7%, *p* < 0.05) postpartum.

**Conclusion**: Our study established the need for improved postpartum follow‐up of T2DM care and significantly improved our rates of scheduled follow‐up or referral. Additionally, we implemented an inpatient postpartum protocol for management of T2DM, which improved medication dosing and timing.

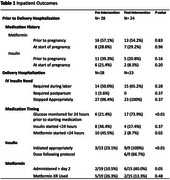

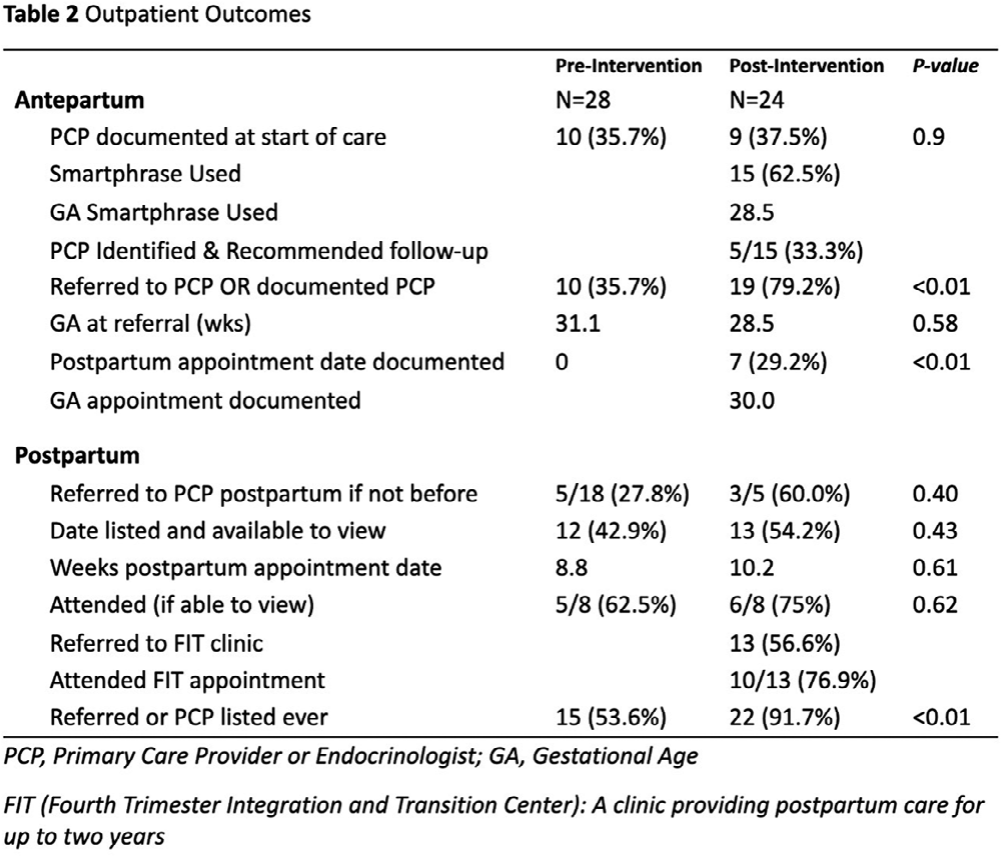

**3:30 PM – 5:00 PM**

## 1270 | **Umbilical Cord Blood Gases as Indicators of Fetal Compromise in Preterm Preeclampsia With Growth‐Restricted Fetuses**

Stephanie Schreiber‐Gonzalez^1^; Renata Mukai^2^; Calla Holmgren^3^; Sydney Whalen^4^; Karlie Snead^5^; Alexandra Wilson^6^; Rachel K. Harrison^7^

^1^Ascension Healthcare, Clarendon Hills, IL; ^2^University of Illinois at Chicago, Chicago, IL; ^3^Advocate Medical Group, Chicago, IL; ^4^University of Illinois at Chicago, University of Illinois at Chicago, IL; ^5^Ascension Healthcare, Ascension, IL; ^6^Advocate Health Care, Advocate, IL; ^7^Department of Maternal‐Fetal Medicine, Advocate Aurora Health, Chicago, IL

**Objective**: To assess severity of umbilical artery Doppler (UAD) abnormalities and correlation to umbilical cord blood gases in pregnancies complicated by fetal growth restriction (FGR) and preeclampsia <34 weeks.

**Study Design**: Retrospective cohort study of 199 patients complicated by FGR and preterm preeclampsia <34 weeks admitted within a large hospital system between 12/2020 and 12/2023. Pregnancies complicated by lethal fetal anomalies were excluded. The pregnancies were stratified by UAD findings: normal, elevated resistance, and absent/reversed end‐diastolic flow (AEDF/REDF). Those with normal UAD were used as the comparison group. The primary outcomes assessed were arterial cord gas components including the arterial pH, pCO_2_, pO_2_< 25, and base excess. Statistical analysis was performed using Stata version 17.0 via ANOVA, chi2 or fisher's exact, and linear and logistic regression to control for confounders.

**Results**: 199 pregnancies met inclusion criteria. UAD were normal in 51.2% (*n* = 102), elevated in 28.6% (*n* = 57), and AEDF/REDF in 20.1% (*n* = 40). Increasing UAD severity was associated with earlier gestational age (GA) at FGR diagnosis, estimated fetal weight (EFW) <3rd percentile (*p* = 0.032), and earlier delivery (*p* = 0.008). Other baseline characteristics were similar across groups  . With regard to the primary outcome, arterial cord pH was not associated with UAD severity on univariate analysis (normal 7.26±0.07, elevated 7.25±0.09, AEDF/REDF UAD 7.21±0.07) (*p* = 0.064). In regression analysis AEDF/REDF was associated with a lower pH [OR ‐0.05, 95% CI(‐0.09 to ‐0.01)], higher pCO_2_ [5.99, 95% CI(0.07 to 11.92)], and increased likelihood of pO_2_ <25 [2.07, 95% CI(1.96 to 6.68)]. When controlling for confounders including GA at FGR diagnosis, GA at delivery, EFW <3rd percentile, and steroid exposure, lower arterial pH remained associated with AEDF/REDF [aOR ‐0.05, 95% CI(‐0.09 to ‐0.01)](see Table 1).

**Conclusion**: Severe UAD abnormalities in pregnancies complicated by preterm preeclampsia and FGR are linked to worse umbilical cord gases, with AEDF/REDF UAD demonstrating worse arterial pH.

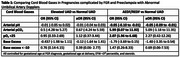

**3:30 PM – 5:00 PM**

## 1271 | **Association Between Maternal Vascular Malperfusion and Blood Pressure 2‐7 Years Postpartum**

Sunitha Suresh^1^; Alexa A. Freedman^2^; Beth Plunkett^3^; Linda M. Ernst^4^

^1^Endeavor Health, Endeavor Health System, IL; ^2^Northwestern University, Chicago, IL; ^3^Endeavor Health Evanston, Evanston, IL; ^4^Endeavor Health, Evanston, IL

**Objective**: Adverse pregnancy outcomes (APO) are associated with increased cardiovascular disease (CVD). Placental pathology can reveal abnormalities that may more specifically reflect CVD risk. The purpose of this analysis was to use a large prospective multicenter study to examine the association of placental maternal vascular malperfusion (MVM) and later‐life blood pressure (BP).

**Study Design**: This is a secondary analysis of the numom2b Heart Health Study which entailed an in‐person study visit 2‐7 years after index pregnancy. Participants who had a placental pathology report in index pregnancy were included. Those with preexisting chronic hypertension in the parent study were excluded. Placental pathology reports were categorized into presence or absence of MVM by a trained perinatal pathologist based on documented lesions in the abstracted report. Mean systolic and diastolic BP (SBP, DBP) at 2‐7 year visit were compared by presence/absence of MVM. Linear models, adjusted for insurance, age, BMI, and race/ethnicity were utilized to evaluate the association between MVM and BP. Secondary analysis was stratified by APO, defined as hypertensive disorder of pregnancy, stillbirth, SGA birth, preterm birth.

**Results**: 629 patients met inclusion criteria, of which 34.9% had MVM. The follow up visit was on average 3.0 years (IQR: 2.5, 3.6) after index pregnancy. Participants with MVM were more likely to be married and less likely to report smoking. There were no differences in age, race/ethnicity, or insurance status. In adjusted models, MVM was associated with higher SBP and DBP (SBP B 2.2 mmHg (0.3, 4.0); DBP B 2.6 mmHg (1.0, 4.2), Table). In models stratified by APO, MVM was associated with higher SBP among those without an APO (B 3.7 mmHg (1.0, 6.4)), but not among those with an APO (B 0.9 mmHg (‐1.6, 3.4) Figure).

**Conclusion**: MVM on placental pathology was associated with higher BP 2‐7 years postpartum.  In stratified analyses, the association with SBP remained among those without an APO.   MVM may identify those at higher risk for CVD regardless of APO and should be included in future risk stratification.

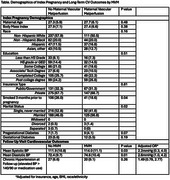

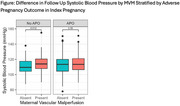

**3:30 PM – 5:00 PM**

## 1272 | **Incidence and Preventability of Severe Early Pregnancy Morbidity at a Tertiary Referral Center**

Susan E. Nourse; Alexandra Gero; Taryn T. Hunt‐Smith; David Turok; Lori M. Gawron; Rebecca Simmons; Michelle P. Debbink; Jessica N. Sanders

University of Utah, Salt Lake City, UT

**Objective**: To identify incidence of severe early pregnancy morbidity (SEPM) by adapting severe maternal morbidity (SMM) definitions to pregnancies <24 weeks; secondarily, to assess preventability of these events using a validated tool

**Study Design**: This is a retrospective cohort study of pregnant patients at a single tertiary care institution between 1/1/2017 to 12/31/2023. Individuals with a documented pregnancy episode or CPT code denoting an abortive outcome of pregnancy were included. We identified hospital encounters at <24 0/7 weeks’ gestation. Using ICD‐10 diagnosis codes, blood product administration records, and intensive care transfer records, we identified encounters with possible SEPM using the following three methods: CDC SMM diagnosis codes, ACOG/SMFM criteria (transfusion of >4 units of blood products or intensive care unit (ICU) transfer), or abortion‐related morbidity, which is defined as pelvic infection, damage to pelvic organs, or hemorrhage with estimated blood loss >500 cc or requiring urgent intervention. We reviewed the electronic health record of all screen‐positive cases to verify the presence of an SEPM event, assess preventability using the Alliance for Innovation on Maternal Health SMM review form, and screen for influence of abortion restrictions.

**Results**: Of 46,181 pregnant patients, we identified 407 (0.88% of all pregnancies) SEPM events, with 233, 87, and 253 events meeting CDC, ACOG, and abortion‐related morbidity criteria, respectively. Median gestational age at SEPM was 14 weeks [IQR 9]. Common SEPM events were hemorrhage (221; 0.47%), blood transfusion (126; 0.27%), ICU transfer (67; 0.15%) and sepsis (58; 0.13%). Common underlying etiologies were early pregnancy loss (130/407, 32%), non‐obstetric infection (53/407, 13%), premature rupture of membranes or obstetric infection (42/407, 10%), and pre‐existing conditions (42/407, 10%). SEPM was preventable in 126/407 (31%) of events, and 54/126 (43%) of preventability was related to restrictive abortion laws.

**Conclusion**: SEPM contributes to preventable morbidity and may be affected by abortion restrictions.

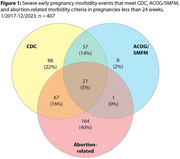

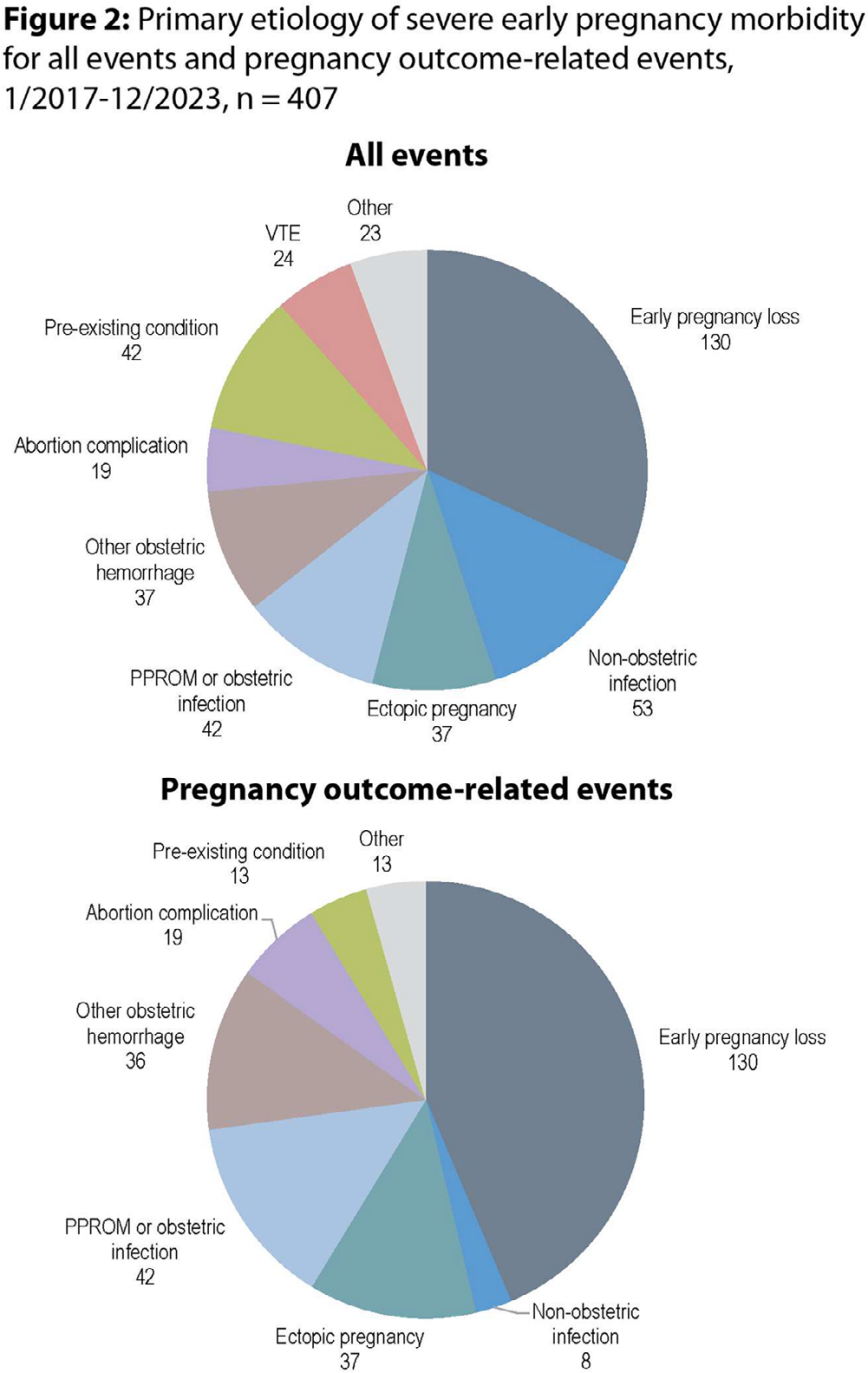

**3:30 PM – 5:00 PM**

## 1273 | **Preconception Glucagon‐Like Peptide‐1 Receptor Agonist Therapy for Type 2 Diabetes Mellitus: A Cost‐Effectiveness Analysis**

Sydney Bethers^1^; Stacia C. Hickey^2^; Amelia H. Gagliuso^2^; Jonathan Purnell^3^; Aaron B. Caughey^3^

^1^Oregon Health & Science University, Portland, OR; ^2^Oregon Health and Science University, Portland, OR; ^3^Oregon Health & Science University, Oregon Health & Science University, OR

**Objective**: As the rate of type 2 diabetes mellitus (T2DM) among women of reproductive age continues to rise, interest has grown in using preconception glucagon‐like peptide‐1 receptor agonist (GLP1 RA) therapy to reduce obstetrical morbidity and mortality associated with T2DM. This study aimed to evaluate if preconception GLP1 RA therapy is a cost‐effective intervention to improve perinatal outcomes in those with pregnancies complicated by T2DM.

**Study Design**: A TreeAge decision‐analytic model was constructed to compare outcomes and effectiveness of one year of GLP‐1 RA therapy before conception in a cohort of 43,152 people with T2DM. Our cohort was estimated from the annual number of live births affected by T2DM in the U.S. Outcomes included preeclampsia, preterm birth, neonatal intensive care unit (NICU) admission, neonatal death, and neurodevelopmental delay. The incremental cost‐effectiveness ratio (ICER) was set to $100,000 per quality‐adjusted life years (QALYs) to compare strategies, accounting for maternal and neonatal utilities.  All model inputs were derived from the literature, and sensitivity analyses were conducted to test the robustness of the results.

**Results**: GLP‐1 RA therapy for one year prior to conception resulted in fewer cases of preeclampsia, preterm birth, NICU admissions, neonatal death, and neurodevelopmental delay compared to no GLP‐1 RA therapy (Table 1).  Although preconception GLP‐1 therapy is more costly, it yields additional QALYs, making it a cost‐effective approach with an ICER of $44,578 per QALY. One‐way sensitivity analysis showed that preconception GLP‐1 RA therapy remained cost‐effective at drug prices up to $6,058 annually.

**Conclusion**: This study demonstrates that one year of preconception GLP‐1 RA therapy is a cost‐effective strategy that improves perinatal outcomes in individuals with preexisting T2DM. These findings support the incorporation of GLP‐1 RA therapy into pre‐pregnancy diabetes management to reduce the risk of pregnancy‐related complications in this high‐risk population.

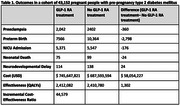

**3:30 PM – 5:00 PM**

## 1274 | **Implementation of Restricted Labor IV Fluid Protocol and Development of Pregnancy Associated Hypertension**

Talia Suner^1^; Turner Simmers^2^; Brian W. James^1^; James D. Toppin^1^; Joseph R. Biggio, Jr.^3^; Frank B. Williams^4^; Barbara Neuhoff^1^

^1^Ochsner Clinic Foundation, New Orleans, LA; ^2^Ochsner Clinic Foundation, New orleans, LA; ^3^Ochsner Health, New Orleans, LA; ^4^Ochsner Clinic Foundation, Ochsner Clinic Foundation, LA

**Objective**: Routine labor management includes bolus and maintenance IV fluids to maintain maternal euvolemia and uterine perfusion. To adapt to the recent national IV fluid shortage, our hospital implemented a restricted labor IV fluid protocol. We hypothesize that decreased IV fluid volume administration during labor will lead to a lower incidence of pregnancy associated hypertension (PAH) diagnosed intrapartum or postpartum.

**Study Design**: We performed a retrospective cohort study of patients during their delivery admission at a single tertiary hospital from May 1, 2024, to May 1, 2025. Included were nulliparous, term patients with a vertex presentation admitted for labor management. Patients with PAH at the time of admission were excluded. We compared patients pre‐ and post‐implementation, which started October 1, 2024. The primary outcome was intrapartum or postpartum PAH, defined as gestational hypertension or preeclampsia. Secondary outcomes included postpartum readmission for hypertension, cesarean delivery, 5‐minute Apgar scores, and neonatal intensive care (NICU). Baseline characteristics and outcomes were compared via chi square or Mann‐Whitney U test where appropriate. Relative risks (RR) were generated with 95% confidence intervals (CI). Alpha was set at 0.05.

**Results**: Among 996 nulliparous patients with singleton term pregnancies, 558 (56%) delivered after October 1, 2024. Blood loss was decreased post‐implementation, but this difference was not clinically significant. No other differences in baseline characteristics were observed between groups (table 1). Diagnosis of PAH intrapartum or postpartum did not change following implementation of the restricted IV fluid protocol (RR 0.97, 95% CI 0.79‐1.19, table 2). No differences were observed in readmission for hypertension (RR 1.04, 95% CI 0.23‐4.65), cesarean delivery (RR 0.99, 95% CI 0.80‐1.24), NICU admission (RR 1.07, 95% CI 0.64‐1.79), or 5‐minute Apgar scores (9 vs 9, *p* = 0.65).

**Conclusion**: Implementation of a restricted IV fluid labor protocol was not associated with decreased risk for intrapartum or postpartum PAH in nulliparous term pregnancies.

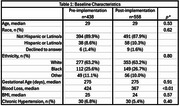

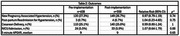

**3:30 PM – 5:00 PM**

## 1275 | **First‐Trimester Fasting Glucose Correlates With Long‐Term Maternal Diabetes Mellitus and Cardiovascular Complications**

Tamar Katzir^1^; Tal Schiller^2^; Maya Oberman^3^; Alon Ben‐Arie^4^; Edi Vaisbuch^3^; Oren Barak^5^

^1^kaplan medical center, Tal shahar, HaMerkaz; ^2^Edith Wolfson Medical Center, Holon, HaMerkaz; ^3^Kaplan Medical Center, Kaplan Medical Center, HaMerkaz; ^4^kaplan medical center, Rehovot, HaMerkaz; ^5^Kaplan Medical Center, Rehovot, HaMerkaz

**Objective**: To examine the association between first‐trimester fasting glucose (FG) and subsequent maternal diabetes mellitus (DM) and cardiovascular disease (CVD).

**Study Design**: We performed a retrospective review of electronic medical records from a large healthcare provider, identifying deliveries since 2012. Pregnancies were included if first‐trimester FG was documented, excluding those with preexisting DM or CVD. Only the earliest delivery per individual was included. Propensity score‐weighted Kaplan‐Meier analysis was used to estimate cumulative incidence of maternal long‐term outcomes by FG category, with censoring when follow‐up data were no longer available. A subanalysis was conducted among those with more than one elevated first trimester FG (“persistent”). The primary outcome, DM and/or CVD diagnoses, (ICD‐10 codes) within five years postpartum, was compared according to the calculated 95th percentile (2SD) FG cutoff.

**Results**: Derived from the 202,960 people who met the inclusion criteria, a first‐trimester FG level of 100 mg/dL was identified as the 95th percentile, with 10,759 people having a first‐trimester FG≥100mg/dL, and 192,201 having an FG below this cutoff. Those with an FG≥100 mg/dL were older and had a higher BMI (31.0±5.7 vs. 29.8±5.5, *p* < 0.001 and 28.2±6.7 vs. 25.05±14.9 kg/m^2^ *p* < 0.0001, respectively). At five years postpartum (*n* = 159,148), compared to those with a FG< 95^th^ percentile, they had higher rates of HbA1C >5.7% [16.9% (*n* = 1,509) vs. 3.3% (*n* = 6,552), *p* < 0.001 and newly diagnosed DM and CVD [2.4% (*n* = 203) vs. 0.6% (*n* = 984), *p* < 0.001 and 0.9% (*n* = 78) vs. 0.6% (*n* = 913), *p* < 0.001, respectively). These findings are further supported by the subanalysis, which showed a significantly higher risk of newly diagnosed DM in the persistent elevated‐FG group (Figure 1).

**Conclusion**: A first‐trimester fasting glucose level ≥100mg/dL is associated with an increased risk of developing diabetes mellitus and cardiovascular disease later in life. In these patients, postpartum care should focus on monitoring and patient education aiming to mitigate these risks.

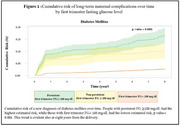

**3:30 PM – 5:00 PM**

## 1276 | **The Paradox of Prenatal War Exposure: A Protective Effect Against Childhood Infections?**

Tamar Wainstock^1^; Polina Schwarzman^2^; Eyal Sheiner^3^

^1^Department of Epidemiology, Biostatistics and Community Health Sciences, Faculty of Health Sciences, Ben‐Gurion University of the Negev, Beer Sheva, HaDarom; ^2^Soroka University Medical Center, Soroka University Medical Center, HaDarom; ^3^Soroka University Medical Center, Faculty of Health Sciences, Ben‐Gurion University of the Negev, beer sheva, HaDarom

**Objective**: Armed conflicts represent an acute and severe source of maternal stress, yet their long‐term effects on offspring immune outcomes remain underexplored. This study aimed to evaluate whether in utero exposure to maternal stress induced by military conflict influences the incidence of infectious morbidity during childhood and adolescence.

**Study Design**: A retrospective population‐based cohort study was conducted, including all singleton live births at a large tertiary medical center between 2008 and 2021. During this period, two distinct military conflicts occurred in the region, each lasting up to three weeks. Offspring were categorized as “exposed” if gestation overlapped with either conflict, and “unexposed” if not. The primary outcome was the incidence of the first diagnosis of infectious morbidity documented in either hospital or community‐based records, up to age 18 years. Multivariable Cox proportional hazards models were used to estimate the association between prenatal conflict exposure and offspring infectious diseases, adjusting for clinically relevant variables.

**Results**: Of the 130656 offspring in the study, 22481 (17.2%) were exposed in‐utero to war, and 8922 (6.8%) were exposed specifically during first trimester  . Offspring exposed in utero to war had a lower risk of infectious morbidities (252.91/ 1000 person years, versus 309.45/1000 person years, *p* = 0.013, Graph). The risk for infectious diseases was lower, regardless of the trimester of exposure, preterm delivery,  gestational diabetes, maternal age, caesarean delivery and ethnicity (aHR = 0.97; 0.95‐0.99), using a Cox proportional hazards model.

**Conclusion**: Prenatal exposure to maternal stress related to brief military conflicts, is associated with a reduced risk of childhood infectious morbidity. These unexpected findings suggest a potential adaptive or compensatory immune mechanism in the exposed offspring. Further research is warranted to explore the underlying biological pathways and to determine whether the nature, timing, or duration of stress exposure plays a role in shaping immune resilience.

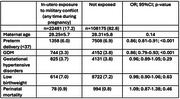

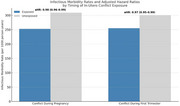

**3:30 PM – 5:00 PM**

## 1277 | **Risk Factors for Preventable Severe Early Pregnancy Morbidity at a Tertiary Academic Center**

Taryn T. Hunt‐Smith; Susan E. Nourse; Alexandra Gero; David Turok; Jessica N. Sanders; Michelle P. Debbink

University of Utah, Salt Lake City, UT

**Objective**: To characterize risk factors for preventable severe early pregnancy morbidity (SEPM) events in pregnancies <24 weeks.

**Study Design**: This was a retrospective cohort study evaluating SEPM <24 weeks from 1/2017 to 12/2023 at a tertiary academic center. We defined SEPM by one or more of the following criteria: (1) CDC‐defined severe maternal morbidity (SMM) diagnosis codes; (2) transfusion of >4 units of blood or ICU‐admission; or (3) or abortion‐related morbidity, including pelvic infection, damage to pelvic organs, or hemorrhage. We included patients aged 12‐55 years with hospital encounters occurring at <24 0/7 weeks or ≤ 42 days following the end of a pregnancy ending <24 weeks. We identified possible SEPM events using ICD‐10 codes, blood product administration records, and intensive care transfer records, and confirmed presence of true SEPM events by medical record review. We assessed preventability using the Alliance for Innovation of Maternal Health SMM review form. We conducted univariate and multivariate logistic regression analysis to estimate independent risk factors for development of a preventable SEPM compared to non‐preventable SEPM. Covariates included age, obesity, race, ethnicity, any maternal comorbidity, insurance status, area deprivation index, and maternal transport from outside facility. We calculated unadjusted (OR) and adjusted odds ratios (aOR) and 95% confidence intervals (CIs).

**Results**: Of 46,181 unique pregnancies, 407 SEPM events were identified (0.88% of all pregnancies). SEPM events were deemed preventable in 126/407 (31%) of events. In a multivariable model, age 24–35 (aOR 2.53, 95% CI 1.01–6.35) and > 35 (aOR 3.30, 95% CI 1.24–8.77) and maternal transport (aOR 2.67, 95% CI 1.50–4.76) were associated with higher odds of preventability. Obesity was associated with lower odds of preventability (aOR 0.54, 95% CI 0.31–0.95) (Table 1).

**Conclusion**: Older age and maternal transport were associated with higher odds of preventability, while BMI was associated with lower odds. Identifying risk factors for preventability may help target interventions to reduce preventable SEPM.

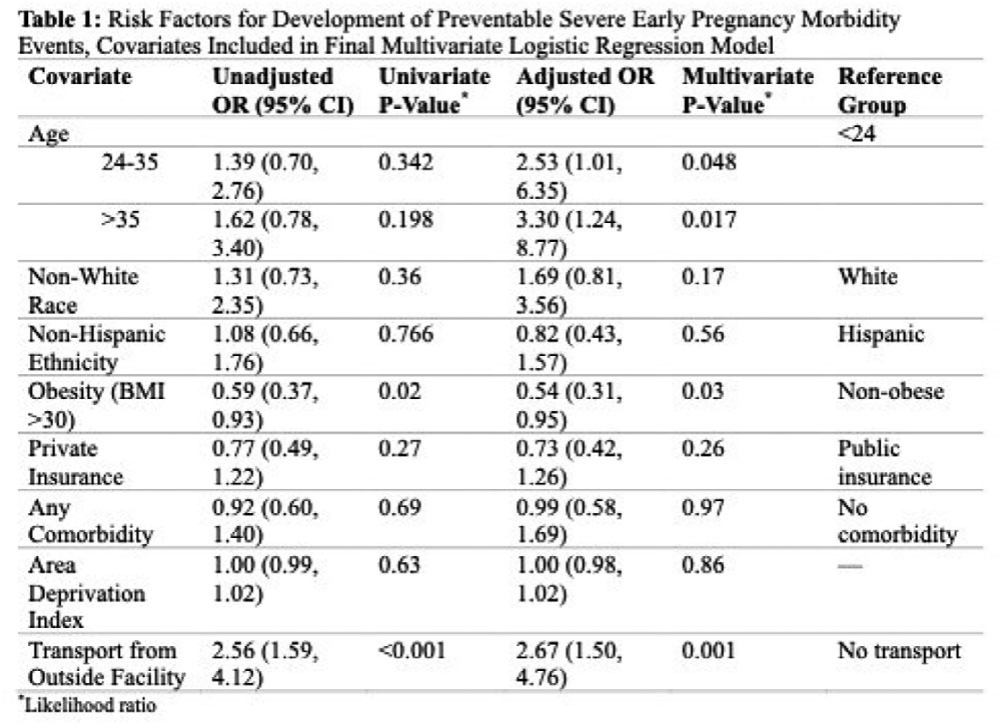

**3:30 PM – 5:00 PM**

## 1278 | **Azithromycin for Labored Cesarean Complicated by IAI Is Associated With Reduced Postpartum Infection and Readmission**

Taylor S. Freret^1^; Ethan Litman^2^; Mark A. Clapp^3^; Sarah E. Little^4^

^1^Beth Israel Deaconess Medical Center, Brookline, MA; ^2^Beth Israel Deaconess Medical Center, Boston, MA; ^3^Department of Obstetrics and Gynecology, Mass General Brigham, Massachusetts General Hospital, MA; ^4^Beth Israel Deaconess Medical Center, Newton, MA

**Objective**: The C/SOAP trial (late 2016) showed that perioperative azithromycin for cesarean birth after labor reduces postpartum infection; patients with IAI were excluded. We sought to analyze if azithromycin use in this excluded population is associated with reductions in infectious outcomes.

**Study Design**: This was a retrospective cohort study (2013‐2025) using the Epic Cosmos platform, a nationwide database of patients receiving care at Epic EHR sites. Laboring persons with a delivery encounter billing diagnosis of IAI who gave birth to a liveborn singleton by cesarean delivery at 24‐43 weeks were included. The primary exposure was perioperative azithromycin. The primary outcomes were identified from ICD codes and occurred within 6 weeks. They included: 1) a composite of post‐discharge infection (endometritis, wound infection, or other infection), and 2) hospital readmission for infection. We used Poisson regression with fixed effects for delivery department and year to estimate relative risks, adjusting for maternal age, delivery BMI, diabetes, parity, prior cesarean delivery, reaching the second stage, rupture of membranes ≥ 18 hours, and gestational age.

**Results**: The cohort comprised 31,909 persons with IAI; azithromycin was administered to 10,733 (33.6%). There was a rapid increase in use among cesarean births after 2016, from 2.0% in 2013 to 58.7% in 2024 (Figure). The rate of post‐discharge infection was 8.9% without and 7.3% with azithromycin (*p* < 0.001); readmission rates were 2.7 vs 2.1% (*p* < 0.001). In both the unadjusted and adjusted models, azithromycin was associated with lower rates of post‐discharge infection (RR 0.77, 95% CI: 0.69–0.85; aRR: 0.76, 95% CI: 0.69–0.84) and hospital readmission for infection (RR 0.73, 95% CI: 0.60–0.88; aRR: 0.72, 95% CI: 0.59–0.87).

**Conclusion**: Azithromycin administration in laboring persons with IAI undergoing cesarean birth increased >20‐fold after publication of the C/SOAP trial, despite being an exclusion criteria. However, its use was associated with 20‐30% reductions in post‐discharge infection and readmission for infection.

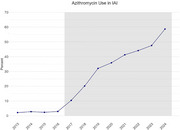

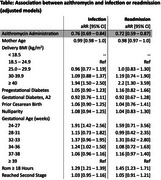

**3:30 PM – 5:00 PM**

## 1279 | **Maternal and Neonatal Outcomes in Pregnant Women With Cardiac Disease: Specialized Versus General Obstetric Care**

Eli Heifetz^1^; Maayan Y. Bas‐Lando^2^; Sarit Helman^3^; Sorina Grisaru‐Granovsky^4^; Amiram Nir^5^; Alexander Ioscovich^5^; Tehila Avitan^6^

^1^The Jerusalem College of Technology, jerusalem, Yerushalayim; ^2^Department of Obstetrics and Gynecology Shaare Zedek Medical Center in Jerusalem Affiliated with the Hebrew University School of Medicine, Shaare Zedek Medical Center, Department of Obstetrics and Gynecology, 12 Bayit Street, Jerusalem, Yerushalayim; ^3^Shaare Zedek Medical Center, shaare zedek medical center, Yerushalayim; ^4^Shaare Zedek Medical Centre affiliated with The Hebrew University, Jerusalem, Yerushalayim; ^5^Shaare Zedek Medical Center, jerusalem, Yerushalayim; ^6^Obstetrics and Gynecology Department, Shaare Zedek Medical Center Affiliated with The Hebrew University, Jerusalem, Yerushalayim

**Objective**: To assess whether management in a specialized multidisciplinary cardiac‐obstetric clinic improves maternal and neonatal outcomes in pregnancies complicated by heart disease, stratified by WHO risk class.

**Study Design**: This retrospective cohort included 439 singleton pregnancies with maternal cardiac disease, categorized as WHO class I‐II or >II‐III. Patients received care in either a dedicated cardiac‐obstetric clinic or general obstetric follow‐up. Maternal characteristics, labor and delivery data, and maternal and neonatal outcomes were compared using t‐tests and Fisher's exact tests.

**Results**: In WHO class I–II pregnancies, patients followed in the specialized clinic were younger (27.9 vs. 30.6 years, *p* = 0.001) and underwent more frequent labor induction (31.6% vs. 18.6%, *p* = 0.018). Fewer required postpartum ICU/CCU admission after vaginal delivery (5% vs. 14%, *p* = 0.049). Rates of maternal cardiac complications and composite maternal adverse outcomes were comparable. Neonatal respiratory complications were more frequent in the specialized group (12.2% vs. 2.1%, *p* = 0.003), while overall composite neonatal outcomes were not significantly different (22.4% vs. 16.2%, *p* = 0.234). In WHO class II–III or IV pregnancies, no significant differences were found in maternal or neonatal outcomes between care settings.

**Conclusion**: In WHO I‐II pregnancies, specialized cardiac‐obstetric care was associated with reduced postpartum critical care needs. Although neonatal respiratory complications were more common in this group, composite neonatal outcomes were comparable. These findings support the role of multidisciplinary care in tailoring management for pregnant women with cardiac disease.

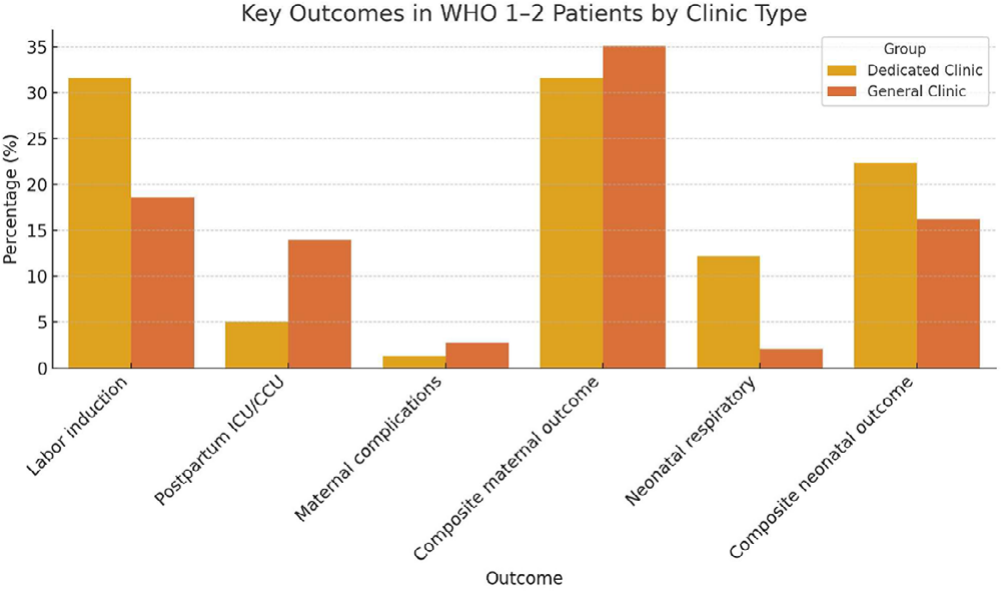

**3:30 PM – 5:00 PM**

## 1280 | **Beyond Recognition: How Different Racial Groups Process Perceived Healthcare Discrimination in Prenatal Care**

Tenisha D. Wilson^1^; Nia Plump^2^; Julia Muller^1^; Traci Johnson^3^; Cheron Phillips^4^; Richelle Smith^4^; Kelly McKay‐Gist^4^; Catalina Montiel^1^; Maura Jones Pullins^5^; Jeannie C. Kelly^6^; Nandini Raghuraman^7^; Melissa Tepe^8^; Candice L. Woolfolk^6^; Shannon Lenze^9^; Ebony Carter^10^

^1^University of North Carolina, Chapel Hill, NC; ^2^EleVATE Collaborative, St. Louis Integrated Health Network, St. Louis, MO; ^3^University of Missouri‐Kansas City, Kansas City, MO; ^4^St. Louis Integrated Health Network, St. Louis, MO; ^5^University of North Carolina, Chapel Hill, Durham, NC; ^6^Washington University School of Medicine, Washington University School of Medicine, MO; ^7^Washington University School of Medicine, St. Louis, MO; ^8^Affinia Healthcare, St. Louis, MO; ^9^Washington University in St. Louis, School of Medicine, Department of Psychiatry, St. Louis, MO; ^10^University of North Carolina, University of North Carolina, NC

**Objective**: To examine the relationship between perceived healthcare discrimination and interpersonal communication quality in prenatal care across racial and ethnic groups using validated instruments.

**Study Design**: Cross‐sectional analysis of baseline data from 171 patients enrolled in a randomized trial of group prenatal care across three Missouri health systems. Perceived healthcare discrimination was measured using the Prenatal Interpersonal Processes of Care Discrimination (PIPC) subscale, and interpersonal communication/empathy with the Consultation and Relational Empathy (CARE) Measure. PIPC scores were grouped into terciles. Race/ethnicity distribution across terciles was assessed with chi‐square tests. Differences in CARE scores across PIPC terciles were evaluated with Kruskal–Wallis tests, and by race/ethnicity using ANOVA with Tukey's HSD post‐hoc comparisons.

**Results**: The sample included 171 patients: 61% Black/non‐Hispanic (BNH) (*n* = 104), 17% Hispanic (*n* = 29), 18% White/non‐Hispanic (WNH) (*n* = 30), and 5% (*n* = 8) another/non‐Hispanic. Spanish was the primary language for all Hispanic participants. Patients in the highest discrimination tercile had significantly lower CARE scores (*p* = 0.03). Discrimination varied by race, with Hispanic patients disproportionately represented in the highest tercile (59%) compared with BNH (28%) and WNH (27%) (*p* < 0.01). Among BNH patients, higher discrimination was associated with significantly lower CARE scores (*p* < 0.01). No significant associations were observed for Hispanic or WNH patients.

**Conclusion**: Higher perceived healthcare discrimination was linked to poorer communication/empathy quality, with the strongest effects among BNH patients. Despite reporting the highest discrimination levels, Hispanic patients did not show reduced CARE scores, suggesting that language and cultural factors may influence how discrimination impacts perceived care quality. Findings underscore the need for targeted bias‐reduction interventions and culturally responsive outcome measures.

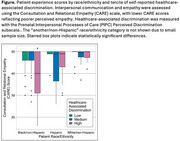

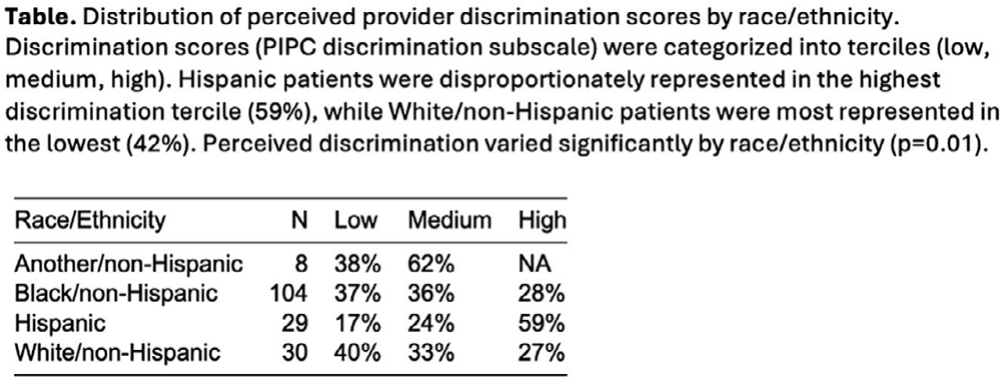

**3:30 PM – 5:00 PM**

## 1281 | **Prepregnancy Risk Factors for Placenta Accreta Spectrum: Understanding Data Structure Using Principal Component Analysis**

Tess EK Cersonsky^1^; Eugenia Alleva^2^; Isabelle C. Band^2^; Angela T. Bianco^2^; Henri M. Rosenberg^3^

^1^Icahn School of Medicine at Mount Sinai, NY; ^2^Icahn School of Medicine at Mount Sinai, New York, NY; ^3^Icahn School of Medicine at Mount Sinai, Icahn School of Medicine at Mount Sinai, NY

**Objective**: Although certain risk factors for placenta accreta spectrum (PAS), such as prior cesarean, uterine surgery, or placenta previa, are well established, many causal factors remain poorly understood. This limits our ability to predict and prepare for PAS deliveries, which carry substantial risk for severe maternal morbidity and mortality. Therefore, we sought to investigate the underlying data in a PAS cohort using principal component analysis (PCA). This information will help build clinical models that focus on important risk factors for PAS.

**Study Design**: This analysis was performed on a cohort built for machine learning analysis comprised of parturients with confirmed PAS and controls. Only variables available prior to the index pregnancy were included.  A case‐matched subset was selected for dimensionality reduction using Kernel PCA. The proportion of variance explained by each principal component was calculated to assess the structure of the data.  Sensitivity analysis was performed to estimate the relative contribution of each variable to the components, providing an approximation of variable importance.

**Results**: Records from 118890 patients were considered, and a subset of 11889 patients and 260 variables were included. Variables fit into 982 components, of which the first 20 explained 50% of the total variance. Component 1 explained 20% of the variance and included several pre‐existing clinical variables, notably, history of cesarean delivery, myomectomy, hysteroscopy, or dilation & curettage.

**Conclusion**: Kernel PCA can help identify underlying data structures in a sample of patients with and without PAS. Most significantly, prior surgical history and gynecologic conditions make up a large portion of the first principal component, suggesting that these factors cluster in a way that is critical for PAS risk stratification.  This information can help reduce dimensions entered into machine learning algorithms, improving ease of use and applicability in clinical settings.

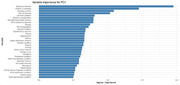

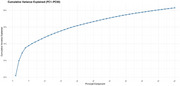

**3:30 PM – 5:00 PM**

## 1282 | **Ergonomics for the Obstetrician During Cesarean Delivery**

Abigail Barger^1^; Rula Atwani^2^; Michelle Barger^3^; Grace Spencer^4^; Tetsuya Kawakita^5^

^1^St. Luke' University Health Network, Bethlehem, PA; ^2^Eastern Virginia Medical School at Old Dominion University, Norfolk, VA; ^3^Autokiniton, Fountain Inn, SC; ^4^EVMS Department of Obstetrics and Gynecology, Macon & Joan Brock Virginia Health Sciences at Old Dominion University, Norfolk, VA; ^5^Eastern Virginia Medical School Department of Obstetrics and Gynecology, Macon & Joan Brock Virginia Health Sciences at Old Dominion University, Norfolk, VA

**Objective**: Surgeon Work Related Musculoskeletal Disorders (WMSD) are prevalent amongst surgeons, with rates ranging from 66% to 94% for open surgery. We aimed to evaluate the ergonomic positions of obstetricians during cesarean deliveries using validated assessment tools and identify risk factors for poor ergonomic positions.

**Study Design**: In this prospective cohort study at a single academic center, residents, fellows, and attendings were photographed at skin incision, uterine incision, and skin closure during cesarean delivery. Postures were scored with the Rapid Entire Body Assessment (REBA) and Rapid Upper Limb Assessment (RULA). Poor ergonomics was defined as REBA ≥ 4 or RULA ≥ 5. Risk factors, including patient characteristics, operative phase, and surgeon demographics, were examined. Adjusted relative risks (aRR) with 95 % confidence intervals (CI) were estimated with modified Poisson regression, clustering by surgeon and patient.

**Results**: We analyzed 191 photographs from 34 cases involving 37 physicians. REBA classified 47 images (24.6 %) and RULA 104 (54.4 %) as poor. Patient characteristics, operative phase, and surgeon demographics are summarized in Table 1. Poor REBA scores were more likely for primary surgeons (aRR 3.68, 95 % CI 1.78–7.62) and residents (PGY 1–2 aRR 2.90, 95 % CI 1.09–7.74; PGY 3–4 aRR 3.95, 95 % CI 1.62–9.59) and less likely with each 5‐year rise in age (aRR 0.70, 95 % CI 0.62–0.79) or 5‐cm gain in height (aRR 0.75, 95 % CI 0.64–0.87) (Table 2). RULA findings echoed these trends, with age aRR 0.90 (95 % CI 0.85–0.95) and height aRR 0.81 (95 % CI 0.74–0.89) protecting against poor posture.

**Conclusion**: Suboptimal posture was pervasive during cesarean delivery, disproportionately affecting younger, shorter, and less‐experienced surgeons. Routine ergonomic monitoring, adjustable operating‐room infrastructure, and simulation‐based training should be integrated into obstetric practice to curb cumulative musculoskeletal strain, prolong surgical careers, and sustain high‐quality patient care.

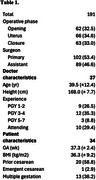

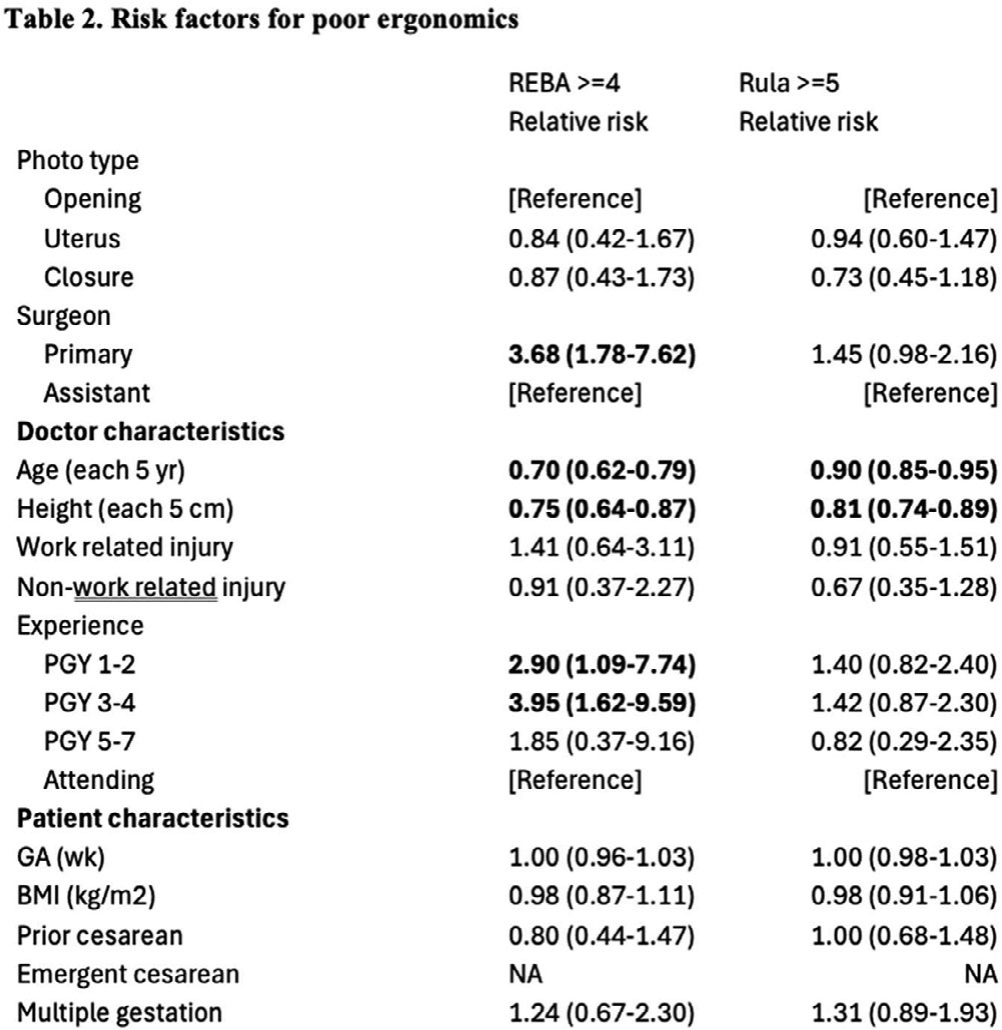

**3:30 PM – 5:00 PM**

## 1283 | **intrapartum Glucose Monitoring for A1GDM: A Randomized Controlled Trial**

Thomas Owens^1^; Erica Glaser^2^; Leonardo Antelo^2^; Johanna A. Suskin^2^; Mia Heiligenstein^3^; Xiteng Yan^2^; Guillaume Stoffels^4^; Zainab Al‐Ibraheemi^5^; Lois Brustman^2^

^1^Icahn School of Medicine at Mount Sinai, Atlanta, GA; ^2^Icahn School of Medicine, Mount Sinai West, New York, NY; ^3^Icahn School of Medicine, Mount Sinai West, New York City, NY; ^4^Icahn School of Medicine at Mount Sinai, New York, NY; ^5^Mount Sinai West, New York City, NY

**Objective**: The objective of this study is to assess the effect of differing intrapartum(IP) glucose monitoring protocols on initial neonatal(neo) blood glucose(BG) level in pregnancies complicated by GDMA1.

**Study Design**: This was a randomized controlled trial of pregnant individuals with singleton gestations and well controlled GDMA1 attempting a vaginal delivery. Written consent obtained and patients were randomly allocated to one of two IP glucose monitoring protocols: Infrequent group(IN) had a single BG measurement on admission vs the frequent group(FR) had BG measured every 4hours(hrs) in latent labor, and every 2hrs in active labor. Exclusion criteria: multiple gestation, non‐compliance, poor glucose control, and/or diabetic fetopathy. The primary outcome was the first neo BG value after delivery. A total of 74 patients were necessary to have 80% power to detect a mean difference of 10mg/dL in glucose values between the two groups. Secondary outcomes included neo BG in the first 24 hrs and neo composite consisting of neo hypoglycemia(HG), NICU admission, shoulder dystocia, and APGAR score at 1min less than 5.

**Results**: From 12/2024 to 5/2025 patients with well controlled GDMA1 were randomized(50 per group). Baseline characteristics of the two groups were comparable(table1). The primary outcome of the first neo BG after delivery was similar between the IN and FR group. The average blood glucose at delivery was 59.5mg/dL(IN) vs 58.5mg/dL(FR) with the mean difference non‐significant (–1.0 mg/dL; 95% CI: −8.3 to 6.8; *p* = 0.78). At 24hrs of life, the mean BG level remained statistically non‐significant, with mean difference of–3.5 mg/dL (95% CI:–8.8 to 1.8;p = 0.20). The incidence of neo HG was similar between the IN and FR groups, 22% vs 26%,(OR 0.92; 95% CI: 0.34 to 2.48;p = 0.87). Composite neo adverse outcome was not statistically significant between the groups(OR 1.52; 95% CI: 0.62 to 3.73;p = 0.36).

**Conclusion**: Our study suggests reducing the frequency of BG monitoring in well controlled GDMA1 patients to an initial value on admission, is acceptable and not associated with an increased risk of neo morbidity.

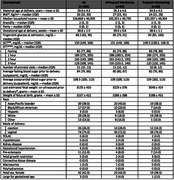

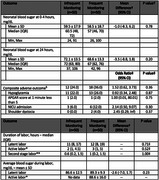

**3:30 PM – 5:00 PM**

## 1284 | **Factors Associated With Pro‐Inflammatory Cervicovaginal Cytokine Milieu Among Gravidas With an Asymptomatic Short Mid‐Trimester Cervix**

Tracy A. Manuck

On behalf of the Eunice Kennedy Shriver National Institute of Child Health and Human Development Maternal‐Fetal Medicine Units Network, Bethesda, MD

University of North Carolina, Chapel Hill, NC

**Objective**: Sociodemographic factors are linked to PTB, but underlying mechanisms are unclear. Inflammation is a key pathway implicated in PTB; it may be detected sub‐clinically in pregnancy. In a prior analysis of the multicenter TOPS RCT of pessary vs. usual care in singletons with a mid‐trimester short CL (≤20 mm) and no prior PTB, we derived a composite pro‐inflammatory cytokine score from 12 cervicovaginal fluid (CVF) cytokines obtained at randomization. Higher scores were associated with PTB and maternal and neonatal infections. Herein, we sought to identify factors associated with CVF inflammation in mid‐pregnancy.

**Study Design**: Secondary analysis of the TOPS RCT; participants with a CVF cytokine score were included. The primary outcome was cytokine category ‐ high (≥75^th^ centile), average (≥25^th^ but <75^th^ centile), or low (<25th centile). The secondary outcome was cytokine score as an ordinal variable. We evaluated the association between demographic and clinical factors present before or at CVF collection and cytokine score category.

**Results**: Of 544 RCT participants, 531 met inclusion criteria: 105 (20%) had low, 305 (57%) average, and 121 (23%) high cytokine scores. Clinical parameters at randomization, including cervical length, presence of intra‐amniotic debris and funneling, and ability to visualize membranes on exam did not vary by cytokine score, nor did reports of vaginal discharge or recent intercourse (Table 1). However, high levels of CVF inflammation were associated with younger age, less education, higher pre‐pregnancy BMI, being single / not living with a partner, receipt of antifungal(s), smoking during pregnancy (Table 1), and vaginal dysbiosis at randomization (Figure 1). These associations were similar when considering the cytokine score ordinally (data not shown).

**Conclusion**: Sociodemographic characteristics have long been recognized as PTB risk factors and are associated with mid‐pregnancy CVF inflammation among asymptomatic gravidas with a short cervical length. These findings suggest that sub‐clinical inflammation may be a key mechanism linking social disadvantage to PTB risk.

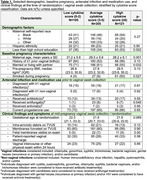

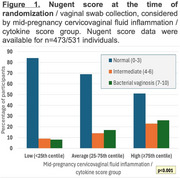

**3:30 PM – 5:00 PM**

## 1285 | **Predictive Value of Maternal sFlt‐1/PlGF Ratio for Adverse Outcomes in Small for Gestational Age Fetuses**

Uri Shemesh^1^; Keren Zloto^2^; Itai Manor^3^; Sharon Kronkop^3^; May Feller^4^; Tal Dadon^4^; Rakeft Yoeli Ullman^5^; Abraham Tsur^5^; Shalom Mazaki‐Tovi^6^; Yoav Yinon^7^; Michal Fishel Bartal^8^; Noa Gonen^9^

^1^The Department of Obstetrics and Gynecology, The Chaim Sheba Medical Center, Ramat‐Gan, Israel, Tel Aviv, Tel Aviv; ^2^sheba medical center, Tel Hashomer, Tel Aviv; ^3^Sheba medical hospital, tel aviv, HaMerkaz; ^4^Sheba medical hospital, Tel Aviv, HaMerkaz; ^5^The Sheba Medical Center, The Sheba Medical Center, HaMerkaz; ^6^The Department of Obstetrics and Gynecology, The Chaim Sheba Medical Center, Ramat‐Gan, Israel, Ramat Gan, HaMerkaz; ^7^Sheba Medical Center, Ramat Gan, Israel, Ramat Gan, HaMerkaz; ^8^Division of Maternal‐Fetal Medicine, Department of Obstetrics, Gynecology and Reproductive Sciences, Sheba Medical Center, Tel Aviv University, Israel, Ramat Gan, HaMerkaz; ^9^Wolfson Medical Center, Holon, HaMerkaz

**Objective**: To determine whether high sFlt‐1/PlGF ratio is associated with hypertensive disorders of pregnancy (HDP), adverse neonatal outcomes, and shorter time to delivery in normotensive individuals presenting with suspected small for gestational (SGA) fetuses.

**Study Design**: This retrospective cohort study included all normotensive individuals with SGA fetuses who underwent sFlt‐1/PlGF testing between August 2023 and May 2024 at a single tertiary center. Individuals with a diagnosis of HDP at the time of testing were excluded. Test results were not used to guide clinical management. Participants were stratified into three groups according to their sFlt‐1/PlGF ratio: 0‐38, 39‐100, and > 100. The rate of HDP, composite adverse neonatal outcomes, and latency to delivery was determine. Diagnostic accuracy (sensitivity, specificity, positive and negative predictive values) was calculated for HDP.

**Results**: Of 141 eligible individuals, 94 (66.7%) had a ratio of 017.7%‐38, 25 () had ratios of  39‐100 group, and 22 (15.6%) had ratios > 100. There was a dose affect relationship between rate of HDP and ratio categories (6.4%, 32%, 54.5%; *p*  < 0.01, respectively), with significant differences between the lowest test and both higher groups. Similar trends were observed in composite neonatal outcome. Median time to delivery decreased with higher ratios (40.7, 29.5, 19.9 days; *p*   =  0.019, respectively), particularly between the low and high ratio groups (*p* = 0.002). (Figure 1) While NPV remained high across categories (0.57–0.88), PPV improved from 0.06 to 0.55 with higher ratios, enhancing rule‐in accuracy for HDP (Figure 2).

**Conclusion**: In normotensive pregnancies complicated by suspected SGA, the sFlt‐1/PlGF ratio is associated with elevated risk of HDP, neonatal morbidity and shorter interval to delivery in a dose‐depended relationship. The findings support the use of this biomarker as a dynamic indicator in individuals with SGA with potential value in risk stratification and clinical decision‐making.

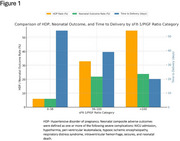

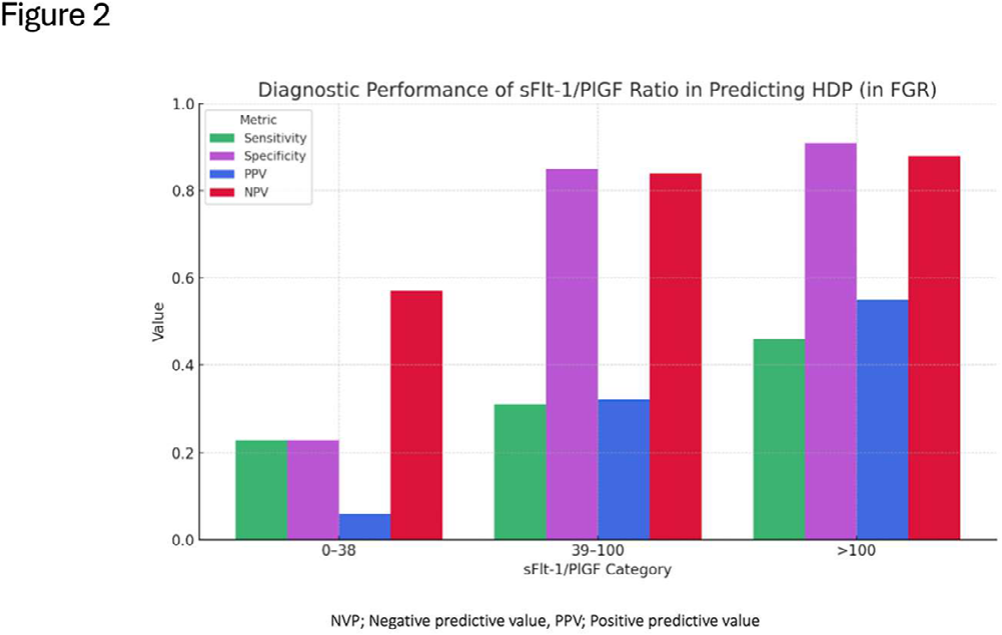

**3:30 PM – 5:00 PM**

## 1286 | **Unmasking Risk in Low/Intermediate sFlt‐1/PlGF Ratios With a Novel Angiogenic Classification**

Valentina Giardini^1^; Caterina Zavettieri^2^; Alessandra Terzaghi^2^; Arianna Pelucchi^2^; Ilaria Re^2^; Leonora Grilli^2^; Marco Casati^3^; Anna Locatelli^4^

^1^Department of Obstetrics, IRCCS San Gerardo dei Tintori Foundation, Monza, Lombardia; ^2^University of Milano‐Bicocca, Milan, Lombardia; ^3^Laboratory Medicine, IRCCS San Gerardo dei Tintori Foundation, Monza, Lombardia; ^4^School of Medicine and Surgery, University of Milano‐Bicocca, Milan, Lombardia

**Objective**: Soluble fms‐like tyrosine kinase‐1 (sFlt‐1) and placental growth factor (PlGF), expressed as the sFlt‐1/PlGF ratio, are widely used for the assessment of placental dysfunction. We aimed to challenge the clinical safety and accuracy of the low and intermediate ratio categories by exploring whether a novel classification based on absolute sFlt‐1 and PlGF levels better improves risk stratification in women with suspected hypertensive disorders (HD) or fetal growth restriction (FGR).

**Study Design**: Retrospective analysis of hospitalized singleton pregnancies beyond 20 weeks complicated by HD, FGR, or both, with low (<38) or intermediate sFlt‐1/PlGF ratios (38–85, or 38–110 if ≥34 weeks) from 2018 to 2022. Pregnancies affected by COVID‐19 or fetal chromosomal abnormalities were excluded. Angiogenic markers were measured using automated assays ‐ Roche. Each patient was classified into one of nine angiogenic groups, based on whether sFlt‐1 and PlGF values were above, within, or below gestational age‐specific reference ranges (10th–90th percentile). Obstetric outcomes were compared across groups.

**Results**: Of the 242 women included, 52% had a low sFlt‐1/PlGF and 48% an intermediate ratio. Based on absolute biomarker values (Table 1), 59% had both markers in range (Category 1); of these, 34% had an intermediate ratio. In contrast, 36% showed abnormal values in Categories 3‐4‐6, including 13% with a low ratio. Compared to Category 1, the combined abnormal marker group had significantly higher rates of adverse outcomes: severe preeclampsia, medically indicated preterm birth, and urgent cesarean sections (all *p* < 0.01–Table 2). Category 6 had the worst profile, with mean gestational age at delivery of 35+4 weeks and the highest incidence of FGR, small‐for‐gestational‐age neonates, and severe hypertensive complications (all *p* < 0.01).

**Conclusion**: A dual‐marker approach uncovers hidden risk within low and intermediate ratio groups, enabling better risk stratification, personalized surveillance, and reduction of unnecessary interventions.

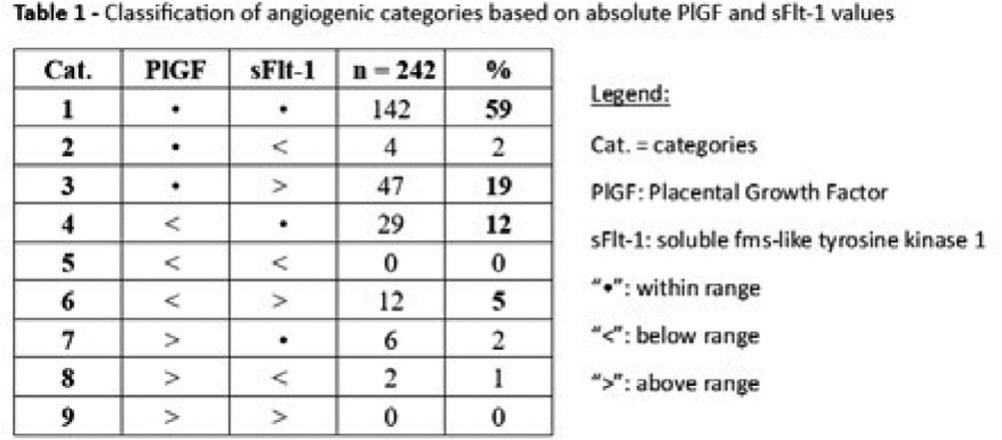

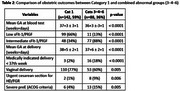

**3:30 PM – 5:00 PM**

## 1287 | **Rising Acute Myocardial Infarction in Pregnancy: National Trends, Risk Factors, and Maternal Health Disparities**

Vanessa Kirschner^1^; Prisca C. Diala^2^; Masoureh Eghbali^3^; Brittany Burton^4^; Yalda Afshar^3^

^1^David Geffen School of Medicine at University of California, Los Angeles (UCLA), LOS ANGELES, CA; ^2^Department of Obstetrics and Gynecology, David Geffen School of Medicine at University of California, Los Angeles (UCLA), Los Angeles, CA; ^3^David Geffen School of Medicine at University of California, Los Angeles (UCLA), Los Angeles, CA; ^4^David Geffen School of Medicine at University of California, Los Angeles (UCLA), Los Angles, CA

**Objective**: To determine recent trends in the incidence of acute myocardial infarction (AMI) during pregnancy and the puerperium in the United States and identify independent sociodemographic, clinical, and obstetric risk factors using a nationally representative cohort, with the goal of informing targeted prevention, improving risk stratification, and guiding the development of integrated care models to reduce maternal morbidity and mortality, particularly among high‐risk populations.

**Study Design**: We conducted a retrospective cohort study using the National Inpatient Sample from 2018–2020. Pregnancy‐related hospitalizations were identified using ICD‐10 codes, and AMI events during pregnancy or the puerperium were included. Survey weights generated national estimates. Multivariable logistic regression identified independent predictors of AMI, adjusting for demographic, clinical, and hospital characteristics.

**Results**: Among 11,079,833 pregnancy‐related hospitalizations, 2225 involved AMI (20.1 per 100,000), with incidence increasing over time (Figure 1). Patients with AMI were more likely to be older, Black, publicly insured, and from the lowest income quartile (Figure 2A). Independent predictors included chronic hypertension (aOR 25.5, 95% CI 15.3–42.6), chronic kidney disease (aOR 11.5, 95% CI 7.2–17.3), coagulopathy (aOR 3.6, 95% CI 2.5–5.1), drug use (aOR 2.4, 95% CI 1.7–3.5), and Black race (aOR 1.5, 95% CI 1.1–2.0). Obstetric complications included eclampsia (aOR 23.2, 95% CI 13.9–38.9), preeclampsia (aOR 2.4, 95% CI 1.8–3.1), and postpartum infection (aOR 6.0, 95% CI 4.5–8.1). Cesarean delivery was associated with decreased odds of AMI (aOR 0.63, 95% CI 0.49–0.81, Figure 2B).

**Conclusion**: The rising incidence of AMI in pregnancy underscores the urgent need for targeted interventions. Our findings highlight key sociodemographic and clinical risk factors, supporting early recognition, improved risk stratification, and integrated obstetric–cardiology care to mitigate severe maternal morbidity and mortality.

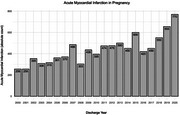

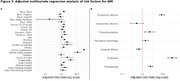

**3:30 PM – 5:00 PM**

## 1288 | **Association Between Timing of Fetal Growth Restriction Diagnosis and Severe Preeclampsia**

Virali Patel^1^; Mariam K. Ayyash^2^; Sascha Wodoslawsky^3^; Lucy M. Vaughn^4^; Amanda Roman^5^; Brooke E. Ciampaglio^4^; Emily F. Rieger^6^; Huda B. Al‐Kouatly^7^

^1^Eunice Kennedy Shriver National Institute of Child Health and Human Development, Philadelphia, PA; ^2^Columbia University Irving Medical Center, New York, NY; ^3^Department of Obstetrics, Gynecology, and Reproductive Science, Icahn School of Medicine at Mount Sinai, New York, NY; ^4^Sidney Kimmel Medical College at Thomas Jefferson Univeristy, Philadelphia, PA; ^5^Thomas Jefferson University, Philadelphia, PA; ^6^Department of Obstetrics and Gynecology, Donald and Barbara Zucker School of Medicine at Hofstra/Northwell, Manhasset, NY; ^7^Division of Maternal‐Fetal Medicine, Department of Obstetrics and Gynecology, Sidney Kimmel Medical College at Thomas Jefferson University, Philadelphia, PA, USA, Philadelphia, PA

**Objective**: To evaluate the temporal relationship between fetal growth restriction (FGR) diagnosis and the onset of severe preeclampsia (PE).

**Study Design**: We conducted a retrospective cohort study of singleton pregnancies complicated by both FGR and severe PE between March 2017 and June 2023 at a single health care system. Pregnancies were included if they had a first‐trimester dating ultrasound, a completed anatomy between 18w0d and 24w0d, and were not affected by major structural or genetic abnormalities. The gestational age at FGR diagnosis and severe PE diagnosis was compared, and the sequence of diagnoses was categorized as (1) FGR preceding severe PE, (2) same‐day diagnosis, or (3) FGR following severe PE. Comparisons between groups were made using chi‐square test for categorical variables and Student's t‐test or Wilcoxon rank‐sum test for continuous variables, as appropriate.

**Results**: Out of 9386 maternal and neonatal charts and ultrasounds reviewed, 998 patients had FGR, 340 patients had severe PE, and 61 patients had both severe PE and FGR. Among the 61 patients with both, the average gestational age at FGR diagnosis was significantly earlier than that at severe PE diagnosis (30.1 ± 5.1 vs 34.8 ± 3.7 weeks, *p* < 0.001). Figure 1 depicts gestational age at FGR diagnosis and severe preeclampsia diagnosis for each patient, sorted by the FGR diagnosis timing. Most patients (52/61, 85.2%) were diagnosed with FGR before the onset of severe PE, with a median interval of 29 days (IQR 13–57). Six patients (9.8%) were diagnosed with FGR and severe PE on the same day, and three patients (4.9%) were diagnosed with FGR after the onset of severe PE (two within 2 days and one 22 days later).

**Conclusion**: In most pregnancies complicated by both FGR and severe PE, FGR is diagnosed four weeks before the onset of severe PE. These findings suggest that early identification of FGR may offer an opportunity for heightened surveillance and earlier recognition of severe PE. Further research is needed to better understand the pathophysiologic relationship between these entities.

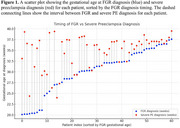

**3:30 PM – 5:00 PM**

## 1289 | **The Impact of Anemia on Fetal Growth Trajectory in Patients With Inflammatory Bowel Disease**

Weirui Xiao^1^; Johanna Bedoy^1^; Mehrnaz Siavoshi^2^; Lorna Kwan^2^; Nirupama Bonthala^1^; Ilina Pluym^1^

^1^David Geffen School of Medicine at University of California, Los Angeles (UCLA), Los Angeles, CA; ^2^Department of Urology, David Geffen School of Medicine at University of California, Los Angeles (UCLA), Los Angeles, CA

**Objective**: To investigate the effect of anemia on longitudinal fetal growth in patients with inflammatory bowel disease (IBD).

**Study Design**: This retrospective cohort study included singleton pregnancies among patients with IBD who delivered at a single academic center from January 2019 to December 2023. Demographic and clinical data was collected. Anemia was defined as a hemoglobin less than 11g/dL at delivery. Estimated fetal weights (EFWs) by Hadlock criteria, obtained from all maternal‐fetal medicine ultrasounds, were collected and plotted against gestational age in days. Fetal growth trajectories were modeled using a fourth root transformation and compared using mixed effects models to account for repeated ultrasound measurements within subjects. Clinical outcomes were compared using Kruskal‐Wallis test or Chi‐squared test.

**Results**: Among 100 deliveries in 89 patients with IBD, 26% (*n* = 26) had anemia upon admission for delivery. Rates of IBD remission at conception (85.1% vs. 88.5%, *p* = 0.80) and disease flares in pregnancy (19.2% vs. 16.2%, *p* = 0.72) were similar between anemic and non‐anemic patients. Neonatal birthweights (3436g ± 371 g vs. 3290g ± 570 g, *p* = 0.21) and rate of neonates born small for gestational age (3.8% vs. 9.5%, *p* = 0.36) did not differ by anemia status. Mean fetal weight gain in grams per day was similar (22.5 vs. 21.2, *p* = 0.15) (Table 1). Mixed effects modeling revealed no significant difference in fetal growth trajectory by anemia status (*p* = 0.230) (Figure 1).

**Conclusion**: Maternal anemia was not associated with altered fetal growth trajectory in pregnancies complicated by IBD.

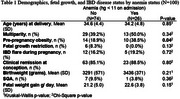

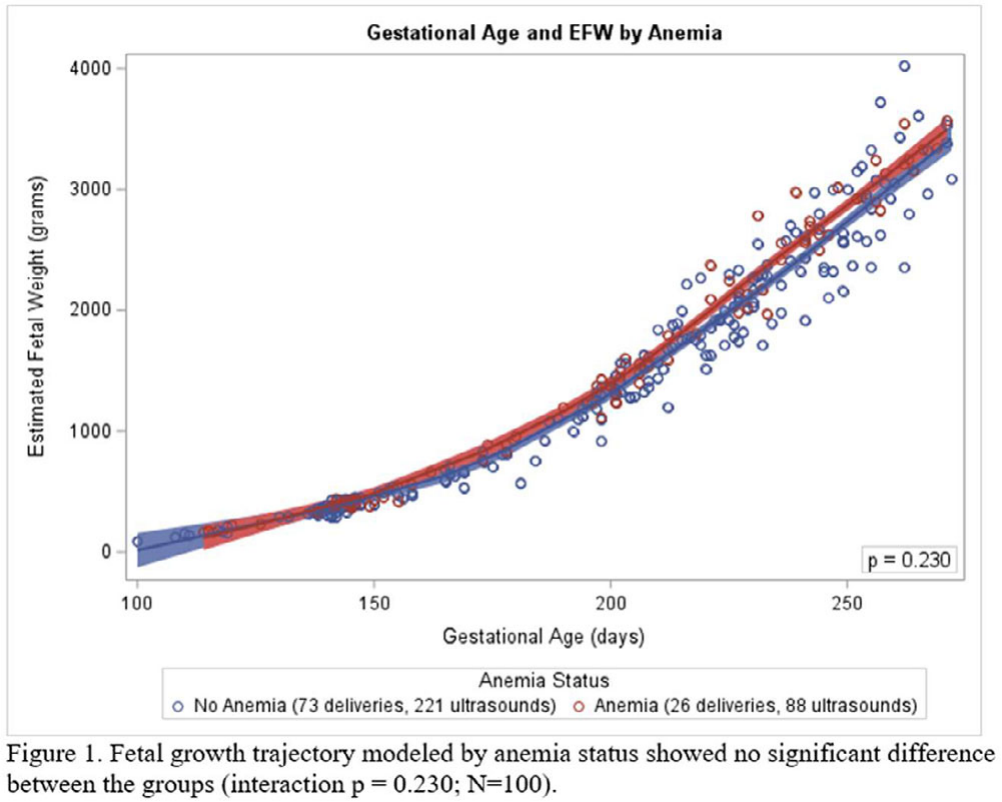

**3:30 PM – 5:00 PM**

## 1290 | **Association of Race and Ethnicity With Adverse Outcomes in Patients With Pre‐Existing Diabetes**

Wendy Tian^1^; Alyssa R. Hersh^1^; Bharti Garg^2^; Aaron B. Caughey^2^

^1^Oregon Health & Science University, Portland, OR; ^2^Oregon Health & Science University, Oregon Health & Science University, OR

**Objective**: The purpose of our study was to evaluate the impact of race/ethnicity on maternal and neonatal outcomes among patients with pre‐existing diabetes (DM).

**Study Design**: We performed a retrospective cohort study among births with pre‐existing DM in California between 2008‐2020. We included singleton, non‐anomalous births with gestational age between 32‐42 weeks. Maternal race/ethnicity was categorized as non‐Hispanic White, non‐Hispanic Black, Hispanic, Asian, and American Indian or Alaska Native (AIAN). Outcomes included cesarean delivery, preterm delivery, NICU admission, macrosomia, hypoglycemia, respiratory distress syndrome (RDS), and jaundice. Statistical analyses were performed utilizing chi squared and multivariable logistic regression with a p‐value of 0.05.

**Results**: There were 54,870 patients with pre‐existing DM included in this study. 17.6% were identified as White, 5.9 % as Black, 65.1% as Hispanic, 10.9% as Asian, and  .57% as AIAN. Black patients with pre‐existing DM were more likely to deliver preterm <37 weeks (aOR 1.48, CI 95% 1.38‐1.59), as well as have infants requiring NICU admission (aOR 1.28, CI 95% 1.19‐1.37) and RDS (aOR 1.38, CI 95% 1.14‐1.66) compared to the white cohort. Hispanic patients with pre‐existing DM were less likely to have infants with RDS (aOR  .78, CI 95% 0.69‐0.88), while AIAN patients were more likely to deliver preterm (aOR 1.48, CI 95% 1.16‐1.86), and have fetal macrosomia (aOR 1.33, CI 95% 1.12‐1.59) compared to the white cohort. Asian patients were less likely to experience adverse outcomes, including preterm delivery <37 weeks (aOR  .74, CI 95%  .68‐.80), macrosomia (aOR  .48, CI 95%  .44‐.52) and RDS (aOR  .79, CI 95%  .66‐.95) compared to the white cohort.

**Conclusion**: Maternal and perinatal outcomes varied by race/ethnicity in patients with pre‐existing DM. Based on our findings, there is an increased risk of adverse outcomes with black and AIAN race in pre‐existing DM patients, while Asian patients had better outcomes. Further investigation should be done to assess structural factors that may contribute to these disparities.

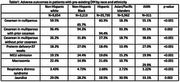

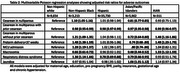

**3:30 PM – 5:00 PM**

## 1291 | **GBS Carriage in Term‐Pregnancies: Concordance of Detection by Prenatal Screening, Culture, and Deep Metagenomic Sequencing**

William L. Riley^1^; Jéssica F. da Conceição Mendonça^2^; Zachary Burcham^2^; Jill M. Maples^3^; Kimberly B. Fortner^4^; Callie F. Reeder^5^; Lindsey Burcham^6^

^1^Georgia Perinatal Consultants, Milton, GA; ^2^University of Tennessee, Knoxville, TN; ^3^University of Tennessee Health Science Center COM Knoxville, Knoxville, TN; ^4^University of Tennessee College of Medicine‐ Knoxville, Knoxville, TN; ^5^UTHSC UTGSM Knoxville, Knoxville, TN; ^6^University of Tennessee, University of Tennessee, TN

**Objective**: Group B Streptococcus (GBS), or Streptococcus agalactiae, is a common component of the vaginal flora but can cause invasive infections in pregnant women, developing fetuses, and neonates. We sought to evaluate concordance between routine prenatal GBS screening and culture‐based detection or deep metagenomic sequencing in term‐pregnant individuals to assess implications for intrapartum antibiotic prophylaxis decision‐making.

**Study Design**: This prospective cohort study enrolled 50 term‐pregnant patients and assessed continuity across standard prenatal GBS screen, and culture‐based analyses, and deep metagenomic sequencing from vaginal swabs collected at the time of admission for labor. After obtaining consent, a sterile speculum examination was performed for vaginal swab collection. Swabs were used to cultivate GBS, confirming identity by full‐length 16S rRNA sequencing, and processed for metagenomic sequencing to detect GBS. Results were compared across all three detection methods and concordance among methods was analyzed to identify GBS colonization.

**Results**: We observed 35.4% (17/48) of individuals had a positive prenatal GBS screen. Culture‐based methods identified viable GBS in 34% (16/47) of patient swabs collected upon admission, while metagenomic sequencing detected reads aligning to GBS in 44% (22/50) of individuals. Concordance between prenatal screening and intrapartum culture was observed in 80% (36/45) of individuals with 24.4% (11/45) positive by both and 55.6% (25/45) negative. Result discordance was observed in 20% (9/45), with 8.9% (4/45) positive by prenatal screening but negative by culture, and 11.1% (5/45) negative by prenatal screening but culture positive. Overall, GBS was detected in 64% (32/50) individuals by one or more methods across this dataset (Figure 1). Metagenomic sequencing conferred higher sensitivity, identifying additional cases that were negative by clinical and culture methods.

**Conclusion**: These findings suggest that a significant proportion of individuals may be treated inappropriately under the current prenatal screening protocols (Figure 2).

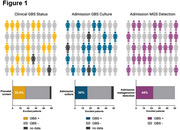

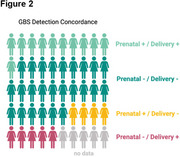

**3:30 PM – 5:00 PM**

## 1292 | **Antepartum vs Postpartum Pulmonary Edema in Preeclampsia: Echocardiographic Findings and Maternal Outcomes**

Wissam Akkary^1^; Ahmed Zaki Moustafa^2^; Megan C. Shepherd^1^; Poyee Tung^3^; Zakaria Doughan^4^; Yossi Bart^1^; Baha M. Sibai^1^

^1^Division of Maternal‐Fetal Medicine, Department of Obstetrics, Gynecology and Reproductive Sciences, McGovern Medical School at UTHealth Houston, Houston, TX; ^2^Division of Maternal‐Fetal Medicine, Department of Obstetrics, Gynecology and Reproductive Sciences, McGovern Medical School at UTHealth Houston, University of Texas ‐ Houston, TX; ^3^Department of Cardiology, McGovern Medical School at UTHealth Houston, Houston, TX; ^4^Department of Obstetrics and Gynecology, McGovern Medical School at UT Health, Houston, Houston, TX

**Objective**: Echocardiographic evaluation in preeclampsia complicated by pulmonary edema may help in establishing pathophysiology and guide management. However, there is limited data comparing findings between antepartum versus postpartum onset of pulmonary edema. Our objective is to compare echocardiographic findings and maternal outcomes between antepartum and postpartum pulmonary edema in patients with preeclampsia.

**Study Design**: This is a retrospective cohort study of patients with preeclampsia complicated by pulmonary edema at a single quaternary center between 2016–2023. Pulmonary edema was defined by clinical and radiographic criteria. Echocardiographic parameters assessing both systolic and diastolic function were compared according to timing of onset of pulmonary edema: antepartum vs postpartum. Data were abstracted by chart review, and ECHO findings were reviewed by a cardiologist. The primary outcome was echocardiographic findings. The secondary outcome was a composite maternal adverse outcome (CMAO), including ICU admission, need for respiratory support, and death.

**Results**: A total of 71 patients were studied. Pulmonary edema was diagnosed in 30 patients (41.7%) in antepartum period and in 42 (58.3%) postpartum. Antepartum cases were more likely to have chronic hypertension, deliver at an earlier gestational age, undergo cesarean delivery, and have a lower fluid balance at delivery (Table 1). Following adjustment for chronic hypertension, CMAO was significantly higher in the postpartum group (aOR 3.93; 95th CI 1.12‐ 13.85). Table 2 compares ECHO findings between the 2 groups. Postpartum pulmonary edema was associated with worse systolic function, including lower fractional shortening (27.7% vs. 32.3%, *p* = 0.03) and lower stroke volume (63.0 mL vs. 76.0 mL, *p* < 0.01).

**Conclusion**: In preeclampsia complicated by pulmonary edema, postpartum onset was associated with worse systolic function and a higher CMAO rate compared to antepartum onset. These findings suggest distinct pathophysiologic mechanisms may underlie pulmonary edema in preeclampsia, highlighting the need for targeted management strategies.

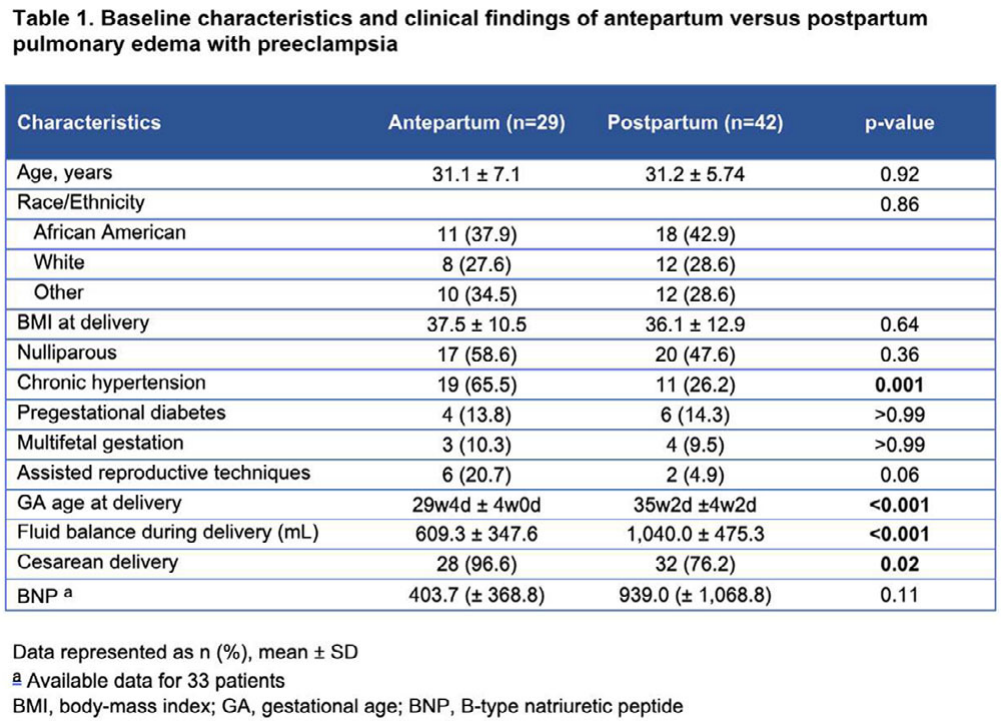

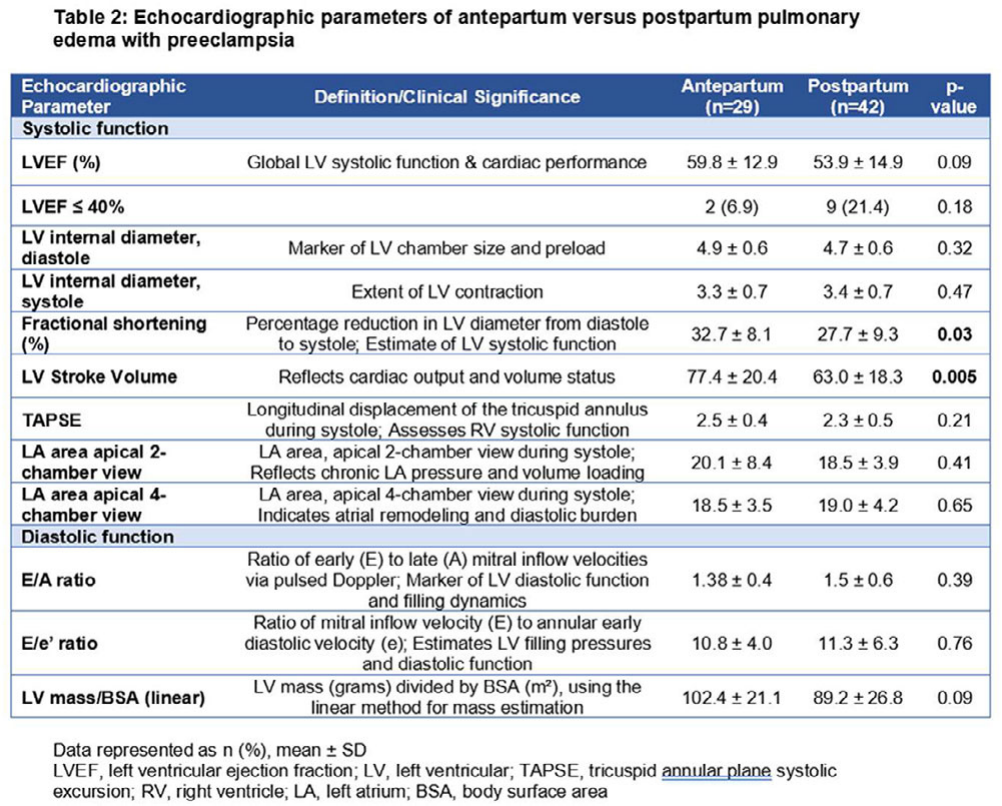

**3:30 PM – 5:00 PM**

## 1294 | **Rethinking Advanced Maternal Age: Stratified Analysis of Severe Maternal Morbidity in Individuals Aged 35–69**

Yanling Dong; Shriddha Nayak; Alexandra D. Forrest; Arthur Jason Vaught; Marika Toscano

Johns Hopkins University, Baltimore, MD

**Objective**: With delayed childbearing and increasing use of in vitro fertilization, pregnancies among individuals approaching perimenopause and menopause are more common. Advanced maternal age (AMA), broadly defined as ≥35 years, encompasses a heterogeneous population with varying risk profiles. This study evaluated the odds of severe maternal morbidity (SMM) across stratified AMA age groups.

**Study Design**: This retrospective cohort study used TriNetX, a global network of de‐identified electronic health records from 106 healthcare organizations. Pregnant individuals aged 12–69 years from July 2020 to July 2025 were included. Eight cohorts were constructed: one reference group (age 20–34) and seven AMA groups (ages 35–39 through 65–69). Cohorts were propensity score matched 1:1 by race, ethnicity, hypertension, pregestational diabetes, obesity, asthma, cardiovascular disease, chronic kidney disease, tobacco use, and multiple gestation. SMM was defined using the CDC's 21 indicators and ICD‐10 codes. The primary outcome was adjusted odds ratio (aOR) for composite SMM within one year of pregnancy; secondary outcomes included aORs for individual indicators.

**Results**: The unmatched reference cohort included 1,601,993 pregnancies. Before matching, unadjusted odds of composite SMM were markedly elevated among individuals aged 50–69 (OR 3–8). After matching, the aOR of composite SMM increased progressively with age (Table 1). Higher age was also associated with increased odds of specific indicators (Figure 1). Puerperal cerebrovascular disorders were strongly associated with age ≥50.  Myocardial infarction, hysterectomy, and venous thromboembolism showed modest associations and increasing effect sizes with age. Indicators with low event rates were excluded from the heatmap.

**Conclusion**: AMA is not a uniform risk category—odds of composite SMM progressively increase with age. The significantly elevated unadjusted odds of SMM among individuals ≥50 likely reflect cumulative comorbidities and may better capture real‐world risk. These findings support individualized counseling and care planning for older pregnant individuals.

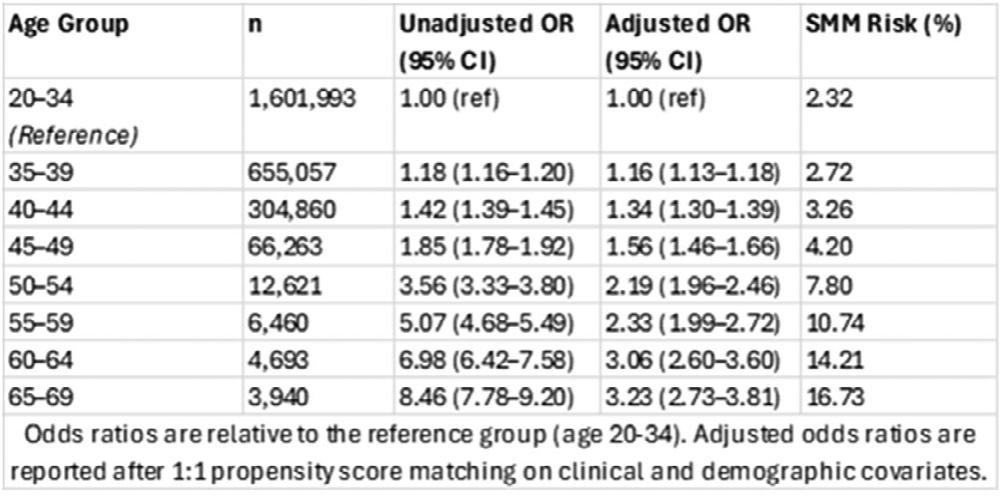

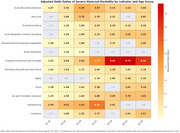

**3:30 PM – 5:00 PM**

## 1295 | **Amniotic IL‐6 as a Predictor of Intra‐Amniotic Infection: A Retrospective Cohort Study**

Yiny Y. Torres Valencia^1^; David Boada^2^; Clara Murillo Bravo*^3^; Sílvia Ferrero^4^; Aleix Fabregat^5^; Berta Fidalgo^5^; Montse Palacio*^2^; Teresa Cobo^6^

^1^Hospital Clínic de Barcelona, Hospital Clínic de Barcelona, Catalonia; ^2^Hospital Clínic Barcelona, BARCELONA, Catalonia; ^3^Hospital Clínic de Barcelona, HOSPITAL CLINIC BARCELONA / BARCELONA, Catalonia; ^4^BCNatal–Barcelona Center for Maternal Fetal and Neonatal Medicine Hospital Sant Joan de Déu and Hospital Clínic de Barcelona, University of Barcelona, BARCELONA, Catalonia; ^5^Hospital Clínic, Hospital Clínic, Catalonia; ^6^Hospital Clínic de Barcelona, Hospital Clinic (Barcelona), Catalonia

**Objective**: Intra‐amniotic infection is the most common origin of spontaneous preterm delivery at early gestational ages. Main concern is the need to perform an amniocentesis for this indication. We aimed to compare early markers of infection in maternal blood and amniotic fluid in patients with Threatened Preterm Labor (PTL), Preterm Premature Rupture of Membranes (PPROM) and cervical incompetence.

**Study Design**: A retrospective cohort study was performed in pregnant patients admitted with a diagnosis of PTL, PPROM or cervical incompetence between 2018 and 2025 who underwent amniocentesis to rule out the occurrence of intra‐amniotic infection. Infection was confirmed by positive amniotic fluid cultures for aerobes, anaerobes, genital mycoplasma, or positive 16S rRNA gene sequencing. Biomarkers analyzed included maternal serum C‐reactive protein (CRP) and leukocyte count, amniotic fluid Interleukin (IL)‐6, and glucose concentrations. Amniotic fluid IL‐6 was assessed using an automated electrochemiluminescence immunoanalyzer providing results in a few minutes. Non‐parametric tests were used for group comparisons, and ROC curves assessed diagnostic performance.

**Results**: The study included 74 PTL, 59 PPROM, and 29 patients with cervical incompetence. Intra‐amniotic infection was present in 54 patients (25 PTL, 20 PPROM, 9 cervical incompetence). An amniotic fluid IL‐6 > 13.9 ng/mL showed excellent diagnostic performance with an AUC of 0.93 (95% Confidence interval: 0.89–0.97), 88% sensitivity, and 89% specificity. Amniotic fluid glucose also showed good diagnostic performance (AUC: 0.90, 95% CI 0.84 ‐ 0.96), while accuracy of maternal CRP (AUC: 0.79 95% CI 0.71 ‐ 0.86) and leukocytes (AUC: 0.56, 95% CI 0.46‐ 0.66) were limited.

**Conclusion**: Amniotic fluid IL‐6 showed the best diagnostic performance to predict intra‐amniotic infection in patients with PTL, PPROM, and cervical incompetence, outperforming traditional inflammatory markers in maternal blood. This rapid point‐of‐care might help in early diagnosis and clinical decision‐making in these high‐risk pregnancies.

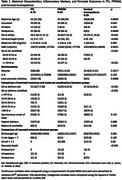

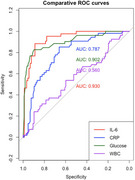

**3:30 PM – 5:00 PM**

## 1296 | **Mode of Scheduled Delivery for Nulliparous Individuals With Class III Obesity**

Yossi Bart; Michal Fishel Bartal; Hector Mendez‐Figueroa; Sean C. Blackwell; Baha M. Sibai

Division of Maternal‐Fetal Medicine, Department of Obstetrics, Gynecology and Reproductive Sciences, McGovern Medical School at UTHealth Houston, Houston, TX

**Objective**: To compare maternal and neonatal outcomes between labor induction and cesarean delivery (CD) without labor among term nulliparous individuals with class III obesity (body mass index [BMI] ≥ 40 kg/m^2^).

**Study Design**: A secondary analysis of the Assessment of Perinatal Excellence (APEX) database, including all nulliparous singleton deliveries ≥ 37 weeks with BMI ≥ 40 kg/m^2^ at delivery. Individuals with spontaneous labor or fetal malformations were excluded. The primary outcome was a composite maternal adverse outcome (CMAO) that included 12 hemorrhagic, operative, and infectious complications, along with venous thromboembolism and death. The composite neonatal adverse outcome (CNAO) included mortality and key morbidity outcomes. Multivariable logistic regression was used to calculate adjusted odds ratio (aOR) with 95% confidence intervals (CI).

**Results**: Out of 115,502, a total of 1922 (2%) individuals met inclusion criteria: 305 (16%) underwent primary CD without labor, while 1617 (84%) underwent labor induction. Induced individuals were younger, had lower rates of diabetes mellitus, and higher rates of alcohol use and later gestational age at delivery. Of those induced, 893 (55%) ultimately had an indicated CD (Figure). Among those who delivered vaginally, 99 (14%) ended up with a 3^rd^/4^th^ degree tear or shoulder dystocia. Compared with CD without labor, the CMAO rate was higher following induction (aOR 2.35, 95% CI 1.21‐4.57; Table). In a sensitivity analysis excluding 3^rd^/4^th^ degree tears, CMAO rates did not differ (aOR 1.32, 95% CI 0.66‐2.62). Induction was associated with higher rates of neonatal admission to intensive or intermediate care (aOR 1.59, 95% CI 1.05‐2.42); the CNAO rate did not differ between groups (aOR 1.60, 95% CI 0.95‐2.71).

**Conclusion**: In term nulliparas with class III obesity, labor induction led to CD in over 50% of the cases and was associated with a higher CMAO rate compared to CD without labor. These findings support individualized counseling and suggest the need for a comparative prospective study evaluating the mode of scheduled delivery in this population.

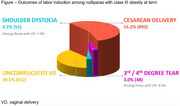

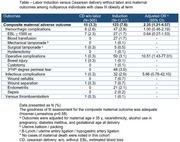

**3:30 PM – 5:00 PM**

## 1297 | **The Utility of Comprehensive Genomic Testing in Families With Stillbirth due to Fetal Disorders**

Yuya Tanaka^1^; Yoshifumi Kasuga^1^; Mamiko Yamada^2^; Koki Shigematsu^1^; Yushi Abe^1^; Naotsugu Ishikawa^1^; Yasuhiko Ogata^1^; Keisuke Akita^1^; Yuka Fukuma^1^; Junko Tamai^1^; Toshimitsu Otani^1^; Marie Fukutake^1^; Satoru Ikenoue^1^; Hisato Suzuki^2^; Fuyuki Miya^2^; Noboru Inagaki^3^; Kenjiro Kosaki^2^; Mamoru Tanaka^1^

^1^Department of Obstetrics and Gynecology, Keio University School of Medicine, Shinjuku‐ku, Tokyo; ^2^Center for Medical Genetics, Keio University School of Medicine, Shinjuku‐ku, Tokyo; ^3^Saint Women's Clinic, Urawa‐ku, Saitama

**Objective**: The Initiative on Rare and Undiagnosed Diseases (IRUD) for postnatal children/adults has achieved a high 47% diagnosis rate with next‐generation sequencing such as whole exome sequencing (WES). In contrast, prenatal genetic testing for families with stillbirth due to fetal disorders has been mostly limited to G‐band karyotyping and microarray analysis, leaving many cases undiagnosed. We aimed to evaluate the utility of comprehensive genomic analysis in fetuses that were electively terminated based on ultrasound findings or resulted in intrauterine fetal death (IUFD).

**Study Design**: With Ethics Committee approval, we performed comprehensive genomic analysis (WES plus detailed copy number analysis of exome data) using fetal appendages (e.g., umbilical cord blood, cord, or villi) and parental blood.

**Results**: Twenty families (including 4 elective terminations and 16 IUFD cases) were enrolled from June 2023 to July 2025, and molecular diagnoses were obtained in ten families (50%). Four involved chromosomal abnormalities: trisomy 18, trisomy 13, monosomy X, trisomy 22. The remaining six were monogenic disorders: three autosomal dominant (Coffin‐Siris syndrome [*ARID1A*], noncompaction cardiomyopathy [*TPM1*], and renal coloboma syndrome [*PAX2*]) and three autosomal recessive (Fukuyama muscular dystrophy/Ciliopathy [*FKTN*/*CEP164*], Nephronophthisis [*NPHP4*] and a likely embryonic lethal disorder [*CES5A*]). In genetic counseling, recurrence risk was low in cases with chromosomal abnormalities and *de novo* monogenic disorders, while a 25% risk was explained in autosomal recessive cases, along with options such as prenatal and preimplantation genetic testing.

**Conclusion**: Molecular diagnoses were identified in 50% (10/20) of cases involving elective termination based on ultrasound findings or IUFD. Comprehensive genomic analysis is effective for genetic counseling, clarifying recurrence risks in subsequent pregnancies, and offering psychological support to families. It may also contribute to the identification of novel embryonic lethal genes, deepening our understanding of the genetic mechanisms underlying fetal death.

